# Evolutionary significance of amino acid permease transporters in 17 plants from Chlorophyta to Angiospermae

**DOI:** 10.1186/s12864-020-6729-3

**Published:** 2020-06-05

**Authors:** Chao Zhang, Nana Kong, Minxuan Cao, Dongdong Wang, Yue Chen, Qin Chen

**Affiliations:** 1 State Key Laboratory of Crop Stress Biology for Arid Areas, College of Agronomy, Northwest A&F University, Yangling, Shaanxi 712100 People’s Republic of China; 2 College of Food Science and Engineering, Northwest A&F University, Yangling, Shaanxi 712100 People’s Republic of China

**Keywords:** *AAP* family, Evolution, Sequencing plants, Phylogenetic analysis, Duplication events

## Abstract

**Background:**

Nitrogen is an indispensable nutrient for plant growth. It is used and transported in the form of amino acids in living organisms. Transporting amino acids to various parts of plants requires relevant transport proteins, such as amino acid permeases (*AAP*s), which were our focus in this study.

**Results:**

We found that 5 *AAP* genes were present in Chlorophyte species and more *AAP* genes were predicted in Bryophyta and Lycophytes. Two main groups were defined and group I comprised 5 clades. Our phylogenetic analysis indicated that the origin of clades 2, 3, and 4 is Gymnospermae and that these clades are closely related. The members of clade 1 included Chlorophyta to Gymnospermae. Group II, as a new branch consisting of non-seed plants, is first proposed in our research. Our results also indicated that the *AAP* family was already present in Chlorophyta and then expanded accompanying the development of vasculature. Concurrently, the *AAP* family experienced multiple duplication events that promoted the generation of new functions and differentiation of sub-functions.

**Conclusions:**

Our findings suggest that the *AAP* gene originated in Chlorophyta, and some non-seed *AAP* genes clustered in one group. A second group, which contained plants of all evolutionary stages, indicated the evolution of *AAP*s. These new findings can be used to guide future research.

## Background

With the evolution of organisms being shaped by local conditions, this provides key information for understanding plants’ appearance and reproduction characteristics. The transition from aquatic to terrestrial environments presents challenges accompanied by physiological and genetic adaptations [1]. As the ancestors of plants, algae play an important role in plant evolution. They are typically water-living and are also closely related to land plants [2]. Following the evolutionary history of plants, the presence of transcription factor gene families significantly increased over evolutionary time [3]. This explosive growth is due to dramatic changes in the environment which result in some new transcription factor families appearing or the enhancement of family members due to adaptation new ecosystems [4, 5].

Thus far, research has been limited to the evolution of transcription factors in the plant kingdom [6]. However, to improve our understanding of the evolution of plant genes, early plants and their ancestors should also be investigated. Fortunately, some early plant species have been sequenced, including various alga, moss, and some other species. Interestingly, an early plant, *Marchantia polymorpha*, has a different level of transcription factor diversity compared with other land plants [7]. Following the evolutionary history of transcription factor families, we can also speculate their earliest function and importance in plants.

The basic conditions for plant growth and development are sunlight, water, and soil. Leaves can be used for photosynthesis to produce organic matter while roots absorb water and nutrients for developmental. Nitrogen is one of the most important nutrients for plant growth and it is required in many different compounds. Nitrogen mainly exists in the form of amino acids in plants, which assimilates within roots and leaves and is transported in the phloem to other organs [8]. To achieve this, amino acid compounds move into the phloem of minor veins in leaves. In roots, amino acids are transported through the xylem [9]. The root cells intake of amino acids is dependent on integral membrane transporter proteins [10]. Many of the proteins which were annotated may facilitate amino acid transport in plants. The two families that associate with these transporters in plants are the amino acid-polyamine-choline (*APC*) family and the amino acid/auxin permease (*AAAP*) family [11, 12]. The *AAAP* family consists of 6 main subgroups, lysine-histidine-like transporters (*LHT*s), amino acid permeases (*AAP*s), proline transporters (*ProT*s), γ-aminobutyric acid transporters (*GAT*s), auxin transporters (*AUX*s) and aromatic and neutral amino acid transporters (*ANT*s) [13, 14]. This large family is found in plants, animals, and fungi.

As one of the amino acids translocators, the *AAP* subfamily has been analyzed in *Arabidopsis thaliana* (8 proteins), *Oryza sativa* (19 proteins), and other plants [15]. Each protein contains an amino acid transporter (*Aa_trans*; PF01490) domain and solute carrier families 5 and 6-like superfamily, which includes the solute-binding domain of SLC5 proteins, SLC6 proteins and NCS1 transporters [16]. The function of *AtAAP1* is to regulate the absorption of amino acids in the endosperm [17], whereas *AtAAP2* transports amino acids from the xylem to the phloem [8], *AtAAP3* is mainly responsible for the absorption and transport of amino acids in the vascular tissue of the root [18], and *AtAAP6* and *AtAAP8* effectively transport neutral acidic amino acids [19]. All *AtAAP*s are located in the plasma membrane [20]. The function of *AAP* genes has also been reported in various plants, such as *Solanum tuberosum* and *Vicia narbonensis*, amongst others [21–23].

The function of *AAP* genes in *A. thaliana* has been thoroughly investigated but only Tegeder and Ward showed the molecular evolution of plant *AAP*s and *LHT*s [13]. This research incorporated many early plant species, which includes red algae, green algae (Chlorophytes and Charophytes), basal non-vascular (*Physcomitrella patens*), non-seed vascular (*Selaginella moellendorffii*), and vascular land plants (eudicots and monocots [13];. According to their study, the *AAP*s of 14 species were identified to indicate the homologs and construct a phylogenetic tree to explain the evolution relationship. In our study, we will identify some new sequencing species which include Chlorophyta, Bryophyta, lycophytes, Gymnospermae and Angiosperms.

In the present study, we will identify the *AAP*s in each evolutionary stage and analyze the protein characteristics, structures, phylogenetic relationships, and gene ontology (GO) annotations of these genes to explain the evolution of *AAP*s in the plant kingdom. Further, the characteristics of *AAP*s will be explored and discussed.

## Results

### Analysis of *AAP* proteins

To perform a phylogenetic analysis of *AAP* proteins in plants, we identified putative *AAP* proteins using the plant sequences listed below as a reference. Combining the sequence data from Tegeder and Ward [13] and Romani et al. [24], 17 plant species were selected, including Chlorophyta (Trebouxiophyceae: *Coccomyxa subellipsoidea*; Chlorophyceae: *Dunaliella salina*, *Volvox carteri, Micromonas pusilla*, *Micromonas sp.*, *Ostreococcus lucimarinus, Chlamydomonas reinhardtii*), Bryophyta (*M. polymorpha*, *Sphagnum fallax*, *Physcomitrella patens*), lycophytes (*S. moellendorffii*), Gymnospermae (*Picea abies*), and angiosperms (*Amborella trichopoda, A. thaliana, S. tuberosum, Zea mays, O. sativa*; Table 1).

**Table 1:** The number of *AAP*s, clade, and genetic characteristics of AAP genes in 17 different stage plants

	Number of ***AAP***s	Number of Group I						Number of Group II	Tandem duplication (pairs)	Segmental duplication (pairs)
		Clade 1A	Clade 1B	Clade 2	Clade 3	Clade 4	Clade 5			
**Chlorophyta**										
*Volvox carteri*	**0**									
*Chlamydomonas reinhardtii*	**0**									
*Dunaliella salina*	**0**									
*Micromonas pusilla*	**0**									
*Micromonas sp.*	**0**									
*Ostreococcus lucimarinus*	**0**									
*Coccomyxa subellipsoidea*	**5**	**1**						**4**	**0**	**0**
**Bryophyta**										
*Sphagnum fallax*	**30**		**4**					**26**	**6**	**0**
*Physcomitrella patens* [13]	**12**		**2**					**10**	**0**	**0**
*Marchantia polymorpha*	**10**	**4**	**1**					**5**	**1**	**0**
**Lycophyte**										
*Selaginella moellendorffii* [13]	**15**	**2**	**2**					**11**	**4**	**1**
**Gymnospermae**										
*Picea abies*	**9**		**2**		**4**	**2**	**1**		**0**	**0**
**Angiosperm**										
**Amborella**										
*Amborella trichopoda*	**16**			**2**	**1**	**1**	**3**	**9**	**3**	**0**
**Eudicots**										
*Arabidopsis thaliana* [13]	**8**			**3**	**1**	**4**			**0**	**5**
*Solanum tuberosum* [25]	**8**			**2**	**2**	**4**			**1**	**1**
**Monocots**										
*Zea mays* [26]	**22**			**4**	**3**	**10**	**5**		**0**	**6**
*Oryza sativa* [27]	**19**			**4**	**3**	**8**	**4**		**5**	**2**

**Fig. 1:**
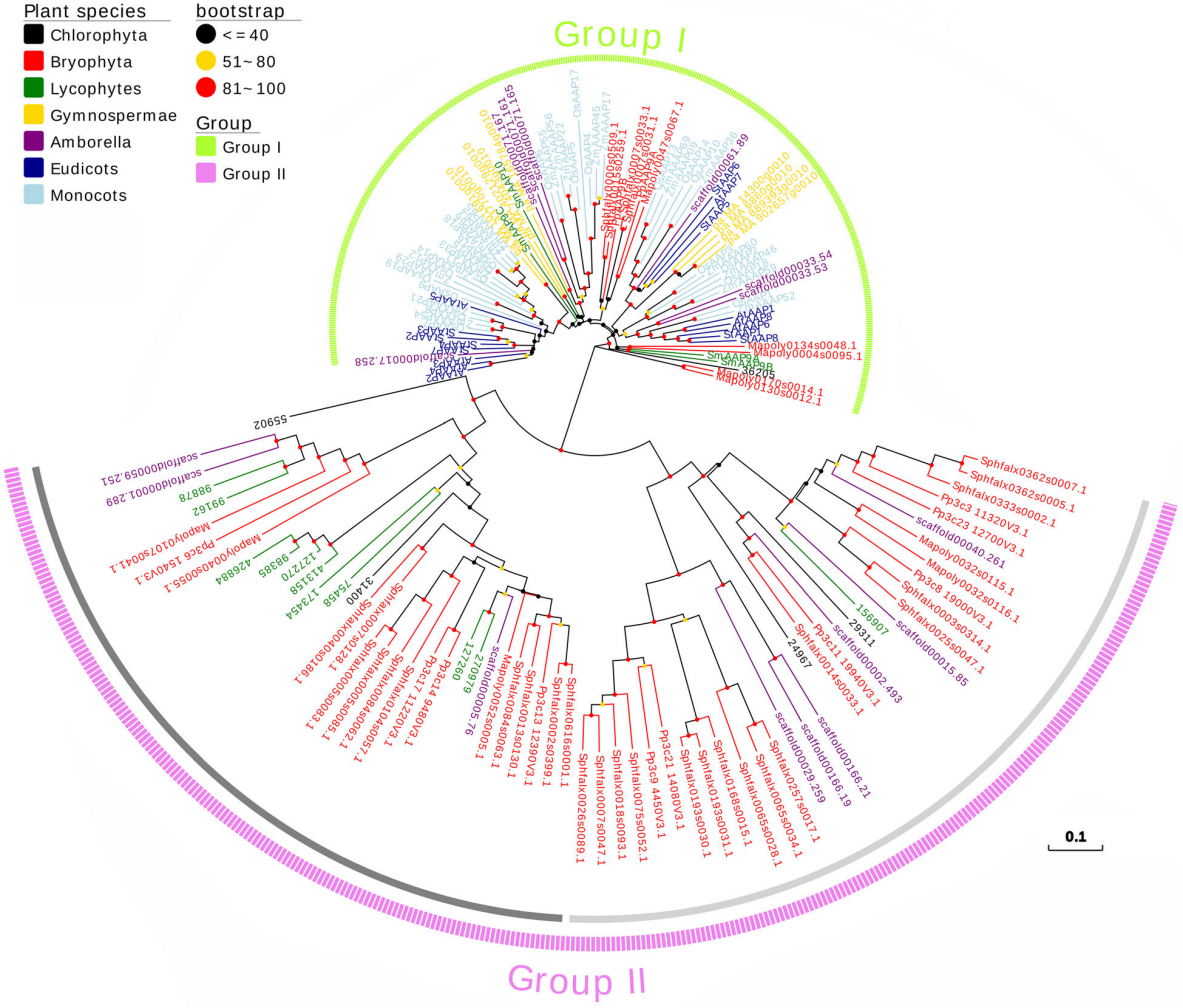
Phylogenetic tree of *AAP* proteins. The unroot tree contains 154 protiens from Chlorophyta to Angiosperms and 7 different colors indicate *AAP*s from different stages. The protein distribution can easily divide into 2 main parts which were showed by greenyellow and violet colors’ dash lines and the group II might be divided into 2 subgroups indicated by gray and lightgray lines, respectively

In total, 210 proteins were blasted, with some genes having more than one transcript and we thus only selected the primary one. Through the analysis of predicted proteins, 154 proteins had *Aa_trans* or *SLC5–6-like_sbd* superfamily which consisted mainly of sequences to recognize the *AAP* proteins (Additional file 13). Only 5 *AAP*-like proteins were predicted in *C. subellipsoidea* from 7 different chlorophyte species we searched were predicted AAP proteins and the amount of AAP proteins in *S. fallax* were larger than others. Each tracheophyte speices also predicted AAP proteins, either. In order to visualize the groups of *AAP* proteins in plants at various stages, we used 7 different colors to distinguish the plant species and noted the plant species (Fig. 1) and the number of *AAP* proteins (Table 1) in each group.

We have provided some information about *AAP* proteins, which included the protein length, domain location and number of transmembrane domains and exons (Additional file 1). While for the most part exons numbered 6–8, in some species only 1 exon was identified and in *C. subellipsoidea* more than 10 exons were identified. In general, the number of exons was relatively stable in all plants. A greater number of exons more short sequences being constructed and the length of the sequence was not correlated with the number of exons.

**Fig. 2:**
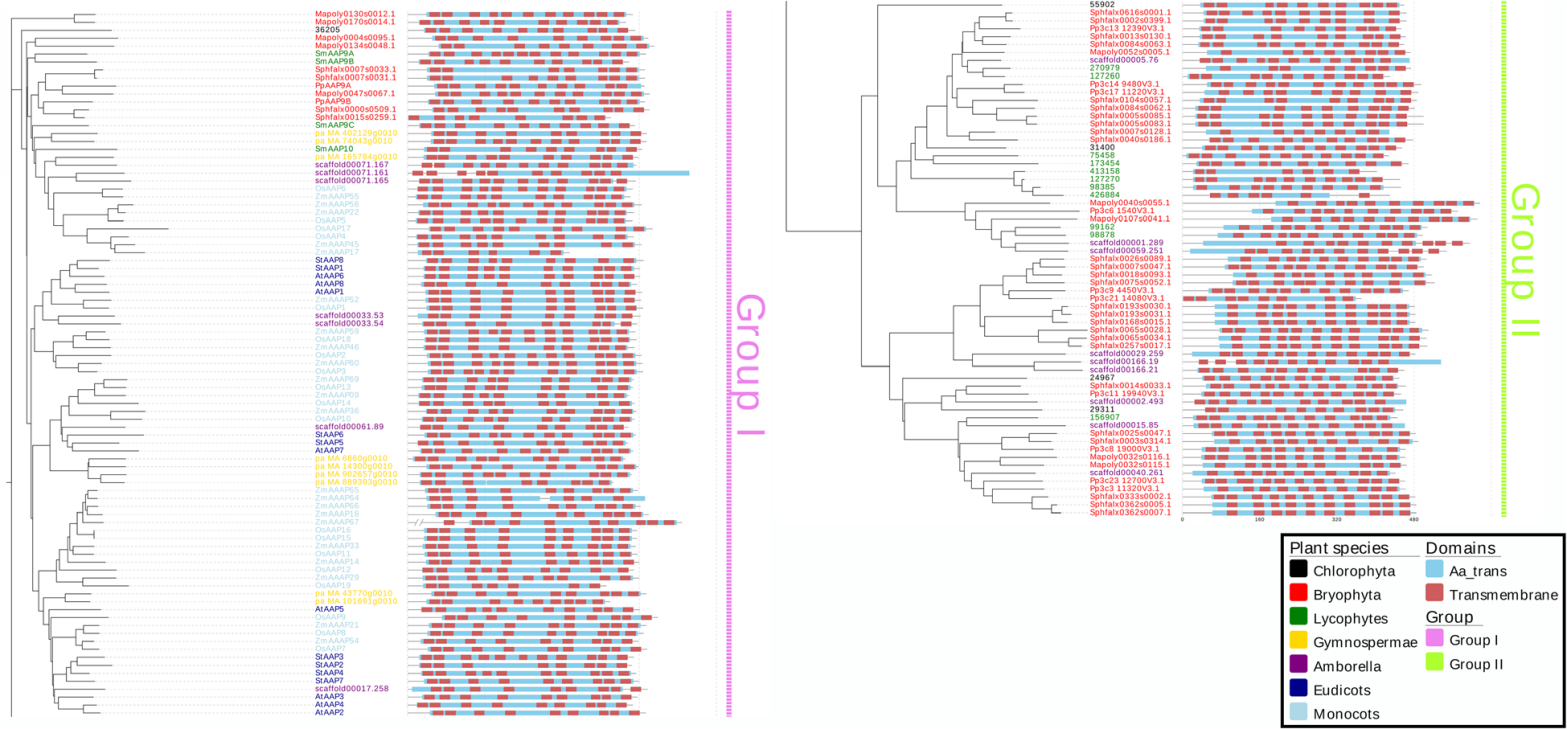
The division of whole *AAP* proteins. The tree shows that the 2 main groups are divided; group I is represented by violet and group II by green. It can be inferred from the phylogenetic tree that the two groups are genetically. Eleven plants in 5 main different evolutionary stages were used to build the phylogenetic tree. The main domain, *Aa_trans* and transmembrane structure. The blue bar in each protein is the location and numbers of *Aa_trans* and the red boxes are transmembrane structures. There are no distinct differences between group members

The *AAP* protein family as an amino acid transporter had specific repetitive sequences. We predicted the location of the main motif, *Aa_trans* domain, and the number of transmembrane domains in each protein. The e-value was set − 5 to confirm that the domain showed all of the proteins in these two kinds of motifs. Most proteins had one main *Aa_trans* domain, except for *Pp3c21_14080V3.1, 413,158, pa_MA_889393g0010, ZmAAAP17, ZmAAAP64,* and *OsAAP19*, which had 2 domains which were all incomplete, and *pa_MA_101691g0010,* which had 3 segments. Six to twelve transmembrane domians were predicted in each protein. Among them, *SmAAP9A* contained 12 domains, *413,158, 426,884* and *ZmAAAP17* each contained 6 transmembrane domians (Additional files 1 and 10) and we showed all transmembrane domians by Fig. 2.

### Phylogenetic analysis of *AAP*

**Fig. 3:**
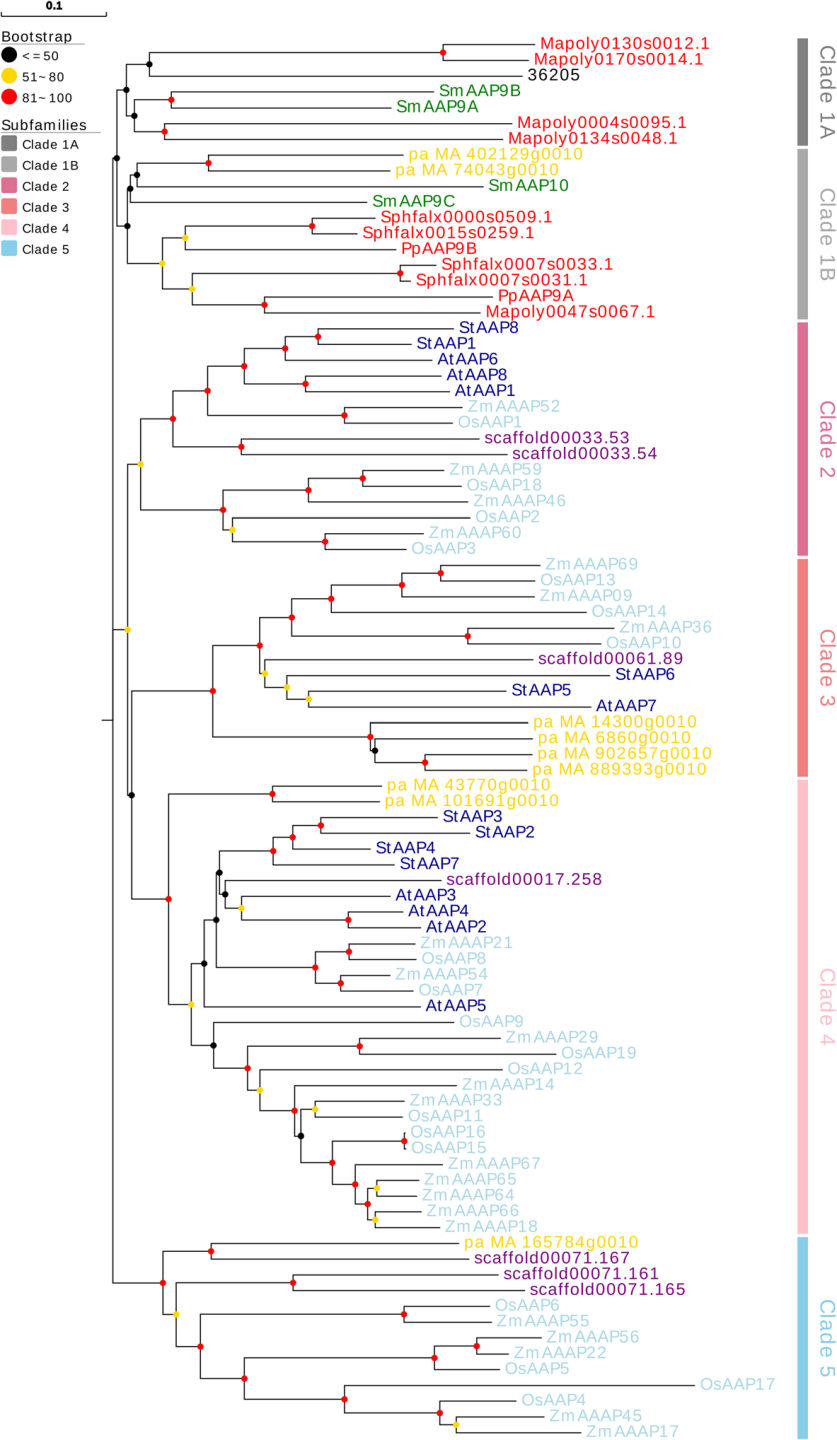
Phylogenetic tree of group I *AAP* members. Group I members are divided into 5 clades are indicated in different colors. The circles represent the bootstrap value. This value is an important for classifying the clades

In order to perform a comprehensive phylogenetic analysis of *AAP* proteins in plants, we selected some representative plant sequences at different evolutionary stages. In total, 154 proteins in 5 different plant stages, from chlorophytes to angiosperms, were used to construct a phylogenetic tree using the Neighbor-Joining method. We choose this method because it was especially well-suited for datasets comprising lineages with largely varying rates of evolution. It can be used in combination with methods that allow for correction of superimposed substitutions [28]. In the unroot tree we could easily divide to 2 main groups (Fig. 1). Group I had more branching events and group II could be clearly divided into 2 parts which could reference the bootstrap values. We selected group I proteins to construct a phylogenetic tree in which the bootstrap values separated group I into 5 clades (Fig. 3). Clade 1 contained non-seed plants and Gymnospermae, and separated into 2 clusters based on the bootstrap values. The other 4 clades comprised seed plants, and Gymnospermae were located in clade 3, 4 and 5. We referenced a part of the grouping method from Tegeder and Ward [13] to classify these proteins. In group I, *P. patens* and *S. moellendorffii AAP* proteins were identical to those identified in Tegeder and Ward [13]. Group II mainly included early plant species from Chlorophyta, Bryophyta, and lycophytes. *A. trichopoda* also appeared in this group as the sister group of the remaining flowering plants. Other early plant *AAP* proteins mainly appeared in clade 1 and amount of these proteins were belonged to clade 1B. But no proteins were appeared in clade 1 till the evolution of angiosperms (Table 1).

### Investigation of gene duplication events and annotations

**Fig. 4:**
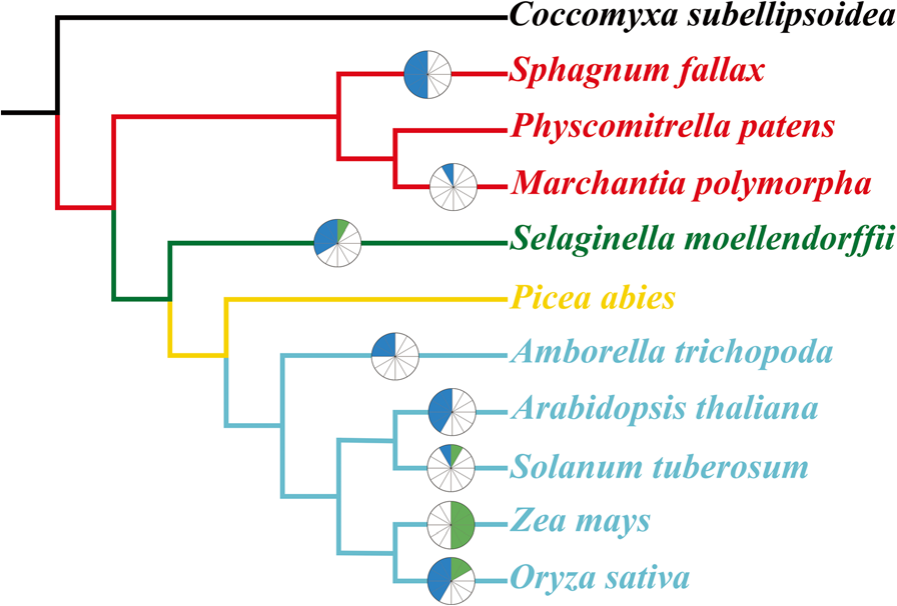
Hypothetical evolutionary models for *AAP*s from plants. The circles represent gene duplication events inferred from the phylogenetic analysis. The blue color indicates the number of tandem duplication and the green one means segmental duplication. The semicircle is divided into 6 parts, and each part is filled with color to represent a duplication event. Representative species of each major taxonomic group are shown at the branch tip. Branches are colored depending on their taxonomy classification

Gene duplication is potentially advantageous as a primary source of genes with new or modified functions [29]. We analyzed all predicted proteins from each species and found that *C. subellipsoidea, P. patens* and *P. abies* exhibited no duplication events. The highest number of tandem duplication events appeared in *S. fallax* and that of segment duplication events appeared in *Z. mays*. *Oryza sativa* had the highest number of duplication events (Additional file 1). Combined with the phylogenetic information it is evident that the duplication events of non-seed plants occurred in 2 main groups. Only *M. polymorpha* had a tandem duplication event that appeared in group II. All angiosperm duplication events belonged to group I except for those occurring in *A. trichopoda.* And *S. fallax* had a duplication event in group I, either (Fig. 4). The analysis of the plant genome duplication database (PGDD) [30] and MCscanX [31] also acquired 8 collinear gene pairs, which were homologous gene pairs in different plants. One of these was identified this event in *S. moellendorffii* for *SmAAP9C*, which had homologous genes in early plants, and the others all appeared in angiosperms (Additional file 3).

To better understand the gene evolution, it was necessary to calculate ratios of non-synonymous to synonymous nucleotide substitutions (*Ka/Ks*). We selected all duplicated Coding sequence (CDS) sequences, from which we had deleted the termination codon, to analyze the *Ka/Ks* ratios using DnaSP6 [32] and PGDD website databases. Firstly, the target genes were aligned using the ClustalX2 ‘align codons’ function. Following this, *Ka* and *Ks* values were analyzed in DnaSP6. In total, 48 gene pairs were analyzed, and *Ks* values could not be determined for 3 collinear gene pairs. *Ka/Ks* ratio values were slightly above 1.0 in only 2 gene pairs (*Sphfalx0007s0031.1/Sphfalx0007s0033.1* and *Sphfalx0362s0005.1/Sphfalx0362s0007.1*), and no *Ka/Ks* ratio values were much greater than 1.0. Collinear genes showed *Ka/Ks* ratios of less than 1.0 between *Z. mays* and *O. sativa*, whereas *Ks* values could not be determined between *A. trichopoda* and *O. sativa,* as well as *S. moellendorffii* and *A. thaliana* (Additional file 3).

We also used same method to calculate *Ka/Ks* ratio values in each of the plant species’ *AAP*s (Additional file 4). The highest *Ka/Ks* value was also *Sphfalx0007s031.1*/*Sphfalx0007s033.1* and in *OsAAP15*/*OsAAP16* and 174/1275 gene pairs the *Ka* value was 0 while the *Ks* value could not be calculated (Additional file 4). Overall, the *Ka/Ks* values of 16 gene pairs were greater than 1, with the majority occurring in monocots and 2 in *S. fallax*, which were duplication pairs (Additional file 6).

**Fig. 5:**
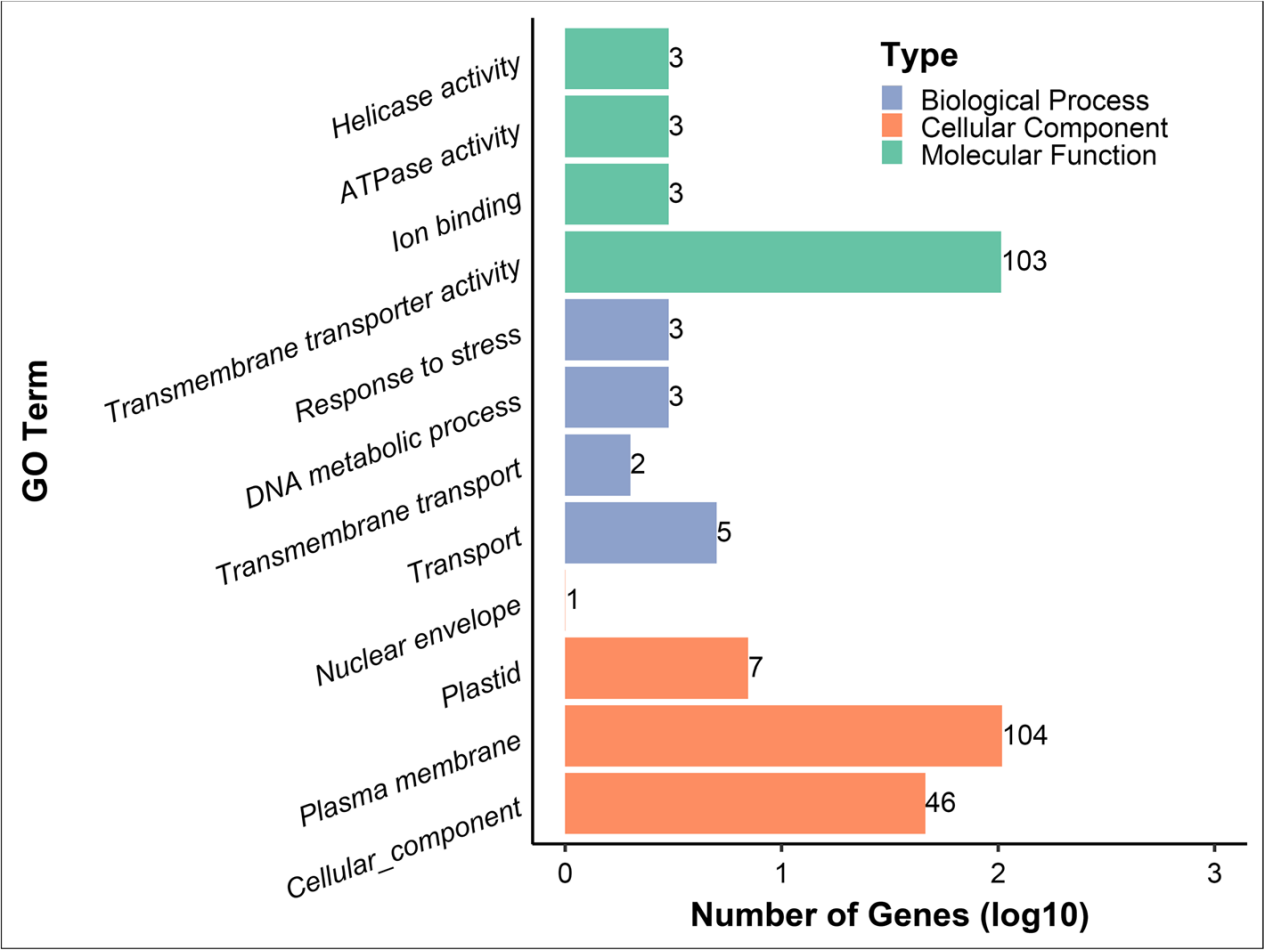
The annotation of gene ontology in whole *AAP*s. Colors indicate the type of gene annotation. The x-axis indicates the logarithm of protein numbers and the y-axis, the number of *AAP* members in each GO term

One hundred fifty-four proteins were annotated through Gene Ontology with specific reference to biological process (BP), molecular function (MF), and cellular component (CC). The results indicated that four aspects of CC were annotated to 154 genes and 46 proteins were predicted be related to CC, with majority of proteins belonging to non-seed plants. Seven proteins, which were all group II members, were located in plastids and only *AtAAP3* existed in the nuclear envelope. Most proteins were located in the plasma membrane. Four aspects of MF were annotated to 103 proteins that were linked to transmembrane transporter activity. Further, *OsAAP13*, *ZmAAAP09*, and *ZmAAAP69* were also associated with ion binding, ATPase activity and helicase activity. Four aspects of BP were annotated to 7 genes. Five proteins in Bryophyta participated in transport processes, two *S. moellendorffii AAP*s were related to transmembrane transport, and *OsAAP13*, *ZmAAAP09,* and *ZmAAAP69* were associated with DNA metabolic processes and stress response (Fig. 5, Additional file 5).

## Discussion

### Analysis of *AAP* proteins

*AAP* proteins belonged to the *AAAP* family and some proteins functioned were absorbing amino acid from roots and leaves and transported to other organs through the phloem. These findings based only on vascular plants and Tegeder and Ward’s research showed that this protein family was predicted in Bryophyta [13]. In the present study, we expanded the plant species investigated in predicting the function of *AAP* proteins. We blasted the target proteins in Chlorophytes and these results were not reported. We then selected some representative plants in various evolutionary stages to explain the evolution of *AAP* proteins.

The FPKM protein families with biased distribution in Coccomyxa from Blanc et al. [33] showed that 9 chlorophytes which they studied all contained *Aa_trans* domain. However, in the present study, *AAP*s just existed in *C. subellipsoidea* belonging to the class of Trebouxiophyceae. From this discovery, we inferred the *AAP*s might originate from Chlorophyta, but we could not find out some other evidences. On the other hand, the studied of Tegeder and Ward [13] showed AAP might only tract back to Bryophyta and Bowman et al. finally indicated that the GH3 protein from *M. polymorpha* which could belong to group I from Zhang’s research [6], but actually it proved that the protein was not related functions [7]. Thus, these hypothesis just depened on the protein prediction and structure analysis. Despite the fact that Chlorophyta are single-celled aquatic eukaryotes with no vascular structure, Blanc presented several protein families which were overrepresented in *C. subellipsoidae*, including those involved in lipid metabolism, transporters, cellulose synthases, and short alcohol dehydrogenases [33]. Work by Tegeder and Ward, as well as the present study, both identified *AAP* proteins in Bryophyta. As we used the database from the Phytozome V12 website, we were able to predict the function of more proteins than Tegeder and Ward (2012).

We predicted 154 *AAP* proteins and analyzed *Aa_trans* and transmembrane domain in each protein. Not only early plants but also other plant species had a phenomenon which was the location of transmembrane domains might locate in *Aa_trans* domain. This condition was more common in *A. trichopoda, S. fallax and C. subellipsoidea*. We also labeled these proteins as ‘Beyond’ in Additional file 10. Additionally, we used the MEME website to acquire the distribution of motifs in each protein. Non-vascular and vascular plants all contained these 10 motifs in the same position and order (Additional files 2, 11 and 12). This structural information validated the potential existence of these predicted proteins.

Exons and introns constituted a genetic sequence and exons which were part of transcript sequences played an important role in gene function. According to the number of exons contained in each plant’s *AAP*s it could be inferred that some introns may have been lost from Chlorophyta in subsequent evolutionary stages. Introns might be lost or gained over evolutionary time, as shown by many comparative studies of orthologous genes [34]. Due to the *AAP* genes in Chlorophyta all displaying the same transcript sequences, the structure of proteins did not vary greatly. Thus, we suggest that the differences in the number of introns/exons between different species is due to a large number of intron losses occurring during plant evolution. This phenomenon has been confirmed by Roy and Penny [35].

### Evolution of *AAP* proteins

The results of phylogenetic showed a majority of non-vascular plants (Chlorophyta and Bryophyta) and *A. trichopoda* were composed of group II. Interestingly, only *A. trichopoda* which belonged to Angiosperm and as a sister of flowering plant existed in group II which because six exogenous genomes constructed *A. trichopoda* mitochondrial genome, one from moss, three from green algae, and two from other flowering plants [36]. And we could not find out any AAP proteins belonged to group II in angiosperms. Group II could be divided into closely related 2 clusters. The phylogenetic tree also suggests that chlorophytes could be the origin of this protein. Due to the fact that the group of proteins all belonged to non-seed plants, it is likely that the function of this group is unrelated to amino acid transport in seeds. This suggests that the function of this protein group could disappear in evolution and the reason for this situation needed to be verified before the function of these genes could be further explained. On the other hand, the duplication events of these plant genes occurred mostly in this group which could mean some functionally redundant proteins were also predicted.

**Fig. 6:**
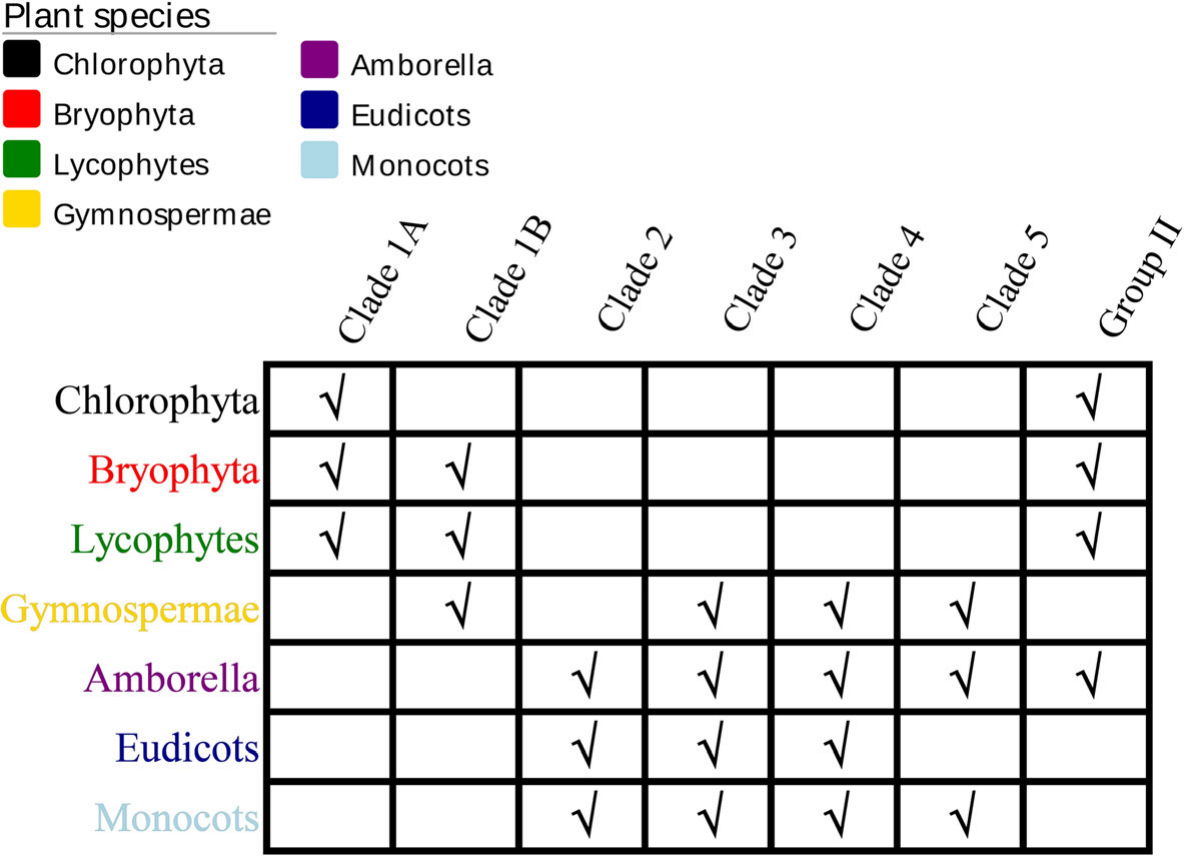
The presence of proteins in different groups and clades. The check mark within boxes indicate the group/clade have *AAP* members. This visually indicated the distribution of the group of proteins in each stage

The classified about group I might indicated the *AAP* proteins’ functional differentiation might occur in Gymnospermae and the distribution of *A. thaliana AAP* proteins in each clade also supports this supposition. This group might contain the primary proteins which are associated with amino acid transport. The phylogenetic tree of group I also indicated that clade 2 was closely related to clade 3 and 4 (Fig. 6). Compared to the phylogenetic trees of Tegeder and Ward, some of our branch proteins were grouped into different groups. These differences might be due to various factors, including the use of a different website to download the protein sequences, adding Gymnospermae and *A. trichopoda AAP* proteins into the analysis, and using a different website/program to analyze phylogenetic relationships. In our tree, we could infer that the functional *AAP* proteins originated from Chlorophyta.

The phylogenetic tree indicated that bryophytes and vascular plants might had a common ancestor that was inherited from *C. subellipsoidea AAP* protein in group I (Additional file 1, Fig. 3). All non-vascular plants and mosses were clustered together, and the familial division started from *P. abies*. In addition, we found one duplication event in both *S. fallax* and *S. moellendorffii*. The evolutionary history of gene duplication events in mosses and lycophytes were independent from those in seed plants. It was not until *A. trichopoda* that duplicated information appeared and was conserved in angiosperms. Two additional duplication events were inferred before or early on in the evolution of flowering plants, since they were already present in the genome of *A. trichopoda*, which is considered a basal flowering plant [24]. Angiosperms proteins were lost from our research in clade 1 (Fig. 6), and none Angiosperms were matches which we searched these proteins via NCBI blast. Conversely, this clade was not closely related to the other 4 clades and the specialization of *P. abies AAP*s might lead to divisions. Based on these phylogenetic inferences, we concluded that *AAP* group I genes have a complex evolutionary history with several specific duplication and loss events. The duplication of genes increased with plant evolution as the *AAP* genes went from one copy in Chlorophyta to dozens in eudicots. With the development of vascular plants, *AAP* members underwent a drastic increase (Fig. 4).

### Gene duplication events, *Ka/Ks* values, and GO annotation information

Gene duplication is a common phenomenon in all life forms and provides resources for novel gene functions [37]. The most obvious contribution of gene duplication to evolution is the provision of new genetic material for mutation, leading to specialized or new gene functions, and contributed to species divergence and origins of species-specific features [38]. Our analysis of the duplication events showed the *AAP* family gene duplications were present in bryophytes. Following the evolution of plants, duplication events appeared in each evolutionary stage except *P. abies* (belonging to Gymnospermae). We blasted some other gymnosperms and acquired no results through the NCBI database. It is possible that there were few sequences for gymnosperm species, and duplication events might be analyzed in future research. Analysis of duplication events in group I revealed that the evolution of *AAP*s was also based on gene replication. With the evolution of plants, duplication of *AAP*s gradually increased, providing evidence for the increasingly important role of this family in plant evolution. There was one duplication event in non-vascular plants and following the development of vascular plants, a drastic increase of duplication events appeared, which confirmed the important role of *AAP* as a transport-related protein.

Through calculating the *Ka* and *Ks* of duplication gene pairs in *S. fallax*, the *Ka/Ks* value of 3 gene pairs were found to be close to 1, meaning that these genes were not suffering natural selection pressure. The *Ka/Ks* values for the other duplicated genes were all consistent with purifying selection which were less than 1. And it was because a mutation that changes a protein is much less likely to be different between two species than one which is silent; that is, most of the time selection eliminates deleterious mutations, keeping the protein as it is [39]. In general, *AAP* duplications were not change protein within a species, as suggested by Arcadi and Barton [40]. The collinearity gene pairs also showed no one was from group II and the *Ka/Ks* value also indicated the evolution was stable (Additional file 3). Group I and group II had not significant evolutionary relationship.

The *Ka/Ks* ratio values of each species showed that most genes were stable and that they were all under purify selection or neutral evolution (Additional file 6). Even though some species exhibited distinct *Ka/Ks* values, the majority did not, which may have been affected by variable sequence alignment. In order to eliminate these distinctions, we separately compared the CDS sequences to calculate their *Ka/Ks* ratios. However, this produced very similar results to the original analysis. In general, the *AAP*s were a relatively stable gene family through the process of plant evolution.

Functional annotation of sequences is a key requirement for the successful generation of functional genomes in biological research. GO annotation is one of the ways to predict the function of genes in terms of cellular components, molecular function and biological processes [41]. In our study many plant species were not model organisms and therefore some GO information could not be acquire from website databases. Blast2GO software conveniently assisted with this problem. Based on the results, many proteins clustered in the plasma membrane and the *AAP* proteins main molecular function was in transmembrane transporter activity. These validated *AAP*s were integral membrane proteins involved in the transport of amino acids into the cell. Interestingly, *OsAAP13*, *ZmAAAP09*, and *ZmAAAP69* responded to stress, and only 2 proteins participated in transmembrane transport. The protein structure and phylogenetic tree confirm that these proteins belonged to the *AAP* family (Fig. 5).

## Conclusion

In recent years, the improvement of plant sequencing technology had provided some support for the study of basal lineages. Simultaneously, it also provides a lot of data for the evolutionary study of gene families. Here, we used these databases for the identification of *AAP*s in the plant kingdom. Firstly, we predicted and analyzed the structure of *AAP* members. Comparing with others rearch, we newly found *AAPs* were present in chlorophyte species and more *AAP* members were also predicted in Bryophyta and Lycophytes [13]. Phylogenetic relationships between members of the whole *AAP* family showed that these members were explicitly divided into two main groups in our research. This group classification contained a group enriched by a large number of non-seed plant family members. Group I members contained all plant stages. This group indicated the origin and evolution of a functional *AAP* gene. Group II enriched non-seed plants which might have special functions. The *AAP* genes in Chlorophyta were predicted in another group and this might advance the period of *AAP* protein from Bryophyta to Chlorophyta.

We found that each member had the same motifs and *Aa_trans* was the main sequence. The prediction of transmembrane structure showed that each member occurred in similar numbers and locations. The results indicated the structures of *AAP* members were relatively conservative in terms of plant evolution. Only the number of exons and introns varied and intron losses might drive this difference during plant evolution. The duplication events indicated that the increase in *AAP*s was based on the emergence of vascular bundles [42].

## Methods

### Analysis of *AAP* proteins in 17 plant species

The 17 plants protein/genome/CDS sequenceswere download from the Phytozome V12 website.^1^*Arabidopsis thaliana*, *O. sativa*, *Z. mays*, and *S. tuberosum* proteins were acquired from researchers [25–27]. We used *AtAAP*s protein sequences as a query to blast against other plants (*e*-value = 10^− 10^). To ensure each protein belonged to the *AAP* subfamily, all target proteins were analyzed by NCBI-CDD^2^ and Pfam^3^ to check that each protein had an amino acid transporter (*Aa_trans*) alignment. To ensure candidate proteins contained complete functional areas for *AAP*, all proteins were aligned using the multiple sequence alignment tool ClustalX2^.^^4^ After excluding small portions of proteins with a length considerably less than 341.30 aa, which is the average length of the *Aa_trans* domain,^5^ the remaining sequences were considered as putative proteins.

The proteins motifs were analyzed through the Pfam website^[5]^ and MEME website^6^ using the default parameters. A combined transmembrane topology and signal peptide was predicted by the TMHMM website.^7^

### Investigation of gene duplication events, *Ka/Ks* ratio values and annotation information

According to Zhang et al. (2018), gene tandem duplication pairs should satisfied two requirements. The first is the similarity of each pair gene sequence should be more than 50% and the second is the genes should be physically located in same chromosome with a distance of less than 50 kb from each other [6]. The PGDD website^8^ was used to search the gene segmental duplication pairs and the MCScanX program^9^ acquired other species databases which did not exist in the website. DnaSP6 software^10^ was used to calculate gene pair *Ka/Ks* ratios to describe the evolutionary pressure. Each CDS sequence was acquired from Phytozome V12 and the termination codons deleted before calculating *Ka/Ks* ratios.

Gene annotation was carried out by searching gene ontology (GO) through the Blast2GO software^.^^11^ After uploading the amino acid sequences to the software, the associated molecular function, cellular components, and biological processes are acquired. This is carried out separately for each species as the software cannot conduct simultaneous species analyzes. Blast2GO is based on the NCBI database, thus all genes can be analyzed at the same time.

### Phylogenetic analysis of *AAP*

The phylogenetic inference was carried out using the MEGA7 software^.^^12^ Seventeen species of plants were included in the tree. The Neighbor-Joining (NJ) method was used to calculate genetic distance [43]. To ensure the accuracy of the analysis, the number of bootstrap replications was set to 1000 with a Poisson substitution model and using the pairwise deletion option to handle missing data. The classification of family members is based on the multiple sequence alignment and the genetic distance in phylogenetic tree.

## Supplementary information

**Additional file 1. **A part of information about all *AAP*s. The table includes protein length, location of *Aa_trans*, number of tranmembrane domains and exons, gene name, duplication events and group of each gene.

**Additional file 2. **The data of motifs for all *AAP*s and specific information for 10 motifs.

**Additional file 3. **The information of duplication and collinearity gene pairs and their *Ka/Ks* rations.

**Additional file 4. ***Ka*, *Ks* and *Ka/Ks* ratios in *AAP* gene for each plant species.

**Additional file 5.** Enrichment and integration of GO annotation information refer to the analysis of Blast2GO.

**Additional file 6. **The enrichment of *Ka/Ks* ration values for each plant. Red dotted line is the genetic selection between gene pairs. The gene pairs that failed to get the *Ks* value are after the gray line.

**Additional file 7. **The protein/cDNA/gene sequences of *AAP* members.

**Additional file 8. **The information of GO annotation for each *AAP* members.

**Additional file 9.** Painting a GO annotation results using R code.

**Additional file 10. **The numbers and location of *Aa_trans* and transmembrane domain.

**Additional file 11. **The logo of *Aa_trans* domain amino acid sequence.

**Additional file 12.** Composition of AAP protein motifs by MEME website.

**Additional file 13..** The result of NCBI-CDD which include the mainly domain in each protein.

## Data Availability

All data generated or analyzed during this study are included in this published article.

## Abbreviations

*AAP*s: Amino acid permeases
*APC*: Amino acid-polyamine-choline
*AAAP*: Amino acid/auxin permease
*LHT*s: Lysine-histidine-like transporters
*ProT*s: Proline transporters
*GAT*s: γ-aminobutyric acid transporters
*AUX*s: Auxin transporters
*ANT*s: Aromatic and neutral amino acid transporters
BP: Biological process
MF: Molecular function
CC: Cellular component
GO: Gene Ontology

## Footnotes

1 https://phytozome.jgi.doe.gov/pz/portal.html

2 https://www.ncbi.nlm.nih.gov/Structure/bwrpsb/bwrpsb.cgi

3 http://pfam.xfam.org/search

4 http://www.clustal.org/clustal2/

5 http://pfam.xfam.org/family/PF01490#tabview=tab6

6 http://meme-suite.org/tools/meme

7 http://www.cbs.dtu.dk/services/TMHMM/

8 http://chibba.agtec.uga.edu/duplication/

9 http://chibba.pgml.uga.edu/mcscan2/

10 http://www.ub.edu/dnasp/

11 https://www.blast2go.com/

12 https://www.megasoftware.net/

## References

[1] Delwiche CF, Cooper ED (2015). The evolutionary origin of a terrestrial Flora. Current Biology Cb.

[2] Harholt J, Moestrup Ø, Ulvskov P (2016). Why plants were terrestrial from the beginning. Trends Plant Sci.

[3] Catarino B, Hetherington AJ, Emms DM, Kelly S, Dolan L (2016). The stepwise increase in the number of transcription factor families in the Precambrian predated the diversification of plants on land. Mol Biol Evol.

[4] Moghe G, Last RL (2015). Something old, something new: conserved enzymes and the evolution of novelty in plant specialized metabolism. Plant Physiol.

[5] Rensing SA (2014). Gene duplication as a driver of plant morphogenetic evolution. Curr Opin Plant Biol.

[6] Zhang C, Zhang L, Wang D, Ma H, Liu B, Shi Z, Ma X, Chen Y, Chen Q (2018). Evolutionary history of the glycoside hydrolase 3 (GH3) family based on the sequenced genomes of 48 plants and identification of Jasmonic acid-related GH3 proteins in Solanum tuberosum. Int J Mol Sci.

[7] Bowman JL, Kohchi T, Yamato KT, Jenkins J, Shu S, Ishizaki K, Yamaoka S, Nishihama R, Nakamura Y, Berger F: Insights into Land Plant Evolution Garnered from the Marchantia polymorpha Genome. Cell 2017, 171(2):págs. 287–304. 10.1016/j.cell.2017.09.030 28985561

[8] Zhang L, Tan Q, Lee R, Trethewy A, Lee YH, Tegeder M (2010). Altered xylem-phloem transfer of amino acids affects metabolism and leads to increased seed yield and oil content in Arabidopsis. Plant Cell.

[9] Rentsch D, Schmidt S, Tegeder M (2007). Transporters for uptake and allocation of organic nitrogen compounds in plants. FEBS Lett.

[10] Boudko DY. Molecular ontology of amino acid. Transport. 2010:379–472.

[11] Chang AB, Lin R, Studley WK, Tran CV, Saier MH (2004). Phylogeny as a guide to structure and function of membrane transport proteins (review). Membr Biochem.

[12] Cheng L, Yuan HY, Ren R, Zhao SQ, Han YP, Zhou QY, Ke DX, Wang YX, Wang L (2016). Genome-wide identification, classification, and expression analysis of amino acid transporter gene family in glycine max. Front Plant Sci.

[13] Tegeder M, Ward JM (2012). Molecular evolution of plant AAP and LHT amino acid transporters. Front Plant Sci.

[14] Lu Y, Song Z, Kai L, Lian X, Cai H (2012). Molecular characterization, expression and functional analysis of the amino acid transporter gene family (OsAATs) in rice. Acta Physiol Plant.

[15] Emery L, Whelan S, Hirschi KD, Pittman JK (2012). Protein phylogenetic analysis of Ca2+/cation Antiporters and insights into their evolution in plants. Front Plant Sci.

[16] Marchler-Bauer A, Bo Y, Han L, He J, Lanczycki CJ, Lu S, Chitsaz F, Derbyshire MK, Geer RC, Gonzales NR (2017). CDD/SPARCLE: functional classification of proteins via subfamily domain architectures. Nucleic Acids Res.

[17] Sanders A, Collier R, Trethewy A, Gould G, Sieker R, Tegeder M (2010). AAP1 regulates import of amino acids into developing Arabidopsis embryos. Plant J.

[18] Fischer W, Kwart M, Hummel S, Frommer WB (1995). Substrate specificity and expression profile of amino acid transporters (AAPs) in Arabidopsis. J Biol Chem.

[19] Rentsch D, Hirner B, Schmelzer E, Frommer WB (1996). Salt stress-induced proline transporters and salt stress-repressed broad specificity amino acid permeases identified by suppression of a yeast amino acid permease-targeting mutant. Plant Cell.

[20] Tegeder M, Rentsch D (2010). Uptake and partitioning of amino acids and peptides. Mol Plant.

[21] Peng B, Kong H, Li Y, Wang L, Zhong M, Sun L, Gao G, Zhang Q, Luo L, Wang G (2014). OsAAP6 functions as an important regulator of grain protein content and nutritional quality in rice. Nat Commun.

[22] Koch W, Kwart M, Laubner M, Heineke D, Stransky H, Frommer WB, Tegeder M (2010). Reduced amino acid content in transgenic potato tubers due to antisense inhibition of the leaf H+/amino acid symporter StAAP1. Plant J.

[23] Rolletschek H, Heim U, Borisjuk L, Saalbach I, Wobus U, Weber H (2005). Ectopic expression of an amino acid transporter (VfAAP1) in seeds of Vicia narbonensis and pea increases storage proteins. Plant Physiol.

[24] Romani F, Reinheimer R, Florent SN, Bowman JL, Moreno JE (2018). Evolutionary history of HOMEODOMAIN LEUCINE ZIPPER transcription factors during plant transition to land. New Phytol.

[25] Ma H, Cao X, Shi S, Li S, Gao J, Ma Y, Zhao Q, Chen Q (2016). Genome-wide survey and expression analysis of the amino acid transporter superfamily in potato (Solanum tuberosum L.). Plant Physiol Biochem.

[26] Lei Sheng LD, Yan H, Zhao Y, Dong Q, Li Q, Li X, Cheng B, Haiyang J. A Genome-Wide Analysis of the AAAP Gene Family in Maize. J Proteomic Bioinform. 2014;07(1):22–33.

[27] Zhao H, Ma H, Li Y, Xin W, Jie Z (2012). Genome-Wide Survey and Expression Analysis of Amino Acid Transporter Gene Family in Rice (Oryza sativa L). Plos One.

[28] Zhang W, Sun Z (2008). Random local neighbor joining: a new method for reconstructing phylogenetic trees. Mol Phylogenet Evol.

[29] Lee TH, Kim J, Robertson JS, Paterson AH (2017). Plant genome duplication database. Methods Mol Biol.

[30] Tae-Ho L, Haibao T, Xiyin W, Paterson AH (2013). PGDD: a database of gene and genome duplication in plants. Nucleic Acids Res.

[31] Yupeng W, Haibao T, Debarry JD, Xu T, Jingping L, Xiyin W, Tae-Ho L, Huizhe J, Barry M, Hui G (2012). MCScanX: a toolkit for detection and evolutionary analysis of gene synteny and collinearity. Nucleic Acids Res.

[32] Rozas J, Ferrer-Mata A, SÃ nchez-DelBarrio JC, Guirao-Rico S, Librado P, Ramos-Onsins SE, Sã n-GA: DnaSP 6: DNA Sequence Polymorphism Analysis of Large Datasets. Mol Biol Evol 2017;34(12). 10.1093/molbev/msx248 29029172

[33] Blanc G, Agarkova I, Grimwood J, Kuo A, Brueggeman A, Dunigan DD, Gurnon J, Ladunga I, Lindquist E, Lucas S. The genome of the polar eukaryotic microalga Coccomyxa subellipsoidea reveals traits of cold adaptation. Genome Biol. 2012;13(5):1–12. 10.1186/gb-2012-13-5-r39 PMC3446292 22630137

[34] Rogozin IB (2012). Origin and evolution of spliceosomal introns. Biol Direct.

[35] Roy SW, Penny D (2007). Patterns of intron loss and gain in plants: intron loss-dominated evolution and genome-wide comparison of O. sativa and A. thaliana. Mol Biol Evol.

[36] Rice DW, Alverson AJ, Richardson AO, Young GJ, Sanchezpuerta MV, Munzinger J, Barry K, Boore JL, Zhang Y, Depamphilis CW (2013). Horizontal transfer of entire genomes via mitochondrial fusion in the angiosperm Amborella. Science.

[37] Zheng W, Zhengkui Z, Yunfeng L, Tengfei L, Qing L, Yuanyuan J, Congcong L, Chao F, Min W, Mian W (2015). Functional evolution of phosphatidylethanolamine binding proteins in soybean and Arabidopsis. Plant Cell.

[38] Zhang J (2003). Evolution by gene duplication: an update. Trends Ecol Evol.

[39] Hurst LD (2002). The K a/ K s ratio: diagnosing the form of sequence evolution. Trends Genet.

[40] Arcadi N, Barton NH (2003). Chromosomal speciation and molecular divergence--accelerated evolution in rearranged chromosomes. Science.

[41] Conesa A, Götz S (2008). Blast2GO: a comprehensive suite for functional analysis in plant genomics. Int J Plant Genom.

[42] Taylor MR, Reinders A, Ward JM (2015). Transport function of Rice amino acid Permeases (AAPs). Plant Cell Physiol.

[43] Singh VK, Jain M, Garg R (2014). Genome-wide analysis and expression profiling suggest diverse roles of GH3 genes during development and abiotic stress responses in legumes. Front Plant Sci.

