# Supplementary figures and images for: Evolutionary significance of amino acid permease transporters in 17 plants from Chlorophyta to Angiospermae
Source: BMC Genomics. 2020 Jun 5;21:391. doi: 10.1186/s12864-020-6729-3 (PMC7275304; doi:10.1186/s12864-020-6729-3)

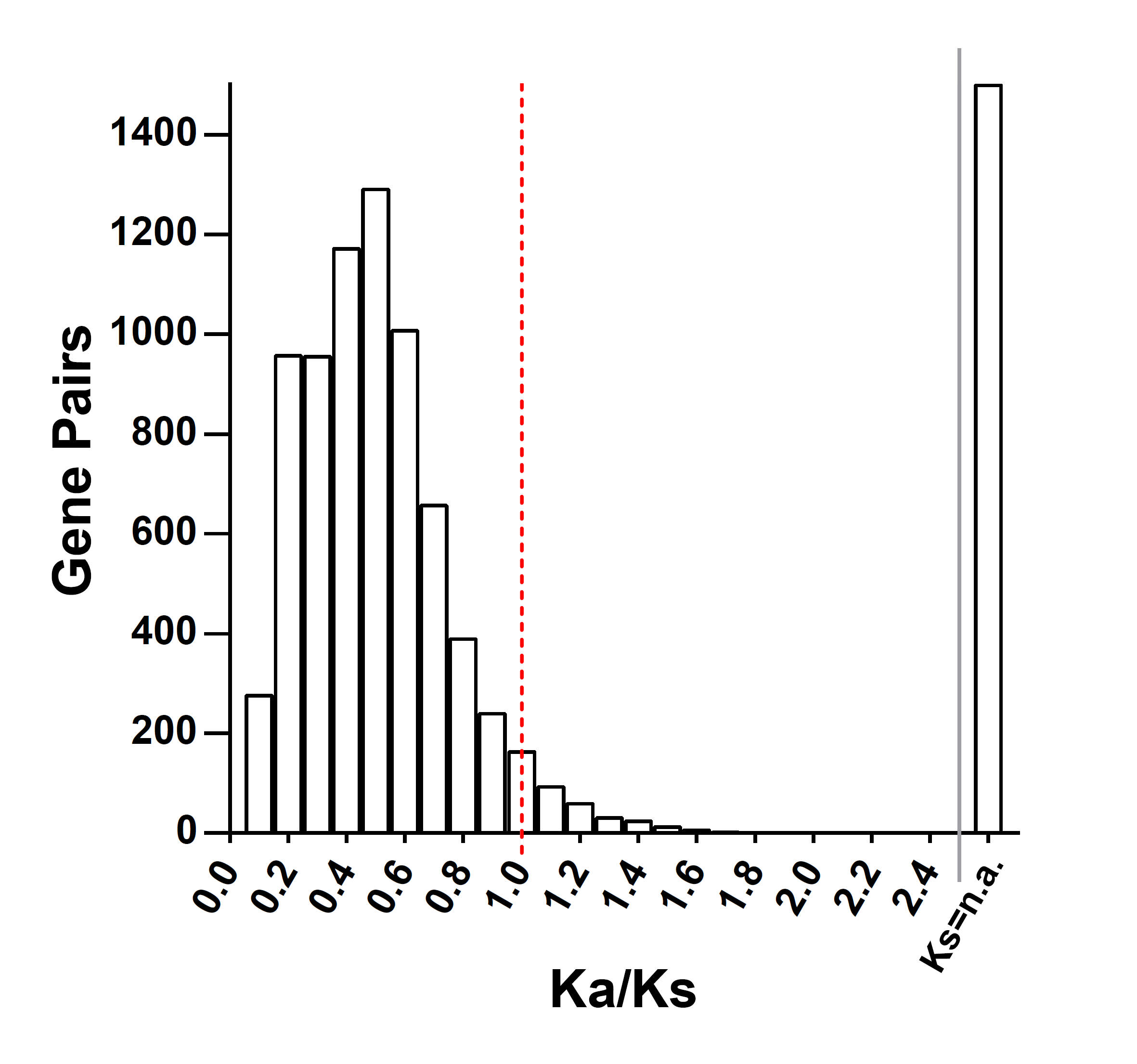

Supplement: Supplementary file 6 — Additional file 6. The enrichment of Ka/Ks ration values for each plant. Red dotted line is the genetic selection between gene pairs. The gene pairs that failed to get the Ks value are after the gray line. [file 12864_2020_6729_MOESM6_ESM.tif]

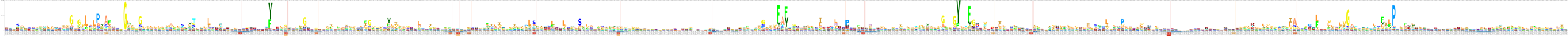

Supplement: Supplementary file 11 — Additional file 11. The logo of Aa_trans domain amino acid sequence. [file 12864_2020_6729_MOESM11_ESM.png]
