# Supplementary material for: Evolutionary significance of amino acid permease transporters in 17 plants from Chlorophyta to Angiospermae
Source: BMC Genomics. 2020 Jun 5;21:391. doi: 10.1186/s12864-020-6729-3 (PMC7275304; doi:10.1186/s12864-020-6729-3)
Supplement: Supplementary file 7 — Additional file 7. The protein/cDNA/gene sequences of AAP members. [file 12864_2020_6729_MOESM7_ESM.docx]

Protein sequences

>36205

MENGEPHNPKLLVDEKGFARSDLEKYDDDGHVARTGGWITAYAHIVCAVIGSGVLSLAWGVSWLGWVAGPIVLFMFAWIT

WYCSALLIDCYRFPDVDGEKRNYTYIQAVKRYLDANMVGTSVGYTVTAGIAATAIRRSDCFHADISNPCEISNNPWIILF

GALQILFSQIQDIDRIWWLSIVATLMSFTYAFIGLGECIAQAAQGSTTGTGTVGGLQIGIDTTAAGKVWGIFQALGNIAF

AYSFSFILIEITDTIQSPGETKKMRRATVYGIATTTFFYACIGIIGYAAFGNSAPGNLLSGFGFYNPWWLIDIANAAIFV

HLLGGYQVWIQPFFGFVEASAFRYFPKSRFLQWELFAVEIPGMGLFRASPFRLIWRTVYVIIVTIVALLLPFFNDIVGLL

GAIGFAPLTVFFPIQMHIVQKKIPMWSGRWCFLQGLNVLCWLISIAAAIGSVEGIYADTRNYTPFQTSYRR*

>31400

MADVEGKAAPDTGHENGNAKEPLGHLNKYDQEYKLPITGDRTGKWWYSAFHNVTAMVGAGVLGLPSAMAYLGWGGGMFIM

VSSWIITLYTLWQLCSMHEMNGKRFNRYHELGQYAFGQKRGLWFVIPFQLIVMIGLAIVYCVTGGKSMQAVWQFLCNKPC

PAFGLSAWIVVFAGAQLFLSQCPNFNSLRVVSFAAAIMSLAYSTIAVGASIASGRQPDAYYNLDTKDTADKVFGVFSALG

TVAFAYGGHNVVLEIQATLPSPPDTFKPMMAGVYVAYALVAWCYFAVSITGYWAFGINVADNVLLTSALKDTVPNGLIIA

ADLFVVIHVIGSFQVYSMPVFDMIETRMVMSGISNALPMRLLYRSVYVIIVAFVAIVLPFFGDLLGFIGAFAFGPTTFWM

PPIIYLIVKKPKINSGHWWASWFCIIYGLIVTIFGSIGGMRGIIKSASTYKFFQ*

>55902

MTAASSTSDVTRLVDQPLSFELERQNGHASTSGSTAPQSKWYDATFHTITAVVGVGVLSLPYAFSYLTWTGGVIALAVTT

ATSLYTGYLLAALHEDKNGHRHNRYRDLGRAIFGEKWGNWAIAPFQWSVLVGLAITYTATAGQSLQAVHSSTCNNAVYKA

VGAGRTDRNCSSALAWWTIVFSFFELFLSQIKDFHSLWWVSLLGAAMSAMYSTLAFATSVAAGSEGASYGPRQESPAALI

LGAFNALGTIMFAFGGHAILLEVQATMQTPPSALKSMMRGLGAAYTVVVIAYFPVASAGYAAFGNVVSPDVLLSVRKPAW

LISIANFMVVIHLAASYQVFAQPIFETAEGWLAARKHRLVDRPIVTRAIVRCSYVALTCFAAILIPFFGDLMGLVGSLGL

MPLTFILPPALWIKATKPKGPELWFNVALMVVYGVAGVLAAIGSVYNIVVHAHEYHTVG*

>24967

MALGEKTGMEDSAHANKVNFSKDPEGQMELDDKQTVPEYVGKGEWYHIGYHMTAAVASVPTLGLPFAVSLLGWGGGLVAL

IAGGLVTMFTSFLVSSMLEYGGKRHIRFRDLSVAVFGKSGWWAVTPFQFAVCIGTTIANHIVGGQAIKAIDVLARGETPV

TLTQYILVFGAVNLILAQCPNFHSIRFVNQTATVCTISFSIIAVALSLYSGFTMDLQPDYTVPGEGVNKLFNIFNGLGIM

AFAYGNTVIPEIGATAKAPAMRTMKGGIIMGYCTIVSAYLCVSITGYWAFGNGVKGLVLGSLTNPGWAVIMAWAFAAVQL

FGTTQVYCQPIYEACDKTFGNILAPTWNLKNTIVRLICRTVFICLCILVGAMLPFFVDFMSLIGAIGFTPMDFVLPQFLW

IKAYKPKGFSKWFSLLVAIIYIIVGIMACIGAVRSIVLNAVNYSLFANL*

>29311

MSKLNDSAHGNDLERKPSKVEEGVIGKHGSTSPLIEITHNDKWYHAGGHICTIIATPAAYAPLPFAFAHLGWEAGVIFLL

LAGLVTWYTSLLLASLDRHDGKRHTRYCDLAGSIYGKGGYWSVIFFQQLASIGNNLTIQIVAGQCLKALYRLYHPECEPT

GACGISLQAWIAVFGASQLILSQLPDISSLREINLVCTLCTVCFAVGCLAMSIYNGNTQVDRSTVSYDVQGDAKPKIFNI

MFSLGIIAFAFGDTILPEVQATVGGDSKKVMYKGVSCGYAILLSSYMVVAIAGYWAFGFDVSPFVVFSFKEPSGMLAALY

IFAVLQIIGCYQIYARPTFGFAYNYMLRPYEGVWSFHNVLMRAIVTTIYMAIITLIAAMIPFFGDFVAFVGAIGFTPMDF

ILPIILWQKVGKHSLIVSIVNWCIVVFYSIIAIAGAIGSIQAINADLANFNVFADLF*

>Sphfalx0000s0509.1

MKFEGAAGGDENLGRAENGNAAAADQFNKRNHALNHIPTSKQLDDDGKTRRRGTVWTASAHVITAVIGSGVLSLAWSVAQ

MGWVVGPTVLLLFALVTYYTALLLTDCYRYPDPVSGKRNYTYMDAVQANLGPRQVFICGLVQYSNLLGTAIGYTITATIS

MVAIKRSDCFHADGDSAPCRESNFIYMVFFGIVQVILSQIPNFDRLWWLSIVAAIMSFSYSTIGLGLGLGKASEGDHSHG

TLTGVEVGDHSLGFATRAQKIWDVCNALGNIAFAYSFSMILIEIQDTLKAPPAENKTMKRATLIGILTTTIFYMSVGCVG

YAAFGDAAPGNLLTGFGFYNPYWLVDFANACIVVHLVGAYQVYTQPLFAFVEDSMSRKFPKSNFIHKELEMNLPLGGPLH

INLFRLVWRTSFVTFTTLVSLMVPFFNDVLGLIGACAFWPLTVYFPVQMYIMQQSIQRWSSTWLALQTLNTVCFFVSLAA

AVGSIAGILTDVKHYTPFKS*

>Sphfalx0007s0033.1

MGWKEDGRIAQAEDRAANGDSNGSAVYDPTKFDEDGKPRRKGNVMTTSAHVITAVIGSGVLSLAWSIAQMGWIAGPSVLL

LFGAITYYTSCLLADCYRYPDPVTGKRNYTYMETVQANLGPKQVWACGLVQYSNLLGTSIGYVITGAQSARAITKSNCFH

SNPDSPCLRSNNGYMISFGIVQLVLSQIPNFGELWWLSYLAAAMSLIYSTTGLGLSIGKIAEGGYSHGSISGTSIGDPSL

AGYNTRAQKTWNVFNALGDMAFAYSFSMILIEIQDTLRAPPAENKSMKRATLIGILTSTGFYMSVGCAGYAAFGNASQGN

LLTGFGFYNPYWLVDFANACVVVHLIGAYQVYTQPLYAFIEEWVSSKFPKSNFINKEHYVKLPFGEPLPINHFRLVWRSI

YVVMTTIVSMLLPFFNDILGLIGACGFWPLTVYFPVEMYIHQTRLPRWSQKWILLQSLSVVTFVISLAAAIGSVASIVSD

VQGYKPFSNDA*

>Sphfalx0007s0031.1

MGWEEDGKIAQAEDGAANGDSNGSAVYDPTKFDEDGKPRRKGNVMTASAHVITAVIGSGVLSLAWSIAQMGWIAGPSVLL

LFAAVTYYTSCLLADCYRYPDPVTGKRNYTYMETVQANLGPKQVWACGLVQYSNLLGTSIGYTITGAQSARAITKSNCFH

SNPDSPCLRSNNGYMISFGIVQLVLSQIPNFGELWWLSYVAAGMSFIYSTIGLGLGIGKIAEGGYSHGSISGTSIGDPSL

AGYNTRAQKTWNVFNALGDMAFAYSFSLILIEIQDTLKAPPAENKSMKRAALIAILTTTGFYMSVGCAGYAAFGNASQGN

LLTGFGFYNPYWLVDFANACVVVHLIGAYQVYTQPLYAFIEEWVSSKFPKSNFINKEHYVKLPFGEPLPINHFRLVWRSI

YVVMTTIVSMLLPFFNDILGLIGAFAFWPLTVYFPVEMYIHQKRLPRWSQKWILLQSLSVVTFVVSLAAAIGSVASIVSD

VQGYKPFSNDA*

>Sphfalx0015s0259.1

MGWVVGPIVLLLFALVTYYTALLLTDCYRSPDPVSGKRNYTYMDAVKANLGPKQVVICGLVQYTNLLGTAIGYTITATTS

MVAIKRSDCFHADGDSAPCHVSNIIYMVFFGIVQVILSQIPDFDRIWWLSIVAAIMSFSYSTIGLGLGLGKASEGDHPHG

TLTGVEIGDPSIGFATKAQKIWDVCNALGNIAFAYSFSMILIEIQDTLKAPPAENKTMKRATLIGILTTTIFYMSVGCVG

YAAFGDGAPGNLLTGFGFYNPYWLVDFANACIVVHLVGAYQVYTQPLFAFVEHTMSRKFPKSDFIHKDLEMKLPWGAPLH

INLFRLVWRTAFVAFTTVVSLVIPFFNDVLGLIGAFSFWPLTVYFPIQMYTVQQSIHKWSSTWLALHTLSVVCFFVSLAA

AVGSIAGILTDLKHYTPFKS*

>Sphfalx0013s0130.1

MATAKDVENVDNGHAPDSSAAEQKALEDWLPVTGNRKAKWWYAAFHNVTAMVGAGVLGLPTAMQWLTWGPGVVVLILSWV

ITLYTLWQMVEMHEIVPGKRFDRYHELGQEAFGEKLGLWIVVPQQLIVQVGVDIVYMVTGGRSLQTIYKLLCKGPCTLEL

HISLWIFIFGSVHFFLSQLPNFNSISGLSLAAAIMSLSYSTIAWAIPARYGHRLPGTNSVVSADYHLPYGHKIGGDVMNI

FNALGTVAFAYAGHNVVLEIQATIPSTPEKPSKSAMWRGVLVAYMVVAACYFPVSLVGYWAYGNNATLLVDGNILTFEAF

PVWLVVVANFMVIVHVMGSYQIYAMPVFDTLETLLVRRLHFPPSFHLRLVTRSLYVIFTMFVGITFPFFGALLGFFGGFA

FAPTTYFLPCIMWLCIVKPKAFSLSWIINWVCIVLGVLLMFAATIGGMANIIVSASTYKFYE*

>Sphfalx0007s0128.1

MAYIPGPQNFSFHEENEGSEDIETPKTIKTQQPKEQRNVEEWLPVTSSRNAKWWYSAFHNVTAMVGAGVLSLPSAMAYLT

WGPGVAVLLSSWVITLFTLWQLVQMHEMKEFPGKRFDRYHELGQHAFGKKLGLWLVVPQQLIVEIGVDIVYMVTGGPSSC

RPIRKTAWIAIFGSVHFFLAQCPNFNAISLVSFCAAIMSLSYSAIAWVAPLASGQVADVSYALPDTSRAGLVFGILNALG

QIAFAYAGHNVVLEIQATLPSTPEKPSKGPMWRGCLVAYVVVAACYFPVAMVGYWAMGNGVGDNVLLSLGKPVWLIAAAR

LMVVVHVIGSYQVYAMPVFDMMETFLVKKLEWNPTRFLRLWVRSLYVAFTIFMAMTIPFFGDLLGFLGGFAFAPTTFFLP

CCMWLTIYKPKAFSMSWILNWVPI*

>Sphfalx0084s0063.1

MATTKDVDDVDNGFAPGPGGKTVDDWLPVTGSRNAKWWYAAFHNVTAMVGAGVLGLPTAMQFLTWGPGVVVLVLSWVITL

YTLWQMVEMHEMVPGKRFDRYHELGQEAFGPKLGLWIVVPQQLIVQVGVDIVYMVTGGKSLQKFYHLVCKGNCDLYKHTS

LWIVIFGSVHFFLSQLPNFNSISGISLAAAIMSLSYSTIAWAIPAHYGHTIPGTTQYVHPDYHLPYHHNVGGYVFEVFNA

LGTVAFAYAGHNVVLEIQATIPSTPERPSKIPMWRGVILAYIVVAACYFPVSLVGYWAYGNNNGLVADGNILAFEGFPVW

LVATANLMVVVHVIGSYQIYAMPVFDQLETVMVKKLHFTPSVLLRLVTRSIYVAVTIFVGVTFPFFGALLGFFGGFAFAP

TTYFLPCIMWLCIYKPKPFSLSWITNWICIILGVLLMFTASIGGLQQIIVDAKTYKFYT*

>Sphfalx0616s0001.1

MFSKVSKSPLASATTDEDFQAYVEEIEPAKPVNVEDWLPVTGSRNAKWWYAAFHNVTAMVGAAVLSLPYAMVYLTWGPGV

IVLVLSWVITLYTLWQMVEMHEMVPGKRFDRYYELGQEAFGEKLGLWIVVPQQLIVGVGVDIVYMVTGGKSLKKFYELTC

KKNCYLQNRLSIWILVFGSVHLFLAQLPNFNSIAGISLAAAIMSLSYSTIAWAIPAHYGHSLLPPGQSPDYHLPPNQSTA

ALVFGAFNALGTVAFAYAGHNIVLEIQATIPSTPHRPSKIAMWRGVLVAYGIVAACYFPVAIVGYWAFGNQVQDNIITFV

AKPVWLVAIANLMVVIHVIGSYQIYAMPVFDMLETVLVKRFHFSPSLQLRLITRSIYVGLTMFVGMTLPFFSSLLGFFGG

FAFAPTTYFLPCCMWLTIKKPRIGSLSWLANWACIILGVMLMLVSSIGGLRQIIVDASSFKFYE*

>Sphfalx0002s0399.1

MFSKDSKGPLASATTDEDFQAYVEEIEPAKPVNVEDWLPVTGSRNAKWWYAAFHNVTAMVGAGVLGLPYAMVYLTWGPGV

IVLVLSWVITLYTLWQMVEMHEMVEGKRFDRYHELGQEAFGEKLGLWIVVPQQVIVEVGVDIVYMVTGGKSLEKFYELTC

KKNCYLQNRLSIWILVFGSVHLFLAQLPNFNSIAGISLAAAIMSLSYSTIAWAIPAHYGHSLLPPGQRPDYHLPPNQSTA

AMVFGAFNALGTVAFAYAGHNVVLEIQATIPSTSHRPSRIAMWRGVLVAYGIVAACYFPVAFVGYWAFGNQVQDNIITFV

AKPVWLVAIANLMVVIHVIGSYQIYAMPVFDMLETLLVKRFRFSPSLRLRLITRSIYVGLTMFVGITLPFFGALLGFFGG

FAFAPTTYFLPCCMWLTIKKPRIGSLSWLANWVCIILGVMLMLVSSIGGLRQIIVDASSYKFYQ*

>Sphfalx0040s0186.1

MPEYFSDPHAVHVVALAPLQDQVADLENFTTTAASDDFQQERRDVENWLPVTSSRNARWWYSAFHNVTAMVGAGVLSLPS

AMAYLTWGPGMALLALSWVVTLFTLWQLVEMHELEAEPGRRFNRYHELGQYVFGEKLGLWLVVPQQLIAMVGVDIVYVLT

GGSSLMGAYDLLCTDPVGCRHIHKTAWIAIFGSVHFFLAQCPNFNSISIVSFSAAIMSVSYSMAAWIAPLCAGRIASVSY

AVPAHESKSTAGIAYFGILNAVGQVAFAFAGHNVVLEIQASLPSTPETPSKVPMWRGCLLAYAVVALCYFPVAIVGYWAM

GNGEGDNVLLSLGKPVWLLAVANLMVVIHVLGSYQIFAMPVFDMMESILVSKLEWNHTRSLRLWVRSLYVAFTIFVAITI

PFFGDLVGFLGGIAFAPTTFLLPCSMWLKIFKPKAFSFSWILNWFCMVLGLLIWLTIPVGGLHQIIIISFSKYKFYG*

>Sphfalx0084s0062.1

MEDRVLQVEDRDYNSKLNDLLPVTASREGKWWYSAVHNVTAIVSAGVLGLPSAMADLTWGPGVVVLTLSWVITFFTLWQM

VEMHEMVPGKRFDRYHELGQQAFGKKLGLWIVIPQQLLVEVSVDIVYMVTGGQALRYIYILNCKGDHHHCPLKPSSTEIN

NNPYAMTSHWILIYGSVHFLFVHIPNFNSMAGISLAAAIMSVSYSTIAWTIPIFHNEHSQHRDIANYTLVNVTADYDLPK

ASTAGHVLSAFNALGVVAFAYAGHNVVLEIQATLRSRPGKLSKIAMWRGVFLAYVIVTICYFPVALVCYWAYGNQLAADS

DSNSNSTFNSNILQFEGFRGITTAANFMVIIHILGSYQIYAMPVFDMLETVLMKKWLLAPSFKLRLITRSTYVGITMVVA

TIIPFFQPLLGFFGGFAYAPTTYFLPCCIWLAVYQPKRFSASWTINWICIILGVLLMLTATIGGGWEIVKEWNTYKFRTF

W*

>Sphfalx0104s0057.1

METQIQANGVHGAHHEDEKPLRLAAFEMEIPIGTRRNGNWWSAAFHNVTAVVSAGVLGLPNAMVFLTWGPGIVVLILSWI

VTLFSFWQLIQLHERPPNKRFNRYHELGQEAFGKTRGFWIVVPLQLLVELSVDILYMVVGGQALQNIYNMNCSGDCPLSN

LGPNPYRRTYIWILVFASVYFLLVQLPTLSSLSKLSLAAAIMSIGYSTIAWIIPVALNHQHNDMTNGNGLVSVTPRYHLP

YYDGTTRGGKFHHTDTEAYVLSIFNALSTVAFAYAGHNVSLEIQATLPSTPEEPSRIAMWRGVKLAYAIIALCYFPVALI

CYWAYGNDLKKSNLGILRYESWPVWLATAANLMVVIHVTGSYLIYAMPLFDMMELVLVGKWRLPPSFKLRLITRSLYVGF

TMVMAMTFPFFKGLLGFFGGFAYAPTTYFLPCCMWLAMCKPNRWSLEWTVNWICIILGVLLMLVGSIGGFWQIFVEAYAY

KFYPN*

>Sphfalx0014s0033.1

MSRAGARSAMEEPAPEKLNSTLEEAHEGSVLDHSNTGTTSKQTAWVTTGRGTWRHAGFHLATTIATPAAFAPLPSAVAAL

GWPAGVISLVMGTLVTSYCSLLLANLWDYNEPNRYVRYRDLGRAIYGRTGYWSVTVFQQIAAIGNNITIQIVAGLSMQAI

FVTYNTSDPSRVTLQEFIIIFGAAQLVLSQLPDIHSLRWFNALCTFCTVAFTIVVMGLLIHAGQNRDGPTDYGVHGTPSN

KVFGIFLALGTIAFSFGDAMLPEIQATIREPAKKNMYKGICLAYSVITTTYWLVAFLGYWAFGFAVQAYVVNSFSGPNWA

ITMANVFAVIQVAGCFQIYCRPTYQYFEFQLMNPKQHRWSLYNSLARLLVTSIYTALVTLIAAAMPFFGDFVALCGAIGF

TPLDFIFPILAFLRVKKPKSRIFWAFNIGIVVVYTLVAILGAIGSIRYIVKDTVRYHFFQNQ*

>Sphfalx0005s0085.1

MEGHVPLSEDVDYNGKLNDLLPVTATREAKWYYSTFHNVTAIVSAGVLGLPTAMADLTWGPGIVLLILSWVITLFTLWQM

VEMHEIVPGKRFDRYHELGQEAFGKRLGLWIVLPQQLLVQVSVDIVYMVVGGQALKNIYMLNCPGCSSKNVGSDDIAEIQ

YESVSLWILIYGSVHLLLVHLPNLNSIAALSLAAAIMSVSYSTIAWAIPVNKGLIQPPDYHLPYYGEPSPAPGPEPPATA

NAHTAHQVLSIFNALGVIAFAYAGHNVVLEIQATLPSTPERPSKIAMWRGVVWAYVIVAACYFPVAIICYWAYGNQLEAY

SNILQFEGMLGQNYKGILTAANVMLIIHILGSYQIYAIPVFDMLETVLAKKWLLPPSLKLRLITRTTYVGFTMFVATIFP

FFQALLGFFGGFAFAPTTYFLPCCIWLIVCKPKRFSMSWTINWICIILGVLLMCAATIGGFWAIVHEWSNYQFSGFWKWQ

DCPGVNSAQNCTAPTPAPL*

>Sphfalx0005s0083.1

MEGHVPLSEDLDWNRRLNDVLPVTASRNAKWYYSIFHNVTAIVSAGVLGLPTAMADLTWGPGIVLLILSWVITLFTLWQM

VEMHEMFPGRRFDRYHELGQEAFGKRLGRWIVIPQQLLVQVSVDIVYMVAGGQALKNIYMLNCHGCSSKNVGSDDIAEKQ

YESASLWILIYGSVHLLLVHLPNLNSIAALSLAAAIMSVSYSTIAWAIPVNKGHHQPQDYHLPYYPEISPAPGPEPPATA

NAHTAHQVLSIFNALGVIAFAYAGHNVVLEIQATLPSTPERPSKIAMWRGVVWAYVIVAACYFPVAIICYWAYGNQLAAY

SNILQFEGMLRHNYKGILTAANVMLIIHILGSYQIYAMPVFDMLETALAKKWLLPPTLKLRLITRTTYVGFTMFVATIFP

FFKALLGFFGGFAFAPTTYFLPCCIWLIVCKPKRFSMSWTINWICIILGVLLMFTATIGGCWALVNEWNSYQFRRFWKWQ

DCPGVNSAQYCTAPTPAPH*

>Sphfalx0362s0005.1

MSKGEADTPNQHKISVAVHQNGTSTDNNDDHASSSTDNNNLYMTSASNVDRLNAGAKFVLESKGNWWHAGYHLSASMAAP

TLLSLPFALDGLGWVPGFLALTIATVVSFYAYTLISKVLEQAELEGHRFLRFRDVAGYFLGRRWGYYPIGAIQIALCIAA

AVVCVLLGGESMQIIYQIYKPNGSMQLYEFIIIFGILMLLLSQLPSFHSLRHINLASLVFCLGFSLCVVGGSIYVGHSKQ

APAKSYSVEGSSVSKMFTIFNSLAIIILNFGIGIIPEIQATLAPPVSGKMFKGLLICYAVVISTFFSVAGAGYWAFGNAS

AGNILINLAPSGGVALIPNWLLFLANIFVIADLFAVALVYSQPIFEIFEGRLSSVKSGKLSMRNVLPRFIIRSLYVSFAT

VIAAAFPFFGDINAIIGAFALTPLDFILPFLLYNVTFKPSIWTIKFWLNSVIIVVFTIVGLMGCISAVRQIVLDVSSYKL

FANV*

>Sphfalx0333s0002.1

MMKGEVDTPNQHKISMPVHENGKVMNKDGHESSGTDNKNFYTSATKVDELNAGAKFVLESKGRWWHAGYHLTVSIAAPAL

LSLPFALDGLSWGPGFVALIIATGVSFYAYTLISKVLEQAEFEGHRFLRFRDVAGYVLGRRWGFYPVGALQIAVCLGTVV

GSTLLGGESMQIIYLIYKPNGLIQLYEFIIIFGTLMLLLSQLPSFHSLRYINLVSLVCCLGYSLCVVGGSIYVGHSRQAP

SKSYSVKGSSVTKMFTIFNSLAIIITTFGNGIIPEIQATLAPPVSGKMFKGLLMCYAVVIATFFSVAGAGYWAFGNASAG

NIFLNFAPSGGVQLIPNWLLFLANMFVIAELFAVALVYSQPTFEIFEGRSSSVESGKFSKRNLLPRLIIRSIYVSFATLI

AAALPFFGDINAIIGAFGFTPLDFVLPFVLYNVTFKPSKRTIKFWLNYVIIVVFTIVGLMGCISAVRQIVLDASSYKLFA

NV*

>Sphfalx0362s0007.1

MSKGEADTPNQHKISMAAHQNGTSTDNNDDHASSSTDNNNLYMTSASNVDGLNAGAKFVLESKGNWWHAGYHLTVSIAGP

SLLSLPFALDGLGWVPGFLALTIATAVSFYAYTLISKVLEQAELEGHRFLRFRDVAGYVLGRRWGYYPIGAIQIALCIAA

VVACVLLGGESMQIIYQIYKPNGSMQLYEFIIIFAILMLLISQLPSFHSLRYINLASLVFCLGFSLCVVGGSIYVGHSKQ

APAKSYSVEGSSVSKMFTIFNSLAIIITTLGNGIIPEIQATLAPPVSGKMFKGLLMCYAVVISTFFSVAGAGYWAFGNAS

AGNIFTNLAPSGGVALIPNWLLFLANIFVIADLFAVALVYSQPTFEIFEGRYSSVKSGKFSMRNVLPRFIIRSLYVSFVT

VIAAAFPFFGDINAIIGAFACTPLDFILPFLLYNVTFKPSIWTIKFWLNYVIIVVFTIVGLMGCISAVRQIVLDVSSYKL

FANV*

>Sphfalx0025s0047.1

MNMEGKDHARDFKMQEANGLTNGFNSLDVVSPSPSLSQEEYRHSSLSDARKDVGTLKVLESKGTWLHAGYHLTTAIAAPS

LLSLPYAFSFLGWAPGLLAITICGLVSSYAYCLLSQVLDDCASKGHRFYRFRELSQFVIGQKWTRFFVMPVQFGVCFVTV

IGAILAGGSAVKLIYKGANANGSIPLAEFVAMFGAVMILLSQLPSFHSLRYINLVSVILCLIYSLAATVGSVLAGYNKHI

PPKDYSVVGNPTDKLFGVFTSLSVMAGVYGVAIIPEIQATIAPPIGGKMVKAIALCYTVVVATFYSVSIAGYWAFGNDAQ

GNLFDNLVPSYGPQLNPTWLVAISGFSIVAQLLAIGLVYLQPAFDVFESLTADANCGRYTLRNVVPRLVLRSTYVSLATF

LAAMLPFFGDIVSLVGAFGYTPLDFVLPMLFYQLVFKPSRRTYMFWLNWVIIVTFTTVGIVGCIATVRHIAIDANNYKLF

ANV*

>Sphfalx0003s0314.1

MMEETGNGMQMAELTTKYTPLDGSGGSSSHSQEDEEQQQQRRRPYSSLSDAADAPKDAGTLFVLESKGTWMHAGYHLTTA

IAGPSLLSLPYAFSFLGWAPGLLALTICGLVSSYAYCLLSQVLDDCASKGHRFYRFRELSQFVIGKSWTNCFVTPVQFGV

CFVTVVGAILAGGFAVKLIYLGVNANGTIPLAAFVAMFGAVMIVLAQLPSFHSLRYINLVSLLLCLTYSLCATAGSVLAG

YNKNVPPKDYSVVGNPAEKMFGVFTALSVMAGVYGVAIIPEIQATMAPPIVGKMVKGIALCYVVVAATFYSVSIAGYWAF

GNGAQGNIFDNLVPSGGPQLNPVWLTAISSFAIVAQLLAIGLVYLQPTFDVLETLTADVNRGKYALRNVVPRLVLRSTYV

SLATLIGAMLPFFGDIVSLVGAFGYTPLDFVLPMLFYQLVFKPSRTTYIFWLNWVIIVSFSIVGVIGCIATMRHIVIDAK

TYKLFADV*

>Sphfalx0026s0089.1

MTFNGSGGLPCEEQFAGRFQKMNAELQLGAPKKSVMPSLDRVGSDHQHDAGQLRGDPESGHHDAAVDHHLQAAGSYKSQG

SQLLVSRQTVHCVGQDAWWEVGFHFIAAVNNAFILGYPALIMAYLGWAMGSICLIGGGIISFYNNCLLGSLHETGGKRHI

RYRDLAGHIYGKGMYRATWFVQYFNLSIANIGTIILAGEALKAIWGAFSDNTSIKLAGWIVIAGICFGLFAFVVPNLHAL

RFFSTCSLLLSLIYTCIAIAIAFSDGLRSAPRDYSLKGTKADRTFNAIGALATIAFAYNTGILPEMQSTIRQPTTTNIYK

ALGMQFTVGTFPFLVLTFVGYWAYGNAANPYLLLSLGGPKSLVTIANAAAFLQAIVSLHIYATPMYEFMDTYFARKDQSE

WSLHSMLVRFITRGTYITVSTFLGALLPFFGDFIALTGAMAAFPLESGIVHHMYLKVKGKGFSTWRLIWHWSIVVISAVL

TIVTCVAAIHYIISDSTYYHAFADI*

>Sphfalx0018s0093.1

MTFKEGLELMVDPNMNAAAPQGGLLSKSTSLHQQQPSRLSCNVQQGAGGVDLLLHEHQDVQLGPGGDPEAAAGGTTGTPG

SSTTGSSGKSVRPLLVSRDTVHRVGQDSWWEVGFHFIAALDNAFILGYPALIMAYLGWATGTICLIGGGIVSFYNNCLLG

SLHETGGKRHIRYRDLAGHIYGRGMYRATWFVQYFNLSIANIGTIILAGQALKAIFGAFSDNTSVKLAGWIVIAGVCFGL

FAFVVPTLHALRFFSSCSLFLSLIYTCIAIGVSFSDGLKSPPRDYSLKGTRADRTFNAIGALATIAFAYNTGILPEMQAT

IRQPTTTNIYKALGMQFTLGTFPFLLLTFVGYWAYGNTASIYLLSSLGGPKSLVTIANAAAFLQAIVSLHVYATPMYEFM

DTHFARKDRSEWSVHSMLVRFITRGTYITISTFLGALLPFFGDFITLTGAMAAFPLESGLIHHMYLKVKGKGFSKWRLLW

HWFIVVISAILTVVTCVAAVRYIIQDSTNYHAFANL*

>Sphfalx0007s0047.1

MVGTKVSGAAMTFCEGGDELTGFQKMAVVDQGVKQPLPSLNVGGGNVQNGQLGGDPEAGHGGGGHYHHEASKSGWSVPVS

RETVHRVGQDSWWEVGFHFIAALNNAFILGYPALIMAYLGFATGSLCLIGGGVISFYNNCLLGSLHETGGKRHIRYRDLA

GHIYGRGMYRATWFVQYFNLSIANVGTIILAGEALKAIWGAFTDNTSVKLAGWIVVAGVCFGLFAFVVPNLHALRFFSTC

SLFLSLIYTCIAIGVAFSDGLKAGPRDYSLKGTKADRTFNAIGALATIAFAYNTGILPEMQATIRQPTTTNIYKALGMQF

TVGTFPFLVLTFVGYWAYGNTANPYLLLSLGGPKSLVTVANAAAFLQAIVSLHIYATPMYEFMDTHFARKDQGDWSAHSM

LVRLITRGTYITISTFLGALLPFFGDFITLTGAMAAFPLESGIIHHMYLKVKGKGFSTWRLTWHWCIVVLSGVLTVATCA

AAVRYIISDSIYYHAFADL*

>Sphfalx0075s0052.1

MVGEKVLGSAMMSMVVHEEGTADPPAGLKSTVPSLVCSVGGYNNQQSDDAGPLDHGNPEAAAAHGAAGDYDHHVEAHAGS

SKQFMSSGALVPPPVSRETVHRVGQDSWWEVGFHFIAAVNNAFILGYPALIMAYLGWATGSLCLIGGGVVSFYNNCLLGS

LHETGGKRHIRYRDLAGHIYGRGMYRLTWFVQYFNLSIANVGTIILAGEALKAIWAACSSTDNSSNLKLATWIVIAGICF

GLFAFMVPTLHALRFFSTCSLLLSLIYTCIAIALAFSNGLKSPPRDYSLIGTKADRTFHAIGALATIAFAYNTGILPEMQ

ATIRQPSTTNIYKALGMQFTVGTFPFLVLTFVGYWAYGSAVQPYLLLSIHGPKSLITIANSATFLQALVCLHIYATPMYE

FMDTYFARTKDESDWSVHRMLVRFITRGTYITISTFLGALLPFFGDFITFTGAMACFPLESGIIHHMYLKVKGKGFSKWR

LAWHWFIVVLSGVLTVATCIAAVRYIISDSVSYHAFADIQQV*

>Sphfalx0168s0015.1

MAMSNGGIENHNGKENTNHLVQGGLGDSVSQLTHRDPELADGNHHVENSNSDTPGTRLSRETVHHVGKDTWWEVGFHLIA

AFDNSYVLGYPGLVMAYLGWIAGPICVGVFYVGSFYNNYLLATLHETGGKRHIRSRDLAGYILGPLVYKATWILQYSILS

VSTIGSIILCGESLQGIWVAYSHNPSAITLPVWVVISGGTYALFAFFVPTLHSLRPFTAVSIFLSLIFICIAIGTSFNDG

FRAAAPRNYSLLGTKADVSFRSIGSLATIAFAFNSNILPEMQAVVRQPTVRNTHKALVMQFTLGTFPIILVMMVAYWAYG

NTVNTYVLNSTSGPRPWVALANVAAFLQMIISIHVYALPMYEFVDTFFGRNYDGKGDWSGHSALIRFLTRGTFIAIATFI

GALLPFFGDFVALTGAFSVFPLNFGLVHLMYLKVNGKNFMPYRVAWHWIMIILAIILTVATATASIRQIISDATTYHVFA

NS*

>Sphfalx0193s0030.1

MAMSNGGIENHNGKVDTNHLMQRGIGNSASQLTHLDPELADGNHHVENSNSDTPDIGPSRETVHHVGKDTWWEAGFHLIA

AFDNSYVLGYPGLVMAYLGWIAGPICVGAFYVGSFYNNYLLATLHETGGKRHIRSRDLAGHILGPLMYKATWILQFLNLS

IGTVGSIILCGESLQGIWVAYSHNSAITLPIWVVISGGTYGLFAFFVPTLHSLRLHTAVSIFLSLIFICIAVGTSVNDGF

RAAAPRDYSLLGTKADVSFRSIGSLATIAFAFNSNILPEMQAVVRQPAVRNMHKALVMQFTLGNFPIILVMMAAYWAYGN

AVNPYLLNSTSGPRPWVALANVTAFLQMIVSIHVYALPMYEFVDTFFGRNYGDKRDWSAHSTLIRFLTRGTFIAIATFFG

ALLPFFGDFVALTGALSVFPLNFGLVHLMYVKVNGKNFALYQVAWHWGMIGLAVILTVTTATASVRQIISDATNTHVFSN

T*

>Sphfalx0065s0034.1

MDNNGAGGVQGPHVVSMKRAGSLRGPAENDYSVVNLGAVAPIPADGDPLPFTDGDSNPGKMKNLPPSSATAHEIGRDPWW

VVGFDLLAASSSSYILSYPQLIMVHLGWIAGPIIMVLLNAAFFYNNCLLGSLHETGGKRQIRTRDLVGYIYGRPMFTAVW

ILQFTVLIVFCIGTFVWAGTTLETIYSSYSSDPSKISLSEWVAVAGACYSVFAFAVPTLHSLRLYSTISLVLTMIITFIT

IGISIKDGVKSGVARDYSIVGTKADKWFSAMGALSIVGFAFNTAILPELQASVKPPTVRNIYKALGLQYTVGAIPVIGLT

LIVYWAYGNVVSGFALESNSGPKWLVTVAKVAAFFQILVTLHIYALPMFEFLDSKFLKAYRSLKPSGNVQNDVPQTGRNW

SARTMLVRFLTRIMFISITTLFGAMFPFFGDILELGGALIVFPLDFAVVHHMYMKVKGKNFSWYRRTWHWLMIVIAVLLT

IASVTGAVRNIIASSSTYKIFHSNN*

>Sphfalx0065s0028.1

MAMNNIGAGGVQGFHVTSMERARSLRTPAENDHSVVNLGAVAPVATDADSLPVTVGDTNSGEMVNPAAPAASAHEIGKDP

WWVVGFSLVAASDSSYMLTYPFLIMSQLGWIAGPIILILLTALSCHNNCLFGSLHETGGKRHIRTRDLISYVYGRRWLTR

TVWIVQFTVLIIFCIGTFIWAGTSLQAIYVTYSNDPTKISLPVWIAIAGACYSIFAFFVPTLHALGLYTAISLLLTMIIT

FISLGISIKDGMKSGVKRDYSLVGTTADKWFGALASLSIVSFAFNIPILPELQANVRPPTVRNIYKALGFEYIVGGTPVI

VLVFVAYWAYGNGVSEYLLYSTSGPTWLVTVANVAAFLQVLVTIHIFALPMFEFFDSMLSKLKRRHAEGDVADDHTLRAR

TGPWSKHKMLLRLVTRTTFISITTLVGAMFPFFGDILELGAALIVFPLNYGLVHHMYLKVNGKTISWYQRAWHWSIIVAA

VVLTVSSVTASTRNIIVSSSTFHIFKSSS*

>Sphfalx0193s0031.1

MAMSNAGIENHNGKDDTNHLVQRGIGNSASQLTHLDPELADRNHHVENSNSNTPDIRPSRETVHHVGKDTWLEVGFHLIA

AFDSSYVLGYPGLVMAYLGWIAGPICVGAFYVGSFYNNYLLATLHETGGKRHIRSRDLAGHILGPLMYKATWILQFLILS

ISVVGSIILCGESLQGIWVAYSHNSSAIMLPIWVVISGCTYGLFAFFVPTLHSLRLHTAASIFLSLIFICIAVGTSFNDG

FRAAAPRDYSLLGTKADVSFRSIGSLATIAFAFNSNILPEMQAVVRQPAVRNMHKALVMQFTLGTFPIILVMMTAYWAYG

NTVNPYLLNSTSGPRPWVALANVTAFLQMIVSIHVYALPMYEFVDTFFGRNYCDKRDWSAHSTLIRFLTRGTFIAIATFF

GALLPFFGDFVALTGAISVFPLSFGLVHLMYLKVNGKNFTLYQVAWHWVMIGLAVILTVATATASVRQIISDATTYRVFS

NT*

>Sphfalx0257s0017.1

MAMNNNGAGGVQGLHLISMTRARSGRRPAENDHAVVNLGAVAPFPADGDSLPFTVGDSNPGKMKNLPPSSASPHEIGQDP

WWVVGFNLVAASSSSYILSYPQLIMTHLGWIAGPIIMVLLNAVFFYNNCLLGSLHETGGKRQIRTRDLVGYIYGRRMFTA

VWILQFTVLIVFCIGTFIWAGTTLEAIYASYSSDPSKISLSEWVAIAGACYSVFAFVVPTLHSLRLYSIISLFLTMIITF

ITIGISIKDGVKSGVARDYSIVGTKADKWFSAMGALSVVGFAFNTAILPELQADVRPPTARNIYKALGLQYTLGAIPIIG

LTLIGYWAYGNVVSGFILESNSGPKWLVTVAKVAAFFQILVTLHIYALPMFEFFDSKLLKAKRSLKPSGNVENDVHQAGG

HWSARTVLVRFLTRITFISITTLLGAMFPFFGDILELSGAVIVFPIDFGLVHHMYLKVKGKNFSLYRRTWHWLMIVTAVL

LTIASVTGASRNIIAVASTYRIFHSNN*

>PpAAP9B

MMAQWHGDHDKLELGRVNACIVNAAPPYDDSNKFDDDGKTRRTGSFWTASAHVITAVIGSGVLSLAWSMAQMGWVAGPLV

LLLFSFVTYYTSSLLADCYRHPDPVTGKRNYTYMDAVKANLGPRQVLLCGVVQYANLLGTSIGYTITAASSMVAITRSDC

FHHKGTKGPCQASNIPYMSMFGFVQIILSQIPEFGELWFLSVLAAVMSFLYSTIGLGLGIAKAVDHQHGYGSITGISVGD

PSVGYVSMSNKIWGICSALGNIAFAYSFSMILIEIQDTLKSSPPENKTMKRASLFGIITTTIFYMSVGCAGYAAFGDNAP

GNLLTGFGFYNPYWLVDFGNACVVVHLVGAYQVYTQPLFAFFENTLSSRWPKSQFIHKEYYLKVPWGEPLHFNLFRLVWR

SMYVVVTTVLSMVLPFFNDVMGLIGAFAFWPLTVYFPVQMFIVQRQVQRWSPKWCWLHLLSVSCFAVSLAAALGSSECMI

SDLKKYKPFQG*

>PpAAP9A

MDGDHVILGLTGNAKLGQTDDNALGHGEKGRAGFDGVDKPIHDPNLNDDDGKPRRKGTVITSAAHIITAVIGSGVLALSW

SFAQMGWIAGPIVLLAFAWCTYYTSRLLADCYRSPDPIHGKRNYIYMDAIKANLGRKQQLVCACVQYSNLIGTSIGYTIA

TATSAKAIQYQNCIHDNGPDDPCLTSTTVYIAIFGVIQIVLSQIPNFGELWWLSYLAAAMSFTYSFIGLGLGISKAATGE

NSHGSLGGTSVCYPSNGETCFTRPQKTWNVFTALGNMAFAYSFSMILIEIQDTIKSPPSESSQMKKATLLGIITTTFFYM

SVAIAGYAAFGDAAPGNLLTGFSTPYWLVDFANTCIVIHLIGAYQVYTQPVYAFVERWCSLRWPNNSFLNLEYNVRLPGR

RNFRVSAFRLIWRTIYVIITTIISMLIPFFNSVLGILGAIGFWPLTVYYPVEMYIRQTHVQRWSHFNRRLQTSILFNLLV

LLCDGYYEGG*

>Pp3c13_12390V3.1

MANPSTREAFQEHMQENGHKKVKTVDEWLPVTGDRKAKWWYSAFHNVTAMVGAGVLGLPNAMVYLTWGPGVVVLVVSWMI

TLYTLWQMVEMHEMVEGKRFDRYHELGQEAFGHDLGLWIVVPQQLIVEVGVDIVYMVTGGTSLQNFYKLVCSGNCPMAHH

TSAWIAIFSSVHFVLAQLPNFNSIAGVSLAAAIMSLSYSTIAWAIPASYGHSTPLVGPVNYKLPVQSVSAHVFNAFNALG

TVAFAYAGHNVVLEIQATIPSTKERPSKIPMWRGVVLAYIIVAICYFPVALIGYWAYGNQVTDNILGYVGRPRGVVAMAN

LMVVVHVIGSYQIYAMPVFDMLESVLVKRFRLAPSRKLRLVTRSLYVAFTAFVGMTFPFFGALLGFFGGFAFAPTTYFLP

CIMWLCIVKPKAFSFSWILNWVIIFLGVLLMLVSSIGGLRAIIVSASTYKFYE*

>Pp3c14_9480V3.1

MTLGRMDSSLDTHNIELQKQSSVLLAPPQRSENSQNTTDLEAWLPISTADRNANWKHAAFHNVTAMMGAGVLALPNAMVY

LTWGPGLLMLILSWVITLFTLWQMVEMHEAVPGKRFDRYHELGQEAFGPKLGLWIVVPMQLVVEVGVDIVYMVTAGKSMQ

HAYNITCGDHCPLQDAIVFWIFLFAIVQLVLAQLPNFNSITAISLAAAIMSISYSTIAWIIPAHYGHTLPGGVKLLAPVS

YGLPQFKPVQLQLPGGSTLQQVPDDLSYNDRLFGAFTALGTIAFAYAGHNVVLEIQSTLPSTPEEPSKLAMWRGVKFAYG

VVAAGYFPVALVGYWAYGNQVTDDIITFVSRPTWLVLIANLMVVVHVIGSYQIYAMPVFDMMESTLVGRLRFKPSTPLRL

ITRSLYVVFTMFIAITFPFFSALLGFFGGFAFSPTTYFLPSIIWLRIYHPNRWSWSWVINWAVIVFGVVLMFVSTIGGFR

SLMVEAANFHFYKN*

>Pp3c17_11220V3.1

MDVPLDTYHIELQKQSSLLLAPPQRGVSGDRLIDLESWLPITAADRSANWKHAAFHNVTAMMGAGVLALPNAMVYLTWGP

GILMLILSWIITLFTLWQMVEMHEAVPGRRFDRYHELGQEAFGPKLGLWIVVPMQLVVEVGVDIVYMVTAGKSLQHAYSI

TCGDHCQLQDSIVFWIFLFAIVQLVLAQLPNFNSIAAISLAAAIMSISYSTIAWAIPAHYGHTLPGNIELLQPVSYDFPP

FKPVQLVQAGGSAVQKAPEDLSTADRWFGAFTALGTIAFAYAGHNVVLEIQSTLPSTPHEPSKIAMWRGVKFAYGVVAIG

YFPVALIGYWAYGNQVTDDIITFVSRPTWLVVIANLMVVVHVIGSYQIYAMPVYDMLESTLVGHLRFNPSMLLRLVTRSL

YVSFTMFVAMTFPFFAALLGFFGGFAFSPTTYFLPSIMWLMIYRPSPMSWSWITNWAVIVFGVVLMFVSTIGGFRSLMTE

AANFHFYT*

>Pp3c11_19940V3.1

MQRGSNMDKVSSIEGEKKFKGDVDGPGSLPGSLVKHPEGRGTWPISAFHLATTIATPAAFAPLPYAMSQLGWIGGVVTLL

VGTAVTYYCTLLLASLWDWDEPNRYVRYRDLGRSIYGAKGYWSVLAFQQIASIGNNITIQIVAGLSMKSIYTTYSSNPSG

MTLQHFIILFGVVELFLSQFPDIHSLRFLNALCTGCTIGFSVSVVALCAHALRNGDADGSSYDIVGSPSDKTFGIFAALG

TIAFSFGDAMLPEIQATLREPAKLNMYKGSTLAYTVIAVSYWTVAFMGYAVFGNTVNPYLVNSFFGPDWLITLANIFAII

QVLGCYQIYCRPTYLYVEQQVMDYNKHPWSLQNALARVGVTATYIVVITVIAAAVPFFGDFVALCGAIGFTPLDFIIPVI

AFLKVRNPKNPLVKLINVAIVVVYSIVAILGAIGAIQFIHHDTNRYQFFANL*

>Pp3c3_11320V3.1

MDQAVKMAGEYGTTMSPEACGGEDKAIYGTAIIKDGGALFVLESKGNWKHAGFHLSTSIVAPALLSLPYAMKGLGWAPGF

LALIIGAVVSFYAYMRISKVLEQAELEGHRLLRFRDMGGYVLGRTWGYYPVSVLQIGLCLGAMIGCIVLGGQSMKLIYKV

FHPNGSMQLYVFTIIFGMVMAVFSQLPSFHSLRYINLLSLLCSLGYSLSAVGGCIYAGHSNEAPPRDYAVVGSPGSKAYG

VFNSLVIIATTYGNGIIPEIQATLAPPVTGKMFKGLLVCYAVVITTFFSVAAAGYWAFGNEAQGNIFINIEPFVPKWLNF

LSNALVLAQLLAVALVYAQPTFEIFEGKSSNIQKGKYSARNLVPRLILRSALVAITTLISAAIPFFGDINAVIGSFGFTP

LDFVLPFILYAGVFHPSPRTPKYWLHWTIVIVFSIVGLLGCVASVRQVVLVASTYKLFANIV*

>Pp3c8_19000V3.1

MRAFEVIGTGYSSLVRDRSAVEEEEGFEAKDAGALFVLESKGTWFHAGYHLTTAIAGPSLLTLPYAFHFLGWGPGLFALT

IAGAVSSYAYCLLSRVLEHYASQGKRCLRFRDLSDVVIGKRWTIWFVIPVQFGVCFVTLIGVILTGGYGCKLIYLGLVPD

GAIRLWVFVALFGAVMMILAQLPSFHSLRHLSLFSLFCCLAYSACAVIGSIIAGHNPNVPPKNYSVTGSPVQKVFGVFTA

ISIMAGVYGVALIPEIQATVAPPVTGKMQKGIALCYTVVLITFYPVAISGYWAFGNQASGNIVDNLAPDKGPDLLPTWLL

GILSIAIVAQLLAIGLVYLQPISEVLESKTGDAKQGKYSIRNVMPRLVFRSLYLAVVTLLAAMLPFFGDIISLIGAFGYT

PLDFVLPMLFYQIVFQPSRQKPIFWLNWTIIIVFTVVGVIGCIASFRSIYMNVQKYHLFGDV*

>Pp3c23_12700V3.1

MNGHQGYTRLEGSSEVEAFSSRPQMPSKDVDGGALFVLESKGNWKHAGFHLTVSIATPALLTLPFALRELGWVAGVLALG

LCAGVSFYAYNILSQVLENSERRGHRFLRFRDLGAHVLGPWGYYGIGGIQFLVCFGTVIGSCIVGGQSMKLIYSILEPES

TRQLSEFVAIFGIFMLVLAQLPSFHSLRYINLASLMCCLGFSLCVVGGCIYAGNSVDAPPKDYSISGTPASKLFGVFEAL

AIIATTFGNGIIPEIQATLAPPVENKMFKGLLVCYTVVVTTFFSVAISGYWAFGNQVAGYVLTNLAPTDGPALVPSWLIL

LANGFALAQLTAVALVYSQPTFEIFEGQTSDVKEGKYSMRNLVPRFLLRSSYVAFATFVSAALPFFGDINGVLGAFCFTP

LDFILPFIFYSFTFGPSRQTPRFWIHWGIVILFSVVGFLGCISSVHQVILDAKYYKWFADL*

>Pp3c9_4450V3.1

MAFSGADADEVAVTTAEQPPFFRVASSTETAHDQEAARSSDIIPLSKETFHRVGEDTWYEVGFHIIAALNTAFILGYPAL

IMGILGWIAGPICLVGGAVISFYNNYLLGGLHETGGKRHVRYRDLAGYIYGPTMYKLTWVAQFLCLIVINIGTIILAGLS

LKSMARAFSDGSEIVKLPGWIAVTGAVVCIFALMVPTLHALRFFSTCSLLLSSIYTFIAIVVAFKDGLKAEGPRDYSLRG

NVTDRTFNAIGALATIAFAFNTGILPEMQATVRQPTTRNIRKALGLQFTVGTFPILVLTFVGYWAYGNTVSVYMFSSVSR

PRSTAVTVANAVAFLQAIISLHVYASPIYEFMDTQFARKGDHEWSRHSVLVRFFTRTAYIGISTFLGALLPLFGDFIALT

GALVAFPLEWGLIHHMYLKVKGKEFGKGRLLWHWSMIVIAVVLTFTTATAGLRFIISDSILYHEFADL*

>Pp3c6_1540V3.1

MSTSGRKGSIAMTERSRALVETTALSTSSSPDGMAPPVQPIERPFIAAPPFSFPTASDTPTEASSVTMTPESSCDQTNFG

SNLIASPWRSPTNRAAGRPPSNFVSPIGTPLHRSLHNLQHYLEEGGHSTTLHVRDTWLPLTESRNGNMVYAAFHNLNAMI

GYQALFLPFAFIYLGWTWGLTVLCLAFTWQMYTKWQLIMLHETEPGKRIRNYVELSQEAFGQTIGFHTTIPAVLNLTVGT

SIGLVVVGGSALELFYLTVCHKCVDNPLSMIEWCIVFSALCLILAQLPNMNSIASVSLAGALMAVSYTTLIWMISVFKKR

PQDISYSLATKGDSPLVTTVAVLNAIGIITFAFRGHNLVLEIQGTLPSTLKEPSSISMWKGAKLANLVLVFCFFPLAIGG

YRGFGNKMLNSGILYSLQAADLSKTARGFLALTFLFVMFSCLSSFQIFSMPVFDMIEQFYTGKWNKKCSPCVRLFSRSVY

VLVVFFMAIAFPFLTSLAGLIGGLNSIPVTFVIPCFMWLSIRRPNKRSFTWCLNWFLAIFGIITSCLVSAASVGVIIQRG

IKLEFFKPHA*

>Pp3c21_14080V3.1

MAYAWYTVAFHIVTALNSGFILGYPALIMAYLGWTAGVLCLLGGGIISFYKNCLLGELHETGGKRQVRYRDLAGHIYAAA

FTVGRHVTLPGWVGVAGAVICVFAFLVPTLHAFRFFSTCSLLLSCVYIFTSVGIALTDGVKAKFSRDYSLKGSNTEKAFN

ALGAMATIAFAFNTGILPEMQATVKEPSVRNMKKALDLQFTVGTLPILMLTFVGYWAYGNDVVPYMLNSVSGPKSAVTVA

NAAAFLQTVVSLHIYCSHIYEFMDTSFSKKGRHEWSFYSITVRLIKRTTYISLSTFLGALLLFFGDFIVLTGAVAVFPPE

SGLVHHMYTKRLIWHWGMVIISAALTVGTVAVGFRFIVVDSINYPAFADL*

>Mapoly0047s0067.1

MSKMGSEEAKYSSVTGSEEKLDQQEQAKSEMQQKRSPFDGVESETGKLLDDDGRPMRTGNTWTATAHIITAVIGSGVLSL

SWSFAQMGWIAGPLVLFSFALCTWYTSRLLADCYRYPHPVTGRRNYIYMDAVKVNVSARSHLICGIMQYSNLVGTSIGYT

IATATSAVAVQKSNCYHSHGRDSPCLASTTSYIAVFGVIQIFLSQIPNFGDLWWLSYVAAIMSFTYATIGLGLGISKAAE

GGHSYGSVGGVRIGTDVTEAQKVWNIFAALGNMAFAYSFSMILIEIEDTIRSPPAENKQMKKATLWGTSVTTAFYMSVAI

AGYLAFGDAAPGNLLTGFFSPYWLVDFANVCIVIHLIGAYQVYTQPVYQFAERWVARRWSKRSFHSRGYTVRLPGGSNFR

LNWFRLTWRTIYVIITTIISMLLPFFNAVLGIIGALAFWPLTVYYPVVMYMNQHKIPRWSPTWIALHSLSFVTFLVSLAG

LIGSVAGIVNDLSQVKPFEKI*

>Mapoly0134s0048.1

MSEETMPASKNRAAMDITHLGLPNRHVDVDGLLTARNSVDFTTVFVDPKSQRAGGLDAHDRTPRTGTMWTATAHVITAVI

GSGVLSLAWAISQMGWIAGPLVLLAFGGITYYTSVMLADSYRFPHPETGKRNPTYMAAVKASLGSREVKACGILQYMSLF

GTCVGYTITTSHSLEAIFRAGCYHAEGHDAECKTSLSYYMLGFGATELIFSQIPNFDKMSWLSIVAAVMSISYSGIGLGL

GIAKAAEHGPRGTLGGLDIGRGPGEVALIEKLLSVANALGNMAFAYSFSTILITIQDTLKSSPPENKIMKKATLIGITTT

TLFYISVGCAGYAAFGNEAPGDLLTDFGFYEPWWLVDFANACLVVHLIGAYQVFAQPVFAFVEGWITKRRPDSHFIHKNF

DLVLPWGSTYHLNLFRLTWRSGYVAVTTLLAILLPFFNAIMGLLGAIGFWPLTIYFPIQMYKKQAAINFGSKKWYMLNAV

SAICLLVTLAAAVASIQSIIQKSKEYKPFSS*

>Mapoly0004s0095.1

MEDSESSKTTQELRMSRIVDRIDQSVDRNVEGTNLVPMSSAELDDDGRPKRTGTFWTASAHAITACIGSGVLSLAWGVAQ

LGWIAGPLVNVSFAVITYFTSVLLVDSYRYMNPVSGRRLYTYMDTVKSYLGRKDTFVCGLVQYTNLFGICIGYTVTASAS

MVAIKRAGCFHERGRHAECHVSNNLYMGIFGAAQIFFSQIPDFAQTWWLSVLAAIMSFSYSGIGLVLSLIKFFDEGQVRG

TLYGWPIGNGQDEYSRAQKVWKTLSAVGDIAFAYSFIIVLLNIQDTLKSPPPENKTMKKVALASNLTCSIFYLSVGCVGY

AAFGNHAPGNLLTGFGFYEPYWLIDFANACIVVHLVGAYQIFAQPLFSFLEGLVLKRWPKKTFLNNTRSVSIRVVGRIRL

NIFHLVWRTIFVLVTTLLALLLPFFNSIMGLLGALAFFPLSVYFPIRIYMKRTNLRHYKCEWWFLCTLMVICLLVSIGAA

IGSVAEILQDTRHYKPFN*

>Mapoly0170s0014.1

MDLTEISEGADDSRKFDDDLKPARTGTWLNATAHVVTAVIGSGVLGLPWSIAQLGWVVGSIMLIFFAWATFYTSALLADC

YRHHVTGRRNYTYMDAVNSHLGKKQVYLCAIAQYLNLLCTSIGYTVTSATSMVAVKRAICFHRNGHEAHCHVSNIPWMIL

FGGIQIFLSQLPDFSHLKFVSIVAAVMSAIYSFIGLGLSVARVSINKHAKGPVWGTQLSAAQKTWSVFQALGNIAFAYSF

CMVLIEIQDTVRGPPKYAMENVTMKKATIWGISITTAFYTTVGCLGFAAFGIHAPGNILTGFGFYNPYWILAIGNICVVI

HLVGAYQVYVQPIFAALERVVHNNWPRKNLIGFRLITRTLFVIAVTLVASALPFFNDINGFIGAISFFPLTVYFPTAMYI

KSKGKALTSTKELTMIWALRIISGLITLVAIIGSVEGIRSSVQGTKLFHTKN*

>Mapoly0130s0012.1

MDVVEISEAADDIKELDDDLKPARTDDIKELDDDLKPARTGNVFNATAHVVTAVIGSGVLGLPWSIAQLGWVAGVIMLVF

FALATLYTSALLADCYRDPVTGKRSYTYMDAVNCHLGKKQVYLCAIAQYLNLLCTSIGYTITTATSMVAVKRALCFHTNG

HDAHCHVSNIPWMIVFGGIQIFLSQLPDFSHLKFVSIVAAIMSAIYSFIGVGLSIARMAIRNVSGPVWGTQLSPAAKTWS

VFQALGNIAFAYSFSMVLIEIQDTLRGTPKYAPETVTMKKGTKWGIGITTAFYTSVGCLGFAAFGQHAPGNILTGFGFYN

PYWILAIGNICVVIHLIGAYQVYVQPIFAASERALRINWAGMKLIWFRLIFRTLFVIAVTFVASALPFFNDINGFIGAIS

FFPLTVYFPTAMYIKSKGKSLTCTREVIIIWVLRIISGLITLVAMIGSVEGIQSSVKGTKLFHTKN*

>Mapoly0052s0005.1

MAPREDSAAETELARPPGSHAPNKDENDVDGPRHPGTIKNVDDWLPVTGSRNAKWWYSTFHNVTAMVGAGVLSLPSAMAY

FGWGFGTVVLVGSWCITLYTLWQMVEMHEMVPGKRFDRYHELGQQAFGPRLGLWIVVPQQLIVEVGVDIVYMVTGGDSLQ

KFHNLIKCPSSASNPDEHCGHIGKSAWIIIFASQHFLLAQLPNFNSIAGVSLAAAVMSLSYSTIAWAAPLAQGRAAVHDY

GLPDKTTATLVFGVFNALGNVAFAYAGHNVVLEIQATIPSTPERPSKIAMWRGVVLAYIVVAACYFPVAIVGYWAFGNTV

KDNVLLQLGKPRWLIAMANFMVVIHVTGSYQLYAMPVFDMIETVLVKKMHIPPSAPLRVVVRCLYVAFTAFVACTLPFFG

ALLGFLGGFAFAPTTYFLPCIMWLVIYKPKRFSLSWCANWVCIVIGVLLMLVSSIGGLRNIIVSASTYKFYQ*

>Mapoly0032s0116.1

MVALETEESGSGSLKHGALESREQWEPKRDAGALVVLESKGSWQHVGFHLTASIAGPPILTFPFAFAALGWAWGMIALVS

AGVVTFYSYNLLSLILEHRAAQGRRHVRFRDLATDILGPKLSYGLVYPMQFVVCLGAVISIVLVGGISAKVVYKVYYPDG

DLQLYQFVVFFGAATMIMGQLPSFHSLRYVNLLSLLMCLTYSFTITGACIQLGYSDRAPPRDYSLPGSEKQQAFAAFNAL

ALIATAYGNSIIPETQATLAPPVEGKMFKGLAMAYSVIVVTFLPVAIAGYWTFGNLSAPSVFTNFQTADGTLLVSKWLIV

MPSSMCVIQLISAAIIYSQPTFDLFERKIADVDKDRFTARNGIPRVIVRVVFLALVTLIAAMFPFFGDVNAIVGSFGFTP

LDFVFPMVFYILVFKPPKRSIKFWGNLIIIIVYSLVGLAGAVAAVRQLILDTSYYKLFADV*

>Mapoly0032s0115.1

MVADETVPSALELGNGTLKQRHHQADPDAANKDAGALFVLESKGTWKHAGFHLSSSIAAPPLLTLPFAFVALGWEYGVLM

LVLGAAVTFYAYNLLSKVLEHLAEQGRRHLRFRDLATDVLGPTLSKWIVYPTQLVVCIGVVIASPLLGGISIKIIYKLYA

PDGDLKLYHFIIIFGIGLIFVGQMPSFHSLRYINFVSLVMTLCYSFTVSGACIYLGKSDRAPAKDYNLPGSETQKVFGAF

NSLAIIATSYGNGIIPEIQATLEPPVTGKMFKGLTVAYAVVISTFFTVAISGYWAFGNSAAGSIFTNFLEADGTPLVPKW

LIVMPNVLCIIQLLSVSIIYCQPTFDLLEGKSADVEKGRFSARNWIPRLILRTTFLCACTLIGAMFPFFGDVNAIIGAFG

FTPLDFVFPMLFYILVFKPPKRSIKFWGNLILLVAYTGVGVVGAIAAIRQLVLDTSYYKLFADI*

>Mapoly0040s0055.1

MEEEHRRRPQSPATVHEVMESDGDSGTVTPTGHSRELAGYVPKSSPHSVAGSTPAASPRHRLDELDTLPAPPFRPMASEL

QHSPSLSDGIFPVAGHTPELDRQRSTASLIPRSGPSQLRPPPTPPKSPATPLSPATPKSPVKAFASWATSPLRSPLPSPL

ATTLRNMKEYLQELGHLTTIDPRDTWLPITESRHGNAYYAAFHNVNASIGFQALLLPLAFTFLGWTSGIVCLIIAFCWMM

YTKWLLVSMHECVPGKRVNRYLDLVEIAFGNGIGKQIIVIQLLILLGGTCIGLITIGGSCLQLFFRTVCPTCQSPLTPIE

WYLVFTILCALLSYFPNLNSVAGVSLIGATMAVAYTTLLWTLSVSVPRMPNVGYEIVSGGSALVTTFGILNALGMIVFAF

RGHNLVLEIQATMPSTLKHPARVPMWRGATAAFALTALCYFPLAIGGYWAYGDKMLPGGILYSLSFFHGQDISRTLQGTT

FMFIVVNSLGSFQVYAMSIFDMIEQVYSKWFDRKCNALLRLIYRTVIVFLCFLGAVAIPFLSTMAGLIGGLTNLPVTFFL

PSLMYLKVVAPRPRSFSWYLNIGLGTFGCALSIAVCAGGVYSIIETGIKLNFFKPA*

>Mapoly0107s0041.1

MRWEQLRSERGEGQETEEMDSSSGGEGSYGGREGPRVVTVREVSFAPNVRSEVSSRSDEPELISIPVTPQGSTPPSQASP

RIFSPGILTPSGAQTPRGLALGAMGAMGASIPGSSKAPSSLPTPSLPTPKTPWTPGLRSPRFLGTPLATPMRRAFVNMKQ

YLEDIGHFTTLNPRDAWLPVTESRNGNVFYAAFHTLNASIGFQSLFLPVAFMYLGWTWGSLALTVAFVWQMYTTHLLVMM

HESVPGKRLNRFIEIAQEAFGPKRGLLIVIPPLIQLSAGYCVALTILGGSALQLFYSTVCTECFYTKVLTVMEWYLVFTC

LCWVVALLPNLNSISYVSLVGSIMAVAYCTLLWAISVDAGRPLPISYDAVRRKSDLATAFTIFVALGNVVFAFRGHNLIP

EIQATMPSSLKNPAHVPMWRGVRIAFVTVAICLFPIAIAGYWAYGDKMLPSGILFSLYHYHVMGASRVLIGMTFLMVALH

AVTTYQIYAMQQFDFFEMLQTMRSNRPVPWWLRLLFRSGFVFFNFFAAVALPFISSLAGFLGGISSIPISFAFPCFMWLW

IKKPSVRSFDWYLNWTLGIFGICLSLCVSVSGIWSIVDTGLRLNFFRPGET*

>SmAAP9A

MAVGMFPPSHDERISYAENGHKLGSLELQQQQKNVDDDGRPCRTGTVWTASAHVVTAVIGSGVLSLAWSMAQIGWIAGPV

VLLIFAAITFFTSLLLTDCYRSPDPVTGKRNYRYKDAVKANLGEIQLWCCALVQYSNLMGTAIGYTITASISMVAINRSD

CFHAKGHNGACNTSNNLYMALFGVVQLMLSQIPNFHKLWWLSIVAAVMSFSYSGIGLGLGISKIIENGHLLGSATGVPIG

LTLGSVTPAKKVWRVFQALGNIAFAYSFSTVLIEIQDTIKSPPAENKTMKKATLIGIITTTTFYLSVGCFGYGAFGNGAR

GNLLTGFGFYDPYWLVDFANACIVVHLVGAYQVFSQPLFEFVESTAANKWPKSGCIHTEHAIRIPFVGTWRVNVFRLLWR

TMYVIFTTIAAMLLPFFNDIVGLIGAAGFWPLTVYFPIEMFIKQKRIESWSWSWVALKTISAACLMISIAAGIGSIEGIL

HSLEKYTPFKTTY*

>SmAAP9C

MEKNWLACSIASKTYDFFFLFRCPSGTVWTASAHVITAVIGSGVLSLAWSMAQLGWAVGPPVLLAFAFVTYYTSILLADC

YRSPDPVTGKRNHTYQDAVAVTLGGAKVWICGIVQYTNLVGTAIGYTITASISMVAISRSDCFHRQGHDGPCYASDYPYM

VVFGAVQILLSQIPDFDRIWWLSIAAAIMSFAYSFIGLGLGMARTFEPGHSYGTATGVRIGMGGLSQTRKIWQVFQSLGN

VAFAYSFSMILIEIQDTLKSPPPENKTMKKATLVGVVTTTAFYMSVGCFGYAAFGNNAPGNLLTGFGFYEPFWLIDFANA

CIVIHLVGAYQVYCQPVFAYVEGHARSRWPKNKFVSHYFRIPIPLLGCYKFTLLTLVWRSAFVVVTTIVSMLLPFFNDVL

GLLGAISFWPLTVYFPIEMYIKQRSIVRWSPKWIGLKALDLGCLLVSVAATLGSVEGIALSLKEYAPFKS*

>SmAAP10

MTAGLDSSLPNGAEASIDMRFHGGAGGSEKQVERTGNVCTASAHVITAVIGSGVLSLAWSIAQFGWVPGPAILFIFSIVT

FYASLLLADCYRSPDPAFGRRNTTYIDAVKNILGGRQEWFCGLAQYGNLIGATIGYTITSGKSMVAISKGHCLRHNRHLS

NPSSCNIHDGRYLLVFGAAQLLFSQIPDIHQIWWLSIVASIMSFSYSFVGLGLSAGQAVHGTQGTAFGIGIGPGPHSVSS

ADKVWGILQALGNIAFAYSFSSILIEIQDTLKSPPSENVSMKRATSIGVLVTTIFYMAVGCVGYAAFGNDAPGNLLTGFA

HSKLFWLVDFANICIIIHLVGGYQVYAQPVFALGEWYASQKWPKSNLVNREYSVTVLTPRIGVFRFTIFKLFWRTLFVLF

TTIVSLVFPFFNAVIGLVGAITFWPLTVYFPVEMYSKQSGVRRWSCKAMALQSLSFVCFLVSLSAAVGSVQGIISSSRRY

KPFEF*

>SmAAP9B

MEAAGAPPPPAAIDKDDDGRPKRQGTVWSAAAHVITGVIGSGVLSLAWSFAQLGWIAGPIVLLIFAYLTYYTSALLADCY

RFPDPTTGKRNYRYKDAVKVTLGEVELWLCALAQYSNLAATAVGYTVTGALSMAAIARANCFHTKGSKALGCGVSVNLYV

TAFGLIQLVFSQIPNFHELWWLSYLATAMSFTYSTIVLVLGLAKLIGIPGGLVTTPAQKTWAVFQALGNVAFAYSFSMIL

IEIQDTLRSTPPENKTMKKATLVGVLATTAFYMSIACVNYAAFGDSAPGNLLSQGFEKPYWLIDFSNACIVLHLVGAYQV

YSQPLFDFVEAWALEKWPHSALNTTHKIKLLHWRYSTTLFRLVWRSLFVIATTVIAMAIPFFNDVLGLLGAMGFWPLTVY

FPIQMHIKQAQIKTWSMRWLKLQAISAFCLVISIAAGIGSIEGIYQDLKAYTPFHANF*

>270979

MAPHENDSTNNGSSPPSSTQAFKEYVEDKGHARTVKSVDDWLPVGSGSRNAKWWYSAFHNVTAMVGAGVLSLPSAMVYLG

WGPGVLVLVLSWVITLYTLWQMVEMHEMVPGKRFDRYHELGQEAFGEKLGLWIVVPQQLIVEVGVDIVYMVTGGTSLMRF

YELVHCKPDDISCKHIKRTYWILVFASVHFFLSQLPNFNSITGVSLAAAVMSLSYSTIAWVAPVHYGQEAKPPMTKVSYA

YPHSPSVANTVFRVFNALGQVAFAYAGHNVVLEIQATIPSSPQKPSKVPMWRGVVVAYIVVAMCYFPVSLVGYWAFGNDT

SYDNVLQRLGRPEWLIAAANLMVVVHVIGSYQIYAMPVFDMLETVLVKKFHFPPGVILRLVARSLYVAFTAFIGITFPFF

GDLLGFFGGFAFAPTTYFLPCIMWLAVYKPRVFSLSWMANWICIVLGVLLMIVATIGGFRNIIMDASTYKFYQ*

>127260

VDEWLPVTSSRNAKWWYSAFHNVTAMVGSGVLALPSAMVYLGWGPGIFVLLLSWTVTLYTLWQMVEMHEMVEGKRFDRYH

ELAQEAFGERLGLWIVVPQQLIVEVGVDIVYMVTGGKSLKRFYELVSCAPDATGCKHIRQSYWILVFASIHFVLAQLPNF

NSISGISLSAAVMSLSYSTIAWTTAIPNAGGPDVSYSYPHSPSAANTVFKVFNALGMIAFAYAGHNVVLEIQATIPSSPS

KPSKGPMWKGVVVAYMVVAICYFPVALIGYWAFGNDTSYDNILQHIGTPHWLIAAANLMLVVHVIGSYQIYAMPVFDMLE

TLLVKKLHLPPGVCLRLIARTVYVAFTAFVAITIPFFGNLLGFFGGFALAPTTYFLPCIIWLAVYKPKRFSFSWLANWIS

IVLGVLLMIAATIGGFRNLVMDASTYKFYQ*

>75458

WLSINDSWTSKWWYAAVHNITAVIGAGVLSLHAAMVDLSWAPGIFVLCVIGVISLSTMWQMIELHELDGKRMDRYHELGQ

RAFGKKLGLWIVVPMQMLVEIGVDTVYLLTAGKSIRKIHSLLYGCPIQDSSCNWELRYCIMAFASVQLLLSQLPHFTSIT

WVSIIAAFMSLGYSTIAWVATLMRERSPTVSYEFPKATSTADVIFRVFSSLGQISFAFAGHNIVLEIQATIPSTIERPSK

ISAWNGALLAYTMTILCYFPNALVGYYVFGNQKNHDMHVLEILDKPVWLVALGNAMVVTHMCGGFQIFAMPLFDNVEMLL

TNLWKVNGGINLRLLVRSIYVAFTCFLAVTFPFFDDLLAFVGGIAFVPTTFLLPCIIWQILRKPRTLGLPWLANMACIGV

GFFLTIASTAGGLRNILLKASHYQFYK*

>156907

MGEEAALAKEKLDAGAAFVLVSKGTWLHAAYHLTTAIVGPAILSLPYAFASLGWELGVLALTMGALVTFYGYNLVSTLLE

QADQRGQRHLRLGDLAVDILGPKWSKYVVFPQMVISFGIVVGSNLLCGQGMLKIYENLVKDGDLKLYHFVMISASIMIIL

SQLPSFHSLRYISLASALLSMGYSLGVVAACIYAGHSKRAPPKDYSIVGSTSARVFHAFNGLSIMASTYGVSIIPEIQAT

IASPVSGKMFKGLLLCYAVVVTTFFSVSISGYWAFGNKATGNLFDNFIPDDNTTLAPDWLLFLIILFIVIQLLAIAVVYS

QPLFDVFETALSDVKRPIFSFRNLLPRLAVRSLYIVLAAFLAAMLPFFGDLNAFIGAVGFLPLAFILPPVLYNIKCKPSP

GTVVFWVNTAIIVVYGAMAVMGSVSSVRQIVLDAHKFKVFSNNTS*

>127270

MSNAASYFVFWNCLRRILRPEHNAKWWYSTVHIVTAMVGAGVLSLPSTMVYLGWAPGMMMLGVSWIITLATMYQMIEMHE

DESGRHDTYQCLGRKAFGDRLGNLIVGSQQIVVQVTANIAYLVTGGQALKRFGDLVLNREIQYGKFELAVAWISAFAGVQ

AVLSLFASFSSMTIVSLMASIMSFSYSTIVWATAIRLKSSQASYGYCNLTYYRAFNALGEIAFAYGGHNVALEIQATMRS

TRHKPSKLPMWNGVLVAYVMVAVCYFPVAGVGYWALGNLTCYENVLDVLDKPKWLIGTANLMLMLHLTGSYQVFALPIYD

ALTCWLEQKKLPINAWIRPLYVGFTCLVAVIIPSFAGLLGLFGGLALGPTTYFLPCIMWLSIKKPRVLGLEWLLNWACIL

FGVVLTIVSAIGSIVNLKHGFEEQNLKVFYFPRLQQCNSTSTNASCPVRH*

>173454

MFFEIRIFSIQGFLLTKWRSWILLNDSWSSKWWYSTVHIVTVTVGAGVLSLPTVMAYFGWALGTMLLVGFLILSLMCYWQ

LIEMHETEHGRRFDRYHELGQHILGRHLGFWLIAPLQAIAQVGIDTVYIIAGANSLEHVYSLFDKCKELDVHKCKGINLT

YWMILFMGVQLLLSQLPHFQSITWVSFIAAVTAIGYCTLAWVGILIKQPALSSGSAASAPTQCFQNVGHGYPHGSKAHLA

FGIFTSLGKLAFAVAAGHNIALEIQATIPSTSRHPSKRAMWRGILVAYLVVAFCYLPVALVGYKVYGDETRDLCSGLDNV

LLRLRNPKPMIVLADLMVFIHLCGSYQVLAMPLFSNFETLVERMFKFEANLKHRMIMRSCYVVLTLMLAAAFPFFGDLEA

FFGGFALIPTTYVIPSVLWHLSRKPEPFSPPWIANLLCISFGIAVMATSTIGGLRNLIMKRRELEFFQ*

>98385

MSNAASYSVFWNSLRGILQPEHHAKWWHSTVHIATAMVGAGVLSLPLNLCVHRAPGMMMQGVSWIITLATMYQLIEMHED

EYDTYRDLGRKAFGDRLGFIVGLQQIVVQVTANIAYLVTGGQALKRFGDLVLSREIQYGKFELAVAWISAFAGVQAVLSL

FASFSSTTIVSLVAAIMSFSYSTIIWATAIRLKSSQVSYLYCNWRYYRASNALGEIAFAYGGQNIALKIQAMMRSTRHKP

SKLPMWNGVLVAYVMVAVCYFPVAGVGYWALGNLTCYENVLDIFLDKPKWLIGTANLMLMLHLTGSYQVFALPIYDGLTC

WLEQKKLPINAWIRPLYVSKGALPGFTCLVAVIIPSFIGHLGLFGGLALGPTTYQLPCIMWLSIKKPRILGLEWLLNWAC

IFFGVVLTIVSRIGSIVNLKHGFEEENLKVFYFLRLQQCNSTSTNASCPVRH*

>413158

MSNAASYSVFWNCLRRILRPEHHAKWWYSTVHIVTAMVGAGVLSLPSTMVYLGWAPGMMMLGVSWIITLATMYQMIEMHE

DESGRHDTYQCLGRKAFGDRLGNLIVGSQQIVGQFLVHDNRLSDSLHHVFQENVIHISLSYSTIVWATAIRLKSSQASYG

YCNLTYYKAFNALGEIAFAYGGHSIALEIQATMRSTRHKPSKLPMWNGVLVAYVMVAVCYFPVAGVGYWALGNLTCYENV

LDVLDKPKWLIGTANLMLMLHLTGSYQVFALPIYEGLEQKNMPINALIRPLYVGFTCLVAVILPSFSGLLGLFGGLALGP

TTYFQLPCIMWLSIKKPRVLGLEWLLNWACILFGVVLTIVSAIGSIVNLKHGFEEQNLKVFYFPRLQQCSSTSTNASCPV

RH*

>99162

MLTPQGNGTYTPTPSSTRPPSNLGSPARQQPNPSSRLLRSPKVLFSPIGTPMRKALTNMRAYLEDIGHITKLNPQEAWLP

ITASRNGNAYYSAFHNLNASIGFQALLLPVALTFLGWTWGVLALVAAFIWQLYTLWILIQLHEAVPGKRHSRYVELAQEA

FGPKLGAWLAIFPVVNLSGGTATGLIIIGGGTLELFYRTVCRDCHGGSLTTVEWYLVFTILCAILAQLPNLNSIAGVSLV

GAVMAVAYTTLVWTLSISRPRPPGITYDIVKPDHTAGNIFSVLNALGIIAFAFRGHNLVLEIQGTMPSSLKHPAKSPMWR

GAKVAFAIVAACYFPIAIAGYWAYGRMMLPSGILFSMYALHPDIPSPWMAITFLFVVLNSISSFQIYSMPMFDAFEQSFT

ARKNKPTPLLARVAFRLFFTFFAFFVGVALPFISSFAGLLGGLTSVPVTFCYPCFMWLKIKKPPRFSFTWYLNWTLGILG

IVFSITFTAGGIWSIVDSGLTLNFFNP*

>98878

MEREPEVSSLPSTPQNNHSIPPSVARSPRRMMLSPMGTPMRKAFGNMKCYLEEIGHIAKLNPQDAWLPITESRNGNAYYS

AFHNLNAGIGFQCLLLPVAFSFLGWFWGVLALVVAFLWQLYTLWILIKLHEVIPGRRYNRYIELAQAAFGERLGSWLTSF

PIISLSAGTAGGLIAIGGSTLHLFYNLVCIKCHGQSLTAIEWYLVFAVLCAIIAQLPNLNSVAGVSLIGAVMAVAYSTMI

WILSVTRDRPPGVSYDVAKPYSSVGAAFSFLNALGVIAFAFRGHNLALEIQATMPSTLKHPAYVPMWRGSKAAYTLVAIC

YFPLAIGGYWAYGKLMLPTGILTSMFVFHRSDISPAWLATCFLFVVVSSLSNFQIYSMPTFDLVEQTYTANTNKPCPKLH

RFVFRLLFVFFGFFVGIAFPFMASFGGLLGGVCSVPVTFCYPCFMWLKIKKPPKLSFSWYLNWTLGILSVVFTIVVTIGG

IWSIVDTGLKFQFFKPQ*

>426884

MRASLSARDQQVGGTESHEREDRMSNAASYSVFRNCQRRILRPEHSGGTQPFTSRRLWAPGMMMLGVSWIITLATMYQLI

EMHEDEKAFGDRLGFIVGLQQIVVQVAANIAYLVTGGQALKRFGDLVLSREIQYGKFELAVAWISAFAGVQAVLSLFASL

SSMTIVSLVAAIMSFSYSTIIWAIAIRLKSSQVSYGYCNWRYYRASNALGEIAFAYGGHNVALEIQATMRSTRHKPSKLP

MWNGVLVAYVMVAVCYFPVAGVGYWALGNLTCYENVLDVLDKPKWLIGTANLMLMLHLTGSYQDLRVGCGDHTFVWRASG

IIRWARSWTNNQSPCIMWLSIKKPRVLGLEWLLNWVKISSFLDRKLLTWWYEPMQACILFGVVLTIVSRIGSIVNLKHGF

EEENLKVFYFPRLQQCNSTSTNAICPVRH*

>pa__MA_101691g0010

MTMTQIHHPTLEVSIESSGLQSGKVSDSKKFDDDGCIKRRGTLGTTSAHIITAVIGSGVLSLAWATAQLGWIGGPAAMLAFTFVTYYTSCLLSDCYRSPDAVTGKRNYTYSLAVRANLAASISMTAIQKSNCFSTHGDDYPCHVSSNPYMIVFGVVEILLSQIPDFEEIWWLSIVAAVMSFTYSTIGLGLGIAKVAEVGHFRGSLTGATSGTVTKADKIWNAFQALGNIAFAYSYSLVLIEIQNTIKSPPAENMTMKKATLLGVATTTVFYMLCGCMGYAAFGENAPGNLLTGFGFYNPFWLIDIANAAIVIHLIGAYQVNLFRLVWRTCFVVSTTLVSMLLPFFNNVVGLLGAVAFWPLTVYFPVSMYIARNKIRRWSSRWVAMQILSGFCFVISVAAASGSVVGIAEALKAYKPFKTA

>pa__MA_14300g0010

MAEVREPLCPNEAAMDEKYGQHHPHSYCNFPNEDEQVQRTGSLWTAVAHIITSVIGAGVLSLSWSIAQLGWIAGPATMIVFALITLYSASLIVDCYRFPDPITGPIRNGSYRDAVRVNLGERRARLCGLVQYVYFYGICVAYTITASMSIRAIRQSNCYHRNGHESPCQFSEQTYIILYGVIQVFLSQLPNFHNLWGLSIVAAMMSFSYATIGFGLGVAKVIENGEIYGNLGGISWSTSITSAQKVWRILQALGDIAFAFPYSSLVLEIQDTLKSPPPENGTMQKASLFSIMVTTSFYMLCGFLGYAAFGENAPGNLLTGFGFYEPYWLIDFANACIVVHLVGAYQVFCQPMFAFIEGWASHKWPNNKFINEECSIRIPLFGLYNVNLLRLCWRTAFVVSTTVIAILFPLFNDVLAILGALNFWPLIVYFPVEMYISQNKVRPLALKWTLLQAFSFISFLLSVGAALGAIEGLVNDTDT

>pa__MA_165784g0010

MEELTDITAMNVEAGRHDSEKQHDISSDDGRVRTGDVRTATAHVITAVIGAGVLSLPWSVAQLGWLLGTPILLAFSWVTYYSAILLTDCYRSPHPVTGARNYKYRDAVKAILGGYKVSLCMWAQYSNLYGCLIGYTITAATSMMAIKKAGCFHERGKNAPCRISGNLYMIIFGVLELILSQLPSLEEISWLSIVAAIMSFAYSSIGLGLSIAKVAGRKHLSGSLMGVPIVELSPAKKTWYMFQALGNMAFSYSFSTVLIEIQDTLKSPPPENKTMKKATKMGLSVTTLFYMSIGLVGYAAFGNDAPGNMLTGFGFYEPFWLIDIGNLCIVIHIVGAYQVFSQPVFAVIEDWVRGRWKMSGFVHRVYTIKFPFRGSVRFTIFRLLLRSSLVVSTSLIAMLLPFFNAIMGLIGAMSFWPLTVYFPVEMYIVQTSVKKWSRKWIFLQFLSLVCLLVTLVAAIGSVAGITMALKHATVFNMKY

>pa__MA_402129g0010

MRRDSVIEYQISAMESGSIEGHGGHRTLVVGKEIMFSPLITDEDGHPQRTGDVWTASSHVITAVIGSGVLSLAWSMSQLGWIAGPLVLLAFSFVTYYTSMLLADTYRSPDPVTGRRNYTYTDAVTAILGGKRVFLCGIVQYLNLVGTTIGYTITASISMVAIGRSDCFHEKGRESPCHISNNLYMAIFGAAQVLLSQIPNFSKIWWLSTLAAVMSLTYSFIGLGLGIGMATEKGHSHGSLGGVGIAGVQKSVDKIWNIFQALGNIAFAYSFSMILIEIQDTVKSPPAENKTMKKASFIGVVVTTMFYISVGCAGYAAFGDHAPGNLLTGFGFYNPFWLVDIANICIVIHLVGAYQVFCQPLYAFVEEWSANTWTKSCFIQNEYKVPIPGLGEFKLNLFRLVWRTCFVVFTTVVSMVLPFFNAIMGVLGAIAFFPLTVYFPIQMHIAQTKLRQWSFKWVALQLMCVLCFFVTMAALVGSIAGVVEVLQHYTPFKTTY

>pa__MA_43770g0010

MAQNDAAPRCYLQVELQGNMEEICTEADHLENGKVSDFKNIDDDGRMKRQGTLWTASAHIITAVIGSGVLSLAWATAQLGWIAGPTAMLVFSFVTYYTSCLLADCYRSPDPVTGKRNYTYIHAVKANLDGFQVWICGFTQYVNLFGTAIGYTIAASISMTAIERSNCFNTHGHEDRCHASNNPYMIVFGIVEILLSQIPDFDQISWLSIVAAVMSFTYSTIGLGLGIAKVVEVGHFRGSVTGATIGTVTKAEKIWDTFQALGNIAFAYSYSLILIEIQDTIKSPPAENKTMKKATLVGVATTTVFYMLCGCMGYAAFGHNAPGNLLTGFGFYNPFWLVDIANAAIVIHLVGAYQVFCQPLFAFIEGWSGKKWQYSDFINKEYALAIPLYGPYKVNLFRLVWRTGFVVSTTLISMLLPFFNNVVGLLGAVAFWPLTVYFPVTMYIAQNKIRRWSSRWVAMQILSAVCLLVSIAAASGSIVGIVEALKTYKPFQTTY

>pa__MA_6860g0010

MEAHSSEVLSRTGTLWTAVAHIITGVIGAGVLSLAWSVAQLGWIAGPVALVVFALVTLYSTFLVSDCYRFPDPVSGPKRNSCYRDAVRVNLGTRRAWLCGLVQYASFYGVCVAYTITASISVRAIRRSNCYHKYGHDASCHFPDLTYMILFGVIQVILCQIPNFHKLWGLSILAATMSFSYATLGFGLGMAKVIENGEIKGNLSGISASTSLTQAQKVWRMLQGLGDIAFAFPYTSLVLEIQDTLKSTPPENRTMKKANLLSLSITTTFYMLCAFLGYAAFGENAPGNLLTGFGFYEPYWLVDFANACIAVHLVAAYQVYCQPIFAFVEGWFSHIWPDNKFINKGIPMRIPFCGLCRVNLLRLCWGTAFVVSTTGIAIVFPLFNDVLGILGALNFWPLVVYFPVEMYIARNKVPRWTLKWNIFQIFSFISLLITVITATGSLEGLVKDKET

>pa__MA_74043g0010

MDCRFDEVESGFNASRRDGNNISNDVGRRGTLVGSQSHSNDDDGREKRKGNVWTASCHVITAVIGSGVLSLAWSMAQLGWVVGPIVLLGFSVVTYYTSILLADCYRSPDPVTGQRNYTYKDCVKAILGGRRVFLCGFIQYLNLLGTSIGYTITASISMVAIGRSNCFHEKGRDSPCHISNNLYMAIFGLAQIVFSQIPNLHKMWWLSVVAAVMSVSYAGIGLGLGIGKATEKNHSYGSLKGAGIGELFEDIDKTWHVFQALGNIAFAYSFSMILIEIQDTIKSPPAENKTMKRSTTIGVAVTTLFYMSVGCVGYAAFGTDAPGNLLTGFGFYNPYWLVDIGNVCIIIHLVGAYQVFAQPLYAFFEEWCSNTWTKSSFIHKEYTVKFPRCGSLNLTLFRLVWRTCFVICTTLVSMILPFFNDIVGILGALAFFPLTVYFPVEMYIVQYKVPRWSCKWMALRLMSLICFVISLVALVGSVAGVINDLRSYKPFKTKY

>pa__MA_889393g0010

MDDKKKHHYSHSNACELHDAVEICRTGTLWTAVAHIITSVIGAGVLSLSWGVAQLGWIAGPVVMIVFALITLYSTFLLVDCYRFPDPVSGPMRNTSYRDAVRVNLGERHARLCALVQYALLYGICVAYTITTSVSIRAISRSNCYHRNGHDSPCHFPDLTGFGLGLAKVIENGEIYGTLGGISTAVSLTRAQKVWRILPALGDIAFAFPFSPLVLEIQDTLKSPPAESRTMKKATLIAIMITTSFYMLCGFLGYAAFGENAPGNLLTGFGFYEPYWLIDFANACIVVHLVGAYQMFCQPIFTFIEGWISHKWPNNMLISKRLGVNVPLFGFCNVNLFKLCWRTAFVVSTTGIAILFPLFNDVVGILGALNFWPLVVYFPVQMYIVQKRVQLWTLRWNLLQTLSFISLLVSVGTAVGSIEGLVKDNET

>pa__MA_902657g0010

MDDNEKHHRSHPLACELDDTVEISRTGTVWTAVAHLITSVIGAGVLSLSWSVAQLGWIAGPAAMIVFALVSLYTTFLLVDCYRFPDPVSGPMRNTSYRKTVRVNLGERKAWLCALVQNAFFYGVCVAYTITTSVSIRAISRSNCYHKNGHDSPCHFRNITYMIIYGVIQVILSQIPSFHKIWGLSILAAIMSFTYSTLGFGLGLAKVIENGKIYGTLGGISTTVSLTRAQKFWRILPALGDIAFAFPFTPLVIEIQDTLKSPPPENKAMRKASLVSMMITASFYMLCGFLGYAAFGENAPGNLLTGFGFYEPYWLIDFANACLAVHLVAAYQVFCQPIFSLVEGWISRKWPSNTLISKRISIRVPLFGFYKVNLLTLCWRTAFVVSTTGIAILFPLFNDVLGVLGALSFWPLVVYFPVEMYIVQKKVQRWTLKWSLLQTLSFIALLISLVTAAGSIEGLVKDKKS

>scaffold00017.258

MGENGVRKQYLQVESQTRVYGDMAIDMQHQGSKCYDDDGRLKRTGTVWTASAHIITAVIGSGVLSLAFAIGQLGWVAGPT

VMVLFSFVTYYTSTLLSDCYRSGDPLTGKRNYTYVDAVRANMSEFNVRLCGWLQYAGLVGVAIGYTIASSISMMAIKRSN

CFHETRGKNPCHVSSNPYMIMFGITEIVLSQIPDFDQIWWLSFLAAVMSFTYSSIGLGMGIGKVAVNGTIKGSMTGISIG

AMTHAGPITAMQKVWRTFQAMGDIAFAYSYSIVLIEIQDSLKSPPSEAKTMRKATMISVTVTTIFYMLCGCMGYAAFGDQ

APGNLLTGFGFYNPYWLLDIANAAIVVHLVGAYQVYCQPLFAFVEKYAVRKWPKSWFINHEFELRVPFYSKSFSLNLFRL

VWRTSFVCMTTLIAMLFPFFNGVVGILGAFGFWPLTVYFPVEMYISHMHIPKWSTKWVCLRALSLACLVISMLAATGSVA

GMIFEMKAAYRPFHTNY*

>scaffold00033.54

MQKEEGVQCLDAGTPFDQTDDDGRVKRTGTLLTASAHIVTAVIGSGVLSLSWAIAQLGWIFGVFVLLIFSLIILYTSFFL

ADCYRYPDPVNGKRNYNYKAAVSAHLGGLKTKICASTQYVFLVGNCLGYAVTASLSMAAVERLNCFHKNGHAAECVASTN

KYLVIYGCIQIVLSQIPNFHKLWGLSIVAAIMSFCYSSIGVGLSIAKIAGEGASKTTCLTGVRVGIDITAGEKFWRVCQA

LGNIAFASAFTAVLLEIQDTLKGPPPAENKVMKKATTISTIITTVFYLLCGCLGYAAFGSKAPGNMLTGFGYYEPFWLID

FANVCVAVHLFGSFQVFAQPLYAVMERVCSRRWPSNPFISNEWTISTLGLCSYSFNFFRITSRTLYVVVLTLIAMIFPFF

NDLVGLIGSIAFWPLTVYFPVEMYIHRKKVQRASTEWCWLQILNLICLLVSVAAAVGSFQGLATSLRTYKPFKTF*

>scaffold00033.53

MHEHDNGIAFQGTAERRSLEIGKASIGFDDDGVEKRTGTFWTASAHIITAVIGSGVLSLAWGMAQLGWIAGAFVLLLFSG

ITYYTSCLLADCYRSPDPVHGKRNYTYTDAVKANLDERRTKLCGIAQYIYLFATCLGYTLTAAISMAAIERSNCFHKHGH

DAHCPPKTNRFLIIFGCIQLFLSQIPNLHKLSWLSVIAAVMSFTYSTIGVGLSIARVSSKGASHNTSLTGVAVGVDVTST

EKIWKVCQALGDVAFAYAFTSILIEIQNTLKSSPPENKVMKKASFLGILVTTMYYMLCGFIGYAAFGNKAPGNFLTGFGF

YEPFWLIDLANVCIAIHLFGSYQVFGQPMFAYLERGCSRRWPSSSFVNNELRTKILGFPFQFNMLRLVARSLTVIIMTII

TMIFPFFNDVVGLAGSMSFWPLAVYFPTEMYIRKRNLNKGSSEWWWLRLLSLLCLFVSIAAAVGSVQGLITSLRSYKLFK

FE*

>scaffold00071.167

MEMEMDEVMAGRAFEDEEDDHKREGTVWTATAHVITAVIGSGVLALGWSVAQLGWMMGPLTVVAFAWVTYYTANLLSDCH

RSPHPTTGHRNHTYIDAVRACLGPQKVLICGVAQYSNLVGTLIGYTITSAISMMAIKRSDCFHENGHNSRCGVSGNLYVA

LFGVLEVILSQLPSLEKISWLSILAAIMSFAYSFIGLGLSIAMVICHGDIRGTLFGVKVGINDIPLTARTWNAFQALGNI

AFAYTYALVLIEIQDTLKSSPPENKAMKRASLYGIGITAIFYFSIGSAGYAAFGNDAPGNMLTGFGFYEPFWLIDIANLF

IVIHLVGAYQVFSQPVFAAWEMYLSSKWSQNSIVHSTYNVKLPLVPSTSFNFTLSKLISRTLLVIIVTLVAMTFPFFNAV

NGLIGAIAFWPLQVYFPTTMYISQMKVKKGTQKWIFLKTLSVCCFFVSLIAVVGSVAGIVDSLKHATPFQTMY*

>scaffold00071.161

MIPTYNAYSYGTVWTAAAHVITAIIGTGVLGLAWSVSQLGWIVGPLFLVGFGFVTYYTSTLQADCYRYPDPVTGKRNYTY

REAVRAFLGSRSLVMCSIAQCAILWGIMIGYTIIAATSMIAVKKSNCFHKNGHNASCKTSENVFMVTYGLFQIVLSQLPS

MHKLAVTSVVAAIMSFGYSGIGLSLSAAKLVSNGVIKGSIGGNSLGNRESSLASNVWNSFQALGNIAFAYVFAHVLTEIQ

DTLRSHPPENKVMKRATLYGIVVTSVFYMSLGCVGYAAFGSKTPGNILTGFGFYEPFWLVDIGNICVVVHLLGAYQVFAQ

PIFAAIEDRVSSKWPSNFLLQARYEVKLPCSTQTSWRVTLFSLIMRTTIVVMTTLVAILVPLFNSVVGLLGAMAFWPLTI

YFPVSMHIVQANVTRGSMKWFLLQCLVGASLIVSVIAGIGSLVDITKSLKHSKPFQAKY*

>scaffold00061.89

MEMTDKQEPLIADAQVRCKRTGDAWTATAHIITAVVGSGVLSLAWCVAQLGWIAGPLAILGFAVITLISSYLLADCCRSP

NSEKGHIRHITYIDAIDFNLGKKSVWICGLVQQLGICGTAIAYTITSAMCLRAIQRSNCFHIEGHEAACKYGDTAYMLTF

GVVQLVCSQIPDFHNMGWLSIVAAAMSFSYSSIGFGLGLAKTIENGKIYGNITGVAMSSTVAKLSRVCQALGDIAFAYPY

SIVLIEIQDTLKAPPPENHTMKRASMTAIVITTFFYLCCGCFGYASFGDSTPGNLLTGFGFYEPYWLIDFANACIVLHLV

GGYQVYSQPLFAFVEKWLIQTFPTCALMKTTYTLHIPFIPTFQLNLLRLCFRTAYVVSTTGLAMFFPYFNQVLGVLGALN

FWPIAVYFPIQMYIVQKNVQTWTSKWLIFQTFSCICLVASVIALTGSIQGIVSEKTS*

>scaffold00071.165

MKEDEGRKMEEGGNMDDGRVRNGTVWTATAHIITAVIGSGVLGLGWSVSQLGWILGPFCLLGFGYVTYYTATLQADCYRY

PNPLTGKRNYTYRDAVRVFLGPRNVFMCGMVQYAILWGSMVSYTIVAASSMVAMKKSNCFHKSGHNSKCGTSGIMYMFIY

GLFQVILSQLPNLEKVSTISVVATITSFVYSGIGLALCIAKFVCQGEIKGSLRGNSIGDTASSSISSNMWNAFQALGSIA

FAYAFAHVLIEIQDTLRPHQPENKTMKRAAVYGMASTAIFYVSLGSAAYAAFGSHTPGNILTGFGFYEPFWLVDIGNLCI

AIHLTGAYQVYGQPIFAAIEDMIYSTWPTNCFVHLQLTVKLPFSNLGGVSLSPLSLLVRTTVVVVTTLVAMLVPFFNSVA

GLIGAIAFWPLTVYFPVNMYIAQARLKRGTMKWVLLQCLLAASFVVSLLAAIGSVADIIKSLKHSKPFKAVY*

>scaffold00005.76

MGGAFDGQENDVSLEKQKAVDDWLPITSSRNAKWWYSAFHNVTAMVGAGVLSLPFAMAELGWGPGVAILVISWIITLYTL

WQMVEMHEMVPGKRFDRYHELGQHAFGEKLGLWIVVPQQLIVEVGVDIVYMVTGGKSLKKFHDTVCPGCKPIKLTYFILI

FSSVHFVLAQLPNFNSISGVSLAAAVMSLSYSTIAWVGSLDKGKQPDVDYSYRASSTSSAVFNFFSALGEVAFAYAGHNV

VLEIQATIPSSPEKPSKKPMWKGCVVAYIVVALCYFPVSLIGYWAFGNKVEENILLSLNKPRWLIAAANMFVVVHVIGSY

QIYAMPVFDMLETVLVKKLKYTPSIVLRLITRSSYVAFTCFVAISLPFFGGLLGFFGGFAFAPTTYFLPCVMWLAVYKPK

RFSFSWIANWFCIILGVLLMIVAPIGALRQIILNAKGYNFYS*

>scaffold00040.261

MTTNEEKVKDLDAGALFVLKSEGSWIHCGYHLTTSIVAPALLSLPFALASLGWVLGLLCLTIGALVTFYSYNLLSLVLEH

HAQQGQRHLRFRDMAHHILGPRWARYFVGPIQFAVCYGAVVGCTLLGGQSMKSIYLINHPNGGMKLFEFVMIFGCLMLVL

AQVPSFHALRHINLVSLILALGYSACATAGSIHVGNSSDAHKDYSLNGDTASRMFAGFNAMAIIATTYGNGIIPEIQATL

APPVKGKMFKGLLVCYSVVFVTFFSVAISGYWAFGNKAGGTILANFFVDGKALVPKWLLVLSNLFALLQVSAVGVVYLQP

TNEVLERKFGDAMSAEFSPRNVIPRLILRSISVALATLLAAMLPFFGDINALIGAFGFIPLDFVLPVVFYNTTFAPSKKS

FLFWGNTTIALVFSAVGLIGAVSAVRQIYLDADNYRLFANV*

>scaffold00002.493

MESGEQEEGWIENRRSKGTWKHAAFHVATTIATPAAYAPLPFALASLGWSLGVFSLVGGTLVTWYSSLIISSLWAWNGEE

HTTYRQLSRSIFGSWGYWAVSFFQQVASLGNNIAIHIAAGTSLKAVYKYFHENGALTLQEFIIFFGVFELFLSQFPDIHS

LRWVNALCTCSTVGFAGTTIGLTLYNGKKIERGSVNYHLEGDLATKIFKAFNALGTIAFSFGDAMLPEIQSTIRNPAKKN

MYKGISLAYGIIVLSYWQVAFSGYWAFGSQVQPYILSSLKVPQWAIVMAHMFAVIQIAGCFQIYCRPTYAYFEENMLSKD

QTGLFRIRNRLVRLFATSLYMVLITCIAAAMPFFGDFVAICGAVGFTPLDFVMPLIAYVKVGKLPKNRSLGLSVKALNLF

IAVWFSGVAILGCIGAVRFTVKDAQTYKFFYDM*

>scaffold00015.85

MEEAQENGRHQFPQNPCENDAGAVFVLESKGKWWHAGFHLVTAIVGPTILTLPYAMSGMGWSLGLLSLTAVFAVTFYSYS

LMSKVLEHCEKEGRRHIRFRELASRILGSGWMFYFVICIQTAINTGVGVGCILLAGQCLQIMYKDVYPDGPLKLYHFIMM

ATVVMVLLSQLPSFHSLRHLNLASLFLSLGYTLLLVISSIVAGYSKNAPPKDYSLETSTSVRTFDSFSSISILASVFGNG

ILPEIQATLAPPVTGKMLKALSLCYIVIFFTFYAAAISGYWAFGNKVNSNILKSLMPDNGPSYVPTWMLGIAIIFVILQL

FAIGMVYSQVAYEIMEKKSADIRQGVFSKRNLVPRIFLRTSYMIFCAFLASMLPFFGDISAVVGAVGFIPLDFILPMLLY

NMSLKPPQKSFTYIINISIIIVFSGVGIMGAFSSIRKLVLDARKFKLFSDDVVG*

>scaffold00001.289

MAELEDVSAPITPRSTTGATTTTPPTPAPRSPWNVPITPRSMAEPPTPGGLRSPIPHSTMMIREVVSVPVTPRSTASVTP

PVPHSPLPPLPRRPPPVISAPPSNLHSPSLTRSPLLHTPTTTGNTPRTRFSTPFASPMRRAIVNMRSYLEDVGHLTKLDP

MDAWLPITESRSGNAYYAAFHNLTAGIGFQALVLPVAFTFLGWTWGIICLSIAYFWQLYTLWILTRLHESVPGTRYSRYI

QLAKAAFGKKLGAWLCKIPIMYLSAGTCTALTIVGGSSMKLFFEIVCGSSCHSDPLTAVEWYLVFTCLAVVLSQLPNLNS

IAGVSLVGAITAVAYCTLIWVLSVVRPRPPGVSYDPIRGKSDPATAFSILNALGIIAFAFRGHNLVLEIQATMPSTLKHP

AHVPMWRGVKVGYVLVAACLFPLAIGGFWSYGHLIPAGGMVSALYGFHMKDIPRGLLGLTSLLVVINCLSSFQIYAMPVF

DDFEHDITKKSNRPCPRWLRSGFRAFFGFIAFFIGVAFPFLSSLAGLLGGVSLPITLAYPCFMWIFIKKPEKYSANWNIN

MGLGCLGIIMSTLVVIGGLWSTIDSGLKLRFFKPE*

>scaffold00166.19

MEDQENGLQKERSPKALSIEPGLSTAHTIDQDPWIQVGLLLVTSFSCGYMLGYSNLILKPLGWTWGLIAMLIIGFLALYA

NWLLAGFHVIDGQRFIRYRDMMGYLFGRRMYHITWSLQFLMFLLGNMGFILLAGRSLKAIHAEFSLSTLRLQIYIIITGA

IYFLFALMIPNMSAMRHWLGVSSVLTITYVVIVMAICVKDGKSSVSKSYAIEGSGTEKFFNAFNAFSAILFADTSGMLPE

IQATLRKPVVKNMRKALCMQFTLGLAIYYAVTILGYWAYGSNVSEYLPNNFSGPKWAVIVANATVFLQTIVSQHMFCTPV

HEALDTRFLHLDQSMHSRDNIKILFVLRAGLFTLNTFMAALIPFLGDFVNLIGSLSLFPLTFVFLSMIFLKVRGKTANSV

VKAWHWINIVGFSVITILTTVAALRLIVENAKAYRIFADN*

>scaffold00029.259

MVRPFDSNILLTNTKTLRIDSLREDRVDIASCESSSLLADSMAGLEESPKKPIGDEFDLKVQEETAHQISHDPWYQVAFV

LTTGVNSAYVLGYSGTIMVYLGWFAGTIGLIAAAAISLYANSLLAKLHEVGGKRHIRYRDLAGHIYGRKMYSLTWALQYV

NLFMINTGYIILAGQALKAIYVLFRDDHALKLPYCIIIAGVVCAMFAFATPHLSALRVWLGFSTFFGLVYIVIGFVLSLK

DGIRSPSRDYSIPGTSTSKVFTSIGATASLVFAYNTGMLPEIQATVKPPVVKNMQKALAFQFTAGVLPLYAVTFVGYWAY

GSSTSSYLLNSVTGPVWVKMVANVAAFLQTVIALHIFASPMYEYLDTTYGMSRQGKTYSMQSILFRVVVRGGYLTVSAFV

AALLPFLGDFMSLTGALSTFPLTFVLANHMYLMVKREKLSSLQKAWHWLNVCGFSLLAAAAAVAAVRLIVVDSRTYHVFA

DI*

>scaffold00059.251

MEERSSEPELVSIPATPKVSTPPLTPDILSSSHSHAVTPSGQRSPRPLSSSLISLSNPASPPPPLKTPRTPWTPSSLISP

RFLSPIGTPMKRVLVNMKAYLEDIGHLTKLNPQDAWLPITESRNGNAYYAAFHNLNAGIGFQALILPVAFTFLGWSWGII

SLVIAYCWQLYTLWVLVRLHEAVPGKRYNRYVELAQAAFGEKLGVWLALFPTVYLSAGTATALILIGGETMKLFFQIVCG

PNCESNPLSTIEWYLVFTTLCIVLSQLPNLNSIAGLSLVGAITAITYSTMSWVLSVSQPRPPSISYQPLRSPSFSVTALS

LLNSLGIIAFTFRGHNLALEIQATMPSTLKHPAHTPMWKGAKVAYLLIAMCLFPIAIGGFWAYGNSMPAGGMLNALYGFH

SHDIPRGLLAVTFLLVVFNCLSSFQIYSMPVFDSFEAGYTSRTNRPCSIWVRSGFRIFYGFISFFIGIALPFLSSLAGLL

GGLTLPVTFAYPCFMWVLIKRPTRFSFSWYLNWTLGILGIAFSMALSAGGVWSMVNSGLKLKFFKPS*

>scaffold00166.21

MEDHDGLQKERNEAKALNDDSGLNTAHRIDHDPWLQVGLLLVTGYNCGYIVSFSNLILKPLGWTWGLIAMVFIAILSLYA

NWLLAGFHIIDGQRFIRYRDLMGCLFGKKIHHMTWALQVLNLLFANMGFILLAGNSLKESYISPSFSLYSLCEIFPLEIH

AAFTSSPLRLQDYIIISGVICFLFAFIVPNMSSMGAWFALSGFFTLIYVVTIIAVSIKDGKSNIKKDYSVSRSNVEKVFN

AFNAISAILFTNTSGMLPEIQCTLHKPAVKNMRKALYLQFTLGLGIYYVITIIGYWAYGSHVSEYLPDQFSGPKWASIIA

NSAIFFQTIISQNVFCSPIHEALDTRFLRLDQSLYSLDNVKRRLLLRGGLFLLNTFIAAMFPFLGDFVNLVGSFSLFPLT

FIFPSMIFIKVQGRKANSAVKAWHWLNIIGFSFVTLVTTIAALRLIVQNAKLYHLFADT*

>AtAAP1

MKSFNTEGHNHSTAESGDAYTVSDPTKNVDEDGREKRTGTWLTASAHIITAVIGSGVLSLAWAIAQLGWIAGTSILLIFSFITYFTSTMLADCYRAPDPVTGKRNYTYMDVVRSYLGGRKVQLCGVAQYGNLIGVTVGYTITASISLVAVGKSNCFHDKGHTADCTISNYPYMAVFGIIQVILSQIPNFHKLSFLSIMAAVMSFTYATIGIGLAIATVAGGKVGKTSMTGTAVGVDVTAAQKIWRSFQAVGDIAFAYAYATVLIEIQDTLRSSPAENKAMKRASLVGVSTTTFFYILCGCIGYAAFGNNAPGDFLTDFGFFEPFWLIDFANACIAVHLIGAYQVFAQPIFQFVEKKCNRNYPDNKFITSEYSVNVPFLGKFNISLFRLVWRTAYVVITTVVAMIFPFFNAILGLIGAASFWPLTVYFPVEMHIAQTKIKKYSARWIALKTMCYVCLIVSLLAAAGSIAGLISSVKTYKPFRTMHE*

>AtAAP2

MGETAAANNHRHHHHHGHQVFDVASHDFVPPQPAFKCFDDDGRLKRTGTVWTASAHIITAVIGSGVLSLAWAIAQLGWIAGPAVMLLFSLVTLYSSTLLSDCYRTGDAVSGKRNYTYMDAVRSILGGFKFKICGLIQYLNLFGIAIGYTIAASISMMAIKRSNCFHKSGGKDPCHMSSNPYMIVFGVAEILLSQVPDFDQIWWISIVAAVMSFTYSAIGLALGIVQVAANGVFKGSLTGISIGTVTQTQKIWRTFQALGDIAFAYSYSVVLIEIQDTVRSPPAESKTMKKATKISIAVTTIFYMLCGSMGYAAFGDAAPGNLLTGFGFYNPFWLLDIANAAIVVHLVGAYQVFAQPIFAFIEKSVAERYPDNDFLSKEFEIRIPGFKSPYKVNVFRMVYRSGFVVTTTVISMLMPFFNDVVGILGALGFWPLTVYFPVEMYIKQRKVEKWSTRWVCLQMLSVACLVISVVAGVGSIAGVMLDLKVYKPFKSTY*

>AtAAP3

MVQNHQTVLAVDMPQTGGSKYLDDDGKNKRTGSVWTASAHIITAVIGSGVLSLAWATAQLGWLAGPVVMLLFSAVTYFTSSLLAACYRSGDPISGKRNYTYMDAVRSNLGGVKVTLCGIVQYLNIFGVAIGYTIASAISMMAIKRSNCFHKSGGKDPCHMNSNPYMIAFGLVQILFSQIPDFDQLWWLSILAAVMSFTYSSAGLALGIAQVVVNGKVKGSLTGISIGAVTETQKIWRTFQALGDIAFAYSYSIILIEIQDTVKSPPSEEKTMKKATLVSVSVTTMFYMLCGCMGYAAFGDLSPGNLLTGFGFYNPYWLLDIANAAIVIHLIGAYQVYCQPLFAFIEKQASIQFPDSEFIAKDIKIPIPGFKPLRLNVFRLIWRTVFVIITTVISMLLPFFNDVVGLLGALGFWPLTVYFPVEMYIAQKKIPRWSTRWVCLQVFSLGCLVVSIAAAAGSIAGVLLDLKSYKPFRSEY*

>AtAAP4

MDVPRPAFKCFDDDGRLKRSGTVWTASAHIITAVIGSGVLSLAWAIGQLGWIAGPTVMLLFSFVTYYSSTLLSDCYRTGDPVSGKRNYTYMDAVRSILGGFRFKICGLIQYLNLFGITVGYTIAASISMMAIKRSNCFHESGGKNPCHMSSNPYMIMFGVTEILLSQIKDFDQIWWLSIVAAIMSFTYSAIGLALGIIQVAANGVVKGSLTGISIGAVTQTQKIWRTFQALGDIAFAYSYSVVLIEIQDTVRSPPAESKTMKIATRISIAVTTTFYMLCGCMGYAAFGDKAPGNLLTGFGFYNPFWLLDVANAAIVIHLVGAYQVFAQPIFAFIEKQAAARFPDSDLVTKEYEIRIPGFRSPYKVNVFRAVYRSGFVVLTTVISMLMPFFNDVVGILGALGFWPLTVYFPVEMYIRQRKVERWSMKWVCLQMLSCGCLMITLVAGVGSIAGVMLDLKVYKPFKTTY*

>AtAAP5

MVVQNVQDLDVLPKHSSDSFDDDGRPKRTGTVWTASAHIITAVIGSGVLSLAWAVAQIGWIGGPVAMLLFSFVTFYTSTLLCSCYRSGDSVTGKRNYTYMDAIHSNLGGIKVKVCGVVQYVNLFGTAIGYTIASAISLVAIQRTSCQQMNGPNDPCHVNGNVYMIAFGIVQIIFSQIPDFDQLWWLSIVAAVMSFAYSAIGLGLGVSKVVENKEIKGSLTGVTVGTVTLSGTVTSSQKIWRTFQSLGNIAFAYSYSMILIEIQDTVKSPPAEVNTMRKATFVSVAVTTVFYMLCGCVGYAAFGDNAPGNLLAHGGFRNPYWLLDIANLAIVIHLVGAYQVYCQPLFAFVEKEASRRFPESEFVTKEIKIQLFPGKPFNLNLFRLVWRTFFVMTTTLISMLMPFFNDVVGLLGAIGFWPLTVYFPVEMYIAQKNVPRWGTKWVCLQVLSVTCLFVSVAAAAGSVIGIVSDLKVYKPFQSEF*

>AtAAP6

MEKKKSMFVEQSFPEHEIGDTNKNFDEDGRDKRTGTWMTGSAHIITAVIGSGVLSLAWAIAQLGWVAGPAVLMAFSFITYFTSTMLADCYRSPDPVTGKRNYTYMEVVRSYLGGRKVQLCGLAQYGNLIGITIGYTITASISMVAVKRSNCFHKNGHNVKCATSNTPFMIIFAIIQIILSQIPNFHNLSWLSILAAVMSFCYASIGVGLSIAKAAGGGEHVRTTLTGVTVGIDVSGAEKIWRTFQAIGDIAFAYAYSTVLIEIQDTLKAGPPSENKAMKRASLVGVSTTTFFYMLCGCVGYAAFGNDAPGNFLTGFGFYEPFWLIDFANVCIAVHLIGAYQVFCQPIFQFVESQSAKRWPDNKFITGEYKIHVPCCGDFSINFLRLVWRTSYVVVTAVVAMIFPFFNDFLGLIGAASFWPLTVYFPIEMHIAQKKIPKFSFTWTWLKILSWTCFIVSLVAAAGSVQGLIQSLKDFKPFQAP*

>AtAAP7

MDIKEDDESRVITPTELQLHDSVTARTGTLWTAVAHIITGVIGAGVLSLAWATAELGWIAGPAALIAFAGVTLLSAFLLSDCYRFPDPNNGPLRLNSYSQAVKLYLGKKNEIVCGVVVYISLFGCGIAYTIVIATCSRAIMKSNCYHRNGHNATCSYGDNNNYFMVLFGLTQIFMSQIPNFHNMVWLSLVAAIMSFTYSFIGIGLALGKIIENRKIEGSIRGIPAENRGEKVWIVFQALGNIAFSYPFSIILLEIQDTLRSPPAEKQTMKKASTVAVFIQTFFFFCCGCFGYAAFGDSTPGNLLTGFGFYEPFWLVDFANACIVLHLVGGYQVYSQPIFAAAERSLTKKYPENKFIARFYGFKLPLLRGETVRLNPMRMCLRTMYVLITTGVAVMFPYFNEVLGVVGALAFWPLAVYFPVEMCILQKKIRSWTRPWLLLRGFSFVCLLVCLLSLVGSIYGLVGAKFG*

>AtAAP8

MDAYNNPSAVESGDAAVKSVDDDGREKRTGTFWTASAHIITAVIGSGVLSLAWAIAQLGWVAGTTVLVAFAIITYYTSTLLADCYRSPDSITGTRNYNYMGVVRSYLGGKKVQLCGVAQYVNLVGVTIGYTITASISLVAIGKSNCYHDKGHKAKCSVSNYPYMAAFGIVQIILSQLPNFHKLSFLSIIAAVMSFSYASIGIGLAIATVASGKIGKTELTGTVIGVDVTASEKVWKLFQAIGDIAFSYAFTTILIEIQDTLRSSPPENKVMKRASLVGVSTTTVFYILCGCIGYAAFGNQAPGDFLTDFGFYEPYWLIDFANACIALHLIGAYQVYAQPFFQFVEENCNKKWPQSNFINKEYSSKVPLLGKCRVNLFRLVWRTCYVVLTTFVAMIFPFFNAILGLLGAFAFWPLTVYFPVAMHIAQAKVKKYSRRWLALNLLVLVCLIVSALAAVGSIIGLINSVKSYKPFKNLD*

>OsAAP1

MGMERPQEKVATTTTAAFNLAESGYADRPDLDDDGREKRTGTLVTASAHIITAVIGSGVLSLAWAIAQLGWVIGPAVLVAFSVITWFCSSLLADCYRSPDPVHGKRNYTYGQAVRANLGVAKYRLCSVAQYVNLVGVTIGYTITTAISMGAIKRSNWFHRNGHDAACLASDTTNMIIFAGIQILLSQLPNFHKIWWLSIVAAVMSLAYSTIGLGLSIAKIAGGAHPEATLTGVTVGVDVSASEKIWRTFQSLGDIAFAYSYSNVLIEIQDTLRSSPAENEVMKKASFIGVSTTTTFYMLCGVLGYAAFGNRAPGNFLTGFGFYEPFWLVDVGNVCIVVHLVGAYQVFCQPIYQFAEAWARSRWPDSAFVNGERVLRLPLGAGDFPVSALRLVWRTAYVVLTAVAAMAFPFFNDFLGLIGAVSFWPLTVYFPVQMYMSQAKVRRFSPTWTWMNVLSLACLVVSLLAAAGSIQGLIKSVAHYKPFSVSS*

>OsAAP2

MASVDLELGRPLSAAAAAYPPPLRRSINDDDVDDDGKPKRTGTEWTASAHIVTAVVGSGVLSLAWSTAQLGWVAGPATLVVFAVITYYTSVLLADCYRAGGDQVSGKRNYTYMDAVESYLGGRQVWFCGLCQYVNLVGTAIGYTITASISAAAVYKSNCFHKNGHSADCSVFTTSYMVVFGVVQVFFSQLQSLHEVAWLSVLAAVMSFSYSAIAVGLSLAQTISGPTGMTTMSGTVIGIDVDLSHKIWQALQALGNIAFAYSYSLVLIEIQDTIRSPPAESKTMRKANALAMPVITAFYTLCGCLGYAAFGNAAPGNMLTGFGFYDPYWLVGLANACIVVHLVGAYQVMSQPVFTAVESWASSRWPRCGFFVTGGGGTRLISVNAFRLAWRTAYVVACTAVAAVVPFFNDVLGLLGAVGFWPLTVYFPVEMYIRRRKLERSSKRWVALQSLNAVCFVVTLASAVASVQGIAESMAHYVPFKSKL*

>OsAAP3

MAKDVEMAVRNGDGGGGGGYYATHPHGGAGGEDVDDDGKQRRTGNVWTASAHIITAVIGSGVLSLAWATAQLGWVVGPVTLMLFALITYYTSGLLADCYRTGDPVSGKRNYTYMDAVAAYLGGWQVWSCGVFQYVNLVGTAIGYTITASISAAAVHKANCYHKNGHDADCGVYDTTYMIVFGVVQIFFSMLPNFSDLSWLSILAAVMSFSYSTIAVGLSLARTISGATGKTTLTGVEVGVDVTSAQKIWLAFQALGDIAFAYSYSMILIEIQDTVKSPPAENKTMKKATLLGVSTTTAFYMLCGCLGYAAFGNAAPGNMLTGFGFYEPYWLIDFANVCIVVHLVGAYQVFCQPIFAAVETFAARRWPGSEFITRERPVVAGRSFSVNMFRLTWRTAFVVVSTVLAIVMPFFNDILGFLGAVGFWPLTVYYPVEMYIRQRRIQRYTSRWVALQTLSLLCFLVSLASAVASIEGVSESLKHYVPFKTKS*

>OsAAP4

MDRRAVVYDAEAVDDHERQGTVWTATSHIVAAVVGSGVLALAWTVAQLGWVVGPLVLVGFSCVTYYTSTLLANCYRYPDPVTGTANREYIDAVRCYLGPKNVMLCGCAQYVNLWGTLVGYTITASASMIAVKRVNCFHREGYGAGDCGASGSTYMVVFGVFQLLLSQLPSLHNIAWLSVVAVATSFGYSFISLGLCAAKWASHGGAVRGTLAGADLDFPRDKAFNVLLALGNIAFSYTFADVLIEIQDTLRSPPAENKTMKRASFYGLSMTTVFYLLLGCTGYAAFGNDAPGNILTGFAFYEPFWLVDIANICVIVHLIGAYQVFAQPIFARLESYVACQWPDAKFINATYYVRVPGRWWPAATVAVAPLKLVLRTIIIMFTTLVAMLLPFFNAVLGLIGALGFWPLSVYFPVSMHVARLGIRRGEPRWWSLQAMSFVCLLISIAASIGSVQDIVHNLKAAAPFKTVN*

>OsAAP5

MNKNAAPEDVESGEHERTGTVWTATAHIVTAVIGSGVLALAWSVAQLGWVAGPLALAGFACVTYYTSTLLANAYRAPHPVTGTRNRTYMDAVRSYLSPREVFMCGIAQYVNLWGTMVGYTITATISMVAIRRSDCIHRNGAGAAARCDNTSATVLMLAFSIVQVVLSQFPGLEHITWLSVVAAVMSFAYSFIGLGLSVAEWVSHGGHLSGRIQGATAASSSKKLWNVLLALGNIAFAYTFAEVLIEIQDTLKPSPPENKTMKKAAMYGIGATTIFYISVGCAGYAAFGSDAPGNILTASGMGPFWLVDIANMCLILHLIGAYQVYAQPIFATMERWISSRWPEAKFINSEYTVNVPLIQRGSVTVAPYKLVLRTVVVIATTVVAMMIPFFNAVLGLLGAFSFWPLTVYFPISMHIAQEKITRGGRWYLLQGLSMVCLMISVAVGIGSVTDIVDSLKVATPFKTVS*

>OsAAP6

MDVEKVERKEVAVDDDGRVRTGTVWTATTHAITAVIGSGVLALPWSVAQMGWVLGPIALVVCAYITYYTAVLLCDCYRTPDPVHGKRNYTYMDVVRSCLGPRDVVVCGIAQYAILWGAMVGYTITTATSIMSVVRTNCHHYKGPDATCGSSGTMYMVLFGLAEVVLSQCPSLEGVTLISVVAAVMSFTYSFVGLFLSAAKVASHGAAHGTLLGVRVGAGGVTASTKAWHFLQALGNIAFAYTYSMLLIEIQDTVKSPPSENVTMKRASLYGIGVTTVFYVSIGCVGYAAFGNAAPGNVLTGFLEPFWLVDIANVAVVIHLVGAYQVYAQPVFACYEKWLASRWPESAFFHREYAVPLGGGRAVRFTLCKLVLRTAFVAVTTVVSLVLPFFNAVLGLLGAVAFWPLTVYFPVTMYMAQAKVQRGSRKWVALQALNVGALVVSLLAAVGSVADMAQRLRHVTIFQTQL*

>OsAAP7

MGENGVVASKLCYPAAAMEVVAAELGHTAGSKLYDDDGRLKRTGTMWTASAHIITAVIGSGVLSLGWAIAQLGWVAGPAVMLLFSFVTYYTSALLADCYRSGDESTGKRNYTYMDAVNANLSGIKVQVCGFLQYANIVGVAIGYTIAASISMLAIKRANCFHVEGHGDPCNISSTPYMIIFGVAEIFFSQIPDFDQISWLSILAAVMSFTYSTIGLGLGVVQVVANGGVKGSLTGISIGVVTPMDKVWRSLQAFGDIAFAYSYSLILIEIQDTIRAPPPSESRVMRRATVVSVAVTTLFYMLCGCTGYAAFGDAAPGNLLTGFGFYEPFWLLDVANAAIVVHLVGAYQVYCQPLFAFVEKWAQQRWPKSWYITKDIDVPLSLSGGGGGGGRCYKLNLFRLTWRSAFVVATTVVSMLLPFFNDVVGFLGAVGFWPLTVYFPVEMYIVQKRIPRWSTRWVCLQLLSLACLAITVASAAGSIAGILSDLKVYKPFATTY*

>OsAAP8

MGENVVGTYYYPPSAAAMDGVELGHAAAGSKLFDDDGRPRRNGTMWTASAHIITAVIGSGVLSLGWAIAQLGWVAGPAVMVLFSLVTYYTSSLLSDCYRSGDPVTGKRNYTYMDAVNANLSGFKVKICGFLQYANIVGVAIGYTIAASISMLAIGRANCFHRKGHGDPCNVSSVPYMIVFGVAEVFFSQIPDFDQISWLSMLAAVMSFTYSVIGLSLGIVQVVANGGLKGSLTGISIGVVTPMDKVWRSLQAFGDIAFAYSYSLILIEIQDTIRAPPPSESAVMKRATVVSVAVTTVFYMLCGSMGYAAFGDDAPGNLLTGFGFYEPFWLLDIANAAIVVHLVGAYQVFCQPLFAFVEKWAAQRWPESPYITGEVELRLSPSSRRCRVNLFRSTWRTAFVVATTVVSMLLPFFNDVVGFLGALGFWPLTVYFPVEMYVVQKKVPRWSTRWVCLQMLSVGCLVISIAAAAGSIAGVMSDLKVYRPFKGY*

>OsAAP9

MLPRSRTLPPRIHDGVVVVERDVRRYQQLPQQVEMEMTTTKRQQDHQVETMTTKKIDEEDEEVDDDGRAKRRGTVWTAASHIITAVIGSGVLSLAWAIAQLGWVVGPTVMLLFAAVIYFTSNLLADCYRTGDPATGRRNYTYMDAVKANLGGAKVKVCGCIQYLNLLGVAIGYTIAASISMMAIQRSNCFHARGEQDPCHASSNVYMIMFGIVQVFFSQIPDFDQVWWLSILAAVMSFTYSAVGLALGAAQVAQNRTFAGSAMGVAVGFVTKTGDVVTPAQKVWRNLQALGDIAFAYSYSIILIEIQDTLRSPPAEARTMRKATGISVVVTSVFYLLCGCMGYAAFGDDAPGNLLTGFGFYKPYWLLDVANMAIVVHLVGAYQVYCQPLFAFVERRAERRWPNGLPGGDYDLGWIKVSVFRLAWRTCFVAVTTVVAMLLPFFNDVVGILGALGFWPLTVYFPVEMYIAHRRIRRWTTTWVGLQALSLACLLVSLAAAVGSIAGVLLDLKSYRPFRSTY*

>OsAAP10

MGGAGEGDGQTEPLLEKLSNSSSSEIDKRTGTAWTATAHIITAVIGSGVLSLAWSVAQLGWVGGPAAMVLFAGVTLVQSSLLADCYIFHDPDNGVVRNRSYVDAVRFYLGEKSQWFCGFFLNINFFGSGVVYTLTSATSMRAIQKANCYHREGHDAPCSVGGDGYYMLMFGLAQVVLSQIPGFHDMAWLSVLSAAMSFTYSLIGFGLGVAKVITNGVIKGGIGGIAMVSATQKVWRVSQAIGDIAFAYPFASVLLEIEDTLRSPPPESETMRTASRASIAVTTFFYLCCGCFGYAAFGDATPGNLLTGFGFYEPYWLIDFANLCVAVHLLGGYQVYSQPVFAAVERRMGGAGAGVVEVAVPAAVAWPSRWRRGCRVNVYRLCFRTAYVAATTALAVWFPYFNQVVGLLGAFTFWPLSIHFPVEMYLVQKKVAPWTPRWLAVRAFSAACLATGAFASVGSAVGVFSSKTS*

>OsAAP11

MGKAAAMEVSASAAAEAGMMVGHGEWRDDDGRARRMGTVWTASAHIITAVIGSGVLSLAWAIAQLGWVAGPAVMLLFAFVIYYTSTLLAECYRSGDPCTGKRNYTYMDAVRANLGGSKVRLCGVIQYANLFGVAIGYTIAASISMLAIKRADCFHEKGHKNPCRSSSNPYMILFGVVQIVFSQIPDFDQIWWLSIVAAIMSFTYSTIGLSLGIAQTVANGGFMGSLTGISVGTGVTSMQKVWRSLQAFGDIAFAYSYSIILIEIQDTIKAPPPSEAKVMKRATMVSVATTTVFYMLCGCMGYAAFGDKSPDNLLTGFGFYEPFWLLDVANAAIVVHLVGAYQVFVQPIFAFVERWAAARWPDGGFISRELRVGPFSLSVFRLTWRTAFVCATTVVSMLLPFFGDVVGLLGAVSFWPLTVYFPVEMYIAQRGVRRGSARWLCLKVLSAACLVVSVAAAAGSIADVVDALKVYRPFSG*

>OsAAP12

MVQIEPLEVSLEAGNQADSALLDDDGRPRRTGTFWTASAHIITAVIGSGVLSLPWATAQLGWVGGPAVMVVFGGVTYFTATLQAECYRTGDEETGARNYTYIGAVRAILGGANAKLCGIIQYANLVGTAVGYTIAASISMQAIKRAGCFHANGHNVPCHISSTPYMLIFGAFEIVFSQIPDFHEIWWLSIVAAVMSFTYSGVGLGLGIAQTVADGGFRGTIAGVTNVTATQKAWRSLQALGNIAFAFAFSNVYTEIQDTIKAPPPSEAKVMKQASLLSIVATSVFYALCGWMGYAAFGNAAPDNLLTGFGFFEPFWLVDAANVAIAVHLIGAYQVYCQPVFAFVERKASRRWPDSGFVNSELRVWPFAISAFRLAWRSVFVCFTTVVAMALPFFGVIVGLLGAISFWPLTVYLPTEMYIAQRGVRRGSALWIGLRALAVAGFVVSAAATTGAVANFVGDFMKFRPFSG*

>OsAAP13

MALGDGDDGAAAAAVPLISDRPKHAAIVRSGTEWTAAAHVITAVIGSGVLSLAWSVAQLGWLAGPGMMLVFAAVTALQSALFADCYRSPDPEVGPHRNRTYANAVERNLGSSSAWVCLLLQQTALFGYGIAYTITASISCRAILRSNCYHTHGHDAPCKYGGSYYMLMFGAAQLFLSFIPDFHDMAWLSVLAAVMSFSYSFIGLGLGLANTIANGTIKGSITGAPTRTPVQKVWHVSQAIGDIAFAYPYSLILLEIQDTLKAPPAENKTMKKASIISIVVTTFFYLCCGCFGYAAFGSDAPGNLLTGFGFYEPYWLIDFANACIILHLLGGYQVYSQPIYQFADRFFAERYPASRFVNDFHTVKLPLLPPCRVNLLRVCFRTVYVASTTAVALAFPYFNEVLALLGALNFWPLAIYFPVEMYFIQRHVPRWSPRWVVLQSFSVLCLLVSAFALVGSIQGLISQKLG*

>OsAAP14

MAPQLPLEVASAPKLDDDGHPQRTGNLWTCVAHIITAVIGCGVLALSWSVAQLGWVAGPIAMVCFAFVTYISAFLLSHCYRSPGSEKMQRNYSYMDAVRVHLGRKHTWLCGLLQYLNLYGIGIAYTITTATCMRAIKRANCYHSEGRDAPCDSNGEHFYMLLFGAAQLLLSFIPNFHKMAWLSVVAAIMSFAYSTIGLGLGLAKTIGDGTVKGNIAGVAMATPMQKVWRVAQAIGDIAFAYPYTIVLLEIQDTLRSPPPESETMQKGNVIAVLATTFFYLCVGCFGYSAFGNAAPGNLLTGFGFYEPYWLIDFANACIVLHLLGGYQMFSQQIFTFADRCFAASFPNSAFVNRSYSVKILPWRRGGGGGGAGRYEVNLQRVCFRTVYVASTTGLALVFPYFNEVLGVLGALVFWPLAIYLPVEMYCVQRRISPWTPRWAALQAFSVVCFVVGTFAFVGSVEGVIRKRLG*

>OsAAP15

MASGQKVVKPMEVSVEAGNAGEAAWLDDDGRARRTGTFWTASAHIITAVIGSGVLSLAWAIAQLGWVAGPAVMLLFAFVIYYTSTLLAECYRTGDPATGKRNYTYMDAVRANLGGAKVTFCGVIQYANLVGVAIGYTIASSISMRAIRRAGCFHHNGHGDPCRSSSNPYMILFGAVQIVFSQIPDFDQIWWLSIVAAVMSFTYSGIGLSLGIVQTISNGGIQGSLTGISIGVGVSSTQKVWRSLQAFGDIAFAYSFSNILIEIQDTIKAPPPSEAKVMKSATRLSVATTTVFYMLCGCMGYAAFGDAAPDNLLTGFGFYEPFWLLDVANVAIVVHLVGAYQVFVQPIFAFVERWASRRWPDSAFIAKELRVGPFALSLFRLTWRSAFVCLTTVVAMLLPFFGNVVGLLGAVSFWPLTVYFPVEMYIAQRGVPRGSARWVSLKTLSACCLVVSIAAAAGSIADVIDALKVYRPFSG*

>OsAAP16

MASGQKVVKPMEVSVEAGNAGEAAWLDDDGRARRTGTFWTASAHIITAVIGSGVLSLAWAIAQLGWVAGPAVMLLFAFVIYYTSTLLAECYRTGDPATGKRNYTYMDAVRANLGGAKVTFCGVIQYANLVGVAIGYTIASSISMRAIRRAGCFHHNGHGDPCRSSSNPYMILFGVVQIVFSQIPDFDQIWWLSIVAAVMSFTYSGIGLSLGIVQTISNGGIQGSLTGISIGVGVSSTQKVWRSLQAFGDIAFAYSFSNILIEIQDTIKAPPPSEAKVMKSATRLSVATTTVFYMLCGCMGYAAFGDAAPDNLLTGFGFYEPFWLLDVANVAIVVHLVGAYQVFVQPIFAFVERWASRRWPDSAFIAKELRVGPFALSLFRLTWRSAFVCLTTVVAMLLPFFGNVVGLLGAVSFWPLTVYFPVEMYIAQRGVPRGSARWVSLKTLSACCLVVSIAAAAGSIADVIDALKVYRPFSG*

>OsAAP17

MAHTSSQKHGNDDVDTGAEAAMDQLAGRSSLSPAREKTRRRPEKSGTVWTATAHIVALLIGSSVLAVAWTFAQLGWVAGPAVVVALSVVTYYSSALLADCYRDDDPLHLGGGAVHGEYIAAVRSYLGPKSVTFCGIIQYGVLWAAMVGYTITSSSSMSAVRRVNRFHRNWLAAGDGDGGGGGGGATGVRYMVVFGAFQLLLSQLPSLENVAWLSVIAVATSFGYSSICLGLCAAKWASHRGGVRGTLAGAAAGSPGEKVFNVLLAVGNIAISYIYSPVLFEIQDTVRTPPSESKTMKRASLYGLAMSAVFYLVLGASGYAAFGDDAPSNILTGAAFHEPFWLVDVANACVVVHFLGAYQVIAQPVFARLEAYVGGRWPESRLVTASYELRLRVPAWTSAPPTAVTLSPARMALRAAVIVATTAVAAMMPFFNAVLGFIAALGFWPLAVYLPVSMHIARVKIRRGEARWWALQGASAALLVVAVGMGVASVRDMVQRLNEAAPFKTTG*

>OsAAP18

MGGGTNGNGGGAASAMDVYLPRTQGDVDDDGKERRTGTVWTATAHIITAVIGSGVLSLAWAMAQLGWVAGPITLLLFAAITFYTCGLLSDCYRVGDPATGKRNYTYTDAVKSYLGGWHVWFCGFCQYVNMFGTGIGYTITASISAAAINKSNCYHWRGHGTDCSQNTSAYIIGFGVLQALFCQLPNFHQLWWLSIIAAVMSFSYAAIAVGLSLAQTIMDPLGRTTLTGTVVGVDVDATQKVWLTFQALGNVAFAYSYAIILIEIQDTLRSPPPENATMRRATAAGISTTTGFYLLCGCLGYSAFGNAAPGNILTGFGFYEPYWLVDVANACIVVHLVGGFQVFCQPLFAAVEGGVARRCPGLLGGGAGRASGVNVFRLVWRTAFVAVITLLAILMPFFNSILGILGSIAFWPLTVFFPVEMYIRQRQLPRFSAKWVALQSLSLVCFLVTVAACAASIQGVLDSLKTYVPFKTRS*

>OsAAP19

MSLADDLAAVERGGHMVPSKAAGVDGDGEPRRTGTMWTASAHIITAVIGSGVLSLAWGVAQLGWVAGPAVMLLFGAVIYCCSVLLVECYRTGDPYTGQRNRTYMDAVRANLGGTKVVFSQIPNLHKMWWLSTLASAMSLSYSAIGIALGVAQIVVLDMFEIEFAANGGIRGTITGVFVGAGAGVTSMQKDTVKPVAPPSTETKVMRKAVAVSVATTTAVYLMCGCVGYAAFGNDSPDNLLTGFGFFEPFWLLDLANAGVVVHLVGTYQVVAQPVFAFLDGRAAAGAWPGSAALGKRRRVLRVGSLAEIEVSPFRLAWRTAFVCVTTAASTLLPFFGSMVGLIGAASFWPLTVYFPVEMYIAQRRVPRGSAQWLSLQALSAGCLVVSVAASAGSIAGVVEAFKAHNPFCWTC*

>StAAP1

MAPEFQKNAMYVSNELENGDVQKNFDDDGREKRTGTLLTASAHIITAVIG

SGVLSLAWAIAQLGWVAGPAVLFAFSFITYFTSTLLADCYRSPGPISGKR

NYTYMDVVRSHLGGVKVTLCGIAQYANLVGVTIGYTITASISMVAVKRSN

CFHKNGHEASCSIESYPYMIIFAVIQIVLSQIPNFHKLSWLSILAAVMSF

TYASIGLGLSIAKVSGVGHHVKTSLTGVVVGVDVSGTEKVWRSFQAIGDI

AFAYAYSTVLIEIQDTLKSSPPESKVMKRASLAGVSTTTLFYVLCGTIGY

AAFGNNAPGNFLTGFGFYEPFWLIDFANVCIAVHLVGAYQVFCQPLYGFV

EARCSQRWPDSKFITSEYAMRVPCCGTYNLNLFRLVWRTTYVIVTAVIAM

IFPFFNDFLGLIGAASFYPLTVYFPIEMYIAQRKIPKYSFTWVWLKILSW

TCLIVSLVAAAGSIQGLATDVKGYKPFSTHQ*

>StAAP2

MADHVSLSVPDESTCFDDDGRPKRSGTVWTASAHIITAVIGSGVLSLAWA

TAQLGWIAGPSVLLLFSAVTYYTCSFLSDCYRTGDQLTGRRNYTYMDVVR

SNLGDVHVKICGVIQYVNLVGVAIGYTIASSISMVAVKRSNCFHNNGHDH

ASCNISSTPYKIMFGVLEIVLSQILNFDQISWLSIAAALMSFTYKTIGLG

LGVAKVVQTGKIQGSISTGTEIDKVWKSFQALGAIASAYSYSLILIEIQD

TLIKSPSAEAKTMKKATQISLVVTTVLYMLCGCFGYAAFGDQSPENLLTG

FGFDDPYWLLDIANVAVVIHLVGAYQVSCQTLFAFVEKKAGEWYPDSNII

TTEIDIPIHRCKPFKLNFFHLIWRTIFVIATTFISVLMPSFNNIVDILGA

FAFWPLTVYFPIKMYIVKKNIPKWSGRWICLQLLSGACLVISIAVAVGSF

AGLVSDLKAFKPFK*

>StAAP3

MTDHVSVTVYDESSSFDDDGRLKRRGNVWTASAHIITAVIDSGVLIVAWA

TAQLGWIAGPVILLLFSIVTYYTSNLLSDCYRKGDQLTGKRNYTYMDAVR

ANLGGVHVKICGILQYANIVGVAVGYTIASSISMVAVKRSNCFHEYGHQA

ACNVSTTPYMIAFGVVQIVLSQIPDFDQISWLSIVAAVMSMTYSTIGLGL

GVAKVAETGKVQGSLTGVSGGTGMQKIWKSSQAVGAIAFAYSYSLILIEI

QDTIKSPPSEAKTMKKASLISVTVTTVFFILCGCFGYAAFGDQSPGSLLT

GFGFYDPYWLLNIANMAVVVHLVGAYQVYCQPLFAYVEKTAAERYPDSII

IMKEIDIPIPGCKPFKLNFFRLVWRTVFVIFTVLISMLMPFFNDIVGILG

AFGFWPLTVYFPVKMYIVEKNITKWSGRWICLQLLSGACLVISIAAAAGS

FAGLVSDLQVSWPFKTLN*

>StAAP4

MTMADKAHQVFEVYGESKCFDDDGRIKRTGSVWTASAHIITAVIGSGVLS

LAWATAQLGWVAGPTVLLLFSFVTYYTSALLSDCYRTGDPVTGKRNYTYM

DAVRANLGGFQVKICGVIQYANLFGVAIGYTIAASISMVAVNRSNCFHKQ

GHRAACNVSSTPYMIIFGVMEIIFSQIPDFDQISWLSIVAAVMSFTYSTI

GLGLGVAQVAETGKIEGSLTGISIGTEVTEMQKIWRSFQALGAIAFAYSY

SLILIEIQDTLKSPPAEAKTMKRATLISVAVTTVFYMLCGCFGYAAFGDQ

SPGNLLTGFGFYNPYWLLDIANVAIVVHLVGAYQVYCQPLFAFVEKTATE

WYPDSKIITKEIDVPIPGFKPFKLNLFRLVWRTIFVIITTVISMLMPFFN

DVVGILGAFGFWPLTVYFPVEMYIVQKRITKWSGRWICLQILSGACLVIS

IAAAAGSFAGVASDLKVYRPFQS*

>StAAP5

MEAQRSLEQEKTSGKEDEDTIRTGTLWTAVAHIISAVIGAGVLSLAWCTA

QLGWIAGPITMLCFAVVTYISASLICDCYRSPDPITGTRNPSYIDAVRVN

LGKKWTWLCGLLQYVSFYGTGIAYVITSATSMREIQRSNCYHKGGEEAVC

QTGTNNFMLIFGIIQIVTSQIPNFHNMAWLSVVAALMSFCYSFIGLGLGF

SKVIENRGIKGSIVGVPTRSAAQKIWLVFQALGDIAFAYPYSIILLEIQD

TLKSPPPENQTMKKASISAIVITTFFYLCCSCFGYAAFGNDTPGNLLTGF

YEPFWLVDFANACIVLHLVGGYQVYSQPVFAFVEKWATQKYPESRFINKF

YAIKLQVLPALQLNLFRLCFRTLYVISTTAIAMAFPYFNQVLGILGALNF

WPMTIYFPVEMYIVQRKIGAWTRKWILLEGFSMVCLIVSLLGLIGSIEGI

VSAKLA*

>StAAP6

MGLDEESDSQMPFLDPNYVSSSSSSSHSSKLALKRTGNEWTALAHIITAV

IGSGVLSLAWSMAQLGWIAGPLTMLVFACVSLTSVFLLCNCYKSPDPEIG

PDRNGCYLDAVQKILGKRNAWFCGIAVRINFIKVAIIYTITSASSMQAIQ

KSNCYHDQGHKATCGYESTRYMVIFGLIQVIVSQIPDFPNMKWLSVVAAV

MSFTYAIIGSALGLAKVIENGEIKGSITGLPSSTAAEKVWLVAQALGNIA

FAFPFSLIFLEVQDTLKAPPPEKITMKKVSIMASCVTTFFYLCCGGFGYA

AFGNSTPGNLLTGFGFYEPYWLVDFANACVILHLVGGYQVFSQPIFAEVE

RWFARKFPDSKFVHKNHTLKPLSMLSFSLNFMRLFFRTAYVAIMTGIAVL

FPYFNQVVGVSGAITFWPIVVYFPAEMYLTQKRIESWKSKAIAFRVFTMV

CLVVILYAFVGSIRGVIVARFG*

>StAAP7

MGDSTNFASKHQLFDVSVNVTESKRFDDDGRIKRTGSVWTASAHIITAVI

GSGVLSLAWAVAQLGWIAGPIVMLLFSFVTYYTSSLLSDCYRSGDPLSGK

RNYTYMDAVQANLGGLQVKICGWIQYVNLFGVAIGYTIASSISMMAVKRS

DCFHKHGHKAPCLEPNTPYMIIFGVIEIVFSQIPDFDQIWWLSIVAAVMS

FTYSTIGLGLGIAQVAETRKIGGSLTGVSIGTVTEMQKVWRTFQALGAIA

FAYSYSLILIEIQDTIKSPPSEAKTMKNATLISVSVTTVFYMLCGCFGYA

AFGDHAPDNLLTGFGFYDPYWLLDIANIAIVVHLVGAYQVYCQPLFAFIE

KTAAEWYPNSKFITKNISVPIPGYKSYNLNLFRLVWRTIFVIISTFISML

LPFFSDIVGILGAFGFWPLTVYYPVEMYIAQKKIPKWSRKWVGLQILSVT

CLIVSIAAAAGSFAGVVSDLKVYKPFKFT*

>StAAP8

MASEFEKNSTNMYVEQSPKGLENGQVQKNVDDDGREKRTGTVLTASAHII

TAVIGSGVLSLAWAMAQLGWVAGPVILFLFSFITYFTSTLLADCYRFPGP

GSGKRNYSYMEVVRSHLGGFKVQLCGIAQYGNLVGITIGYTITASISMKA

VVRSNCFHKEGHQASCTVSNYPYMVIFAIIQIILSQIQNFHKLSWLSILA

AVMSFAYSLIGLGLSIAKVAGAGHHVKTSLTGTIVGVDVSGSQKVWRCLQ

SIGDIAFAYAFATILIEIQDTLRSPPAENKVMKRASLVGVFTTTLFYVLC

GTIGYAAFGNNAPGNFLTGFGFYEPFWLIDFANVCIAIHLIGAYQVFCQP

IYGFVEGRCSEKWPDNKFIKSQHDINIPWFGVYNLNYFRMLWRTIYVIIT

AIIAMIFPFFNAILGLIGAASFYPLTVYFPIEMHIAQRKIPKYSFKWIWL

HILSWACLIVSLVAAVGSIEGLTQDLKTYKPFKPQDHD*

>ZmAAAP45

MADSRRSVVYDAEGGDDHERQGTAWTATSHIVAAVVGSGVLALAWTVAQLGWVVGPLVLVGFSCVTYYTSALLADCYRYPDPVHGAVNREYIDAVRCYLDRKNVVLCGCAQYVNLWGTLVGYTITASASMIAIKRVNCFHRDGYGAAGCNPSGSTYMVVFGLFQLLLSQLPSLHNIAWLSVVAVATSLGYSFISLGLCSAKWASHGGHVRGTLAGAAAVAGRADDDKQAAFNVLLALGNIAFSYTFADVLIEIQDTLRSPPAENRTMKRASAYGLAITTVFYLALGCTGYAAFGDHAPGNILTGFAFYEPFWLVDAANVCVVLHLVGAYQVFAQPIFARLESCVACRWPDAKLINATYYVRVPPCLLLLRTSSSSSPPPTLAVAPLKLVLRTIVIMFTTLVAMLVPFFNAVLGLIGALGFWPLSVYFPVSMHMARLNIRRGEIRWWMLQAMSFVCLLISVAASIGSVHDIVHNLKAAAPFNTAN*

>ZmAAAP18

MRRQSSLARSCSSVAPASPPPQNGGVNSKHLVPPMEVSAEAGNAGAAEWLDDDGRPRRKGTFWTASAHIITAVIGSGVLSLAWAIAQLGWVAGPTAMLLFAFVTYYTATLLAECYRTGDPDTGKRNYTYMDAVRSNLGGAKVAFCGVIQYANLVGVAIGYTIASSISMKAIRRAGCFHTHGHGDPCKSSSTPYMILFGAAQVVFSQIPDFDQIWWLSIVAAVMSFTYSSIGLSLGIVQTVSNGGFKGSLTSIGFGAGVNSTQKVWHTLQAFGDIAFAYSFSNILIEIQDTIKAPPPSESKVMQKATRLSVATTTVFYMLCGCMGYAAFGDDAPDNLLTGFGFYEPFWLLDVANVAIVVHLVGAYQVFCQPIFAFVERRAAAAWPDSAFVSRELRAGPFALSPFRLAWRSAFVCVTTVVAMLLPFFGDVAGLLGAVSFWPLTVYFPVEMYIKQRRVPRGSARWISLQTLSVTCLLVSIAAAAGSIADVVDALKVYRPFSG*

>ZmAAAP17

MSDDRRTVAYDAEAGDGHERQAGTVWTATSHIVAVVAGSGVLALPWTVAQLGWVLGPLVLVGFSCVTYYTSALLADCYRYPDPVHGAVVNRQYVDAVRCYLVREPMGHSRRLHHHRQRQHDAMKRASFYGLGAATAFYLALGCAGYAAFGDDAPGNVLTGFAFHEPSWLVDAANACVVVHLVGAYQVFAQPIFARLESCAACRWPDAKLVNATYYVRVPPFLLRSASSPPTVAVAPLKLVLRTIVIMFTTLVAMLLPFFNAVLGLIGALGFWPLSVYFPVSMHMARLNIRRGELRWWALQAMSFVCLLVSIGASIGSVQDIVHNLKAAVPFKTVN*

>ZmAAAP64

MAVSHNVGSKHGVAPLEVSVEAGNGGAAEWLDDDGRPRRTGTFWTASAHIVTAVIGSGVLSLAWAIAQLGWVAGPAAMLLFAFVTYYTATLLAECYRTGDPETGKRNYTYMDAVRSNLGGAKVAFCGVIQYANLVGVAIGYTIAASISMKAVRRAGCFHAHGHADPCNSSSTPYMILFGVVQILFSQIPDFDQIWWLSIVAAVMSFTYSSIGLSLGIAQTISNGGFMGSLTGISIGAGVTSTQKIWHTLQAFGDIAFAYSFSNILIEIQVSNNRDLVLYTALQQDFPPLFLTKAAVLTLVLVQDTIKAPPPSESKVMQKATRLSVATTTIFYMLCGCMGYAAFGDKAPDNLLTGFGFFEPFWLIDIANVAIVVHLVGAYQVFCQPIFAFVERRAAAAWPDSAFVSQELRVGPFAVSVFRLTWRSSFVCVTTVVAMLLPFFGNVVGFLGAVSFWPLTVYFPVEMYIKQRRVPRGSTKWICLQTLSVSCLLVSV

>ZmAAAP14

MAAKATGHVGTEAMEVSVEVANGDDDAARLDDDGRPRRRGTMWTASAHIITAVIGAGVLSLAWAMAQLGWAAGTAMMLLFAGISYYTSTLLAECYRCGEPGTGKRNYTYTEAVRAILGGAKFKLCGVIQYANLVGIAVGYTIAASISMLAIKRADCFHDRGHRNPCRSSSNPYMILFGAVEIVFSQIPDFDQIWWLSIVAAAMSFTYATIGLALGIAQTVANGGFKGSLTGVNVGDGITPMQKVWRSLQAFGNISFAYSYAYILIEIQDTIKAPPPSEVTVMKKATMVSVATTTVFYMLCGCMGYAAFGDDAPDNLLTGFGFYEPFWLLDVANAAIVVHLVGAYQVFCQPLFAFVEKRAAARWPDSRFMTRELRLGPFVLGVFRLTWRTAFVCLTTVVAMMLPFFGDVVGLLGAVSFWPLSVYFPVEMYKAQRRVRRWSTRWLCLQTLSAVCLLVSIAGAVGSTAGVINAVNLHRPFSG*

>ZmAAAP55

MDVERKVVEADDDGRVRTGTVWTATTHAITAVIGSGVLALPWSVAQMGWVLGPVALVGCAYITYFTAVLLSDCYRTPDPVHGKRNRTYMDVVRSCLGPRDVVVCGLAQYAILWGTMVGYTITTATSIMAVARTDCRHHRGHDAACASSGTVYMVAFGVVEVVLSQFPSLEKLTIISVVAAVMSCTYSFVGLFLSAAKLASNHGARGSLLGVKIAAGVSASTKTWHSLQALGNVAFAYTYSMLLIEIQDTVKAPPSENVTMKRASFYGISVTTIFYVSLGCIGYAAFGNAAPGNVLTGFDEPFWLVDVANIAVVVHLVGAYQVYAQPIFACYEKWLGSRWPDSAFFHHEYAVRLPGCAVRFTMCKLVLRTAFVAATTVVSLMLPFFNAVLGLLGAIAFWPLTVYFPVTMYIAQAKVAPGSRKWVALQALNVGALLVSLLAAVGSVADMVQRLGHVTIFQTQL*

>ZmAAAP56

MDKSGGEAAAAAAAADDVERRGGDYEQDEHERRGTVWTATAHIVTAVIGSGVLALAWSVAQLGWVAGPLALAGFACVTYYTSTLLAGAYRAPHPVTGHRNRTYMDAVRSYLSPREVFMCGVAQYVNLWGTMVGYTITATISMAAIRQADCLRRDGAGAGARCDAPGTVLMLAFSVVQVVLSQFPGLEHITWLSVVAAAMSFAYSFAGLGLSVGHWVSRGGGGLGGRVAGAAAASSTRKLWNVLLALGNIAFAYTFAEVLIEIQDTLKSPPPENRTMKKAAMYGIGATTIFYISVGCAGYAAFGSNAPGNILAAGGLGPLWLVDIANMCLILHLIGAYQVYAQPVFASVERWAASRWPEAKFMSSAYTVSVSIPLLQRGSVTVAPHKLVLRTAIVGATTAVALAIPFFNAVLGLLGAFSFWPLTVYFPISMHIAQGKIARGTKWWCLLQALSMVCLVISVAVGVGSVTDIVDSLKASSSPFKIVG*

>ZmAAAP52

MVSERQQAAGKVAAFNLTEAGFGDGSDLLDDDGRERRTGTLVTASAHIITAVIGSGVLSLAWAIAQLGWVIGPVVLLAFSAITWFCSSLLADCYRAPPGPGQGKRNYTYGQAVRSYLGESKYRLCSLAQYVNLVGVTIGYTITTAISMGAIKRSNCFHSRGHGADCEASNTTNMIIFAGIQILLSQLPNFHKLWWLSIVAAVMSLAYSSIGLGLSIAKIAGGVHVKTSLTGAAVGVDVTAAEKVWKTFQSLGDIAFAYTYSNVLIEIQDTLRSSPPENVVMKKASFIGVSTTTAFYMLCGVLGYAAFGSDAPGNFLTGFGFYDPFWLIDVGNVCIAVHLVGAYQVFCQPIYQFVEAWARGRWPDCAFLHAELAVVAGSSFTASPFRLVWRTAYVVLTALVATVFPFFNDFLGLIGAVSFWPLTVYFPIQMYMAQAKTRRFSPAWTWMNVLSYACLFVSLLAAAGSVQGLVKDLKGYKPLFKVS*

>ZmAAAP21

MAENNVVATYYYPTAAPAAMEVCGAELGQGKPDKCFDDDGRPKRNGTMWTASAHIITAVIGSGVLSLGWAIAQLGWVAGPVVMLLFSLVTYYTSSLLADCYRSGDPSTGKRNYTYMDAVNANLSGIKVQICGFLQYANIVGVAIGYTIAASISMLAIRRANCFHQKGHGNPCKISSTPYMIIFGVAEIFFSQIPDFDQISWLSILAAVMSFTYSSIGLGLGVVQVIANRGVQGSLTGITIGVVTPMDKVWRSLQAFGDVAFAYSYSLILIEIQDTIRAPPPSESTVMKRATVVSVAVTTLFYMLCGCMGYAAFGDGAPGNLLTGFGFYEPFWLLDVANAAIVVHLVGAYQVYCQPLFAFVEKWAAQRWPDSAYITGEVEVPLPLPASRRRCCKVNLFRATWRTAFVVATTVVSMLLPFFNDVVGFLGALGFWPLTVYFPVEMYVVQKKVPRWSSRWVCLQMLSLGCLVISIAAAAGSIAGIASDLKVYRPFKSY*

>ZmAAAP60

MTQQDVEMAARHGTGADGAGFYPQPRNGAGGETLDDDGKKKRTGTVWTASAHIITAVIGSGVLSLAWSTAQLGWVVGPLTLMIFALITYYTSSLLADCYRSGDQLTGKRNYTYMDAVAAYLGRWQVLSCGVFQYVNLVGTAVGYTITASISAAAVHKANCFHNKGHAADCSTYDTMYMVVFGIVQIFFSQLPNFSDLSWLSIVAAIMSFSYSSIAVGLSLARTISGRSGTTTLTGTEIGVDVDSAQKVWLALQALGNIAFAYSYSMILIEIQDTVKSPPAENKTMKKATLMGVTTTTAFYMLAGCLGYSAFGNAAPGNILTGFGFYEPYWLIDFANVCIVVHLVGAYQVFSQPIFAALETAAAKRWPNARFVTREHPLVAGRFHVNLLRLTWRTAFVVVSTVLAIVLPFFNDILGFLGAIGFWPLTVYYPVEMYIRQRRIQKYTSRWVALQLLSFLCFLVSLASAVASIEGVTESLKHYVPFKTKS*

>ZmAAAP59

MVGAMRGGAMELEDRLATLPRFRGDHDDDGKERRTGTVWTATAHIITAVIGSGVLSLAWAMAQLGWVAGPLTLVLFAAITFYTCGLLADCYRVGDPVTGKRNYTYTEAVKSNLGGWYVWFCGFCQYVNMFGTGIGYTITASISAAAINKSNCFHWHGHDADCSQNTSAYIIGFGVVQVIFSQLHNFHKLWWLSIIAAIMSFSYSAIAVGLSLAQIVMGPTGKTTMTGTLVGVDVDAAQKVWMTFQALGNVAFAYSYAIILIEIQDTLRSPPAENKTMRRATMMGISTTTGFYMLCGCLGYAAFGNAASGNILTGFGFYEPFWLVDFANACIVVHLVGGFQVFCQPLFAAVEGAVAARYPGSTREYGAAGLNVFRLVWRTAFVAVITLLAILMPFFNSILGILGSIAFWPLTVFFPVEMYIRQRQVRRFSTKWIALQSLSFVCFLVTAASCAASVQGVVDSLKTYVPFKTRS*

>ZmAAAP09

MAVHHALEVLDGRCDDDGHPRRTGTAWTCAAHIITAVIGSGVLSLAWSVAQLGWVVGPACMFCFALVTYVSAALLADCYRRGDPGNGPRNRSYMDAVRVYLGKKHTWACGSLQYVSMYGCGVAYTITTATSIRAILKANCYHEHGHGAHCEYGGSYYMLIFGGAQLLLSFIPEFHDMAWLSIVAAVMSFSYSFIGIGLGLATTIANGTIKGSITGVRMRTPMQKVWRVSQAVGDIAFSYPYSLILLEIQDTLKSPPAENKTMKRASIGSILVTTFFYLCCGCFGYAAFGSDSPGNLLTGFGFYEPYWLIDFANACIILHLLGGYQVYSQPIFQFADRFFAERFPDSGFVNDFHTVRVACLPACRVNLLRVCFRALYVASTTAVAVAFPYFNEVLALLGALNFWPLAIYFPVEMYFVQRNVPRWSTRWVVLQTFSVVCLLVSTFALVGSIEGLITQKLG*

>ZmAAAP66

MAPAPHNGLNNNHPVAPMDVSVEAGNAGAAEWLDDDGRPRRSGTFWTASAHIITAVIGSGVLSLAWAIAQLGWVAGPAAMLLFAFVTYYTAALLAECYRTGHPETGKRNYTYMDAVRSNLGGVKVVFCGVIQYANLVGVAIGYTIASAISMKAVRRAGCFHAHGHADPCKSSSTPYMVLFGGVQILFSQIPDFDQIWWLSIVAAVMSFTYSSIGLSLGIAQTVSNGGFKGSLTGISIGAGVTSTQKIWHTLQAFGDIAFAYSFSNILIEIQDTIKAPPPSESKVMQKATRLSVATTTVFYMLCGCMGYAAFGDNAPDNLLTGFGFYEPFWLLDVANVAIVVHLVGAYQVFCQPIFAFVERRAAAAWPDSAFVSRELRVGPFSLSVFRLTWRSAFVCVTTVVAMLLPFFGDVVGLLGAVSFWPLTVYFPVEMYIKQLRVPRGSTKWICLQTLSVSCLLVSVAAAAGSIADVIAALKVYKPFSG*

>ZmAAAP36

MGRSGGGDGDGDGDRLLLGKPLESSSSCSSSDESLVKRTGTVWTAMAHIITAVIGSGVLSLAWSVAQLGWVGGPAAMVFFAGVTAVQSTLIADCYICHHPERGGVVRNRSYVDAVRIYLGDKSHLFCGFFLNLSLFGTGVVYTLTSATSMRAIRKANCYHREGHDAPCSVGGDGYYMLLFGLAQVLLSQIPNFHEMAGLSIFAAVMSCFYAFVGVGLGVAKVIANGVIMGGIGGIPLVSTTQKVWRVSQALGDILFAYPFSLVLLEIEDTLRSPPPESETMKKATRASIAITTLFYLCCGCFGYASFGDGTPGNLLTGFGFYEPYWLIDLANLAIVLHLLGGYQVYTQPVFAFADRKFGGGATVVEAPLLPVPGARRVNANVFRLCFRTAYVAATTALAVWFPYFNQIIGLLGSFTFWPLAVYFPVEMYLTRNKVAPWTNQWLAIHAFSLVCLLISAFASVGSAVGVFGSETS*

>ZmAAAP54

MEVSSVEFGHHAAAASKCFDDDGRLKRTGTMWTASAHIITAVIGSGVLSLAWAIAQLGWVAGPTVMLLFSFVTYYTSALLADCYRSGDACTGKRNYTYMDAVNANLSGVKVWFCGFLQYANIVGVAIGYTIAASISMLAIQRANCFHVEGHGDPCNISSTPYMIIFGVVQIFFSQIPDFDQISWLSILAAVMSFTYSTIGLGLGIAQVVSNKGVQGSLTGISVGLVTPVDKMWRSLQAFGDIAFAYSYSLILIEIQDTIRAPPPSESKVMRRATVVSVAVTTFFYMLCGCMGYAAFGDNAPGNLLTGFGFYEPFWLLDVANAAIAVHLVGAYQVYCQPLFAFVEKWARQRWPKSRYITGEVDVPLPLGTAGGRCYKLSLFRLTWRTAFVVATTVVSMLLPFFNDVVGLLGALGFWPLTVYFPVEMYIVQKKVPRWSTRWVCLQLLSVACLVITVASAAGSVAGIVSDLKVYKPFVTTS*

>ZmAAAP69

MALGEGGDHGAALPLIADQAKHAAAGGIVRSGSMWTAAAHVITAVIGSGVLSLAWSIAQLGWVAGPAAMLVFAAVTALQSTLFADCYRSPDPEHGPHRNRTYAKAVDRNLGSNSSWVCMLLQHTALFGYGIAYTITASISCRAILKANCYHEHGHDAHCDYDGNYYMLIFGGVQLLLSFIPDFHDMAWLSVVAAAMSFSYAFIGLGLGLARTIANGTIKGSITGVRMRTPMQKVWRVSQAIGDIAFAYPYSLILLEIQDTLKSPPAENKTMKRASMISILVTTFFYLCCGCLGYAAFGSDAPGNLLTGFGLYGPYWLIDFANACIILHLLGGYQVYSQPIFQFAERLLAERFPDSGFVNGGSYTVRFACLRACRVNPLRVCLRTLYVASTTAVAVALPYFNEVLALLGALSFWPLAIYFPVEMYFIQRNVRRWSARWVVLQTFSVVCLLVSAFALVGSIEGLISKKLG*

>ZmAAAP29

MEVAGNHVQSCRTELPEPQKPLVDDDGRPLRTGTLWTASAHIITAVIGSGVLSLAWGVAQLGWAGGPAAMVLFAAVIYYTSTLLAECYRCGDPTFGPRNRTYIDAVRATLGDSKERLCGAIQLSNLFGIGIGVSIAASVSMQAIRRAGCFHYRGHEDPCHASTSPYIAVFGVMQIVFSQIPDLDKVWWLSTVAAIMSFSYSTIGILLGVVQIVEHGGPRGSLAGVIGAGARVTMMQKVWRSLQAFGNIAFAYGFSIILLEIQDTIKSPPPSEAKVMKKATAVSVAVTTVIYLLCGCVGYAAFGGAAPDNLLTGFGFYEPFWLLDVANAFVVVHLVGTYQVMSQPVFAYVERRAAAAWPGSALVRDRHVRVGRAVAFSVSPARLAWRTAYVCVTTAVAMLLPFFGSVVGLIGAASFWPLTVYFPVEMYIAQHRVARGSMRWLLLQGLSAGCLVVSVAAAAGSIAGVVEDLKAHNPFCWSC*

>ZmAAAP46

MRDGGGAMDVDMQARGGGASHGGELDDDGKEKRTGTVWTASAHIITAVIGSGVLSLAWAMAQLGWVAGPVILLLFAAITYYTSCLLTDCYRFGDPVTGKRNYTYTEAVESYLGGRYVWFCGFCQYANMFGTGIGYTITASASAAAILKSNCFHWHGHDADCTQNTGSYIVGFGVVQVIFSQLSNFHELWWLSVLAAAMSFCYSTIAVGLALGQTISGPTGKTTLYGTQVGVDVGSAEEKIWLTFQALGNIAFAYSYTIVLIEIQDTLRSPPAENKTMRQASVLGVATTTAFYMLCGCLGYSAFGNAAPGDILSGFYEPYWLVDFANVCIVIHLVGGFQVFLQPLFAAVEADVAARWPACSARERRGGVDVFRLLWRTAFVALITLCAVLLPFFNSILGILGSIGFWPLTVFFPVEMYIRQQQIPRFSATWLALQALSIFCFVITVAAGAASVQGVRDSLKTYVPFQTRS*

>ZmAAAP33

MVASKAAPFDEVSSVEAGAYGGRDDDGRPRRTGTVWTASAHIITAVIGSGVLSLAWAIAQLGWAAGPAVMLLFAVVIYYTSTLLAECYRSGDPVAGKRNYTYMDAVRASLGGAKVRLCGAIQYANLFGVAIGYTIAASISMLAIKRADCFHAKGHKHACRSSSNPYMILFGVAEVVFSQIPDFDQIWWLSIVAAVMSFTYATIGLVLGIMQTVANGGFQGSLTGISIGAGVTPTEKVWRSLQAFGNIAFAYSYSIILIEIQDTVKAPPPSEAKVMKRATMVSVATTTVFYMLCGCMGYAAFGDAAPDNLLTGFGFYEPFWLLDIANVAIVVHLVGAYQVFCQPLFAFVEKWAAATWPDSAFIAREFRVGPFALSLFRLTWRTAFVCLTTVAAMLLPFFGDVVGLLGAVSFWPLTVYFPIEMYVVQRAVRRWSTHWICLQMLSAACLLVSVAAAAGSIADVIGALKVYRPFSG*

>ZmAAAP67

MEPNLQSALEEQTKILRALSARLAAQEARWRSWESKVAHHSVSIHDLEVAVATVPSATLRSERDAQVAATRECLAVVADDWGGLFGTGDGCIVADNWGGLFEYPAHFQEEQDIDNCIPINDGTRFTEEDDDNHPATLEPLLTDTTTAAEVDSIANTDDVEADSLASASTVGVATSQAAEMQLHVGWARWLEGQADYLRHDRMLHSNLDSLTSSNHPLLQDASATNNSPTQYSTECFSHDIVLLRTISAAPTSEDRALQQRRLECLVAWPEWRRIGGRNYIMLEHHPNRMFDACDRFWHCVFALYDFGSVQGLQRKLERLCLAGSAPDLEVLAVLSGLRARSLSDQRAANHTRTSMRSISTTATPFRLCSTRSGYIASGTSTGTTCSTRSQSWISSEFEHSSIPTPNQKVFNLSSDVLNKRDPWVGTFDHIFTELGQPQSLEAVRLLCHLRHQLPDCSQLEFTYEVFAAQKVFEELSLSSPATWVNIGKLLASLCWKIPWPPPHIQVGLGCGSEALRSLPWLSPTIASTNEERIFIISEDGKVDGVVDQTMDEQIAAAVFMPACIEASLNDGMNAGKTREAFADHQYSEYDSRLKDKDQKILFEDEIDLSLFNYKFDSYTTDSPAIDAEVELRSTDLWPYWKVDCLCKIIPRQLLAENAMMLIRNTVDKLIMKHKEIQVETEQIAGEDDVNEGDPSILRFLLASRDKSADMERSQELLETLVILIVKSVKDPRSVVCKTALMTCADIFKAYGALMVHSIDPSLVQQLLLTASQDKRFVWETATTTFISLTSWIFPLLLKPTMLPYLKNKNTGTWPKASRLDDDGRPRRKGTFWTASAHIITAVIGSGVLSLAWAIAQLGWVAGPAALLLFAFVTYYTATLLAECYRTGDPDTGKRNYTYMDAVRSNLGGTKVLLCGVIQYANLVGVAIGYTIASSISMKAVRRAGCFHVHGHGDPCRSSSTPYMILFGLVQILFSQIPDFDEIWWLSIVAAVMSFTYSSIGLSLGIVQTISNGGFMGSLTSISFGAGVSSTQKVWHTLQAFGDIAFAYSFSNILIEIQDTIKAPPPSESKVMQKATCVSVATTTIFYMLCGCMGYAAFGDNAPDNLLTGFGFYEPFWLLDVANVAIVVHLVGAYQVFCQPIFAFVERRAAAAWPDSAFISRELRVGPFALSLFRLTWRSSFVCVTTVVAMLLPFFGDVVGLLGAVSFWPLTVYFPVEMYIKHRRVPRGSTRWICLQTLSVTCLLVSIAAAAGSIADVIDALKVYRPFSG*

>ZmAAAP65

MASHNGTKHLAPMEVSVEAGNGGGAEWLDDDGRPRRTGTFWTASAHIITAVIGSGVLSLAWAIAQLGWVAGPAAMLLFAFVTYYTATLLAECYRTGDPDTGKRNYTYMDAVRSNLGGAKVAFCGAIQYANLVGVAIGYTIASSISMQAVSRAGCFHKRGHAVPCKSSSNPYMILFGAVQILFSQIPDFDQIWWLSIVAAVMSFTYSAIGLSLGIAQTVANGGFKGSLTGISIGADVTSTQKVWHSLQAFGDIAFAYSFSNILIEIQDTIKAPPPSESKVMQKATRLSVATTTIFYMLCGCMGYAAFGDKAPDNLLTGFGFFEPFWLIDVANVAIVVHLVGAYQVFCQPIFAFVERRAAAAWPDSAFVSRELRVGPLALSVFRLTWRSAFVCVTTVVAMLLPFFGNVVGFLGAVSFWPLTVYFPVEMYIKQRRVPRGSTKWVCLQTLSVACLVVSIAAAAGSIADVIEALKVYHPFSS*

>ZmAAAP22

MDKSAVAAYDVERGDYEEEHERRGTVWTATAHIVTAVIGSGVLALAWSVAQLGWVAGTLALAGFACVTYYTSTLLANAYRAPHPVTGDRNRTYMDAVRSYLSPREVFMCGIAQYVNLWGTMVGYTITATISMAAIRQSNCFRRSGAGAHCDAPGTVLMLAFGVVQVVLSQFPGLEHITWLSVVAAVMSFAYSFIGLGLSVGQWVSHGGGLGGRIAGAAAASPTRKLWNVLLALGNIAFAYTFAEVLIEIQDTLKSPPPENRTMKKAAMYGIGATTIFYISVGCAGYAAFGSDAPGNILTAGGLGPFWLVDIANMCLILHLIGAYQVYAQPIFASVERWAASRWPEAKFISSAYTVSIPLMQRGSVTVAPYKLVLRTVLVAATTVVALMIPFFNAVLGLLGAFSFWPLTVYFPISMHIAQDKITRGTKWYLLQALSMVCLMISVAVGIGSVTDIVDSLKVSSNPLKTVS*

CDS Sequences

>55902

ATGACAGCGGCTTCTTCAACCTCTGATGTAACTAGACTTGTAGATCAGCCCCTCTCATTTGAGCTTGAAAGGCAGAACGG

GCATGCGTCAACATCAGGCTCTACAGCACCACAGAGCAAATGGTACGATGCAACTTTTCACACGATCACCGCAGTAGTTG

GTGTGGGGGTGCTATCGCTTCCATATGCCTTCTCCTACCTCACGTGGACTGGCGGTGTAATCGCCCTGGCGGTGACAACA

GCAACCAGCCTCTACACCGGGTACCTGCTAGCAGCACTGCACGAGGACAAAAACGGCCACAGGCATAATAGATACAGGGA

TCTGGGGAGGGCTATCTTTGGGGAGAAGTGGGGCAATTGGGCGATTGCCCCATTCCAGTGGTCTGTGCTGGTGGGCCTGG

CCATCACATACACTGCTACTGCAGGCCAGAGCCTCCAGGCAGTGCACAGCAGCACGTGCAACAACGCTGTGTACAAGGCG

GTTGGAGCGGGGCGGACTGATAGGAACTGTTCTAGTGCATTGGCCTGGTGGACGATTGTCTTCTCCTTCTTCGAGCTATT

TCTGTCCCAGATAAAGGACTTCCACTCCCTCTGGTGGGTATCGCTGCTGGGGGCGGCCATGTCAGCGATGTACTCAACGC

TGGCATTTGCAACATCGGTGGCAGCTGGGTCGGAGGGCGCCAGCTACGGGCCACGCCAGGAGAGCCCGGCCGCACTCATA

CTGGGAGCGTTCAATGCGCTGGGCACCATCATGTTTGCCTTTGGCGGCCATGCCATCCTGCTGGAGGTGCAGGCAACGAT

GCAGACGCCGCCGTCAGCGCTCAAGTCGATGATGCGCGGCCTGGGTGCGGCCTACACGGTGGTAGTGATCGCGTACTTCC

CTGTGGCGAGCGCGGGGTATGCGGCGTTTGGGAATGTGGTGTCCCCGGACGTGCTGCTGTCCGTGCGCAAGCCTGCCTGG

CTCATCAGCATCGCCAATTTCATGGTGGTCATCCACCTGGCAGCCTCCTACCAGGTGTTTGCGCAGCCAATATTCGAGAC

GGCGGAGGGCTGGCTGGCGGCGCGCAAGCACCGGCTGGTGGATCGGCCGATAGTGACGCGCGCGATCGTGCGCTGCAGCT

ACGTGGCACTCACCTGTTTCGCGGCCATCCTCATCCCATTCTTCGGCGACCTCATGGGGCTGGTGGGTTCGTTGGGACTG

ATGCCGCTCACTTTCATTCTGCCGCCCGCCCTTTGGATCAAGGCGACAAAGCCCAAGGGCCCGGAGCTGTGGTTCAATGT

GGCGCTCATGGTGGTGTATGGGGTTGCGGGAGTGCTGGCAGCCATCGGATCTGTGTACAACATCGTTGTGCACGCACATG

AGTATCACACTGTGGGCTGA

>24967

ATGGCGCTGGGCGAGAAGACCGGCATGGAAGACTCGGCACACGCCAACAAAGTCAACTTTTCGAAAGACCCGGAGGGCCA

GATGGAACTCGACGATAAGCAGACCGTGCCAGAATATGTTGGCAAGGGCGAGTGGTACCACATCGGCTACCACATGACCG

CGGCTGTGGCCTCTGTGCCCACCCTTGGCCTGCCCTTTGCTGTGTCTCTCCTCGGCTGGGGTGGAGGCCTGGTTGCGCTG

ATTGCTGGGGGCCTGGTGACCATGTTCACCTCCTTCCTGGTCTCTAGCATGCTTGAGTATGGCGGCAAACGCCACATCCG

CTTCCGCGACCTGTCTGTTGCTGTCTTTGGCAAGTCCGGCTGGTGGGCTGTGACCCCCTTCCAATTCGCGGTTTGCATCG

GGACCACCATTGCCAACCACATCGTGGGGGGGCAGGCCATCAAGGCCATAGACGTGCTGGCGCGGGGGGAGACACCGGTG

ACACTGACGCAGTACATTTTGGTGTTTGGAGCGGTCAACCTGATCCTTGCCCAGTGCCCCAACTTCCACTCCATCCGCTT

TGTCAACCAGACCGCCACTGTCTGCACAATCTCATTCTCAATCATCGCAGTCGCACTCTCTCTGTACTCAGGTTTCACAA

TGGATCTGCAGCCAGACTACACAGTCCCAGGAGAAGGGGTGAACAAGCTCTTCAACATCTTCAATGGCCTGGGTATCATG

GCTTTTGCTTATGGAAACACCGTCATTCCAGAGATCGGTGCAACCGCCAAGGCGCCAGCCATGCGGACAATGAAGGGTGG

CATCATTATGGGCTACTGCACCATTGTGTCTGCCTACCTCTGTGTCTCCATTACTGGGTACTGGGCCTTTGGCAACGGCG

TCAAGGGACTTGTTCTGGGCAGTCTCACAAACCCGGGCTGGGCTGTCATCATGGCCTGGGCCTTTGCTGCCGTGCAGCTC

TTCGGCACGACCCAGGTGTACTGCCAGCCTATCTACGAGGCCTGCGACAAGACCTTTGGCAATATCCTGGCGCCCACATG

GAACCTTAAGAACACAATTGTCCGCCTTATCTGCCGCACTGTCTTCATCTGCCTCTGCATCCTGGTTGGCGCAATGTTGC

CCTTCTTTGTGGACTTCATGAGTCTGATCGGTGCAATTGGCTTCACTCCTATGGACTTTGTGCTACCCCAATTCCTGTGG

ATCAAGGCCTACAAGCCAAAGGGATTCTCAAAGTGGTTCTCCCTCCTGGTCGCTATCATCTACATCATTGTGGGCATCAT

GGCATGCATCGGTGCTGTCAGGAGCATTGTCCTGAACGCAGTCAACTACAGCCTCTTTGCCAACCTGTGA

>31400

ATGGCCGACGTCGAGGGTAAAGCTGCACCAGACACCGGCCATGAGAATGGAAATGCCAAAGAACCGCTGGGCCATCTCAA

CAAGTACGATCAGGAATATAAACTTCCGATTACGGGAGACCGCACAGGGAAATGGTGGTATTCTGCCTTCCACAATGTTA

CAGCGATGGTCGGCGCTGGAGTGCTGGGCCTTCCTTCAGCTATGGCGTACCTGGGATGGGGCGGTGGCATGTTCATCATG

GTGTCCTCCTGGATCATCACGCTGTACACGCTGTGGCAGCTGTGCTCCATGCACGAGATGAACGGCAAACGCTTCAACCG

CTACCACGAATTAGGCCAGTACGCTTTCGGGCAGAAGAGAGGCCTGTGGTTTGTCATCCCCTTCCAACTCATCGTCATGA

TTGGGCTGGCCATCGTGTACTGTGTCACCGGAGGCAAGAGCATGCAGGCTGTGTGGCAGTTCTTGTGCAACAAGCCCTGC

CCGGCATTTGGGCTGTCTGCATGGATCGTTGTGTTTGCCGGCGCACAGCTCTTCCTCTCTCAGTGCCCCAACTTCAACAG

CCTGCGTGTGGTGTCCTTCGCGGCAGCAATCATGTCACTCGCATACAGCACGATCGCTGTGGGGGCATCCATTGCCTCTG

GCCGTCAGCCTGACGCTTACTACAACCTTGACACAAAGGACACTGCCGACAAGGTGTTCGGTGTCTTCAGTGCCCTGGGC

ACTGTTGCCTTTGCGTATGGAGGACACAATGTTGTGCTTGAGATCCAGGCAACGCTGCCCTCTCCGCCCGACACCTTCAA

GCCCATGATGGCTGGTGTCTACGTCGCCTATGCACTTGTTGCTTGGTGCTACTTTGCTGTTTCCATTACTGGGTACTGGG

CGTTTGGTATCAACGTCGCAGACAACGTGCTCCTGACCAGTGCTCTGAAAGACACCGTTCCCAACGGACTCATCATTGCT

GCTGACCTCTTTGTGGTCATCCACGTCATCGGCAGTTTCCAAGTGTACTCCATGCCGGTATTTGACATGATTGAGACCAG

GATGGTGATGTCAGGCATCAGCAATGCCCTGCCCATGCGCCTGTTGTACCGCAGCGTCTATGTCATCATCGTGGCATTCG

TGGCCATCGTGCTTCCCTTCTTTGGGGATCTCCTGGGCTTTATTGGAGCCTTCGCCTTTGGCCCCACAACGTTCTGGATG

CCCCCCATCATATACCTGATTGTGAAGAAGCCCAAGATCAACAGCGGCCACTGGTGGGCCAGCTGGTTCTGCATCATCTA

CGGCCTCATCGTCACCATCTTCGGTTCCATCGGAGGCATGCGTGGCATCATCAAGAGCGCCAGCACCTACAAGTTCTTCC

AGTGA

>36205

ATGGAGAATGGCGAGCCTCACAATCCTAAACTTTTGGTAGATGAGAAGGGTTTCGCCCGCTCAGATCTCGAGAAGTACGA

TGACGACGGTCATGTTGCCAGGACAGGTGGCTGGATCACGGCCTACGCCCACATCGTGTGCGCTGTCATTGGCAGCGGCG

TCCTGTCCCTCGCCTGGGGAGTCTCCTGGCTTGGCTGGGTGGCGGGCCCCATCGTGCTCTTCATGTTTGCATGGATCACC

TGGTACTGCTCAGCGCTGCTCATCGACTGTTACCGCTTCCCGGACGTTGATGGGGAGAAGAGGAATTATACCTACATCCA

GGCAGTCAAGCGCTACCTAGATGCCAACATGGTGGGGACTTCCGTCGGCTACACAGTGACTGCTGGGATTGCTGCCACGG

CCATTCGTCGCTCCGACTGCTTTCACGCTGACATCAGCAACCCCTGCGAAATCTCCAACAATCCCTGGATAATCCTGTTC

GGCGCGCTCCAGATCCTCTTCTCTCAGATCCAGGACATTGACCGCATCTGGTGGCTGAGTATTGTCGCCACTCTGATGAG

CTTCACTTATGCCTTCATCGGCCTCGGGGAGTGCATTGCCCAAGCCGCACAGGGGAGCACCACTGGCACGGGCACTGTTG

GGGGCCTCCAGATTGGCATTGACACCACCGCCGCAGGGAAAGTCTGGGGCATCTTCCAGGCGCTTGGTAACATTGCCTTC

GCGTACAGCTTCAGCTTCATTCTGATTGAGATCACGGACACAATCCAGTCACCAGGCGAGACCAAGAAGATGAGGAGGGC

GACAGTGTATGGCATCGCCACGACAACTTTCTTCTACGCATGCATCGGCATCATCGGCTATGCCGCGTTTGGTAACAGCG

CGCCAGGAAACCTGCTCAGCGGGTTTGGCTTTTACAATCCATGGTGGCTTATTGATATCGCCAACGCAGCCATCTTTGTG

CATCTCCTCGGAGGCTACCAGGTGTGGATCCAGCCATTCTTCGGCTTTGTGGAAGCATCAGCTTTCAGATATTTCCCAAA

GAGCAGGTTCCTGCAGTGGGAGCTCTTTGCTGTTGAGATTCCCGGCATGGGCCTGTTCAGGGCGAGCCCCTTCCGCCTGA

TCTGGCGCACCGTCTACGTCATCATCGTCACAATTGTGGCACTGCTGCTCCCCTTCTTCAATGACATTGTCGGCCTTCTT

GGTGCAATCGGCTTTGCTCCACTGACTGTCTTCTTCCCGATCCAGATGCACATTGTGCAGAAGAAGATTCCCATGTGGTC

TGGGCGGTGGTGCTTCCTGCAGGGCCTCAATGTCCTCTGCTGGCTCATCTCCATCGCCGCCGCCATCGGCTCTGTGGAAG

GAATCTATGCAGACACTCGCAACTACACCCCCTTCCAGACCTCCTACCGCCGCTGA

>29311

ATGTCGAAGCTCAACGACAGTGCACACGGAAATGACTTGGAGAGGAAGCCTTCAAAAGTTGAGGAGGGAGTGATCGGCAA

GCACGGATCAACGTCTCCCCTGATCGAGATCACGCACAATGACAAGTGGTACCACGCTGGCGGCCACATTTGCACCATCA

TCGCAACACCAGCAGCATACGCGCCGCTACCCTTTGCCTTCGCGCACTTGGGCTGGGAGGCCGGAGTCATCTTTCTGCTG

CTGGCCGGGTTAGTCACCTGGTACACATCCCTGCTGCTAGCTTCCCTGGACCGCCACGACGGCAAGCGTCACACGCGCTA

CTGCGACCTGGCAGGCTCCATCTACGGCAAGGGCGGCTACTGGTCGGTCATCTTCTTCCAGCAGCTGGCGTCCATTGGCA

ACAACCTGACCATCCAGATCGTGGCTGGCCAGTGCCTCAAGGCACTGTACCGCCTCTACCACCCGGAGTGTGAGCCAACC

GGCGCCTGCGGAATCTCCCTGCAGGCCTGGATTGCAGTCTTCGGCGCCTCCCAGCTCATTCTGTCCCAGCTGCCAGACAT

CTCCTCCCTAAGGGAGATCAACCTCGTGTGCACCCTCTGCACCGTCTGCTTCGCTGTCGGCTGCCTGGCCATGTCAATCT

ACAACGGAAACACGCAGGTGGACCGTTCCACAGTCAGCTACGATGTGCAGGGAGATGCGAAGCCCAAGATCTTCAACATC

ATGTTCTCCCTGGGCATCATTGCCTTTGCGTTCGGAGACACCATCCTGCCAGAGGTCCAGGCCACTGTTGGGGGTGACTC

CAAGAAGGTGATGTACAAAGGAGTCAGCTGCGGCTACGCTATCCTGCTGTCATCCTACATGGTTGTCGCCATCGCCGGCT

ACTGGGCGTTCGGCTTTGATGTGTCGCCGTTCGTGGTGTTCTCCTTCAAGGAGCCCTCCGGCATGTTAGCAGCGCTGTAC

ATCTTTGCCGTGCTGCAGATCATTGGCTGCTACCAGATCTATGCAAGGCCCACCTTTGGATTTGCCTACAACTACATGCT

GAGGCCCTACGAGGGCGTCTGGTCATTCCACAACGTTCTGATGAGGGCCATTGTGACTACCATCTACATGGCCATCATCA

CCCTTATTGCTGCCATGATCCCCTTCTTCGGGGACTTTGTCGCGTTTGTGGGAGCTATCGGTTTCACCCCAATGGACTTC

ATTCTGCCGATCATCCTGTGGCAGAAAGTGGGCAAGCACTCGCTCATCGTCAGCATTGTCAACTGGTGCATCGTGGTCTT

CTACAGCATCATTGCCATTGCAGGCGCAATCGGATCCATCCAGGCCATCAATGCCGACCTTGCAAACTTTAACGTCTTTG

CAGACCTGTTCTGA

>Sphfalx0000s0509

ATGAAGTTTGAAGGAGCTGCAGGTGGCGATGAGAACCTGGGACGGGCAGAGAATGGAAATGCAGCAGCTGCCGACCAGTT

CAACAAGAGAAACCACGCTCTCAATCATATTCCCACCTCCAAACAGCTGGACGACGATGGCAAAACCAGACGCAGAGGGA

CGGTGTGGACAGCATCAGCACATGTGATAACAGCAGTCATAGGCTCAGGGGTGTTGTCTCTGGCCTGGAGTGTGGCACAG

ATGGGCTGGGTCGTGGGTCCCACTGTGCTCCTCCTCTTTGCCCTGGTCACATACTACACTGCCCTGCTGCTCACAGACTG

CTATAGGTACCCTGACCCTGTCTCGGGCAAGAGGAACTATACTTACATGGATGCTGTGCAAGCAAACCTGGGGCCAAGGC

AAGTGTTCATCTGTGGGCTAGTACAGTACTCCAATTTGTTGGGGACTGCCATTGGGTACACCATCACTGCGACAATAAGC

ATGGTAGCTATAAAGAGGTCAGACTGCTTTCATGCAGATGGAGATAGTGCACCATGTCGGGAATCCAATTTCATATACAT

GGTGTTCTTCGGGATAGTGCAGGTCATATTGTCACAGATCCCTAACTTTGATCGACTCTGGTGGCTCTCCATAGTAGCTG

CAATAATGTCTTTCTCGTATTCCACAATTGGCCTTGGCCTCGGCTTAGGAAAAGCTTCAGAAGGAGATCATTCTCATGGC

ACTTTGACTGGGGTTGAAGTTGGTGATCATAGTCTTGGCTTTGCAACACGAGCTCAAAAGATCTGGGATGTTTGCAATGC

GCTTGGCAACATTGCCTTTGCATATTCCTTCTCCATGATCCTTATTGAAATCCAGGACACACTGAAAGCTCCACCTGCTG

AGAATAAAACAATGAAGAGGGCAACTCTTATTGGGATACTCACCACCACAATATTCTACATGAGTGTGGGCTGTGTGGGT

TATGCTGCCTTTGGGGATGCTGCCCCTGGCAATCTCCTCACTGGCTTTGGGTTCTACAATCCCTATTGGCTTGTTGACTT

TGCCAATGCTTGCATTGTTGTCCATCTTGTCGGTGCTTATCAGGTTTATACCCAGCCACTTTTTGCATTTGTGGAGGATT

CAATGTCCCGAAAATTCCCCAAGAGCAACTTCATTCACAAGGAGCTTGAAATGAATCTCCCATTGGGGGGACCATTACAT

ATCAACCTATTCCGCCTTGTCTGGCGCACTTCTTTTGTCACATTCACAACACTGGTGTCCCTTATGGTTCCCTTCTTCAA

TGACGTACTGGGATTAATTGGTGCATGCGCATTTTGGCCACTTACAGTATACTTCCCAGTTCAAATGTACATTATGCAAC

AGAGCATACAGAGATGGAGCTCAACATGGCTTGCACTCCAAACCTTAAACACGGTTTGCTTCTTTGTATCCCTAGCTGCA

GCTGTTGGATCCATTGCTGGAATTCTCACAGACGTGAAACATTACACCCCATTCAAATCCTAG

>Sphfalx0002s0399

ATGTTTTCCAAAGACTCCAAAGGCCCCTTGGCATCTGCCACTACCGATGAAGATTTCCAGGCATATGTGGAGGAAATTGA

GCCTGCAAAACCAGTCAATGTTGAGGATTGGCTGCCAGTGACTGGGAGCAGAAATGCCAAATGGTGGTATGCAGCATTCC

ATAATGTGACAGCTATGGTGGGTGCGGGAGTTCTTGGCCTGCCTTATGCTATGGTTTACCTTACCTGGGGTCCTGGAGTG

ATAGTGTTGGTGTTATCGTGGGTCATCACACTCTACACCTTGTGGCAAATGGTGGAGATGCATGAGATGGTGGAAGGCAA

GCGCTTTGATCGCTACCATGAATTGGGTCAGGAAGCGTTTGGGGAGAAATTAGGACTCTGGATTGTGGTACCTCAGCAGG

TCATCGTGGAGGTAGGCGTGGACATTGTGTATATGGTAACTGGTGGCAAATCCTTGGAGAAGTTCTATGAGCTTACTTGC

AAGAAGAATTGCTACTTGCAAAATCGACTTTCTATCTGGATTCTTGTGTTTGGGTCTGTGCACTTGTTTTTGGCTCAACT

ACCGAACTTCAACTCCATTGCGGGAATTTCATTAGCTGCAGCTATCATGTCACTCAGCTACTCAACAATTGCATGGGCCA

TCCCTGCCCATTATGGACACTCCTTGCTTCCTCCCGGGCAGAGGCCAGACTATCATTTGCCACCAAATCAGTCAACAGCA

GCAATGGTCTTTGGTGCTTTCAATGCATTGGGAACAGTAGCATTTGCATATGCTGGCCACAACGTCGTGCTGGAGATACA

AGCCACAATCCCATCAACTTCTCATCGGCCATCAAGAATTGCCATGTGGAGAGGAGTCCTGGTTGCTTATGGAATTGTGG

CTGCATGCTACTTTCCTGTTGCTTTTGTTGGTTATTGGGCGTTCGGTAACCAGGTACAAGACAACATCATCACGTTTGTG

GCCAAACCTGTGTGGCTGGTTGCCATTGCCAATCTCATGGTTGTCATTCATGTCATCGGAAGTTATCAGATCTATGCAAT

GCCAGTGTTTGATATGCTGGAAACACTGCTTGTGAAGAGATTTCGTTTTTCTCCTTCTCTCCGACTTCGGCTCATTACTC

GCTCCATCTATGTTGGTTTGACAATGTTTGTTGGCATAACCCTTCCGTTTTTTGGTGCCTTGCTGGGATTCTTTGGAGGG

TTTGCCTTTGCCCCAACAACATACTTTTTGCCTTGCTGTATGTGGCTGACTATCAAAAAGCCAAGAATTGGAAGCCTATC

GTGGCTTGCTAATTGGGTGTGTATCATACTAGGTGTCATGCTAATGCTTGTATCATCCATTGGTGGACTTCGACAAATTA

TCGTTGATGCCTCCAGCTACAAGTTCTATCAATGA

>Sphfalx0003s0314

ATGATGGAGGAAACTGGGAACGGCATGCAGATGGCGGAATTGACAACCAAGTACACACCATTAGATGGCTCAGGAGGTTC

TTCATCACACTCACAAGAGGACGAGGAGCAGCAGCAGCAGCGGCGGCGGCCATATTCAAGCTTGAGCGATGCTGCAGATG

CTCCCAAAGACGCCGGGACTCTGTTTGTTCTTGAATCCAAAGGGACATGGATGCATGCAGGGTATCATTTGACAACGGCC

ATCGCCGGTCCATCGCTGCTCTCGTTGCCGTATGCATTCTCCTTTCTGGGATGGGCACCCGGATTGCTGGCGCTGACAAT

CTGTGGCCTCGTTTCAAGCTATGCCTACTGCTTGCTGTCGCAAGTCTTGGACGATTGTGCTTCCAAAGGCCATCGCTTCT

ACCGATTCCGAGAACTCTCCCAGTTCGTCATCGGAAAATCTTGGACAAACTGCTTTGTGACACCGGTGCAATTTGGAGTG

TGTTTTGTGACTGTTGTTGGTGCTATTTTAGCTGGTGGCTTTGCGGTGAAGCTTATATACCTGGGAGTGAATGCGAATGG

AACGATCCCATTGGCAGCATTTGTGGCAATGTTTGGGGCAGTGATGATAGTTCTTGCACAGCTACCATCATTTCATTCCT

TGCGGTATATAAATCTGGTGTCTCTATTACTTTGCTTGACCTACAGCCTTTGTGCTACTGCTGGCTCTGTTTTGGCAGGC

TACAATAAGAATGTACCACCTAAAGATTACTCTGTGGTTGGAAATCCTGCGGAGAAAATGTTTGGGGTTTTCACTGCACT

TTCTGTCATGGCGGGAGTTTATGGAGTGGCTATTATCCCTGAAATTCAGGCAACAATGGCTCCTCCAATTGTTGGGAAAA

TGGTGAAGGGCATTGCTTTGTGCTACGTTGTGGTGGCAGCCACATTTTATTCGGTGTCCATTGCTGGGTACTGGGCTTTT

GGGAATGGTGCACAGGGGAATATATTTGATAACTTGGTACCTAGTGGTGGACCTCAATTGAATCCAGTTTGGCTCACAGC

TATTTCAAGCTTTGCAATCGTTGCTCAACTACTTGCTATTGGCCTTGTGTATCTTCAGCCAACATTTGATGTGTTGGAAA

CATTGACGGCTGATGTAAATCGTGGGAAATATGCTTTGCGAAATGTGGTTCCACGCTTGGTGCTGCGCTCCACTTATGTT

TCCTTGGCAACACTAATTGGGGCCATGCTCCCATTTTTTGGGGACATTGTTTCTTTGGTTGGAGCGTTTGGATATACACC

ATTGGATTTTGTCCTGCCCATGCTTTTCTACCAACTTGTCTTCAAGCCCTCAAGAACAACGTATATATTTTGGCTCAATT

GGGTCATCATAGTAAGTTTTTCAATAGTAGGGGTCATTGGATGCATAGCCACAATGCGTCATATTGTGATTGATGCCAAA

ACCTACAAATTGTTTGCTGATGTGTAA

>Sphfalx0005s0083

ATGGAGGGTCATGTTCCGCTTAGTGAGGATCTGGATTGGAATCGCAGGCTGAATGACGTGCTGCCTGTGACTGCGAGCAG

GAACGCCAAATGGTATTACTCCATCTTCCATAATGTAACGGCTATAGTGAGTGCTGGAGTTCTTGGCCTGCCCACTGCCA

TGGCAGATCTCACCTGGGGTCCAGGCATAGTCCTCTTGATCCTATCATGGGTCATCACCCTCTTCACCTTGTGGCAAATG

GTAGAGATGCATGAGATGTTCCCCGGCAGACGTTTTGACAGGTACCATGAACTGGGCCAGGAAGCGTTTGGGAAGAGGTT

GGGACGGTGGATCGTGATACCACAACAGCTTCTTGTCCAGGTCAGCGTGGACATCGTGTACATGGTGGCCGGAGGCCAAG

CGTTGAAAAACATCTACATGTTGAACTGCCATGGATGCAGTTCGAAAAATGTGGGGAGCGATGATATTGCTGAAAAACAA

TACGAGTCGGCTTCCCTCTGGATTCTTATCTATGGCTCCGTGCACTTGCTACTCGTCCATCTCCCGAACTTGAACTCCAT

TGCTGCACTCTCTTTGGCTGCAGCCATCATGTCAGTCAGCTACTCAACAATTGCGTGGGCCATACCCGTAAACAAGGGAC

ATCACCAACCTCAGGACTACCATTTGCCATATTACCCTGAAATTAGTCCAGCACCCGGGCCCGAGCCTCCGGCAACGGCA

AATGCCCATACAGCACATCAAGTACTGAGTATTTTCAATGCGTTAGGGGTGATAGCATTTGCGTATGCAGGACACAACGT

GGTTTTGGAGATTCAAGCCACGCTTCCCTCAACTCCCGAAAGGCCTTCCAAAATCGCCATGTGGAGAGGGGTCGTCTGGG

CTTACGTGATTGTGGCCGCTTGCTACTTCCCAGTGGCTATCATTTGTTACTGGGCTTATGGAAACCAGCTTGCAGCCTAT

TCCAATATCCTTCAATTCGAAGGCATGCTCCGACACAATTATAAGGGGATCCTCACTGCAGCTAATGTCATGTTGATTAT

CCACATTCTCGGAAGTTATCAGATCTATGCTATGCCAGTATTTGACATGCTGGAGACGGCGCTGGCGAAGAAATGGCTTC

TTCCTCCTACCTTGAAACTTCGTCTGATCACTCGCACTACTTATGTCGGTTTTACAATGTTTGTGGCTACAATCTTCCCA

TTTTTCAAAGCGTTGCTTGGATTCTTTGGAGGTTTCGCCTTTGCTCCAACAACATACTTTTTACCTTGTTGTATATGGTT

GATTGTCTGTAAGCCCAAACGATTCAGCATGTCATGGACAATAAACTGGATTTGCATCATATTGGGGGTCCTACTCATGT

TTACTGCAACCATTGGTGGCTGCTGGGCACTCGTGAACGAATGGAACAGCTACCAGTTTAGGAGGTTTTGGAAATGGCAA

GATTGTCCAGGAGTAAACTCAGCTCAATATTGTACTGCTCCAACCCCAGCTCCACACTAA

>Sphfalx0005s0085

ATGGAGGGTCATGTTCCGCTTAGTGAGGATGTGGATTACAATGGCAAGCTGAATGACTTGCTGCCTGTCACTGCGACCAG

GGAAGCCAAATGGTATTACTCCACCTTCCATAATGTGACGGCTATAGTGAGTGCTGGAGTTCTTGGCCTGCCCACTGCCA

TGGCAGATCTCACCTGGGGTCCAGGCATAGTCCTCTTGATCCTATCATGGGTCATCACCCTCTTCACCTTGTGGCAAATG

GTAGAGATGCATGAGATCGTGCCCGGGAAACGTTTTGACAGGTACCATGAACTGGGCCAGGAAGCGTTTGGGAAGAGGTT

GGGACTGTGGATCGTGTTACCACAACAGCTTCTTGTCCAGGTAAGCGTGGACATCGTGTACATGGTGGTCGGAGGCCAAG

CGCTGAAAAACATTTACATGTTGAACTGCCCTGGATGCAGTTCGAAAAATGTGGGGAGCGATGATATTGCTGAAATACAA

TACGAGTCGGTTTCCCTCTGGATTCTTATCTATGGCTCAGTGCACTTGCTACTCGTCCATCTCCCGAACTTGAACTCCAT

TGCTGCACTCTCTTTGGCTGCAGCCATCATGTCAGTCAGCTACTCAACAATCGCGTGGGCCATACCCGTAAACAAGGGAC

TGATCCAACCTCCGGACTACCATTTGCCATATTACGGTGAACCTAGTCCAGCACCCGGGCCCGAGCCTCCGGCAACGGCA

AATGCCCATACAGCACATCAAGTACTGAGTATTTTCAATGCGTTAGGGGTGATAGCATTTGCGTATGCAGGACACAACGT

GGTCTTGGAGATTCAAGCCACCCTTCCCTCAACTCCCGAAAGGCCTTCCAAAATCGCCATGTGGAGAGGGGTCGTCTGGG

CTTACGTGATTGTGGCCGCTTGCTACTTCCCAGTGGCTATCATTTGTTACTGGGCTTATGGAAACCAGCTTGAAGCCTAT

TCCAATATCCTTCAATTCGAAGGCATGCTCGGACAGAATTATAAGGGGATCCTCACTGCAGCTAATGTCATGTTGATTAT

CCACATTCTCGGAAGTTATCAGATCTATGCTATACCAGTCTTTGACATGCTGGAGACAGTGCTGGCGAAGAAATGGCTTC

TTCCTCCTTCCTTGAAACTTCGTCTGATCACTCGCACTACTTATGTCGGTTTTACAATGTTTGTGGCTACAATCTTCCCA

TTTTTCCAAGCGTTGCTTGGATTCTTTGGAGGTTTTGCCTTTGCTCCAACAACATACTTTTTACCTTGTTGTATATGGTT

GATTGTCTGTAAGCCCAAACGATTCAGCATGTCATGGACCATAAACTGGATTTGCATCATATTGGGGGTCCTACTCATGT

GTGCTGCAACCATTGGTGGCTTCTGGGCAATCGTGCATGAATGGAGCAACTACCAGTTTAGCGGGTTTTGGAAATGGCAA

GATTGTCCAGGAGTAAACTCAGCTCAAAATTGTACTGCTCCAACCCCAGCTCCACTCTAA

>Sphfalx0007s0031

ATGGGGTGGGAAGAAGATGGAAAAATTGCACAGGCTGAGGATGGAGCTGCAAATGGGGACAGTAATGGCTCTGCTGTCTA

TGATCCGACAAAATTTGATGAGGATGGCAAACCACGACGCAAAGGAAATGTCATGACAGCGTCAGCACATGTGATCACAG

CAGTTATAGGATCAGGGGTGCTGTCATTAGCATGGAGTATCGCACAAATGGGTTGGATTGCAGGACCTTCTGTGCTGCTA

TTGTTTGCAGCTGTCACGTACTACACCTCTTGCTTGCTAGCTGATTGCTACCGGTACCCTGACCCTGTCACTGGCAAGCG

CAACTACACCTACATGGAGACTGTGCAAGCCAATCTGGGTCCAAAGCAAGTGTGGGCCTGTGGTTTAGTGCAGTACTCGA

ATTTGCTGGGGACTTCTATTGGCTACACCATCACTGGTGCACAAAGTGCAAGAGCCATCACAAAGTCTAACTGCTTCCAT

TCGAATCCAGATTCACCTTGCCTAAGATCAAATAATGGGTACATGATAAGCTTTGGGATAGTGCAACTTGTCCTCTCACA

GATCCCCAATTTTGGCGAATTATGGTGGCTTTCTTATGTTGCTGCTGGCATGTCCTTCATTTACTCTACAATTGGCTTGG

GCCTTGGCATAGGGAAGATTGCAGAGGGAGGTTACTCACATGGGTCCATATCTGGCACTTCAATTGGTGATCCTAGTTTG

GCTGGTTATAACACTCGTGCTCAAAAAACTTGGAATGTATTTAATGCCCTTGGAGATATGGCTTTTGCTTATTCCTTCTC

ACTGATTCTCATTGAAATCCAGGATACACTAAAAGCTCCTCCTGCTGAGAACAAGAGCATGAAGCGAGCCGCACTTATAG

CAATTCTTACCACCACTGGGTTCTACATGTCAGTTGGCTGTGCGGGTTATGCAGCCTTTGGCAATGCTTCACAAGGAAAT

CTTCTGACAGGCTTTGGGTTCTATAACCCCTACTGGCTTGTTGACTTTGCCAATGCATGTGTTGTTGTTCATCTCATTGG

CGCCTATCAGGTGTATACACAACCACTATATGCATTTATTGAGGAATGGGTGTCCAGCAAGTTTCCCAAGAGTAACTTTA

TCAACAAGGAACACTATGTGAAGCTCCCCTTTGGTGAGCCATTGCCAATCAACCATTTCAGGTTGGTGTGGCGCTCTATC

TATGTGGTAATGACCACAATTGTTTCCATGCTGCTTCCATTTTTTAACGACATACTGGGTTTGATTGGAGCTTTTGCCTT

CTGGCCCCTGACAGTGTACTTTCCAGTTGAGATGTACATTCATCAAAAAAGACTCCCAAGATGGAGCCAAAAATGGATTC

TTCTGCAGTCACTAAGTGTGGTGACTTTTGTAGTATCACTTGCTGCAGCAATTGGATCTGTGGCAAGTATAGTCTCAGAT

GTGCAGGGCTACAAACCTTTCAGTAATGATGCCTAG

>Sphfalx0007s0033

ATGGGGTGGAAAGAAGATGGAAGAATCGCACAGGCTGAGGATAGAGCTGCCAATGGGGACAGTAATGGCTCTGCTGTCTA

TGATCCGACAAAATTTGATGAGGATGGCAAACCACGACGCAAAGGAAATGTCATGACAACATCAGCACATGTGATCACAG

CAGTTATAGGGTCAGGGGTGCTGTCATTAGCATGGAGTATCGCACAAATGGGTTGGATTGCAGGACCTTCTGTGCTGCTA

TTGTTTGGAGCTATCACATACTACACCTCTTGCTTGCTAGCTGATTGCTACCGGTACCCTGACCCTGTCACTGGCAAGCG

CAACTACACCTACATGGAGACTGTGCAAGCCAATCTGGGTCCAAAGCAAGTGTGGGCCTGTGGTTTAGTGCAGTACTCGA

ATTTGCTGGGGACTTCTATTGGCTACGTCATCACTGGTGCACAAAGTGCAAGAGCCATCACAAAGTCTAACTGCTTCCAT

TCGAATCCAGATTCACCTTGCCTAAGATCAAATAATGGGTACATGATAAGCTTTGGGATAGTGCAACTTGTCCTCTCACA

GATCCCCAATTTTGGCGAATTATGGTGGCTTTCTTATCTTGCTGCTGCCATGTCCCTCATTTACTCTACAACTGGCTTGG

GCCTTAGCATAGGGAAGATTGCAGAGGGAGGTTACTCACATGGGTCCATATCTGGCACTTCAATTGGTGATCCTAGTTTG

GCTGGTTATAACACTCGTGCTCAAAAAACTTGGAATGTATTTAATGCCCTTGGAGATATGGCTTTTGCTTATTCCTTCTC

AATGATTCTCATTGAAATCCAGGATACACTAAGAGCTCCTCCTGCTGAGAACAAGAGCATGAAGCGAGCCACACTTATAG

GAATTCTTACCAGCACTGGGTTCTACATGTCAGTTGGCTGTGCGGGTTATGCAGCCTTTGGCAATGCTTCACAAGGAAAT

CTTCTGACAGGCTTTGGGTTCTATAACCCCTACTGGCTTGTTGACTTTGCCAATGCATGTGTTGTTGTTCATCTCATTGG

CGCCTATCAGGTGTATACACAACCACTATATGCATTTATTGAGGAATGGGTGTCCAGCAAGTTTCCCAAGAGTAACTTTA

TCAACAAGGAACACTATGTGAAGCTCCCCTTTGGTGAGCCATTGCCAATCAACCATTTCAGGTTGGTGTGGCGCTCTATC

TATGTGGTAATGACCACAATTGTTTCCATGCTGCTTCCATTTTTTAACGACATACTGGGTTTGATTGGAGCTTGTGGCTT

CTGGCCCCTGACAGTGTACTTTCCAGTTGAGATGTACATTCATCAAACAAGACTCCCAAGATGGAGCCAAAAATGGATTC

TTCTGCAGTCACTAAGTGTGGTGACTTTTGTAATATCACTTGCTGCAGCAATCGGATCTGTGGCAAGTATAGTCTCAGAT

GTGCAGGGCTACAAACCTTTCAGTAATGATGCCTAG

>Sphfalx0007s0047

ATGGTGGGGACTAAAGTATCCGGCGCCGCGATGACGTTTTGTGAAGGAGGTGATGAACTTACGGGTTTCCAAAAGATGGC

TGTGGTGGATCAGGGAGTGAAGCAGCCGTTACCATCGCTCAATGTGGGCGGAGGAAATGTGCAAAATGGGCAATTGGGTG

GGGACCCGGAAGCAGGCCATGGTGGTGGAGGTCATTATCATCATGAAGCCTCCAAGTCTGGGTGGTCAGTCCCGGTGTCC

AGGGAGACGGTCCATCGCGTCGGACAAGACAGCTGGTGGGAGGTGGGTTTCCATTTCATTGCGGCTCTCAACAATGCTTT

CATACTAGGCTATCCAGCTCTCATCATGGCATACCTGGGTTTTGCAACTGGCTCTCTCTGCCTCATCGGAGGTGGTGTGA

TCTCTTTCTACAACAACTGCTTACTGGGAAGTCTTCATGAGACTGGTGGGAAGCGCCACATTCGATACCGTGACCTTGCA

GGTCATATCTATGGTCGTGGCATGTACAGAGCAACGTGGTTTGTGCAGTATTTCAACTTGAGTATTGCGAATGTGGGAAC

CATCATTCTGGCTGGTGAAGCTCTTAAGGCAATATGGGGGGCATTCACGGATAACACCAGTGTCAAGCTGGCAGGCTGGA

TTGTGGTTGCTGGTGTCTGTTTTGGCTTGTTTGCTTTTGTGGTTCCCAATTTACATGCACTGCGGTTCTTCTCAACCTGC

TCACTGTTTCTGTCTCTCATCTACACCTGTATTGCCATAGGTGTTGCATTCTCTGATGGTCTGAAAGCTGGACCCAGGGA

CTACTCGTTGAAAGGCACAAAAGCTGATAGAACATTCAATGCAATTGGAGCTCTAGCTACAATAGCGTTTGCATACAATA

CTGGCATCCTACCTGAGATGCAGGCAACAATTAGACAGCCAACAACAACAAACATCTATAAGGCTCTTGGAATGCAGTTC

ACAGTTGGAACCTTCCCTTTCTTAGTTCTCACCTTTGTGGGTTACTGGGCTTATGGAAACACAGCCAACCCTTACCTGTT

GCTCTCTCTTGGTGGCCCAAAATCCTTGGTGACAGTTGCCAATGCTGCTGCCTTCTTGCAAGCTATTGTGTCTCTCCATA

TCTATGCAACGCCCATGTATGAGTTCATGGACACACACTTTGCCAGAAAGGATCAGGGTGATTGGTCTGCTCACAGTATG

CTTGTCCGCCTCATCACACGAGGCACATACATCACTATCAGCACATTCCTTGGAGCTTTGCTGCCATTCTTTGGAGACTT

CATCACCCTCACCGGTGCAATGGCAGCCTTCCCTCTTGAGTCTGGCATCATCCACCACATGTACCTCAAGGTGAAGGGAA

AGGGGTTCAGTACATGGAGGCTAACATGGCACTGGTGCATTGTAGTTTTGTCAGGAGTACTGACTGTAGCCACATGTGCA

GCAGCAGTCCGGTACATCATCTCTGATTCAATATACTATCATGCTTTTGCTGACTTGTAA

>Sphfalx0007s0128

ATGGCTTACATCCCGGGACCGCAGAATTTCTCTTTCCATGAAGAGAATGAGGGCTCTGAAGATATAGAAACTCCGAAGAC

AATCAAGACCCAACAACCGAAAGAACAGAGGAATGTGGAGGAATGGTTGCCAGTTACGAGCTCGAGGAATGCCAAGTGGT

GGTACTCGGCCTTCCATAACGTCACGGCCATGGTGGGTGCCGGTGTTTTGAGCTTGCCCAGCGCAATGGCATATCTCACG

TGGGGGCCAGGGGTCGCAGTGTTGTTGTCGTCTTGGGTCATCACACTCTTCACGCTTTGGCAGCTCGTGCAGATGCACGA

AATGAAGGAATTTCCAGGCAAGCGCTTCGATCGCTACCACGAGCTAGGCCAACACGCTTTCGGCAAGAAGCTGGGACTCT

GGCTGGTTGTGCCACAACAGCTCATTGTGGAAATCGGCGTCGACATTGTGTACATGGTGACTGGAGGACCGTCATCATGT

CGTCCCATTCGAAAGACAGCATGGATTGCCATCTTCGGATCTGTCCATTTCTTTCTTGCACAATGTCCAAACTTCAATGC

GATCTCCCTTGTGTCTTTCTGTGCTGCAATCATGTCCCTCAGCTATTCAGCGATAGCTTGGGTTGCACCATTGGCCTCAG

GACAAGTTGCAGATGTTAGTTATGCATTACCAGATACCTCAAGGGCAGGTCTTGTCTTTGGCATTCTAAATGCACTGGGA

CAAATTGCATTCGCATATGCAGGCCATAATGTGGTGCTTGAGATTCAAGCAACACTGCCATCGACACCAGAGAAACCTTC

CAAGGGTCCAATGTGGCGTGGCTGCTTGGTGGCATATGTAGTGGTTGCTGCTTGCTATTTTCCAGTAGCAATGGTGGGTT

ATTGGGCCATGGGCAATGGAGTGGGAGACAATGTGCTCCTTTCTTTGGGCAAACCTGTGTGGCTCATTGCTGCTGCCAGG

CTCATGGTGGTTGTTCATGTCATTGGAAGCTATCAGGTGTATGCCATGCCTGTCTTTGACATGATGGAGACCTTCCTGGT

GAAGAAGCTAGAGTGGAATCCCACCCGATTTCTCCGCCTGTGGGTTCGATCACTCTATGTTGCTTTTACTATTTTCATGG

CAATGACAATCCCTTTCTTTGGAGATCTCCTTGGATTTCTTGGTGGATTTGCTTTTGCACCAACAACCTTCTTTCTGCCA

TGCTGCATGTGGCTCACGATCTACAAGCCCAAAGCTTTCAGCATGTCATGGATTCTTAATTGGGTACCCATTTAA

>Sphfalx0013s0130

ATGGCGACTGCGAAGGATGTGGAGAACGTCGACAATGGGCATGCACCGGATTCATCTGCGGCGGAACAAAAGGCTTTGGA

AGATTGGCTTCCCGTGACAGGGAACCGGAAGGCTAAATGGTGGTATGCAGCTTTCCACAATGTCACAGCTATGGTGGGTG

CCGGCGTTCTTGGTCTACCAACTGCCATGCAATGGCTGACCTGGGGTCCAGGAGTGGTAGTCCTAATTCTTTCCTGGGTG

ATTACACTTTACACTCTGTGGCAAATGGTGGAGATGCATGAGATAGTGCCCGGCAAACGCTTTGACAGGTATCATGAACT

CGGCCAGGAAGCTTTCGGTGAGAAATTGGGATTGTGGATTGTGGTACCACAACAGCTGATTGTGCAGGTGGGTGTTGACA

TCGTCTACATGGTGACAGGAGGCAGATCTTTGCAAACAATCTACAAATTGCTATGCAAGGGTCCTTGCACTCTTGAATTG

CACATATCCCTCTGGATTTTTATCTTTGGCTCCGTGCACTTCTTCCTGTCCCAGCTGCCAAATTTCAACTCTATTTCTGG

CCTCTCCTTGGCTGCAGCTATCATGTCGCTCAGCTACTCAACAATTGCATGGGCTATCCCTGCTCGTTATGGACACAGAC

TACCAGGAACAAACTCGGTGGTGTCTGCTGACTATCATCTGCCATACGGTCACAAGATAGGGGGAGATGTGATGAACATC

TTCAATGCATTAGGAACCGTGGCATTTGCATATGCAGGACACAATGTGGTGCTGGAGATTCAAGCCACAATCCCATCCAC

TCCAGAAAAACCTTCCAAATCTGCAATGTGGCGAGGAGTGCTGGTGGCTTACATGGTGGTTGCTGCTTGCTATTTCCCAG

TCTCTCTCGTTGGTTACTGGGCTTATGGCAACAATGCAACACTGCTGGTTGATGGCAACATCCTTACATTCGAAGCCTTC

CCAGTTTGGCTGGTTGTAGTGGCCAATTTTATGGTTATCGTCCATGTCATGGGAAGCTATCAGATCTATGCGATGCCCGT

GTTTGATACCCTGGAGACTCTGTTGGTCAGGAGGCTTCATTTCCCTCCATCCTTTCATCTTCGACTTGTCACTCGCTCTC

TTTATGTCATTTTCACAATGTTTGTTGGTATAACCTTCCCATTTTTCGGGGCTTTGCTTGGATTCTTTGGGGGATTTGCC

TTTGCTCCAACAACATACTTTTTACCATGTATTATGTGGTTGTGTATCGTCAAGCCTAAAGCCTTCAGTCTCTCATGGAT

CATAAACTGGGTTTGCATCGTATTAGGAGTCCTACTCATGTTTGCAGCAACAATTGGTGGGATGGCGAATATTATTGTCT

CGGCGTCAACATACAAGTTCTATGAGTAG

>Sphfalx0014s0033

ATGTCGAGAGCTGGAGCAAGATCGGCAATGGAGGAACCAGCGCCCGAGAAGCTGAACAGTACACTAGAGGAAGCTCATGA

GGGATCTGTACTCGATCATTCCAATACAGGCACCACTTCAAAACAGACTGCTTGGGTGACAACAGGCAGAGGGACATGGA

GACACGCCGGCTTTCATTTGGCCACCACCATTGCTACGCCCGCGGCGTTTGCACCTCTACCCTCTGCCGTTGCCGCCTTG

GGCTGGCCTGCTGGAGTAATCAGCTTGGTGATGGGCACATTGGTGACAAGCTATTGCAGTCTGTTGCTGGCCAATCTGTG

GGACTACAATGAACCCAACCGCTATGTTCGCTACCGTGACCTTGGTCGCGCCATTTATGGAAGGACGGGGTATTGGTCTG

TCACTGTATTTCAGCAAATAGCAGCCATTGGCAACAACATCACTATTCAAATTGTTGCAGGCTTGAGCATGCAGGCAATC

TTTGTGACATATAATACCTCAGACCCATCAAGGGTAACACTACAGGAGTTTATCATCATCTTTGGGGCAGCACAACTTGT

ACTGTCACAACTACCTGACATCCACTCATTGCGGTGGTTCAATGCCCTCTGCACATTCTGCACAGTGGCCTTCACCATTG

TTGTTATGGGTCTCTTGATTCATGCAGGGCAGAATAGAGATGGGCCTACAGACTATGGAGTGCATGGAACACCAAGTAAT

AAAGTGTTTGGGATTTTCCTTGCACTAGGCACAATAGCTTTCTCCTTTGGTGATGCAATGCTTCCAGAAATACAGGCAAC

AATCCGGGAGCCAGCAAAGAAGAATATGTACAAAGGAATATGCTTGGCATACTCAGTGATCACAACCACATACTGGCTAG

TTGCATTTCTGGGATACTGGGCTTTTGGCTTTGCTGTACAAGCATATGTTGTCAACTCCTTCAGTGGTCCCAACTGGGCC

ATCACTATGGCCAATGTCTTTGCTGTTATTCAGGTGGCAGGTTGCTTTCAGATCTACTGTCGACCAACTTACCAGTATTT

CGAGTTCCAGCTCATGAACCCAAAGCAACACCGGTGGTCACTTTACAACAGCTTGGCCCGGCTACTGGTAACCTCAATAT

ACACAGCACTGGTCACCCTAATTGCTGCTGCCATGCCATTTTTTGGTGACTTTGTGGCACTCTGTGGAGCTATTGGGTTC

ACACCTCTTGACTTCATTTTCCCAATACTTGCGTTCCTCCGAGTCAAGAAGCCCAAAAGTCGAATTTTCTGGGCATTCAA

CATCGGGATCGTAGTCGTGTACACATTGGTAGCCATTTTGGGTGCAATTGGTTCCATCCGGTATATTGTCAAGGATACAG

TACGATATCACTTCTTCCAGAATCAGTGA

>Sphfalx0015s0259

ATGGGCTGGGTAGTGGGTCCCATTGTGCTGCTCCTCTTTGCCCTGGTCACATACTACACCGCCCTGCTGCTCACAGATTG

CTACAGAAGCCCTGATCCTGTCTCTGGCAAGAGAAACTACACCTACATGGATGCTGTGAAAGCAAACTTGGGACCAAAGC

AAGTGGTTATCTGTGGGCTAGTGCAGTACACCAATCTGTTGGGGACTGCAATTGGATATACCATCACTGCTACAACAAGC

ATGGTAGCTATAAAGAGATCCGACTGCTTTCATGCAGATGGAGATAGTGCACCATGTCATGTATCCAACATCATATACAT

GGTTTTCTTTGGCATAGTACAGGTCATTTTGTCTCAGATCCCTGACTTTGATCGAATCTGGTGGCTCTCAATAGTTGCTG

CAATTATGTCATTCTCATATTCAACCATTGGCCTTGGCCTTGGTTTAGGGAAAGCTTCAGAAGGAGATCATCCTCATGGC

ACTTTGACTGGGGTAGAAATTGGTGACCCCAGTATTGGCTTTGCAACAAAAGCTCAAAAGATCTGGGATGTTTGCAATGC

ACTTGGCAACATAGCATTTGCATACTCATTCTCCATGATCCTTATTGAAATCCAGGACACATTGAAAGCTCCACCTGCTG

AGAACAAAACTATGAAGAGAGCAACTCTTATTGGGATACTCACCACCACCATATTCTACATGTCTGTGGGCTGTGTGGGC

TATGCTGCCTTTGGGGATGGGGCCCCTGGCAATCTCCTTACAGGCTTTGGATTCTACAATCCATATTGGCTTGTTGACTT

TGCCAACGCTTGCATTGTTGTCCATCTTGTTGGAGCTTATCAGGTCTACACCCAACCACTGTTTGCATTCGTCGAACACA

CAATGTCCCGCAAGTTCCCCAAGAGTGACTTCATCCACAAAGACCTTGAGATGAAGCTCCCATGGGGAGCACCTTTACAT

ATCAACCTGTTCCGTCTTGTCTGGCGCACGGCATTTGTAGCATTCACCACAGTTGTGTCCCTTGTGATTCCCTTCTTCAA

CGACGTACTAGGGCTAATTGGAGCTTTCTCATTTTGGCCCCTCACAGTCTACTTCCCAATTCAAATGTACACAGTGCAAC

AGAGCATACACAAATGGAGCTCAACATGGCTAGCACTCCATACCTTGAGTGTCGTGTGCTTCTTTGTATCATTAGCTGCA

GCAGTGGGATCTATTGCTGGAATTCTCACCGATCTCAAACATTACACTCCATTTAAATCCTAG

>Sphfalx0018s0093

ATGACGTTTAAGGAAGGATTGGAGCTCATGGTCGACCCAAACATGAATGCCGCGGCTCCTCAAGGGGGACTATTGTCGAA

GTCGACGTCGTTGCATCAGCAGCAGCCATCGCGGCTTTCATGCAATGTGCAGCAAGGAGCTGGTGGAGTCGATCTTCTTC

TTCATGAGCATCAAGATGTGCAATTGGGCCCCGGGGGGGATCCGGAAGCAGCAGCAGGAGGTACTACTGGTACTCCGGGC

TCCAGTACTACCGGCAGCTCCGGCAAGTCCGTCCGCCCACTGCTGGTGTCCAGAGACACCGTCCATCGCGTGGGTCAAGA

CAGTTGGTGGGAGGTGGGTTTCCATTTCATTGCAGCACTGGACAATGCCTTCATCCTGGGCTATCCAGCTCTCATCATGG

CCTATCTGGGCTGGGCAACTGGCACCATCTGCCTCATTGGAGGTGGCATCGTCTCGTTCTACAACAACTGCTTGTTGGGC

AGTCTTCATGAGACTGGTGGAAAGCGCCACATTCGATACCGCGACCTTGCTGGTCACATCTATGGTCGCGGCATGTACAG

AGCAACATGGTTTGTGCAGTACTTCAACTTGAGCATTGCAAATATTGGAACCATCATTCTGGCTGGTCAAGCCCTTAAGG

CAATTTTTGGGGCTTTCAGTGATAACACCAGTGTCAAGCTGGCGGGCTGGATTGTGATTGCGGGTGTCTGTTTCGGCTTA

TTTGCCTTTGTGGTTCCCACTTTACATGCGCTGCGATTCTTCTCCAGTTGCTCGCTGTTTCTGTCCCTCATCTACACCTG

CATAGCCATTGGTGTTTCCTTCTCTGATGGTCTCAAAAGTCCACCACGGGACTACTCTTTGAAAGGGACAAGAGCTGATC

GAACTTTCAATGCAATTGGAGCTCTAGCAACAATCGCATTTGCATACAACACTGGCATCCTCCCTGAGATGCAGGCAACA

ATCAGGCAGCCGACAACTACAAACATCTACAAAGCTCTTGGAATGCAATTCACATTAGGGACTTTCCCTTTCTTATTGCT

CACCTTTGTCGGCTACTGGGCTTATGGAAATACAGCCAGTATTTACTTATTGTCGTCTCTTGGTGGTCCAAAGTCGTTGG

TCACCATTGCCAATGCTGCTGCCTTCTTGCAAGCTATTGTCTCTCTCCATGTCTATGCAACACCCATGTATGAGTTCATG

GACACACATTTTGCCAGGAAGGATCGGAGTGAGTGGTCTGTGCACAGCATGCTTGTCCGCTTCATCACCCGAGGCACCTA

CATCACTATCAGTACATTCCTTGGAGCTTTGCTTCCTTTCTTTGGGGACTTCATCACTCTCACTGGTGCCATGGCTGCCT

TCCCTCTCGAATCTGGCCTTATCCACCACATGTACCTCAAGGTGAAGGGGAAGGGATTCAGCAAATGGAGACTACTATGG

CACTGGTTTATTGTAGTTATTTCAGCAATTCTAACTGTAGTCACATGTGTAGCAGCAGTTCGGTACATCATCCAAGATTC

AACAAACTACCATGCTTTTGCTAACTTGTAA

>Sphfalx0025s0047

ATGAATATGGAGGGAAAAGATCATGCTAGGGACTTCAAGATGCAAGAAGCGAATGGTTTGACAAACGGGTTCAACTCACT

GGATGTGGTGTCACCATCACCATCACTCTCACAAGAGGAGTATCGACATTCAAGCTTGAGCGATGCTCGCAAAGATGTGG

GGACTCTGAAGGTTCTTGAATCAAAAGGAACGTGGTTGCATGCAGGATATCATTTGACAACAGCCATTGCTGCTCCATCA

CTGCTTTCATTGCCATATGCATTCTCATTTCTGGGATGGGCACCAGGATTACTGGCAATCACAATCTGTGGCCTGGTGTC

AAGCTATGCATATTGCTTACTTTCTCAAGTCTTAGATGATTGTGCTTCCAAAGGCCATCGATTCTATCGTTTCCGTGAAC

TCTCCCAATTTGTAATTGGACAAAAATGGACGAGATTTTTTGTGATGCCTGTGCAATTTGGAGTATGTTTTGTGACTGTT

ATTGGTGCTATTTTAGCAGGTGGCAGTGCGGTTAAGCTGATTTATAAGGGAGCAAATGCCAATGGGAGTATTCCATTAGC

AGAATTTGTGGCAATGTTTGGTGCTGTGATGATACTTCTTTCACAATTACCATCCTTTCATTCACTGCGCTATATCAACT

TGGTTTCTGTAATACTGTGCTTAATCTACAGCCTTGCTGCTACTGTAGGCTCTGTTTTGGCTGGCTATAACAAGCACATC

CCACCTAAAGATTACTCTGTAGTTGGAAATCCCACTGACAAACTGTTTGGTGTTTTCACCTCACTATCGGTCATGGCTGG

AGTTTATGGGGTGGCAATAATCCCAGAAATACAGGCAACAATTGCACCTCCAATTGGTGGGAAGATGGTGAAAGCTATTG

CATTGTGCTACACAGTAGTGGTGGCCACTTTTTATTCAGTATCCATTGCTGGGTACTGGGCTTTTGGAAATGATGCACAA

GGGAATTTATTTGATAACTTGGTGCCAAGTTATGGACCCCAACTTAATCCAACTTGGCTGGTAGCTATTTCGGGCTTTTC

AATAGTTGCTCAACTCCTTGCTATTGGCCTGGTGTATCTTCAGCCAGCATTTGACGTATTTGAATCATTAACAGCTGATG

CAAATTGCGGGAGGTACACCTTGCGCAATGTGGTTCCTCGATTAGTTTTGCGTTCAACTTATGTGTCCTTGGCAACATTT

CTTGCAGCCATGCTTCCATTCTTTGGGGACATAGTCTCCCTAGTTGGAGCATTTGGATACACACCATTGGACTTTGTCCT

ACCCATGCTTTTCTACCAACTTGTGTTCAAGCCATCAAGAAGGACTTATATGTTTTGGCTCAACTGGGTCATCATAGTGA

CTTTTACAACAGTTGGAATTGTTGGATGTATAGCTACAGTACGTCACATTGCAATTGATGCCAATAATTATAAATTGTTT

GCTAATGTGTAA

>Sphfalx0026s0089

ATGACGTTTAACGGATCAGGTGGATTACCATGTGAGGAGCAGTTTGCGGGCCGGTTCCAAAAGATGAATGCGGAGCTGCA

GCTGGGGGCGCCCAAGAAGTCGGTCATGCCATCGCTCGACCGCGTGGGATCAGATCATCAGCACGATGCAGGACAATTGA

GGGGGGATCCGGAATCAGGCCATCATGATGCAGCAGTGGATCATCACCTACAAGCTGCTGGCTCCTACAAGTCTCAGGGA

TCCCAACTTCTGGTGTCCAGACAGACGGTTCATTGCGTCGGTCAAGATGCTTGGTGGGAGGTGGGCTTCCATTTCATTGC

AGCAGTCAACAATGCCTTCATCCTGGGCTACCCAGCTCTCATCATGGCGTATCTGGGTTGGGCAATGGGCTCCATCTGCC

TCATCGGAGGCGGCATCATCTCCTTCTACAACAACTGTTTGCTGGGCAGTCTTCACGAGACTGGCGGCAAGCGCCACATT

CGTTACCGTGATCTTGCGGGCCACATCTATGGTAAGGGTATGTACAGAGCAACTTGGTTTGTGCAGTACTTCAACTTGAG

TATTGCAAACATCGGAACCATCATTCTCGCTGGTGAAGCTCTCAAGGCAATATGGGGGGCATTCAGTGATAACACAAGCA

TTAAGCTGGCTGGTTGGATTGTGATTGCGGGCATCTGTTTTGGCTTGTTTGCCTTTGTGGTTCCCAATTTACATGCCCTG

CGATTCTTCTCCACCTGTTCACTGCTTCTGTCTCTTATCTACACCTGCATAGCCATAGCTATCGCCTTTTCAGATGGTCT

AAGAAGTGCACCCAGGGACTACTCACTCAAAGGAACAAAAGCTGATAGAACATTCAATGCAATTGGAGCTCTAGCTACAA

TTGCATTTGCATACAACACTGGCATTCTCCCTGAGATGCAGTCGACAATAAGACAGCCAACAACTACGAACATATATAAG

GCTCTTGGAATGCAATTCACAGTTGGGACTTTCCCCTTCTTAGTGCTCACCTTTGTTGGCTACTGGGCTTATGGGAATGC

TGCCAATCCTTACTTGTTGCTCTCCCTTGGTGGCCCAAAGTCCTTAGTCACCATTGCCAACGCTGCCGCCTTCTTGCAAG

CTATTGTCTCTCTCCATATCTATGCAACACCAATGTATGAGTTCATGGACACATACTTTGCCAGGAAGGATCAGAGCGAA

TGGTCTCTTCACAGTATGCTTGTGCGCTTCATCACCCGAGGCACCTACATCACTGTCAGCACATTCCTTGGAGCTTTGCT

TCCTTTCTTTGGGGACTTCATCGCCCTCACTGGCGCCATGGCTGCCTTCCCCCTCGAGTCTGGCATCGTCCACCACATGT

ACCTCAAGGTGAAGGGGAAGGGTTTCAGCACATGGAGGCTAATATGGCACTGGTCAATTGTAGTTATTTCAGCAGTGCTG

ACGATAGTCACTTGTGTAGCTGCAATTCATTACATCATCTCTGATTCAACATACTACCATGCTTTTGCTGACATATAA

>Sphfalx0040s0186

ATGCCAGAGTACTTCTCGGACCCTCATGCCGTTCATGTTGTTGCATTAGCGCCCCTGCAAGACCAAGTCGCAGACCTGGA

GAACTTTACAACAACCGCAGCTTCCGACGACTTCCAGCAAGAGAGGAGAGATGTGGAGAATTGGTTGCCTGTCACTAGCT

CCCGCAACGCGCGATGGTGGTACTCAGCATTCCACAACGTCACAGCCATGGTCGGTGCAGGCGTCCTAAGTCTGCCCAGC

GCCATGGCTTATCTCACCTGGGGACCAGGCATGGCGCTGTTGGCGTTGTCGTGGGTGGTCACCCTCTTCACTTTGTGGCA

GCTTGTCGAGATGCACGAGCTGGAAGCTGAGCCTGGCAGACGCTTCAACCGATACCATGAGCTTGGGCAGTACGTGTTTG

GTGAGAAGCTCGGGCTTTGGCTTGTGGTGCCACAGCAGCTGATCGCCATGGTCGGCGTCGACATTGTGTACGTATTGACA

GGTGGAAGCTCGTTGATGGGTGCATATGATTTGTTGTGCACCGATCCAGTTGGGTGTCGCCATATTCACAAGACGGCCTG

GATTGCAATCTTCGGCTCCGTGCATTTCTTTCTTGCACAATGCCCGAATTTCAATTCCATCTCCATTGTGTCTTTCTCTG

CTGCAATCATGTCTGTCAGCTATTCAATGGCAGCTTGGATTGCGCCATTGTGCGCGGGAAGAATCGCAAGCGTGAGCTAC

GCCGTACCTGCTCATGAGTCCAAGTCTACAGCAGGAATTGCGTACTTCGGAATTCTCAACGCCGTCGGGCAAGTCGCGTT

TGCATTTGCAGGACACAATGTGGTACTTGAGATTCAAGCATCGCTTCCGTCGACACCAGAGACACCCTCCAAGGTCCCCA

TGTGGAGGGGTTGCCTACTAGCTTACGCGGTGGTTGCGCTTTGCTATTTTCCTGTCGCAATAGTGGGCTATTGGGCGATG

GGGAATGGAGAGGGCGACAATGTCCTCCTCTCCTTGGGGAAACCTGTCTGGCTCCTTGCTGTTGCAAACCTCATGGTCGT

CATCCACGTTCTTGGCAGCTATCAGATTTTCGCAATGCCAGTTTTCGACATGATGGAGAGCATTCTCGTGTCGAAGCTGG

AGTGGAACCATACTCGCTCTCTCCGATTGTGGGTGCGCTCGCTCTATGTCGCATTCACCATCTTTGTGGCAATTACAATT

CCGTTTTTCGGAGATCTGGTCGGATTCCTCGGAGGCATTGCATTTGCACCAACAACTTTCCTCCTTCCTTGCTCCATGTG

GCTGAAGATCTTCAAGCCAAAGGCCTTTAGCTTCTCCTGGATCCTTAACTGGTTCTGTATGGTGTTGGGTTTACTCATTT

GGCTGACTATACCAGTTGGTGGTCTGCACCAAATCATCATCATCAGCTTCTCAAAGTATAAGTTCTATGGATAG

>Sphfalx0065s0028

ATGGCAATGAACAATATTGGAGCTGGAGGTGTTCAAGGGTTCCATGTCACCTCAATGGAGCGAGCTAGATCTCTCCGAAC

GCCTGCTGAAAATGATCATTCTGTTGTAAATTTAGGAGCTGTTGCTCCTGTTGCAACTGATGCAGACTCTTTACCGGTTA

CAGTTGGAGACACCAATTCCGGAGAAATGGTGAATCCTGCAGCGCCAGCAGCGTCTGCCCATGAGATTGGCAAAGATCCA

TGGTGGGTGGTGGGATTCAGTTTGGTAGCTGCCAGCGACAGCAGCTACATGTTAACTTATCCTTTTCTCATCATGTCACA

ATTAGGGTGGATCGCTGGGCCCATTATCCTGATCCTGCTTACTGCTCTCTCCTGCCACAACAATTGTTTATTCGGTAGTC

TTCACGAAACTGGAGGGAAGCGCCATATTCGAACTCGTGATCTCATTAGCTACGTCTACGGTCGACGGTGGTTGACAAGA

ACGGTATGGATAGTGCAATTCACCGTCTTAATCATTTTTTGTATTGGAACTTTCATATGGGCTGGAACATCTTTACAGGC

AATTTACGTTACTTACAGCAATGATCCGACCAAGATCTCATTGCCGGTCTGGATTGCCATAGCTGGTGCCTGTTATAGTA

TCTTCGCGTTTTTCGTGCCCACTTTGCATGCGTTGGGACTCTACACCGCCATATCTCTTCTCCTCACTATGATCATCACC

TTCATATCACTTGGCATCTCCATCAAAGACGGCATGAAATCTGGAGTAAAAAGGGACTATTCACTCGTCGGCACTACAGC

TGACAAATGGTTCGGTGCATTGGCATCTCTATCCATTGTTAGCTTTGCATTCAACATCCCCATTCTCCCAGAATTGCAGG

CTAATGTGAGGCCACCCACAGTGCGAAATATTTACAAAGCTCTTGGATTTGAGTACATAGTCGGTGGCACCCCGGTCATA

GTGCTCGTATTTGTTGCATATTGGGCTTATGGTAACGGTGTGAGTGAGTACCTACTGTATTCAACCTCTGGTCCCACATG

GCTGGTTACAGTTGCCAATGTAGCGGCATTCTTACAAGTCCTAGTCACCATACACATCTTTGCTTTGCCGATGTTTGAGT

TCTTTGACTCAATGCTCTCCAAGCTAAAAAGGCGCCATGCCGAGGGGGATGTTGCTGACGACCACACACTCAGAGCTAGG

ACTGGGCCGTGGTCAAAACACAAGATGCTGCTTCGCCTCGTCACGCGCACAACGTTCATTAGCATCACTACTTTAGTTGG

AGCTATGTTTCCATTTTTTGGAGACATTCTTGAACTGGGTGCAGCTTTGATTGTCTTTCCTCTCAACTATGGCTTGGTCC

ACCACATGTATTTGAAGGTGAACGGCAAAACAATTTCATGGTACCAGCGTGCATGGCATTGGTCAATCATAGTAGCAGCC

GTTGTTTTAACTGTGTCATCTGTAACTGCATCTACGCGAAACATTATCGTAAGTTCATCTACTTTTCACATCTTTAAGAG

TAGCAGTTGA

>Sphfalx0065s0034

ATGGACAATAATGGAGCTGGAGGTGTTCAAGGGCCCCATGTCGTCTCAATGAAGCGAGCTGGATCTCTCCGAGGGCCTGC

TGAAAATGATTATTCAGTTGTAAATTTAGGAGCTGTTGCTCCTATTCCAGCTGATGGGGACCCTTTACCGTTTACAGATG

GAGACAGCAATCCTGGAAAAATGAAGAATCTTCCACCTTCATCAGCAACCGCCCATGAGATCGGCCGAGATCCATGGTGG

GTGGTGGGATTTGATTTGCTAGCTGCGAGTTCCAGTAGTTACATCTTGAGTTATCCTCAACTCATCATGGTACATTTAGG

CTGGATAGCTGGGCCCATTATCATGGTCCTGCTTAATGCTGCCTTCTTCTACAACAATTGCTTATTGGGTAGTCTTCATG

AAACTGGAGGGAAGCGCCAAATTCGAACTCGTGATCTTGTCGGCTACATCTATGGTCGACCAATGTTCACAGCGGTATGG

ATACTGCAATTCACCGTCTTGATCGTGTTTTGTATTGGGACGTTCGTATGGGCTGGAACAACATTAGAGACAATTTACTC

TTCTTACAGCAGTGATCCGAGCAAGATCTCACTGTCGGAGTGGGTTGCCGTAGCTGGTGCTTGTTATAGTGTCTTCGCGT

TTGCGGTGCCCACTTTGCATTCGTTGCGACTCTACAGCACCATTTCTCTTGTCCTCACTATGATCATCACCTTCATAACC

ATTGGCATTTCCATCAAGGACGGTGTGAAATCTGGAGTGGCAAGGGACTATTCAATTGTTGGCACCAAAGCTGACAAATG

GTTCAGTGCAATGGGAGCTCTGTCCATTGTTGGCTTTGCATTCAACACCGCCATTCTCCCGGAATTGCAGGCTTCTGTGA

AGCCACCCACGGTGCGAAATATTTACAAAGCTCTTGGACTTCAGTACACGGTCGGTGCCATCCCAGTCATAGGGCTCACA

TTGATTGTATATTGGGCTTATGGTAATGTTGTGAGTGGATTCGCACTGGAGTCAAACTCTGGTCCCAAATGGTTGGTTAC

AGTCGCCAAAGTAGCAGCATTCTTCCAAATCCTCGTCACCTTACATATCTATGCTTTACCGATGTTTGAATTCTTGGACT

CGAAGTTCTTGAAGGCATATAGGAGCCTCAAACCCAGTGGGAATGTTCAAAATGATGTCCCCCAAACTGGTCGGAATTGG

TCAGCTCGCACGATGCTAGTTCGCTTCCTCACGCGCATCATGTTCATTAGCATCACAACTTTGTTTGGAGCTATGTTTCC

ATTTTTTGGAGACATTCTTGAACTCGGTGGAGCCCTGATTGTCTTTCCTCTCGACTTTGCCGTGGTGCACCACATGTATA

TGAAGGTGAAGGGCAAGAATTTTTCATGGTACCGACGCACATGGCATTGGTTAATGATAGTGATAGCCGTTCTTTTAACG

ATAGCATCTGTGACTGGAGCTGTGCGAAATATTATTGCATCTTCATCCACCTATAAAATCTTTCACAGTAATAACTAA

>Sphfalx0075s0052

ATGGTGGGGGAGAAGGTCTTAGGCTCTGCGATGATGTCGATGGTTGTTCACGAAGAAGGAACTGCAGATCCGCCGGCTGG

GTTGAAGTCGACGGTGCCATCGCTAGTCTGCAGTGTGGGAGGATATAATAATCAGCAATCTGATGATGCAGGGCCATTGG

ACCACGGCAATCCGGAAGCAGCAGCAGCCCATGGTGCAGCCGGGGATTATGATCATCACGTCGAAGCTCATGCAGGCAGC

TCTAAGCAGTTCATGTCTAGTGGGGCACTAGTGCCGCCGCCCGTGTCCAGAGAGACAGTCCATCGCGTCGGCCAAGATAG

TTGGTGGGAAGTGGGTTTCCACTTCATTGCAGCAGTGAACAATGCTTTCATTCTGGGCTACCCAGCTCTCATCATGGCGT

ATCTGGGATGGGCCACCGGCTCCCTCTGCCTCATTGGCGGCGGCGTCGTCTCTTTCTACAACAACTGCTTGTTGGGGAGT

CTTCATGAGACTGGCGGCAAACGCCACATTCGTTACCGCGACCTTGCAGGTCACATCTACGGTCGTGGCATGTACAGATT

GACATGGTTTGTGCAGTACTTCAACTTGAGTATTGCAAATGTGGGAACAATCATTTTGGCTGGTGAAGCTCTTAAGGCAA

TATGGGCTGCATGCAGTAGCACTGACAACAGCAGCAATCTGAAGCTAGCAACCTGGATTGTAATTGCGGGCATTTGTTTT

GGTTTGTTTGCCTTCATGGTTCCCACTTTACATGCACTGCGATTCTTCTCCACCTGCTCACTGCTTCTGTCTCTCATCTA

CACCTGCATAGCCATAGCTCTTGCATTTTCTAATGGTCTGAAAAGTCCACCCAGGGACTACTCCTTGATAGGCACCAAAG

CTGATAGAACATTCCATGCAATTGGAGCTCTAGCTACAATTGCATTTGCATACAACACTGGTATCCTCCCTGAGATGCAG

GCAACAATCAGGCAGCCGTCAACAACAAACATCTACAAGGCTCTTGGAATGCAATTCACAGTGGGGACTTTCCCTTTCTT

AGTCCTCACTTTTGTTGGTTACTGGGCTTATGGAAGTGCCGTCCAGCCTTACTTGCTGCTCTCTATCCATGGCCCAAAAT

CTTTAATCACAATTGCCAATTCTGCTACTTTCTTGCAAGCCCTTGTCTGTCTCCATATCTATGCAACACCCATGTATGAG

TTCATGGACACTTACTTTGCCAGAACTAAGGATGAAAGCGATTGGTCTGTCCACAGAATGCTTGTCCGCTTCATCACAAG

GGGCACCTATATCACTATCAGCACATTCCTTGGTGCTCTGTTACCTTTCTTTGGTGACTTCATCACCTTCACTGGTGCCA

TGGCTTGCTTCCCGCTTGAGTCTGGCATCATCCACCACATGTACCTCAAGGTGAAGGGCAAGGGGTTCAGTAAGTGGAGG

TTAGCATGGCACTGGTTCATTGTAGTACTGTCAGGAGTACTGACTGTAGCTACTTGTATAGCAGCAGTCCGATACATCAT

CTCTGATTCAGTATCCTACCATGCTTTTGCTGACATTCAACAAGTCTAG

>Sphfalx0084s0062

ATGGAGGATCGTGTTCTCCAGGTTGAGGATCGGGATTACAATAGCAAACTGAATGACTTGCTACCTGTGACTGCGAGCAG

GGAAGGCAAATGGTGGTACTCCGCTGTCCATAATGTGACGGCTATAGTGAGTGCTGGAGTTCTTGGTCTACCCAGTGCCA

TGGCAGATCTCACCTGGGGTCCAGGCGTAGTCGTGTTGACCCTGTCATGGGTCATCACCTTTTTCACCTTGTGGCAAATG

GTAGAGATGCATGAGATGGTGCCCGGTAAACGTTTTGACAGGTACCATGAACTGGGCCAGCAAGCGTTTGGAAAGAAGTT

GGGACTGTGGATAGTAATACCACAACAGCTTCTTGTGGAGGTAAGCGTGGATATCGTATACATGGTGACTGGAGGCCAAG

CGTTGAGATACATCTATATCTTGAACTGTAAAGGGGATCATCATCATTGCCCTTTGAAACCTTCGAGCACGGAGATAAAC

AATAACCCATACGCGATGACTTCTCACTGGATTCTTATTTATGGCTCGGTGCACTTCCTATTCGTCCATATCCCGAACTT

CAACTCGATGGCTGGAATCTCTTTGGCTGCAGCCATCATGTCAGTCAGCTACTCGACAATTGCGTGGACCATACCTATAT

TCCACAATGAACACAGCCAACATCGTGACATTGCAAATTATACGCTTGTAAACGTAACTGCAGACTACGATTTGCCAAAG

GCCAGTACAGCAGGTCATGTACTGAGCGCTTTCAATGCGTTAGGGGTTGTAGCATTCGCGTATGCAGGACACAACGTGGT

TTTGGAGATTCAAGCCACTCTCCGCTCCAGGCCCGGAAAACTATCCAAGATCGCCATGTGGAGAGGGGTGTTCTTGGCTT

ACGTGATTGTCACGATTTGCTACTTCCCAGTGGCTCTCGTCTGTTACTGGGCTTATGGAAACCAGCTTGCAGCCGATTCC

GATTCCAATTCCAATTCCACTTTCAATTCCAATATCCTTCAATTCGAAGGCTTCCGCGGCATAACCACTGCAGCTAATTT

CATGGTCATCATCCACATTCTCGGAAGTTATCAGATCTATGCTATGCCAGTCTTTGACATGCTAGAGACAGTGCTGATGA

AAAAATGGCTTTTAGCTCCTTCCTTCAAACTTCGTCTAATCACTCGCTCTACTTACGTCGGTATTACAATGGTAGTGGCG

ACGATCATACCATTTTTCCAACCTCTTCTTGGATTTTTTGGAGGCTTCGCTTATGCTCCAACTACGTACTTTTTGCCTTG

TTGTATATGGTTGGCTGTCTATCAGCCCAAGCGATTCAGTGCATCTTGGACAATAAACTGGATTTGCATCATATTGGGGG

TCTTACTCATGTTAACGGCAACTATTGGTGGCGGCTGGGAAATCGTCAAAGAATGGAACACCTACAAATTTAGAACTTTT

TGGTAA

>Sphfalx0084s0063

ATGGCGACTACTAAGGATGTGGACGATGTCGACAACGGGTTTGCACCTGGACCAGGAGGAAAGACTGTGGACGATTGGTT

ACCTGTGACAGGGAGCAGGAATGCTAAATGGTGGTATGCGGCTTTCCACAATGTGACGGCTATGGTCGGTGCCGGTGTTC

TTGGCCTGCCTACTGCCATGCAATTCCTGACCTGGGGTCCAGGAGTGGTTGTTTTGGTGCTTTCATGGGTTATCACGCTC

TATACACTTTGGCAGATGGTGGAAATGCATGAGATGGTGCCTGGCAAGCGGTTTGATCGGTATCATGAACTTGGTCAGGA

GGCTTTTGGCCCAAAGCTGGGACTCTGGATTGTGGTGCCACAGCAGCTCATTGTGCAGGTGGGTGTGGACATCGTGTACA

TGGTGACAGGAGGCAAATCTTTGCAGAAATTCTATCATCTGGTCTGCAAGGGCAACTGCGATCTGTATAAGCACACATCC

CTTTGGATTGTGATATTTGGCTCTGTGCATTTCTTCCTCTCCCAACTCCCCAATTTCAACTCAATTTCTGGGATCTCCTT

GGCTGCAGCAATCATGTCACTCAGTTACTCCACAATCGCATGGGCCATCCCTGCCCATTATGGACACACGATACCAGGAA

CAACTCAATATGTACATCCTGACTATCATCTACCTTACCATCACAACGTAGGTGGATATGTCTTTGAAGTTTTTAACGCA

TTGGGAACTGTGGCATTTGCTTATGCGGGCCACAATGTCGTATTGGAGATCCAGGCCACCATCCCATCAACTCCTGAAAG

ACCTTCCAAAATTCCAATGTGGCGGGGAGTCATCCTCGCTTACATTGTCGTTGCAGCTTGTTATTTCCCAGTATCTCTTG

TCGGATACTGGGCTTATGGCAACAACAATGGACTTGTAGCGGATGGGAACATCCTTGCATTTGAAGGCTTCCCAGTTTGG

CTAGTTGCAACAGCCAATCTCATGGTTGTCGTCCATGTTATTGGAAGTTACCAGATATATGCCATGCCGGTCTTTGATCA

GCTGGAGACTGTGATGGTGAAAAAGTTGCATTTCACTCCATCTGTCTTACTTCGACTCGTCACTCGCTCTATTTATGTTG

CTGTAACGATATTTGTTGGTGTAACCTTCCCATTTTTCGGGGCTTTGCTTGGCTTCTTTGGAGGCTTTGCTTTTGCTCCA

ACAACATACTTTTTACCATGTATTATGTGGTTGTGTATCTACAAGCCAAAACCCTTTAGTCTCTCATGGATCACCAACTG

GATTTGTATCATATTGGGGGTCCTGCTAATGTTTACAGCATCAATTGGTGGCCTTCAGCAAATCATTGTGGATGCAAAAA

CCTACAAGTTCTATACATAG

>Sphfalx0104s0057

ATGGAGACTCAAATACAGGCAAATGGAGTTCACGGCGCGCATCATGAGGATGAGAAGCCTCTGCGCTTGGCGGCCTTTGA

AATGGAAATACCCATAGGTACCAGACGCAACGGCAATTGGTGGTCCGCCGCCTTCCACAATGTTACAGCGGTCGTGAGTG

CCGGCGTTCTTGGCCTACCCAACGCCATGGTCTTCCTCACCTGGGGTCCAGGCATAGTGGTTTTGATTCTTTCGTGGATC

GTCACCCTATTCTCCTTCTGGCAACTCATTCAGCTCCATGAACGCCCTCCGAACAAGCGCTTCAACAGGTACCATGAGTT

GGGGCAGGAAGCGTTCGGGAAGACTCGAGGGTTTTGGATCGTGGTACCGCTACAACTCCTGGTGGAGCTGAGCGTCGACA

TCCTGTACATGGTGGTAGGCGGCCAGGCCTTGCAGAACATCTACAACATGAACTGCAGCGGCGATTGTCCGTTGAGCAAC

CTGGGTCCGAATCCATATCGTCGGACTTACATTTGGATTCTGGTGTTCGCTTCCGTCTACTTCCTGCTCGTCCAACTCCC

TACCTTGAGCTCGCTCTCAAAACTCTCCTTGGCTGCAGCCATCATGAGCATCGGCTACTCGACGATTGCGTGGATTATTC

CGGTTGCACTCAATCACCAACATAACGACATGACAAATGGCAATGGGCTTGTTAGCGTAACTCCTCGCTATCATTTGCCC

TATTACGACGGAACAACGCGAGGAGGAAAGTTTCACCACACGGATACAGAGGCATACGTACTCAGCATCTTCAACGCGTT

GAGCACTGTAGCATTCGCGTATGCAGGACACAATGTTTCGTTGGAGATTCAAGCCACGCTGCCATCGACTCCTGAAGAGC

CGTCCAGGATCGCGATGTGGCGAGGAGTGAAGCTGGCTTATGCTATCATCGCTCTTTGCTACTTCCCTGTGGCCCTCATT

TGCTACTGGGCCTACGGCAACGATCTCAAGAAAAGCAACTTGGGTATTCTGCGCTACGAGAGCTGGCCGGTTTGGCTGGC

TACTGCAGCCAATCTAATGGTCGTCATCCACGTGACTGGAAGCTACTTGATCTATGCAATGCCACTGTTTGATATGATGG

AGCTTGTGTTGGTGGGAAAATGGCGTCTCCCTCCTTCTTTCAAGCTTCGTCTCATCACTCGCTCTCTTTATGTCGGTTTC

ACAATGGTTATGGCTATGACCTTCCCATTTTTCAAAGGGCTCTTGGGTTTCTTTGGCGGATTTGCATATGCTCCAACCAC

GTACTTCTTACCATGTTGCATGTGGTTGGCTATGTGCAAACCCAATAGGTGGAGTCTCGAGTGGACCGTAAACTGGATTT

GCATTATACTTGGCGTATTACTTATGTTGGTGGGCTCCATTGGTGGGTTCTGGCAAATATTTGTTGAGGCATATGCCTAC

AAATTTTATCCAAACTAA

>Sphfalx0168s0015

ATGGCGATGTCGAATGGAGGGATTGAGAATCACAACGGCAAAGAGAACACCAATCATTTAGTGCAGGGAGGACTCGGGGA

TTCGGTCTCGCAACTCACACATCGCGATCCCGAATTAGCTGATGGAAATCACCATGTTGAGAACTCCAACTCCGATACTC

CAGGCACACGCCTGTCAAGAGAAACAGTCCATCACGTCGGAAAAGACACATGGTGGGAAGTGGGATTCCATCTGATTGCG

GCTTTCGACAATAGCTACGTGCTCGGATATCCTGGGCTTGTCATGGCGTATTTGGGCTGGATCGCCGGGCCAATTTGTGT

GGGAGTTTTTTACGTCGGATCCTTCTACAACAACTACTTGCTGGCTACTCTTCATGAGACTGGAGGGAAGCGGCACATTC

GATCTCGTGATCTCGCAGGCTATATCTTAGGTCCCCTCGTGTACAAAGCGACGTGGATTCTGCAGTACTCGATCTTGAGC

GTTTCAACAATTGGCAGCATTATATTGTGTGGTGAATCTTTGCAGGGAATCTGGGTGGCTTACAGCCACAACCCATCAGC

AATTACACTACCAGTCTGGGTTGTGATATCTGGTGGCACTTATGCTCTCTTTGCTTTTTTTGTACCAACTTTGCATTCAC

TGCGGCCCTTCACAGCTGTCTCAATTTTCCTTTCTCTCATTTTCATCTGTATAGCTATTGGCACTTCTTTCAATGATGGC

TTCAGGGCTGCAGCACCAAGGAATTACTCATTACTGGGCACCAAAGCTGATGTGTCCTTCAGATCAATTGGGAGCCTGGC

CACAATTGCATTTGCATTCAATAGCAACATCCTTCCTGAGATGCAGGCAGTAGTGAGGCAACCAACTGTGAGGAACACAC

ACAAGGCCCTTGTAATGCAGTTCACACTTGGCACCTTCCCTATCATCTTAGTCATGATGGTTGCATATTGGGCCTATGGG

AATACAGTCAATACTTATGTACTGAACTCCACATCGGGTCCCAGGCCTTGGGTTGCACTTGCCAATGTGGCTGCCTTCCT

ACAAATGATCATCTCAATTCATGTCTATGCATTGCCTATGTACGAATTTGTTGACACATTCTTTGGAAGAAACTATGATG

GTAAAGGGGACTGGTCTGGTCACAGTGCATTGATTCGTTTCCTTACACGTGGGACATTCATTGCAATTGCCACCTTCATT

GGGGCTCTACTTCCATTTTTCGGAGACTTTGTTGCTCTGACGGGTGCATTCTCTGTCTTTCCCCTCAACTTCGGGTTGGT

GCATCTCATGTACTTGAAGGTAAATGGTAAGAATTTTATGCCCTATCGAGTTGCTTGGCACTGGATCATGATAATTCTCG

CAATAATTCTTACAGTTGCCACTGCAACCGCATCCATCAGGCAAATAATCTCAGATGCAACCACCTACCATGTCTTTGCT

AATTCTTAG

>Sphfalx0193s0030

ATGGCAATGTCGAATGGAGGAATTGAGAATCACAACGGCAAAGTGGACACCAATCATTTAATGCAGCGAGGAATCGGGAA

TTCGGCCTCGCAACTGACCCATCTTGATCCCGAATTAGCAGATGGAAATCACCATGTTGAGAACTCCAACTCCGATACTC

CAGACATAGGCCCGTCCAGAGAAACAGTCCATCACGTCGGCAAAGACACATGGTGGGAGGCGGGATTCCATTTGATTGCG

GCTTTCGACAACAGCTACGTGCTCGGATATCCTGGGCTCGTTATGGCGTATTTGGGCTGGATCGCGGGACCAATTTGTGT

GGGAGCATTTTACGTCGGATCCTTCTACAACAACTACTTGCTGGCTACTCTTCATGAGACTGGAGGGAAGCGCCACATTC

GATCTCGTGATCTCGCAGGCCATATCTTAGGTCCCCTCATGTACAAAGCGACATGGATTCTGCAGTTTTTGAACTTGAGC

ATTGGAACAGTTGGCAGCATCATATTGTGTGGTGAATCTTTGCAGGGAATCTGGGTGGCTTACAGCCACAACTCAGCAAT

CACGCTGCCAATCTGGGTTGTGATATCTGGTGGCACTTATGGTCTCTTTGCTTTTTTTGTACCAACTTTGCATTCACTGC

GGCTCCACACAGCTGTCTCAATTTTCCTTTCTCTTATTTTCATTTGCATAGCTGTTGGCACTTCTGTCAATGATGGCTTC

AGGGCTGCAGCACCGAGGGATTACTCATTACTGGGCACCAAAGCTGATGTGTCCTTCAGATCCATTGGGAGCCTGGCCAC

AATTGCATTTGCATTCAATAGTAACATCCTTCCTGAGATGCAGGCAGTAGTGAGGCAACCAGCTGTGAGGAACATGCACA

AGGCCCTTGTAATGCAGTTCACACTTGGCAACTTCCCTATCATCTTAGTCATGATGGCTGCTTATTGGGCTTATGGGAAT

GCAGTCAATCCTTACTTACTGAACTCCACATCAGGTCCCAGACCTTGGGTTGCACTTGCCAATGTAACAGCCTTCTTGCA

AATGATCGTCTCAATTCACGTCTATGCATTGCCTATGTATGAATTTGTTGACACATTCTTTGGAAGAAACTATGGTGATA

AACGGGACTGGTCTGCTCACAGTACATTGATTCGTTTCCTTACACGTGGGACATTCATTGCAATTGCCACCTTCTTTGGG

GCTCTACTTCCATTTTTCGGAGACTTTGTTGCTCTGACGGGTGCATTATCTGTCTTTCCCCTCAACTTTGGGTTGGTGCA

TCTCATGTACGTGAAGGTAAATGGCAAGAATTTTGCACTCTATCAAGTTGCTTGGCACTGGGGCATGATAGGTCTAGCAG

TGATTCTGACAGTTACCACAGCAACTGCATCTGTCAGGCAAATAATCTCAGATGCAACCAATACCCATGTCTTTTCTAAT

ACTTAG

>Sphfalx0193s0031

ATGGCGATGTCGAATGCAGGAATTGAGAATCACAACGGCAAAGATGACACCAATCATTTAGTGCAGCGAGGAATCGGGAA

TTCGGCCTCGCAACTGACCCATCTTGATCCCGAATTAGCAGACAGGAATCACCATGTTGAGAACTCCAACTCCAATACTC

CAGACATACGCCCGTCCAGAGAAACAGTCCATCACGTCGGCAAAGACACATGGTTGGAGGTGGGATTCCATCTGATTGCG

GCTTTCGACAGCAGCTACGTGCTCGGATATCCTGGGCTCGTTATGGCGTATTTGGGCTGGATCGCGGGACCAATTTGTGT

GGGAGCATTTTACGTCGGATCCTTCTACAACAACTACTTGCTGGCTACTCTTCATGAGACTGGAGGGAAGCGCCACATTC

GATCTCGTGATCTCGCAGGCCATATCTTAGGTCCCCTCATGTACAAAGCGACATGGATTCTGCAGTTTTTGATCTTGAGC

ATTTCAGTAGTTGGCAGCATCATATTGTGTGGTGAATCTTTGCAGGGAATCTGGGTGGCTTACAGCCACAACTCATCAGC

AATCATGCTGCCAATCTGGGTTGTGATATCTGGTTGCACTTATGGTCTCTTTGCTTTTTTTGTACCAACTTTGCATTCAC

TGCGGCTCCACACGGCTGCCTCTATTTTCCTTTCTCTCATTTTCATTTGCATAGCTGTTGGCACTTCTTTCAATGATGGC

TTCAGGGCTGCAGCACCAAGGGATTACTCATTACTGGGTACCAAAGCTGATGTGTCCTTCAGATCCATTGGGAGCCTGGC

CACAATTGCATTTGCATTCAATAGTAACATCCTTCCTGAGATGCAGGCAGTAGTGAGGCAACCAGCTGTGAGGAACATGC

ACAAGGCCCTTGTAATGCAGTTCACACTTGGCACCTTCCCTATCATCTTAGTCATGATGACTGCTTATTGGGCTTATGGG

AACACAGTCAATCCTTACTTACTGAACTCCACATCAGGTCCAAGACCTTGGGTTGCACTTGCCAATGTAACAGCCTTCTT

GCAAATGATTGTCTCAATTCATGTCTATGCATTGCCTATGTATGAATTTGTTGACACATTCTTTGGAAGAAACTATTGTG

ATAAACGGGACTGGTCTGCTCACAGTACATTGATTCGTTTCCTTACACGTGGGACATTCATTGCAATTGCCACCTTCTTT

GGTGCTCTACTTCCATTTTTTGGAGACTTTGTTGCTCTGACGGGTGCAATATCTGTCTTTCCCCTCAGCTTTGGGTTGGT

GCATCTCATGTACTTGAAGGTAAATGGCAAGAATTTTACACTCTATCAAGTTGCTTGGCACTGGGTCATGATAGGTCTAG

CAGTGATTCTGACAGTTGCCACAGCAACTGCATCTGTCAGGCAAATAATCTCAGATGCAACCACCTACCGTGTCTTTTCT

AATACTTAG

>Sphfalx0257s0017

ATGGCAATGAACAATAATGGAGCTGGAGGTGTTCAAGGGCTCCATCTCATCTCAATGACGCGAGCGAGATCTGGCCGAAG

GCCTGCTGAAAATGATCATGCAGTTGTAAATTTAGGAGCTGTTGCTCCTTTTCCAGCTGATGGGGACTCTTTACCATTTA

CAGTTGGAGACAGCAATCCTGGAAAAATGAAGAATCTTCCACCTTCATCAGCGTCCCCCCATGAGATCGGCCAAGATCCA

TGGTGGGTGGTGGGATTTAATTTGGTAGCTGCCAGTTCCAGTAGTTACATCTTGAGTTATCCTCAACTCATCATGACACA

TTTAGGGTGGATAGCTGGGCCCATTATCATGGTCCTGCTTAATGCTGTCTTCTTCTACAACAATTGCTTATTGGGTAGTC

TTCATGAAACTGGAGGGAAGCGCCAAATTCGAACTCGTGATCTTGTCGGCTACATCTATGGTCGACGAATGTTCACAGCG

GTATGGATACTGCAATTCACCGTCTTGATCGTGTTTTGTATTGGGACGTTCATATGGGCTGGAACAACATTAGAGGCAAT

TTACGCTTCTTACAGCAGTGATCCGAGCAAGATCTCATTGTCGGAGTGGGTCGCCATAGCTGGTGCTTGTTATAGTGTCT

TCGCGTTTGTGGTGCCCACTTTGCATTCGTTGCGACTCTACAGCATCATTTCTCTTTTCCTCACTATGATCATCACCTTC

ATAACCATTGGCATTTCCATCAAGGACGGTGTGAAATCTGGAGTAGCAAGGGACTATTCAATTGTTGGCACCAAAGCTGA

CAAATGGTTCAGTGCAATGGGAGCTCTGTCCGTTGTTGGCTTTGCATTCAACACCGCCATTCTCCCGGAATTGCAGGCTG

ATGTGAGGCCACCCACGGCGCGAAATATTTACAAAGCTCTTGGACTTCAGTACACGCTCGGTGCCATCCCAATCATAGGG

CTCACATTGATTGGATATTGGGCTTATGGTAATGTCGTGAGTGGATTCATACTGGAGTCAAACTCTGGTCCCAAATGGTT

GGTTACAGTCGCCAAAGTAGCAGCATTCTTTCAAATCCTTGTCACCTTACATATCTATGCTTTGCCGATGTTTGAATTCT

TTGACTCGAAGTTGTTGAAGGCAAAAAGGAGCCTCAAACCCAGTGGGAATGTTGAAAATGATGTCCACCAAGCTGGTGGG

CATTGGTCAGCTCGCACGGTGCTAGTTCGCTTCCTCACGCGCATCACGTTCATTAGCATCACAACTTTGCTTGGAGCCAT

GTTTCCATTCTTTGGCGACATTCTTGAACTCAGTGGAGCTGTGATTGTCTTTCCTATTGACTTTGGCTTGGTGCATCACA

TGTATTTGAAGGTGAAGGGGAAGAATTTTTCATTGTACCGACGCACGTGGCATTGGTTAATGATAGTGACAGCCGTTCTT

TTAACGATAGCATCTGTGACTGGAGCTTCGCGAAATATTATTGCAGTTGCATCCACCTATCGCATCTTTCACAGTAATAA

TTAA

>Sphfalx0333s0002

ATGATGAAAGGCGAAGTTGACACTCCCAATCAGCACAAGATTTCTATGCCTGTTCATGAGAATGGCAAGGTCATGAACAA

GGATGGTCATGAGAGCAGCGGAACAGACAACAAGAATTTCTACACTAGTGCAACTAAAGTCGATGAGTTGAATGCGGGTG

CAAAGTTTGTGCTTGAATCCAAAGGGCGCTGGTGGCATGCAGGGTATCATCTTACAGTATCAATTGCAGCTCCTGCACTT

CTGAGTTTGCCATTTGCACTTGATGGGTTGAGTTGGGGGCCGGGGTTTGTTGCACTAATAATTGCAACTGGTGTCTCCTT

CTATGCTTACACTCTTATCTCCAAAGTACTGGAACAAGCTGAATTTGAAGGACATCGCTTCCTTCGATTCCGGGATGTTG

CAGGTTATGTTCTTGGAAGGAGATGGGGCTTTTATCCTGTAGGAGCACTTCAAATTGCTGTCTGCCTTGGTACTGTTGTT

GGCAGTACTCTCCTTGGTGGTGAGAGCATGCAGATCATATACCTCATCTACAAACCAAACGGTTTGATTCAGCTCTATGA

GTTTATCATCATCTTTGGGACCCTGATGTTGCTACTCTCACAGTTACCATCATTCCACTCCTTGCGCTACATCAACTTGG

TATCACTTGTGTGCTGTCTGGGTTACAGTCTTTGTGTTGTTGGTGGCTCCATATATGTAGGTCATTCGAGGCAAGCACCC

AGCAAAAGTTATTCTGTTAAAGGCAGTTCTGTAACCAAGATGTTCACCATTTTCAACTCTCTGGCTATTATCATAACAAC

GTTTGGGAATGGAATCATTCCAGAGATTCAGGCAACTCTGGCTCCACCAGTATCAGGCAAGATGTTCAAGGGCTTGTTGA

TGTGCTATGCAGTAGTTATAGCAACATTCTTTTCTGTAGCCGGTGCTGGATATTGGGCTTTTGGCAATGCATCAGCAGGC

AACATCTTTCTCAATTTTGCCCCAAGTGGTGGGGTTCAGCTCATTCCAAACTGGCTGCTATTTCTGGCCAATATGTTTGT

GATTGCTGAGCTCTTTGCTGTGGCCCTGGTGTACTCACAACCAACATTTGAGATCTTTGAAGGTCGCTCCTCAAGTGTTG

AAAGTGGCAAGTTCTCTAAGCGCAATCTGCTCCCACGGCTCATAATTCGGTCAATTTATGTGTCCTTTGCTACTCTTATT

GCTGCTGCACTTCCCTTCTTTGGTGACATCAATGCTATTATCGGTGCCTTTGGATTCACACCTTTGGATTTTGTCTTGCC

ATTTGTCCTTTACAATGTCACCTTCAAACCCTCGAAACGGACTATAAAATTCTGGCTGAATTATGTGATTATAGTTGTGT

TCACTATTGTTGGCCTTATGGGATGCATTTCAGCTGTCCGCCAGATTGTGTTGGATGCAAGTTCATACAAACTATTTGCT

AATGTCTAG

>Sphfalx0362s0005

ATGTCGAAAGGCGAAGCCGACACTCCCAATCAGCATAAGATTTCTGTGGCTGTTCATCAGAATGGCACGAGCACAGACAA

CAACGATGATCATGCGAGCAGCAGCACAGACAACAACAATCTCTACATGACTAGTGCGAGTAACGTCGATAGGTTGAATG

CGGGTGCAAAGTTTGTGCTCGAATCCAAAGGGAATTGGTGGCATGCAGGGTATCATCTCTCAGCATCTATGGCAGCTCCG

ACGCTTCTGAGCTTGCCATTTGCACTTGATGGGTTGGGTTGGGTACCAGGGTTTCTTGCACTAACAATAGCAACTGTTGT

CTCCTTCTATGCTTACACTCTCATCTCCAAAGTACTGGAACAAGCTGAATTAGAAGGACATCGCTTCCTTCGATTCCGGG

ATGTTGCAGGTTATTTTCTTGGAAGGAGATGGGGGTATTATCCTATAGGAGCAATTCAAATTGCTCTCTGCATCGCTGCT

GCTGTTGTGTGTGTGCTCCTTGGTGGTGAGAGCATGCAGATTATATACCAAATCTACAAACCAAATGGTTCAATGCAGCT

CTATGAGTTTATCATCATCTTTGGGATCCTGATGTTGCTACTCTCGCAGTTACCATCATTCCACTCCTTGCGCCACATCA

ACTTGGCATCACTTGTGTTCTGTCTGGGTTTTAGTCTTTGTGTTGTTGGTGGCTCCATATATGTAGGTCATTCAAAACAA

GCACCTGCAAAAAGTTATTCTGTTGAGGGCAGTTCTGTATCTAAAATGTTCACCATTTTCAACTCTTTGGCTATTATCAT

CCTAAATTTTGGGATTGGAATCATTCCAGAGATCCAGGCAACTCTGGCTCCACCTGTATCAGGCAAGATGTTCAAGGGCT

TGTTGATCTGCTATGCAGTAGTCATATCAACATTCTTTTCTGTAGCGGGTGCTGGATATTGGGCTTTTGGCAATGCATCA

GCAGGCAATATCTTAATCAATCTTGCCCCAAGTGGTGGGGTTGCACTCATTCCAAACTGGCTGCTATTTCTGGCCAATAT

TTTTGTCATTGCTGATCTCTTTGCTGTGGCTCTGGTGTACTCACAACCAATATTTGAGATCTTTGAAGGTCGTCTCTCGA

GTGTCAAAAGTGGCAAGTTGTCTATGCGTAATGTGCTACCGCGGTTCATAATTCGGTCACTTTATGTGTCCTTTGCTACT

GTTATTGCTGCTGCATTTCCCTTCTTTGGTGACATCAATGCTATTATCGGTGCCTTTGCCCTCACCCCTTTGGATTTTAT

CTTACCGTTTCTCCTTTACAATGTCACCTTCAAACCCTCCATCTGGACAATCAAATTCTGGCTGAATTCTGTGATTATAG

TTGTGTTCACTATTGTTGGCCTTATGGGATGCATTTCAGCTGTCCGCCAGATTGTGTTGGATGTGAGTTCATACAAACTA

TTTGCTAATGTATAG

>Sphfalx0362s0007

ATGTCGAAAGGCGAAGCCGACACTCCCAATCAGCATAAGATTTCTATGGCTGCTCATCAGAATGGCACGAGCACAGACAA

CAACGATGATCATGCGAGCAGCAGCACAGACAACAACAATCTCTACATGACTAGTGCGAGTAACGTCGATGGGTTGAATG

CGGGTGCAAAGTTTGTGCTCGAATCCAAAGGGAATTGGTGGCATGCAGGGTATCATCTCACAGTATCTATTGCAGGTCCG

TCGCTTCTGAGCTTGCCATTTGCACTTGATGGGTTGGGTTGGGTACCAGGGTTTCTTGCACTAACAATAGCAACTGCTGT

CTCCTTCTATGCTTACACTCTCATCTCCAAAGTACTGGAACAAGCTGAATTAGAAGGACATCGCTTCCTTCGATTCCGGG

ATGTTGCAGGTTATGTTCTTGGAAGGAGATGGGGCTATTATCCTATAGGAGCAATTCAAATTGCTCTCTGCATCGCTGCT

GTTGTTGCCTGTGTGCTCCTTGGTGGTGAGAGCATGCAGATTATATACCAAATCTACAAACCAAATGGTTCGATGCAGCT

CTATGAGTTTATCATCATCTTTGCGATCCTGATGTTGCTAATCTCACAGTTACCATCATTCCACTCCTTGCGCTACATCA

ACTTGGCATCACTTGTGTTCTGTCTGGGTTTTAGTCTTTGTGTTGTTGGTGGCTCCATATATGTAGGTCATTCAAAACAA

GCACCTGCAAAAAGTTATTCTGTTGAGGGCAGTTCTGTATCTAAAATGTTCACCATTTTCAACTCTTTGGCTATTATCAT

CACAACGCTTGGGAATGGAATCATTCCAGAGATCCAGGCAACTCTGGCTCCACCGGTATCAGGCAAGATGTTCAAGGGCT

TGTTGATGTGCTATGCAGTAGTCATATCAACATTCTTTTCTGTAGCGGGTGCTGGATATTGGGCTTTTGGCAATGCATCA

GCAGGCAATATCTTTACCAATCTTGCCCCAAGTGGTGGGGTTGCACTCATTCCAAACTGGCTGCTATTTCTGGCCAATAT

TTTTGTCATTGCTGACCTCTTTGCTGTGGCTCTGGTGTACTCACAACCAACATTTGAGATCTTTGAAGGTCGTTACTCGA

GTGTCAAAAGTGGCAAGTTCTCTATGCGTAATGTGCTACCGCGGTTCATAATTCGGTCACTTTATGTGTCCTTTGTTACT

GTTATTGCTGCTGCATTTCCCTTCTTTGGTGACATCAATGCTATTATCGGTGCCTTTGCATGCACCCCTTTGGATTTTAT

CTTACCGTTTCTCCTTTACAATGTCACCTTCAAACCCTCGATCTGGACAATCAAATTCTGGCTGAATTATGTGATTATAG

TTGTGTTCACTATTGTTGGCCTTATGGGATGCATTTCAGCTGTCCGCCAGATTGTGTTGGATGTGAGTTCATACAAACTA

TTTGCTAATGTATAG

>Sphfalx0616s0001

ATGTTTTCCAAAGTCTCCAAAAGCCCCTTGGCATCTGCCACTACGGATGAAGATTTCCAGGCATATGTGGAGGAAATTGA

GCCTGCAAAACCAGTCAATGTTGAGGATTGGCTGCCAGTGACTGGGAGCAGAAATGCCAAATGGTGGTATGCAGCATTCC

ATAATGTGACAGCTATGGTGGGTGCGGCAGTTCTTAGTCTGCCTTATGCTATGGTTTACCTTACCTGGGGTCCAGGAGTG

ATAGTGTTGGTGTTATCGTGGGTCATCACACTCTACACCTTGTGGCAAATGGTGGAGATGCATGAGATGGTGCCAGGCAA

GCGCTTTGATCGCTACTATGAATTGGGTCAGGAAGCGTTTGGGGAGAAATTGGGACTCTGGATTGTGGTACCTCAGCAGC

TCATCGTAGGCGTAGGCGTGGACATTGTGTATATGGTAACTGGTGGCAAATCCTTGAAGAAGTTCTATGAACTTACTTGC

AAGAAGAATTGCTACTTGCAAAATCGACTTTCTATCTGGATTCTTGTGTTTGGGTCTGTGCACTTGTTTTTGGCTCAACT

ACCGAACTTCAACTCCATTGCGGGAATTTCATTAGCTGCAGCTATCATGTCACTCAGCTACTCAACAATTGCATGGGCCA

TCCCTGCCCATTATGGACACTCCTTGCTTCCTCCCGGGCAGAGCCCAGACTATCATTTGCCACCAAATCAGTCAACAGCA

GCACTGGTCTTTGGTGCTTTCAATGCATTGGGAACAGTAGCATTTGCATACGCTGGCCACAACATCGTGCTGGAGATACA

AGCCACAATCCCATCAACTCCTCATCGGCCATCAAAAATTGCCATGTGGAGAGGAGTCCTGGTTGCTTATGGAATTGTGG

CTGCATGCTACTTCCCTGTTGCTATTGTTGGTTACTGGGCATTCGGTAACCAGGTACAAGACAACATCATCACGTTTGTG

GCCAAACCCGTGTGGCTGGTTGCCATTGCCAATCTCATGGTTGTCATTCATGTCATCGGAAGTTATCAGATCTATGCAAT

GCCAGTGTTTGATATGCTGGAAACAGTGCTTGTGAAGAGATTCCATTTTTCTCCTTCTCTGCAACTTCGGCTCATTACTC

GCTCCATCTATGTTGGTTTGACAATGTTTGTGGGCATGACCCTTCCGTTTTTTAGTTCCTTGCTGGGATTCTTTGGAGGG

TTTGCCTTTGCCCCAACAACATACTTTTTGCCTTGCTGTATGTGGCTGACTATCAAAAAGCCAAGAATTGGAAGCCTATC

GTGGCTTGCTAATTGGGCGTGTATCATACTAGGTGTCATGCTAATGCTGGTATCCTCCATTGGTGGACTTCGACAAATTA

TCGTTGATGCCTCCAGCTTCAAGTTCTATGAATGA

>Pp3c3_11320

ATGGATCAGGCGGTCAAAATGGCAGGTGAATACGGCACAACAATGAGTCCTGAAGCTTGTGGAGGAGAAGACAAAGCAAT

CTATGGCACCGCGATCATCAAGGATGGTGGAGCACTGTTTGTGCTGGAATCTAAAGGGAACTGGAAGCATGCTGGCTTCC

ATCTTAGTACATCTATTGTGGCTCCTGCACTTCTCAGCCTTCCCTATGCAATGAAGGGCTTGGGATGGGCACCTGGCTTT

CTGGCTCTTATCATCGGAGCTGTCGTTTCCTTCTACGCTTACATGCGGATTTCCAAGGTACTCGAGCAGGCAGAATTGGA

AGGTCATCGCCTCCTCCGCTTTAGGGATATGGGGGGTTATGTCCTAGGACGTACATGGGGCTACTACCCTGTCTCCGTCC

TTCAAATTGGTTTGTGCTTGGGCGCTATGATCGGTTGCATTGTCCTCGGCGGCCAGAGCATGAAGCTGATTTACAAGGTG

TTTCATCCCAACGGCTCAATGCAACTATACGTGTTCACGATTATCTTCGGGATGGTGATGGCGGTGTTTTCGCAGCTGCC

CTCTTTCCACTCTTTACGCTACATCAACTTGTTGTCTCTGCTGTGCAGCCTGGGATACAGTCTTAGTGCAGTTGGAGGTT

GCATCTATGCAGGCCACTCAAACGAAGCGCCACCCAGGGACTACGCAGTTGTAGGATCTCCAGGTTCCAAAGCGTATGGT

GTGTTCAACTCTCTGGTGATCATTGCCACAACCTACGGTAATGGCATCATTCCCGAGATTCAGGCAACGTTGGCTCCACC

TGTTACAGGAAAGATGTTTAAGGGTTTGCTTGTCTGCTACGCAGTCGTTATAACCACGTTCTTCTCCGTGGCAGCAGCTG

GATATTGGGCATTCGGAAATGAAGCCCAGGGAAATATTTTCATTAATATTGAACCTTTTGTTCCCAAGTGGCTCAACTTC

CTGTCTAATGCCCTCGTGCTTGCACAACTCCTGGCTGTAGCCTTGGTATACGCACAGCCGACCTTCGAAATCTTTGAGGG

CAAATCGTCAAATATACAGAAAGGCAAATATTCCGCCCGAAACCTTGTCCCACGTCTCATCCTCCGATCTGCACTTGTGG

CTATTACCACACTCATAAGTGCAGCTATACCATTCTTTGGAGACATTAATGCCGTGATCGGTTCCTTCGGCTTTACCCCT

CTTGACTTCGTCCTGCCATTCATCTTGTATGCTGGTGTCTTCCATCCCTCTCCTAGAACGCCCAAGTATTGGCTCCACTG

GACTATCGTCATCGTATTTAGCATTGTTGGGCTACTGGGATGCGTCGCCTCCGTCCGTCAAGTAGTTCTCGTTGCCAGCA

CATACAAATTATTCGCTAATATAGTTTGA

>Pp3c6_1540

ATGAGCACTTCAGGTAGGAAGGGCTCGATCGCCATGACGGAAAGAAGTCGTGCTCTTGTAGAAACGACAGCGTTATCGAC

ATCTTCGAGCCCTGATGGAATGGCGCCACCAGTGCAGCCCATTGAGAGACCTTTTATCGCTGCTCCTCCTTTCTCGTTTC

CTACAGCTTCCGACACACCAACAGAGGCTTCTTCTGTGACAATGACGCCGGAGTCCAGCTGCGACCAAACAAATTTTGGG

TCCAACCTTATAGCGTCGCCATGGCGAAGTCCCACAAATAGAGCTGCGGGCAGACCGCCGTCGAATTTCGTCTCACCGAT

TGGGACACCCCTTCACAGGAGCCTGCACAATCTGCAACATTACCTCGAAGAAGGTGGACATTCCACAACACTCCATGTGA

GAGACACATGGCTTCCACTGACCGAGAGCCGCAATGGAAATATGGTATACGCAGCATTCCACAATTTGAATGCCATGATA

GGGTATCAGGCTCTATTCCTCCCATTCGCCTTCATTTACCTTGGCTGGACATGGGGCTTGACTGTATTATGCTTGGCTTT

CACGTGGCAAATGTACACAAAATGGCAACTTATCATGCTGCACGAAACCGAACCTGGAAAGCGAATCCGGAACTATGTTG

AACTTTCGCAAGAAGCTTTTGGGCAGACCATAGGGTTTCACACGACAATTCCTGCTGTACTGAACTTGACTGTAGGGACC

TCCATAGGTCTTGTGGTTGTGGGAGGGAGCGCACTAGAGCTTTTCTACCTGACTGTCTGCCACAAGTGTGTCGATAATCC

TCTGTCAATGATCGAGTGGTGCATCGTATTCTCTGCTCTTTGTCTTATCTTAGCGCAATTGCCGAATATGAACTCCATAG

CCAGTGTTTCACTAGCTGGAGCTCTCATGGCTGTCTCATACACTACCCTAATCTGGATGATCTCCGTCTTCAAAAAACGA

CCCCAAGACATCTCTTATTCATTGGCAACGAAGGGGGATAGTCCGTTAGTGACAACAGTTGCCGTCTTAAATGCAATTGG

GATCATAACCTTCGCCTTCCGAGGCCATAACCTCGTTCTTGAAATACAGGGAACGCTGCCGTCTACGTTGAAAGAGCCAT

CGTCTATATCTATGTGGAAGGGAGCAAAACTTGCAAACTTGGTTCTGGTCTTTTGCTTTTTTCCATTAGCAATTGGTGGT

TATCGTGGATTTGGAAATAAGATGCTCAATTCAGGAATCTTGTACTCACTTCAAGCTGCGGATTTGTCCAAAACTGCGAG

AGGATTCCTTGCTTTAACCTTCCTCTTTGTAATGTTCAGTTGTCTGAGCTCCTTCCAGATCTTCTCCATGCCAGTTTTTG

ACATGATCGAGCAATTCTACACAGGAAAATGGAACAAAAAGTGTAGTCCCTGTGTGAGGTTATTCTCTAGATCGGTGTAT

GTTCTAGTGGTCTTCTTCATGGCGATTGCATTTCCCTTTTTGACAAGCCTGGCTGGCCTTATAGGAGGCCTCAACTCCAT

TCCTGTTACTTTTGTCATACCCTGCTTCATGTGGCTAAGTATTCGACGACCTAACAAGCGGAGTTTCACCTGGTGCCTTA

ACTGGTTCTTGGCAATCTTTGGGATAATCACAAGTTGTCTTGTCAGTGCTGCAAGCGTAGGTGTTATCATCCAAAGAGGG

ATTAAGCTCGAGTTTTTCAAGCCTCATGCCTGA

>Pp3c8_19000

ATGCGTGCTTTCGAGGTGATTGGGACGGGTTACAGCAGTTTGGTCAGAGATCGGTCTGCTGTGGAGGAGGAGGAGGGTTT

CGAGGCGAAGGATGCAGGGGCGCTGTTCGTGCTCGAGTCCAAGGGAACATGGTTTCATGCAGGGTATCATTTGACCACAG

CTATAGCCGGGCCATCGTTGCTCACGTTGCCGTATGCTTTCCATTTCTTGGGGTGGGGCCCTGGATTATTCGCGCTCACC

ATTGCTGGGGCTGTATCAAGTTATGCGTACTGCTTGTTATCCAGGGTGTTGGAACATTATGCCTCTCAAGGGAAACGGTG

CCTTCGGTTTCGAGATCTATCCGACGTTGTGATAGGGAAACGATGGACGATTTGGTTTGTCATACCCGTGCAGTTTGGAG

TATGTTTTGTGACGCTAATCGGAGTTATCTTAACTGGTGGCTATGGTTGCAAGCTCATCTATCTGGGACTAGTTCCGGAT

GGCGCAATTCGCTTGTGGGTATTTGTAGCCTTATTTGGTGCAGTGATGATGATTCTTGCACAGCTGCCGTCTTTCCATTC

ACTGAGACACTTGAGTCTCTTCTCCCTCTTCTGCTGCTTAGCATACAGCGCATGCGCCGTGATCGGATCTATCATTGCAG

GTCATAATCCAAATGTACCCCCGAAAAACTACAGCGTAACAGGTAGTCCTGTTCAGAAAGTGTTCGGAGTCTTCACAGCT

ATATCCATCATGGCTGGAGTATATGGTGTGGCTCTTATCCCTGAAATACAGGCAACAGTGGCGCCTCCTGTAACAGGGAA

GATGCAGAAAGGTATCGCCTTATGCTACACAGTTGTGTTGATCACCTTCTACCCTGTTGCAATCTCTGGCTACTGGGCAT

TTGGTAACCAAGCATCAGGCAACATCGTCGACAATCTCGCCCCTGACAAAGGTCCCGATCTCTTGCCGACATGGCTGTTG

GGCATCTTAAGTATCGCCATTGTTGCACAGCTTCTCGCCATTGGTTTGGTGTATTTACAACCCATCTCTGAAGTGCTGGA

AAGCAAAACTGGAGATGCAAAACAAGGGAAGTACAGTATCCGAAATGTAATGCCTCGTCTTGTCTTCCGGTCTCTTTACT

TGGCAGTGGTAACGCTCTTGGCGGCGATGCTTCCATTCTTCGGAGACATCATCTCCCTGATTGGAGCGTTTGGGTATACG

CCGCTTGACTTCGTCCTGCCCATGCTGTTCTACCAGATTGTGTTCCAGCCATCGAGACAGAAACCAATCTTTTGGTTGAA

TTGGACGATTATTATAGTGTTCACGGTCGTTGGGGTGATTGGGTGCATAGCTTCCTTCCGCAGCATCTACATGAATGTGC

AAAAGTATCATTTGTTCGGGGATGTATGA

>Pp3c9_4450

ATGGCGTTCTCCGGGGCAGATGCAGATGAAGTTGCGGTAACCACCGCAGAGCAGCCACCTTTTTTCCGGGTCGCATCCTC

AACCGAGACGGCTCACGATCAGGAAGCTGCCCGCAGTAGCGACATAATACCCCTCTCCAAAGAAACATTTCACCGCGTGG

GCGAAGACACTTGGTACGAAGTGGGATTCCACATCATTGCTGCGCTAAACACAGCGTTCATCCTTGGTTACCCGGCGCTG

ATTATGGGAATTCTGGGCTGGATTGCTGGACCCATCTGTCTAGTCGGAGGCGCTGTGATCTCATTCTACAACAACTATCT

GTTAGGTGGCCTGCACGAGACTGGCGGCAAGCGCCATGTCCGATACCGAGATCTCGCAGGCTACATCTATGGGCCCACCA

TGTACAAACTTACATGGGTAGCGCAGTTTTTGTGTCTCATCGTCATCAACATCGGAACGATCATTCTGGCCGGGCTATCT

CTGAAGAGCATGGCCAGAGCATTCTCAGACGGCTCGGAAATAGTGAAGCTGCCAGGTTGGATTGCAGTCACAGGCGCTGT

GGTTTGCATTTTCGCGCTGATGGTCCCAACCTTGCACGCGCTGCGGTTCTTTTCCACCTGCTCCTTGCTCTTGAGCAGCA

TCTACACGTTCATAGCCATTGTCGTCGCCTTTAAGGATGGATTGAAGGCTGAAGGTCCTCGAGACTACTCTCTGAGGGGG

AACGTCACTGACAGGACTTTCAACGCAATCGGGGCGCTGGCAACTATCGCCTTCGCATTCAACACTGGAATCCTTCCGGA

GATGCAGGCAACAGTGCGGCAGCCAACAACGAGGAACATACGAAAAGCTTTGGGATTGCAGTTCACTGTGGGCACTTTCC

CAATCCTTGTGCTCACTTTTGTTGGGTACTGGGCTTATGGCAACACAGTCTCGGTGTACATGTTCAGCTCCGTGTCCAGG

CCCAGATCCACTGCAGTCACTGTCGCGAATGCAGTCGCTTTCCTTCAAGCTATAATAAGTCTTCATGTTTATGCCAGTCC

AATCTATGAGTTCATGGACACGCAGTTTGCAAGGAAGGGGGATCATGAGTGGTCGCGTCACAGCGTGCTTGTGCGCTTCT

TCACTCGCACCGCATACATTGGAATAAGCACTTTCTTGGGGGCCTTGCTGCCCCTTTTTGGCGACTTCATCGCTCTTACT

GGAGCACTCGTGGCGTTCCCTCTTGAATGGGGCTTGATACACCACATGTATTTGAAGGTTAAGGGCAAAGAATTCGGGAA

GGGCCGACTATTATGGCATTGGAGCATGATTGTGATTGCAGTCGTCCTGACCTTCACAACCGCCACAGCTGGTTTGAGAT

TCATCATCTCCGACTCAATTCTTTATCATGAATTTGCCGACTTGTGA

>Pp3c11_19940

ATGCAACGGGGCAGCAATATGGACAAAGTTTCAAGTATCGAGGGTGAGAAGAAGTTCAAGGGCGATGTCGACGGCCCCGG

ATCCTTACCTGGGTCTCTTGTGAAGCATCCGGAGGGTAGAGGGACATGGCCGATTTCAGCTTTCCATCTGGCCACCACCA

TCGCCACTCCTGCAGCCTTCGCTCCCTTGCCCTACGCCATGTCGCAGTTGGGTTGGATAGGAGGTGTGGTGACATTACTG

GTGGGGACGGCCGTCACTTACTACTGTACTCTCCTGCTGGCGAGCTTGTGGGACTGGGATGAGCCTAATCGATATGTTCG

CTACCGAGACCTTGGGCGCTCCATCTACGGAGCGAAGGGGTACTGGTCCGTATTGGCTTTTCAGCAAATTGCGTCCATAG

GCAACAATATTACCATTCAGATTGTGGCTGGGCTGAGCATGAAGTCGATCTATACCACTTACTCGAGCAACCCGAGCGGC

ATGACGCTGCAGCACTTCATCATTCTTTTTGGGGTGGTGGAGCTCTTCCTGTCGCAGTTTCCGGACATCCACTCTCTTCG

CTTTCTTAATGCTCTCTGCACTGGCTGCACCATCGGCTTCTCTGTGTCGGTGGTGGCCCTCTGCGCCCATGCTCTAAGAA

ATGGAGACGCAGACGGTTCGAGCTACGACATAGTTGGAAGTCCATCTGACAAAACTTTCGGCATTTTCGCGGCCCTCGGC

ACAATTGCCTTCTCGTTTGGCGACGCCATGCTGCCCGAAATTCAGGCAACACTTCGAGAACCAGCCAAGTTGAACATGTA

CAAGGGTTCCACGCTTGCATACACAGTGATTGCGGTTTCGTACTGGACAGTGGCCTTCATGGGTTACGCTGTGTTCGGAA

ACACGGTGAATCCGTATCTAGTCAATTCATTTTTCGGACCTGATTGGCTCATTACGTTAGCCAACATTTTTGCTATAATC

CAAGTCCTCGGTTGCTATCAGATATACTGCCGTCCAACGTACCTTTACGTCGAGCAACAAGTGATGGACTACAACAAACA

CCCCTGGAGCCTCCAGAACGCTTTGGCAAGGGTGGGAGTAACCGCAACCTACATTGTCGTTATCACTGTGATTGCAGCAG

CAGTTCCATTCTTCGGCGACTTCGTTGCACTTTGTGGAGCAATTGGATTCACTCCTTTGGACTTCATCATCCCTGTCATT

GCATTCCTCAAGGTGCGAAACCCGAAGAACCCACTTGTGAAGCTCATCAATGTGGCCATTGTGGTGGTGTACTCCATTGT

GGCCATTCTGGGGGCCATCGGCGCCATCCAGTTCATTCATCACGACACCAACCGCTACCAATTTTTTGCAAACCTGTAA

>Pp3c13_12390

ATGGCCAACCCATCCACGCGAGAAGCGTTCCAGGAGCACATGCAGGAGAATGGACACAAGAAAGTGAAGACTGTCGACGA

GTGGCTGCCTGTGACCGGCGATCGCAAAGCCAAATGGTGGTACTCCGCCTTCCACAATGTTACCGCAATGGTGGGCGCCG

GAGTGCTGGGATTGCCCAACGCCATGGTCTACTTAACATGGGGACCGGGAGTGGTGGTGCTGGTGGTGTCGTGGATGATC

ACGCTGTACACGCTCTGGCAGATGGTGGAAATGCACGAGATGGTCGAGGGGAAGCGTTTCGATCGGTACCATGAGCTCGG

ACAGGAGGCATTCGGACACGATCTGGGCTTGTGGATCGTAGTTCCCCAGCAATTAATCGTCGAGGTCGGTGTCGATATCG

TGTACATGGTTACAGGCGGAACGTCACTGCAGAATTTCTACAAGCTTGTGTGCTCAGGCAACTGCCCGATGGCACACCAC

ACAAGTGCCTGGATCGCAATCTTCTCGTCAGTGCATTTCGTTCTGGCGCAGTTGCCGAACTTCAACTCCATTGCCGGGGT

CTCCTTGGCTGCCGCTATCATGTCCTTGAGTTACTCGACGATCGCGTGGGCCATTCCAGCTTCCTACGGCCATAGCACAC

CCCTCGTCGGCCCCGTAAACTACAAGCTCCCGGTTCAGTCTGTGTCAGCGCATGTATTCAATGCCTTCAACGCCCTTGGG

ACCGTCGCATTCGCTTACGCAGGCCACAATGTCGTCCTGGAGATTCAAGCCACGATCCCCTCCACGAAGGAGCGGCCCTC

CAAGATCCCCATGTGGAGAGGCGTGGTGCTGGCCTACATCATCGTTGCCATCTGTTACTTCCCCGTCGCGCTCATTGGCT

ACTGGGCTTACGGCAATCAAGTCACAGACAACATCCTCGGTTATGTCGGTAGGCCGAGGGGGGTCGTGGCCATGGCGAAC

CTCATGGTTGTCGTTCACGTTATTGGAAGCTACCAGATCTACGCCATGCCTGTTTTTGATATGTTAGAGTCTGTTCTTGT

GAAACGCTTCCGGCTTGCTCCCTCCAGGAAGCTACGGCTCGTCACTCGCTCGCTCTACGTCGCTTTTACTGCGTTTGTCG

GTATGACGTTCCCCTTCTTTGGAGCTCTTCTTGGTTTCTTCGGAGGATTCGCCTTCGCTCCTACAACATACTTTCTGCCG

TGTATCATGTGGCTGTGCATCGTCAAGCCCAAGGCATTCAGTTTCTCGTGGATTTTGAACTGGGTCATCATATTCCTGGG

AGTGCTGCTCATGCTGGTTTCGTCCATCGGAGGTTTGAGGGCGATAATTGTGTCCGCATCAACCTACAAATTCTACGAAT

GA

>PpAAP9A

ATGGACGGGGACCATGTCATTCTTGGGCTCACTGGAAACGCCAAACTTGGGCAGACAGATGACAATGCTCTAGGGCATGG

CGAAAAGGGCAGAGCTGGTTTTGACGGCGTGGACAAGCCTATTCATGATCCCAACCTGAATGATGACGACGGAAAACCTC

GCCGCAAAGGGACAGTCATTACTTCGGCAGCCCATATTATAACGGCTGTCATAGGTTCTGGTGTTTTGGCGTTGTCATGG

AGCTTCGCACAAATGGGGTGGATTGCAGGACCAATTGTCCTTCTTGCTTTTGCTTGGTGTACCTACTACACCTCTCGACT

CCTCGCTGATTGTTATCGGTCCCCCGACCCTATCCACGGGAAGCGTAACTACATATACATGGATGCTATCAAAGCTAATT

TAGGGAGAAAGCAGCAGTTGGTTTGTGCTTGTGTTCAATATAGTAACCTTATTGGCACAAGTATTGGCTACACTATTGCC

ACTGCAACCAGTGCCAAGGCAATTCAGTATCAGAATTGTATTCACGACAATGGACCTGACGATCCATGCCTGACGTCAAC

AACAGTTTACATTGCGATATTTGGAGTCATCCAGATAGTCTTATCACAAATTCCAAATTTTGGCGAGCTTTGGTGGCTTT

CTTACCTTGCTGCTGCTATGTCGTTCACATATTCCTTCATTGGGTTGGGACTCGGTATCTCAAAAGCTGCAACGGGAGAG

AATTCTCATGGAAGTTTGGGCGGTACATCCGTTTGCTATCCTTCAAATGGTGAAACATGCTTCACACGACCACAAAAGAC

GTGGAATGTGTTCACCGCTCTTGGAAATATGGCTTTCGCATACTCTTTCTCCATGATACTTATCGAGATTCAGGACACAA

TAAAAAGTCCCCCTTCTGAATCCTCTCAGATGAAAAAAGCCACGTTACTTGGAATCATAACCACGACATTTTTTTACATG

TCGGTGGCCATAGCTGGGTACGCGGCATTTGGTGATGCTGCACCAGGGAATTTGCTCACTGGATTCAGTACTCCATATTG

GCTTGTGGATTTTGCTAACACCTGCATTGTGATCCATCTTATCGGTGCCTATCAGGTTTACACTCAACCTGTATATGCCT

TCGTGGAGCGATGGTGTTCTCTGCGATGGCCCAACAACTCATTTCTCAACCTGGAGTATAATGTTAGACTGCCAGGAAGG

CGCAATTTCAGAGTGAGCGCATTCCGCTTGATCTGGCGTACCATTTACGTCATTATCACCACCATAATTTCAATGTTAAT

CCCATTTTTCAACTCTGTTTTGGGAATCCTTGGGGCCATTGGGTTTTGGCCATTGACAGTTTATTACCCGGTGGAGATGT

ACATTAGGCAAACGCACGTGCAGAGATGGAGCCATTTTAATAGGAGATTGCAGACGAGCATTTTATTCAACCTGCTCGTG

CTACTGTGTGATGGGTATTATGAGGGCGGTTGA

>Pp3c14_9480

ATGACTTTGGGAAGGATGGATTCGTCGCTCGACACTCACAACATCGAACTCCAAAAGCAATCTTCCGTTCTGCTGGCACC

ACCTCAAAGGAGTGAAAACAGCCAGAACACAACCGACCTGGAAGCATGGCTACCCATCTCAACCGCAGACCGCAATGCCA

ACTGGAAGCATGCGGCTTTCCACAACGTCACGGCGATGATGGGAGCAGGAGTTCTCGCTTTGCCCAACGCCATGGTCTAC

CTTACATGGGGTCCAGGTCTCCTAATGCTCATCCTTTCATGGGTCATCACCCTCTTCACACTCTGGCAAATGGTGGAGAT

GCACGAAGCTGTGCCCGGAAAGCGATTCGACAGATACCATGAGCTTGGTCAAGAAGCGTTTGGCCCCAAACTGGGGCTAT

GGATCGTGGTCCCGATGCAGTTAGTAGTCGAGGTTGGCGTGGACATTGTTTACATGGTAACAGCAGGGAAATCCATGCAG

CACGCGTACAACATCACCTGCGGCGACCACTGCCCATTGCAGGACGCCATCGTCTTCTGGATCTTCCTCTTCGCCATCGT

CCAGCTTGTGCTCGCTCAGCTACCCAACTTCAACTCCATAACTGCCATTTCGTTAGCTGCCGCCATCATGTCCATTAGCT

ACTCTACCATAGCGTGGATCATTCCTGCTCATTATGGGCACACCCTTCCGGGCGGCGTCAAGCTACTGGCACCGGTAAGC

TATGGCTTGCCGCAGTTCAAGCCTGTTCAACTGCAGTTGCCAGGCGGTTCTACCCTACAGCAAGTTCCAGATGATTTATC

CTACAATGATCGGTTGTTTGGAGCTTTCACTGCCCTTGGCACAATTGCGTTTGCCTATGCAGGGCATAACGTGGTGTTAG

AGATTCAGTCCACTTTGCCCTCCACTCCAGAGGAGCCATCCAAGCTTGCTATGTGGCGAGGAGTGAAATTTGCTTACGGA

GTTGTGGCAGCCGGATATTTTCCAGTTGCATTAGTTGGGTATTGGGCTTACGGCAACCAAGTGACGGACGACATTATCAC

ATTTGTGTCACGGCCAACTTGGTTGGTGCTGATTGCCAATCTGATGGTGGTTGTTCATGTCATTGGGAGCTACCAGATTT

ATGCCATGCCGGTTTTCGACATGATGGAATCAACTCTGGTGGGCCGCTTGAGGTTCAAACCATCAACACCTCTTCGGCTC

ATCACCCGCTCTCTCTACGTCGTCTTCACCATGTTCATTGCCATCACGTTCCCATTCTTCTCAGCTTTGCTGGGCTTCTT

CGGTGGCTTTGCTTTTTCGCCGACTACTTATTTTTTGCCGTCCATCATATGGTTGAGGATATACCATCCAAATCGCTGGA

GCTGGTCATGGGTCATCAACTGGGCGGTGATTGTATTTGGCGTGGTGCTGATGTTTGTGTCGACCATCGGTGGTTTCCGA

TCCCTGATGGTCGAAGCAGCCAATTTCCACTTCTATAAGAACTAA

>Pp3c17_11220

ATGGATGTGCCGCTCGATACTTACCATATCGAGCTCCAGAAGCAATCCTCCCTTCTCTTGGCACCTCCACAACGAGGCGT

CAGCGGTGATCGCTTGATTGATTTGGAATCATGGCTACCCATCACTGCGGCAGACCGCAGCGCCAACTGGAAGCATGCGG

CTTTCCACAACGTGACGGCCATGATGGGAGCGGGTGTTCTCGCTTTGCCCAATGCCATGGTCTACCTCACATGGGGTCCA

GGTATCCTAATGCTCATTCTGTCATGGATCATCACCCTCTTCACTCTCTGGCAGATGGTGGAAATGCACGAAGCTGTCCC

GGGTAGGCGGTTTGATAGATATCATGAACTAGGCCAGGAAGCATTCGGTCCCAAATTGGGGCTATGGATCGTGGTCCCGA

TGCAATTAGTAGTCGAGGTAGGTGTGGACATCGTTTACATGGTGACCGCAGGAAAATCTCTGCAACACGCTTACTCCATC

ACCTGCGGCGACCACTGCCAGTTGCAGGACAGCATTGTCTTCTGGATCTTCCTCTTCGCCATCGTGCAGCTTGTGCTAGC

CCAGCTGCCCAACTTCAACTCCATAGCTGCCATCTCGTTAGCTGCCGCCATCATGTCCATCAGCTACTCCACCATAGCCT

GGGCCATTCCTGCTCACTATGGCCACACTCTCCCCGGCAACATCGAGCTACTGCAGCCGGTAAGCTACGACTTCCCGCCG

TTCAAACCTGTCCAGCTGGTGCAAGCGGGAGGTTCCGCTGTACAAAAGGCTCCAGAAGATTTGTCAACAGCAGACCGGTG

GTTCGGAGCTTTCACTGCCCTCGGAACAATCGCATTTGCCTATGCAGGGCACAACGTGGTATTGGAGATTCAATCCACGT

TGCCTTCCACTCCACACGAGCCATCCAAGATCGCCATGTGGCGAGGGGTCAAATTTGCTTACGGAGTTGTGGCAATCGGC

TATTTCCCTGTTGCGTTGATTGGGTATTGGGCTTACGGTAACCAAGTGACGGACGATATCATCACCTTCGTCTCACGGCC

AACCTGGTTAGTGGTCATCGCCAATTTGATGGTGGTCGTCCATGTGATCGGAAGCTACCAGATCTATGCCATGCCAGTTT

ACGACATGCTGGAATCAACCCTGGTGGGGCACTTGCGTTTCAATCCGTCGATGCTTCTTCGACTCGTCACCCGCTCTCTC

TACGTCAGCTTCACCATGTTCGTCGCTATGACGTTCCCGTTTTTTGCTGCTTTGCTGGGCTTCTTCGGAGGATTTGCATT

CTCACCGACCACGTACTTTTTGCCTTCCATCATGTGGCTAATGATATACCGGCCGAGTCCCATGAGCTGGTCATGGATCA

CAAACTGGGCGGTGATCGTATTCGGCGTGGTGTTGATGTTTGTGTCGACCATCGGTGGTTTCCGATCCTTGATGACTGAA

GCAGCCAATTTCCACTTCTACACATAG

>Pp3c21_14080

ATGGCATATGCGTGGTACACAGTGGCATTCCACATCGTGACAGCTTTAAATTCGGGTTTTATTCTTGGGTACCCCGCTCT

GATCATGGCGTACCTGGGCTGGACTGCCGGTGTCCTCTGCCTGCTCGGAGGGGGTATCATCTCTTTTTACAAAAATTGTC

TTCTCGGAGAGCTGCATGAGACCGGCGGCAAGCGCCAAGTTCGCTACCGGGACCTTGCAGGCCACATCTACGCTGCAGCC

TTCACAGTTGGAAGACATGTGACGTTGCCTGGATGGGTGGGCGTCGCCGGAGCAGTGATATGCGTCTTCGCATTTTTGGT

TCCCACCCTTCACGCATTCCGGTTCTTCTCCACTTGCTCTTTGCTCCTCAGCTGTGTCTACATATTCACCAGCGTCGGCA

TCGCTCTCACTGACGGTGTGAAGGCGAAGTTTTCTCGAGACTACTCTCTGAAAGGTAGTAACACTGAAAAGGCGTTCAAT

GCATTAGGAGCCATGGCAACAATAGCATTTGCATTCAACACGGGCATTCTTCCAGAAATGCAGGCAACAGTGAAAGAGCC

ATCAGTTAGGAACATGAAGAAGGCACTTGACCTTCAATTCACAGTGGGGACACTCCCAATCCTGATGCTCACCTTTGTTG

GATACTGGGCATACGGCAACGACGTCGTCCCATACATGTTGAATTCTGTCTCTGGGCCCAAATCTGCAGTTACAGTCGCT

AACGCCGCTGCCTTCTTGCAAACGGTCGTTTCTCTCCATATTTATTGCTCGCACATTTACGAGTTCATGGATACCTCCTT

CAGTAAGAAAGGACGCCATGAATGGTCTTTCTATAGCATAACCGTGCGGTTGATCAAGCGCACGACGTATATCTCTCTCA

GCACTTTCCTGGGAGCGTTGCTTCTCTTCTTCGGAGACTTCATTGTTCTCACCGGAGCAGTGGCTGTGTTTCCTCCAGAG

TCTGGATTGGTTCACCACATGTATACAAAGCGACTGATTTGGCACTGGGGTATGGTGATCATCTCGGCAGCCTTGACTGT

TGGAACCGTCGCTGTGGGTTTCCGATTCATTGTTGTTGACTCTATCAACTACCCTGCATTCGCCGACTTGTAA

>Pp3c23_12700

ATGAATGGGCATCAAGGTTATACGAGATTGGAGGGCTCTTCCGAGGTGGAAGCTTTCAGTTCACGTCCTCAAATGCCATC

TAAAGACGTTGATGGGGGCGCGCTCTTTGTTCTTGAATCCAAAGGAAACTGGAAGCACGCTGGCTTCCATTTGACCGTGT

CCATAGCCACCCCTGCGTTGCTTACCTTACCCTTTGCGCTACGAGAACTCGGGTGGGTGGCTGGAGTTTTAGCACTCGGT

CTCTGCGCAGGTGTTTCATTCTATGCCTACAATATTCTCTCGCAAGTTCTCGAGAATTCTGAACGCCGGGGCCATCGGTT

TCTCCGATTCCGTGATCTGGGAGCACATGTTCTAGGGCCATGGGGCTACTACGGTATTGGCGGGATTCAGTTTCTTGTAT

GCTTCGGAACGGTCATCGGTAGCTGCATCGTTGGGGGCCAAAGTATGAAGTTGATTTATAGCATCCTTGAACCGGAAAGC

ACAAGACAACTCTCCGAATTCGTTGCTATCTTCGGGATTTTCATGTTGGTGCTCGCTCAATTGCCTTCCTTTCACTCGCT

ACGTTATATCAACCTGGCATCTTTGATGTGTTGCTTGGGCTTCTCTCTGTGTGTTGTGGGAGGTTGCATCTATGCAGGCA

ATTCTGTTGATGCCCCACCAAAAGACTACTCTATATCCGGCACCCCTGCTTCCAAGCTTTTTGGCGTCTTTGAAGCACTT

GCCATCATCGCTACTACATTTGGAAATGGAATTATTCCGGAGATTCAGGCAACATTGGCTCCACCGGTCGAGAATAAAAT

GTTCAAGGGCTTGTTGGTTTGCTACACAGTGGTGGTCACAACTTTCTTCTCTGTCGCCATATCTGGATACTGGGCCTTCG

GAAATCAAGTTGCAGGCTACGTCCTGACCAACCTTGCTCCAACTGATGGACCCGCTCTAGTCCCAAGCTGGCTTATCCTC

TTGGCCAATGGATTTGCGCTTGCGCAACTCACTGCTGTGGCTTTGGTGTACTCGCAACCCACATTCGAGATCTTTGAAGG

CCAGACTTCCGACGTGAAGGAAGGCAAGTATTCGATGCGCAACTTGGTGCCACGTTTCCTGCTGCGATCCTCGTATGTTG

CCTTCGCCACCTTCGTCTCCGCTGCTCTGCCCTTCTTTGGCGACATTAACGGCGTCTTGGGAGCATTCTGCTTCACCCCA

CTAGATTTCATCCTTCCCTTCATCTTCTACTCCTTCACCTTCGGCCCCTCGCGGCAGACGCCCAGGTTCTGGATCCACTG

GGGCATTGTCATCTTGTTCAGCGTGGTTGGGTTTCTAGGATGCATCTCCTCGGTTCACCAAGTTATCTTGGATGCCAAAT

ACTACAAGTGGTTTGCTGATTTGTAG

>PpAAP9B

ATGATGGCGCAGTGGCATGGAGATCATGACAAACTTGAATTAGGAAGAGTAAATGCTTGTATTGTCAATGCTGCACCGCC

CTATGATGACAGCAACAAATTTGATGATGATGGGAAAACGCGCCGCACAGGATCTTTCTGGACAGCCTCCGCGCATGTAA

TAACTGCTGTGATAGGGTCCGGGGTGCTATCCCTAGCGTGGAGCATGGCACAAATGGGGTGGGTGGCTGGGCCCTTGGTG

CTTCTACTTTTCTCATTCGTGACTTACTATACATCTTCGTTGTTGGCGGACTGCTATCGCCACCCTGATCCGGTTACTGG

CAAACGTAATTACACCTACATGGATGCTGTGAAAGCTAACTTAGGACCTCGACAAGTACTGCTTTGTGGTGTGGTGCAAT

ATGCTAACCTCTTGGGAACTTCAATTGGCTACACCATTACTGCAGCATCAAGCATGGTGGCTATTACAAGATCGGACTGC

TTCCATCACAAGGGTACCAAAGGACCATGTCAAGCCTCGAACATTCCATACATGAGTATGTTTGGTTTCGTACAGATTAT

TCTGTCCCAGATACCAGAGTTTGGAGAGCTGTGGTTCCTGTCTGTACTAGCTGCCGTAATGTCCTTTCTATATTCCACAA

TCGGCTTGGGACTCGGTATCGCCAAAGCTGTGGACCACCAGCATGGATATGGGTCAATAACCGGGATTTCAGTTGGCGAT

CCATCGGTAGGATATGTCTCCATGAGCAATAAGATTTGGGGGATATGTAGCGCACTCGGCAACATAGCATTTGCTTACTC

GTTCTCGATGATTCTAATCGAGATTCAGGACACCTTGAAATCTTCGCCCCCTGAAAACAAAACAATGAAGCGGGCTAGTC

TGTTTGGTATCATTACCACCACCATATTCTACATGTCGGTGGGATGTGCTGGGTACGCAGCTTTTGGAGATAATGCCCCA

GGCAACTTGCTGACCGGATTTGGATTCTACAACCCCTACTGGTTGGTAGACTTCGGCAACGCTTGCGTTGTTGTTCATCT

GGTTGGAGCCTACCAGGTGTATACACAACCACTGTTTGCATTCTTCGAGAATACGCTTTCAAGCAGATGGCCTAAGAGTC

AATTCATCCATAAAGAGTATTATTTGAAAGTTCCATGGGGTGAACCCTTGCACTTCAACCTCTTTCGATTGGTATGGCGT

TCCATGTATGTGGTTGTAACCACGGTGCTGTCAATGGTTCTACCTTTCTTCAACGATGTTATGGGTCTGATTGGGGCATT

TGCATTCTGGCCTCTAACTGTCTACTTCCCAGTTCAGATGTTCATCGTGCAACGACAGGTCCAAAGGTGGAGCCCAAAAT

GGTGCTGGTTACACCTTCTCTCGGTTTCCTGTTTTGCTGTTTCCTTAGCAGCAGCATTGGGATCAAGCGAATGTATGATC

AGTGATCTGAAAAAATACAAACCCTTTCAAGGTTAG

>Mapoly0107s0041

ATGCGGTGGGAGCAGCTGCGAAGTGAGAGAGGAGAGGGTCAGGAGACGGAGGAGATGGATTCGAGCTCGGGGGGCGAGGG

GAGCTACGGAGGGAGGGAGGGGCCGAGAGTGGTGACAGTGCGCGAGGTGAGCTTTGCGCCCAATGTGCGGTCGGAGGTGT

CATCACGGTCGGACGAGCCGGAGCTGATCTCGATCCCGGTGACACCGCAGGGGTCGACGCCGCCGAGCCAGGCGTCGCCA

CGCATCTTCTCGCCAGGAATTTTGACACCGAGCGGCGCTCAGACGCCCAGGGGGTTAGCGCTGGGCGCAATGGGCGCAAT

GGGCGCGAGCATTCCGGGAAGCAGCAAGGCGCCGTCGAGCTTGCCGACCCCGAGCCTGCCGACGCCGAAAACGCCGTGGA

CGCCGGGGCTGCGGTCGCCTCGGTTCCTGGGCACGCCGCTGGCCACGCCCATGCGCCGCGCTTTTGTGAACATGAAACAA

TATTTGGAGGACATTGGCCACTTCACGACGCTAAACCCTCGCGACGCCTGGCTACCGGTGACAGAGTCCCGAAATGGCAA

CGTCTTTTACGCTGCCTTCCACACCCTCAACGCATCAATTGGCTTCCAGTCCCTCTTCCTGCCCGTGGCCTTTATGTACC

TCGGCTGGACTTGGGGCAGTCTTGCTCTGACAGTAGCATTCGTGTGGCAGATGTACACCACGCATCTCCTCGTCATGATG

CACGAGTCGGTGCCCGGCAAGCGCCTCAATCGCTTCATTGAGATCGCGCAAGAAGCTTTCGGGCCGAAGCGCGGATTGCT

GATCGTCATACCGCCGCTGATCCAGCTGTCGGCGGGCTACTGCGTGGCTCTGACTATTCTGGGAGGGAGTGCTCTGCAGC

TGTTCTACTCCACGGTGTGCACCGAGTGCTTTTACACCAAAGTGCTCACGGTCATGGAGTGGTACCTCGTGTTCACGTGC

CTGTGTTGGGTCGTGGCCCTGCTGCCCAACCTCAATTCCATCTCGTATGTGTCGCTGGTCGGATCCATCATGGCCGTCGC

CTACTGCACTCTGCTCTGGGCCATCTCGGTCGATGCAGGCCGCCCCCTGCCCATCTCCTACGACGCCGTTCGACGTAAGT

CGGACCTGGCCACCGCTTTCACCATCTTCGTTGCGCTCGGCAACGTCGTCTTCGCCTTCAGAGGCCACAATCTCATTCCA

GAAATTCAGGCGACCATGCCTTCCAGCTTGAAGAACCCTGCTCACGTGCCCATGTGGAGAGGAGTGAGGATTGCCTTCGT

GACGGTGGCCATCTGCCTCTTCCCCATTGCAATCGCCGGCTACTGGGCTTACGGAGACAAGATGCTGCCGTCGGGGATCC

TATTCTCGCTGTACCACTACCACGTGATGGGGGCGTCGAGGGTGCTGATCGGGATGACATTCCTGATGGTGGCGCTGCAC

GCGGTGACCACGTACCAGATCTACGCCATGCAGCAGTTCGACTTCTTCGAGATGCTGCAGACGATGAGGTCGAACCGGCC

CGTGCCCTGGTGGCTGCGCCTGCTCTTCCGCTCGGGCTTCGTCTTCTTCAACTTCTTCGCGGCCGTGGCGCTGCCCTTCA

TCAGCAGCTTGGCCGGCTTCCTGGGCGGCATCAGCTCCATCCCCATCAGCTTCGCCTTCCCGTGCTTCATGTGGCTCTGG

ATCAAGAAGCCCAGCGTCCGAAGCTTCGACTGGTACCTCAACTGGACGCTCGGCATCTTCGGCATCTGCCTGTCTCTGTG

CGTGAGCGTCAGCGGCATCTGGAGCATCGTCGACACCGGATTGAGGTTGAACTTCTTCCGCCCCGGAGAAACATAA

>Mapoly0130s0012

ATGGACGTGGTTGAGATCTCGGAGGCAGCGGACGATATCAAGGAATTGGACGATGATCTGAAACCTGCGAGGACAGACGA

TATCAAGGAATTGGACGATGATCTGAAACCTGCGAGGACAGGAAACGTGTTCAACGCCACGGCCCATGTAGTAACGGCAG

TGATAGGGTCTGGAGTTTTGGGTTTGCCTTGGAGTATTGCGCAGTTGGGATGGGTGGCAGGTGTTATCATGTTGGTGTTC

TTTGCATTGGCGACATTGTATACTTCGGCGCTCTTGGCAGATTGTTATAGAGATCCCGTGACGGGAAAGAGAAGTTATAC

GTACATGGATGCGGTGAATTGTCATCTGGGAAAAAAACAGGTTTACTTGTGTGCTATAGCCCAGTACCTGAATCTTCTAT

GTACGTCTATAGGCTATACTATCACTACTGCAACGAGTATGGTTGCAGTCAAGAGAGCATTATGCTTCCATACAAATGGT

CACGACGCTCATTGCCACGTTTCCAACATTCCATGGATGATAGTGTTTGGTGGGATTCAGATATTTCTGTCACAGTTGCC

AGATTTCAGTCATCTGAAGTTTGTATCAATTGTGGCTGCTATTATGTCTGCGATATACTCCTTTATTGGAGTGGGGCTCA

GCATTGCCAGAATGGCAATTAGAAATGTTTCGGGTCCGGTATGGGGTACTCAACTTTCTCCTGCTGCGAAGACGTGGTCC

GTGTTTCAGGCTCTAGGCAATATAGCGTTTGCGTATTCCTTCAGTATGGTGCTGATCGAAATCCAAGACACTCTCAGAGG

TACACCTAAATATGCACCGGAGACTGTGACGATGAAAAAAGGGACGAAATGGGGGATAGGCATCACAACGGCGTTTTACA

CTTCAGTTGGTTGCCTCGGCTTTGCAGCATTTGGACAGCATGCTCCTGGTAATATTTTAACAGGGTTTGGTTTCTACAAC

CCATACTGGATCTTGGCCATAGGAAATATTTGTGTGGTAATACATCTCATTGGTGCATATCAGGTTTATGTACAGCCAAT

TTTTGCTGCTAGCGAAAGAGCTCTCCGCATCAACTGGGCTGGAATGAAGCTAATTTGGTTCCGCTTGATATTTAGAACCT

TATTTGTTATAGCCGTCACTTTTGTTGCTAGCGCCTTGCCATTCTTCAACGATATCAATGGTTTTATCGGAGCAATTTCG

TTTTTTCCTCTCACTGTGTATTTCCCCACTGCAATGTATATAAAATCAAAAGGCAAGTCACTAACCTGTACTAGAGAGGT

AATCATTATATGGGTTTTGAGAATCATATCTGGGTTGATAACACTAGTTGCTATGATAGGTTCTGTAGAGGGCATCCAAA

GTTCTGTGAAGGGTACAAAACTTTTTCATACCAAGAATTAG

>Mapoly0134s0048

ATGTCAGAGGAGACCATGCCTGCCTCCAAGAATCGGGCAGCCATGGATATAACTCATCTCGGTCTGCCGAATCGTCACGT

CGACGTCGATGGGCTTCTTACAGCAAGAAACTCCGTGGACTTCACGACAGTGTTCGTTGACCCCAAATCACAAAGGGCCG

GCGGGCTTGATGCCCATGATCGTACTCCCAGAACTGGAACAATGTGGACAGCGACAGCTCATGTCATCACAGCCGTCATC

GGCTCGGGGGTTTTGTCATTGGCATGGGCAATTTCTCAAATGGGGTGGATTGCAGGTCCCTTGGTGCTGCTAGCATTTGG

TGGAATCACCTACTATACATCAGTCATGCTTGCCGATAGCTATCGCTTCCCTCATCCTGAGACAGGAAAGAGGAACCCAA

CGTACATGGCTGCAGTCAAGGCAAGCTTAGGATCTAGAGAGGTCAAAGCTTGTGGAATTCTGCAGTACATGTCGCTGTTT

GGAACTTGTGTCGGGTACACTATAACGACATCACACAGCCTCGAAGCAATTTTTCGGGCAGGATGCTATCACGCCGAAGG

ACATGATGCAGAATGCAAAACATCACTCAGCTATTACATGTTGGGTTTTGGTGCCACAGAACTAATTTTCTCTCAAATAC

CCAACTTTGATAAGATGTCCTGGTTATCGATAGTTGCAGCAGTGATGTCCATAAGCTACTCCGGAATCGGACTCGGACTC

GGGATTGCCAAAGCTGCTGAGCATGGGCCTCGTGGGACACTCGGTGGATTGGACATAGGCCGAGGACCAGGGGAAGTGGC

ATTGATCGAAAAGCTTCTGTCTGTCGCAAATGCTTTGGGAAACATGGCCTTTGCCTACTCTTTCTCTACAATCTTAATAA

CGATACAAGACACGCTAAAAAGTTCTCCTCCTGAGAACAAGATCATGAAGAAAGCAACTCTGATCGGTATCACAACGACA

ACATTGTTTTACATTTCTGTTGGATGCGCTGGATATGCTGCTTTCGGGAACGAGGCGCCTGGAGACCTGCTCACGGATTT

CGGATTCTACGAACCGTGGTGGCTAGTCGACTTTGCAAATGCTTGTCTTGTCGTGCATCTTATCGGGGCGTACCAAGTTT

TTGCGCAGCCAGTCTTCGCATTCGTCGAGGGCTGGATCACCAAGAGACGACCGGATAGTCATTTTATTCATAAGAATTTT

GACCTCGTTCTGCCATGGGGATCTACGTACCATTTGAATCTGTTTCGACTCACGTGGAGGTCGGGCTATGTGGCCGTGAC

CACGCTGTTGGCCATATTGTTGCCGTTTTTCAACGCAATTATGGGACTTCTGGGCGCGATAGGATTTTGGCCTCTCACTA

TCTACTTTCCGATTCAGATGTACAAGAAGCAAGCTGCCATCAATTTCGGTTCCAAGAAATGGTACATGCTTAATGCAGTA

AGTGCCATCTGCTTACTGGTGACTTTGGCTGCCGCGGTAGCTTCGATTCAAAGCATAATCCAGAAGAGCAAGGAATATAA

ACCCTTCTCCTCCTGA

>Mapoly0170s0014

ATGGATTTGACTGAGATCTCGGAGGGCGCGGACGATAGTCGCAAGTTCGATGATGATCTGAAACCTGCAAGGACGGGAAC

CTGGTTGAACGCCACTGCACATGTTGTGACAGCAGTCATAGGGTCTGGAGTTTTGGGTTTGCCATGGAGTATAGCGCAGT

TAGGATGGGTGGTAGGTTCTATCATGCTTATTTTCTTTGCATGGGCCACATTCTATACTTCGGCGCTGTTGGCAGATTGT

TACAGGCATCATGTGACGGGAAGGAGAAACTACACATACATGGATGCGGTGAATTCTCATCTGGGAAAAAAACAGGTTTA

TTTGTGCGCTATAGCTCAGTACCTTAATCTTCTATGCACGTCTATAGGCTATACCGTCACTAGTGCAACCAGTATGGTTG

CAGTAAAGAGAGCAATATGTTTCCACAGGAATGGTCACGAGGCTCATTGTCACGTTTCCAACATTCCGTGGATGATATTG

TTCGGTGGGATTCAGATATTCCTGTCACAGTTGCCAGATTTTAGTCATCTGAAGTTTGTATCGATTGTAGCTGCTGTCAT

GTCTGCGATATACTCGTTCATTGGACTGGGGCTGAGCGTTGCCAGAGTGTCAATTAACAAACATGCCAAGGGTCCGGTGT

GGGGAACTCAACTTTCTGCTGCCCAGAAGACTTGGTCCGTGTTTCAGGCTCTAGGCAACATAGCGTTCGCGTATTCCTTC

TGCATGGTGCTGATTGAAATCCAAGACACTGTCAGAGGGCCACCTAAATATGCAATGGAGAATGTGACGATGAAAAAGGC

GACGATATGGGGGATAAGTATCACGACGGCGTTTTACACAACAGTAGGTTGTTTGGGTTTTGCGGCATTTGGAATCCATG

CTCCGGGTAATATTCTAACCGGATTTGGATTCTACAACCCGTATTGGATCTTGGCTATAGGAAATATATGTGTGGTTATA

CATCTTGTTGGTGCATATCAGGTTTATGTACAGCCAATTTTCGCTGCTCTGGAACGAGTTGTACACAACAATTGGCCTAG

AAAGAATCTAATTGGGTTCCGCTTGATAACTAGAACTTTGTTTGTGATAGCAGTCACTCTCGTAGCTAGCGCCTTGCCCT

TCTTCAACGATATCAATGGTTTTATTGGAGCGATATCGTTTTTCCCTCTCACTGTTTATTTCCCCACTGCAATGTATATA

AAATCAAAAGGCAAGGCACTTACCTCTACCAAAGAGTTAACCATGATATGGGCTTTGAGAATCATATCCGGCCTCATAAC

ACTAGTTGCCATTATAGGGTCTGTAGAGGGGATCCGTAGTTCTGTACAGGGTACAAAGCTTTTTCACACCAAGAATTAG

>Mapoly0032s0115

ATGGTAGCGGATGAGACAGTTCCCAGCGCACTGGAATTGGGAAACGGAACCTTGAAGCAAAGGCATCATCAAGCAGATCC

GGACGCTGCCAATAAGGATGCAGGGGCCTTGTTCGTCTTGGAATCCAAAGGGACGTGGAAGCACGCAGGATTTCATCTGT

CGTCGTCGATTGCCGCGCCGCCGCTGCTGACGCTGCCATTCGCCTTTGTGGCCTTGGGCTGGGAGTATGGAGTGCTGATG

CTCGTGCTGGGCGCCGCTGTCACATTCTACGCCTACAACTTACTGTCCAAGGTGTTGGAGCATCTGGCCGAGCAAGGTCG

CCGTCACCTTCGATTCCGTGACTTGGCCACCGACGTTCTCGGACCTACTCTCTCGAAATGGATCGTTTATCCGACGCAGC

TCGTAGTCTGTATAGGGGTGGTAATTGCAAGTCCCCTTCTGGGAGGAATCAGTATCAAAATTATATACAAACTGTATGCT

CCTGATGGAGATCTCAAGCTCTATCATTTCATCATCATATTTGGAATCGGATTGATATTCGTCGGCCAGATGCCATCATT

CCATTCCTTACGTTATATCAACTTCGTGTCTCTCGTTATGACTCTCTGCTACAGCTTCACAGTTTCCGGAGCTTGCATTT

ATCTAGGTAAATCGGACAGAGCTCCTGCTAAGGATTACAATCTACCGGGAAGTGAAACTCAGAAAGTTTTTGGAGCGTTC

AACTCCTTGGCCATCATTGCAACGTCGTATGGAAATGGAATCATTCCGGAAATACAGGCAACTCTGGAGCCGCCTGTGAC

AGGCAAAATGTTCAAGGGTTTGACAGTGGCTTATGCCGTGGTCATCTCCACCTTCTTCACCGTCGCCATTTCCGGGTACT

GGGCTTTCGGAAATTCTGCAGCAGGCAGCATCTTCACCAATTTCCTTGAAGCCGATGGAACGCCACTTGTTCCCAAATGG

CTCATTGTCATGCCCAACGTCCTATGCATCATCCAGCTTCTGTCTGTCTCCATTATATACTGCCAACCGACATTCGATTT

GTTGGAGGGCAAGAGTGCAGACGTAGAGAAAGGACGATTTTCTGCTCGGAATTGGATCCCTCGTCTGATTCTGCGCACGA

CATTTCTGTGCGCGTGTACTCTGATCGGCGCCATGTTTCCCTTCTTCGGCGATGTGAACGCCATAATCGGGGCCTTCGGG

TTCACTCCGCTGGACTTCGTGTTCCCAATGCTGTTCTACATCCTGGTCTTCAAGCCGCCCAAGCGCTCCATCAAATTCTG

GGGCAACCTCATCCTCCTCGTTGCTTACACTGGGGTCGGTGTGGTGGGCGCCATCGCTGCCATCCGCCAGCTCGTTCTGG

ACACCAGCTATTATAAGCTTTTTGCCGATATTTAG

>Mapoly0032s0116

ATGGTGGCGCTCGAGACGGAGGAATCGGGCAGCGGATCTCTGAAGCATGGGGCGCTGGAGTCGCGGGAGCAGTGGGAGCC

GAAGAGAGACGCCGGTGCGCTGGTCGTCCTCGAATCCAAAGGGAGCTGGCAACACGTCGGGTTCCATTTGACGGCTTCAA

TCGCTGGACCTCCGATTCTCACGTTCCCATTCGCATTTGCGGCATTGGGCTGGGCATGGGGAATGATCGCGCTCGTGTCC

GCCGGCGTCGTCACCTTCTACTCCTACAACTTGCTTTCGCTGATCTTGGAGCACCGCGCTGCCCAAGGTCGTCGCCACGT

CCGATTCCGCGACTTGGCCACAGACATTTTGGGGCCGAAACTCTCATATGGGCTTGTTTATCCGATGCAGTTTGTTGTGT

GTCTAGGCGCAGTGATTTCCATTGTCTTGGTTGGAGGAATCAGTGCCAAAGTTGTGTACAAAGTATACTACCCGGATGGA

GATCTGCAGCTGTATCAATTCGTCGTCTTCTTCGGGGCGGCCACGATGATCATGGGCCAACTCCCGTCGTTCCACTCGCT

GCGCTACGTCAACCTTCTGTCGCTGCTGATGTGTCTGACCTACAGCTTCACCATAACCGGGGCCTGCATCCAGTTAGGAT

ACTCCGACCGAGCTCCCCCGAGAGACTACAGTTTACCGGGGAGTGAGAAACAGCAAGCATTCGCGGCGTTCAACGCCTTG

GCGCTGATCGCTACGGCGTACGGGAATTCGATCATTCCCGAAACTCAGGCAACTCTGGCGCCTCCGGTCGAGGGGAAGAT

GTTCAAAGGACTGGCGATGGCGTACTCGGTCATAGTGGTGACGTTTCTGCCTGTGGCCATCGCCGGATACTGGACGTTCG

GAAACCTCTCGGCCCCCAGCGTGTTCACCAACTTCCAAACAGCGGACGGAACACTGCTCGTTTCGAAATGGCTGATCGTT

ATGCCCAGCTCCATGTGCGTCATCCAGCTTATATCTGCCGCAATTATATATAGTCAACCGACGTTTGATCTTTTCGAGCG

GAAGATCGCGGATGTGGACAAGGATCGATTCACAGCTCGGAACGGGATTCCTCGGGTGATAGTGCGCGTTGTTTTTCTGG

CTCTGGTGACTCTGATAGCTGCGATGTTCCCATTCTTTGGTGACGTGAACGCGATCGTGGGCTCGTTCGGATTCACTCCT

CTAGACTTCGTGTTCCCCATGGTCTTTTACATCCTGGTGTTCAAGCCGCCCAAACGTTCCATCAAATTCTGGGGCAATCT

GATCATCATCATTGTTTACAGCCTCGTGGGCCTGGCCGGGGCTGTAGCTGCTGTACGCCAGCTGATTCTGGACACGAGCT

ACTACAAACTGTTTGCGGATGTTTGA

>Mapoly0004s0095

ATGGAAGACTCAGAGTCTTCGAAGACAACACAGGAACTGCGAATGAGTAGGATTGTTGATCGAATCGATCAGAGTGTTGA

CAGGAATGTAGAAGGAACAAATCTCGTACCAATGTCAAGCGCTGAACTGGACGATGATGGGCGTCCTAAACGCACAGGAA

CGTTTTGGACGGCATCTGCCCATGCAATAACCGCATGTATAGGATCTGGAGTATTATCTTTGGCATGGGGAGTCGCTCAG

CTTGGCTGGATAGCTGGGCCCTTGGTGAATGTTTCCTTTGCCGTCATTACATACTTCACTTCGGTTCTGCTCGTTGATTC

CTACCGCTACATGAATCCTGTCTCTGGGAGGCGGCTGTACACATATATGGATACAGTGAAGAGTTATCTGGGGCGGAAGG

ACACGTTTGTTTGCGGACTTGTGCAGTATACCAATCTTTTCGGTATTTGCATTGGTTACACCGTTACCGCCTCAGCCAGT

ATGGTGGCAATCAAACGGGCCGGCTGTTTTCATGAAAGAGGACGTCATGCAGAATGTCACGTATCAAACAATTTGTACAT

GGGTATTTTTGGCGCAGCTCAAATCTTCTTCTCGCAAATTCCAGATTTTGCTCAGACTTGGTGGCTCTCAGTACTTGCCG

CAATCATGTCATTCTCCTACTCAGGCATCGGTCTTGTATTGAGTCTCATTAAATTCTTCGATGAAGGACAAGTTAGGGGG

ACACTTTATGGTTGGCCTATTGGTAATGGACAGGATGAATACAGTAGAGCTCAGAAAGTATGGAAGACATTGTCGGCAGT

AGGCGATATTGCCTTTGCATACAGTTTCATTATAGTACTTCTTAACATACAGGACACGCTGAAAAGCCCTCCGCCAGAGA

ACAAGACCATGAAAAAGGTTGCGCTGGCATCAAACCTTACTTGCAGTATTTTCTACTTGTCCGTTGGTTGTGTGGGCTAC

GCTGCCTTCGGTAACCATGCTCCGGGGAACCTCCTCACAGGCTTCGGGTTTTACGAGCCATATTGGCTCATCGACTTCGC

CAATGCATGCATCGTCGTTCATCTTGTGGGAGCTTACCAGATTTTTGCTCAACCGCTTTTCTCTTTCTTAGAAGGTTTGG

TATTGAAAAGATGGCCAAAGAAGACCTTTCTCAACAATACTCGAAGTGTGTCCATACGTGTTGTTGGTAGAATCCGACTG

AACATTTTCCACCTGGTCTGGAGAACCATCTTCGTTCTGGTCACGACGCTTCTCGCTCTATTATTGCCTTTTTTCAACAG

CATTATGGGCTTGCTAGGAGCTTTAGCCTTCTTCCCGCTAAGTGTCTATTTCCCCATCCGGATCTATATGAAACGAACCA

ACTTGCGACATTACAAATGTGAGTGGTGGTTTCTATGTACATTAATGGTTATCTGCCTCCTAGTTTCTATCGGTGCTGCA

ATTGGATCTGTTGCAGAGATCCTTCAAGATACAAGACACTACAAACCTTTCAACTGA

>Mapoly0040s0055

ATGGAAGAGGAGCATCGGCGACGTCCGCAATCACCCGCCACGGTGCACGAAGTCATGGAGTCTGACGGGGACTCGGGGAC

CGTCACGCCGACGGGACACTCCAGAGAGCTCGCCGGTTACGTGCCGAAATCGTCACCGCATTCTGTAGCCGGTAGTACGC

CGGCGGCGAGCCCCCGACACCGACTGGACGAGCTCGATACCTTACCTGCTCCGCCCTTCAGGCCCATGGCGAGCGAGTTG

CAGCACTCGCCGTCCCTCAGCGACGGCATCTTTCCCGTCGCCGGGCACACGCCGGAGCTCGACCGGCAGCGAAGCACGGC

GAGTCTGATTCCCAGGAGCGGGCCGTCGCAGCTGCGGCCTCCTCCGACTCCTCCCAAATCTCCCGCGACGCCCCTATCTC

CTGCGACGCCCAAGTCCCCCGTCAAGGCCTTCGCGTCGTGGGCCACGTCACCGCTGCGCAGCCCGCTGCCGTCCCCGCTG

GCCACCACGCTGCGCAACATGAAGGAGTATCTGCAGGAGCTCGGGCACCTGACGACCATCGACCCCCGCGACACGTGGCT

CCCCATCACCGAGTCCAGGCACGGCAATGCCTACTACGCCGCATTCCACAACGTAAACGCTTCGATCGGATTTCAAGCTC

TGCTCTTGCCCCTCGCCTTCACCTTCCTCGGCTGGACCAGTGGGATTGTGTGTCTCATCATTGCTTTCTGCTGGATGATG

TACACCAAGTGGTTACTGGTGTCTATGCACGAGTGTGTTCCGGGGAAGAGAGTGAATCGGTACCTTGATCTCGTCGAAAT

AGCATTCGGAAACGGCATCGGTAAACAGATCATTGTAATACAACTCCTGATCCTACTTGGAGGAACATGCATCGGACTCA

TTACCATCGGAGGCAGCTGTTTGCAGCTCTTCTTCCGAACAGTGTGCCCGACCTGTCAGAGCCCGCTCACTCCCATCGAG

TGGTACCTCGTGTTCACCATCCTCTGCGCCCTGTTATCCTACTTCCCCAACCTCAACTCTGTTGCAGGAGTGTCCCTCAT

AGGAGCAACCATGGCCGTTGCCTACACCACTCTGTTGTGGACTCTGAGTGTCAGCGTCCCCAGAATGCCCAATGTGGGCT

ACGAGATTGTCTCCGGAGGATCCGCCCTGGTCACAACTTTTGGCATACTCAATGCCCTCGGCATGATCGTCTTCGCCTTC

AGAGGACACAATCTCGTTCTCGAGATTCAGGCTACCATGCCATCCACCTTGAAGCACCCGGCACGCGTTCCCATGTGGAG

AGGTGCGACGGCAGCGTTTGCTCTCACAGCTCTGTGCTACTTCCCCCTGGCTATAGGAGGATACTGGGCTTACGGAGACA

AGATGCTGCCGGGGGGAATTCTGTACTCGCTGAGTTTCTTTCATGGACAGGACATATCGCGCACCCTGCAGGGGACGACG

TTCATGTTCATCGTGGTGAACTCGCTGGGCAGTTTTCAGGTGTACGCGATGTCGATCTTCGACATGATCGAGCAGGTGTA

CAGCAAGTGGTTCGACCGGAAGTGCAACGCGCTCTTGCGCCTGATCTACCGCACGGTGATCGTGTTCCTGTGCTTTCTGG

GGGCGGTGGCCATCCCGTTCCTGTCGACGATGGCTGGGCTCATCGGCGGCCTCACGAATCTGCCCGTCACCTTCTTCCTC

CCGTCGCTCATGTACCTGAAGGTCGTGGCGCCGCGCCCCCGCAGCTTCTCCTGGTACCTCAACATCGGCCTCGGCACTTT

CGGCTGCGCCCTCTCGATCGCCGTCTGCGCCGGCGGCGTCTACAGCATCATCGAGACCGGCATCAAGCTCAATTTCTTCA

AGCCCGCCTGA

>Mapoly0047s0067

ATGTCGAAAATGGGCTCCGAGGAAGCTAAATACAGCTCGGTAACCGGATCTGAAGAAAAGCTGGACCAGCAGGAGCAGGC

AAAATCTGAGATGCAGCAGAAGAGGTCTCCCTTCGATGGAGTGGAGTCCGAAACTGGCAAACTCTTGGATGACGACGGGA

GGCCCATGCGCACAGGAAACACTTGGACCGCTACAGCTCACATCATCACTGCTGTTATCGGGTCCGGAGTGCTATCGCTG

TCGTGGAGCTTCGCGCAGATGGGATGGATTGCAGGGCCCCTGGTGCTCTTCAGCTTTGCGCTCTGTACGTGGTACACGTC

CAGGCTCTTGGCCGACTGTTACCGGTATCCCCACCCGGTCACCGGCAGACGGAACTACATCTACATGGATGCGGTTAAAG

TTAACGTCTCAGCTCGGTCCCATTTGATCTGCGGTATCATGCAGTACAGCAACCTCGTCGGTACCTCCATCGGCTACACC

ATCGCCACGGCCACCAGTGCAGTAGCAGTGCAAAAGTCAAATTGCTATCACTCGCATGGCCGCGACTCACCGTGCTTGGC

CTCCACCACATCTTACATTGCAGTGTTCGGTGTCATCCAGATTTTCCTGTCACAAATCCCAAATTTTGGGGACCTATGGT

GGCTTTCGTATGTTGCCGCAATCATGTCCTTCACATACGCCACCATTGGTCTTGGACTCGGCATTTCTAAAGCTGCCGAG

GGTGGCCATTCATACGGCAGCGTAGGTGGAGTACGAATCGGCACAGATGTGACTGAAGCTCAAAAAGTGTGGAACATCTT

TGCGGCTTTAGGAAATATGGCATTTGCTTACTCCTTCTCCATGATTCTAATTGAAATTGAGGACACTATCCGAAGTCCTC

CGGCTGAAAACAAGCAGATGAAAAAAGCCACCTTGTGGGGCACCTCTGTCACAACAGCTTTCTACATGTCCGTGGCCATT

GCTGGTTACTTGGCATTTGGTGATGCTGCACCCGGCAATCTATTGACTGGATTCTTTAGCCCATATTGGCTGGTGGATTT

TGCAAACGTATGCATCGTCATCCATCTGATTGGAGCATATCAGGTGTACACTCAACCTGTGTACCAATTTGCGGAGAGGT

GGGTAGCGAGAAGGTGGAGCAAAAGAAGCTTTCACAGCAGAGGTTACACCGTCAGGCTTCCTGGAGGGTCCAACTTCAGA

CTAAACTGGTTTCGTTTGACCTGGAGAACAATATACGTGATCATCACCACAATCATTTCCATGTTATTACCCTTCTTCAA

TGCCGTTCTAGGTATCATTGGAGCCCTTGCTTTCTGGCCACTGACAGTTTACTATCCTGTCGTCATGTACATGAACCAGC

ACAAGATCCCCCGCTGGAGCCCGACCTGGATAGCTCTTCATAGTCTCAGTTTTGTGACCTTCCTCGTCTCGCTGGCTGGT

CTGATCGGATCTGTGGCAGGCATTGTTAACGATTTGTCGCAGGTTAAACCGTTCGAGAAAATTTAA

>Mapoly0052s0005

ATGGCGCCTCGTGAGGATTCCGCCGCGGAGACCGAGCTGGCCAGGCCGCCGGGGAGCCACGCTCCGAACAAAGATGAAAA

TGATGTCGACGGACCGCGACACCCCGGCACGATCAAGAACGTGGATGACTGGCTTCCAGTGACAGGATCTCGAAACGCAA

AGTGGTGGTACTCCACCTTCCACAATGTCACGGCTATGGTGGGCGCGGGCGTGCTCAGTCTTCCCTCCGCCATGGCATAT

TTCGGATGGGGTTTTGGAACGGTGGTTCTCGTAGGATCGTGGTGCATCACGCTGTACACGCTATGGCAGATGGTGGAAAT

GCACGAGATGGTTCCGGGCAAGAGGTTCGATCGATACCACGAGTTGGGGCAGCAGGCATTCGGGCCCAGGCTCGGGCTTT

GGATCGTCGTTCCTCAGCAGCTCATCGTCGAAGTCGGCGTCGACATCGTCTACATGGTGACGGGCGGTGACTCGCTGCAG

AAGTTCCATAACCTGATCAAGTGCCCGAGTTCGGCATCCAACCCCGATGAGCATTGCGGCCATATCGGCAAATCTGCGTG

GATCATCATCTTCGCCTCGCAGCACTTTCTGCTCGCCCAGCTTCCCAATTTCAACTCAATTGCTGGTGTGTCACTGGCGG

CTGCGGTCATGTCCTTGAGCTATTCGACGATCGCATGGGCTGCACCACTCGCGCAAGGCAGGGCAGCTGTTCACGACTAT

GGACTGCCCGACAAAACCACAGCAACGCTGGTTTTCGGAGTGTTCAACGCGTTGGGCAACGTCGCGTTCGCATACGCAGG

CCACAATGTGGTGCTGGAGATCCAGGCCACCATCCCCTCCACGCCGGAGAGGCCTTCGAAGATCGCCATGTGGCGAGGAG

TGGTTCTGGCCTACATCGTGGTGGCCGCCTGCTACTTCCCAGTTGCCATCGTCGGCTACTGGGCGTTCGGCAACACTGTC

AAGGACAACGTCCTCCTGCAGCTCGGCAAACCGAGGTGGCTGATTGCCATGGCCAACTTCATGGTGGTCATTCACGTCAC

TGGCAGTTACCAGCTGTACGCGATGCCCGTGTTCGACATGATTGAAACGGTTTTGGTCAAGAAAATGCACATCCCTCCTT

CGGCACCTCTGCGAGTTGTAGTTCGCTGTCTTTATGTTGCGTTCACGGCCTTCGTGGCGTGCACTCTGCCTTTCTTTGGA

GCTCTTCTCGGATTTCTGGGAGGCTTCGCTTTTGCTCCTACAACATACTTCTTACCGTGCATCATGTGGCTGGTCATCTA

CAAACCGAAACGCTTCAGCCTTTCGTGGTGTGCTAATTGGGTTTGCATTGTTATAGGTGTACTACTCATGCTTGTCTCGT

CGATCGGAGGACTTAGGAACATCATCGTTAGCGCCTCAACTTACAAGTTCTACCAGTAA

>75458

TGGCTATCGATCAACGATTCCTGGACCTCCAAATGGTGGTATGCGGCGGTCCACAACATCACTGCCGTGATTGGAGCTGG

AGTTCTTTCGCTCCACGCAGCTATGGTCGACTTAAGCTGGGCTCCAGGGATTTTCGTGCTGTGCGTTATCGGCGTCATAA

GTCTCAGCACAATGTGGCAAATGATCGAGCTCCACGAGCTCGATGGGAAACGCATGGACAGATACCATGAGCTGGGCCAG

AGGGCTTTCGGAAAGAAGCTCGGGCTCTGGATTGTGGTACCCATGCAAATGCTCGTCGAGATCGGTGTCGACACCGTCTA

CCTCCTCACCGCTGGAAAATCCATCCGGAAGATCCACAGCCTTCTCTATGGTTGCCCAATTCAAGACTCTTCATGTAACT

GGGAGCTGCGATATTGCATCATGGCGTTTGCATCAGTCCAGCTTCTCCTCTCGCAGCTTCCACATTTCACCTCCATCACC

TGGGTTTCGATTATTGCCGCTTTCATGTCTCTCGGCTACTCCACAATTGCTTGGGTCGCCACCTTAATGCGTGAACGAAG

CCCCACAGTCAGCTATGAATTTCCAAAAGCAACTTCTACTGCGGATGTTATTTTCAGGGTGTTTAGCTCGCTGGGCCAGA

TCTCGTTTGCATTTGCGGGACACAATATCGTTCTTGAGATCCAGGCCACTATTCCTTCCACCATTGAGAGACCCTCCAAG

ATCTCGGCCTGGAATGGAGCTCTCCTGGCCTACACGATGACCATCTTGTGTTATTTCCCAAATGCCCTGGTCGGGTATTA

CGTTTTTGGGAATCAAAAGAACCATGACATGCACGTCCTTGAGATTCTGGACAAGCCTGTTTGGCTCGTCGCACTAGGAA

ATGCAATGGTGGTGACGCATATGTGTGGTGGCTTTCAGATCTTCGCAATGCCGCTGTTTGACAATGTGGAAATGCTTCTC

ACAAACCTGTGGAAGGTTAATGGAGGGATCAATCTTAGATTACTTGTTCGATCAATTTACGTTGCATTTACATGCTTTCT

TGCTGTGACATTTCCTTTCTTTGACGATTTGCTCGCTTTTGTTGGGGGAATAGCCTTCGTCCCAACAACATTCCTGCTAC

CTTGTATCATCTGGCAAATTCTTCGCAAGCCCAGAACATTGGGATTGCCATGGCTAGCGAACATGGCTTGCATAGGAGTA

GGCTTCTTTCTTACAATCGCTTCCACAGCTGGTGGGCTTCGAAACATTCTCCTCAAGGCTTCGCATTACCAGTTCTACAA

ATAA

>SmAAP9C

ATGGAAAAGAACTGGTTAGCTTGTTCCATAGCTTCTAAAACTTACGATTTCTTTTTCCTGTTTCGTTGTCCCTCAGGAAC

TGTGTGGACTGCCTCGGCTCATGTGATCACAGCAGTGATCGGCTCTGGAGTTTTGTCACTGGCGTGGAGCATGGCTCAGC

TTGGCTGGGCAGTCGGACCCCCCGTCCTGCTCGCATTTGCATTTGTCACGTATTACACCTCCATCCTCCTCGCGGATTGC

TATCGCAGCCCCGATCCAGTCACAGGGAAGCGGAATCACACGTACCAAGATGCGGTGGCAGTCACTCTAGGGGGTGCAAA

GGTTTGGATTTGCGGCATTGTCCAGTACACCAACTTGGTTGGAACAGCAATCGGCTACACAATCACTGCGTCGATTAGCA

TGGTGGCGATAAGTCGATCAGATTGCTTCCATCGCCAAGGACACGATGGTCCGTGCTACGCCTCGGATTACCCGTACATG

GTGGTGTTTGGAGCGGTGCAGATTTTGCTGTCACAAATCCCGGATTTTGATCGAATATGGTGGCTGTCCATCGCCGCAGC

TATCATGTCCTTCGCCTACTCCTTCATTGGGCTTGGCCTCGGGATGGCGCGAACTTTCGAGCCGGGGCACTCATACGGAA

CAGCCACCGGAGTCCGCATTGGAATGGGAGGACTTTCGCAAACGCGAAAGATCTGGCAAGTCTTCCAGTCGCTGGGAAAT

GTCGCGTTTGCATACTCTTTTTCCATGATCCTCATCGAAATACAGGATACGTTAAAATCTCCACCACCTGAGAACAAGAC

CATGAAGAAGGCGACGCTGGTGGGAGTCGTCACCACCACCGCGTTTTACATGTCGGTGGGGTGCTTTGGCTACGCGGCCT

TTGGAAACAATGCTCCTGGAAACCTCCTCACGGGCTTTGGCTTCTACGAGCCCTTCTGGCTCATCGATTTCGCAAACGCT

TGCATCGTCATCCACCTTGTAGGAGCGTACCAAGTCTATTGCCAGCCAGTGTTCGCCTATGTCGAAGGCCATGCGCGATC

ACGGTGGCCAAAGAACAAGTTTGTCAGCCACTACTTTAGGATCCCGATCCCTCTCCTGGGCTGTTACAAATTCACGCTGC

TCACGCTTGTCTGGAGGAGCGCTTTTGTGGTAGTGACCACCATCGTGTCGATGCTGCTCCCCTTCTTCAACGACGTCCTG

GGCCTCCTTGGCGCGATCTCGTTTTGGCCGCTCACAGTCTACTTTCCAATCGAGATGTATATTAAGCAGAGAAGCATCGT

GAGGTGGTCGCCAAAGTGGATCGGTCTTAAGGCCCTTGATTTGGGCTGCTTGCTCGTGTCCGTGGCCGCGACGCTGGGAT

CCGTGGAAGGCATTGCGCTATCCCTCAAAGAATACGCCCCCTTCAAGTCATAG

>98385

ATGAGTAACGCTGCAAGCTACTCCGTCTTTTGGAATAGTCTACGGGGTATACTGCAACCGGAACACCATGCCAAGTGGTG

GCACTCAACCGTTCACATCGCGACGGCTATGGTAGGAGCCGGAGTTTTGAGCCTTCCACTAAATTTGTGTGTCCACAGGG

CTCCTGGAATGATGATGCAGGGCGTTTCGTGGATAATCACACTGGCTACCATGTACCAATTGATCGAGATGCACGAGGAT

GAGTACGACACTTACCGGGATCTCGGAAGAAAAGCATTCGGGGACAGGCTTGGGTTCATAGTTGGTTTGCAGCAGATCGT

AGTTCAGGTGACTGCCAACATTGCTTACTTGGTAACTGGCGGCCAGGCGCTCAAGAGGTTCGGGGATCTTGTTCTGAGTC

GCGAGATTCAGTACGGGAAGTTTGAGCTTGCTGTGGCATGGATATCCGCGTTTGCTGGAGTTCAGGCGGTACTGTCGTTG

TTTGCCAGTTTCTCGTCCACGACAATCGTCTCTCTGGTGGCCGCCATCATGTCTTTCAGCTACTCGACTATAATTTGGGC

AACAGCTATACGGCTTAAGAGTTCGCAAGTATCGTATCTCTACTGCAACTGGAGATACTACAGAGCCTCTAATGCACTCG

GAGAGATTGCGTTTGCCTATGGAGGCCAAAACATTGCTCTCAAGATCCAAGCAATGATGAGATCAACGAGGCACAAACCG

TCTAAACTACCAATGTGGAATGGAGTTTTGGTGGCTTATGTGATGGTTGCCGTTTGCTATTTCCCAGTCGCTGGAGTTGG

CTATTGGGCTCTTGGCAACTTGACTTGCTACGAAAACGTTCTGGATATTTTCCTCGATAAACCAAAGTGGCTGATAGGGA

CAGCCAACTTGATGCTTATGCTGCACCTCACAGGGAGTTACCAGGTTTTTGCTTTGCCGATTTATGATGGACTAACTTGC

TGGCTGGAGCAAAAGAAACTGCCAATCAATGCTTGGATTAGGCCTCTTTACGTAAGTAAGGGAGCGCTGCCAGGATTTAC

GTGTTTGGTTGCGGTGATCATACCTTCGTTTATCGGGCATCTGGGATTATTCGGTGGGCTCGCTCTTGGACCAACAACGT

ATCAGTTGCCGTGTATAATGTGGCTAAGCATCAAGAAGCCGAGAATCCTTGGTCTTGAGTGGCTTTTAAACTGGGCATGC

ATTTTTTTTGGCGTCGTCCTCACGATTGTGTCGAGAATTGGAAGCATCGTAAATCTCAAACACGGCTTCGAAGAGGAAAA

CCTCAAAGTATTCTACTTCCTGCGATTGCAGCAGTGCAATTCGACCTCCACAAATGCAAGCTGTCCCGTGCGGCATTGA

>413158

ATGAGTAACGCTGCAAGCTACTCCGTCTTTTGGAATTGTCTACGGCGTATACTGCGACCGGAACACCATGCCAAGTGGTG

GTACTCAACCGTTCACATCGTGACGGCCATGGTAGGAGCCGGAGTTCTGAGCCTTCCGTCGACAATGGTCTACCTGGGAT

GGGCTCCTGGAATGATGATGCTGGGCGTTTCGTGGATAATCACACTGGCTACCATGTACCAAATGATTGAGATGCACGAG

GACGAGTCAGGGAGACACGACACTTACCAGTGTCTCGGCCGGAAAGCATTCGGGGACAGGCTTGGCAATCTCATTGTTGG

CTCGCAGCAGATCGTAGGTCAGTTTCTCGTCCATGACAATCGTCTCTCTGATAGCCTCCATCATGTCTTTCAGGAGAATG

TAATTCACATTTCACTCAGCTACTCAACTATAGTTTGGGCAACAGCTATCCGGCTTAAGAGTTCGCAAGCATCGTATGGC

TACTGCAACTTGACATACTACAAAGCCTTTAATGCACTCGGAGAGATTGCGTTTGCCTACGGAGGCCACAGCATTGCTCT

CGAGATCCAAGCGACGATGAGATCGACGAGGCACAAGCCGTCTAAGCTACCAATGTGGAATGGAGTTTTGGTGGCCTATG

TCATGGTTGCCGTTTGCTATTTCCCAGTCGCTGGAGTTGGCTACTGGGCTCTCGGCAACTTGACTTGCTACGAAAACGTT

CTGGACGTCCTCGACAAACCAAAGTGGCTGATAGGAACAGCCAACTTGATGCTTATGCTGCACCTCACAGGGAGTTACCA

GGTTTTTGCTTTGCCGATTTACGAGGGACTGGAGCAAAAGAATATGCCAATCAATGCTTTGATTAGGCCTCTTTACGTAG

GATTTACGTGTTTGGTTGCGGTGATCCTACCTTCGTTTTCCGGGCTTCTGGGATTATTCGGTGGGCTCGCTCTTGGACCA

ACAACGTATTTCCAGTTGCCGTGTATAATGTGGCTAAGCATCAAGAAGCCAAGAGTCCTTGGTCTTGAGTGGCTTTTAAA

TTGGGCATGCATTTTGTTTGGCGTCGTCCTCACGATTGTGTCGGCAATTGGAAGCATCGTAAATCTCAAACACGGCTTCG

AAGAGCAAAATCTCAAAGTATTCTACTTCCCGCGATTGCAGCAGTGCAGCTCGACCTCAACAAACGCAAGCTGTCCCGTG

CGGCATTGA

>270979

ATGGCGCCACACGAGAACGACAGCACCAACAATGGCTCCTCGCCTCCCTCCTCCACGCAGGCATTCAAGGAGTATGTCGA

GGACAAAGGCCACGCTCGAACTGTGAAATCCGTGGACGATTGGCTGCCCGTTGGCAGTGGCTCCAGGAACGCCAAGTGGT

GGTACTCGGCCTTCCATAATGTCACCGCCATGGTTGGAGCTGGAGTTCTTAGCCTCCCCTCGGCGATGGTCTATCTTGGC

TGGGGACCAGGGGTCCTGGTTCTTGTACTCTCGTGGGTGATCACACTCTACACGCTGTGGCAAATGGTGGAAATGCACGA

AATGGTCCCGGGGAAGAGATTTGATCGCTACCACGAACTCGGGCAGGAGGCTTTCGGAGAGAAACTTGGACTGTGGATCG

TCGTGCCCCAGCAGCTCATCGTCGAAGTTGGAGTGGACATTGTCTATATGGTCACAGGAGGGACGTCTCTCATGAGATTC

TACGAGCTCGTCCATTGCAAGCCCGATGACATCAGCTGCAAGCACATCAAGAGAACTTACTGGATCCTCGTCTTTGCCTC

AGTTCACTTCTTCTTGTCCCAGCTCCCAAACTTCAACTCCATCACCGGAGTCTCCTTGGCAGCAGCCGTCATGTCTCTCA

GTTACTCGACGATTGCATGGGTTGCTCCAGTGCATTACGGCCAAGAAGCAAAGCCACCAATGACCAAAGTGAGCTACGCT

TATCCTCACTCTCCATCAGTGGCGAACACTGTGTTTAGAGTTTTCAATGCTCTCGGACAAGTGGCGTTTGCCTACGCCGG

TCACAACGTTGTTCTTGAGATCCAGGCGACGATCCCTTCCTCGCCGCAAAAGCCATCCAAAGTTCCAATGTGGAGGGGAG

TGGTCGTGGCGTATATTGTCGTGGCTATGTGTTACTTCCCGGTTTCCTTGGTTGGATACTGGGCTTTTGGAAATGATACC

AGCTATGACAATGTTCTCCAGCGTCTCGGGAGACCCGAGTGGCTCATCGCGGCTGCCAATCTCATGGTTGTGGTTCACGT

TATTGGAAGCTACCAGATCTATGCGATGCCAGTGTTTGATATGCTGGAAACCGTTCTGGTCAAGAAGTTTCACTTCCCTC

CAGGTGTGATTCTTCGTCTCGTCGCTCGATCACTCTATGTTGCTTTCACTGCCTTTATTGGGATAACTTTTCCTTTTTTT

GGAGACTTGCTGGGCTTTTTCGGAGGTTTTGCGTTTGCTCCCACGACTTACTTTCTCCCGTGTATTATGTGGCTGGCTGT

TTACAAACCCAGAGTTTTCAGTCTTTCGTGGATGGCAAACTGGATTTGTATTGTCTTGGGAGTGCTGCTAATGATTGTAG

CTACTATTGGAGGCTTCAGAAATATCATCATGGATGCATCAACATACAAGTTTTATCAGTAG

>99162

ATGCTCACGCCGCAGGGGAATGGGACTTACACTCCCACTCCATCCAGCACTCGCCCGCCATCGAATCTTGGATCCCCGGC

TCGCCAGCAACCCAATCCATCCTCGCGCCTCCTGCGATCGCCCAAGGTGCTCTTCTCGCCGATTGGAACGCCCATGAGGA

AGGCTCTCACGAATATGAGAGCTTATCTCGAGGATATCGGGCATATCACCAAGCTCAATCCCCAGGAAGCGTGGCTCCCC

ATCACGGCCTCGCGCAATGGGAATGCTTACTACTCGGCGTTCCACAATCTCAACGCGAGCATTGGATTCCAAGCGCTCTT

GCTCCCGGTGGCTCTTACGTTCCTTGGCTGGACTTGGGGAGTTTTGGCTCTGGTGGCAGCTTTTATTTGGCAGCTCTACA

CACTCTGGATCCTCATCCAGCTACACGAAGCTGTCCCTGGCAAACGCCACAGTCGATACGTCGAGCTCGCTCAAGAAGCT

TTTGGACCAAAGCTTGGAGCGTGGCTAGCAATCTTCCCGGTCGTAAATCTCTCGGGAGGAACCGCAACCGGGCTCATCAT

CATCGGCGGTGGAACTCTGGAGCTCTTTTACAGAACCGTGTGCCGGGATTGCCACGGAGGATCACTCACAACCGTGGAGT

GGTACCTCGTCTTCACGATCTTGTGTGCTATCCTCGCGCAGCTTCCAAACCTCAACTCCATCGCTGGAGTTTCTCTGGTT

GGAGCCGTCATGGCCGTGGCTTACACCACCTTGGTCTGGACGCTATCCATCAGCAGGCCGAGACCACCGGGAATAACGTA

TGATATCGTCAAGCCGGATCACACCGCCGGGAACATCTTCTCGGTTCTCAATGCTCTCGGGATCATCGCGTTTGCATTTC

GCGGACACAATCTAGTGCTCGAGATCCAGGGAACGATGCCATCCTCTTTGAAACACCCGGCTAAGTCTCCAATGTGGAGA

GGAGCCAAAGTTGCGTTTGCGATAGTCGCTGCTTGCTACTTCCCGATCGCCATCGCCGGATACTGGGCTTACGGACGAAT

GATGCTTCCCAGTGGAATTCTCTTCTCCATGTACGCGCTCCATCCAGACATCCCAAGCCCGTGGATGGCCATCACCTTCT

TGTTCGTCGTCCTCAACTCGATCAGCAGCTTCCAGATCTACTCCATGCCGATGTTCGACGCGTTCGAGCAGAGTTTCACG

GCCAGGAAGAACAAGCCAACGCCTCTCCTCGCTCGCGTTGCCTTCCGCCTCTTCTTCACCTTCTTCGCGTTCTTCGTCGG

AGTGGCGCTGCCTTTCATCTCCAGCTTCGCGGGGCTGCTAGGCGGACTGACGAGCGTCCCCGTGACGTTCTGCTACCCGT

GCTTCATGTGGCTCAAGATCAAGAAGCCACCGAGGTTCAGCTTCACCTGGTATCTCAACTGGACGCTGGGGATCCTGGGC

ATTGTTTTTAGCATCACCTTCACGGCTGGAGGGATCTGGAGTATAGTCGACAGTGGTCTCACGCTTAACTTCTTCAATCC

ATAA

>98878

ATGGAACGGGAACCCGAGGTCTCTTCGCTGCCTTCCACTCCTCAGAACAACCACAGCATTCCGCCATCTGTTGCCCGTTC

TCCGAGGAGAATGATGCTGTCACCGATGGGAACTCCAATGCGCAAAGCTTTCGGCAACATGAAGTGCTACCTGGAAGAAA

TCGGGCATATTGCAAAACTTAATCCGCAAGACGCATGGCTGCCTATCACCGAGTCTCGAAATGGCAATGCCTACTACTCG

GCCTTCCACAATCTCAACGCTGGGATTGGTTTTCAGTGCCTCCTCCTGCCTGTGGCTTTCAGTTTCCTCGGCTGGTTTTG

GGGAGTTTTAGCGCTGGTGGTAGCGTTCTTGTGGCAGCTCTACACTCTCTGGATTCTCATCAAGCTGCACGAAGTCATAC

CTGGGAGACGATACAATCGCTACATCGAGCTGGCCCAAGCAGCTTTTGGGGAAAGATTGGGATCATGGCTTACTTCATTC

CCGATTATAAGCTTATCAGCTGGAACAGCCGGCGGTCTAATTGCCATCGGTGGCAGCACACTGCATCTCTTCTACAACTT

GGTTTGCATCAAATGCCATGGACAAAGCTTGACAGCCATCGAGTGGTACCTGGTTTTTGCAGTCTTGTGCGCCATTATAG

CACAGCTGCCCAACCTCAACTCGGTCGCAGGAGTTTCTCTCATTGGAGCAGTCATGGCAGTAGCTTACTCGACGATGATA

TGGATCTTGTCAGTTACACGGGATAGACCACCCGGTGTTAGCTACGACGTTGCGAAGCCATACTCATCGGTTGGAGCAGC

ATTCTCCTTCTTGAATGCTCTAGGAGTGATTGCATTCGCCTTTCGGGGCCATAATCTGGCTCTCGAGATCCAGGCAACCA

TGCCTTCGACTCTCAAGCACCCGGCCTACGTTCCCATGTGGAGAGGTAGCAAGGCAGCGTATACTCTCGTGGCGATTTGC

TACTTCCCTCTCGCGATAGGAGGCTACTGGGCTTACGGAAAACTGATGCTCCCGACTGGAATCCTCACCTCCATGTTTGT

CTTCCATCGCTCGGACATATCTCCGGCGTGGCTCGCGACGTGTTTCCTCTTCGTCGTGGTGAGCTCTCTCAGCAACTTCC

AGATTTACTCCATGCCAACGTTCGATCTCGTGGAGCAAACTTACACCGCGAACACCAACAAGCCGTGTCCCAAGCTTCAT

CGGTTTGTGTTTCGGCTGCTCTTTGTCTTCTTTGGCTTCTTCGTGGGGATTGCGTTCCCTTTTATGGCGAGCTTTGGAGG

ACTTTTGGGAGGAGTTTGCAGCGTTCCAGTCACATTTTGCTACCCGTGCTTCATGTGGCTCAAGATAAAAAAGCCACCGA

AGTTGAGCTTCAGCTGGTACCTCAACTGGACGCTTGGAATCTTGAGCGTTGTCTTTACTATTGTCGTCACGATTGGAGGA

ATCTGGAGCATCGTGGACACTGGATTGAAGTTCCAGTTTTTCAAACCACAGTAG

>173454

ATGTTCTTTGAGATTCGTATTTTTTCGATCCAGGGATTCCTCCTCACGAAATGGCGCTCCTGGATCTTGCTCAACGATTC

CTGGTCGAGCAAATGGTGGTACTCGACCGTGCACATTGTCACCGTGACGGTGGGAGCCGGGGTCTTGAGCTTGCCGACGG

TCATGGCCTACTTTGGATGGGCGCTCGGAACTATGCTGCTGGTTGGATTCCTGATCCTGAGCCTGATGTGCTACTGGCAG

CTGATTGAAATGCACGAGACTGAACATGGCCGCCGGTTCGATCGATACCACGAGCTGGGACAGCACATCCTGGGACGCCA

TCTCGGATTCTGGCTCATTGCTCCGCTCCAGGCCATCGCCCAAGTGGGAATTGACACGGTTTACATCATCGCCGGGGCGA

ATTCCCTCGAGCACGTCTACTCACTCTTTGACAAATGCAAAGAGCTGGACGTCCATAAATGCAAGGGCATCAACCTGACT

TACTGGATGATATTGTTCATGGGGGTGCAGCTCTTGCTCTCGCAGCTCCCGCACTTTCAGTCCATCACCTGGGTGTCATT

CATCGCTGCTGTTACAGCCATTGGGTATTGCACACTTGCCTGGGTTGGAATTCTGATTAAGCAACCTGCTTTAAGTTCAG

GTTCAGCTGCATCTGCACCTACACAATGTTTTCAGAATGTTGGGCATGGCTATCCACATGGTTCCAAAGCTCACTTAGCT

TTCGGGATCTTCACCTCTCTCGGGAAGCTGGCATTTGCAGTAGCAGCCGGGCACAACATTGCTCTCGAGATCCAGGCCAC

GATCCCGAGCACTAGTCGCCACCCTTCGAAAAGAGCGATGTGGAGAGGGATTCTGGTGGCCTATCTGGTGGTTGCGTTTT

GCTACTTGCCTGTGGCGCTTGTGGGATACAAGGTTTACGGGGATGAAACACGCGACTTGTGCTCCGGCTTGGACAATGTC

CTGCTCAGGCTCAGGAATCCCAAACCGATGATTGTTCTCGCGGATTTGATGGTTTTCATACATCTGTGTGGCAGCTACCA

AGTGCTTGCAATGCCACTTTTTAGCAATTTTGAGACGCTCGTGGAGAGGATGTTCAAATTTGAGGCGAACCTCAAACATA

GAATGATCATGAGATCGTGTTATGTTGTTCTGACGTTGATGCTGGCCGCAGCGTTTCCTTTCTTTGGTGATCTTGAAGCC

TTCTTTGGAGGTTTTGCTTTAATTCCAACGACTTACGTGATACCTTCTGTTCTCTGGCATTTGAGTAGGAAGCCCGAGCC

ATTTAGTCCGCCATGGATAGCAAACCTGTTGTGTATTTCCTTTGGAATAGCGGTGATGGCTACTTCGACGATAGGTGGCC

TTCGTAACTTGATAATGAAACGGCGGGAACTGGAATTCTTTCAATAA

>SmAAP9A

ATGGCTGTTGGAATGTTTCCTCCTTCGCACGATGAGAGAATCTCTTACGCTGAGAATGGTCACAAGCTCGGATCTTTGGA

GCTCCAGCAGCAGCAGAAAAACGTCGACGACGATGGACGTCCATGCCGCACAGGAACTGTGTGGACAGCCTCGGCCCATG

TCGTGACCGCCGTCATCGGCAGCGGAGTTTTGTCCCTGGCGTGGAGCATGGCCCAGATTGGCTGGATCGCTGGCCCCGTT

GTGCTGCTCATCTTCGCCGCCATCACCTTCTTCACGTCGCTGCTGCTCACGGATTGCTACCGCTCTCCGGACCCCGTCAC

CGGCAAGCGAAACTACAGATACAAGGACGCCGTCAAAGCCAACCTCGGTGAGATCCAGCTCTGGTGCTGCGCGCTTGTCC

AGTACAGCAACTTGATGGGAACGGCCATCGGTTACACCATCACTGCCTCGATAAGTATGGTGGCCATCAATCGATCCGAC

TGCTTCCACGCCAAGGGCCACAATGGCGCCTGTAACACCTCCAACAATCTCTACATGGCTCTGTTTGGAGTGGTCCAGCT

GATGCTGTCACAGATCCCAAACTTCCACAAGCTCTGGTGGCTGTCCATCGTCGCGGCCGTTATGTCGTTCTCGTACTCCG

GGATCGGCCTGGGACTCGGCATCAGCAAGATCATCGAGAATGGCCATCTCTTAGGATCAGCAACCGGCGTCCCCATTGGT

TTGACTCTCGGCAGTGTAACACCCGCCAAGAAAGTCTGGAGAGTCTTCCAGGCCCTTGGCAACATCGCGTTTGCGTATTC

TTTCTCCACCGTTCTCATCGAAATCCAGGACACCATCAAGTCACCACCAGCCGAGAACAAAACGATGAAGAAGGCGACGC

TGATTGGGATCATCACCACCACCACCTTCTACTTGTCGGTCGGATGCTTCGGCTACGGTGCCTTTGGAAATGGTGCTCGG

GGCAATCTTCTCACCGGCTTCGGCTTCTACGACCCTTACTGGCTCGTCGACTTTGCAAATGCCTGCATCGTTGTCCATCT

TGTCGGAGCTTATCAGGTCTTCAGTCAGCCACTGTTTGAGTTTGTGGAGTCGACTGCTGCGAACAAGTGGCCAAAATCAG

GCTGCATCCACACTGAGCACGCCATCCGAATCCCGTTCGTTGGAACCTGGCGAGTTAACGTCTTCCGGCTCCTGTGGAGA

ACCATGTACGTCATCTTCACCACCATTGCTGCAATGCTGCTTCCCTTCTTCAACGACATCGTCGGTTTGATCGGTGCTGC

TGGATTCTGGCCGCTCACTGTCTACTTCCCCATCGAGATGTTCATCAAGCAAAAGCGGATCGAGAGCTGGTCCTGGTCCT

GGGTTGCTCTGAAGACCATTAGTGCAGCCTGCCTGATGATATCCATCGCTGCCGGGATCGGTTCTATCGAAGGTATTCTC

CATAGCCTCGAAAAGTACACGCCGTTCAAGACCACGTACTGA

>SmAAP10

ATGACCGCGGGGCTGGATTCTTCTCTCCCGAATGGCGCCGAGGCCAGCATCGACATGCGATTCCATGGCGGCGCCGGAGG

CAGCGAGAAACAAGTGGAGCGCACAGGGAACGTTTGCACTGCCTCCGCTCATGTAATCACTGCCGTGATTGGATCCGGAG

TGTTGTCCCTTGCATGGAGCATCGCCCAGTTTGGATGGGTTCCCGGCCCAGCTATTCTATTTATTTTTTCCATCGTGACC

TTCTATGCGTCTTTGCTCCTCGCGGATTGCTACAGGAGCCCAGACCCGGCCTTTGGGAGAAGGAACACTACCTACATTGA

TGCCGTCAAGAACATCTTGGGTGGCAGGCAAGAATGGTTTTGCGGTCTCGCCCAGTATGGCAATCTCATCGGTGCTACTA

TTGGATACACCATAACTTCTGGGAAGAGCATGGTGGCGATTAGCAAAGGTCACTGTTTACGCCATAACCGGCACCTAAGC

AATCCATCATCGTGCAACATCCATGACGGGAGGTACTTGCTCGTTTTCGGAGCTGCTCAGCTCCTCTTCTCGCAGATACC

AGACATACACCAGATTTGGTGGCTTTCCATCGTCGCGTCGATCATGTCTTTTTCGTATTCGTTTGTCGGGCTGGGTCTCA

GCGCTGGTCAAGCAGTACATGGAACTCAAGGTACCGCATTTGGGATTGGCATTGGACCTGGACCGCACTCTGTCAGCTCA

GCTGATAAAGTCTGGGGTATACTCCAGGCGCTTGGGAATATTGCTTTTGCTTACTCGTTCTCATCCATCCTTATCGAGAT

TCAAGATACACTCAAGTCGCCCCCATCCGAGAACGTAAGCATGAAGAGAGCTACGTCAATCGGTGTTCTCGTCACCACCA

TATTCTACATGGCTGTCGGCTGCGTCGGCTACGCAGCTTTTGGAAACGATGCTCCGGGAAATCTCTTGACCGGATTCGCG

CACAGCAAGTTGTTCTGGCTTGTCGACTTTGCGAACATCTGCATCATCATCCATCTCGTCGGCGGCTATCAGGTATATGC

TCAACCAGTGTTCGCGCTGGGGGAATGGTACGCCTCCCAGAAGTGGCCAAAGTCGAACCTCGTAAACAGGGAATACTCCG

TAACGGTGCTCACCCCGAGGATTGGAGTTTTCAGGTTCACCATCTTCAAGCTCTTCTGGCGGACCTTGTTCGTGCTCTTC

ACGACCATTGTCTCGCTCGTGTTCCCGTTTTTCAACGCCGTCATCGGCCTGGTTGGAGCCATCACCTTCTGGCCGCTCAC

CGTCTACTTCCCGGTGGAGATGTACTCGAAGCAGTCGGGCGTGCGGAGGTGGTCGTGCAAGGCTATGGCACTCCAGAGCT

TGAGCTTTGTGTGCTTCCTCGTCTCGCTGTCCGCGGCCGTGGGATCGGTACAGGGAATCATCAGCTCGTCCAGGCGCTAC

AAGCCGTTTGAGTTCTAA

>SmAAP9B

ATGGAGGCGGCGGGCGCTCCTCCTCCGCCAGCCGCGATCGACAAGGACGACGACGGCCGCCCCAAGCGCCAAGGGACGGT

GTGGTCGGCGGCCGCTCACGTAATCACTGGCGTGATTGGATCCGGGGTCCTCTCCCTGGCCTGGAGCTTCGCGCAGCTGG

GATGGATCGCCGGCCCGATTGTGCTGCTCATCTTTGCTTATTTGACTTATTACACCTCGGCCTTGCTAGCCGATTGCTAC

AGGTTCCCGGATCCCACCACTGGGAAGCGGAACTATCGATACAAAGACGCTGTGAAAGTCACATTGGGTGAAGTGGAATT

GTGGCTGTGCGCGCTGGCTCAGTACAGTAATTTGGCTGCCACTGCGGTGGGATACACTGTTACCGGTGCTTTGAGCATGG

CAGCAATCGCTCGAGCGAATTGCTTTCACACCAAAGGCTCGAAAGCGTTGGGATGCGGTGTCTCTGTCAATCTGTACGTG

ACGGCCTTCGGGTTGATCCAGCTCGTTTTCTCGCAAATCCCAAACTTCCACGAGCTATGGTGGCTTTCGTACCTTGCGAC

AGCCATGTCCTTCACGTATTCAACAATCGTCTTGGTCCTGGGGCTGGCGAAACTTATAGGCATTCCTGGTGGTCTAGTGA

CGACCCCGGCTCAAAAGACTTGGGCGGTGTTCCAGGCGCTGGGAAACGTTGCGTTTGCCTACTCTTTTTCAATGATTCTG

ATCGAAATTCAGGACACGCTAAGATCAACACCTCCAGAGAACAAGACAATGAAGAAAGCAACACTCGTTGGAGTATTAGC

CACGACTGCCTTCTACATGTCAATCGCCTGTGTCAACTACGCGGCCTTTGGTGATTCTGCTCCTGGAAACTTACTGAGCC

AAGGCTTCGAAAAGCCTTACTGGCTCATTGACTTTTCAAACGCTTGCATCGTTCTCCACTTGGTTGGAGCTTACCAGGTG

TACAGTCAGCCTCTATTCGATTTCGTCGAAGCCTGGGCACTGGAGAAATGGCCACACAGCGCTCTCAACACCACTCACAA

GATCAAGCTCCTTCACTGGAGGTACTCCACCACGCTCTTCCGGCTCGTCTGGAGAAGCCTCTTCGTGATAGCAACCACAG

TGATAGCCATGGCGATCCCCTTCTTCAACGACGTGCTGGGTTTGCTGGGCGCGATGGGCTTCTGGCCTCTCACCGTCTAC

TTCCCGATCCAGATGCACATCAAGCAAGCCCAGATCAAAACGTGGTCGATGCGGTGGCTCAAGCTCCAAGCCATCAGCGC

GTTTTGTCTAGTCATCTCCATCGCCGCGGGTATCGGATCCATCGAAGGGATCTACCAGGACCTCAAGGCTTATACTCCCT

TCCACGCTAATTTCTAA

>156907

ATGGGGGAAGAGGCTGCGCTCGCCAAAGAGAAGCTCGACGCTGGAGCGGCATTCGTGCTAGTATCCAAAGGGACATGGCT

ACACGCAGCGTACCATCTCACCACAGCCATCGTTGGTCCAGCGATTCTAAGCCTTCCATACGCTTTCGCGTCACTCGGCT

GGGAATTGGGAGTTTTGGCCCTGACAATGGGAGCTCTTGTCACCTTCTATGGCTACAATCTCGTCTCCACGCTGCTCGAG

CAAGCGGATCAAAGAGGCCAGAGACACTTGCGCTTGGGAGATTTGGCGGTGGACATTCTTGGACCAAAGTGGAGTAAATA

CGTTGTTTTTCCGCAGATGGTCATCAGCTTTGGCATCGTTGTTGGCAGCAATCTCTTGTGCGGACAAGGAATGCTCAAAA

TCTACGAGAACTTGGTGAAAGATGGCGACCTTAAGCTTTACCACTTCGTGATGATCAGCGCGTCGATCATGATCATCCTG

TCCCAGCTTCCATCTTTCCACTCGCTTCGCTACATCAGCCTCGCCTCGGCGCTCCTCTCCATGGGATACAGCCTTGGCGT

GGTGGCGGCGTGCATCTACGCAGGTCACTCTAAGCGAGCGCCACCAAAGGATTACTCCATCGTTGGGAGCACCAGCGCTA

GAGTTTTCCACGCTTTCAATGGCCTCTCGATCATGGCCTCGACGTATGGAGTGAGCATTATACCCGAGATCCAGGCGACT

ATAGCTTCTCCAGTGTCCGGGAAGATGTTTAAAGGATTGCTGCTTTGCTACGCGGTCGTGGTGACGACGTTCTTCTCGGT

TTCCATCTCGGGCTACTGGGCTTTTGGCAACAAAGCCACTGGAAATCTCTTCGACAACTTCATTCCAGACGACAACACAA

CGCTAGCTCCCGACTGGCTCTTGTTCCTGATCATTCTCTTCATCGTCATCCAGCTCCTCGCAATCGCAGTGGTTTATTCG

CAGCCTCTCTTTGATGTCTTCGAGACTGCTCTTTCCGACGTCAAAAGGCCAATCTTCTCGTTTAGAAACTTGCTCCCCCG

GCTCGCAGTGAGATCACTCTACATAGTTTTGGCAGCGTTTCTAGCGGCAATGCTCCCGTTCTTCGGAGATCTCAATGCGT

TCATAGGCGCCGTGGGGTTCTTGCCTCTAGCTTTCATTCTCCCCCCGGTTCTCTACAACATCAAGTGTAAGCCATCGCCA

GGGACGGTGGTCTTCTGGGTAAACACAGCCATTATCGTGGTGTACGGTGCCATGGCGGTCATGGGAAGCGTTTCTTCCGT

ACGCCAGATCGTCCTGGACGCTCACAAGTTCAAGGTCTTCTCCAATAACACAAGCTAA

>127260

GTGGACGAATGGCTGCCAGTGACGAGCTCGAGGAACGCCAAATGGTGGTACTCCGCGTTTCACAACGTAACCGCGATGGT

AGGATCGGGCGTGCTGGCGTTACCCTCGGCTATGGTTTATCTGGGATGGGGGCCTGGGATCTTCGTGCTCCTCCTCTCCT

GGACCGTCACGCTCTACACGCTATGGCAAATGGTGGAGATGCACGAGATGGTGGAGGGCAAGCGCTTCGATCGCTACCAC

GAGCTCGCGCAGGAGGCGTTTGGCGAGCGCCTGGGGCTGTGGATCGTGGTGCCGCAGCAGCTCATCGTCGAGGTCGGCGT

CGACATCGTCTACATGGTCACCGGCGGGAAATCGCTCAAGCGATTCTACGAGCTGGTGAGCTGCGCGCCCGACGCGACCG

GATGCAAGCACATCCGGCAGTCGTATTGGATCCTGGTGTTCGCGTCGATCCACTTCGTGCTGGCGCAGCTCCCCAATTTC

AACTCCATCTCGGGGATTTCGCTCTCCGCGGCGGTCATGTCTCTCAGCTATTCCACGATCGCCTGGACGACCGCGATCCC

CAATGCCGGGGGGCCGGATGTGAGCTACAGCTATCCTCACTCCCCATCGGCAGCCAACACGGTGTTCAAGGTTTTCAATG

CGCTGGGAATGATCGCGTTCGCCTATGCCGGGCACAACGTTGTTCTTGAGATCCAGGCAACCATTCCATCCTCGCCGAGC

AAGCCGTCCAAGGGCCCGATGTGGAAGGGAGTGGTGGTGGCGTACATGGTCGTGGCGATTTGCTACTTCCCGGTGGCTCT

CATCGGCTACTGGGCATTTGGGAACGATACTTCTTACGACAACATTCTCCAACACATCGGTACTCCACACTGGCTCATCG

CTGCTGCCAATCTCATGCTCGTTGTTCATGTCATTGGCAGCTACCAGATTTACGCAATGCCTGTGTTTGACATGCTCGAG

ACGCTTCTTGTCAAGAAGCTCCACTTGCCTCCTGGCGTTTGCCTCCGTCTCATCGCCAGAACAGTCTACGTTGCATTTAC

AGCGTTTGTAGCGATCACAATCCCGTTCTTCGGAAATCTTCTCGGTTTCTTTGGAGGATTTGCACTAGCGCCCACAACTT

ATTTCCTACCGTGTATAATATGGCTGGCAGTGTACAAACCCAAGCGATTCAGCTTTTCATGGCTCGCAAACTGGATATCG

ATCGTGCTGGGCGTGCTGCTAATGATAGCAGCCACAATCGGGGGATTCCGGAACTTGGTAATGGATGCGTCGACTTACAA

GTTCTATCAGTGA

>426884

ATGAGGGCCTCTCTATCAGCCCGTGATCAACAGGTGGGTGGTACAGAATCTCATGAGCGCGAAGATCGCATGAGTAACGC

TGCAAGCTACTCCGTCTTTCGGAATTGTCAACGGCGTATACTGCGACCGGAACACAGTGGTGGTACTCAACCGTTCACAT

CGCGACGGCTATGGGCTCCTGGAATGATGATGCTGGGCGTTTCGTGGATAATCACACTGGCTACCATGTACCAATTGATC

GAGATGCACGAGGATGAAAAAGCATTCGGGGACAGGCTTGGGTTCATAGTTGGTTTGCAGCAGATCGTAGTCCAGGTGGC

TGCCAACATTGCTTACTTGGTAACTGGCGGCCAGGCGCTCAAGAGGTTCGGGGATCTTGTTCTGAGTCGCGAGATTCAGT

ACGGGAAGTTTGAGCTTGCTGTGGCATGGATATCCGCGTTTGCTGGAGTTCAGGCGGTACTGTCGTTGTTTGCCAGTTTG

TCGTCCATGACAATCGTCTCTCTGGTGGCCGCCATCATGTCTTTCAGCTACTCGACTATAATTTGGGCAATAGCTATACG

GCTTAAGAGTTCGCAAGTATCGTATGGCTACTGCAACTGGAGATACTACAGAGCCTCTAATGCACTCGGAGAGATTGCGT

TTGCCTACGGAGGCCACAACGTTGCTCTCGAGATCCAAGCGACGATGAGATCGACGAGGCACAAGCCGTCCAAGCTACCA

ATGTGGAATGGAGTTTTGGTGGCCTATGTCATGGTTGCCGTTTGCTATTTCCCAGTCGCTGGAGTTGGCTACTGGGCTCT

CGGCAACTTGACTTGCTACGAAAACGTTCTGGACGTCCTAGACAAACCAAAGTGGCTGATAGGAACAGCCAACTTGATGC

TTATGCTGCATCTCACAGGGAGTTACCAGGATTTACGTGTTGGTTGCGGTGATCATACCTTCGTTTGGAGGGCATCTGGG

ATTATTCGGTGGGCTCGCTCTTGGACCAACAACCAGTCGCCGTGTATAATGTGGCTAAGCATCAAGAAGCCAAGAGTCCT

TGGTCTTGAGTGGCTTTTAAACTGGGTAAAAATCTCATCATTTTTAGATCGGAAGCTTTTGACATGGTGGTATGAACCAA

TGCAGGCATGCATTTTGTTTGGCGTCGTCCTCACGATTGTGTCGAGAATTGGAAGCATCGTAAATCTCAAACACGGCTTC

GAAGAGGAAAACCTCAAAGTATTCTACTTCCCGCGATTGCAGCAGTGCAATTCGACCTCAACAAACGCAATCTGTCCCGT

GCGGCATTGA

>127270

ATGAGTAACGCTGCAAGCTACTTCGTCTTTTGGAATTGTCTACGGCGTATACTGCGACCGGAACACAATGCTAAGTGGTG

GTACTCAACCGTTCACATCGTGACGGCTATGGTAGGAGCTGGAGTTCTGAGCCTTCCGTCCACAATGGTCTACCTGGGAT

GGGCTCCTGGAATGATGATGCTGGGCGTTTCGTGGATAATCACACTGGCTACCATGTACCAAATGATTGAGATGCACGAG

GACGAGTCAGGGAGACACGACACTTACCAGTGTCTCGGCCGGAAAGCATTCGGGGACAGGCTTGGGAATCTCATAGTTGG

CTCGCAGCAGATCGTAGTTCAGGTGACCGCCAACATTGCTTACTTGGTAACTGGCGGCCAGGCGCTCAAGAGGTTCGGGG

ATCTTGTTCTGAATCGCGAGATTCAGTACGGGAAGTTTGAGCTTGCTGTGGCATGGATATCCGCGTTTGCTGGAGTTCAG

GCGGTGCTGTCGTTGTTTGCCAGTTTCTCGTCCATGACAATCGTCTCTCTGATGGCCTCCATCATGTCTTTCAGCTACTC

GACTATAGTTTGGGCAACAGCTATCCGGCTTAAGAGTTCGCAAGCATCGTATGGCTACTGCAACTTGACATACTACAGAG

CCTTTAATGCACTCGGAGAGATTGCGTTTGCCTACGGAGGCCACAACGTTGCTCTCGAGATCCAAGCGACGATGAGATCG

ACGAGGCACAAGCCGTCCAAGCTACCAATGTGGAATGGAGTTTTGGTGGCCTATGTCATGGTTGCCGTTTGCTATTTCCC

AGTCGCTGGAGTTGGCTACTGGGCTCTCGGCAACTTGACTTGCTACGAAAACGTTCTGGACGTCCTCGACAAACCAAAGT

GGCTGATAGGAACAGCCAACTTGATGCTTATGCTGCATCTCACAGGGAGTTACCAGGTTTTTGCTTTACCGATTTATGAT

GCTCTCACTTGCTGGCTGGAGCAAAAGAAACTGCCAATCAATGCTTGGATTAGGCCTCTTTACGTAGGATTTACGTGTTT

GGTTGCGGTGATCATACCTTCGTTTGCCGGGCTTCTGGGATTATTCGGTGGGCTCGCTCTTGGACCAACAACGTATTTCT

TGCCGTGTATAATGTGGCTAAGCATCAAGAAGCCAAGAGTCCTTGGTCTTGAGTGGCTTTTAAACTGGGCATGCATTTTG

TTTGGCGTCGTCCTCACGATTGTGTCGGCAATTGGAAGCATCGTAAATCTCAAGCACGGCTTCGAAGAGCAAAATCTCAA

AGTATTCTACTTCCCGCGATTGCAGCAGTGCAACTCGACCTCAACAAACGCAAGCTGTCCCGTGCGGCATTGA

>pa_MA_101691g0010

ATGACAATGACTCAGATTCATCATCCAACGTTGGAGGTTTCAATAGAATCAAGTGGCTTGCAGAGCGGAAAGGTTTCAGATTCCAAGAAGTTCGACGACGATGGGTGCATCAAACGCCGAGGCACATTGGGGACAACAAGTGCGCATATTATCACAGCAGTGATCGGGTCCGGCGTGCTCTCATTGGCATGGGCAACAGCTCAACTAGGATGGATTGGCGGACCCGCAGCCATGCTTGCTTTCACCTTTGTCACATACTATACGTCTTGCCTGCTTTCAGACTGCTACAGGTCTCCGGATGCAGTTACTGGAAAGAGAAATTATACCTATAGTCTTGCGGTGAGAGCCAATCTAGCTGCATCCATCAGCATGACGGCCATTCAGAAGTCAAATTGCTTCAGCACTCATGGAGATGACTACCCATGCCACGTCTCTAGTAATCCATACATGATAGTCTTCGGAGTGGTAGAGATCCTACTTTCTCAGATACCAGACTTTGAGGAAATATGGTGGCTCTCTATCGTCGCTGCGGTCATGTCATTCACGTATTCTACCATCGGACTTGGCCTCGGTATCGCCAAAGTAGCAGAGGTGGGACACTTTAGAGGGAGTCTCACAGGAGCCACGAGTGGTACAGTAACTAAAGCTGACAAAATATGGAATGCGTTCCAAGCTCTGGGTAACATTGCATTTGCTTACTCCTATTCCCTGGTTCTCATAGAAATTCAGAACACAATAAAATCGCCTCCTGCAGAGAACATGACGATGAAGAAAGCAACGCTACTGGGTGTGGCAACGACAACGGTGTTCTACATGCTGTGTGGTTGCATGGGCTACGCAGCTTTTGGTGAGAATGCACCAGGAAACCTCTTGACAGGCTTTGGCTTTTACAATCCCTTTTGGTTGATTGACATTGCGAACGCTGCCATTGTCATCCACTTAATCGGTGCATATCAAGTGAATCTGTTCAGGCTGGTGTGGAGGACATGTTTTGTGGTGAGCACCACATTGGTTTCCATGCTGCTTCCATTTTTCAATAATGTTGTGGGACTTCTTGGAGCAGTGGCATTCTGGCCATTGACAGTATACTTTCCAGTGAGCATGTACATAGCAAGGAATAAGATCCGGCGCTGGTCTTCTAGGTGGGTCGCTATGCAAATATTGAGTGGATTTTGTTTCGTTATTTCAGTGGCAGCTGCCAGCGGATCCGTAGTGGGTATTGCTGAAGCTTTAAAAGCGTACAAGCCCTTCAAGACAGCGTAG

>pa_MA_14300g0010

ATGGCAGAAGTACGAGAGCCTTTATGTCCAAACGAGGCTGCCATGGATGAAAAGTATGGACAACATCATCCACATTCATATTGCAATTTCCCTAACGAGGACGAACAAGTCCAACGAACAGGAAGCTTATGGACAGCAGTGGCTCACATAATTACGTCAGTGATAGGGGCAGGGGTATTATCATTGTCATGGAGTATTGCGCAGTTGGGATGGATTGCAGGGCCTGCAACTATGATTGTCTTTGCTCTTATCACATTGTATTCTGCTTCCTTGATTGTGGATTGTTACAGGTTCCCAGATCCAATCACTGGGCCCATCAGAAATGGTTCTTACAGGGATGCAGTACGAGTGAATCTGGGTGAAAGAAGAGCACGGTTGTGTGGATTAGTTCAATATGTGTACTTCTATGGAATTTGCGTTGCTTACACAATCACTGCTTCAATGAGCATCAGAGCAATCAGGCAATCAAATTGTTACCACAGAAATGGACATGAGTCTCCATGCCAGTTCTCAGAGCAAACATATATCATCCTTTATGGAGTAATACAAGTTTTCCTATCTCAATTACCAAACTTTCATAATCTTTGGGGACTTTCAATAGTTGCAGCAATGATGTCCTTTTCGTATGCAACAATAGGATTCGGTCTCGGCGTTGCCAAAGTAATTGAAAATGGGGAGATTTATGGAAATCTGGGTGGAATCAGCTGGAGCACCTCTATTACTAGTGCCCAGAAAGTTTGGCGGATCCTCCAAGCTCTTGGTGACATTGCATTTGCATTTCCATATTCTTCACTTGTCCTTGAGATTCAGGATACTCTGAAGTCACCACCCCCAGAAAACGGAACTATGCAGAAAGCATCCTTGTTTTCTATCATGGTAACAACATCCTTCTATATGCTGTGTGGTTTTCTGGGGTATGCAGCCTTTGGGGAAAATGCACCTGGAAACCTCTTGACAGGATTTGGATTCTATGAGCCTTACTGGCTGATTGACTTTGCTAATGCATGCATTGTGGTTCACCTGGTTGGAGCATACCAAGTATTTTGTCAACCAATGTTTGCTTTCATTGAAGGTTGGGCATCTCATAAATGGCCAAACAACAAATTCATCAATGAGGAATGTTCCATACGTATTCCGTTATTTGGGTTGTACAATGTCAATCTGTTGAGGCTGTGTTGGAGAACTGCCTTTGTGGTGTCGACTACTGTCATTGCAATCTTATTTCCATTATTCAATGACGTGCTGGCAATATTGGGTGCTCTGAACTTTTGGCCATTGATAGTTTACTTTCCAGTGGAGATGTACATTTCTCAGAATAAAGTCCGACCTTTGGCACTCAAGTGGACCCTCTTGCAAGCTTTCAGCTTCATTTCTTTTCTTCTATCGGTGGGAGCTGCTCTTGGTGCCATAGAAGGCCTTGTGAATGACACTGACACATAG

>pa_MA_165784g0010

ATGGAGGAGCTGACCGACATTACAGCTATGAATGTAGAGGCTGGACGCCACGACAGTGAAAAACAACATGATATTAGTTCTGACGATGGGCGTGTTAGAACAGGGGATGTGCGGACTGCAACAGCTCATGTGATTACTGCGGTAATAGGTGCAGGAGTTTTATCTTTGCCATGGAGTGTTGCTCAGCTTGGATGGCTTCTAGGGACACCCATTCTCCTGGCTTTCTCGTGGGTTACATATTACAGTGCTATTCTACTTACAGATTGCTATAGGTCACCTCACCCAGTCACTGGAGCCAGGAACTACAAATACAGGGATGCTGTGAAGGCCATTCTAGGCGGCTATAAGGTCTCGCTATGTATGTGGGCTCAATATTCAAATCTTTACGGATGTCTAATTGGTTACACAATTACGGCAGCTACCAGCATGATGGCAATAAAAAAGGCGGGATGCTTTCATGAACGAGGGAAGAATGCCCCTTGTCGTATATCAGGCAATCTATATATGATTATCTTTGGTGTGCTAGAACTTATATTGTCTCAGCTACCGAGTTTGGAGGAAATTTCGTGGCTTTCAATCGTTGCAGCCATTATGTCTTTTGCATACTCTTCTATTGGGCTCGGTCTTAGCATAGCAAAAGTTGCAGGTAGAAAACATCTCTCTGGAAGCCTAATGGGAGTACCTATCGTTGAACTTTCACCAGCAAAGAAAACCTGGTATATGTTCCAAGCATTGGGCAACATGGCTTTTTCCTACTCATTTTCAACTGTTCTTATTGAAATACAGGATACCCTGAAATCTCCCCCGCCGGAGAACAAAACAATGAAAAAGGCAACTAAAATGGGACTAAGTGTGACGACGCTTTTCTACATGTCCATAGGCTTGGTAGGGTATGCAGCTTTCGGGAATGATGCTCCTGGAAATATGCTAACTGGATTTGGATTCTACGAACCATTCTGGTTGATTGACATTGGTAACCTCTGCATAGTTATTCACATAGTGGGTGCATATCAGGTTTTCTCTCAGCCTGTATTTGCAGTAATTGAAGACTGGGTTCGTGGTAGATGGAAAATGAGTGGCTTTGTCCATAGGGTTTACACTATAAAGTTTCCATTTCGTGGATCGGTTCGGTTCACAATTTTTCGTCTGTTACTGAGGAGCTCACTTGTGGTGTCGACGAGTCTGATTGCAATGCTTCTGCCATTTTTCAACGCCATAATGGGGCTTATCGGTGCCATGTCATTCTGGCCACTCACGGTTTATTTTCCCGTTGAAATGTATATTGTTCAAACCAGTGTTAAAAAGTGGAGTCGAAAATGGATCTTCCTGCAGTTCCTTAGCTTAGTCTGCCTTCTTGTAACTCTGGTTGCTGCCATAGGATCAGTAGCAGGGATCACTATGGCTCTCAAGCATGCCACTGTTTTCAACATGAAATATTAG

>pa_MA_402129g0010

ATGAGAAGGGACAGCGTAATAGAATATCAGATTTCTGCAATGGAGTCCGGTTCAATTGAAGGTCATGGCGGTCACAGGACTTTGGTTGTGGGGAAGGAAATTATGTTTAGCCCTCTTATAACGGATGAAGATGGCCACCCACAGCGCACTGGTGATGTTTGGACTGCATCTTCCCACGTGATAACAGCAGTAATTGGGTCCGGCGTGCTATCGTTGGCATGGAGTATGTCACAGCTGGGTTGGATAGCAGGGCCACTGGTTCTGTTGGCCTTTTCCTTTGTGACCTATTACACCTCCATGTTGCTCGCTGATACGTATCGGTCGCCTGATCCTGTTACTGGGAGGCGCAACTATACGTATACAGATGCCGTCACAGCCATTCTTGGTGGGAAAAGGGTCTTTCTATGCGGGATTGTACAGTATCTGAATCTGGTGGGTACAACTATTGGATATACCATCACTGCTTCTATAAGCATGGTGGCAATAGGGCGATCAGACTGTTTTCACGAAAAAGGGAGGGAATCGCCTTGCCATATATCAAACAATTTGTACATGGCGATTTTTGGAGCAGCCCAAGTGTTACTCTCACAGATTCCTAATTTCAGCAAAATTTGGTGGTTGTCAACACTTGCAGCTGTAATGTCTCTTACGTATTCGTTCATTGGACTCGGTCTAGGAATCGGCATGGCCACAGAGAAAGGGCATTCTCATGGTAGCTTAGGAGGAGTAGGGATCGCTGGTGTCCAAAAATCAGTTGATAAAATCTGGAATATCTTTCAAGCTCTTGGGAATATTGCATTTGCTTATTCCTTTTCAATGATTCTAATTGAAATACAGGACACTGTAAAATCTCCACCAGCAGAGAACAAGACAATGAAAAAGGCATCGTTCATTGGCGTTGTGGTGACCACCATGTTCTATATATCTGTTGGCTGTGCTGGATATGCAGCATTTGGAGACCATGCTCCTGGCAATCTGCTAACGGGTTTCGGATTCTACAATCCCTTCTGGCTTGTCGACATAGCCAACATTTGCATTGTTATTCACTTGGTGGGCGCTTACCAGGTGTTCTGCCAACCTCTGTATGCGTTCGTGGAGGAATGGAGCGCCAATACATGGACCAAGAGCTGCTTCATCCAAAACGAATACAAAGTGCCTATCCCCGGATTGGGAGAGTTTAAGCTCAATCTGTTTCGGTTGGTGTGGAGGACTTGTTTTGTGGTTTTTACTACAGTGGTGTCGATGGTTCTTCCCTTCTTCAATGCCATCATGGGCGTGCTTGGAGCTATAGCCTTCTTCCCCCTCACAGTTTATTTCCCCATACAGATGCACATAGCTCAAACTAAGCTCAGGCAATGGAGCTTCAAGTGGGTGGCCCTGCAGCTTATGTGTGTACTGTGCTTCTTTGTGACAATGGCAGCACTCGTGGGTTCCATAGCTGGTGTTGTGGAAGTTCTTCAGCATTACACTCCATTTAAAACCACCTATTGA

>pa_MA_43770g0010

ATGGCTCAGAATGACGCAGCTCCTCGTTGCTACTTGCAAGTAGAATTACAAGGCAATATGGAGGAGATTTGCACAGAAGCCGATCACTTGGAGAACGGAAAGGTTTCAGATTTCAAGAACATTGACGACGATGGCCGCATGAAACGCCAAGGCACATTGTGGACAGCAAGTGCGCATATTATCACAGCGGTCATAGGATCTGGAGTGCTCTCATTGGCATGGGCAACAGCTCAATTAGGATGGATTGCCGGACCCACAGCCATGCTTGTTTTCTCCTTTGTCACATATTATACGTCTTGCCTTCTTGCAGACTGCTACAGGTCTCCTGATCCAGTTACTGGAAAGCGAAATTATACCTATATTCATGCTGTGAAAGCCAATCTAGATGGCTTCCAGGTGTGGATATGCGGTTTTACTCAGTACGTGAATCTATTCGGAACCGCGATTGGCTATACAATAGCTGCATCCATCAGTATGACGGCCATTGAGAGGTCAAATTGCTTCAACACTCATGGACATGAGGATCGATGCCACGCCTCCAACAATCCATACATGATTGTCTTTGGAATAGTAGAGATCCTACTTTCTCAGATACCAGACTTCGACCAAATATCGTGGCTCTCTATAGTTGCTGCGGTGATGTCATTCACCTATTCTACTATTGGACTTGGCCTCGGTATCGCCAAAGTAGTAGAGGTAGGACACTTTAGAGGGAGCGTCACGGGAGCCACCATTGGTACAGTAACTAAAGCTGAGAAAATATGGGATACGTTCCAAGCTCTTGGGAACATTGCATTTGCTTACTCCTATTCACTTATTCTCATAGAAATTCAGGACACAATAAAATCGCCTCCAGCAGAGAACAAGACAATGAAGAAAGCAACGCTAGTGGGCGTGGCAACCACAACGGTGTTCTACATGCTGTGTGGTTGCATGGGCTACGCAGCTTTTGGTCACAATGCGCCAGGAAACCTGTTGACAGGCTTTGGCTTTTACAATCCCTTTTGGTTGGTTGACATTGCCAACGCTGCTATTGTAATCCACTTAGTTGGTGCATATCAAGTTTTCTGCCAACCATTGTTTGCATTTATTGAAGGGTGGTCCGGGAAAAAGTGGCAATACAGTGACTTTATCAACAAAGAATACGCTCTGGCAATACCTTTGTATGGGCCATATAAGGTGAATCTGTTCAGGCTGGTGTGGAGGACAGGTTTTGTGGTGAGCACCACATTGATTTCCATGCTGCTTCCATTCTTCAACAATGTTGTGGGACTTCTGGGCGCAGTGGCATTCTGGCCATTGACAGTTTACTTTCCAGTGACAATGTACATAGCACAGAATAAGATCCGGCGCTGGTCTTCTAGGTGGGTTGCTATGCAAATTTTGAGTGCCGTCTGTCTCCTTGTTTCAATAGCAGCCGCCAGCGGATCCATAGTGGGTATTGTTGAAGCTTTAAAAACGTACAAACCCTTCCAAACAACATATTGA

>pa_MA_6860g0010

ATGGAGGCCCATAGCTCAGAAGTTCTCTCCCGAACGGGCACGCTATGGACGGCAGTGGCTCATATCATCACAGGAGTCATAGGGGCAGGAGTGTTATCGTTGGCATGGAGTGTTGCCCAATTGGGATGGATTGCAGGGCCTGTAGCTCTGGTTGTTTTTGCTCTTGTAACGTTGTATTCCACTTTCTTGGTCTCGGATTGTTACAGGTTCCCAGATCCCGTCTCTGGGCCCAAGAGAAACTCTTGTTACAGAGACGCAGTTCGAGTGAATTTGGGTACAAGAAGAGCATGGCTGTGTGGACTAGTTCAATACGCCTCCTTCTATGGAGTCTGCGTTGCTTACACCATCACTGCTTCAATCAGTGTGAGAGCAATCAGGCGATCAAATTGTTATCACAAATATGGACATGACGCTTCATGCCATTTCCCAGATCTCACCTATATGATCCTCTTTGGAGTTATACAAGTTATCTTGTGTCAAATACCAAACTTTCATAAACTATGGGGCCTTTCAATACTTGCAGCAACAATGTCCTTTAGCTATGCAACATTAGGATTTGGCCTCGGCATGGCCAAAGTTATTGAAAATGGAGAGATTAAAGGAAATCTGAGTGGAATCAGCGCGAGCACCTCTCTTACTCAAGCCCAAAAAGTTTGGAGGATGCTCCAAGGTCTTGGCGACATTGCATTTGCATTTCCATATACCTCCCTTGTCCTTGAAATTCAGGATACTCTCAAGTCAACACCCCCAGAGAACAGAACTATGAAGAAAGCGAATTTGTTGTCACTGAGCATAACAACTACCTTCTATATGCTATGTGCTTTTCTGGGTTATGCAGCTTTTGGTGAAAATGCACCTGGAAACCTGTTGACAGGATTTGGATTCTACGAGCCTTACTGGCTTGTTGATTTTGCTAATGCATGCATTGCCGTTCACCTGGTTGCAGCATACCAAGTGTATTGTCAACCAATCTTCGCTTTCGTTGAAGGATGGTTTTCTCATATTTGGCCCGACAATAAATTCATCAACAAGGGAATTCCCATGCGCATTCCTTTTTGTGGGTTGTGCAGAGTCAATCTCCTGAGGCTGTGCTGGGGAACTGCATTTGTGGTGTCAACAACTGGCATTGCCATCGTATTTCCATTATTCAATGATGTGCTGGGAATTTTGGGTGCTCTGAATTTCTGGCCATTGGTAGTGTACTTTCCAGTCGAGATGTACATTGCACGGAATAAAGTCCCAAGATGGACACTCAAGTGGAACATTTTCCAAATATTCAGCTTCATATCTCTTCTTATAACAGTGATAACGGCTACTGGCTCCTTAGAAGGCCTTGTGAAAGACAAGGAGACATAG

>pa_MA_74043g0010

ATGGATTGCAGATTCGATGAAGTGGAGTCTGGATTCAATGCATCAAGGAGAGATGGTAATAACATCTCCAATGATGTTGGCAGGCGTGGAACGTTAGTCGGGTCCCAGAGCCATAGCAATGACGATGATGGCCGTGAAAAACGAAAAGGAAATGTATGGACAGCTTCTTGTCATGTGATAACAGCTGTAATTGGGTCTGGAGTGCTATCTTTAGCGTGGAGTATGGCTCAACTTGGATGGGTGGTAGGGCCAATTGTTCTTTTAGGCTTCTCAGTTGTGACATATTATACTTCAATTTTACTTGCGGACTGTTATCGTTCACCAGACCCTGTTACTGGACAGCGTAACTATACATACAAGGATTGTGTCAAAGCCATTCTGGGTGGTAGAAGAGTATTTTTATGTGGGTTTATACAGTACTTAAATCTTCTGGGGACATCAATTGGATATACCATCACTGCTTCTATCAGCATGGTGGCTATTGGGCGATCAAACTGCTTTCATGAGAAGGGGAGGGACTCTCCTTGCCATATCTCTAATAACCTGTACATGGCCATTTTTGGGCTAGCACAGATTGTATTTTCACAGATACCAAACTTGCACAAAATGTGGTGGTTGTCAGTAGTGGCTGCGGTTATGTCAGTTTCATATGCTGGAATTGGGTTGGGGCTTGGAATTGGCAAAGCTACAGAGAAAAACCATTCTTATGGTTCTTTGAAAGGAGCTGGGATTGGAGAGCTTTTCGAGGATATAGATAAAACTTGGCATGTGTTTCAGGCCCTTGGCAATATTGCATTTGCGTATTCTTTCTCCATGATTCTCATTGAGATACAGGATACGATAAAATCACCACCGGCAGAGAACAAAACCATGAAAAGATCAACAACTATTGGGGTGGCAGTCACCACTCTCTTCTATATGTCTGTAGGTTGTGTAGGCTATGCAGCCTTTGGAACCGATGCTCCTGGCAACCTTCTCACAGGCTTTGGATTTTACAACCCATATTGGTTGGTTGACATTGGAAATGTCTGCATTATAATCCATCTGGTTGGGGCTTATCAGGTATTCGCCCAACCCTTGTATGCATTCTTTGAGGAATGGTGCTCTAATACATGGACCAAAAGCAGTTTCATACACAAGGAGTATACAGTTAAATTTCCGCGCTGTGGGTCATTGAATCTCACCCTCTTTCGTCTGGTGTGGAGGACCTGTTTTGTGATTTGTACAACATTGGTGTCCATGATTCTTCCTTTCTTCAATGACATTGTGGGCATTTTGGGAGCGCTGGCCTTCTTTCCACTCACTGTTTATTTCCCAGTAGAAATGTACATAGTTCAATACAAGGTTCCACGTTGGAGCTGTAAGTGGATGGCTCTCCGTTTAATGAGCCTAATATGCTTTGTTATATCCTTGGTTGCCTTGGTTGGCTCAGTTGCAGGTGTCATAAATGACCTGCGGAGCTACAAGCCATTCAAGACTAAGTATTAG

>pa_MA_889393g0010

ATGGATGATAAGAAGAAACACCATTATTCACATTCCAATGCCTGTGAACTCCACGACGCAGTAGAAATCTGCCGAACAGGCACGCTATGGACGGCAGTGGCTCATATAATTACATCAGTGATAGGAGCAGGAGTGTTATCCTTGTCATGGGGTGTCGCGCAGTTGGGATGGATTGCAGGGCCTGTAGTCATGATTGTCTTTGCTCTTATAACATTGTATTCCACTTTCTTGCTTGTGGATTGTTACAGGTTCCCAGATCCAGTCTCTGGGCCAATGAGAAACACTTCTTACAGGGATGCCGTACGAGTGAATCTTGGTGAAAGACACGCCCGGTTGTGTGCATTAGTTCAGTATGCGTTACTGTATGGAATATGCGTTGCGTACACAATCACTACTTCAGTCAGCATCAGAGCAATCAGCAGATCGAATTGTTACCACAGAAATGGGCATGATTCTCCATGCCATTTCCCAGATCTTACCGGATTCGGTCTGGGCCTTGCCAAAGTAATTGAAAATGGAGAGATTTATGGAACTCTGGGAGGAATCTCTACAGCCGTCTCTCTTACTCGAGCCCAGAAAGTTTGGCGGATCTTACCAGCTCTTGGTGACATTGCATTTGCATTTCCATTTTCTCCACTAGTCCTTGAAATTCAGGATACTTTGAAGTCTCCACCCGCAGAAAGCAGAACTATGAAAAAAGCAACATTGATTGCTATCATGATAACAACATCCTTCTATATGCTATGTGGTTTTCTGGGCTATGCAGCCTTTGGGGAAAATGCACCCGGTAACCTATTGACAGGATTTGGATTCTACGAGCCTTACTGGCTGATTGACTTTGCCAATGCATGCATTGTGGTTCACCTGGTGGGAGCATACCAAATGTTTTGTCAACCGATTTTCACATTCATTGAAGGTTGGATTTCTCATAAATGGCCAAATAATATGTTAATCAGTAAGAGACTCGGCGTAAACGTTCCCTTATTTGGGTTCTGTAATGTCAATCTATTCAAGCTGTGTTGGAGAACTGCATTTGTGGTGTCAACTACTGGCATTGCCATTTTATTTCCATTATTCAATGACGTGGTGGGAATTTTGGGTGCTCTGAACTTTTGGCCATTGGTGGTGTACTTTCCCGTGCAGATGTACATTGTACAGAAAAGAGTCCAACTCTGGACACTCAGGTGGAACCTTTTGCAAACATTGAGCTTCATATCTCTTCTTGTGTCAGTGGGAACGGCTGTTGGCTCCATAGAAGGCCTTGTCAAAGACAACGAAACATAG

>pa_MA_902657g0010

ATGGACGATAACGAGAAACACCATCGTTCACATCCCCTTGCCTGTGAACTCGACGACACAGTAGAAATCTCCCGAACAGGCACGGTATGGACAGCAGTGGCTCACTTAATTACATCAGTGATAGGGGCAGGAGTGTTATCCTTGTCATGGAGTGTTGCTCAGTTGGGATGGATTGCAGGGCCTGCAGCAATGATTGTCTTTGCTCTTGTATCACTGTATACCACTTTCTTGCTTGTGGATTGTTACAGGTTCCCGGATCCAGTCTCTGGGCCCATGAGAAACACTAGTTACAGGAAAACAGTCCGAGTGAATCTAGGTGAAAGAAAAGCATGGTTGTGTGCATTAGTTCAAAATGCGTTCTTCTATGGAGTCTGCGTTGCATACACAATCACTACTTCAGTCAGCATAAGAGCAATCAGCAGATCGAACTGTTACCACAAAAATGGACATGACTCTCCATGCCATTTTCGTAATATTACCTATATGATCATCTATGGAGTTATACAAGTAATCTTGAGTCAAATACCAAGCTTTCATAAGATATGGGGCCTTTCAATACTGGCAGCGATAATGTCCTTTACGTATTCAACGTTAGGCTTCGGTCTGGGCCTGGCCAAAGTAATTGAAAATGGAAAAATATACGGAACTCTGGGTGGAATCTCTACAACTGTTTCTCTTACTCGAGCCCAGAAATTTTGGCGGATTTTACCAGCTCTTGGTGACATTGCATTTGCATTTCCATTTACTCCACTTGTGATTGAAATTCAGGATACCTTGAAGTCTCCACCCCCAGAAAACAAAGCTATGAGGAAAGCATCACTGGTTTCTATGATGATAACAGCATCCTTCTATATGCTATGTGGTTTTCTGGGCTATGCAGCGTTTGGGGAAAATGCACCTGGGAACCTATTGACAGGATTTGGATTCTATGAACCTTACTGGCTCATTGACTTTGCCAATGCATGCCTTGCGGTTCACCTGGTGGCAGCATATCAAGTGTTTTGTCAACCGATTTTCTCTCTCGTTGAAGGTTGGATTTCTCGTAAATGGCCCAGCAATACGTTAATCAGTAAGAGAATTAGCATACGCGTTCCCTTATTTGGGTTCTATAAAGTCAATCTGCTGACGCTGTGTTGGAGAACTGCATTTGTTGTGTCAACTACTGGCATTGCCATCTTATTTCCATTGTTCAATGATGTGCTGGGAGTTTTGGGTGCTCTGAGCTTTTGGCCATTGGTAGTGTACTTTCCAGTGGAGATGTACATTGTGCAGAAAAAAGTCCAACGATGGACACTCAAGTGGAGCCTTTTGCAGACATTGAGTTTCATAGCCCTTCTTATATCACTGGTAACTGCTGCTGGCTCCATAGAAGGCCTTGTTAAAGACAAGAAATCATAG

>scaffold00001.289

ATGGCCGAGCTTGAGGACGTCTCGGCACCCATTACCCCACGGTCCACTACCGGAGCCACCACCACGACACCGCCGACTCC

CGCCCCACGCTCGCCATGGAACGTCCCCATCACCCCTCGCTCCATGGCTGAACCCCCAACACCTGGCGGCCTCCGCTCGC

CGATCCCCCACTCCACGATGATGATACGTGAAGTCGTCTCTGTCCCTGTAACTCCGAGATCAACAGCCTCTGTCACGCCT

CCTGTCCCTCACTCCCCTTTACCCCCTCTCCCTCGACGCCCTCCTCCCGTGATTTCAGCTCCGCCCTCCAATCTCCACTC

TCCGTCCCTCACTCGATCGCCTCTCCTGCATACACCGACGACCACAGGCAACACGCCACGGACACGGTTCTCGACGCCGT

TTGCGAGCCCCATGAGGAGGGCAATTGTGAACATGCGGTCGTATTTGGAAGATGTTGGACACCTCACGAAGCTGGATCCG

ATGGATGCTTGGCTGCCCATCACCGAGTCGAGGAGTGGGAATGCCTACTACGCTGCGTTTCATAATCTGACGGCTGGGAT

TGGGTTTCAGGCTCTTGTGTTGCCTGTTGCATTCACCTTTCTTGGATGGACATGGGGGATCATCTGTTTGTCCATAGCCT

ACTTCTGGCAGCTCTACACCCTCTGGATCCTCACTCGCCTCCACGAATCTGTCCCCGGTACCCGCTACAGCCGCTACATC

CAGCTCGCTAAAGCCGCTTTCGGGAAGAAATTGGGAGCATGGCTCTGTAAGATCCCCATCATGTACCTATCGGCAGGCAC

ATGCACGGCATTGACAATTGTCGGGGGCAGTAGCATGAAACTCTTCTTCGAGATCGTGTGCGGATCATCGTGTCACTCTG

ATCCCCTGACCGCAGTGGAGTGGTATCTGGTGTTCACATGTTTGGCTGTAGTCCTCTCTCAATTGCCCAATCTCAACTCC

ATTGCTGGTGTCTCACTGGTTGGGGCAATCACTGCTGTGGCCTACTGCACTCTCATTTGGGTGCTCTCGGTGGTCCGGCC

GCGACCACCAGGTGTGTCCTATGATCCCATCAGGGGGAAATCGGATCCGGCCACTGCTTTCTCCATTTTAAACGCTTTGG

GTATCATTGCTTTTGCATTTAGAGGCCACAACCTGGTTCTTGAGATTCAGGCAACGATGCCGTCGACACTGAAGCACCCA

GCTCACGTGCCAATGTGGAGAGGAGTTAAAGTGGGGTACGTGCTCGTAGCTGCCTGCTTGTTCCCTCTTGCAATAGGGGG

TTTTTGGTCCTATGGCCACTTGATACCTGCCGGGGGGATGGTCAGCGCTCTGTATGGCTTCCATATGAAAGACATTCCAA

GGGGCTTGCTGGGGCTGACAAGCTTGCTAGTGGTGATAAACTGCCTAAGCTCCTTCCAAATCTATGCAATGCCTGTCTTC

GATGACTTTGAGCACGACATAACAAAGAAGAGCAACCGCCCATGCCCTCGTTGGCTGCGCTCAGGGTTCCGAGCATTCTT

TGGGTTTATAGCCTTCTTCATTGGGGTGGCCTTCCCCTTCTTGTCAAGCCTTGCAGGGCTTCTAGGAGGGGTCTCTTTAC

CAATAACATTGGCTTACCCCTGTTTCATGTGGATCTTCATCAAGAAGCCTGAGAAATATAGTGCAAATTGGAATATCAAC

ATGGGTTTGGGTTGCCTGGGCATAATCATGAGCACTTTGGTTGTGATTGGAGGACTTTGGAGTACCATTGACTCTGGTCT

GAAGCTACGGTTCTTCAAGCCTGAGTGA

>scaffold00002.493

ATGGAGAGTGGAGAACAAGAAGAAGGGTGGATAGAGAATAGGAGGAGCAAAGGGACATGGAAACATGCAGCTTTCCATGT

TGCCACCACCATTGCCACCCCTGCTGCTTACGCACCTTTGCCCTTTGCCCTCGCTTCTTTAGGCTGGTCTCTTGGGGTTT

TTAGCCTGGTTGGTGGGACATTGGTGACCTGGTATTCCAGCCTCATCATCTCATCTTTGTGGGCATGGAATGGCGAAGAG

CACACCACTTATCGACAGCTTTCCCGTAGTATCTTTGGGTCTTGGGGTTATTGGGCCGTCTCATTTTTCCAACAAGTGGC

TTCTCTGGGAAACAATATAGCTATCCATATTGCAGCGGGCACAAGCTTAAAGGCAGTTTACAAGTACTTTCATGAGAATG

GTGCCCTCACTTTGCAGGAGTTCATCATATTTTTTGGGGTTTTTGAGCTGTTTCTATCACAGTTTCCGGATATCCATTCC

CTTCGTTGGGTTAATGCTTTATGCACTTGCAGCACTGTCGGATTTGCTGGTACCACTATTGGTCTGACTCTCTACAATGG

GAAAAAGATTGAGAGGGGATCGGTGAACTATCATTTAGAAGGAGACTTAGCCACTAAGATATTCAAGGCATTCAATGCCC

TGGGAACCATTGCTTTCTCATTTGGAGATGCAATGCTTCCTGAAATACAGAGTACTATAAGGAACCCTGCAAAGAAAAAT

ATGTATAAAGGCATATCTCTCGCATATGGAATTATTGTCCTGAGCTACTGGCAAGTAGCTTTCAGTGGATACTGGGCATT

TGGGTCTCAAGTTCAGCCCTATATTTTGTCATCCTTGAAAGTTCCACAATGGGCCATTGTAATGGCCCACATGTTTGCTG

TAATCCAAATAGCAGGGTGTTTCCAGATATATTGCCGGCCAACTTATGCATACTTCGAGGAAAACATGTTATCTAAAGAT

CAGACTGGCCTATTCAGAATTCGCAATCGACTAGTTCGCCTCTTTGCAACCTCTCTCTACATGGTTCTAATTACTTGTAT

TGCTGCTGCAATGCCCTTTTTTGGGGACTTTGTTGCAATTTGTGGGGCAGTGGGTTTCACACCTCTGGATTTTGTAATGC

CCCTAATTGCTTATGTGAAAGTAGGGAAACTACCAAAGAACAGAAGTTTGGGTCTCTCAGTTAAGGCCCTTAACCTTTTT

ATTGCTGTTTGGTTCTCTGGGGTAGCCATTCTGGGTTGCATAGGTGCTGTGAGATTCACTGTTAAAGACGCACAAACTTA

CAAGTTCTTTTATGACATGTAA

>scaffold00005.76

ATGGGTGGTGCATTTGATGGACAGGAGAACGATGTATCCTTGGAGAAGCAGAAGGCCGTGGACGATTGGCTTCCGATCAC

CTCGTCTCGCAATGCGAAGTGGTGGTACTCTGCCTTCCACAATGTCACAGCCATGGTCGGCGCTGGAGTCCTCAGCTTGC

CCTTTGCCATGGCCGAGCTTGGATGGGGTCCTGGTGTTGCAATTCTCGTCATCTCATGGATTATAACACTATACACCCTC

TGGCAAATGGTTGAGATGCATGAGATGGTCCCCGGAAAGAGGTTTGATCGATACCATGAATTGGGGCAACATGCTTTTGG

AGAAAAGCTCGGCCTCTGGATTGTGGTGCCACAACAACTGATTGTGGAAGTCGGGGTCGACATAGTTTACATGGTCACAG

GGGGAAAATCCCTGAAAAAATTCCATGATACAGTGTGCCCTGGATGCAAACCTATAAAGCTCACCTATTTCATCCTCATC

TTTTCCTCTGTTCACTTCGTTCTCGCTCAGTTACCTAACTTCAATTCCATCTCTGGGGTCTCTTTGGCTGCAGCAGTCAT

GTCTCTAAGTTACTCAACAATAGCTTGGGTTGGATCCCTTGATAAAGGGAAGCAACCAGATGTAGACTATAGTTACAGGG

CTTCATCAACTTCTTCTGCAGTATTTAACTTCTTCAGTGCACTTGGAGAGGTCGCCTTCGCGTATGCAGGTCACAATGTG

GTTTTAGAGATTCAAGCAACAATCCCTTCATCACCTGAGAAGCCATCAAAGAAACCCATGTGGAAGGGTTGTGTCGTTGC

TTACATTGTTGTGGCCCTTTGCTATTTCCCTGTCTCTCTAATTGGTTATTGGGCCTTTGGAAATAAAGTCGAAGAAAACA

TTCTCCTTAGCTTGAATAAACCACGGTGGCTAATTGCAGCTGCTAACATGTTTGTGGTTGTCCATGTTATTGGAAGCTAC

CAGATTTATGCAATGCCTGTGTTTGACATGCTTGAAACTGTGCTGGTAAAAAAGCTTAAGTATACTCCCAGCATAGTGCT

ACGGTTAATTACTCGCAGCAGTTATGTTGCATTCACATGCTTTGTTGCTATAAGCCTTCCTTTCTTTGGTGGACTTCTTG

GGTTCTTTGGCGGATTTGCTTTTGCCCCAACAACATATTTTCTTCCATGCGTAATGTGGCTTGCTGTCTACAAGCCGAAA

AGGTTCAGCTTCTCTTGGATAGCAAACTGGTTCTGCATCATCCTTGGTGTATTACTCATGATCGTTGCTCCTATTGGTGC

ACTAAGGCAGATTATCCTTAACGCTAAGGGCTACAACTTCTACTCATGA

>scaffold00015.85

ATGGAAGAAGCACAGGAAAATGGCAGGCATCAATTTCCTCAGAATCCCTGTGAGAACGATGCTGGAGCTGTCTTTGTCCT

TGAATCCAAAGGGAAATGGTGGCACGCGGGATTTCATCTTGTGACTGCGATCGTAGGGCCGACAATACTGACGCTGCCGT

ATGCAATGAGTGGCATGGGTTGGAGTTTGGGGCTTCTCTCTCTTACTGCCGTATTTGCAGTCACATTCTATTCATATTCT

CTCATGTCAAAGGTGTTGGAGCACTGCGAGAAAGAGGGCCGAAGGCACATACGCTTCAGAGAGCTTGCGTCTCGTATCCT

CGGATCGGGATGGATGTTTTACTTCGTGATTTGCATTCAAACTGCCATCAACACGGGAGTTGGAGTTGGGTGCATTTTGC

TTGCAGGCCAATGTCTTCAGATAATGTACAAAGACGTTTATCCTGATGGTCCACTCAAGTTGTACCATTTCATTATGATG

GCAACTGTAGTCATGGTTTTGTTATCGCAATTGCCCTCCTTCCACTCCCTTAGACATTTGAACCTCGCTTCTCTCTTCCT

CAGCTTGGGCTACACCTTGCTCCTCGTCATTTCCTCTATTGTTGCAGGGTACTCCAAAAATGCCCCTCCTAAGGATTACT

CACTTGAAACTTCAACATCTGTCCGCACATTTGATTCTTTCAGTTCGATTTCAATCCTAGCCTCTGTTTTCGGTAATGGA

ATCCTCCCTGAGATTCAGGCAACTCTTGCTCCTCCAGTAACTGGAAAAATGCTTAAAGCGTTGTCCTTGTGTTATATAGT

CATATTCTTCACCTTCTACGCGGCTGCGATCTCAGGGTATTGGGCATTTGGAAACAAGGTGAACTCCAACATTTTGAAAA

GCTTGATGCCTGACAATGGGCCATCTTATGTCCCAACCTGGATGCTCGGTATCGCTATAATTTTTGTTATCTTGCAGCTC

TTTGCAATTGGCATGGTGTATTCTCAAGTCGCATACGAGATAATGGAAAAGAAATCAGCGGACATAAGGCAAGGAGTGTT

CTCCAAGAGAAATTTGGTCCCTCGGATCTTCCTCAGGACATCATACATGATCTTTTGTGCTTTTCTTGCATCAATGCTCC

CCTTCTTTGGTGACATAAGCGCAGTGGTGGGTGCTGTTGGGTTCATCCCACTTGATTTCATCCTCCCCATGCTTTTATAC

AACATGAGTCTCAAGCCCCCACAAAAGTCATTCACATACATCATCAACATATCTATCATCATTGTATTCTCAGGCGTCGG

AATCATGGGTGCTTTCTCATCAATTCGGAAGTTGGTCCTTGATGCTCGAAAGTTCAAGCTTTTCAGCGATGATGTTGTTG

GGTAA

>scaffold00017.258

ATGGGGGAGAACGGGGTGAGGAAGCAGTATTTGCAGGTGGAGAGCCAGACCAGAGTGTATGGGGACATGGCCATCGATAT

GCAGCATCAGGGGTCGAAGTGCTACGACGACGATGGGCGTCTCAAGAGGACCGGCACGGTCTGGACCGCGAGCGCCCACA

TAATCACCGCCGTGATCGGCTCCGGCGTTCTCTCTCTAGCCTTTGCCATTGGGCAGCTCGGCTGGGTCGCCGGCCCGACC

GTGATGGTCCTCTTCTCCTTTGTGACCTACTACACCTCCACCTTGCTCTCCGACTGTTACAGGTCCGGAGACCCGCTCAC

AGGAAAACGAAACTACACTTACGTGGATGCTGTCAGAGCCAACATGAGTGAGTTTAATGTCCGATTGTGTGGATGGCTTC

AGTACGCCGGACTCGTTGGAGTGGCAATTGGCTACACCATTGCTTCGTCTATAAGTATGATGGCGATAAAGAGGTCGAAC

TGTTTCCATGAAACTCGAGGCAAGAATCCATGCCACGTATCGAGCAACCCTTACATGATAATGTTTGGCATCACCGAAAT

CGTGCTCTCTCAGATCCCGGACTTCGATCAGATCTGGTGGCTCTCCTTTCTCGCTGCCGTCATGTCCTTCACCTACTCCT

CCATCGGCCTCGGCATGGGCATAGGCAAAGTTGCAGTGAACGGGACAATCAAAGGGAGCATGACAGGGATCAGCATCGGC

GCTATGACCCACGCAGGCCCGATCACGGCGATGCAGAAAGTGTGGCGCACCTTCCAAGCCATGGGCGACATAGCCTTCGC

TTACTCTTACTCCATAGTCCTTATAGAGATTCAAGACAGCCTGAAATCTCCGCCGTCTGAGGCCAAGACCATGAGAAAGG

CCACCATGATCAGCGTCACCGTGACCACCATATTCTACATGCTCTGTGGGTGCATGGGATACGCAGCCTTCGGTGACCAG

GCCCCCGGCAATTTGCTCACCGGCTTCGGCTTCTACAACCCATACTGGCTCCTCGACATCGCGAACGCCGCCATCGTGGT

CCACTTGGTGGGTGCTTACCAGGTCTATTGCCAGCCCCTGTTCGCCTTCGTCGAGAAATACGCCGTGCGGAAGTGGCCGA

AGAGTTGGTTCATTAACCACGAGTTCGAGCTCAGGGTCCCATTCTACAGCAAATCCTTCAGCCTGAATCTCTTTAGGTTG

GTGTGGAGGACGTCCTTTGTGTGCATGACCACTCTCATTGCCATGCTCTTCCCTTTCTTTAATGGCGTGGTGGGGATCCT

TGGGGCTTTTGGGTTTTGGCCATTGACTGTGTATTTCCCTGTGGAGATGTATATTTCACATATGCACATACCCAAGTGGT

CCACTAAGTGGGTTTGCCTTCGGGCCCTGAGCTTGGCTTGTCTCGTTATCTCCATGCTTGCAGCTACTGGATCAGTTGCA

GGCATGATTTTTGAGATGAAGGCAGCTTACAGACCCTTCCATACAAACTACTGA

>scaffold00029.259

ATGGTCCGGCCCTTTGATAGCAACATACTTCTCACAAATACTAAAACCCTTAGAATCGATTCATTGAGAGAGGACCGAGT

GGACATTGCTTCTTGCGAGTCGTCTTCCTTACTGGCCGATTCCATGGCTGGTCTTGAAGAGAGTCCAAAAAAGCCCATTG

GCGATGAATTTGACCTCAAAGTTCAAGAAGAGACGGCTCACCAGATCAGCCATGATCCTTGGTATCAAGTTGCGTTTGTT

CTTACAACTGGGGTTAACAGTGCATACGTTTTGGGATATTCTGGCACAATTATGGTTTATTTAGGTTGGTTTGCTGGCAC

AATTGGGTTAATTGCCGCCGCTGCCATATCGCTATATGCCAATTCTCTTCTTGCCAAGCTTCATGAAGTTGGGGGAAAAA

GGCACATTAGATATAGGGACCTCGCAGGCCATATTTATGGTAGGAAGATGTATTCTCTTACATGGGCCTTGCAATATGTA

AACCTCTTTATGATTAACACTGGATACATCATATTGGCTGGGCAAGCTTTGAAGGCGATATATGTTTTATTCAGGGATGA

TCATGCTTTAAAGCTTCCATACTGTATTATAATTGCTGGAGTTGTATGTGCTATGTTTGCCTTTGCAACTCCTCATTTAT

CTGCTTTGCGGGTGTGGCTTGGATTTTCAACATTCTTCGGCCTTGTATACATTGTAATAGGTTTTGTGTTGTCTCTTAAA

GATGGAATAAGGAGCCCTTCAAGGGATTACAGCATTCCAGGAACTTCAACAAGCAAAGTATTCACCTCCATTGGTGCCAC

AGCGAGTCTTGTTTTTGCTTACAATACAGGGATGCTACCTGAGATACAGGCAACTGTGAAGCCACCAGTTGTAAAGAATA

TGCAGAAAGCCCTCGCCTTTCAGTTCACAGCTGGTGTGCTCCCACTATATGCAGTTACATTTGTTGGGTATTGGGCGTAT

GGATCCTCAACTTCATCCTATCTCCTTAACAGCGTGACTGGCCCTGTTTGGGTGAAAATGGTGGCAAATGTGGCCGCTTT

CTTGCAAACAGTCATTGCTTTGCATATATTTGCAAGCCCAATGTACGAGTACTTGGATACAACATATGGTATGTCTAGAC

AGGGAAAGACATACTCTATGCAAAGCATCTTATTCCGGGTTGTAGTGAGAGGTGGCTATCTCACTGTAAGCGCCTTTGTC

GCTGCTCTCCTACCGTTTCTTGGCGATTTTATGAGCCTGACAGGTGCACTAAGCACCTTCCCTCTAACCTTTGTGCTCGC

CAACCACATGTATTTGATGGTGAAGAGGGAGAAACTCAGTTCTCTGCAAAAGGCATGGCATTGGCTTAATGTGTGTGGGT

TCAGTTTGTTGGCTGCAGCTGCGGCCGTTGCCGCAGTGAGGCTCATTGTTGTGGACTCGAGAACGTATCATGTATTTGCT

GATATCTGA

>scaffold00033.53

ATGCATGAGCACGATAATGGAATTGCGTTTCAGGGCACCGCAGAAAGGAGGAGCCTCGAGATAGGGAAAGCTTCTATTGG

GTTTGACGACGATGGCGTCGAAAAAAGAACCGGGACGTTCTGGACGGCAAGTGCTCATATCATAACAGCGGTTATAGGGT

CAGGGGTGCTTTCCCTTGCATGGGGGATGGCTCAGTTGGGGTGGATCGCAGGGGCGTTTGTTCTGCTTTTGTTTTCCGGA

ATCACATACTATACATCTTGTTTACTGGCGGACTGTTATAGATCCCCTGACCCAGTCCATGGAAAGAGGAACTATACCTA

TACAGACGCCGTAAAAGCCAATTTAGATGAAAGAAGAACCAAATTGTGTGGGATTGCACAATATATATACCTTTTCGCAA

CCTGCCTGGGTTACACTCTCACTGCAGCAATCAGCATGGCGGCCATAGAGAGATCGAATTGTTTTCACAAGCATGGCCAC

GACGCACACTGCCCACCGAAAACCAACAGGTTCCTCATAATCTTCGGCTGCATTCAATTGTTTCTCTCTCAAATTCCGAA

TCTTCACAAGCTTTCATGGCTCTCTGTAATCGCTGCGGTTATGTCTTTCACTTACTCCACCATAGGCGTGGGGCTCTCCA

TCGCCAGAGTCTCAAGCAAAGGAGCTTCGCATAACACGTCTCTTACGGGAGTGGCAGTTGGCGTGGACGTGACATCGACT

GAGAAGATTTGGAAGGTTTGCCAGGCCCTCGGAGACGTTGCCTTCGCTTATGCTTTCACCTCAATCCTTATTGAAATTCA

GAACACATTGAAAAGCTCCCCACCTGAGAACAAAGTGATGAAGAAGGCGAGCTTTTTGGGAATTTTGGTGACCACCATGT

ACTACATGCTCTGTGGTTTTATAGGCTATGCAGCTTTTGGCAATAAGGCTCCCGGAAATTTCCTCACCGGATTCGGATTC

TATGAACCCTTTTGGTTGATTGATTTAGCCAACGTTTGCATTGCCATTCATCTCTTTGGATCATATCAGGTCTTTGGACA

ACCGATGTTTGCATACTTGGAGAGGGGTTGCTCGAGGCGGTGGCCATCCAGTAGCTTCGTGAACAACGAGTTGAGAACCA

AAATTCTTGGTTTTCCCTTCCAATTTAACATGCTCCGCTTGGTTGCGAGGTCATTGACTGTGATAATTATGACCATCATC

ACCATGATATTCCCCTTCTTTAACGACGTGGTGGGGTTAGCAGGGTCTATGTCATTCTGGCCACTTGCAGTCTACTTCCC

AACCGAGATGTATATTCGCAAAAGGAATTTAAATAAAGGCTCCAGCGAGTGGTGGTGGCTGCGCCTGCTGAGCCTACTTT

GCCTGTTTGTCTCTATAGCAGCCGCAGTCGGCTCGGTTCAGGGCTTGATTACCTCTCTCAGGTCTTACAAACTCTTCAAG

TTCGAGTAA

>scaffold00033.54

ATGCAGAAAGAGGAAGGAGTCCAGTGCCTCGATGCCGGAACGCCCTTTGACCAAACAGACGACGATGGGCGAGTCAAGAG

AACAGGGACATTGCTAACAGCGAGTGCACATATCGTTACTGCTGTGATAGGATCAGGGGTGCTTTCTCTCTCATGGGCAA

TAGCTCAGTTGGGGTGGATTTTTGGGGTTTTCGTGCTCCTGATTTTCTCTCTCATCATTTTATACACCTCGTTTTTTCTG

GCGGATTGTTATAGATATCCAGATCCTGTTAATGGCAAGAGAAACTACAATTACAAAGCTGCCGTGAGTGCCCATTTAGG

CGGGCTCAAAACCAAAATATGTGCGTCCACACAGTATGTCTTTCTCGTCGGAAACTGTTTAGGTTACGCAGTAACTGCAT

CGCTGAGCATGGCGGCGGTGGAGAGATTGAATTGCTTCCATAAGAATGGGCATGCAGCTGAATGCGTCGCTTCGACGAAC

AAGTACCTGGTCATCTATGGATGTATTCAGATTGTGCTCTCTCAAATCCCCAATTTTCACAAGCTTTGGGGCCTCTCTAT

TGTTGCAGCTATCATGTCCTTCTGTTACTCCTCTATTGGTGTTGGGCTTTCCATTGCCAAAATTGCAGGTGAAGGAGCTT

CGAAAACAACGTGTCTTACAGGGGTAAGAGTTGGGATAGATATCACTGCGGGAGAGAAGTTTTGGAGGGTTTGCCAAGCT

CTCGGAAATATTGCATTTGCCTCCGCATTTACTGCTGTGCTCTTAGAAATCCAGGATACATTGAAAGGCCCTCCTCCTGC

TGAGAACAAAGTGATGAAGAAGGCAACTACAATATCAACCATCATCACTACTGTCTTCTACTTGCTTTGCGGTTGCTTGG

GCTATGCAGCATTTGGCAGCAAGGCACCAGGAAATATGCTCACTGGGTTCGGGTATTATGAACCCTTTTGGTTGATAGAT

TTTGCCAATGTCTGTGTTGCTGTTCATCTCTTTGGATCATTTCAAGTGTTTGCCCAGCCCCTATATGCAGTCATGGAGAG

GGTGTGCTCGAGGCGATGGCCTTCTAACCCTTTCATCAGCAATGAATGGACAATTTCAACCTTGGGATTATGCTCCTACT

CATTCAACTTTTTCAGGATCACATCAAGAACGTTGTATGTGGTGGTCCTCACCCTCATCGCCATGATCTTCCCCTTCTTC

AACGACTTGGTGGGGTTGATTGGGTCCATTGCCTTTTGGCCCCTTACAGTTTACTTCCCTGTAGAGATGTACATCCACCG

AAAGAAGGTCCAGCGCGCGTCTACCGAGTGGTGCTGGCTGCAGATACTCAACTTGATTTGCCTCTTGGTCAGTGTTGCCG

CGGCCGTGGGCTCCTTTCAAGGCCTTGCCACCTCTTTGAGGACTTACAAGCCTTTCAAGACCTTCTAA

>scaffold00040.261

ATGACTACCAATGAAGAAAAAGTGAAGGACCTTGACGCAGGGGCACTTTTTGTTCTCAAATCAGAAGGTTCATGGATTCA

TTGTGGGTACCACCTAACGACCTCCATTGTTGCCCCTGCTCTATTGAGCCTGCCTTTTGCACTTGCTTCTCTTGGTTGGG

TGCTCGGGCTTTTGTGCTTGACAATTGGGGCTTTGGTCACTTTTTATTCATACAACCTTCTCTCTCTAGTGCTAGAGCAC

CATGCACAGCAAGGCCAGCGCCACCTAAGGTTCAGGGACATGGCCCATCATATTCTAGGACCTAGATGGGCTCGATATTT

TGTGGGTCCAATTCAATTTGCGGTTTGTTATGGTGCGGTCGTTGGTTGTACCTTGCTCGGAGGCCAAAGCATGAAGTCCA

TATACTTAATCAACCATCCAAACGGGGGAATGAAGCTATTCGAATTCGTAATGATATTTGGATGCCTCATGTTGGTGTTG

GCTCAAGTTCCCTCCTTCCATGCCTTGAGACACATAAATTTGGTTTCCCTAATTCTCGCTCTTGGTTATAGTGCATGTGC

CACTGCTGGATCCATCCATGTTGGAAATTCGAGTGATGCTCATAAAGACTATTCTCTCAATGGAGACACTGCAAGTCGCA

TGTTTGCAGGATTCAATGCCATGGCCATCATAGCAACCACCTATGGGAATGGTATCATCCCAGAAATACAGGCAACATTG

GCACCACCAGTGAAAGGGAAAATGTTTAAGGGACTATTAGTTTGTTACAGTGTGGTCTTTGTTACATTCTTTAGTGTTGC

CATTTCTGGGTATTGGGCATTTGGAAACAAGGCAGGGGGTACAATCCTGGCCAATTTCTTTGTTGATGGAAAAGCCCTTG

TCCCCAAGTGGCTCCTCGTCCTCTCTAACCTTTTTGCACTCCTTCAAGTATCAGCTGTTGGTGTGGTGTACTTGCAACCC

ACGAATGAAGTTTTGGAAAGAAAATTCGGCGATGCTATGAGCGCCGAATTCTCGCCCCGAAATGTTATTCCCAGGTTAAT

ATTGCGGTCAATTTCGGTGGCATTGGCCACCTTGTTGGCCGCAATGCTACCCTTCTTCGGAGATATCAACGCATTGATTG

GGGCTTTTGGATTTATCCCCTTGGACTTTGTTTTGCCGGTTGTTTTCTATAATACCACATTTGCCCCATCAAAGAAGAGC

TTCCTCTTTTGGGGAAATACCACAATTGCCCTTGTTTTCTCAGCCGTTGGATTGATCGGGGCAGTGTCTGCAGTTCGTCA

GATCTATTTGGATGCCGATAACTATAGATTGTTTGCTAATGTGTAA

>scaffold00059.251

ATGGAAGAAAGGAGCTCTGAACCAGAGCTGGTCTCTATACCTGCAACTCCAAAGGTCTCCACCCCACCTTTGACACCGGA

CATTCTCTCTAGCTCACATTCCCATGCTGTAACTCCCTCCGGTCAGAGATCACCGAGGCCCCTGTCTTCTTCTCTTATTT

CTCTCTCTAACCCTGCATCTCCTCCCCCGCCATTAAAGACCCCAAGAACTCCATGGACCCCCTCTTCTCTTATTTCTCCA

AGGTTTTTGAGCCCAATAGGAACCCCAATGAAGAGAGTTTTGGTGAATATGAAGGCCTATTTAGAAGACATTGGGCATTT

GACTAAGCTAAATCCACAAGATGCATGGCTCCCCATTACCGAGTCGAGAAACGGGAACGCATACTATGCTGCATTTCACA

ATCTCAATGCCGGGATCGGGTTTCAGGCTCTCATTCTTCCTGTGGCTTTCACTTTTCTTGGATGGAGTTGGGGGATTATC

TCTTTGGTAATAGCATATTGTTGGCAACTCTACACACTCTGGGTTTTAGTGAGGTTACATGAAGCTGTTCCTGGGAAGAG

ATACAACAGATATGTGGAGCTTGCACAAGCAGCATTTGGCGAAAAGCTTGGAGTATGGTTAGCTCTCTTCCCAACAGTGT

ACCTCTCAGCAGGAACTGCAACTGCTCTTATCTTAATTGGTGGGGAGACCATGAAGCTCTTCTTCCAGATAGTGTGTGGA

CCCAACTGTGAATCTAACCCTCTCTCCACTATTGAATGGTACCTTGTCTTCACCACCCTTTGTATTGTTCTCTCTCAATT

GCCAAACCTCAACTCCATTGCAGGGCTCTCTCTAGTAGGGGCCATCACAGCCATCACATACTCCACCATGTCGTGGGTCC

TCTCTGTTAGCCAGCCTAGACCACCTTCCATCTCTTACCAACCCCTCAGATCTCCTTCCTTCTCAGTGACCGCTCTCTCT

CTCTTGAATTCTCTTGGAATCATTGCTTTCACGTTTAGAGGACACAATCTGGCACTAGAGATTCAGGCAACAATGCCCTC

AACCCTCAAGCACCCAGCTCATACACCAATGTGGAAAGGTGCTAAAGTTGCATATTTGTTGATTGCCATGTGCTTGTTTC

CCATAGCAATTGGGGGCTTTTGGGCATATGGAAACTCGATGCCTGCAGGGGGGATGCTGAACGCCTTGTATGGGTTCCAC

AGCCATGACATCCCCAGGGGCCTCCTTGCAGTGACATTCCTCTTAGTGGTATTCAATTGCCTGAGCAGCTTCCAGATATA

CTCAATGCCAGTTTTTGACAGCTTCGAGGCCGGTTACACAAGTCGCACAAATAGACCATGCTCAATATGGGTTCGTTCTG

GTTTCCGAATTTTCTATGGCTTCATCTCTTTCTTTATTGGCATAGCCCTTCCTTTCCTCTCGAGCCTAGCTGGGCTCTTA

GGAGGGCTCACTCTACCAGTTACATTTGCCTATCCTTGCTTTATGTGGGTTCTCATTAAGAGGCCAACAAGATTCAGTTT

TAGCTGGTATCTCAACTGGACCCTTGGAATATTGGGGATTGCTTTTAGCATGGCTCTCTCAGCTGGGGGTGTCTGGAGCA

TGGTGAATAGTGGATTGAAGCTCAAATTCTTCAAGCCAAGCTGA

>scaffold00061.89

ATGGAGATGACAGACAAACAGGAACCTCTTATCGCTGATGCCCAAGTACGGTGCAAGAGAACAGGGGATGCATGGACTGC

AACTGCTCATATAATCACTGCAGTGGTTGGTTCAGGGGTTCTGTCTCTAGCATGGTGCGTAGCACAGCTGGGTTGGATTG

CAGGGCCTTTGGCAATCCTGGGTTTTGCAGTAATCACACTCATTTCTTCCTATTTGCTAGCTGATTGTTGCAGGTCCCCT

AATTCTGAGAAGGGGCACATTAGGCATATCACTTACATCGATGCCATAGATTTTAATCTAGGTAAGAAGAGCGTGTGGAT

ATGTGGGTTGGTTCAACAATTAGGGATTTGTGGCACTGCGATTGCTTACACAATTACTTCAGCTATGTGCTTGAGAGCTA

TTCAGAGATCTAATTGCTTTCACATAGAAGGGCATGAAGCTGCATGTAAATATGGAGATACAGCATATATGTTAACATTT

GGGGTGGTACAGTTGGTATGCTCTCAGATACCTGATTTCCACAACATGGGATGGCTCTCCATTGTTGCAGCAGCTATGTC

TTTCTCCTATTCATCTATTGGATTCGGCCTTGGCCTTGCCAAAACAATTGAAAATGGAAAAATATATGGAAATATAACAG

GAGTGGCAATGTCATCAACGGTTGCGAAATTGTCAAGGGTGTGCCAGGCTCTAGGAGACATTGCTTTCGCATATCCATAT

TCCATTGTTCTCATCGAGATACAGGATACACTCAAGGCACCTCCACCAGAGAACCACACAATGAAGAGAGCTTCAATGAC

AGCCATTGTGATTACTACTTTCTTCTATCTGTGTTGTGGATGCTTTGGCTATGCCTCCTTTGGAGACTCAACACCTGGAA

ATCTCTTGACTGGGTTCGGTTTTTATGAGCCATATTGGCTCATAGACTTTGCAAATGCCTGCATTGTGCTTCATTTGGTG

GGGGGCTATCAGGTGTACAGTCAGCCCCTTTTTGCATTTGTAGAGAAATGGTTAATCCAAACATTCCCAACTTGTGCACT

AATGAAAACAACATACACACTTCACATCCCATTCATTCCTACTTTCCAGTTAAATTTACTTAGGCTTTGCTTTAGGACTG

CATATGTTGTCTCAACCACTGGCTTGGCAATGTTTTTTCCTTACTTCAATCAAGTGCTGGGTGTACTTGGGGCTCTTAAT

TTCTGGCCAATTGCTGTTTATTTCCCTATTCAAATGTACATTGTGCAAAAGAATGTGCAGACCTGGACAAGCAAGTGGCT

TATTTTTCAGACCTTCAGCTGTATTTGTTTGGTTGCATCTGTTATAGCCCTAACTGGATCCATTCAAGGGATTGTAAGTG

AGAAAACAAGTTAA

>scaffold00071.161

ATGATACCTACATACAATGCTTATAGCTATGGAACAGTATGGACGGCAGCAGCCCATGTGATTACAGCAATAATTGGGAC

TGGGGTCCTGGGGTTGGCCTGGAGTGTTTCTCAACTTGGTTGGATTGTTGGCCCCCTTTTCCTTGTTGGCTTTGGTTTTG

TGACTTATTACACATCAACTCTTCAAGCAGACTGCTATAGGTACCCTGACCCAGTCACAGGCAAGAGAAACTACACTTAC

AGGGAAGCTGTTAGAGCCTTTCTAGGTTCAAGAAGTCTGGTAATGTGCTCAATCGCACAATGTGCGATTCTTTGGGGTAT

AATGATTGGGTACACAATCATAGCAGCCACCAGTATGATAGCAGTGAAGAAGTCAAATTGCTTTCATAAGAATGGGCATA

ATGCTAGTTGCAAGACATCTGAGAATGTGTTTATGGTTACCTATGGATTGTTTCAAATAGTATTGTCCCAGCTACCAAGT

ATGCACAAATTAGCAGTGACTTCGGTGGTGGCAGCGATCATGTCGTTTGGTTATTCAGGGATAGGCCTCTCTCTAAGTGC

AGCAAAGTTGGTGTCTAATGGTGTGATCAAAGGAAGCATTGGAGGAAATTCTCTAGGGAATAGAGAGTCCTCTTTGGCGA

GCAATGTGTGGAATAGTTTCCAGGCTTTAGGAAATATTGCTTTCGCATACGTTTTCGCTCATGTTCTAACAGAAATTCAG

GACACTTTGAGATCACATCCTCCAGAGAACAAAGTCATGAAGAGAGCTACATTGTATGGGATTGTCGTTACTTCCGTGTT

CTACATGTCTCTTGGTTGTGTTGGGTATGCAGCATTTGGGAGTAAAACACCAGGCAACATCCTCACTGGATTTGGATTCT

ATGAACCATTTTGGCTTGTCGACATTGGGAACATATGCGTTGTGGTTCACCTCCTAGGGGCCTACCAGGTGTTCGCCCAG

CCGATCTTTGCGGCCATCGAGGACCGAGTCTCCTCAAAGTGGCCATCAAATTTCTTATTACAAGCTCGCTATGAAGTAAA

ATTACCTTGTTCAACCCAAACCTCATGGAGAGTGACCCTTTTCTCTTTGATCATGCGCACAACCATTGTGGTGATGACAA

CCTTAGTGGCTATTCTGGTGCCCTTGTTCAACTCAGTTGTAGGCCTCCTTGGTGCCATGGCCTTCTGGCCTCTCACTATA

TATTTCCCAGTTTCTATGCACATTGTTCAAGCTAATGTAACGAGGGGCAGTATGAAGTGGTTCCTCCTCCAATGCTTGGT

GGGGGCCTCTCTCATTGTCTCTGTCATAGCTGGCATAGGCTCATTGGTGGACATAACCAAAAGCCTTAAGCATTCAAAGC

CCTTCCAGGCTAAGTACTAA

>scaffold00071.165

ATGAAGGAAGATGAAGGAAGAAAGATGGAAGAGGGAGGGAACATGGATGATGGACGAGTGAGAAATGGGACAGTTTGGAC

TGCAACTGCCCATATAATTACAGCAGTGATAGGATCTGGGGTGTTGGGATTGGGGTGGAGTGTTTCTCAGTTGGGTTGGA

TTTTGGGGCCTTTTTGTCTCCTTGGCTTTGGCTATGTCACCTACTACACAGCAACTCTTCAAGCTGATTGCTATAGATAC

CCCAATCCACTCACTGGAAAGAGAAACTACACCTACAGAGATGCAGTGAGAGTATTTCTTGGGCCGAGAAATGTATTTAT

GTGTGGAATGGTGCAATATGCAATTCTTTGGGGTTCAATGGTGTCATACACCATCGTGGCTGCTTCAAGCATGGTTGCCA

TGAAGAAATCAAATTGCTTCCATAAGAGCGGGCACAACAGCAAATGTGGGACTTCAGGGATTATGTATATGTTTATCTAT

GGTTTATTTCAAGTGATATTGTCCCAATTACCAAATTTGGAGAAGGTATCAACGATATCAGTGGTGGCAACAATCACCTC

CTTTGTGTATTCAGGAATAGGACTAGCTCTATGCATAGCAAAGTTTGTGTGTCAAGGGGAGATCAAAGGAAGCTTAAGAG

GGAACTCCATTGGTGACACTGCTTCTTCCTCCATTTCAAGCAACATGTGGAATGCCTTCCAAGCTCTAGGAAGCATTGCT

TTTGCTTATGCTTTTGCCCATGTTCTCATAGAGATACAGGACACTTTGAGGCCACATCAACCGGAGAATAAAACTATGAA

GAGAGCAGCAGTTTATGGGATGGCATCCACTGCGATATTCTATGTCTCACTGGGTTCTGCAGCCTATGCCGCGTTCGGAA

GTCACACTCCTGGAAACATCCTAACTGGTTTCGGATTCTATGAACCCTTTTGGCTGGTGGACATCGGGAACCTTTGCATT

GCCATTCATCTCACAGGAGCTTACCAGGTGTACGGGCAACCCATCTTTGCCGCAATTGAGGACATGATATACTCGACCTG

GCCAACAAACTGCTTTGTCCATCTGCAATTGACAGTCAAATTACCATTTTCGAACTTGGGTGGCGTAAGCTTGTCCCCCC

TGTCTCTCCTCGTGCGCACAACCGTAGTTGTGGTAACAACGTTAGTGGCCATGCTTGTGCCGTTCTTCAACTCAGTTGCT

GGTCTCATTGGGGCTATAGCATTTTGGCCTCTCACTGTATACTTCCCTGTGAACATGTACATAGCACAGGCTAGGCTAAA

GAGGGGTACCATGAAATGGGTCCTCTTACAATGCCTCCTAGCTGCTTCTTTTGTGGTCTCTCTTTTAGCTGCTATCGGTT

CGGTGGCTGATATCATCAAGAGCCTCAAGCATTCGAAGCCCTTCAAAGCTGTGTATTAA

>scaffold00071.167

ATGGAGATGGAGATGGATGAGGTCATGGCAGGTAGGGCATTCGAGGATGAAGAAGATGATCACAAGAGAGAAGGAACAGT

GTGGACCGCGACGGCTCATGTGATCACAGCTGTGATTGGGTCGGGGGTTCTTGCACTAGGATGGAGCGTGGCCCAATTGG

GGTGGATGATGGGGCCATTAACTGTGGTGGCCTTTGCATGGGTTACTTACTACACAGCAAACCTTCTATCCGATTGTCAC

AGGTCTCCTCATCCAACCACTGGCCACCGGAATCACACTTACATCGATGCTGTGAGGGCATGCCTAGGTCCACAAAAAGT

GCTCATATGTGGGGTTGCTCAATACTCGAACCTTGTTGGAACTTTGATTGGATACACCATTACGTCTGCCATTAGTATGA

TGGCAATAAAGAGGTCAGATTGCTTCCATGAAAACGGCCATAATTCAAGATGTGGAGTGTCTGGAAATTTATATGTGGCA

CTATTTGGGGTGCTTGAAGTGATATTATCTCAGCTCCCCAGCTTGGAGAAAATTTCGTGGCTCTCAATTTTGGCTGCAAT

TATGTCTTTTGCTTATTCTTTCATTGGGCTTGGGCTTAGTATTGCCATGGTCATCTGTCATGGAGATATTAGAGGCACCT

TATTTGGAGTCAAGGTTGGCATCAATGACATTCCTCTAACAGCCAGGACATGGAATGCATTCCAAGCCCTTGGCAACATT

GCTTTCGCTTACACTTATGCATTGGTTCTTATAGAAATTCAGGACACTCTGAAATCTTCTCCCCCGGAAAATAAGGCGAT

GAAGAGGGCCTCGTTGTACGGGATCGGTATTACTGCAATATTTTACTTCTCAATTGGTTCTGCTGGTTATGCTGCTTTCG

GGAATGATGCTCCCGGCAACATGCTCACCGGGTTCGGTTTTTACGAGCCCTTTTGGCTTATTGACATTGCCAACTTGTTC

ATTGTCATCCACCTGGTGGGTGCTTACCAAGTATTTTCACAACCAGTTTTTGCAGCATGGGAGATGTATCTCTCCTCCAA

ATGGTCACAAAATAGCATTGTGCACTCCACCTACAATGTGAAATTACCTTTGGTGCCTTCAACCTCCTTCAATTTTACCC

TCTCAAAGCTCATCTCTCGCACTCTTCTTGTGATCATAGTGACATTGGTTGCCATGACTTTCCCATTCTTTAATGCAGTA

AATGGTCTCATAGGTGCCATTGCTTTTTGGCCATTGCAAGTTTATTTCCCCACAACCATGTACATCTCTCAAATGAAAGT

GAAGAAGGGGACCCAAAAATGGATATTCTTAAAGACTCTTAGTGTATGTTGCTTTTTTGTTAGCCTCATAGCAGTTGTTG

GCTCAGTGGCTGGTATAGTTGATAGTCTTAAGCATGCCACACCTTTCCAAACCATGTATTAG

>scaffold00166.19

ATGGAAGACCAGGAGAATGGCCTGCAGAAGGAGAGAAGTCCAAAGGCACTTAGCATCGAACCTGGCTTAAGCACTGCTCA

TACAATTGATCAGGATCCATGGATACAAGTGGGGCTTCTTCTTGTCACAAGCTTCAGCTGTGGTTACATGTTGGGCTATT

CTAATCTTATTCTAAAGCCTTTAGGGTGGACATGGGGGCTTATCGCTATGCTTATTATCGGATTCTTGGCTCTCTATGCA

AACTGGCTCTTGGCTGGGTTTCACGTAATCGATGGACAGAGGTTCATAAGATACAGGGATATGATGGGCTACTTATTTGG

AAGAAGAATGTATCATATAACCTGGTCTCTGCAATTTCTAATGTTTCTCCTTGGAAACATGGGTTTTATCCTACTTGCAG

GGAGATCTCTAAAGGCGATTCATGCTGAGTTTAGTCTCTCCACACTGAGACTTCAAATCTACATCATCATAACCGGGGCT

ATCTACTTTCTCTTTGCTTTGATGATTCCAAATATGTCAGCCATGAGGCACTGGTTAGGAGTCTCAAGCGTTCTCACTAT

TACATATGTAGTGATTGTAATGGCAATCTGCGTAAAGGATGGGAAATCTAGTGTGAGTAAGAGTTATGCCATAGAGGGGT

CAGGTACAGAGAAATTTTTCAATGCATTCAATGCATTTTCAGCAATTCTTTTTGCTGACACATCGGGCATGCTCCCCGAA

ATTCAGGCAACACTGCGAAAGCCTGTTGTGAAAAACATGAGAAAGGCACTATGCATGCAATTCACATTAGGTCTGGCAAT

ATACTATGCTGTGACCATTTTAGGATATTGGGCATATGGATCTAATGTCTCTGAGTATCTCCCCAACAATTTCAGTGGAC

CCAAGTGGGCTGTTATAGTTGCAAATGCAACTGTCTTCTTACAAACAATCGTATCGCAACATATGTTTTGTACACCAGTA

CATGAGGCTCTTGATACAAGATTCTTACACTTAGATCAAAGTATGCATTCAAGAGATAACATCAAGATCCTCTTCGTATT

GCGTGCTGGCCTTTTCACTCTGAACACATTTATGGCTGCCCTGATTCCTTTCTTGGGAGATTTTGTGAACTTAATCGGTT

CTCTCTCTCTTTTCCCCCTCACTTTTGTCTTCCTAAGCATGATCTTCTTGAAGGTTCGAGGAAAAACAGCAAATAGTGTG

GTGAAAGCATGGCACTGGATCAATATAGTTGGATTCTCTGTTATAACCATACTAACAACTGTAGCGGCTCTTCGATTAAT

TGTGGAGAATGCAAAGGCTTACAGAATATTTGCAGATAATTAA

>scaffold00166.21

ATGGAAGACCATGATGGGTTGCAGAAAGAGAGAAATGAAGCAAAAGCATTGAATGATGACAGTGGTTTGAACACAGCTCA

TAGAATTGATCATGATCCATGGCTACAAGTGGGGCTTCTTCTGGTTACAGGCTACAATTGTGGATATATCGTGAGCTTTT

CAAACCTCATTCTGAAGCCCTTAGGATGGACATGGGGGTTGATTGCTATGGTTTTTATAGCCATTTTATCTCTCTATGCA

AACTGGCTCTTAGCAGGGTTTCACATAATTGATGGGCAGAGGTTCATAAGATACAGAGATTTGATGGGCTGTTTATTTGG

AAAGAAGATACATCATATGACATGGGCTCTCCAAGTTTTGAATCTTTTGTTTGCAAATATGGGTTTCATTCTTCTTGCTG

GAAATTCACTAAAGGAAAGCTACATTTCTCCTTCATTTTCCCTTTATTCTCTCTGCGAAATATTTCCCCTTGAGATCCAT

GCGGCGTTTACATCCTCACCATTACGACTTCAAGATTACATAATTATAAGTGGAGTGATCTGTTTCCTCTTCGCTTTCAT

TGTCCCCAATATGTCATCCATGGGAGCATGGTTCGCCCTCTCAGGCTTCTTCACCCTCATTTATGTAGTCACTATAATAG

CAGTCTCTATAAAAGATGGAAAGTCTAACATAAAAAAAGACTATAGTGTTAGTAGATCAAATGTGGAGAAGGTTTTCAAT

GCATTCAATGCAATTTCAGCTATTCTTTTCACTAATACCTCAGGCATGCTTCCTGAGATCCAGTGTACTCTTCATAAACC

AGCAGTGAAGAACATGAGGAAAGCATTGTATCTGCAATTCACCTTGGGCCTAGGAATATACTACGTTATAACAATCATAG

GATATTGGGCTTATGGTTCACATGTCTCTGAGTATCTTCCTGATCAATTTAGTGGGCCTAAGTGGGCTTCAATCATTGCA

AATTCAGCAATATTCTTTCAAACAATCATATCGCAGAATGTGTTCTGTTCACCAATTCATGAGGCACTTGATACTAGATT

CTTAAGACTTGACCAGAGCTTGTATTCGCTCGACAATGTCAAGAGGCGTTTATTGCTGCGGGGTGGCCTGTTTCTCCTCA

ACACATTTATAGCTGCCATGTTTCCTTTCTTAGGAGATTTCGTGAACCTTGTTGGTTCTTTCTCACTCTTCCCTCTAACT

TTTATCTTCCCAAGCATGATCTTTATAAAGGTTCAAGGGAGAAAAGCTAACAGTGCGGTGAAGGCATGGCACTGGCTAAA

CATCATCGGTTTTTCGTTTGTAACACTTGTAACAACCATTGCGGCCCTTCGGTTGATTGTACAGAACGCTAAGCTATACC

ATCTGTTTGCAGACACGTGA

>AtAAP1

ATGAAGAGTTTCAACACAGAAGGACACAACCACTCCACGGCGGAATCCGGCGATGCCTACACCGTGTCGGACCCGACAAA

GAACGTCGATGAAGATGGTCGAGAGAAGCGTACCGGGACGTGGCTTACGGCGAGTGCGCATATTATCACGGCGGTGATAG

GCTCCGGAGTGTTGTCTTTAGCATGGGCTATAGCTCAGCTTGGTTGGATCGCAGGGACATCGATCTTACTCATTTTCTCG

TTCATTACTTACTTCACCTCCACCATGCTTGCCGATTGCTACCGTGCGCCGGATCCCGTCACCGGAAAACGGAATTACAC

TTACATGGACGTTGTTCGATCTTACCTCGGTGGTAGGAAAGTGCAGCTCTGTGGAGTGGCACAATATGGGAATCTGATTG

GGGTCACTGTTGGTTACACCATCACTGCTTCTATTAGTTTGGTAGCGGTAGGGAAATCGAACTGCTTCCACGATAAAGGG

CACACTGCGGATTGTACTATATCGAATTATCCGTATATGGCGGTTTTTGGCATTATTCAGGTTATTCTTAGCCAGATCCC

AAATTTCCACAAGCTCTCTTTTCTTTCCATTATGGCCGCGGTCATGTCCTTTACTTATGCAACTATTGGAATCGGTCTAG

CCATCGCAACCGTCGCAGGTGGGAAAGTGGGTAAGACGAGTATGACGGGCACAGCGGTTGGAGTAGATGTAACCGCAGCT

CAAAAGATATGGAGATCGTTTCAAGCGGTTGGGGACATAGCGTTCGCCTATGCTTATGCCACGGTTCTCATCGAGATTCA

GGATACACTAAGATCTAGCCCAGCTGAGAACAAAGCCATGAAAAGAGCAAGTCTTGTGGGAGTATCAACCACCACTTTTT

TCTACATCTTATGTGGATGCATCGGCTATGCTGCATTTGGAAACAATGCCCCTGGAGATTTCCTCACAGATTTCGGGTTT

TTCGAGCCCTTTTGGCTCATTGACTTTGCAAACGCTTGCATCGCTGTCCACCTTATTGGTGCCTATCAGGTGTTCGCGCA

GCCGATATTCCAGTTTGTTGAGAAAAAATGCAACAGAAACTATCCAGACAACAAGTTCATCACTTCTGAATATTCAGTAA

ACGTACCTTTCCTTGGAAAATTCAACATTAGCCTCTTCAGATTGGTGTGGAGGACAGCTTATGTGGTTATAACCACTGTT

GTAGCTATGATATTCCCTTTCTTCAACGCGATCTTAGGTCTTATCGGAGCAGCTTCCTTCTGGCCTTTAACGGTTTATTT

CCCTGTGGAGATGCACATTGCACAAACCAAGATTAAGAAGTACTCTGCTAGATGGATTGCGCTGAAAACGATGTGCTATG

TTTGCTTGATCGTCTCGCTCTTAGCTGCAGCCGGATCCATCGCAGGACTTATAAGTAGTGTCAAAACCTACAAGCCCTTC

CGGACTATGCATGAGTGA

>AtAAP2

ATGGGTGAAACCGCTGCCGCCAATAACCACCGTCACCACCACCATCACGGCCACCAGGTCTTTGACGTGGCCAGCCACGA

TTTCGTCCCTCCACAACCGGCTTTTAAATGCTTCGATGATGATGGCCGCCTCAAAAGAACTGGGACTGTTTGGACCGCGA

GCGCTCATATAATAACTGCGGTTATCGGATCCGGCGTTTTGTCATTGGCGTGGGCGATTGCACAGCTCGGATGGATCGCT

GGCCCTGCTGTGATGCTATTGTTCTCTCTTGTTACTCTTTACTCCTCCACACTTCTTAGCGACTGCTACAGAACCGGCGA

TGCAGTGTCTGGCAAGAGAAACTACACTTACATGGATGCCGTTCGATCAATTCTCGGTGGGTTCAAGTTCAAGATTTGTG

GGTTGATTCAATACTTGAATCTCTTTGGTATCGCAATTGGATACACGATAGCAGCTTCCATAAGCATGATGGCGATCAAG

AGATCCAACTGCTTCCACAAGAGTGGAGGAAAAGACCCATGTCACATGTCCAGTAATCCTTACATGATCGTATTTGGTGT

GGCAGAGATCTTGCTCTCTCAGGTTCCTGATTTCGATCAGATTTGGTGGATCTCCATTGTTGCAGCTGTTATGTCCTTCA

CTTACTCTGCCATTGGTCTAGCTCTTGGAATCGTTCAAGTTGCAGCGAATGGAGTTTTCAAAGGAAGTCTCACTGGAATA

AGCATCGGAACAGTGACTCAAACACAGAAGATATGGAGAACCTTCCAAGCACTTGGAGACATTGCCTTTGCGTACTCATA

CTCTGTTGTCCTAATCGAGATTCAGGATACTGTAAGATCCCCACCGGCGGAATCGAAAACGATGAAGAAAGCAACAAAAA

TCAGTATTGCCGTCACAACTATCTTCTACATGCTATGTGGCTCAATGGGTTATGCCGCTTTTGGAGATGCAGCACCGGGA

AACCTCCTCACCGGTTTTGGATTCTACAACCCGTTTTGGCTCCTTGACATAGCTAACGCCGCCATTGTTGTCCACCTCGT

TGGAGCTTACCAAGTCTTTGCTCAGCCCATCTTTGCCTTTATTGAAAAATCAGTCGCAGAGAGATATCCAGACAATGACT

TCCTCAGCAAGGAATTTGAAATCAGAATCCCCGGATTTAAGTCTCCTTACAAAGTAAACGTTTTCAGGATGGTTTACAGG

AGTGGCTTTGTCGTTACAACCACCGTGATATCGATGCTGATGCCGTTTTTTAACGACGTGGTCGGGATCTTAGGGGCGTT

AGGGTTTTGGCCCTTGACGGTTTATTTTCCGGTGGAGATGTATATTAAGCAGAGGAAGGTTGAGAAATGGAGCACGAGAT

GGGTGTGTTTACAGATGCTTAGTGTTGCTTGTCTTGTGATCTCGGTGGTCGCCGGGGTTGGATCAATCGCCGGAGTGATG

CTTGATCTTAAGGTCTATAAGCCATTCAAGTCTACATATTGA

>AtAAP3

ATGGTTCAAAACCACCAAACAGTTCTCGCCGTCGATATGCCACAAACCGGCGGCTCCAAGTACTTGGACGACGACGGGAA

AAACAAAAGAACTGGGAGTGTTTGGACGGCGAGTGCACACATAATAACGGCAGTGATAGGTTCGGGAGTTTTGTCACTAG

CGTGGGCTACGGCGCAGCTAGGTTGGCTCGCCGGACCGGTGGTGATGTTGCTCTTCTCTGCCGTCACTTATTTCACTTCT

TCTCTTCTTGCTGCTTGTTACCGCTCCGGCGACCCTATCTCCGGCAAGAGGAACTACACTTATATGGATGCTGTCCGATC

AAATCTCGGTGGCGTGAAGGTGACGCTATGTGGGATTGTTCAGTATCTTAATATCTTTGGTGTTGCTATTGGCTACACAA

TTGCTTCAGCTATAAGCATGATGGCAATAAAGAGATCAAACTGTTTCCACAAGAGTGGAGGGAAAGATCCATGTCACATG

AACAGTAATCCTTACATGATAGCTTTTGGATTAGTCCAGATTCTATTCTCTCAGATTCCAGATTTTGATCAACTTTGGTG

GCTCTCAATCCTCGCCGCCGTTATGTCCTTCACTTATTCCTCAGCCGGTCTCGCCCTCGGCATAGCCCAAGTTGTCGTTA

ATGGGAAGGTGAAGGGAAGTCTCACTGGGATTAGCATAGGAGCAGTAACAGAGACACAGAAGATATGGAGGACCTTTCAA

GCTCTTGGAGACATTGCTTTTGCTTACTCTTACTCCATTATCCTCATCGAGATTCAGGACACAGTGAAGTCACCACCATC

AGAAGAGAAGACGATGAAGAAGGCAACACTTGTGAGCGTCAGTGTAACGACTATGTTCTATATGTTGTGTGGATGTATGG

GATATGCAGCCTTTGGAGACTTGTCTCCGGGAAATCTCTTAACCGGTTTCGGGTTTTATAATCCTTATTGGCTTCTTGAC

ATTGCAAATGCAGCCATTGTGATTCACCTTATTGGTGCATACCAAGTCTATTGCCAACCTCTGTTTGCTTTCATCGAGAA

GCAAGCTTCCATTCAATTCCCTGATAGTGAGTTCATTGCAAAAGATATCAAAATTCCAATTCCTGGTTTCAAGCCTCTCC

GCTTGAATGTCTTCAGGTTGATATGGAGGACAGTGTTTGTGATCATAACGACAGTTATCTCAATGCTTCTTCCGTTTTTC

AACGACGTTGTGGGTCTGCTCGGGGCACTAGGGTTTTGGCCATTGACGGTGTATTTTCCAGTGGAAATGTACATCGCGCA

GAAGAAGATACCTAGATGGAGCACCAGATGGGTTTGCCTTCAAGTCTTCAGCTTAGGGTGTCTAGTAGTTAGCATTGCTG

CAGCTGCAGGGTCCATAGCTGGAGTACTTCTTGATCTAAAGTCCTACAAGCCATTTCGAAGCGAATACTGA

>AtAAP4

ATGGATGTTCCACGGCCAGCTTTCAAATGTTTTGATGACGATGGCCGGCTTAAACGTTCAGGGACGGTTTGGACCGCGAG

TGCGCATATCATAACCGCCGTGATTGGATCTGGTGTTCTATCGCTTGCGTGGGCTATAGGTCAACTCGGTTGGATCGCAG

GTCCTACAGTGATGTTGTTGTTCTCTTTTGTCACTTACTACTCTTCCACGCTTCTTAGCGACTGCTACAGAACCGGAGAT

CCTGTCTCTGGGAAGAGAAACTATACTTACATGGACGCTGTCCGATCAATCCTAGGTGGCTTTAGGTTCAAGATTTGTGG

GCTGATTCAGTATTTGAATCTGTTTGGTATCACGGTCGGGTACACAATCGCAGCATCTATAAGTATGATGGCGATCAAGA

GGTCCAACTGTTTCCACGAGAGCGGAGGGAAAAACCCGTGTCACATGTCGAGCAATCCATACATGATCATGTTTGGTGTG

ACCGAGATCTTGCTCTCTCAGATCAAAGATTTTGACCAGATTTGGTGGCTCTCCATTGTCGCTGCTATCATGTCCTTCAC

ATACTCTGCAATCGGTTTAGCTCTCGGAATCATTCAAGTCGCGGCAAATGGAGTTGTCAAGGGAAGTCTCACCGGAATTA

GCATCGGCGCAGTGACTCAGACCCAAAAAATATGGAGAACCTTTCAAGCACTTGGAGACATTGCCTTTGCTTATTCATAC

TCTGTTGTTCTTATTGAAATTCAGGACACTGTAAGATCTCCACCAGCAGAATCAAAAACGATGAAGATCGCCACAAGAAT

CAGCATCGCTGTTACAACGACATTTTACATGCTATGTGGTTGTATGGGCTATGCCGCCTTCGGAGATAAAGCACCGGGAA

ACCTCTTAACCGGTTTTGGTTTCTACAATCCGTTTTGGCTCCTTGACGTGGCTAACGCTGCCATAGTTATCCACCTTGTA

GGAGCTTATCAAGTCTTTGCTCAGCCCATCTTCGCCTTTATTGAGAAACAAGCTGCCGCTAGGTTTCCCGACAGTGACTT

GGTGACCAAGGAATACGAAATCCGAATCCCTGGTTTTAGGTCACCGTACAAAGTCAACGTTTTCAGAGCAGTTTACCGAA

GCGGGTTTGTGGTTTTGACCACTGTGATATCCATGCTTATGCCGTTTTTCAACGACGTCGTAGGGATTTTAGGTGCGTTA

GGGTTTTGGCCTTTGACGGTTTACTTTCCGGTGGAGATGTATATAAGACAGAGGAAGGTTGAGAGATGGAGTATGAAGTG

GGTTTGTCTGCAGATGTTGAGCTGTGGTTGTTTGATGATCACGTTGGTCGCCGGAGTTGGCTCCATCGCCGGAGTAATGC

TAGACCTTAAGGTTTACAAGCCGTTCAAGACTACTTACTAA

>AtAAP5

ATGGTCGTTCAGAATGTTCAAGATTTGGATGTTCTTCCGAAACATAGCTCGGATTCATTCGATGATGATGGTCGTCCCAA

GCGAACCGGGACGGTGTGGACAGCAAGTGCACACATAATAACAGCAGTAATTGGGTCAGGAGTGTTGTCATTGGCATGGG

CCGTTGCTCAGATTGGTTGGATTGGTGGTCCAGTGGCTATGTTACTCTTTTCTTTCGTTACTTTTTACACATCTACCCTT

CTCTGTTCTTGTTACCGATCTGGTGACTCTGTTACCGGCAAAAGAAACTACACTTACATGGACGCTATTCACTCCAACCT

CGGTGGGATTAAGGTGAAAGTATGTGGAGTTGTGCAATATGTTAATCTATTTGGTACAGCAATTGGATATACAATTGCAT

CAGCTATAAGTTTGGTAGCAATCCAAAGGACAAGTTGCCAACAGATGAATGGACCAAATGATCCTTGTCATGTTAATGGA

AATGTTTACATGATTGCGTTTGGTATTGTACAAATCATATTCTCTCAGATTCCTGATTTTGATCAGTTATGGTGGCTTTC

CATTGTCGCTGCGGTCATGTCTTTTGCTTATTCTGCCATTGGACTTGGCCTTGGTGTCTCTAAAGTGGTAGAGAACAAAG

AAATTAAAGGTAGTCTCACTGGAGTCACTGTTGGGACAGTGACACTGAGTGGGACAGTGACATCGTCGCAGAAAATATGG

AGAACATTTCAGTCTCTTGGCAACATTGCGTTTGCGTACTCTTACTCGATGATTCTCATCGAAATTCAGGACACGGTGAA

ATCACCGCCTGCCGAAGTAAACACGATGAGAAAAGCGACTTTTGTAAGTGTGGCAGTGACTACAGTCTTCTACATGCTAT

GTGGCTGTGTAGGGTATGCAGCATTTGGGGATAACGCACCCGGAAATCTCCTCGCTCATGGAGGGTTTAGAAACCCATAT

TGGTTGCTTGACATTGCTAATCTCGCTATAGTGATCCATCTAGTCGGTGCTTACCAAGTCTATTGCCAACCCCTCTTTGC

CTTCGTCGAGAAGGAAGCCTCTAGGAGGTTTCCAGAGAGCGAATTCGTCACAAAAGAGATCAAGATCCAACTCTTCCCGG

GGAAGCCCTTTAACTTGAATCTCTTCAGGCTCGTATGGAGGACGTTCTTCGTGATGACAACAACGTTGATCTCAATGTTA

ATGCCTTTCTTTAACGATGTAGTCGGTCTCTTGGGGGCCATTGGTTTTTGGCCTTTAACGGTTTATTTCCCAGTGGAAAT

GTACATTGCACAGAAGAATGTTCCGAGATGGGGCACAAAGTGGGTTTGTCTACAAGTCTTGAGTGTGACATGTCTTTTTG

TGTCCGTAGCCGCGGCCGCGGGATCTGTTATTGGCATTGTTAGTGATCTCAAGGTCTACAAACCTTTCCAGTCTGAATTC

TGA

>AtAAP6

ATGGAGAAGAAGAAGAGCATGTTCGTTGAACAGAGCTTCCCGGAGCATGAAATTGGCGATACTAACAAAAACTTTGACGA

GGATGGCCGCGACAAAAGAACTGGGACATGGATGACCGGGAGTGCACACATAATAACGGCCGTGATAGGGTCGGGAGTGT

TGTCTTTGGCGTGGGCAATCGCACAACTTGGATGGGTGGCAGGACCCGCCGTACTAATGGCTTTTTCTTTCATAACATAT

TTTACATCAACCATGCTTGCCGATTGTTACCGTTCCCCTGACCCTGTTACCGGCAAACGCAACTACACCTACATGGAAGT

TGTCCGATCCTATCTAGGAGGAAGAAAAGTGCAATTATGTGGATTGGCTCAATACGGGAATCTGATTGGAATAACAATCG

GCTACACAATCACAGCTTCAATTAGCATGGTGGCAGTGAAGAGGTCGAATTGTTTCCACAAAAATGGGCATAATGTTAAA

TGTGCCACTTCAAACACTCCCTTCATGATCATATTTGCAATCATCCAAATTATTCTTAGCCAAATCCCAAATTTCCATAA

CCTCTCTTGGCTCTCCATTCTTGCGGCCGTAATGTCCTTTTGTTATGCCTCCATCGGTGTTGGTCTCTCCATCGCCAAAG

CGGCGGGTGGCGGTGAGCACGTAAGAACAACACTGACAGGAGTTACGGTCGGGATTGATGTATCGGGTGCCGAGAAAATA

TGGAGAACGTTCCAAGCGATTGGGGACATTGCATTTGCCTACGCATACTCAACTGTTCTCATTGAAATACAGGACACCTT

GAAAGCAGGTCCTCCATCGGAGAACAAAGCCATGAAAAGAGCAAGCCTTGTGGGTGTCTCCACAACAACCTTCTTCTACA

TGTTATGCGGTTGTGTTGGTTACGCTGCCTTTGGAAATGATGCTCCTGGAAATTTCCTAACTGGTTTTGGTTTCTATGAG

CCATTCTGGCTAATCGACTTTGCCAATGTCTGCATCGCCGTGCACCTTATCGGCGCCTACCAAGTCTTTTGTCAACCAAT

TTTTCAGTTCGTAGAGAGCCAGAGCGCGAAACGTTGGCCTGATAACAAGTTTATTACAGGAGAATACAAAATCCATGTCC

CTTGCTGTGGTGATTTTAGTATCAACTTCCTCAGATTGGTATGGAGGACTTCATATGTTGTGGTCACCGCGGTTGTAGCC

ATGATCTTCCCTTTCTTCAACGATTTCTTGGGTCTTATTGGAGCAGCTTCCTTCTGGCCTTTGACTGTTTACTTTCCCAT

TGAGATGCATATTGCTCAGAAGAAGATACCGAAATTCTCTTTCACTTGGACTTGGTTAAAAATCTTGAGTTGGACTTGTT

TCATTGTGTCCCTCGTTGCTGCAGCCGGATCAGTGCAAGGACTCATACAAAGTCTCAAGGATTTCAAGCCTTTCCAGGCT

CCTTAG

>AtAAP7

ATGGACATTAAAGAAGATGACGAGTCTCGAGTTATAACACCAACCGAGCTTCAACTTCATGATTCAGTTACTGCAAGAAC

AGGAACGTTATGGACGGCGGTGGCACATATAATAACAGGAGTGATAGGAGCTGGAGTGTTGTCGTTGGCTTGGGCCACGG

CGGAGCTCGGCTGGATCGCTGGTCCGGCCGCCCTTATAGCCTTCGCCGGGGTCACACTTCTCTCTGCTTTTCTTCTTTCC

GATTGCTACCGTTTCCCTGATCCTAACAACGGTCCCCTCCGACTCAATTCTTACTCTCAAGCCGTTAAATTGTATTTAGG

GAAGAAGAATGAAATTGTATGTGGAGTCGTTGTATACATTTCCCTTTTTGGTTGTGGCATTGCTTATACCATTGTTATAG

CTACATGCAGTAGAGCAATTATGAAATCGAATTGTTATCATAGAAATGGACACAATGCAACATGTTCATATGGAGACAAC

AACAACTACTTCATGGTTTTGTTTGGTTTGACTCAGATATTTATGTCACAAATACCTAATTTCCACAACATGGTATGGCT

CTCTCTTGTCGCCGCTATTATGTCTTTTACTTACTCGTTCATTGGCATCGGCCTCGCCCTTGGCAAAATCATAGAGAATC

GAAAAATTGAGGGAAGTATAAGGGGAATACCAGCAGAAAACAGAGGTGAAAAGGTATGGATAGTGTTCCAAGCTCTTGGG

AACATTGCCTTTTCATATCCTTTCTCAATCATACTTCTTGAGATTCAGGACACATTGAGATCACCACCGGCAGAGAAGCA

AACGATGAAGAAAGCCTCGACGGTTGCGGTATTCATCCAAACATTCTTCTTCTTCTGTTGTGGATGTTTTGGGTACGCGG

CCTTTGGAGATTCCACCCCGGGAAATCTCTTGACCGGTTTCGGCTTCTATGAGCCATTCTGGCTCGTCGATTTCGCCAAC

GCTTGCATTGTTCTTCATTTAGTTGGTGGATATCAGGTATACAGTCAACCAATTTTCGCGGCTGCGGAGAGATCGCTAAC

CAAGAAATATCCGGAAAATAAGTTTATCGCCAGATTCTACGGATTCAAACTGCCATTGTTGCGAGGAGAGACGGTGAGAT

TGAACCCAATGAGGATGTGTTTGAGAACGATGTACGTGTTGATCACAACGGGAGTGGCAGTGATGTTTCCCTACTTCAAT

GAAGTATTAGGAGTGGTGGGGGCACTTGCATTTTGGCCTCTGGCAGTGTATTTTCCTGTGGAGATGTGTATATTGCAGAA

GAAAATCCGAAGTTGGACGCGACCATGGCTTCTTCTTAGAGGTTTCAGCTTTGTGTGCTTGCTCGTCTGTCTCTTGTCTC

TTGTTGGATCTATTTATGGACTTGTTGGAGCTAAATTCGGATGA

>AtAAP8

ATGGACGCATACAACAATCCCTCGGCGGTGGAGTCGGGTGACGCCGCCGTGAAAAGCGTCGACGACGATGGTCGAGAGAA

GAGAACGGGAACATTTTGGACGGCGAGTGCGCACATAATCACGGCGGTCATAGGCTCAGGGGTGCTGTCGTTGGCTTGGG

CTATAGCACAGCTTGGTTGGGTGGCAGGAACCACAGTTTTGGTCGCTTTCGCCATCATTACTTACTACACGTCCACCTTG

CTCGCCGACTGTTACCGTTCGCCGGACTCCATCACCGGAACACGCAACTATAATTACATGGGCGTCGTCCGATCTTACCT

TGGTGGTAAAAAGGTTCAGCTATGTGGAGTGGCACAGTACGTGAATCTCGTAGGGGTCACTATTGGTTACACCATCACTG

CCTCCATAAGCTTAGTAGCGATTGGGAAATCAAATTGTTATCATGACAAGGGACATAAAGCGAAATGTTCTGTATCGAAT

TATCCATACATGGCGGCATTTGGGATCGTCCAGATCATTCTGAGCCAGCTTCCTAACTTCCACAAGCTCTCTTTCCTATC

CATCATCGCCGCGGTTATGTCCTTCTCTTATGCGTCTATCGGAATAGGCCTAGCCATCGCTACTGTAGCAAGTGGGAAGA

TTGGTAAGACAGAATTGACAGGGACAGTGATAGGTGTGGACGTAACTGCGTCTGAAAAAGTTTGGAAATTGTTTCAAGCG

ATTGGAGACATTGCCTTTTCATACGCTTTTACCACTATTCTCATCGAGATTCAGGACACATTGAGATCAAGCCCACCAGA

GAACAAAGTGATGAAACGAGCAAGTCTTGTCGGAGTCTCAACCACAACTGTTTTCTACATCTTGTGTGGTTGCATCGGAT

ATGCTGCGTTCGGCAACCAAGCCCCTGGTGACTTCCTTACCGATTTTGGTTTTTACGAACCTTATTGGCTCATCGACTTT

GCCAATGCTTGCATTGCTCTCCATCTAATAGGTGCCTATCAGGTGTATGCGCAGCCGTTTTTCCAGTTTGTTGAGGAAAA

CTGCAACAAAAAATGGCCTCAAAGCAATTTCATCAACAAAGAATACTCGTCAAAGGTTCCTTTGCTTGGAAAATGTCGTG

TCAACCTCTTCAGACTGGTTTGGAGGACATGCTATGTTGTTTTGACAACATTTGTAGCAATGATATTCCCCTTCTTCAAT

GCGATCTTGGGTTTGCTAGGGGCATTCGCGTTCTGGCCACTCACAGTTTATTTTCCGGTGGCAATGCACATTGCGCAGGC

TAAAGTCAAGAAGTATTCTCGTAGATGGTTGGCCTTGAACCTCCTCGTATTGGTTTGCTTGATCGTCTCGGCCCTTGCCG

CCGTAGGATCCATCATTGGCTTAATTAATAGTGTCAAGTCATACAAGCCCTTCAAGAATTTAGACTAG

>StAAP1

ATGGCACCTGAATTTCAAAAGAACGCTATGTACGTCTCAAATGAACTCGAAAATGGAGACGTTCAAAAAAACTTTGATGA

TGATGGACGTGAAAAAAGAACAGGTACTTTGTTGACTGCGAGTGCACATATTATTACTGCTGTGATTGGCTCAGGAGTGC

TTTCACTAGCATGGGCTATAGCTCAATTAGGATGGGTGGCTGGTCCTGCTGTTCTCTTTGCCTTTTCTTTCATCACATAT

TTCACTTCTACACTTCTTGCCGACTGTTACCGATCTCCGGGACCGATTTCCGGCAAGAGAAACTATACTTACATGGACGT

TGTCCGCTCTCACTTAGGAGGTGTGAAGGTGACACTGTGTGGAATTGCACAATATGCTAACCTTGTTGGAGTTACAATTG

GATATACAATTACAGCATCTATTAGTATGGTGGCTGTAAAGAGGTCAAATTGTTTTCACAAAAATGGGCATGAAGCAAGT

TGCTCAATTGAAAGTTACCCATACATGATAATATTTGCAGTAATTCAAATAGTTCTTAGCCAAATTCCAAATTTCCACAA

GCTCTCATGGTTATCAATTCTTGCTGCTGTTATGTCTTTTACTTATGCTTCTATTGGTCTTGGACTATCCATAGCCAAAG

TCTCAGGTGTGGGGCACCATGTGAAGACAAGCCTAACAGGGGTGGTAGTAGGTGTGGATGTATCTGGAACAGAAAAAGTA

TGGAGAAGTTTCCAAGCTATTGGAGATATTGCATTTGCTTATGCTTATTCCACAGTTCTTATTGAAATACAGGACACACT

GAAATCATCACCACCAGAAAGCAAGGTAATGAAGAGAGCCTCACTAGCTGGAGTTTCCACCACAACTTTATTCTATGTAC

TTTGTGGTACCATTGGCTATGCAGCCTTTGGAAACAATGCTCCAGGAAATTTTCTTACTGGATTTGGTTTCTATGAACCT

TTTTGGCTAATTGACTTTGCCAACGTCTGCATCGCTGTCCACCTTGTCGGAGCTTATCAGGTTTTCTGCCAACCATTATA

TGGATTCGTGGAGGCTCGTTGCAGCCAGCGATGGCCAGACAGCAAATTCATCACCTCAGAATACGCGATGCGAGTTCCAT

GCTGTGGAACTTACAACCTCAACTTATTCAGGTTGGTGTGGAGAACAACATATGTTATAGTGACAGCGGTGATTGCAATG

ATATTCCCCTTCTTCAACGACTTCTTGGGGTTGATCGGAGCAGCATCGTTTTATCCATTAACCGTTTACTTCCCAATAGA

GATGTACATTGCTCAGAGAAAGATACCAAAGTATTCTTTCACATGGGTATGGCTGAAAATATTGAGTTGGACTTGTTTGA

TCGTATCACTTGTTGCAGCTGCTGGATCCATCCAGGGACTTGCCACTGATGTTAAGGGTTACAAGCCTTTCTCAACTCAT

CAATAA

>StAAP2

ATGGCAGACCATGTTTCGTTGAGTGTGCCTGATGAATCCACATGTTTTGATGATGATGGCCGTCCCAAAAGAAGTGGTAC

AGTTTGGACTGCAAGTGCACACATAATAACAGCTGTGATTGGTTCTGGAGTTCTTTCATTAGCTTGGGCTACTGCTCAGC

TTGGATGGATTGCTGGACCTTCTGTTTTGCTACTCTTCTCTGCTGTTACTTACTACACTTGTAGTTTTCTGTCTGATTGT

TATCGTACAGGAGATCAACTTACTGGAAGAAGAAACTATACTTATATGGATGTTGTTCGAAGCAATCTTGGTGATGTTCA

CGTTAAGATTTGTGGGGTTATTCAATACGTAAATCTTGTTGGAGTTGCAATTGGTTACACAATTGCATCTTCTATTAGCA

TGGTTGCAGTAAAAAGATCTAACTGTTTCCACAACAATGGTCATGATCACGCTTCTTGCAATATTTCAAGCACACCCTAC

AAGATCATGTTTGGAGTCTTGGAAATTGTCTTGTCACAAATCCTAAATTTTGATCAGATTTCGTGGCTTTCTATTGCCGC

AGCTCTTATGTCCTTCACTTACAAGACCATTGGGCTTGGCTTAGGAGTTGCCAAAGTTGTACAAACAGGAAAAATACAAG

GGAGTATAAGTACTGGAACTGAAATCGACAAGGTATGGAAAAGCTTTCAAGCTCTTGGAGCTATAGCTTCTGCTTATTCT

TACTCCCTCATCCTTATTGAGATTCAGGATACACTGATCAAATCACCATCAGCAGAAGCAAAGACGATGAAGAAGGCAAC

ACAAATAAGTCTGGTAGTAACAACTGTTCTCTACATGCTCTGTGGCTGCTTTGGCTATGCAGCATTTGGAGACCAATCCC

CAGAAAACCTACTGACTGGATTTGGATTTGACGATCCATATTGGCTACTGGACATCGCAAACGTTGCCGTCGTTATCCAC

CTAGTAGGTGCATATCAAGTTTCCTGCCAAACCCTTTTTGCTTTTGTTGAAAAAAAAGCAGGTGAATGGTACCCAGACAG

CAACATCATCACGACAGAGATCGATATCCCAATCCATCGCTGTAAGCCCTTCAAACTCAACTTTTTCCATCTAATTTGGA

GGACTATTTTTGTGATCGCCACCACTTTCATTTCTGTGCTGATGCCATCCTTTAACAACATCGTTGACATTCTTGGAGCG

TTTGCATTTTGGCCACTTACAGTATACTTCCCAATAAAAATGTACATTGTCAAAAAAAATATTCCCAAATGGAGTGGTAG

GTGGATTTGCCTTCAGTTGCTTAGTGGAGCTTGCCTTGTTATCTCAATTGCTGTAGCTGTTGGTTCTTTTGCTGGACTTG

TTTCTGATTTAAAAGCTTTCAAGCCTTTCAAGTAG

>StAAP3

ATGACAGATCATGTTTCTGTTACTGTGTATGATGAATCGAGTTCTTTTGATGACGATGGTCGTTTGAAAAGAAGAGGGAA

TGTTTGGACTGCAAGTGCTCATATAATTACTGCTGTGATTGATTCTGGAGTTTTAATAGTTGCTTGGGCTACTGCACAGC

TTGGATGGATTGCTGGTCCAGTTATATTGCTTCTCTTCTCCATTGTTACTTACTACACTTCTAATTTGCTCTCTGATTGT

TATCGTAAAGGTGATCAACTTACTGGGAAAAGAAACTATACGTATATGGATGCTGTTCGAGCCAATCTAGGTGGTGTTCA

TGTTAAGATTTGTGGGATACTTCAATATGCAAATATTGTTGGAGTCGCGGTTGGGTATACTATCGCGTCTTCTATTAGTA

TGGTAGCTGTGAAAAGGTCAAATTGTTTCCATGAATACGGTCATCAGGCTGCTTGTAATGTTTCAACAACTCCATACATG

ATCGCGTTTGGAGTGGTGCAGATTGTCTTGTCACAAATCCCAGATTTTGATCAGATTTCGTGGCTTTCTATTGTGGCTGC

TGTTATGTCTATGACATACTCAACGATTGGTCTTGGTTTGGGAGTTGCTAAAGTTGCTGAAACAGGGAAAGTACAAGGAA

GTTTAACTGGGGTTAGTGGTGGAACTGGAATGCAGAAGATTTGGAAAAGCTCTCAAGCTGTTGGAGCTATAGCTTTCGCG

TATTCTTACTCTCTCATACTTATTGAGATTCAGGATACAATCAAATCACCACCATCAGAAGCTAAGACGATGAAGAAGGC

ATCGTTGATAAGTGTGACAGTAACAACTGTTTTCTTCATACTCTGTGGTTGCTTTGGTTATGCAGCATTTGGAGATCAAT

CCCCTGGAAGTTTACTAACTGGATTTGGATTCTACGATCCTTATTGGCTTCTCAACATCGCAAACATGGCTGTCGTTGTA

CACCTAGTGGGTGCTTACCAAGTTTACTGCCAACCCCTTTTCGCCTATGTTGAAAAAACAGCAGCTGAACGGTACCCTGA

CAGTATAATCATCATGAAGGAGATTGATATTCCGATCCCTGGCTGTAAGCCCTTCAAGCTCAACTTTTTCCGTCTAGTAT

GGAGGACAGTTTTCGTGATCTTCACAGTTCTCATTTCTATGTTGATGCCATTCTTTAACGATATTGTTGGCATTCTTGGA

GCCTTTGGATTTTGGCCACTTACAGTCTACTTCCCAGTAAAAATGTACATTGTGGAAAAGAATATTACCAAATGGAGTGG

AAGATGGATCTGCCTTCAACTACTTAGTGGTGCTTGCCTTGTTATCTCGATTGCTGCAGCTGCTGGTTCTTTTGCTGGAC

TTGTTTCTGATTTACAAGTTTCCTGGCCTTTCAAGACGCTTAATTAA

>StAAP4

ATGACGATGGCAGACAAAGCACATCAAGTATTTGAGGTCTATGGTGAATCGAAATGTTTTGATGACGATGGTCGAATCAA

AAGGACTGGGTCTGTTTGGACGGCAAGTGCTCACATCATAACTGCTGTGATAGGATCAGGAGTTTTATCATTGGCTTGGG

CTACTGCTCAGCTTGGATGGGTTGCTGGTCCAACCGTATTGCTTCTATTCTCCTTTGTTACTTACTATACCTCTGCTTTG

CTTTCCGATTGTTACCGGACCGGTGATCCAGTTACCGGAAAAAGAAATTATACTTATATGGATGCTGTTCGAGCCAATCT

TGGTGGATTTCAAGTTAAGATTTGTGGTGTAATTCAATATGCAAATCTTTTTGGAGTTGCAATTGGTTATACCATCGCGG

CTTCCATTAGCATGGTGGCTGTGAATAGATCTAATTGTTTCCATAAACAAGGTCATCGTGCTGCTTGCAATGTTTCAAGC

ACTCCCTACATGATCATTTTTGGAGTAATGGAAATTATCTTCTCACAAATCCCAGATTTCGATCAGATTTCTTGGCTTTC

TATTGTTGCTGCTGTTATGTCCTTTACTTACTCTACAATTGGTCTTGGTTTAGGAGTTGCTCAAGTTGCAGAAACAGGAA

AAATTGAAGGAAGTTTAACTGGGATTAGCATTGGAACTGAAGTAACTGAAATGCAGAAGATTTGGAGAAGCTTTCAAGCT

CTTGGAGCTATAGCTTTTGCTTATTCTTACTCCCTAATCCTTATCGAAATTCAGGATACACTCAAATCACCACCAGCAGA

AGCAAAGACAATGAAAAGGGCAACACTAATTAGTGTGGCAGTAACAACAGTTTTCTACATGCTTTGTGGTTGCTTTGGGT

ATGCAGCATTTGGAGATCAATCCCCTGGAAACCTACTAACCGGATTCGGATTCTACAACCCCTATTGGCTTCTTGACATC

GCAAATGTAGCCATCGTTGTTCACCTAGTAGGTGCCTACCAAGTTTACTGCCAACCCCTTTTTGCCTTTGTTGAAAAAAC

AGCAACTGAATGGTACCCTGACAGCAAAATCATTACAAAAGAGATCGATGTACCAATCCCGGGATTTAAGCCTTTCAAGC

TCAACCTTTTCCGTCTAGTCTGGAGGACAATATTCGTGATCATCACCACAGTTATATCAATGTTAATGCCATTCTTTAAC

GACGTCGTTGGCATTCTTGGAGCTTTTGGATTTTGGCCACTTACAGTATACTTCCCAGTAGAAATGTACATTGTGCAAAA

GAGAATTACCAAATGGAGTGGAAGATGGATATGTCTTCAAATACTTAGTGGTGCTTGCCTTGTTATCTCAATTGCTGCAG

CTGCTGGTTCTTTTGCTGGAGTTGCTTCTGATTTAAAAGTTTACAGGCCTTTTCAGAGTTAA

>StAAP5

ATGGGGTTGGATGAAGAAAGTGACTCTCAAATGCCATTTTTGGACCCAAATTATGTTTCTTCTTCTTCTTCTTCTTCCCA

CTCATCTAAGCTTGCCCTCAAAAGAACAGGAAATGAATGGACAGCTTTGGCACATATAATTACAGCAGTAATTGGTTCTG

GTGTTCTATCACTAGCATGGAGCATGGCACAGCTAGGTTGGATTGCTGGTCCATTGACTATGCTCGTATTTGCTTGTGTC

TCTCTTACTTCTGTATTTCTTCTCTGCAATTGCTATAAGTCTCCTGATCCAGAAATTGGCCCTGATAGGAATGGCTGTTA

TCTTGATGCTGTACAGAAGATTTTAGGAAAAAGAAATGCCTGGTTTTGTGGAATCGCTGTTCGTATAAATTTCATCAAGG

TTGCAATTATATATACGATTACATCCGCCTCCAGTATGCAAGCTATCCAGAAATCAAACTGCTATCATGATCAAGGTCAC

AAGGCTACCTGTGGATATGAAAGTACTAGGTATATGGTAATATTTGGATTAATCCAAGTTATAGTGTCTCAAATTCCTGA

TTTTCCGAATATGAAGTGGCTCTCCGTAGTTGCTGCTGTCATGTCCTTCACATATGCAATTATTGGATCAGCACTTGGCT

TAGCCAAAGTAATAGAAAATGGAGAAATCAAAGGCAGCATTACAGGACTGCCAAGTTCTACAGCTGCTGAGAAAGTGTGG

TTGGTTGCTCAAGCACTCGGGAACATTGCCTTTGCCTTTCCATTCTCTCTTATATTTTTGGAGGTTCAGGACACATTAAA

GGCACCTCCTCCAGAAAAGATCACCATGAAGAAGGTCTCAATAATGGCATCCTGTGTTACAACTTTTTTCTACCTTTGCT

GTGGAGGATTTGGTTATGCAGCCTTTGGTAATTCCACACCAGGGAACCTCTTGACAGGATTCGGTTTCTATGAACCATAT

TGGCTTGTAGACTTTGCTAATGCATGTGTTATTCTTCATCTTGTTGGTGGATATCAGGTTTTCAGCCAGCCAATATTTGC

AGAAGTTGAGAGATGGTTTGCCAGAAAATTTCCAGATAGCAAGTTCGTTCACAAGAATCACACTCTGAAACCTCTATCAA

TGCTGTCATTTAGTTTGAATTTTATGAGATTATTCTTCCGAACAGCGTATGTTGCAATAATGACTGGGATTGCTGTATTG

TTCCCTTACTTTAACCAGGTCGTAGGGGTGTCCGGAGCAATAACATTTTGGCCTATCGTCGTTTATTTCCCTGCAGAGAT

GTACTTGACTCAGAAAAGAATTGAAAGTTGGAAAAGCAAAGCTATCGCGTTTCGCGTCTTTACAATGGTATGTCTGGTTG

TGATATTGTACGCATTTGTTGGGTCTATTAGAGGAGTGATTGTTGCTAGGTTCGGGTAA

>StAAP6

ATGGAAGCTCAACGTTCTCTAGAACAAGAGAAAACTTCAGGCAAAGAGGATGAAGATACAATTAGAACAGGAACTTTGTG

GACTGCAGTTGCGCACATAATTTCAGCTGTAATCGGAGCTGGTGTTCTCTCTCTAGCCTGGTGCACAGCACAATTAGGAT

GGATTGCAGGGCCAATCACCATGCTTTGTTTTGCAGTTGTTACATACATATCTGCATCTCTCATATGTGATTGTTACAGG

TCACCTGATCCCATAACAGGAACGCGAAATCCCTCTTACATTGATGCCGTTAGAGTTAATCTTGGCAAGAAATGGACGTG

GTTGTGTGGTTTGCTTCAGTATGTGAGCTTCTATGGGACTGGTATTGCTTATGTAATTACAAGTGCAACAAGTATGAGAG

AAATTCAGAGATCAAATTGTTACCACAAAGGAGGAGAAGAAGCTGTTTGCCAGACTGGGACAAACAACTTCATGCTGATT

TTTGGCATTATTCAGATAGTAACGTCGCAGATACCAAATTTTCATAACATGGCGTGGTTGTCCGTTGTTGCTGCGTTAAT

GTCATTTTGCTACTCTTTCATCGGATTGGGCCTTGGATTTTCTAAAGTGATTGAAAATAGGGGTATCAAGGGAAGCATTG

TGGGAGTACCAACAAGGAGTGCTGCTCAGAAGATTTGGTTAGTTTTCCAGGCACTCGGAGACATTGCTTTTGCCTATCCT

TATTCGATTATTCTCCTCGAGATACAGGATACACTAAAATCGCCTCCACCAGAAAACCAGACCATGAAGAAGGCGTCAAT

AAGCGCAATAGTTATCACCACTTTCTTCTATCTATGCTGTAGTTGCTTTGGATATGCAGCCTTTGGGAACGACACACCAG

GGAACCTCTTGACAGGTTTCTACGAGCCGTTTTGGCTTGTTGATTTTGCCAATGCCTGCATAGTCCTTCATCTGGTTGGA

GGGTATCAGGTATACAGCCAGCCAGTATTCGCATTTGTTGAAAAATGGGCTACTCAGAAGTACCCTGAGAGCAGATTCAT

AAATAAATTCTACGCCATCAAACTCCAGGTGTTGCCAGCTCTCCAGTTGAACCTCTTCCGGTTATGTTTTCGAACTCTAT

ATGTTATATCTACAACCGCAATAGCAATGGCATTTCCATATTTCAACCAGGTATTAGGAATCTTAGGAGCATTGAACTTT

TGGCCTATGACCATATATTTTCCAGTGGAAATGTACATTGTGCAAAGAAAAATTGGAGCTTGGACAAGAAAATGGATACT

TCTTGAAGGTTTCAGTATGGTTTGCTTGATTGTATCTTTACTGGGCTTGATTGGATCAATTGAGGGAATAGTTAGTGCTA

AATTAGCTTAA

>StAAP7

ATGGGAGATTCTACCAATTTTGCATCAAAACATCAACTTTTTGATGTTTCTGTGAATGTAACTGAATCCAAGCGTTTTGA

CGACGATGGACGTATCAAAAGAACTGGGAGTGTTTGGACTGCAAGTGCTCATATCATAACTGCGGTGATTGGTTCAGGCG

TTTTATCTTTGGCTTGGGCTGTAGCTCAACTTGGTTGGATTGCTGGTCCTATTGTTATGCTTTTATTCTCTTTTGTTACT

TATTACACCTCTTCTCTGCTTTCCGATTGTTACCGCTCCGGTGACCCACTTTCCGGCAAGAGAAATTATACTTACATGGA

TGCTGTACAAGCAAATCTCGGTGGCTTACAGGTAAAGATTTGTGGATGGATTCAGTATGTGAATCTGTTTGGAGTTGCTA

TTGGATACACAATTGCTTCTTCAATTAGCATGATGGCTGTTAAAAGGTCAGATTGTTTTCATAAACATGGTCATAAAGCA

CCTTGTTTAGAACCAAATACTCCATATATGATCATATTTGGAGTAATCGAAATCGTCTTCTCACAAATACCAGATTTTGA

TCAAATTTGGTGGCTTTCAATTGTTGCTGCTGTAATGTCTTTCACTTACTCGACAATCGGTTTAGGTTTAGGCATTGCTC

AAGTAGCAGAAACTCGAAAAATTGGAGGAAGTTTAACTGGAGTTAGCATTGGAACTGTGACTGAAATGCAAAAAGTTTGG

AGAACTTTTCAAGCACTTGGAGCTATTGCTTTTGCCTATTCTTACTCCCTCATCCTTATCGAGATTCAGGATACAATCAA

ATCCCCACCTTCAGAAGCCAAGACAATGAAAAATGCAACTCTAATTAGTGTATCAGTAACAACAGTTTTCTACATGCTCT

GTGGCTGTTTTGGCTATGCAGCATTTGGAGACCATGCTCCTGACAATTTACTAACTGGTTTTGGATTCTACGACCCGTAT

TGGCTACTCGATATAGCCAACATAGCCATCGTCGTTCATCTTGTAGGTGCATACCAGGTTTACTGCCAACCCCTTTTCGC

CTTCATTGAAAAAACAGCAGCAGAATGGTACCCTAACAGTAAATTCATCACCAAGAATATTAGTGTCCCAATCCCGGGCT

ATAAATCGTACAACCTCAACCTATTCAGGCTAGTTTGGAGGACGATCTTCGTTATCATCTCCACTTTCATCTCTATGCTG

TTGCCATTCTTCAGCGACATCGTTGGAATACTTGGAGCATTTGGATTTTGGCCTTTGACTGTTTATTATCCAGTGGAAAT

GTACATTGCACAAAAGAAGATACCAAAATGGAGTAGAAAATGGGTTGGTCTTCAAATTCTGAGTGTTACTTGCCTTATTG

TCTCAATTGCTGCAGCTGCTGGTTCTTTTGCTGGTGTTGTATCTGATCTTAAAGTTTACAAGCCTTTCAAATTTACTTAG

>StAAP8

ATGGCATCAGAATTTGAGAAGAATAGTACCAATATGTATGTAGAACAATCACCAAAAGGACTAGAAAATGGACAAGTTCA

GAAAAATGTTGATGATGATGGACGCGAAAAAAGAACAGGGACCGTGCTAACGGCGAGTGCTCATATAATAACTGCAGTGA

TTGGGTCAGGAGTGTTGTCACTAGCATGGGCAATGGCTCAGCTAGGGTGGGTGGCTGGTCCTGTTATTCTCTTCCTCTTT

TCTTTCATCACCTATTTTACTTCAACATTGCTCGCCGATTGTTATCGATTTCCGGGCCCCGGCTCCGGCAAGAGAAACTA

TAGTTACATGGAGGTTGTCCGCTCTCACTTAGGAGGATTTAAAGTGCAACTTTGTGGAATAGCTCAATATGGTAATTTAG

TAGGAATTACAATTGGATACACAATTACTGCATCTATTAGTATGAAGGCAGTAGTAAGGTCAAATTGTTTTCACAAGGAG

GGGCACCAAGCTAGTTGCACAGTATCAAATTACCCATATATGGTTATTTTTGCAATAATACAAATAATTCTAAGCCAAAT

ACAAAATTTCCATAAACTTTCATGGCTATCAATTCTTGCAGCAGTTATGTCTTTTGCTTACTCTCTTATTGGTCTTGGAC

TTTCCATAGCCAAAGTTGCTGGTGCTGGGCATCATGTGAAGACTAGCCTAACAGGGACAATAGTTGGTGTAGACGTGTCA

GGATCTCAGAAAGTTTGGAGATGTTTACAATCTATTGGAGATATTGCCTTTGCTTATGCTTTTGCCACCATTCTCATTGA

AATACAGGACACATTGAGATCACCACCAGCAGAGAACAAAGTTATGAAGAGAGCATCACTTGTTGGAGTTTTTACCACAA

CTTTATTTTATGTGTTATGTGGTACAATTGGCTATGCAGCCTTTGGCAACAATGCTCCTGGAAATTTTCTCACTGGTTTT

GGTTTCTATGAACCCTTTTGGCTAATCGATTTCGCTAACGTTTGCATCGCCATCCACCTCATCGGAGCTTACCAGGTATT

TTGCCAACCAATATATGGCTTTGTGGAGGGTCGTTGTAGTGAAAAATGGCCAGACAACAAATTCATTAAATCCCAACATG

ACATAAACATCCCATGGTTTGGTGTTTACAATCTTAACTATTTTAGGATGCTGTGGAGGACAATATATGTTATAATAACA

GCAATAATTGCTATGATTTTCCCATTTTTCAATGCAATATTAGGCTTAATTGGGGCAGCTTCATTTTATCCATTAACTGT

TTATTTCCCAATAGAAATGCACATTGCACAAAGGAAAATACCTAAATATTCATTCAAATGGATATGGTTACATATATTGA

GTTGGGCTTGCTTGATTGTGTCACTTGTTGCTGCTGTTGGATCCATAGAAGGCCTTACACAAGATCTCAAGACATATAAG

CCCTTCAAACCTCAAGATCATGATTGA

>ZmAAAP09

ATGGCGGTGCACCACGCGCTCGAGGTGCTCGACGGCCGCTGCGACGACGACGGCCACCCGCGCAGGACTGGGACTGCGTGGACGTGCGCGGCGCACATCA

TCACGGCGGTGATCGGCTCCGGGGTGCTGTCCCTGGCCTGGAGCGTGGCGCAGCTGGGCTGGGTGGTCGGCCCGGCCTGCATGTTCTGCTTCGCGCTCGT

CACCTACGTCTCCGCGGCGCTGCTCGCCGACTGCTACCGGCGCGGTGACCCCGGCAACGGGCCACGGAACCGCTCCTATATGGACGCCGTCCGTGTCTAC

CTCGGCAAGAAGCATACCTGGGCGTGCGGCTCACTGCAGTACGTGAGCATGTACGGCTGCGGCGTCGCCTATACCATCACCACCGCCACTAGCATAAGGG

CGATTCTGAAGGCGAACTGCTACCACGAGCATGGACACGGCGCGCACTGCGAGTACGGTGGCAGCTACTACATGCTCATCTTCGGCGGCGCCCAGCTCCT

CCTCTCCTTCATACCGGAATTCCACGACATGGCGTGGCTCTCCATCGTCGCCGCGGTCATGTCATTCTCGTACTCCTTCATCGGCATTGGCCTCGGCCTC

GCAACAACCATTGCTAACGGGACGATCAAAGGAAGCATAACAGGTGTTCGGATGAGGACGCCTATGCAGAAGGTTTGGCGCGTCTCGCAGGCCGTCGGCG

ACATCGCCTTCTCGTACCCCTACTCCTTGATCCTCCTGGAAATACAGGACACCCTGAAGTCACCGCCGGCCGAGAACAAGACGATGAAGAGGGCATCGAT

TGGCTCGATCCTCGTCACGACGTTCTTCTACCTCTGCTGCGGCTGCTTCGGCTACGCCGCCTTCGGGAGCGACTCGCCGGGCAACCTCCTCACCGGCTTC

GGCTTCTACGAGCCGTACTGGCTCATCGACTTCGCCAACGCCTGCATCATCCTCCACCTGCTGGGCGGGTACCAGGTGTACAGCCAGCCCATCTTCCAGT

TCGCGGACCGGTTCTTCGCGGAGCGGTTCCCCGACAGCGGGTTCGTGAACGACTTCCACACCGTCCGGGTCGCGTGCCTGCCGGCGTGCCGGGTGAACCT

GCTGCGCGTGTGCTTCCGGGCGCTGTACGTGGCGTCCACGACGGCGGTGGCCGTGGCGTTCCCCTACTTCAACGAGGTGCTGGCGCTGCTGGGCGCGCTC

AACTTCTGGCCGCTGGCCATCTACTTCCCCGTGGAGATGTACTTCGTCCAGCGGAACGTGCCCCGGTGGTCCACACGCTGGGTCGTCCTGCAGACCTTCA

GCGTCGTCTGCCTGCTCGTCAGCACCTTCGCGCTCGTCGGCTCCATCGAGGGGCTAATCACCCAGAAGCTAGGCTAG

>ZmAAAP14

ATGGCTGCGAAGGCAACAGGCCACGTGGGCACGGAGGCGATGGAGGTATCCGTGGAGGTGGCGAACGGCGACGACGACGCGGCACGGCTAGACGACGATG

GGCGGCCGCGGCGGAGGGGCACCATGTGGACGGCGAGCGCGCACATAATCACTGCGGTCATCGGCGCCGGCGTCCTGTCGCTGGCGTGGGCAATGGCGCA

GCTTGGCTGGGCGGCGGGGACCGCCATGATGCTGCTCTTCGCCGGCATCAGCTACTACACCTCCACGCTGCTCGCCGAGTGCTACCGCTGCGGCGAGCCG

GGCACCGGGAAGCGCAACTACACGTACACGGAGGCCGTGCGCGCCATCCTCGGCGGTGCCAAGTTCAAGCTCTGCGGCGTCATCCAGTACGCCAACCTCG

TCGGCATCGCCGTCGGATACACCATCGCCGCCTCAATCAGCATGCTGGCGATCAAGAGGGCGGATTGCTTCCACGACAGAGGGCACAGGAACCCGTGCCG

CAGCTCCAGCAACCCCTACATGATCCTATTCGGCGCCGTCGAGATCGTCTTCTCACAGATACCGGACTTTGACCAGATATGGTGGCTCTCCATCGTCGCC

GCCGCCATGTCCTTCACCTACGCCACCATCGGCCTCGCACTCGGCATCGCGCAGACCGTCGCCAACGGCGGGTTCAAGGGCAGCCTCACCGGCGTCAATG

TCGGCGACGGGATCACGCCGATGCAGAAGGTCTGGCGCAGCCTGCAGGCCTTCGGCAACATCTCCTTCGCCTACTCCTACGCCTACATCCTCATCGAGAT

ACAGGACACGATCAAGGCGCCGCCGCCATCGGAGGTGACGGTGATGAAGAAGGCCACGATGGTGAGCGTGGCGACGACGACGGTGTTCTACATGCTGTGC

GGCTGCATGGGCTACGCGGCGTTCGGCGACGACGCCCCGGACAACCTCCTGACTGGGTTCGGCTTCTACGAGCCCTTCTGGCTCCTGGACGTCGCCAACG

CCGCCATCGTCGTGCACCTCGTTGGCGCGTACCAGGTCTTCTGCCAGCCGCTCTTCGCCTTCGTGGAGAAGCGGGCGGCAGCCAGGTGGCCCGACAGCCG

GTTCATGACCCGGGAGCTGAGGCTTGGACCCTTCGTGCTCGGCGTGTTCCGTCTGACCTGGCGGACGGCCTTCGTCTGCCTCACCACCGTCGTCGCCATG

ATGCTCCCTTTCTTCGGCGACGTCGTGGGGCTGCTCGGCGCCGTCTCCTTCTGGCCGCTCAGCGTCTACTTCCCCGTCGAGATGTACAAAGCGCAGCGTC

GCGTGCGCAGGTGGAGCACGCGCTGGCTCTGCCTCCAGACGCTCAGCGCCGTGTGCCTCCTCGTCTCCATCGCCGGCGCCGTTGGCTCCACGGCCGGCGT

CATAAACGCAGTCAATTTGCACCGACCATTCAGTGGCTAA

>ZmAAAP17

ATGAGCGACGACAGGAGAACGGTAGCGTACGATGCTGAAGCCGGCGACGGTCATGAGAGGCAAGCAGGGACGGTGTGGACGGCGACGTCGCACATTGTCG

CCGTGGTGGCGGGCTCCGGCGTGCTGGCGCTGCCGTGGACGGTGGCGCAGTTGGGGTGGGTGCTGGGCCCCCTCGTCCTGGTGGGCTTCTCCTGCGTCAC

CTACTACACCTCCGCGCTCCTCGCCGACTGCTACCGCTACCCGGACCCCGTCCACGGCGCCGTCGTCAACCGCCAGTACGTCGACGCTGTCCGCTGCTAC

CTGGTACGTGAACCTATGGGCCACTCTCGTCGGCTACACCATCACCGCCAGCGCCAGCATGATGCCATGAAGCGGGCCTCTTTCTACGGCCTCGGCGCCG

CCACTGCCTTCTACCTCGCGCTGGGCTGCGCCGGCTACGCCGCCTTCGGGGACGACGCGCCGGGGAACGTCCTCACGGGCTTCGCCTTCCACGAGCCCTC

CTGGCTCGTCGACGCCGCCAACGCCTGCGTCGTCGTACACCTCGTCGGCGCCTACCAGGTGTTCGCGCAGCCCATCTTCGCGAGGCTCGAGAGCTGCGCC

GCGTGCCGCTGGCCCGACGCCAAACTCGTCAACGCCACCTACTACGTGCGCGTGCCGCCGTTCCTCCTGCGCTCCGCGTCGTCGCCGCCCACCGTGGCCG

TAGCGCCGCTCAAGCTCGTGCTGCGCACCATCGTCATCATGTTCACGACGCTCGTGGCCATGCTGCTGCCCTTCTTCAACGCCGTGCTGGGCCTCATCGG

CGCGCTGGGATTCTGGCCGCTCTCCGTCTACTTCCCTGTCAGCATGCACATGGCCAGGCTCAACATCCGCCGCGGCGAGCTCCGGTGGTGGGCGCTGCAG

GCCATGAGCTTCGTCTGCCTCCTCGTCTCCATCGGCGCCAGCATCGGCTCCGTGCAGGACATCGTGCACAACCTCAAGGCCGCCGTGCCATTCAAGACCG

TAAATTGA

>ZmAAAP18

ATGAGGAGGCAGAGCTCGCTAGCTCGCTCGTGCTCCTCAGTGGCACCGGCGTCGCCGCCGCCTCAAAACGGAGGAGTGAACAGCAAGCACCTGGTCCCCC

CCATGGAGGTGTCGGCGGAGGCCGGGAACGCCGGTGCCGCCGAGTGGCTAGACGACGACGGCCGCCCCCGCCGCAAGGGCACGTTCTGGACGGCCAGCGC

GCACATCATCACCGCCGTCATCGGCTCCGGCGTGCTGTCCCTGGCCTGGGCCATCGCGCAGCTGGGCTGGGTCGCCGGCCCCACCGCCATGCTCCTCTTC

GCCTTCGTCACCTACTACACCGCCACGCTGCTCGCCGAGTGCTACCGCACCGGCGACCCCGACACGGGCAAGCGCAACTACACGTACATGGACGCCGTGC

GCTCCAACCTCGGCGGCGCCAAGGTCGCCTTCTGCGGCGTCATCCAGTACGCCAACCTCGTCGGCGTCGCCATCGGGTACACCATCGCGTCGTCCATCAG

CATGAAGGCCATCAGGAGGGCGGGCTGCTTCCACACCCACGGCCACGGCGACCCCTGCAAGAGCTCCAGCACGCCATACATGATCCTCTTCGGCGCTGCG

CAGGTCGTCTTCTCCCAGATACCGGACTTCGATCAGATATGGTGGCTCTCCATTGTCGCTGCAGTCATGTCCTTCACTTACTCGTCTATCGGACTGTCCC

TCGGAATCGTACAGACCGTCTCCAACGGTGGGTTCAAGGGCAGTCTCACCAGCATCGGCTTCGGCGCCGGCGTGAACTCCACGCAGAAGGTCTGGCACAC

GCTGCAGGCCTTCGGCGACATCGCGTTCGCCTACTCCTTCTCCAACATCCTCATCGAGATCCAGGACACGATCAAGGCGCCGCCACCGTCGGAGTCGAAG

GTGATGCAGAAGGCGACGCGCCTCAGCGTGGCGACGACGACCGTGTTCTACATGCTGTGCGGGTGCATGGGGTACGCGGCGTTCGGCGACGACGCGCCCG

ACAACCTGCTCACCGGCTTCGGCTTCTACGAGCCCTTCTGGCTGCTGGACGTGGCCAACGTGGCCATCGTGGTGCACCTCGTGGGCGCGTACCAGGTGTT

CTGCCAGCCCATCTTCGCCTTCGTCGAGCGCCGCGCCGCCGCGGCCTGGCCGGACAGCGCCTTCGTCTCGCGGGAGCTCCGCGCCGGCCCGTTCGCCCTC

AGCCCTTTCCGCCTCGCGTGGCGGTCGGCGTTCGTGTGCGTCACCACCGTCGTCGCCATGCTGCTGCCTTTCTTCGGCGACGTGGCGGGGCTCCTCGGTG

CCGTCTCCTTCTGGCCGCTCACCGTCTACTTCCCCGTCGAGATGTACATCAAGCAGCGCCGCGTGCCTCGTGGCAGCGCCAGATGGATCAGCCTCCAGAC

GCTCAGCGTCACGTGCCTCCTCGTCTCCATCGCCGCCGCCGCCGGTTCCATCGCCGACGTCGTCGACGCGCTCAAGGTGTACCGGCCGTTCAGCGGCTAA

>ZmAAAP21

ATGGCGGAGAACAACGTCGTGGCCACGTACTACTACCCGACGGCAGCGCCGGCGGCCATGGAGGTCTGCGGCGCGGAGCTCGGCCAGGGCAAGCCCGACA

AGTGCTTCGACGACGATGGCCGCCCCAAGCGCAATGGGACGATGTGGACGGCGAGCGCGCACATCATCACGGCGGTGATCGGCTCCGGGGTGCTCTCGCT

GGGGTGGGCCATCGCGCAGCTCGGCTGGGTGGCCGGACCCGTCGTCATGCTGCTCTTCTCGCTCGTCACCTACTACACCTCGTCGCTGCTCGCAGACTGC

TACCGCTCCGGCGACCCCAGCACCGGCAAGCGGAACTACACCTACATGGACGCCGTCAACGCGAACCTCAGTGGCATCAAGGTCCAGATCTGCGGGTTCC

TGCAGTACGCCAACATCGTGGGCGTGGCCATCGGCTACACCATCGCTGCCTCCATTAGCATGCTCGCGATCAGGAGGGCCAACTGCTTCCACCAGAAGGG

ACACGGCAACCCCTGCAAGATCTCCAGCACGCCCTACATGATCATCTTCGGCGTGGCGGAGATCTTCTTCTCGCAGATCCCGGACTTCGACCAGATCTCC

TGGCTCTCCATCCTCGCCGCCGTCATGTCCTTCACCTACTCCTCCATTGGGCTCGGCCTGGGCGTCGTCCAAGTCATCGCGAACAGAGGCGTGCAGGGCA

GCCTGACCGGCATCACCATCGGCGTGGTGACCCCGATGGACAAGGTGTGGCGCAGCCTCCAGGCGTTCGGCGACGTCGCCTTCGCCTACTCCTACTCCCT

CATCCTGATCGAGATCCAGGACACCATCCGGGCGCCGCCGCCGTCGGAGTCGACGGTGATGAAGCGCGCCACGGTGGTGAGCGTGGCGGTCACCACGCTC

TTCTACATGCTGTGCGGCTGCATGGGGTACGCGGCGTTCGGCGACGGCGCGCCCGGGAACCTCCTCACGGGCTTCGGCTTCTACGAGCCCTTCTGGCTCC

TGGACGTGGCCAACGCCGCCATCGTGGTCCACCTGGTCGGCGCCTACCAGGTCTACTGCCAGCCGCTGTTCGCCTTCGTGGAGAAGTGGGCCGCGCAGCG

GTGGCCGGACTCGGCGTACATCACCGGGGAGGTCGAGGTCCCGCTCCCGCTCCCGGCGAGCCGGCGGCGGTGCTGCAAGGTGAACCTGTTCCGGGCGACG

TGGCGGACGGCGTTCGTCGTGGCCACGACGGTCGTGTCCATGCTGCTGCCCTTCTTCAACGACGTGGTGGGCTTCCTGGGCGCGCTCGGCTTCTGGCCGC

TCACCGTCTACTTCCCCGTCGAGATGTACGTGGTGCAGAAGAAGGTGCCGCGGTGGAGCTCCCGGTGGGTGTGCCTGCAGATGCTCAGCCTCGGCTGCCT

CGTCATCTCCATCGCCGCCGCAGCCGGGTCCATCGCCGGCATCGCGTCCGACCTCAAAGTCTACCGCCCGTTCAAGTCCTACTGA

>ZmAAAP22

ATGGACAAGAGCGCGGTGGCGGCATACGACGTCGAGCGGGGCGACTACGAGGAGGAGCACGAACGGAGAGGGACGGTATGGACGGCGACGGCGCACATTG

TGACGGCGGTGATCGGCTCCGGCGTGCTGGCGCTGGCCTGGAGCGTGGCGCAGCTGGGCTGGGTCGCGGGGACCCTCGCGCTCGCCGGCTTCGCCTGCGT

CACCTACTACACCTCCACGCTGCTCGCCAACGCCTACCGCGCGCCGCACCCCGTCACCGGCGACAGGAACCGCACCTACATGGACGCCGTCAGATCGTAC

CTCAGTCCCAGGGAGGTGTTCATGTGCGGGATCGCGCAGTACGTGAACCTGTGGGGCACCATGGTTGGGTACACCATCACGGCGACCATAAGCATGGCCG

CGATCAGGCAGTCCAACTGCTTCCGCCGGAGCGGCGCCGGCGCGCACTGCGACGCGCCGGGGACCGTGCTCATGCTGGCGTTCGGTGTGGTCCAGGTGGT

CCTGTCCCAGTTCCCCGGCCTGGAGCACATCACCTGGCTCTCCGTCGTCGCGGCGGTCATGTCGTTCGCCTACTCCTTCATCGGCCTCGGCCTCTCGGTG

GGGCAGTGGGTGTCTCACGGCGGCGGCCTCGGCGGCAGGATCGCTGGTGCTGCCGCGGCGTCCCCCACCAGAAAGCTCTGGAACGTGCTTCTTGCCCTGG

GGAACATTGCCTTCGCCTACACTTTCGCTGAAGTGCTAATTGAGATCCAGGACACGCTCAAGTCACCGCCACCGGAGAACAGGACGATGAAGAAGGCAGC

GATGTACGGGATTGGAGCCACTACCATCTTCTACATCTCTGTTGGCTGCGCTGGGTACGCTGCGTTTGGTTCAGATGCTCCGGGCAACATCCTGACGGCA

GGCGGGTTGGGTCCCTTCTGGCTCGTCGACATTGCCAACATGTGCCTCATCCTCCACCTGATCGGTGCATACCAGGTATATGCGCAGCCTATCTTTGCTT

CGGTTGAGAGGTGGGCTGCCTCCCGGTGGCCGGAGGCCAAGTTCATCAGCAGCGCGTACACCGTCAGCATCCCGCTCATGCAGAGAGGATCGGTGACCGT

GGCGCCTTACAAGCTCGTCCTAAGGACTGTTCTAGTCGCCGCGACGACTGTGGTGGCGCTGATGATACCCTTCTTCAACGCTGTGCTGGGGCTCCTCGGC

GCATTCAGCTTCTGGCCGCTCACGGTTTACTTCCCCATAAGCATGCACATTGCCCAGGACAAGATCACCAGGGGGACCAAGTGGTACCTTCTGCAGGCTT

TGAGCATGGTCTGTTTGATGATTTCAGTGGCCGTGGGTATAGGCTCTGTGACTGACATTGTCGATAGCCTCAAGGTCTCTTCCAACCCTTTAAAAACTGT

AAGCTAA

>ZmAAAP29

ATGGAGGTTGCCGGAAACCATGTTCAGAGCTGTCGCACGGAGCTGCCGGAGCCGCAGAAGCCGCTGGTGGACGACGACGGGCGGCCGCTCCGCACGGGCA

CGCTGTGGACGGCCAGCGCGCACATCATCACGGCGGTGATCGGCTCCGGCGTGCTGTCGCTCGCGTGGGGCGTGGCCCAGCTTGGGTGGGCGGGCGGCCC

CGCCGCGATGGTGCTCTTCGCCGCCGTCATCTACTACACGTCCACGCTGCTCGCCGAGTGCTACCGCTGCGGCGACCCCACGTTCGGCCCGCGCAACCGC

ACCTACATCGACGCCGTCCGCGCCACCCTCGGCGACTCCAAGGAGAGGCTCTGCGGCGCCATCCAGCTCTCCAACCTCTTCGGCATCGGCATCGGCGTCT

CCATTGCCGCCTCCGTCAGCATGCAGGCGATCCGGAGGGCCGGCTGCTTCCACTACCGAGGGCACGAGGACCCGTGCCACGCCTCCACCAGCCCATACAT

CGCCGTCTTCGGCGTCATGCAGATCGTCTTCTCGCAGATCCCCGACCTAGACAAGGTATGGTGGCTGTCCACCGTGGCCGCCATCATGTCCTTCTCCTAC

TCCACCATCGGCATCTTGCTCGGTGTTGTTCAGATTGTAGAACACGGAGGACCGAGGGGGAGCCTCGCCGGCGTCATCGGTGCTGGCGCCAGGGTCACCA

TGATGCAGAAGGTGTGGCGCAGCCTGCAGGCTTTTGGGAACATCGCGTTCGCGTACGGTTTCTCTATCATCCTGCTCGAGATACAGGACACGATAAAGTC

CCCGCCGCCGTCGGAGGCTAAGGTGATGAAGAAGGCGACGGCAGTGAGCGTGGCGGTGACCACGGTGATCTACCTGCTGTGCGGCTGCGTCGGGTACGCG

GCGTTCGGGGGCGCGGCGCCGGACAACCTGCTCACGGGCTTCGGCTTCTACGAGCCCTTCTGGCTGCTGGACGTGGCCAACGCGTTTGTGGTGGTGCACC

TGGTGGGCACCTACCAGGTCATGTCCCAGCCCGTCTTCGCCTACGTCGAGCGCCGGGCGGCCGCCGCGTGGCCTGGCAGCGCGCTGGTTCGCGACAGGCA

CGTGAGGGTGGGGCGAGCCGTGGCCTTCTCCGTCAGCCCGGCGCGGCTCGCGTGGCGCACGGCGTACGTGTGCGTCACCACGGCGGTGGCGATGCTGCTG

CCCTTCTTTGGCTCCGTTGTCGGTCTCATCGGCGCCGCCTCCTTCTGGCCGCTCACCGTCTACTTCCCCGTGGAGATGTACATCGCGCAGCACCGAGTGG

CGCGGGGCAGCATGCGGTGGCTGCTCTTGCAGGGGCTCAGCGCCGGGTGCCTTGTTGTGTCCGTTGCTGCCGCTGCCGGGTCCATCGCCGGCGTCGTGGA

AGACCTCAAGGCGCACAATCCCTTCTGCTGGTCGTGCTGA

>ZmAAAP33

ATGGTGGCGAGCAAGGCCGCCCCGTTCGACGAGGTGTCCTCGGTGGAGGCGGGGGCCTACGGGGGCCGCGACGACGACGGGCGGCCGCGCCGGACGGGCA

CGGTGTGGACGGCCAGCGCCCACATTATCACGGCCGTCATCGGCTCCGGGGTGCTCTCGCTGGCGTGGGCCATCGCGCAGCTGGGCTGGGCCGCGGGCCC

CGCCGTCATGCTGCTCTTCGCCGTCGTCATCTACTACACCTCCACGCTGCTCGCCGAGTGCTACCGCTCCGGCGACCCAGTGGCGGGGAAGCGCAACTAC

ACCTACATGGACGCCGTCCGCGCAAGCCTCGGCGGCGCCAAGGTCAGGCTCTGCGGCGCCATCCAGTACGCCAACCTCTTCGGCGTCGCAATCGGATACA

CAATCGCCGCATCCATCAGCATGCTCGCCATCAAGAGGGCGGACTGCTTCCACGCCAAGGGCCACAAGCACGCGTGCCGCAGCTCCAGCAACCCCTACAT

GATCCTCTTCGGCGTCGCCGAGGTCGTCTTCTCGCAGATACCGGACTTCGACCAGATTTGGTGGCTCTCCATCGTCGCCGCCGTCATGTCCTTCACCTAC

GCCACCATCGGCCTCGTCCTCGGCATCATGCAGACCGTCGCCAACGGGGGCTTCCAGGGCAGCCTCACCGGGATCAGCATCGGCGCCGGCGTCACCCCGA

CGGAGAAGGTCTGGCGGAGCCTGCAGGCCTTCGGCAACATCGCCTTCGCCTATTCCTACTCCATCATCCTCATCGAGATCCAGGACACGGTCAAGGCGCC

GCCGCCATCCGAGGCCAAGGTGATGAAGAGGGCGACCATGGTGAGCGTGGCCACCACAACGGTGTTCTACATGCTGTGCGGGTGCATGGGCTACGCCGCC

TTCGGCGACGCCGCGCCGGACAACCTCCTCACCGGCTTCGGCTTCTACGAGCCCTTCTGGCTGCTGGACATCGCCAACGTCGCCATCGTCGTGCACCTCG

TCGGCGCCTACCAGGTCTTCTGCCAGCCGCTCTTCGCCTTCGTCGAGAAGTGGGCAGCGGCCACGTGGCCCGACAGCGCCTTCATCGCCCGGGAGTTCCG

CGTGGGCCCCTTCGCGCTCAGCCTGTTCCGCCTCACCTGGCGCACGGCGTTCGTGTGCCTCACCACCGTCGCCGCCATGCTGCTCCCCTTCTTCGGCGAC

GTCGTCGGCCTGCTCGGCGCCGTCTCCTTCTGGCCGCTCACCGTCTACTTCCCCATCGAGATGTACGTCGTGCAGCGCGCCGTGCGCAGGTGGAGCACGC

ACTGGATCTGCCTCCAGATGCTCAGCGCTGCGTGCCTCCTCGTGTCCGTCGCCGCCGCAGCGGGCTCCATCGCCGACGTCATCGGCGCGCTCAAGGTGTA

CCGCCCGTTCAGCGGCTAG

>ZmAAAP36

ATGGGGCGGTCAGGAGGCGGCGACGGGGACGGCGACGGCGACCGGCTCCTCCTCGGCAAGCCCTTGGAGTCGTCGTCGTCGTGCAGCTCGTCCGACGAGA

GCCTGGTCAAGAGAACCGGCACGGTATGGACGGCGATGGCGCACATCATCACGGCGGTGATCGGGTCCGGCGTGCTGTCCCTGGCGTGGAGCGTGGCGCA

GCTGGGGTGGGTGGGCGGGCCTGCGGCGATGGTGTTCTTCGCCGGCGTCACCGCGGTGCAGTCCACCCTCATCGCCGACTGCTACATCTGCCACCACCCG

GAGCGAGGAGGCGTCGTCAGGAACCGCTCCTACGTCGACGCCGTGCGCATCTACCTGGGCGACAAGAGCCATTTGTTTTGCGGCTTCTTCCTCAACCTGA

GCTTGTTTGGCACCGGTGTGGTGTACACGCTCACTTCCGCCACTAGCATGAGGGCGATCCGGAAGGCCAATTGCTACCACAGGGAAGGCCACGACGCGCC

GTGCTCGGTGGGAGGAGACGGCTACTACATGCTGCTCTTCGGCCTCGCGCAGGTGCTGCTGTCGCAGATACCCAACTTCCACGAGATGGCGGGGCTCTCC

ATCTTCGCCGCCGTCATGTCCTGCTTCTACGCCTTCGTCGGCGTCGGCCTCGGCGTCGCCAAAGTCATCGCAAACGGGGTGATCATGGGCGGCATCGGAG

GCATCCCGCTGGTGTCCACGACGCAGAAGGTGTGGCGAGTCTCGCAGGCCCTCGGGGACATCTTGTTCGCCTACCCTTTCTCGTTGGTGCTGCTGGAAAT

AGAGGACACGCTGAGGTCGCCGCCGCCGGAGAGCGAGACGATGAAAAAGGCGACGAGAGCGAGCATCGCTATCACCACCCTCTTCTACCTCTGCTGCGGG

TGCTTTGGCTACGCGTCGTTCGGCGACGGCACCCCGGGCAACCTCCTCACCGGCTTCGGCTTCTACGAGCCCTACTGGCTCATCGACCTCGCCAACCTCG

CCATCGTTCTCCACCTCCTCGGCGGCTACCAGGTGTACACGCAGCCGGTGTTCGCGTTCGCGGACCGCAAGTTCGGCGGCGGGGCCACGGTCGTCGAGGC

GCCGCTGCTGCCGGTGCCGGGCGCGCGCCGCGTGAACGCGAACGTGTTCAGGCTGTGCTTCCGCACGGCGTACGTGGCGGCGACCACGGCGCTGGCCGTC

TGGTTCCCCTACTTCAACCAGATCATCGGGCTGCTCGGCTCCTTCACCTTCTGGCCGCTCGCCGTCTACTTCCCCGTCGAGATGTACCTCACGAGGAACA

AGGTGGCGCCGTGGACCAACCAGTGGCTCGCCATCCATGCGTTCAGCCTCGTCTGCCTGCTCATCAGCGCGTTCGCCTCCGTCGGCTCTGCGGTTGGCGT

GTTCGGGTCGGAGACGAGCTGA

>ZmAAAP45

ATGGCCGACAGCAGGAGAAGTGTAGTGTACGATGCTGAAGGCGGCGACGATCATGAGAGGCAAGGGACGGCGTGGACGGCGACGTCGCACATCGTGGCGG

CGGTGGTGGGCTCCGGCGTGCTGGCGCTGGCGTGGACGGTGGCGCAGCTGGGGTGGGTGGTGGGTCCCCTCGTGCTGGTGGGCTTCTCTTGCGTGACCTA

CTACACGTCGGCGCTGCTCGCAGACTGCTACCGCTACCCGGACCCCGTCCACGGCGCCGTCAACCGCGAGTACATCGACGCCGTCCGATGCTACCTGGAC

CGGAAGAACGTGGTGCTGTGCGGGTGCGCCCAGTACGTGAACCTGTGGGGCACTCTCGTCGGCTACACCATCACCGCCAGCGCAAGCATGATCGCGATTA

AGCGCGTCAACTGCTTCCACCGGGACGGCTACGGCGCCGCCGGCTGCAACCCGTCGGGGAGCACGTACATGGTGGTGTTCGGGCTCTTCCAGCTCCTGCT

GTCGCAGCTGCCCAGCCTCCACAACATCGCCTGGCTGTCCGTGGTGGCCGTGGCCACCTCCCTGGGCTACTCCTTCATCAGCCTGGGCCTGTGCTCCGCC

AAGTGGGCCTCGCACGGCGGCCACGTGCGCGGCACGCTGGCGGGCGCCGCCGCCGTGGCCGGCCGGGCCGACGACGACAAGCAGGCGGCCTTCAACGTGC

TCCTCGCGCTCGGCAACATCGCCTTCTCCTACACCTTCGCCGACGTGCTCATCGAGATCCAGGACACGCTGCGCTCGCCGCCCGCCGAAAACCGCACCAT

GAAGCGGGCCTCCGCCTACGGCCTCGCCATCACCACCGTCTTCTACCTCGCGCTCGGCTGCACCGGCTACGCCGCCTTCGGCGACCACGCGCCGGGGAAC

ATCCTCACGGGCTTCGCCTTCTACGAGCCCTTCTGGCTCGTCGACGCCGCCAACGTCTGCGTCGTCCTGCACCTCGTCGGCGCCTACCAGGTCTTCGCGC

AGCCCATCTTCGCGAGGCTCGAGTCCTGCGTCGCCTGCCGCTGGCCCGACGCCAAGCTCATCAACGCCACCTACTACGTGCGCGTGCCGCCATGCCTCCT

CCTCCTCCGCACGTCGTCGTCCTCGTCGCCGCCGCCCACCCTCGCCGTCGCGCCGCTCAAGCTCGTCCTGCGCACCATCGTCATCATGTTCACAACGCTC

GTCGCCATGCTGGTGCCCTTCTTCAACGCCGTGCTGGGCCTCATCGGCGCGCTCGGCTTCTGGCCGCTCTCCGTCTACTTCCCCGTCAGCATGCACATGG

CAAGGCTCAACATCCGACGCGGAGAGATCCGCTGGTGGATGCTGCAGGCCATGAGCTTCGTCTGCCTCCTCATCTCCGTCGCAGCCAGCATCGGATCCGT

GCACGACATCGTGCACAACCTCAAGGCCGCCGCGCCATTCAACACTGCAAACTGA

>ZmAAAP46

ATGCGTGACGGAGGAGGCGCCATGGACGTCGACATGCAAGCCCGCGGTGGCGGTGCTAGCCATGGCGGCGAACTTGACGACGACGGCAAGGAGAAGAGGA

CAGGGACGGTGTGGACGGCGTCGGCGCACATCATCACGGCGGTGATCGGGTCCGGCGTGCTGTCGCTGGCGTGGGCGATGGCCCAGCTGGGCTGGGTGGC

CGGGCCTGTGATCCTGCTGCTCTTCGCGGCCATCACCTACTACACCTCCTGCCTCCTCACCGACTGCTACCGCTTCGGCGACCCTGTCACCGGGAAGCGC

AACTACACCTACACTGAGGCCGTCGAGTCCTACCTAGGCGGCAGGTATGTCTGGTTCTGCGGCTTCTGCCAGTACGCCAACATGTTCGGCACCGGCATCG

GCTACACCATCACGGCTTCTGCCAGCGCTGCGGCTATCCTCAAGTCCAACTGCTTCCACTGGCACGGGCACGACGCCGACTGCACCCAGAACACGGGCTC

CTACATCGTCGGCTTCGGCGTGGTGCAGGTCATCTTCAGCCAGCTCTCAAACTTCCACGAGCTCTGGTGGCTCTCCGTGCTCGCGGCCGCCATGTCCTTC

TGCTACTCCACCATCGCCGTCGGGCTCGCCCTCGGCCAGACCATCTCAGGTCCCACGGGCAAGACGACGCTCTACGGCACGCAGGTCGGGGTGGACGTTG

GTAGCGCGGAGGAGAAGATATGGCTGACGTTCCAGGCTCTCGGCAACATCGCGTTTGCCTACTCCTACACCATTGTTCTCATTGAAATCCAGGACACGCT

GCGATCTCCGCCGGCCGAGAACAAGACGATGCGGCAGGCGTCGGTCCTGGGCGTGGCGACGACCACGGCGTTCTACATGCTGTGCGGCTGCCTGGGCTAC

TCGGCGTTCGGCAACGCGGCGCCGGGTGACATCCTGTCGGGCTTCTACGAGCCCTACTGGCTGGTGGACTTCGCCAACGTCTGCATCGTGATCCACCTCG

TGGGCGGCTTCCAGGTGTTCCTGCAGCCGCTGTTCGCCGCGGTCGAGGCCGACGTGGCGGCGCGCTGGCCGGCCTGCTCCGCGCGGGAGCGCCGCGGCGG

CGTCGACGTGTTCCGCCTGCTGTGGCGCACCGCGTTCGTCGCGCTCATCACCCTGTGCGCCGTCCTGCTGCCCTTCTTCAACAGCATCCTGGGAATCCTC

GGCAGCATCGGCTTCTGGCCGCTCACCGTCTTCTTCCCCGTCGAGATGTACATCCGCCAGCAGCAGATCCCGCGGTTCAGCGCCACGTGGCTGGCGCTGC

AGGCCCTCAGCATCTTCTGCTTCGTCATCACCGTCGCGGCCGGGGCTGCCTCGGTACAGGGCGTGCGCGACTCGCTCAAAACCTACGTGCCCTTCCAGAC

CAGGTCGTGA

>ZmAAAP52

ATGGTGTCGGAGAGGCAGCAGGCGGCGGGGAAGGTGGCCGCCTTCAACCTCACGGAGGCCGGGTTCGGCGACGGGTCGGACCTGCTGGACGACGACGGGC

GCGAGAGGCGCACGGGGACCCTGGTGACGGCGAGCGCGCACATCATCACGGCGGTGATCGGGTCGGGCGTGCTGTCGCTGGCGTGGGCGATCGCGCAGCT

GGGGTGGGTGATCGGCCCCGTGGTGCTGCTGGCCTTCTCCGCCATCACCTGGTTCTGCTCCAGCCTACTCGCCGACTGCTACCGCGCGCCGCCGGGCCCC

GGCCAGGGCAAGCGGAACTACACCTACGGACAGGCCGTCAGGTCATACCTGGGGGAGTCCAAGTACCGGCTGTGCTCGCTGGCGCAGTACGTGAACCTGG

TGGGCGTCACCATCGGCTACACCATCACCACGGCCATCAGCATGGGGGCGATCAAGCGTTCCAACTGCTTCCACAGCAGGGGCCACGGCGCCGACTGCGA

GGCGTCCAACACCACCAACATGATCATCTTCGCGGGCATCCAGATCCTGCTGTCGCAGCTCCCCAACTTCCACAAGCTCTGGTGGCTCTCCATCGTCGCC

GCCGTCATGTCCCTCGCCTACTCCTCCATCGGACTCGGCCTCTCCATCGCAAAGATCGCAGGTGGGGTGCACGTTAAGACGTCGCTGACTGGTGCCGCCG

TGGGGGTGGACGTCACCGCGGCCGAGAAGGTCTGGAAGACGTTCCAGTCGCTGGGGGACATCGCCTTCGCCTACACCTACTCCAACGTGCTGATCGAGAT

CCAGGACACGCTGCGGTCGAGCCCGCCGGAGAACGTGGTGATGAAGAAGGCGTCCTTCATCGGCGTGTCCACCACCACCGCGTTCTACATGCTGTGCGGC

GTGCTGGGCTACGCGGCGTTCGGCAGCGACGCGCCGGGCAACTTCCTCACGGGCTTCGGCTTCTACGACCCCTTCTGGCTCATCGACGTCGGCAACGTCT

GCATCGCCGTGCACCTGGTCGGCGCCTACCAGGTCTTCTGCCAGCCCATCTACCAGTTCGTGGAGGCCTGGGCGCGGGGCCGCTGGCCCGACTGCGCCTT

CCTCCACGCCGAGCTCGCCGTCGTCGCCGGCTCCTCCTTCACGGCCAGCCCGTTCCGCCTCGTGTGGCGCACCGCCTACGTCGTGCTCACCGCGCTCGTC

GCCACGGTCTTCCCATTCTTCAACGACTTCCTGGGGCTCATCGGCGCCGTCTCCTTCTGGCCGCTCACCGTCTACTTCCCCATCCAGATGTACATGGCGC

AGGCCAAGACGCGCCGCTTCTCGCCGGCGTGGACGTGGATGAACGTGCTCAGCTACGCTTGCCTCTTCGTCTCGCTGCTCGCCGCCGCGGGCTCAGTGCA

GGGGCTCGTCAAGGATCTCAAGGGATACAAGCCATTGTTCAAGGTCTCCTAA

>ZmAAAP54

ATGGAGGTGAGCTCCGTGGAGTTCGGTCATCACGCGGCGGCCGCCTCAAAGTGCTTTGACGACGACGGTCGCCTCAAGCGCACAGGGACGATGTGGACGG

CGAGCGCGCACATTATCACGGCCGTGATAGGGTCCGGGGTGCTGTCGCTCGCGTGGGCCATCGCGCAGCTCGGCTGGGTGGCAGGCCCCACCGTCATGCT

GCTCTTCTCCTTCGTCACCTACTACACATCGGCCCTACTCGCCGACTGCTACCGCTCCGGCGACGCCTGCACCGGCAAGCGCAACTACACGTACATGGAC

GCGGTTAACGCCAATCTCAGTGGCGTCAAGGTCTGGTTCTGCGGGTTCCTGCAGTACGCCAACATCGTCGGAGTCGCCATAGGCTACACCATTGCCGCCT

CTATTAGCATGCTGGCGATCCAGAGGGCGAACTGCTTCCACGTGGAGGGGCACGGGGACCCCTGCAACATCTCCAGCACGCCCTACATGATCATCTTCGG

CGTCGTGCAGATTTTCTTCTCGCAGATCCCGGACTTCGACCAGATATCGTGGCTCTCCATCCTCGCCGCCGTCATGTCGTTCACCTACTCCACCATCGGC

CTGGGCCTGGGCATCGCGCAGGTGGTGTCCAACAAGGGCGTGCAGGGCAGCCTGACGGGGATCAGCGTCGGCTTGGTCACCCCGGTCGACAAGATGTGGC

GCAGCCTGCAGGCGTTCGGCGACATCGCCTTCGCCTACTCCTACTCGCTCATCCTCATCGAGATCCAGGACACCATCCGCGCGCCGCCGCCGTCCGAGTC

CAAGGTCATGCGGCGCGCCACCGTCGTCAGCGTGGCCGTCACCACGTTCTTCTACATGCTGTGCGGGTGCATGGGGTACGCCGCGTTCGGGGACAACGCC

CCCGGGAACCTCCTCACGGGCTTCGGCTTCTACGAGCCCTTCTGGCTCCTCGACGTCGCCAACGCCGCCATCGCCGTGCACCTCGTCGGCGCCTACCAGG

TCTACTGCCAGCCCCTGTTCGCCTTCGTCGAGAAGTGGGCGCGCCAGAGGTGGCCCAAGTCCCGCTACATCACGGGCGAGGTCGACGTCCCGCTCCCGCT

CGGGACCGCCGGCGGCCGGTGCTACAAGCTCAGCCTGTTCCGGCTGACGTGGCGGACGGCGTTCGTGGTGGCCACGACGGTGGTGTCCATGCTGCTGCCC

TTCTTCAACGACGTGGTCGGGCTCCTGGGCGCGCTGGGGTTCTGGCCGCTCACCGTCTACTTCCCCGTGGAGATGTACATCGTGCAGAAGAAGGTGCCCA

GGTGGAGCACGCGGTGGGTGTGCCTGCAGCTGCTCAGCGTCGCCTGCCTCGTCATCACCGTCGCCTCCGCCGCAGGCTCCGTTGCCGGGATCGTCTCTGA

CCTCAAAGTGTACAAACCGTTCGTCACCACCTCCTGA

>ZmAAAP55

ATGGACGTGGAGAGGAAGGTGGTGGAGGCGGACGACGACGGCCGCGTCAGAACAGGGACGGTGTGGACGGCGACGACGCACGCCATCACCGCCGTGATAG

GCTCCGGCGTGCTGGCGCTGCCCTGGAGCGTGGCGCAGATGGGCTGGGTCCTCGGCCCCGTCGCCCTCGTCGGCTGCGCCTACATCACCTACTTCACCGC

CGTCCTCCTGTCCGACTGCTACCGCACGCCGGACCCCGTCCACGGCAAACGGAACCGCACCTACATGGACGTCGTCCGCTCATGCCTCGGGCCTAGAGAT

GTGGTGGTGTGTGGACTCGCACAGTACGCGATCCTCTGGGGGACAATGGTGGGCTACACCATCACCACTGCCACGAGCATCATGGCCGTCGCGCGCACGG

ACTGCCGCCACCACAGGGGCCACGACGCGGCCTGCGCCTCGTCCGGGACGGTGTACATGGTGGCGTTCGGCGTCGTCGAGGTGGTCCTGTCCCAGTTCCC

GAGCCTGGAGAAGCTCACGATCATCTCCGTGGTCGCTGCCGTCATGTCGTGCACCTACTCCTTCGTCGGGCTGTTCCTGAGCGCCGCCAAGCTCGCGTCG

AACCACGGGGCGCGCGGCAGCCTCCTCGGCGTCAAGATCGCCGCGGGCGTCTCCGCGTCGACCAAGACGTGGCACTCACTGCAGGCACTCGGCAACGTTG

CCTTCGCGTACACCTACTCCATGCTTCTCATAGAAATCCAGGACACTGTGAAGGCACCGCCATCAGAGAACGTGACGATGAAGAGGGCCAGCTTCTACGG

CATCAGCGTGACGACCATCTTCTACGTCTCGCTCGGGTGCATCGGCTACGCGGCCTTCGGCAACGCCGCGCCGGGGAACGTGCTTACCGGCTTCGACGAG

CCGTTCTGGCTCGTCGACGTCGCCAACATCGCCGTGGTCGTCCACTTGGTTGGGGCATATCAGGTGTACGCGCAGCCGATCTTCGCGTGCTACGAGAAGT

GGCTGGGGTCCCGGTGGCCGGACTCGGCCTTCTTCCACCACGAGTACGCGGTGCGCCTGCCCGGCTGCGCGGTGCGGTTCACGATGTGCAAGCTGGTGCT

GCGCACGGCGTTCGTGGCCGCCACGACGGTGGTGTCGCTGATGCTGCCCTTCTTCAACGCCGTGCTCGGGCTGCTGGGCGCCATCGCCTTCTGGCCGCTG

ACGGTGTACTTCCCGGTGACCATGTACATCGCGCAGGCCAAGGTGGCGCCCGGCAGCCGCAAGTGGGTGGCGCTGCAGGCGCTCAATGTGGGCGCGCTCC

TGGTTTCGCTGCTCGCCGCCGTGGGCTCCGTGGCCGACATGGTGCAGCGCCTGGGCCATGTCACCATCTTTCAGACGCAGCTCTGA

>ZmAAAP56

ATGGACAAGAGCGGCGGCGAGGCGGCGGCGGCGGCAGCAGCAGCAGACGACGTCGAGCGGCGGGGGGGCGACTACGAGCAAGACGAGCACGAGCGGAGAG

GGACGGTGTGGACGGCGACGGCGCACATTGTGACGGCGGTGATCGGCTCCGGCGTGCTGGCGCTGGCCTGGAGCGTGGCGCAGCTGGGCTGGGTCGCGGG

GCCCCTCGCGCTCGCCGGCTTCGCGTGCGTCACCTACTACACCTCCACGCTGCTCGCCGGCGCCTACCGCGCGCCGCACCCCGTCACCGGCCACAGGAAC

CGCACCTACATGGACGCCGTCAGATCGTACCTCAGTCCCAGGGAGGTGTTCATGTGCGGGGTCGCGCAGTACGTGAACCTGTGGGGCACCATGGTCGGGT

ACACCATCACGGCGACCATAAGCATGGCCGCGATCAGGCAGGCCGACTGCCTCCGCCGGGACGGCGCCGGCGCCGGCGCGCGCTGCGACGCGCCAGGGAC

CGTGCTGATGCTGGCGTTCAGCGTGGTCCAGGTGGTGCTGTCCCAGTTCCCGGGCCTGGAGCACATCACCTGGCTGTCCGTCGTCGCGGCGGCCATGTCG

TTCGCCTACTCCTTCGCCGGCCTCGGCCTCTCCGTAGGGCACTGGGTGTCTCGTGGCGGCGGCGGCCTCGGGGGCAGGGTTGCAGGTGCCGCCGCGGCAT

CCTCCACCAGGAAGCTCTGGAACGTGCTTCTCGCCCTGGGGAACATTGCCTTCGCCTACACTTTCGCTGAAGTATTGATCGAGATCCAGGATACACTCAA

GTCACCACCACCGGAGAACAGGACGATGAAGAAGGCAGCAATGTACGGGATCGGAGCCACCACCATCTTCTACATCTCCGTCGGCTGCGCTGGGTACGCC

GCGTTCGGTTCGAATGCTCCGGGCAACATCTTGGCGGCAGGCGGGCTGGGTCCCTTGTGGCTCGTCGACATTGCCAACATGTGCCTCATCCTCCACCTGA

TCGGTGCATACCAGGTATACGCTCAGCCTGTCTTCGCTTCGGTTGAGAGGTGGGCCGCCTCACGGTGGCCAGAAGCCAAGTTCATGAGCAGCGCATACAC

CGTGTCCGTCAGCATCCCCCTCTTGCAGAGAGGATCGGTCACCGTCGCGCCGCACAAGCTCGTCCTGAGGACCGCCATAGTCGGCGCGACGACTGCGGTG

GCGCTGGCGATACCCTTCTTCAACGCCGTGCTGGGGCTCCTCGGCGCGTTCAGCTTCTGGCCGCTCACGGTCTACTTCCCCATCAGCATGCACATCGCCC

AGGGCAAGATCGCCAGGGGGACCAAGTGGTGGTGCCTTCTGCAGGCTCTGAGCATGGTTTGCTTGGTGATCTCGGTGGCCGTGGGTGTAGGCTCTGTCAC

TGACATTGTCGATAGCCTCAAGGCCTCTTCTAGCCCTTTCAAAATTGTAGGCTAA

>ZmAAAP59

ATGGTAGGTGCGATGCGCGGCGGAGCCATGGAGTTGGAGGACCGCCTGGCCACCCTTCCTCGCTTCCGCGGCGATCACGATGACGACGGCAAGGAAAGGA

GGACAGGGACGGTATGGACGGCAACGGCGCACATCATCACGGCGGTGATCGGCTCCGGCGTGCTGTCGCTGGCGTGGGCGATGGCGCAGCTGGGGTGGGT

GGCCGGGCCGTTGACCCTGGTGCTCTTCGCGGCGATCACCTTCTACACCTGCGGCCTCCTCGCAGACTGCTACCGCGTTGGCGACCCCGTGACGGGCAAG

CGCAACTACACCTACACCGAGGCCGTCAAGAGCAACCTGGGCGGCTGGTACGTCTGGTTCTGCGGCTTCTGCCAGTACGTCAACATGTTCGGCACAGGCA

TCGGCTACACCATCACAGCCTCCATCAGTGCAGCGGCCATCAACAAGTCCAACTGCTTCCACTGGCACGGCCACGATGCCGACTGCAGCCAGAACACCAG

CGCCTACATCATCGGCTTCGGCGTGGTGCAGGTCATCTTCAGCCAGCTCCACAACTTCCACAAGCTGTGGTGGCTCTCCATCATCGCCGCCATCATGTCC

TTCTCCTACTCCGCCATCGCCGTGGGCCTCTCCCTGGCGCAGATCGTCATGGGCCCCACGGGGAAGACCACCATGACCGGCACCCTGGTCGGGGTGGACG

TGGACGCTGCGCAGAAGGTGTGGATGACGTTCCAGGCGCTGGGCAACGTGGCCTTCGCGTACTCGTACGCCATCATCCTCATCGAGATCCAGGACACGCT

GCGCTCCCCGCCCGCCGAGAACAAGACCATGCGCCGCGCCACCATGATGGGCATCTCCACCACCACCGGCTTCTACATGTTGTGCGGCTGCCTCGGCTAC

GCCGCGTTCGGCAACGCCGCGTCGGGGAACATCCTCACCGGCTTCGGCTTCTACGAGCCCTTCTGGCTCGTCGACTTCGCCAACGCCTGCATCGTCGTGC

ACCTCGTCGGCGGCTTCCAGGTCTTCTGCCAGCCGCTGTTCGCGGCCGTCGAGGGCGCCGTGGCGGCGCGGTACCCCGGGTCGACGCGCGAGTACGGCGC

CGCGGGCCTCAACGTCTTCCGCCTCGTGTGGCGCACGGCGTTCGTGGCTGTCATCACGCTGCTGGCCATCCTCATGCCCTTCTTCAACAGCATCCTGGGC

ATCCTCGGCAGCATCGCCTTCTGGCCGCTCACCGTCTTCTTCCCCGTCGAGATGTACATCCGGCAGCGGCAGGTGCGAAGGTTCAGCACCAAGTGGATAG

CGCTGCAGAGCCTCAGCTTCGTCTGTTTCCTCGTCACCGCCGCCTCCTGCGCGGCCTCCGTGCAGGGCGTGGTCGACTCGCTCAAGACCTACGTGCCGTT

CAAGACGAGGTCGTGA

>ZmAAAP60

ATGACGCAGCAGGACGTGGAGATGGCGGCGCGCCACGGGACCGGCGCCGACGGAGCGGGATTCTACCCTCAGCCGCGGAACGGCGCCGGCGGCGAGACGC

TCGACGACGACGGCAAGAAGAAGCGCACGGGAACGGTATGGACGGCAAGCGCGCACATCATCACAGCCGTCATCGGCTCCGGCGTGCTCTCCCTCGCCTG

GTCGACTGCACAGCTGGGCTGGGTCGTGGGGCCGCTCACCCTGATGATCTTTGCCTTGATCACGTACTACACCTCTAGCCTTCTTGCTGACTGCTACCGC

AGCGGCGATCAGCTCACCGGCAAGAGGAACTACACCTACATGGACGCTGTTGCCGCGTACCTGGGTCGATGGCAAGTCCTGTCCTGTGGTGTTTTCCAGT

ATGTTAACTTGGTTGGAACTGCCGTTGGGTATACAATTACAGCGTCCATCAGTGCAGCGGCCGTGCACAAGGCAAACTGCTTCCACAACAAGGGCCACGC

GGCCGACTGCAGCACCTACGACACCATGTACATGGTCGTATTTGGGATCGTTCAGATCTTCTTCTCTCAGCTCCCTAACTTCAGCGACCTTTCGTGGCTG

TCCATCGTCGCCGCCATCATGTCGTTCTCTTACTCCAGCATCGCCGTCGGCCTCTCGTTGGCGCGGACCATTTCAGGCCGTAGTGGTACGACCACTCTGA

CCGGCACTGAGATCGGAGTCGACGTTGATTCAGCCCAGAAGGTCTGGCTCGCGCTTCAAGCTCTTGGCAACATCGCGTTCGCTTACTCCTACTCCATGAT

TCTCATCGAAATCCAAGACACGGTGAAGTCTCCTCCAGCCGAGAACAAGACGATGAAGAAGGCGACGCTGATGGGCGTGACGACCACCACGGCGTTCTAC

ATGCTTGCTGGCTGCCTCGGGTACTCGGCATTCGGGAACGCGGCGCCAGGGAACATCCTGACCGGGTTCGGCTTCTACGAGCCCTACTGGCTGATCGACT

TCGCCAACGTCTGCATCGTGGTGCACCTGGTGGGCGCGTACCAGGTCTTCTCCCAGCCCATCTTCGCGGCCTTGGAGACGGCGGCCGCCAAGCGCTGGCC

GAACGCCAGGTTCGTCACGCGCGAGCACCCCCTCGTGGCCGGCAGGTTCCACGTCAACCTGCTCAGGCTGACGTGGAGGACGGCGTTCGTGGTGGTGAGC

ACGGTGCTCGCCATCGTGTTGCCCTTCTTCAACGATATCCTGGGCTTCCTCGGCGCCATCGGCTTCTGGCCGCTCACCGTGTACTACCCAGTGGAGATGT

ACATCCGGCAGCGGCGTATACAGAAGTACACCAGCAGGTGGGTGGCGCTGCAGCTGCTCAGCTTCCTGTGCTTCCTGGTCTCGCTCGCCTCGGCGGTCGC

GTCCATCGAGGGAGTCACCGAGTCGCTCAAACACTACGTTCCCTTTAAGACCAAGTCGTGA

>ZmAAAP64

ATGGCCGTGTCACACAACGTTGGGAGCAAGCACGGTGTCGCGCCGCTGGAGGTGTCGGTGGAGGCCGGGAACGGCGGAGCCGCCGAGTGGCTGGACGACG

ATGGCCGGCCTCGCCGCACGGGCACGTTCTGGACGGCCAGCGCGCACATCGTCACCGCCGTCATCGGCTCCGGGGTGCTCTCGCTCGCCTGGGCGATCGC

GCAGCTGGGCTGGGTCGCCGGCCCCGCCGCCATGCTCCTCTTCGCCTTCGTCACATACTACACCGCCACGCTGCTCGCCGAGTGCTACCGCACGGGCGAC

CCGGAGACGGGCAAGCGCAACTACACCTACATGGACGCCGTGCGCTCCAACCTCGGCGGCGCCAAGGTCGCGTTCTGCGGCGTCATACAGTACGCCAACC

TCGTCGGCGTCGCCATCGGCTACACCATCGCGGCGTCCATCAGCATGAAGGCCGTCAGGAGAGCTGGGTGCTTCCACGCCCACGGGCACGCTGATCCCTG

CAACAGCTCCAGCACCCCGTACATGATCCTCTTCGGCGTCGTGCAGATCCTCTTCTCGCAGATACCCGACTTCGACCAGATTTGGTGGCTCTCCATTGTC

GCCGCCGTCATGTCCTTCACTTACTCTTCCATCGGGCTCTCCCTCGGCATCGCACAGACCATCTCCAATGGTGGGTTCATGGGCAGTCTCACTGGCATCA

GCATCGGCGCCGGTGTCACCTCCACGCAGAAGATCTGGCATACGCTTCAGGCATTCGGAGACATCGCCTTCGCCTACTCCTTCTCCAACATCCTCATCGA

GATCCAAGTAAGCAACAATCGAGATCTTGTACTCTACACTGCACTGCAACAGGATTTCCCTCCCCTGTTTCTAACAAAGGCCGCTGTTCTAACTCTGGTT

CTTGTTCAGGACACGATCAAGGCACCGCCACCGTCGGAGTCCAAGGTGATGCAGAAGGCGACGCGCCTCAGCGTGGCGACGACCACCATCTTCTACATGC

TGTGCGGGTGCATGGGGTACGCGGCGTTCGGCGACAAGGCGCCGGACAACCTCCTCACCGGCTTCGGCTTCTTCGAGCCGTTCTGGCTCATCGACATCGC

CAACGTCGCCATCGTCGTGCACCTGGTCGGCGCGTACCAGGTGTTCTGCCAGCCCATCTTCGCCTTCGTCGAGCGCCGCGCCGCCGCGGCCTGGCCCGAC

AGCGCCTTCGTCTCCCAGGAGCTCCGCGTGGGCCCCTTCGCGGTCAGCGTGTTCCGCCTGACATGGCGGTCGTCCTTCGTGTGCGTCACCACCGTCGTTG

CCATGCTGCTGCCGTTCTTCGGCAACGTGGTGGGGTTCCTCGGCGCCGTCTCCTTCTGGCCGCTCACCGTCTACTTCCCCGTCGAGATGTACATCAAGCA

GCGCCGCGTGCCGCGCGGCAGCACCAAGTGGATCTGTCTCCAGACGCTCAGCGTCAGCTGCCTCCTCGTCTCCGTG

>ZmAAAP65

ATGGCGTCGCACAACGGCACCAAGCACCTGGCGCCGATGGAGGTGTCGGTGGAGGCCGGGAACGGCGGCGGCGCCGAGTGGCTGGACGACGACGGGCGGC

CGCGGCGCACGGGCACGTTCTGGACGGCCAGCGCGCACATCATCACGGCCGTCATCGGCTCGGGAGTCCTGTCCCTGGCCTGGGCCATCGCGCAGCTGGG

GTGGGTGGCCGGCCCCGCCGCCATGCTGCTCTTCGCCTTCGTCACCTACTACACCGCCACGCTGCTCGCCGAGTGCTACCGCACGGGGGACCCGGACACG

GGCAAGCGCAACTACACGTACATGGACGCCGTGCGCTCCAACCTCGGCGGCGCCAAGGTCGCCTTCTGCGGCGCCATCCAGTACGCCAACCTCGTCGGCG

TCGCCATCGGCTACACCATCGCCTCCTCCATCAGCATGCAGGCCGTCAGCAGGGCCGGGTGCTTCCACAAGCGTGGGCACGCCGTCCCCTGCAAGAGCTC

CAGCAACCCCTACATGATCCTCTTCGGCGCCGTCCAGATCCTCTTCTCCCAGATACCGGACTTCGACCAGATTTGGTGGCTCTCCATCGTCGCCGCAGTC

ATGTCCTTCACCTACTCCGCCATCGGCTTGTCCCTCGGCATCGCACAGACTGTTGCAAACGGTGGGTTCAAGGGAAGCCTCACCGGCATCAGCATCGGCG

CCGACGTCACCTCCACGCAGAAAGTGTGGCACAGCCTGCAGGCCTTCGGCGACATCGCGTTCGCCTACTCCTTCTCCAACATCCTCATCGAGATCCAAGA

CACGATCAAGGCGCCGCCGCCGTCGGAGTCGAAGGTGATGCAGAAGGCGACGCGGCTGAGCGTGGCGACGACGACCATCTTCTACATGCTGTGCGGGTGC

ATGGGGTACGCGGCGTTCGGCGACAAGGCGCCGGACAACCTGCTGACGGGGTTCGGCTTCTTCGAGCCATTCTGGCTGATCGACGTGGCCAACGTGGCCA

TCGTGGTGCACCTGGTGGGCGCGTACCAGGTGTTCTGCCAGCCCATCTTCGCCTTCGTGGAGCGCCGCGCCGCCGCGGCCTGGCCCGACAGCGCCTTCGT

CTCGCGGGAGCTCCGCGTGGGGCCCCTCGCCCTCAGCGTGTTCCGCCTCACGTGGCGGTCGGCGTTCGTGTGCGTCACCACCGTCGTGGCCATGCTGCTC

CCCTTCTTCGGCAACGTGGTGGGGTTCCTCGGCGCCGTCTCCTTCTGGCCCCTCACCGTCTACTTCCCCGTCGAGATGTACATCAAGCAGCGCCGCGTGC

CCCGCGGCAGCACCAAGTGGGTCTGCCTCCAGACGCTCAGCGTCGCGTGCCTGGTCGTCTCCATCGCCGCCGCCGCCGGCTCCATCGCCGACGTCATCGA

GGCGCTCAAGGTTTACCACCCGTTCAGCAGTTAA

>ZmAAAP66

ATGGCACCCGCGCCGCACAACGGACTGAACAACAACCACCCGGTCGCGCCCATGGATGTGTCGGTGGAGGCCGGGAACGCCGGAGCCGCCGAGTGGCTAG

ACGACGACGGCCGGCCGCGCCGCAGTGGCACGTTCTGGACGGCCAGCGCGCACATCATCACCGCCGTCATCGGCTCGGGAGTCCTCTCCCTGGCGTGGGC

CATCGCGCAGCTGGGCTGGGTGGCCGGCCCCGCCGCCATGCTCCTCTTCGCCTTCGTCACCTACTACACGGCGGCGCTGCTCGCCGAGTGCTACCGCACG

GGCCACCCGGAGACGGGCAAGCGCAACTACACCTACATGGACGCCGTGCGCTCCAACCTCGGCGGCGTCAAGGTCGTCTTCTGCGGCGTCATACAGTACG

CCAACCTCGTCGGCGTCGCCATCGGCTACACCATCGCGTCGGCCATCAGCATGAAGGCCGTCAGGAGGGCCGGGTGCTTCCACGCCCACGGGCATGCGGA

CCCCTGCAAGAGCTCCAGCACCCCGTACATGGTCCTGTTCGGCGGTGTCCAGATTCTCTTCTCGCAGATACCGGACTTCGATCAGATTTGGTGGCTCTCC

ATTGTCGCCGCCGTCATGTCCTTCACTTACTCTTCCATCGGACTCTCCCTCGGCATCGCACAGACCGTCTCCAATGGTGGGTTCAAGGGCAGCCTCACTG

GCATCAGTATTGGCGCCGGCGTCACCTCCACGCAGAAGATCTGGCACACGCTCCAGGCGTTCGGCGACATCGCGTTCGCCTACTCCTTCTCCAACATCCT

CATCGAGATCCAAGATACGATCAAGGCGCCGCCACCGTCGGAGTCGAAGGTGATGCAGAAGGCGACGCGGCTGAGCGTGGCGACCACGACAGTATTCTAC

ATGCTGTGCGGGTGCATGGGGTACGCGGCGTTCGGCGACAACGCGCCGGACAACCTCCTCACCGGCTTCGGCTTCTACGAGCCCTTCTGGCTGCTCGACG

TCGCCAACGTGGCCATCGTCGTGCACCTGGTCGGCGCCTACCAGGTGTTCTGCCAGCCCATCTTCGCCTTCGTGGAGCGCCGCGCCGCCGCGGCCTGGCC

GGACAGCGCCTTCGTCTCCCGGGAGCTCCGCGTGGGTCCCTTCTCCCTCAGCGTGTTCCGCCTGACGTGGCGGTCCGCGTTCGTGTGCGTCACCACCGTC

GTCGCCATGCTGCTGCCGTTCTTCGGCGACGTGGTGGGGCTCCTCGGCGCCGTCTCCTTCTGGCCGCTCACCGTCTACTTCCCCGTCGAAATGTACATCA

AGCAGCTCCGCGTGCCCCGCGGCAGCACCAAGTGGATCTGCCTCCAGACGCTCAGCGTCAGCTGTCTCCTTGTCTCCGTCGCCGCTGCAGCAGGATCCAT

TGCCGATGTCATCGCCGCCCTCAAGGTCTACAAGCCGTTCAGCGGTTAG

>ZmAAAP67

ATGGAGCCCAACCTTCAATCCGCACTGGAGGAACAAACGAAGATTCTTCGCGCGCTCAGTGCCCGCCTCGCTGCCCAAGAAGCGCGGTGGCGCAGTTGGG

AGTCGAAAGTGGCGCACCATTCGGTGTCGATCCACGACCTTGAGGTAGCAGTGGCCACTGTGCCATCCGCGACGTTGAGGTCGGAGCGAGACGCTCAGGT

CGCCGCGACCCGCGAGTGTCTGGCCGTGGTCGCTGACGACTGGGGTGGCCTCTTCGGCACAGGCGACGGCTGTATCGTCGCCGATAACTGGGGTGGGCTG

TTCGAGTATCCTGCTCACTTCCAGGAGGAACAGGACATCGACAACTGCATCCCCATCAATGATGGCACCCGCTTCACCGAGGAAGACGACGACAACCACC

CCGCCACGCTCGAACCACTGCTCACCGACACCACCACCGCCGCCGAGGTCGATTCCATCGCCAACACCGACGATGTCGAGGCTGACTCTCTCGCCTCCGC

CTCCACGGTGGGGGTCGCCACATCCCAGGCCGCCGAGATGCAGCTCCACGTCGGGTGGGCGCGCTGGCTGGAGGGACAGGCCGACTACCTAAGACACGAT

CGGATGCTCCACTCCAATCTAGATTCCCTCACTTCGAGCAACCACCCGCTGCTCCAGGATGCCTCAGCCACCAACAACTCGCCCACCCAGTACTCGACAG

AATGCTTCAGCCATGACATCGTCTTGTTGAGGACTATCAGTGCTGCGCCCACAAGCGAGGACCGCGCGCTGCAGCAGAGGCGCCTCGAGTGCCTCGTCGC

GTGGCCCGAGTGGCGGAGGATTGGCGGACGGAACTACATCATGCTCGAGCATCACCCCAACAGGATGTTCGACGCGTGCGATAGGTTCTGGCACTGCGTC

TTCGCGCTCTATGACTTCGGCTCTGTGCAGGGCCTGCAGAGGAAGCTGGAGCGGCTCTGCCTGGCGGGGAGCGCACCTGACCTGGAAGTCCTCGCCGTGC

TTTCGGGCCTGCGCGCGCGAAGCCTTTCCGACCAGCGTGCCGCTAACCATACCCGCACGTCCATGCGGAGCATATCCACGACCGCCACCCCGTTCAGACT

CTGCTCTACACGGAGCGGCTACATCGCAAGCGGGACTTCGACGGGCACGACTTGCAGCACCAGGTCACAGAGCTGGATCTCCTCCGAGTTCGAGCACTCC

TCCATTCCGACCCCCAACCAGAAGGTATTCAACCTCTCGTCTGATGTCCTCAATAAGAGAGATCCGTGGGTCGGTACCTTCGACCACATTTTCACCGAGC

TCGGCCAACCACAAAGCCTAGAAGCTGTCCGACTTCTCTGTCACCTACGTCATCAGCTACCAGACTGCTCCCAGTTGGAATTCACATACGAGGTGTTTGC

TGCACAGAAGGTGTTCGAGGAATTGTCTCTTAGCTCCCCTGCTACTTGGGTCAATATTGGGAAGCTCTTAGCCTCTCTGTGCTGGAAAATTCCTTGGCCA

CCACCACATATACAAGTTGGGTTGGGATGTGGCAGTGAGGCTTTACGTTCACTTCCATGGTTGTCTCCCACCATAGCTAGTACCAATGAGGAGAGAATAT

TCATCATATCTGAGGATGGCAAGGTTGACGGTGTGGTTGATCAGACGATGGATGAACAGATCGCAGCTGCAGTTTTTATGCCAGCTTGTATAGAAGCTTC

GCTTAATGATGGGATGAATGCAGGAAAAACCAGGGAGGCCTTTGCTGATCATCAGTATTCTGAGTATGACAGTAGATTGAAAGATAAGGATCAGAAAATA

TTATTTGAAGATGAGATTGATTTATCATTGTTCAACTACAAGTTCGATTCCTACACAACAGATAGTCCTGCCATTGATGCTGAAGTAGAGCTTCGTTCTA

CGGATCTTTGGCCATACTGGAAGGTTGATTGCTTGTGCAAGATAATTCCAAGACAGCTACTAGCAGAGAACGCAATGATGCTTATAAGGAACACCGTTGA

CAAGCTGATTATGAAGCATAAAGAAATACAAGTTGAAACTGAACAGATTGCGGGTGAGGATGATGTAAATGAAGGGGATCCTAGCATTCTACGCTTCCTA

CTTGCTAGCCGAGACAAGTCAGCAGACATGGAACGATCGCAGGAGCTTCTGGAGACTCTAGTTATTCTTATCGTGAAATCTGTGAAGGATCCAAGAAGTG

TTGTATGCAAAACTGCACTAATGACTTGTGCAGACATCTTTAAGGCATATGGTGCCTTAATGGTTCACTCAATCGATCCATCGCTAGTACAACAGCTGCT

TCTGACGGCTTCACAAGATAAGCGTTTTGTCTGGGAAACTGCTACAACAACTTTTATATCATTGACAAGCTGGATCTTCCCTTTACTACTGAAGCCAACA

ATGTTGCCTTACCTAAAGAACAAGAATACAGGGACCTGGCCAAAAGCGTCAAGGCTGGACGACGACGGCCGGCCCCGGCGCAAGGGCACGTTCTGGACAG

CCAGCGCGCACATCATCACCGCCGTCATCGGCTCGGGCGTGCTCTCCCTGGCCTGGGCCATCGCGCAGCTCGGCTGGGTCGCCGGCCCCGCCGCCTTGCT

CCTCTTCGCCTTCGTCACCTACTACACCGCCACGCTGCTCGCCGAGTGCTACCGCACCGGCGACCCCGACACGGGCAAGCGCAACTACACGTACATGGAC

GCCGTGCGCTCCAACCTCGGCGGCACCAAGGTCCTGCTCTGCGGCGTCATCCAGTACGCCAACCTCGTCGGCGTCGCCATCGGCTACACCATCGCGTCGT

CCATCAGCATGAAGGCCGTCAGGAGAGCCGGGTGCTTCCACGTCCACGGTCACGGCGATCCATGCAGGAGCTCCAGCACCCCGTACATGATCCTCTTCGG

CCTTGTGCAGATCCTCTTCTCCCAGATACCGGACTTCGATGAGATTTGGTGGCTCTCCATTGTCGCTGCAGTCATGTCCTTCACCTATTCTTCCATCGGA

CTGTCCCTCGGAATCGTACAGACCATCTCCAACGGCGGGTTCATGGGCAGTCTCACCAGCATCAGCTTCGGCGCCGGTGTGAGCTCCACGCAGAAGGTCT

GGCACACCCTGCAGGCCTTCGGCGACATCGCCTTCGCCTACTCCTTCTCGAACATCCTCATCGAAATCCAAGACACGATCAAAGCACCGCCGCCGTCGGA

GTCGAAGGTCATGCAGAAGGCGACGTGCGTCAGCGTGGCGACGACGACCATCTTCTACATGCTGTGCGGGTGCATGGGGTACGCGGCGTTCGGCGACAAC

GCGCCGGACAACCTCCTCACCGGCTTCGGCTTCTACGAGCCCTTCTGGCTGCTCGACGTCGCCAACGTCGCCATCGTCGTGCACCTCGTCGGCGCGTACC

AGGTGTTCTGCCAGCCCATCTTCGCCTTCGTCGAGCGCCGCGCCGCCGCGGCCTGGCCGGACAGCGCCTTCATCTCACGGGAGCTCCGCGTGGGCCCCTT

CGCGCTCAGCTTGTTCCGCCTCACGTGGCGGTCGTCGTTCGTGTGCGTCACCACCGTCGTCGCCATGCTGCTGCCGTTCTTCGGTGACGTGGTCGGGCTC

CTCGGCGCCGTCTCCTTCTGGCCACTTACCGTCTACTTCCCCGTCGAGATGTACATCAAGCATCGCCGCGTGCCCCGTGGCAGCACCAGGTGGATCTGCC

TTCAGACGCTCAGCGTCACCTGCCTCCTCGTCTCCATTGCCGCCGCCGCCGGTTCCATTGCTGACGTCATCGACGCGCTCAAGGTGTACCGGCCGTTCAG

CGGCTAA

>ZmAAAP69

ATGGCGCTCGGCGAGGGCGGGGATCACGGCGCCGCCCTGCCCCTCATCGCGGACCAGGCGAAACATGCCGCCGCCGGTGGCATCGTCCGAAGCGGGAGCA

TGTGGACGGCAGCGGCACACGTGATCACGGCGGTGATCGGGTCCGGCGTGCTGTCGCTGGCGTGGAGCATCGCGCAGCTGGGGTGGGTGGCCGGGCCGGC

CGCCATGCTCGTCTTCGCGGCCGTGACGGCGCTCCAGTCCACGCTCTTCGCCGACTGCTACCGGTCGCCGGACCCCGAGCACGGCCCTCACCGCAACCGC

ACCTACGCCAAAGCCGTGGACCGCAACCTAGGTAGCAACAGCTCGTGGGTGTGCATGCTTCTGCAGCACACGGCCCTGTTCGGCTATGGCATCGCCTACA

CCATCACGGCCTCCATCAGCTGCAGGGCGATCCTGAAGGCGAACTGCTACCACGAGCATGGGCATGACGCGCACTGCGACTACGATGGCAACTACTACAT

GCTCATCTTCGGCGGCGTCCAGCTCCTCCTCTCCTTCATACCTGACTTCCACGACATGGCGTGGCTCTCCGTCGTCGCCGCGGCCATGTCGTTCTCGTAC

GCCTTCATCGGCCTCGGCCTCGGCCTCGCAAGAACCATTGCTAACGGGACGATCAAAGGAAGCATAACAGGTGTTCGGATGAGGACGCCTATGCAGAAGG

TTTGGCGCGTCTCGCAGGCCATCGGCGACATTGCCTTCGCGTACCCCTACTCCTTGATCCTCCTGGAAATACAGGACACCCTGAAGTCACCGCCGGCCGA

GAACAAGACGATGAAGAGGGCGTCGATGATCTCGATCCTCGTCACGACATTCTTCTACCTCTGCTGCGGCTGCTTGGGCTACGCCGCCTTCGGCAGCGAC

GCGCCGGGCAACCTCCTTACCGGCTTCGGCTTGTACGGTCCGTACTGGCTCATCGACTTCGCCAACGCGTGCATCATCCTCCACCTGCTGGGCGGGTACC

AGGTGTACAGCCAGCCGATATTCCAGTTCGCGGAGCGGCTCTTGGCGGAGCGGTTCCCGGACAGCGGGTTCGTGAACGGCGGCTCCTACACCGTCCGGTT

CGCGTGCCTGCGGGCGTGCCGGGTGAACCCGCTGCGCGTGTGCCTCCGGACGCTGTACGTGGCCTCCACGACGGCGGTGGCCGTGGCGTTACCCTACTTC

AACGAGGTGCTGGCGCTGCTGGGCGCGCTCAGCTTCTGGCCGCTGGCCATCTACTTCCCCGTGGAGATGTACTTCATCCAGCGGAACGTGCGCCGGTGGT

CCGCCCGCTGGGTCGTCCTGCAGACCTTCAGCGTCGTCTGCCTGCTCGTCAGCGCCTTCGCGCTCGTCGGCTCCATCGAGGGGCTAATCAGCAAGAAGCT

AGGCTAG

>OsAAP1

ATGGGGATGGAGAGGCCGCAAGAGAAGGTGGCCACCACCACCACCGCCGCCTTCAACCTCGCCGAGTCCGGCTACGCCGA

CCGCCCCGACCTCGACGACGACGGCCGCGAGAAGCGCACAGGGACGCTGGTGACGGCGAGCGCGCACATAATAACGGCGG

TGATCGGCTCCGGCGTGCTGTCGCTGGCGTGGGCGATAGCGCAGCTGGGGTGGGTGATCGGGCCGGCCGTGCTGGTGGCG

TTCTCGGTCATAACCTGGTTCTGCTCCAGCCTCCTCGCCGACTGCTACCGATCTCCCGACCCCGTCCATGGCAAGCGCAA

CTACACCTACGGCCAAGCCGTCAGGGCCAACCTAGGTGTGGCCAAGTACAGGCTCTGCTCGGTGGCACAGTACGTCAATC

TCGTCGGCGTCACCATTGGCTACACCATCACTACGGCCATCAGCATGGGTGCGATCAAACGGTCCAACTGGTTCCATCGC

AACGGCCACGACGCAGCCTGCTTGGCATCTGACACGACCAACATGATCATATTTGCTGGCATCCAAATCCTCCTCTCGCA

GCTGCCGAATTTTCACAAAATTTGGTGGCTCTCCATTGTCGCTGCTGTCATGTCACTGGCCTACTCAACCATTGGCCTTG

GCCTCTCCATTGCAAAAATTGCAGGTGGGGCCCACCCCGAGGCAACCCTCACAGGGGTGACTGTTGGAGTGGATGTGTCT

GCAAGTGAGAAAATCTGGAGAACTTTTCAGTCACTTGGTGACATTGCCTTTGCATACTCCTACTCCAATGTCCTCATAGA

AATTCAGGACACGCTGCGGTCGAGCCCGGCGGAGAACGAGGTGATGAAGAAGGCGTCGTTCATCGGAGTCTCGACGACGA

CGACGTTCTACATGCTGTGCGGCGTGCTCGGCTACGCGGCGTTCGGCAACCGCGCGCCGGGGAACTTCCTCACCGGCTTC

GGCTTCTACGAGCCCTTCTGGCTCGTCGACGTCGGCAACGTCTGCATCGTCGTCCACCTCGTCGGCGCCTACCAGGTCTT

CTGCCAGCCCATCTACCAGTTCGCCGAGGCCTGGGCGCGCTCGCGGTGGCCGGACAGCGCCTTCGTCAACGGCGAGCGCG

TGCTCCGGCTGCCGCTCGGCGCCGGCGACTTCCCCGTCAGCGCGCTCCGCCTCGTCTGGCGCACGGCCTACGTCGTGCTC

ACCGCCGTCGCCGCCATGGCGTTCCCCTTCTTCAACGACTTCCTCGGCCTCATCGGCGCCGTCTCCTTCTGGCCGCTCAC

CGTCTACTTCCCCGTCCAGATGTACATGTCTCAGGCCAAGGTCCGGCGATTCTCGCCGACGTGGACGTGGATGAACGTGC

TCAGCCTCGCCTGCCTCGTCGTCTCCCTCCTCGCCGCCGCCGGCTCCATCCAGGGCCTCATCAAATCCGTCGCACATTAC

AAGCCATTCAGCGTCTCCTCATGA

>OsAAP2

ATGGCGTCGGTCGACTTGGAGCTGGGGCGACCTCTGTCGGCGGCGGCGGCGGCGTACCCTCCTCCGCTGCGGCGGAGCAT

CAACGACGACGACGTTGACGACGACGGGAAGCCCAAGCGAACAGGAACGGAGTGGACGGCGAGCGCGCACATCGTCACGG

CGGTGGTCGGCTCCGGCGTGCTGTCGCTGGCATGGTCCACGGCGCAGCTCGGCTGGGTCGCCGGCCCGGCCACCCTCGTC

GTGTTCGCGGTCATCACCTACTACACCTCCGTGCTCCTCGCCGACTGCTACCGCGCCGGCGGCGATCAGGTCTCCGGGAA

GAGGAACTACACATACATGGACGCCGTCGAGTCCTATCTAGGTGGTCGGCAAGTGTGGTTCTGTGGTCTCTGTCAGTACG

TTAACCTGGTTGGAACTGCAATCGGGTACACCATCACAGCATCCATCAGTGCCGCGGCGGTGTACAAGTCCAACTGCTTC

CACAAGAACGGCCACTCCGCCGACTGCAGCGTCTTCACCACCTCGTACATGGTGGTGTTCGGCGTTGTCCAGGTCTTCTT

CTCCCAGCTGCAGAGCCTCCACGAGGTGGCGTGGCTGTCCGTGCTCGCCGCCGTCATGTCCTTCTCCTACTCGGCCATCG

CCGTCGGCCTCTCCTTGGCACAAACCATATCAGGTCCTACTGGTATGACGACTATGTCTGGGACTGTAATCGGAATAGAT

GTTGATTTGTCGCACAAGATATGGCAGGCACTGCAAGCCCTCGGAAACATCGCGTTCGCATATTCCTACTCCCTGGTTCT

CATTGAAATCCAGGACACGATCAGGTCGCCGCCGGCGGAGAGCAAGACGATGAGGAAGGCGAACGCGCTGGCCATGCCGG

TGATCACGGCGTTCTACACGCTCTGCGGCTGCCTCGGCTACGCGGCGTTCGGGAACGCGGCGCCGGGGAACATGCTCACG

GGCTTCGGCTTCTACGACCCCTACTGGCTCGTCGGCCTCGCCAACGCCTGCATCGTCGTGCACCTCGTCGGCGCCTACCA

GGTGATGTCCCAGCCCGTCTTCACCGCCGTCGAGTCCTGGGCGTCCTCCCGGTGGCCCCGGTGCGGCTTCTTCGTCACCG

GCGGCGGCGGAACGAGGCTGATCAGCGTGAACGCGTTCAGGCTCGCGTGGCGCACGGCGTACGTCGTGGCGTGCACCGCG

GTCGCCGCCGTGGTGCCGTTCTTCAACGACGTGCTCGGCCTCCTCGGCGCCGTCGGGTTCTGGCCGCTCACCGTGTACTT

CCCCGTGGAGATGTACATCCGGCGGCGGAAGCTGGAGAGGTCGTCCAAGAGGTGGGTGGCGCTGCAGAGCCTCAACGCCG

TGTGCTTCGTGGTGACGCTCGCCTCAGCGGTCGCGTCCGTGCAGGGGATCGCCGAATCGATGGCGCACTATGTACCGTTC

AAGTCAAAGTTGTAA

>OsAAP3

ATGGCGAAGGACGTGGAGATGGCGGTGCGGAACGGAGACGGCGGCGGCGGCGGCGGCTACTACGCCACCCACCCGCACGG

CGGCGCCGGCGGCGAGGACGTCGACGACGACGGCAAGCAGCGGCGAACCGGTAACGTATGGACGGCGAGCGCGCACATCA

TCACGGCGGTGATCGGCTCCGGCGTGCTCTCTCTCGCATGGGCAACGGCGCAGCTCGGCTGGGTGGTCGGGCCGGTGACT

CTGATGCTCTTCGCCCTCATCACGTACTACACCTCTGGGCTCCTCGCCGACTGCTACCGCACTGGCGATCCGGTCAGCGG

CAAGCGCAACTACACCTACATGGATGCCGTTGCGGCCTACTTAGGTGGCTGGCAAGTCTGGTCCTGTGGTGTTTTCCAAT

ATGTCAACCTGGTTGGGACAGCAATTGGGTACACAATCACAGCATCCATCAGCGCAGCGGCTGTGCACAAGGCCAACTGC

TACCACAAGAACGGCCACGATGCCGATTGCGGTGTCTACGACACCACGTACATGATCGTCTTTGGAGTCGTCCAGATCTT

CTTCTCCATGCTGCCCAACTTCAGTGACCTCTCATGGCTTTCCATCCTCGCCGCGGTCATGTCATTCTCATACTCGACCA

TTGCCGTTGGCCTCTCGCTTGCGCGAACAATATCAGGTGCTACTGGTAAGACTACTCTGACTGGCGTTGAGGTTGGAGTT

GACGTCACTTCAGCCCAGAAGATCTGGCTCGCGTTCCAAGCGCTCGGTGACATCGCGTTCGCCTACTCCTACTCCATGAT

CCTTATAGAAATTCAGGACACGGTGAAGTCTCCACCGGCGGAGAACAAGACGATGAAGAAGGCAACGCTGCTGGGGGTGT

CGACCACGACGGCGTTCTACATGCTGTGCGGGTGCCTGGGGTACGCGGCGTTCGGGAACGCGGCGCCGGGGAACATGCTC

ACCGGGTTCGGCTTCTACGAGCCCTACTGGCTGATCGACTTCGCCAACGTCTGCATCGTGGTCCACCTGGTCGGCGCCTA

CCAGGTGTTCTGCCAGCCCATCTTCGCCGCCGTCGAGACGTTCGCCGCCAGGCGGTGGCCGGGCTCGGAGTTCATCACCC

GGGAGCGCCCCGTCGTGGCCGGCAGGTCGTTCAGCGTCAACATGTTCAGGCTGACGTGGCGGACGGCGTTCGTGGTCGTC

AGCACGGTGCTCGCCATCGTGATGCCCTTCTTCAACGACATCCTGGGCTTCCTCGGCGCCGTCGGGTTCTGGCCGCTGAC

GGTGTACTACCCGGTGGAGATGTACATCCGGCAGCGGCGGATACAGCGGTACACGTCCAGGTGGGTGGCGCTGCAGACGC

TCAGCCTCCTCTGCTTCCTCGTCTCGCTCGCCTCCGCCGTCGCCTCCATCGAGGGCGTCAGCGAGTCGCTCAAGCACTAC

GTCCCCTTCAAGACCAAGTCGTGA

>OsAAP4

ATGGACAGGAGAGCAGTAGTGTATGATGCTGAAGCAGTTGATGATCATGAGAGACAAGGGACGGTGTGGACGGCGACGTC

GCACATCGTGGCGGCGGTGGTCGGCTCCGGCGTGCTGGCGCTGGCGTGGACGGTGGCGCAGCTGGGGTGGGTGGTGGGGC

CCCTCGTCCTCGTTGGCTTCTCATGTGTCACTTACTACACATCTACCCTCCTCGCCAATTGCTACCGCTACCCCGACCCC

GTCACCGGCACCGCCAACCGCGAGTACATCGACGCCGTTCGCTGCTACCTCGGGCCGAAGAACGTGATGTTGTGTGGGTG

TGCGCAGTATGTCAACCTGTGGGGTACACTTGTCGGGTACACCATCACAGCGAGTGCAAGCATGATAGCGGTGAAGCGGG

TGAACTGCTTCCACCGGGAAGGGTACGGCGCCGGCGACTGCGGCGCGTCGGGGAGCACGTACATGGTGGTGTTCGGCGTC

TTCCAGCTCCTCCTCTCCCAGCTCCCCTCCCTCCACAACATCGCCTGGCTCTCCGTCGTCGCCGTCGCCACCTCCTTCGG

CTACTCCTTCATCAGCCTCGGCCTCTGCGCCGCCAAGTGGGCCTCCCACGGCGGCGCCGTCCGCGGCACCCTCGCCGGCG

CCGACCTCGACTTCCCCCGCGACAAGGCCTTCAACGTCCTCCTCGCCCTCGGCAACATCGCCTTCTCCTACACCTTCGCC

GACGTCCTCATCGAGATCCAGGACACGCTCCGCTCGCCGCCGGCGGAGAACAAGACCATGAAGAGGGCCTCCTTCTACGG

CCTCTCCATGACCACCGTCTTCTACCTCCTCCTCGGCTGCACCGGCTACGCCGCCTTCGGCAACGACGCCCCCGGCAACA

TCCTCACCGGCTTCGCCTTCTACGAGCCCTTCTGGCTCGTCGACATCGCCAACATCTGCGTCATCGTCCACCTCATCGGC

GCCTACCAAGTGTTCGCGCAGCCGATCTTCGCGAGGCTGGAGAGCTACGTGGCGTGCCAGTGGCCGGACGCCAAGTTCAT

CAACGCGACCTACTACGTGAGGGTGCCGGGGAGGTGGTGGCCGGCGGCGACGGTGGCGGTGGCGCCGCTGAAGCTGGTGC

TGCGGACGATCATCATCATGTTCACCACGCTGGTGGCGATGCTCCTCCCCTTCTTCAACGCCGTGCTGGGCCTCATCGGG

GCGCTCGGCTTCTGGCCGCTCTCCGTCTACTTCCCGGTGAGCATGCACGTCGCCCGCCTCGGCATCCGCCGCGGCGAGCC

GCGGTGGTGGTCGCTGCAGGCCATGAGCTTCGTCTGCCTCCTCATCTCCATCGCCGCCAGCATCGGCTCCGTCCAGGACA

TCGTCCACAACCTCAAGGCTGCTGCACCCTTCAAGACTGTCAACTGA

>OsAAP5

ATGAACAAGAACGCCGCACCGGAAGACGTCGAGAGCGGCGAGCACGAGCGGACAGGGACGGTATGGACGGCGACGGCGCA

CATTGTTACGGCGGTGATCGGCTCCGGCGTGCTGGCGCTGGCGTGGAGCGTGGCGCAGCTGGGTTGGGTGGCCGGGCCGC

TCGCCCTCGCCGGCTTCGCCTGCGTCACCTACTACACCTCCACTCTGCTCGCCAACGCCTACCGCGCGCCGCACCCCGTC

ACCGGCACCAGGAACCGCACATACATGGACGCCGTCAGATCATACCTCAGTCCTAGAGAGGTGTTCATGTGCGGAATCGC

GCAGTACGTCAACCTGTGGGGCACCATGGTCGGCTACACAATCACCGCAACCATAAGCATGGTCGCGATCAGGAGGTCGG

ATTGCATCCATCGGAACGGCGCCGGCGCCGCCGCGCGGTGCGACAACACGTCGGCGACGGTGCTCATGCTGGCGTTCAGC

ATCGTGCAGGTGGTGCTGTCCCAGTTCCCGGGCCTGGAGCACATCACCTGGCTGTCCGTCGTCGCCGCCGTCATGTCGTT

CGCCTACTCCTTCATCGGCCTCGGCCTGTCCGTGGCGGAGTGGGTGTCGCACGGCGGCCACCTCAGCGGCAGGATCCAGG

GCGCCACCGCGGCGTCCTCCAGCAAGAAGCTCTGGAACGTACTGCTCGCACTGGGGAACATCGCCTTCGCCTACACCTTT

GCAGAAGTGCTAATTGAGATCCAGGACACACTGAAACCGTCACCACCGGAGAACAAGACCATGAAGAAGGCAGCGATGTA

CGGGATTGGAGCCACCACCATCTTCTACATCTCCGTTGGCTGCGCCGGGTACGCCGCGTTCGGTTCAGATGCTCCGGGCA

ACATCCTGACGGCGTCCGGTATGGGGCCCTTCTGGCTCGTCGACATTGCCAACATGTGCCTCATCCTCCATCTCATCGGA

GCATATCAGGTTTATGCACAGCCCATATTTGCGACAATGGAGAGATGGATCTCCTCCCGGTGGCCGGAGGCCAAGTTCAT

CAACAGCGAGTACACCGTAAACGTGCCGCTGATCCAGCGAGGATCGGTGACCGTGGCGCCGTACAAGCTCGTCCTCCGGA

CCGTCGTAGTCATCGCGACGACGGTGGTGGCGATGATGATACCGTTCTTCAACGCGGTGCTGGGGCTCCTCGGCGCCTTC

AGCTTCTGGCCACTGACTGTTTACTTCCCCATCAGCATGCACATTGCGCAGGAGAAGATCACCAGGGGAGGGAGGTGGTA

TCTCCTGCAAGGCCTGAGCATGGTGTGCTTGATGATCTCGGTGGCAGTGGGCATTGGCTCTGTCACTGACATTGTTGATA

GCCTGAAGGTTGCAACCCCTTTCAAAACTGTCAGCTAA

>OsAAP6

ATGGACGTGGAGAAGGTGGAGAGGAAGGAGGTGGCCGTCGACGACGATGGCCGCGTCAGAACAGGAACGGTATGGACGGC

GACGACGCACGCCATCACCGCCGTGATCGGGTCAGGCGTGCTGGCGCTGCCGTGGAGCGTGGCCCAGATGGGGTGGGTGC

TCGGCCCCATCGCCCTCGTCGTCTGCGCCTACATCACCTACTACACCGCCGTCCTCCTCTGCGACTGCTACCGCACGCCG

GACCCCGTCCACGGCAAGCGGAACTACACCTACATGGACGTCGTCCGCTCATGCCTCGGGCCTCGAGACGTGGTCGTGTG

TGGCATTGCGCAGTACGCGATTCTCTGGGGCGCAATGGTGGGTTACACCATCACGACCGCTACGAGCATCATGTCAGTGG

TGCGCACGAACTGCCACCACTACAAGGGGCCGGACGCGACCTGCGGCTCGTCCGGGACGATGTACATGGTGCTGTTCGGC

CTCGCGGAGGTCGTCCTGTCCCAGTGCCCGAGCCTGGAGGGGGTGACGCTCATCTCCGTCGTCGCCGCCGTCATGTCGTT

CACCTACTCCTTCGTCGGGCTCTTCCTCAGCGCCGCTAAGGTCGCGTCGCACGGCGCGGCGCACGGCACCCTCCTCGGCG

TCAGGGTCGGCGCCGGAGGCGTCACCGCGTCGACCAAGGCGTGGCACTTCCTGCAGGCGCTCGGGAACATCGCCTTCGCG

TACACCTACTCCATGCTGCTCATCGAGATCCAGGACACGGTGAAGTCGCCGCCGTCGGAGAACGTGACGATGAAGAGGGC

GAGCCTGTACGGCATCGGCGTCACGACCGTCTTCTACGTGTCGATCGGGTGCGTCGGGTACGCGGCGTTCGGCAACGCCG

CGCCCGGGAACGTCCTCACCGGCTTCCTCGAGCCGTTCTGGCTCGTCGACATCGCCAACGTTGCCGTCGTCATCCACCTG

GTCGGAGCGTACCAGGTGTACGCGCAGCCGGTGTTCGCGTGCTACGAGAAGTGGCTGGCGAGCCGTTGGCCGGAGTCGGC

GTTCTTCCACCGGGAGTACGCGGTGCCGCTGGGCGGCGGGCGCGCGGTGCGGTTCACGCTGTGCAAGCTGGTGCTGCGCA

CGGCGTTCGTGGCCGTGACGACGGTGGTGTCGCTGGTGCTGCCGTTCTTCAACGCCGTGCTCGGGCTGCTCGGCGCCGTC

GCGTTCTGGCCGCTCACGGTGTACTTCCCGGTGACCATGTACATGGCGCAGGCGAAGGTGCAGAGAGGCAGCCGGAAGTG

GGTGGCGCTGCAGGCGCTCAACGTCGGCGCGCTCGTCGTGTCGCTGCTCGCGGCGGTGGGCTCGGTGGCCGACATGGCGC

AGCGCCTGCGCCACGTCACCATCTTCCAAACGCAGCTCTGA

>OsAAP7

ATGGGGGAGAACGGTGTGGTGGCGAGCAAGCTGTGCTACCCGGCGGCGGCCATGGAGGTGGTCGCCGCCGAGCTCGGCCA

CACGGCCGGCTCCAAGCTGTACGACGACGACGGCCGCCTCAAGCGCACCGGGACGATGTGGACGGCGAGCGCGCACATCA

TCACGGCGGTGATCGGCTCCGGCGTGCTGTCGCTGGGGTGGGCGATCGCGCAGCTGGGTTGGGTGGCCGGCCCCGCCGTC

ATGCTGCTCTTCTCGTTCGTCACCTACTACACCTCCGCGCTGCTCGCCGACTGCTACCGCTCCGGCGACGAGAGCACCGG

CAAGCGCAACTACACCTACATGGACGCCGTGAACGCCAACCTGAGTGGCATCAAGGTCCAGGTCTGCGGGTTCCTGCAGT

ACGCCAACATCGTCGGCGTCGCCATCGGCTACACCATTGCCGCCTCCATTAGCATGCTGGCGATCAAGCGGGCGAACTGC

TTCCACGTCGAGGGGCACGGCGACCCGTGCAACATCTCGAGCACGCCGTACATGATCATCTTCGGCGTGGCGGAGATCTT

CTTCTCGCAGATCCCGGACTTCGACCAGATCTCGTGGCTGTCCATCCTCGCCGCCGTCATGTCGTTCACCTACTCCACCA

TCGGGCTCGGCCTCGGCGTCGTGCAGGTGGTGGCCAACGGCGGCGTCAAGGGGAGCCTCACCGGGATCAGCATCGGCGTG

GTGACGCCCATGGACAAGGTGTGGCGGAGCCTGCAGGCGTTCGGCGACATCGCCTTCGCCTACTCCTACTCCCTCATCCT

CATCGAGATCCAGGACACCATCCGGGCGCCGCCGCCGTCGGAGTCGAGGGTGATGCGGCGCGCCACCGTGGTGAGCGTCG

CCGTCACCACGCTCTTCTACATGCTCTGCGGCTGCACGGGGTACGCGGCGTTCGGCGACGCCGCGCCGGGCAACCTCCTC

ACCGGGTTCGGCTTCTACGAGCCCTTCTGGCTCCTCGACGTTGCCAACGCCGCCATCGTCGTCCACCTCGTCGGCGCCTA

CCAGGTCTACTGCCAGCCGCTGTTCGCCTTCGTCGAGAAGTGGGCGCAGCAGCGGTGGCCGAAATCATGGTACATCACCA

AGGATATCGACGTGCCGCTCTCCCTCTCCGGCGGCGGCGGCGGCGGCGGAAGGTGCTACAAGCTGAACCTGTTCAGGCTG

ACATGGAGGTCGGCGTTCGTGGTGGCGACGACGGTGGTGTCGATGCTGCTGCCGTTCTTCAACGACGTGGTGGGGTTCCT

CGGCGCGGTGGGGTTCTGGCCGCTCACCGTCTACTTCCCGGTGGAGATGTACATCGTGCAGAAGAGGATACCGAGGTGGA

GCACGCGGTGGGTGTGCCTGCAGCTGCTCAGCCTCGCCTGCCTCGCCATCACCGTCGCCTCCGCCGCCGGCTCCATCGCC

GGAATCCTCTCCGACCTCAAGGTCTACAAGCCGTTCGCCACCACCTACTAA

>OsAAP8

ATGGGGGAGAACGTGGTTGGCACGTACTACTACCCGCCTTCGGCGGCCGCCATGGACGGCGTGGAGCTCGGCCACGCCGC

CGCCGGCTCCAAGCTCTTCGACGACGACGGCCGCCCCAGGCGCAACGGGACGATGTGGACGGCGAGCGCGCACATCATCA

CGGCGGTGATCGGCTCCGGCGTGCTGTCGCTGGGGTGGGCCATCGCGCAGCTCGGCTGGGTGGCCGGGCCGGCGGTCATG

GTGCTCTTCTCCCTCGTCACCTACTACACCTCATCCCTCCTCTCCGATTGCTACCGCTCCGGCGACCCCGTCACCGGCAA

GCGGAACTACACCTACATGGACGCCGTGAACGCCAACCTGAGCGGGTTCAAGGTGAAGATCTGCGGGTTCTTGCAGTACG

CCAACATCGTCGGCGTCGCCATCGGCTACACCATCGCGGCGTCCATCAGCATGCTGGCGATCGGGAGGGCCAACTGCTTC

CACAGGAAGGGGCACGGCGACCCGTGCAACGTCTCCAGCGTGCCCTACATGATCGTCTTCGGCGTCGCCGAGGTCTTCTT

CTCGCAGATCCCCGACTTCGATCAGATCTCCTGGCTCTCCATGCTCGCCGCCGTCATGTCCTTCACCTACTCCGTCATCG

GCCTCAGCCTCGGCATCGTCCAAGTCGTCGCGAACGGAGGGTTGAAGGGAAGCCTGACCGGGATCAGCATCGGCGTGGTG

ACGCCGATGGACAAGGTGTGGAGGAGCCTGCAGGCGTTCGGCGACATCGCGTTCGCCTACTCCTACTCGCTGATCCTCAT

CGAGATCCAGGACACCATCCGGGCGCCGCCGCCGTCGGAGTCGGCGGTGATGAAGCGCGCCACGGTGGTGAGCGTGGCGG

TGACCACGGTGTTCTACATGCTCTGCGGCAGCATGGGGTACGCGGCGTTCGGCGACGACGCGCCGGGGAACCTCCTCACC

GGGTTCGGCTTCTACGAGCCCTTCTGGCTCCTCGACATCGCCAACGCCGCCATCGTCGTCCACCTCGTCGGCGCCTACCA

GGTGTTCTGCCAGCCGCTCTTCGCCTTCGTCGAGAAGTGGGCGGCGCAGCGGTGGCCGGAGTCGCCGTACATCACCGGGG

AGGTGGAGCTCCGCCTCTCGCCGTCGTCGAGGCGGTGCAGGGTGAACCTGTTCCGGTCGACGTGGCGCACGGCGTTCGTC

GTCGCCACCACGGTGGTGTCCATGCTGCTGCCCTTCTTCAACGACGTGGTCGGCTTCCTCGGCGCGCTCGGATTCTGGCC

GCTCACCGTCTACTTCCCCGTGGAGATGTACGTGGTGCAGAAGAAGGTGCCACGGTGGAGCACACGGTGGGTGTGCCTGC

AGATGCTCAGCGTCGGCTGCCTCGTCATCTCCATCGCCGCCGCCGCGGGCTCCATCGCCGGCGTCATGTCGGATCTCAAG

GTTTACCGCCCGTTCAAGGGTTACTGA

>OsAAP9

ATGTTGCCGAGAAGCCGAACGCTCCCGCCAAGGATTCATGATGGCGTCGTGGTGGTAGAACGCGATGTGAGGAGGTACCA

GCAGCTGCCGCAGCAGGTGGAGATGGAGATGACGACGACCAAGAGGCAGCAGGATCATCAGGTGGAGACGATGACGACGA

AGAAGATTGATGAAGAAGATGAAGAGGTGGACGACGACGGTCGGGCGAAGCGGAGGGGGACGGTGTGGACGGCGGCGTCG

CACATCATCACGGCGGTGATCGGGTCAGGTGTGCTGTCGCTGGCGTGGGCGATCGCGCAGCTGGGCTGGGTGGTGGGCCC

CACCGTCATGCTCCTCTTTGCTGCCGTCATCTACTTCACCTCCAACCTCCTCGCCGACTGCTACCGCACCGGCGACCCCG

CCACCGGCAGAAGGAACTACACCTACATGGACGCCGTCAAGGCCAACCTCGGCGGTGCCAAGGTGAAGGTTTGCGGATGC

ATTCAGTACCTCAACCTTTTGGGAGTGGCCATCGGTTACACCATCGCCGCCTCCATTAGCATGATGGCGATCCAGCGTTC

CAACTGCTTCCACGCGAGAGGAGAGCAGGATCCATGCCACGCCTCCAGCAACGTGTACATGATCATGTTCGGCATCGTCC

AGGTGTTCTTCTCCCAGATCCCCGACTTCGACCAAGTCTGGTGGCTCTCCATCCTCGCCGCCGTCATGTCCTTCACCTAC

TCCGCCGTCGGCCTCGCCCTCGGCGCCGCCCAGGTCGCCCAGAACCGCACGTTCGCCGGCAGCGCCATGGGCGTCGCCGT

GGGCTTCGTCACCAAGACCGGCGACGTCGTCACCCCCGCGCAGAAGGTGTGGCGCAACCTGCAGGCGCTGGGGGACATCG

CCTTCGCCTACTCCTACTCCATCATCCTCATCGAGATCCAGGACACGCTGCGGTCGCCGCCGGCGGAGGCGAGGACGATG

CGGAAGGCGACGGGGATCAGCGTGGTGGTGACGAGCGTGTTCTACCTGCTGTGCGGATGCATGGGGTACGCGGCGTTCGG

CGACGACGCGCCGGGGAACCTCCTCACCGGCTTCGGCTTCTACAAGCCCTACTGGCTGCTGGACGTGGCCAACATGGCGA

TCGTGGTACACCTGGTGGGGGCGTACCAGGTGTACTGCCAGCCGCTCTTCGCCTTCGTGGAGAGGAGGGCGGAGCGGCGG

TGGCCCAACGGCCTCCCCGGCGGCGACTACGACCTGGGGTGGATAAAGGTGAGCGTGTTCAGGTTGGCGTGGCGGACGTG

CTTCGTGGCGGTGACGACGGTGGTGGCGATGCTGCTGCCCTTCTTCAACGACGTGGTGGGCATCCTGGGGGCGCTCGGCT

TCTGGCCGCTCACCGTCTACTTCCCCGTCGAGATGTACATCGCCCACCGCCGCATCCGGAGGTGGACAACCACCTGGGTC

GGCCTGCAGGCGCTCAGCCTCGCCTGCCTTCTCGTCTCGCTCGCCGCCGCCGTCGGCTCCATCGCCGGCGTCCTGCTCGA

CCTCAAGTCCTACCGTCCATTCCGCTCCACCTACTAA

>OsAAP10

ATGGGAGGAGCAGGGGAAGGCGACGGCCAGACGGAACCGCTCCTCGAGAAGCTCTCGAATTCCTCGTCGTCGGAGATTGA

CAAGAGAACAGGAACGGCATGGACGGCGACGGCGCACATCATAACGGCGGTGATCGGCTCCGGCGTGCTGTCGCTGGCGT

GGAGCGTGGCGCAGCTCGGCTGGGTCGGAGGACCGGCGGCCATGGTGCTCTTCGCCGGAGTGACCCTAGTCCAGTCCTCC

CTGCTCGCCGACTGCTACATTTTTCATGACCCGGACAACGGCGTCGTCAGGAACAGGTCCTACGTGGACGCTGTGAGGTT

TTACCTAGGTGAGAAGAGCCAGTGGTTCTGTGGCTTTTTCCTCAACATCAACTTTTTCGGGAGTGGGGTGGTGTACACAC

TCACCTCGGCCACCAGCATGAGGGCGATTCAGAAGGCAAACTGCTACCACCGGGAAGGGCACGACGCGCCGTGCTCCGTC

GGCGGAGACGGCTACTACATGCTCATGTTCGGGCTCGCGCAGGTGGTGCTCTCGCAGATACCGGGCTTCCATGACATGGC

GTGGCTCTCCGTCCTGTCGGCGGCCATGTCCTTCACCTACTCCCTCATCGGCTTCGGCCTCGGCGTCGCCAAAGTCATAA

CTAATGGAGTGATCAAGGGAGGAATCGGGGGGATCGCCATGGTGTCCGCGACGCAGAAGGTGTGGCGAGTCTCGCAGGCG

ATCGGGGACATCGCGTTCGCCTACCCCTTCGCGTCGGTTCTGCTGGAAATCGAGGACACGCTGAGGTCGCCGCCGCCGGA

GAGCGAGACGATGAGGACGGCGTCGAGGGCGAGCATCGCGGTGACCACCTTCTTCTACCTCTGCTGCGGGTGCTTCGGCT

ACGCGGCCTTCGGCGACGCCACGCCCGGTAACCTCCTCACCGGCTTCGGCTTCTACGAGCCCTACTGGCTCATCGACTTC

GCCAACCTCTGCGTCGCCGTCCACCTCCTCGGCGGCTATCAGGTGTACTCGCAGCCAGTGTTCGCGGCGGTGGAGCGGCG

GATGGGCGGCGCGGGGGCGGGCGTGGTGGAGGTGGCGGTGCCGGCGGCGGTGGCGTGGCCGTCGCGGTGGCGGCGCGGCT

GCCGCGTGAACGTGTACAGGCTGTGCTTCCGGACGGCGTACGTGGCGGCGACGACGGCGCTGGCCGTGTGGTTCCCCTAC

TTCAACCAGGTGGTCGGGCTGCTCGGCGCCTTCACCTTCTGGCCGCTGTCCATCCACTTCCCCGTCGAGATGTACCTCGT

GCAGAAGAAGGTGGCGCCGTGGACGCCGCGGTGGCTCGCCGTCCGCGCCTTCAGCGCCGCCTGCCTCGCCACCGGCGCCT

TCGCCTCCGTCGGCTCCGCCGTCGGTGTGTTCTCCTCCAAGACCAGCTAA

>OsAAP11

ATGGGGAAGGCGGCGGCGATGGAGGTGTCGGCGTCGGCGGCGGCGGAGGCGGGGATGATGGTGGGTCATGGGGAGTGGCG

CGACGACGACGGGCGGGCGCGGCGGATGGGGACGGTGTGGACGGCGAGCGCGCACATCATCACGGCGGTGATCGGCTCCG

GCGTGCTGTCGCTGGCGTGGGCGATCGCGCAGCTCGGCTGGGTGGCCGGCCCCGCCGTCATGCTGCTCTTCGCCTTCGTC

ATCTACTACACCTCCACGCTGCTCGCCGAGTGCTACCGCTCCGGCGACCCGTGCACCGGCAAGCGCAACTACACCTACAT

GGACGCCGTCCGCGCCAACCTCGGCGGCTCCAAGGTCCGCCTCTGCGGCGTCATCCAGTACGCCAACCTCTTCGGTGTCG

CCATCGGCTACACCATCGCCGCCTCCATCAGCATGCTGGCGATCAAGAGGGCGGATTGCTTCCACGAGAAGGGGCACAAG

AACCCGTGCAGGAGCTCGAGCAACCCGTACATGATCCTGTTCGGCGTCGTGCAGATCGTGTTCTCACAGATCCCGGATTT

CGATCAGATATGGTGGCTGTCCATCGTCGCCGCCATCATGTCCTTCACCTATTCGACGATTGGCCTCTCGCTCGGCATCG

CGCAGACCGTCGCCAACGGCGGGTTCATGGGCAGCCTCACCGGCATTAGCGTCGGCACCGGCGTCACCTCCATGCAGAAG

GTCTGGCGCAGCCTCCAGGCCTTCGGTGACATCGCGTTCGCCTACTCCTACTCCATCATCCTCATCGAGATCCAGGACAC

GATCAAGGCGCCGCCGCCATCGGAGGCGAAGGTGATGAAGCGCGCGACGATGGTGAGCGTGGCGACGACGACGGTGTTCT

ACATGCTGTGCGGGTGCATGGGGTACGCGGCGTTCGGGGACAAGTCGCCGGACAACCTGCTCACCGGGTTCGGCTTCTAC

GAGCCGTTCTGGCTGCTCGACGTCGCCAACGCTGCCATCGTCGTGCACCTCGTCGGCGCCTACCAGGTGTTCGTCCAGCC

GATCTTCGCGTTCGTCGAGCGGTGGGCGGCGGCGAGGTGGCCGGACGGCGGCTTCATCTCCCGGGAGCTCCGCGTGGGCC

CCTTCTCGCTCAGCGTGTTCCGCCTGACATGGCGCACGGCGTTCGTCTGCGCCACCACCGTCGTGTCCATGCTCCTCCCG

TTCTTCGGCGACGTGGTGGGGCTCCTCGGCGCCGTCTCGTTCTGGCCGCTCACCGTCTACTTCCCCGTCGAGATGTACAT

CGCGCAGCGCGGCGTGCGGCGAGGGAGCGCGCGGTGGCTCTGCCTCAAGGTCCTCAGCGCCGCCTGCCTCGTCGTCTCCG

TCGCCGCCGCCGCCGGCTCCATCGCCGACGTGGTCGACGCGCTCAAGGTGTACCGGCCGTTCAGCGGGTAG

>OsAAP12

ATGGTTCAGATCGAGCCGCTCGAGGTGTCACTGGAGGCCGGCAACCAGGCGGACTCGGCGTTGCTCGACGACGACGGGCG

GCCGCGCCGCACCGGCACGTTCTGGACGGCGAGCGCGCACATCATCACCGCCGTGATCGGGTCCGGGGTGCTGTCGCTGC

CGTGGGCGACGGCGCAGCTCGGGTGGGTCGGCGGGCCCGCCGTGATGGTGGTGTTCGGCGGCGTCACCTACTTCACCGCC

ACGCTCCAGGCCGAGTGCTACCGCACCGGCGACGAGGAGACCGGCGCCCGCAACTACACCTACATCGGCGCCGTCCGCGC

CATCCTCGGCGGCGCCAACGCCAAGCTCTGCGGCATCATCCAGTACGCCAACCTCGTCGGCACCGCCGTCGGCTACACCA

TCGCCGCCTCCATCAGCATGCAGGCCATCAAGAGGGCGGGCTGCTTCCACGCCAATGGCCACAACGTCCCGTGCCACATC

TCGAGCACCCCGTACATGCTCATCTTCGGCGCCTTCGAGATCGTCTTCTCGCAGATCCCCGACTTCCACGAGATCTGGTG

GCTGTCCATCGTCGCCGCCGTCATGTCCTTCACCTACTCCGGCGTCGGCCTCGGCCTCGGCATCGCCCAGACCGTCGCCG

ACGGCGGGTTCCGCGGCACGATCGCCGGCGTCACCAACGTCACCGCCACGCAGAAGGCGTGGCGGTCGCTGCAGGCGCTG

GGCAACATCGCCTTCGCGTTCGCCTTCTCCAACGTGTACACCGAGATCCAGGACACGATCAAGGCGCCGCCGCCGTCGGA

GGCGAAGGTGATGAAGCAGGCGTCGCTGCTGAGCATCGTGGCGACGTCGGTGTTCTACGCGCTGTGCGGGTGGATGGGCT

ACGCGGCGTTCGGCAACGCGGCGCCGGACAACCTCCTCACCGGATTCGGCTTCTTCGAGCCGTTCTGGCTCGTCGACGCC

GCCAACGTCGCCATCGCCGTGCACCTCATCGGCGCCTACCAGGTGTACTGCCAGCCGGTCTTCGCCTTCGTCGAGCGCAA

GGCGTCGCGCCGGTGGCCCGACAGCGGCTTCGTCAACAGCGAGCTCCGGGTGTGGCCCTTCGCCATCAGCGCGTTCCGGC

TGGCGTGGCGCTCGGTGTTCGTCTGCTTCACCACCGTGGTCGCCATGGCGCTGCCCTTCTTCGGCGTCATCGTCGGCCTC

CTCGGTGCCATCTCCTTCTGGCCGCTCACCGTCTACCTCCCCACGGAGATGTACATCGCGCAGCGCGGCGTGCGGCGCGG

CAGCGCACTGTGGATCGGGCTCAGGGCGCTCGCCGTCGCCGGCTTCGTCGTGTCGGCCGCCGCCACCACGGGCGCCGTCG

CCAACTTCGTCGGCGACTTCATGAAATTCCGTCCCTTCAGTGGATAG

>OsAAP13

ATGGCGCTCGGCGACGGGGACGACGGCGCGGCCGCCGCCGCCGTTCCTCTCATCTCGGATCGCCCCAAGCATGCCGCCAT

CGTCAGAAGCGGGACGGAGTGGACGGCGGCGGCGCACGTGATCACGGCGGTGATCGGGTCCGGGGTGCTGTCGCTGGCGT

GGAGCGTGGCGCAGCTGGGGTGGCTGGCGGGGCCGGGGATGATGCTCGTGTTCGCGGCCGTGACGGCGCTGCAGTCGGCG

CTGTTCGCCGACTGCTACCGCTCGCCGGACCCGGAGGTCGGCCCGCACCGCAACCGCACCTACGCCAACGCCGTCGAGCG

CAACCTAGGTAGCTCGAGCGCGTGGGTCTGCTTGTTGCTGCAGCAAACGGCCTTGTTCGGCTACGGCATCGCCTACACCA

TCACCGCCTCCATCAGTTGCAGGGCGATCCTGAGGTCCAACTGCTACCACACGCACGGCCACGACGCGCCCTGCAAATAC

GGGGGTAGCTACTACATGCTCATGTTCGGCGCGGCGCAGCTGTTCCTCTCCTTCATACCGGACTTCCACGACATGGCGTG

GCTGTCGGTCCTCGCCGCGGTCATGTCGTTCTCCTACTCGTTCATCGGCCTCGGCCTCGGCCTCGCCAACACAATTGCTA

ATGGAACGATCAAAGGAAGCATAACAGGTGCTCCAACGAGAACCCCTGTGCAGAAGGTCTGGCACGTCTCGCAGGCCATC

GGCGACATCGCATTCGCGTACCCGTACTCATTAATCCTCTTGGAAATTCAGGACACACTGAAGGCTCCACCGGCCGAGAA

CAAGACGATGAAGAAGGCGTCCATCATCTCGATCGTCGTCACCACCTTCTTCTACCTCTGCTGCGGCTGCTTCGGCTACG

CCGCCTTCGGGAGCGACGCCCCTGGCAACCTCCTCACCGGCTTCGGCTTCTACGAGCCCTACTGGCTCATCGACTTCGCC

AACGCCTGCATCATCCTCCACCTGCTCGGCGGCTACCAGGTGTACAGCCAGCCGATATACCAGTTCGCGGACAGGTTCTT

CGCGGAGAGGTACCCGGCGAGCCGGTTCGTGAACGACTTCCACACGGTGAAGCTGCCGCTGCTGCCGCCGTGTCGGGTGA

ACCTCCTGCGGGTGTGCTTCCGGACGGTGTACGTGGCGAGCACCACGGCGGTGGCGCTCGCCTTCCCCTACTTCAACGAG

GTGCTCGCGCTGCTCGGCGCCCTCAACTTCTGGCCGCTCGCCATCTACTTCCCCGTGGAGATGTACTTCATCCAGCGCCA

TGTCCCGCGGTGGTCGCCCCGGTGGGTCGTGCTGCAGTCGTTCAGCGTCCTCTGCCTCCTCGTCAGCGCCTTCGCGCTCG

TCGGCTCCATCCAGGGCCTCATCAGCCAGAAGCTAGGCTAA

>OsAAP14

ATGGCGCCGCAGCTGCCGCTCGAGGTGGCCTCTGCTCCCAAGCTCGACGACGACGGCCACCCACAACGCACCGGGAATCT

ATGGACGTGCGTAGCGCACATTATCACCGCGGTGATCGGGTGCGGCGTGCTGGCGCTGTCGTGGAGCGTCGCGCAGCTCG

GCTGGGTGGCCGGCCCCATCGCCATGGTGTGCTTCGCCTTCGTCACCTACATCTCGGCCTTCCTGCTGTCGCACTGCTAC

AGGTCTCCTGGCTCGGAGAAGATGCAGAGGAACTACTCCTACATGGACGCCGTCAGAGTTCACTTAGGGAGGAAGCACAC

TTGGTTATGTGGGTTGCTGCAGTACCTGAACTTGTACGGGATAGGAATTGCTTACACAATCACTACGGCAACTTGTATGA

GGGCAATTAAGAGGGCGAACTGCTACCACAGCGAAGGCCGTGACGCTCCCTGCGACTCGAACGGTGAACACTTCTACATG

CTGCTCTTCGGAGCAGCCCAGCTGCTGCTGTCCTTCATACCCAATTTCCACAAGATGGCGTGGCTGTCCGTCGTCGCGGC

GATCATGTCCTTCGCCTACTCCACCATCGGCCTCGGCCTCGGCCTCGCCAAGACCATTGGTGATGGAACTGTCAAAGGGA

ACATTGCCGGTGTTGCGATGGCCACCCCAATGCAGAAAGTTTGGCGAGTGGCTCAAGCAATTGGCGACATCGCATTCGCC

TACCCGTACACCATCGTTCTCCTGGAGATACAGGACACGCTGAGATCGCCACCGCCGGAGAGCGAGACGATGCAGAAGGG

CAACGTGATCGCGGTCCTCGCCACCACCTTCTTCTACCTCTGCGTCGGCTGCTTCGGCTACTCCGCCTTCGGCAACGCCG

CGCCGGGCAACCTCCTCACCGGCTTCGGCTTCTACGAGCCCTACTGGCTCATAGACTTCGCCAATGCCTGCATCGTGCTC

CACCTCCTCGGCGGCTACCAGATGTTTAGCCAGCAGATATTCACGTTCGCCGACCGGTGCTTCGCGGCGAGCTTCCCGAA

CAGCGCGTTCGTGAACAGGTCGTACTCCGTCAAGATCCTCCCGTGGCGGCGCGGCGGCGGCGGCGGCGGCGCGGGGAGGT

ACGAGGTGAACCTGCAGCGGGTGTGCTTCAGGACGGTGTACGTGGCGAGCACGACGGGGCTGGCGCTGGTGTTCCCCTAC

TTCAACGAGGTGCTGGGCGTGCTCGGCGCGCTCGTGTTCTGGCCGCTCGCCATCTACCTCCCCGTCGAGATGTACTGCGT

GCAGCGGCGGATCTCGCCGTGGACGCCGCGGTGGGCCGCGCTGCAGGCGTTCAGCGTCGTCTGCTTCGTCGTCGGCACGT

TCGCGTTCGTCGGCTCGGTGGAAGGCGTCATCAGAAAGAGGCTTGGCTAG

>OsAAP15

ATGGCGTCGGGGCAGAAGGTGGTGAAGCCGATGGAGGTGTCGGTGGAGGCCGGGAACGCCGGGGAGGCGGCGTGGCTGGA

CGACGACGGGCGGGCGCGGCGGACGGGCACGTTCTGGACGGCGAGCGCGCACATCATCACCGCCGTCATCGGCTCCGGCG

TGCTGTCGCTGGCGTGGGCGATCGCGCAGCTGGGCTGGGTGGCCGGCCCCGCCGTGATGCTCCTCTTCGCCTTCGTCATC

TACTACACCTCCACCCTCCTCGCCGAGTGCTACCGCACCGGCGACCCTGCCACCGGCAAGCGCAACTACACCTACATGGA

CGCCGTGCGCGCCAACCTCGGCGGCGCCAAGGTCACCTTCTGCGGCGTCATCCAGTACGCCAACCTCGTCGGCGTCGCCA

TCGGCTACACCATCGCGTCGTCCATCAGCATGCGCGCCATCAGGAGGGCCGGCTGCTTCCACCACAACGGCCATGGTGAC

CCGTGCCGCAGCTCCAGCAACCCTTACATGATCCTCTTCGGCGCCGTGCAGATCGTCTTCTCGCAGATCCCTGACTTCGA

CCAGATTTGGTGGCTGTCCATCGTCGCCGCCGTCATGTCCTTCACCTACTCCGGCATCGGCCTCTCCCTCGGCATCGTCC

AGACAATCTCCAATGGCGGGATCCAGGGCAGCCTCACCGGAATCAGCATCGGCGTCGGCGTCAGCTCAACGCAGAAGGTG

TGGCGCAGCCTGCAGGCATTCGGCGACATCGCCTTCGCATACTCCTTCTCCAACATCCTCATCGAGATCCAAGACACGAT

CAAGGCGCCGCCGCCGTCGGAGGCGAAGGTGATGAAGAGCGCGACGAGGCTGAGCGTGGCGACGACCACGGTGTTCTACA

TGCTGTGCGGGTGCATGGGCTACGCGGCGTTCGGCGACGCGGCGCCCGACAACCTCCTCACCGGCTTCGGGTTCTACGAG

CCCTTCTGGCTGCTCGACGTCGCCAACGTCGCCATCGTCGTGCACCTCGTCGGCGCCTACCAGGTGTTCGTCCAGCCAAT

CTTCGCCTTCGTCGAGCGCTGGGCCTCCCGCCGGTGGCCGGACAGCGCGTTCATCGCCAAGGAGCTCCGCGTGGGGCCCT

TCGCGCTCAGCCTCTTCCGCCTGACGTGGCGCTCGGCGTTCGTCTGCCTCACCACCGTCGTCGCCATGCTCCTCCCCTTC

TTCGGCAACGTGGTGGGTCTCCTCGGCGCCGTCTCCTTCTGGCCGCTCACCGTCTACTTCCCCGTCGAGATGTACATCGC

GCAGCGCGGCGTGCCACGTGGCAGCGCGAGGTGGGTCTCACTCAAGACGCTCAGCGCGTGCTGCCTCGTCGTCTCCATCG

CCGCCGCCGCGGGCTCCATTGCTGACGTCATCGACGCGCTCAAGGTGTACAGGCCGTTCAGCGGATGA

>OsAAP16

ATGGCGTCGGGGCAGAAGGTGGTGAAGCCGATGGAGGTGTCGGTGGAGGCCGGGAACGCCGGGGAGGCGGCGTGGCTGGA

CGACGACGGGCGGGCGCGGCGGACGGGCACGTTCTGGACGGCGAGCGCGCACATCATCACCGCCGTCATCGGCTCCGGCG

TGCTGTCGCTGGCGTGGGCGATCGCGCAGCTGGGCTGGGTGGCCGGTCCCGCCGTGATGCTCCTCTTCGCCTTCGTCATC

TACTACACCTCCACCCTCCTCGCCGAGTGCTACCGCACCGGCGACCCGGCCACCGGCAAGCGAAACTACACCTACATGGA

CGCCGTGCGCGCCAACCTCGGCGGCGCCAAGGTCACCTTCTGCGGCGTCATCCAGTACGCCAACCTCGTCGGCGTCGCCA

TCGGCTACACCATCGCGTCGTCCATCAGCATGCGCGCCATCAGGAGGGCCGGCTGCTTCCACCACAACGGCCATGGTGAC

CCGTGCCGCAGCTCCAGCAACCCTTACATGATCCTCTTCGGCGTCGTGCAGATCGTCTTCTCGCAGATCCCGGACTTCGA

CCAGATTTGGTGGCTGTCCATCGTCGCCGCCGTCATGTCCTTCACCTACTCCGGCATCGGCCTCTCCCTCGGCATCGTCC

AGACAATCTCCAATGGCGGGATCCAGGGCAGCCTCACCGGAATCAGCATCGGCGTCGGCGTCAGCTCAACGCAGAAGGTG

TGGCGCAGCCTGCAGGCATTCGGCGACATCGCCTTCGCATACTCCTTCTCCAACATCCTCATCGAGATCCAAGACACGAT

CAAGGCGCCGCCGCCGTCGGAGGCGAAGGTGATGAAGAGCGCGACGAGGCTGAGCGTGGCGACGACCACGGTGTTCTACA

TGCTGTGCGGGTGCATGGGCTACGCGGCGTTCGGCGACGCGGCGCCCGACAACCTCCTCACGGGCTTCGGCTTCTACGAG

CCCTTCTGGCTGCTCGACGTCGCCAACGTCGCCATCGTCGTGCACCTCGTCGGCGCCTACCAGGTGTTCGTCCAGCCAAT

CTTCGCCTTCGTCGAGCGCTGGGCCTCCCGCCGGTGGCCGGACAGCGCGTTCATCGCCAAGGAGCTCCGCGTGGGGCCCT

TCGCGCTCAGCCTCTTCCGCCTGACGTGGCGCTCGGCGTTCGTCTGCCTCACCACAGTCGTCGCCATGCTCCTCCCCTTC

TTCGGCAACGTGGTGGGTCTCCTCGGCGCCGTCTCCTTCTGGCCGCTCACCGTCTACTTCCCCGTCGAGATGTACATCGC

GCAGCGCGGCGTGCCACGTGGCAGCGCGAGGTGGGTCTCGCTCAAGACGCTCAGCGCGTGCTGCCTCGTCGTCTCCATCG

CCGCCGCTGCGGGCTCCATTGCTGACGTCATCGACGCTCTCAAGGTGTACAGACCGTTCAGCGGATGA

>OsAAP17

ATGGCTCACACGAGCTCGCAAAAGCACGGCAACGACGACGTCGACACCGGCGCGGAGGCGGCAATGGACCAGCTCGCCGG

GAGGTCGTCGTTGTCACCGGCGCGGGAGAAGACGAGGAGGCGGCCGGAGAAGAGCGGCACGGTGTGGACGGCGACGGCGC

ACATCGTCGCGCTCCTGATCGGGTCCAGCGTCCTGGCGGTGGCGTGGACCTTCGCCCAGCTCGGGTGGGTCGCCGGCCCC

GCCGTCGTCGTCGCCCTCTCCGTCGTCACCTACTACTCCTCGGCCCTCCTCGCCGACTGCTACCGCGACGACGACCCTCT

TCACCTCGGCGGCGGCGCCGTCCATGGCGAGTACATCGCCGCCGTCCGCTCCTACCTTGGTCCGAAAAGCGTGACATTCT

GCGGCATCATCCAGTACGGCGTTCTCTGGGCGGCGATGGTTGGCTACACCATCACATCAAGTTCAAGCATGAGTGCGGTG

CGAAGAGTGAACCGCTTCCACCGCAACTGGTTGGCCGCCGGCGACGGCGACGGTGGCGGCGGCGGCGGCGGCGCCACCGG

CGTCAGGTACATGGTGGTGTTCGGCGCGTTCCAGCTGCTCCTCTCGCAGCTTCCCAGCCTCGAGAATGTAGCGTGGCTCT

CCGTCATCGCCGTCGCGACGTCGTTCGGGTACTCCTCCATCTGCCTCGGCCTCTGCGCCGCCAAGTGGGCGTCCCACCGC

GGCGGCGTCCGTGGCACGCTCGCCGGCGCCGCCGCCGGCTCGCCGGGGGAGAAGGTCTTCAATGTCCTCCTCGCCGTTGG

GAACATCGCTATCTCCTACATCTATTCTCCCGTGCTCTTCGAAATCCAGGACACGGTGAGAACGCCTCCGTCGGAGAGCA

AGACGATGAAGAGGGCGTCGCTGTACGGCCTCGCCATGAGCGCCGTCTTCTACCTCGTGCTCGGAGCATCCGGCTACGCC

GCCTTCGGCGACGACGCGCCCAGCAACATCCTCACCGGCGCCGCCTTCCACGAGCCCTTCTGGCTCGTCGACGTCGCGAA

CGCCTGCGTCGTCGTCCACTTCCTCGGAGCCTACCAGGTCATCGCGCAGCCGGTGTTCGCGAGGCTGGAGGCGTACGTCG

GCGGGCGGTGGCCGGAGTCCAGGCTCGTCACGGCCAGCTACGAGCTCCGCCTCCGCGTGCCGGCGTGGACGTCCGCGCCG

CCGACGGCCGTGACACTGTCGCCGGCGAGGATGGCGCTGCGGGCGGCGGTGATCGTGGCGACGACGGCGGTGGCGGCGAT

GATGCCGTTCTTCAACGCCGTGCTGGGCTTCATCGCCGCGCTCGGGTTCTGGCCGCTCGCCGTCTACCTCCCGGTGAGCA

TGCACATCGCGAGGGTCAAGATCCGGAGGGGGGAGGCGCGGTGGTGGGCGCTGCAGGGCGCGAGCGCGGCGCTGCTCGTC

GTCGCCGTCGGCATGGGCGTCGCGTCCGTGCGCGACATGGTGCAGAGGTTGAACGAAGCTGCTCCCTTCAAGACCACGGG

CTAA

>OsAAP18

ATGGGAGGAGGGACCAACGGCAACGGCGGCGGAGCGGCGTCAGCTATGGACGTCTACCTTCCCCGGACCCAAGGCGACGT

CGACGACGACGGCAAGGAGAGGAGGACAGGGACGGTGTGGACGGCGACGGCGCACATAATCACGGCGGTGATCGGGTCCG

GCGTGCTGTCGCTGGCGTGGGCGATGGCGCAGCTGGGGTGGGTGGCTGGCCCCATCACCCTCCTCCTCTTCGCCGCCATC

ACCTTCTACACCTGCGGCCTCCTCTCCGACTGCTACCGCGTCGGCGACCCGGCCACCGGCAAGCGCAACTACACCTACAC

CGACGCCGTCAAGTCCTACCTCGGTGGCTGGCACGTCTGGTTCTGCGGCTTCTGCCAGTACGTCAACATGTTCGGCACCG

GCATCGGCTACACCATCACCGCCTCCATCTCCGCCGCGGCTATCAACAAGTCCAACTGCTACCACTGGCGCGGCCATGGC

ACGGACTGCAGCCAGAACACGAGCGCCTACATCATCGGCTTCGGCGTCCTGCAGGCCCTCTTCTGCCAGCTCCCAAACTT

CCACCAGCTCTGGTGGCTGTCCATCATCGCCGCCGTCATGTCCTTCTCGTACGCCGCCATCGCCGTCGGCTTGTCGCTGG

CGCAGACCATCATGGACCCGCTGGGGAGGACGACGCTGACGGGCACGGTGGTCGGCGTCGACGTCGACGCCACGCAGAAG

GTGTGGCTCACGTTCCAGGCGCTGGGGAACGTCGCCTTCGCCTACTCCTACGCCATCATCCTCATCGAGATCCAGGACAC

GCTGCGGTCGCCGCCGCCGGAGAACGCGACGATGCGGCGCGCCACGGCGGCGGGGATCTCGACGACCACGGGGTTCTACC

TGCTGTGCGGCTGCCTGGGCTACTCGGCGTTCGGGAACGCGGCGCCGGGCAACATCCTCACCGGCTTCGGCTTCTACGAG

CCATACTGGCTGGTGGACGTGGCCAACGCCTGCATCGTGGTGCACCTCGTCGGCGGGTTCCAGGTGTTCTGCCAGCCGCT

GTTCGCCGCCGTGGAGGGCGGCGTGGCGCGGCGGTGCCCGGGGCTGCTCGGCGGCGGCGCGGGGCGGGCCAGCGGCGTGA

ACGTGTTCCGGCTTGTGTGGAGGACGGCGTTCGTGGCGGTGATCACGCTGCTGGCCATCCTGATGCCCTTCTTCAACAGC

ATCCTGGGAATCCTGGGGAGCATCGCGTTCTGGCCGCTCACCGTCTTCTTCCCCGTCGAGATGTACATCCGGCAGCGGCA

GCTGCCGCGGTTCAGCGCCAAGTGGGTGGCGCTGCAGAGCCTGAGCCTCGTCTGCTTCCTCGTCACCGTCGCCGCCTGCG

CCGCCTCCATCCAGGGCGTCCTCGACTCGCTCAAGACCTACGTGCCCTTCAAGACCAGGTCCTGA

>OsAAP19

ATGTCGCTCGCCGACGACCTGGCCGCCGTCGAGCGCGGCGGCCACATGGTGCCATCGAAGGCGGCGGGTGTCGACGGCGA

CGGGGAGCCGCGGCGGACGGGCACCATGTGGACGGCGAGCGCGCACATCATCACGGCGGTGATCGGGTCCGGCGTGCTGT

CGCTGGCGTGGGGCGTCGCGCAGCTGGGGTGGGTGGCCGGCCCCGCGGTGATGCTGCTGTTCGGCGCCGTCATCTACTGC

TGCTCCGTGCTCCTCGTCGAGTGCTACCGCACCGGCGACCCGTACACCGGCCAGCGCAACCGCACCTACATGGACGCCGT

CCGCGCCAACCTCGGCGGGACCAAGGTCGTGTTCTCGCAGATCCCCAACCTCCACAAGATGTGGTGGCTCTCCACGCTCG

CCTCCGCCATGTCGCTGTCCTACTCCGCCATCGGCATCGCCCTCGGCGTCGCGCAGATCGTAGTGCTTGACATGTTTGAG

ATCGAGTTTGCAGCGAACGGGGGAATCAGGGGCACCATCACGGGCGTCTTCGTCGGCGCCGGCGCCGGCGTCACCTCGAT

GCAGAAGGACACGGTGAAGCCGGTGGCGCCGCCGTCGACGGAGACGAAGGTGATGAGGAAAGCGGTGGCGGTGAGCGTGG

CGACGACGACGGCGGTGTACCTGATGTGCGGGTGCGTCGGGTACGCGGCGTTCGGGAACGACTCGCCGGACAACCTCCTC

ACCGGGTTCGGGTTCTTCGAGCCCTTCTGGCTGCTCGACCTCGCCAACGCCGGCGTCGTGGTGCACCTCGTAGGCACGTA

CCAGGTGGTGGCGCAGCCGGTGTTCGCGTTCCTCGACGGGCGCGCCGCGGCGGGCGCGTGGCCCGGGAGCGCGGCGCTGG

GCAAGAGGAGGCGGGTGCTCCGCGTGGGCTCCCTGGCCGAGATCGAGGTGAGCCCGTTCCGTCTGGCGTGGCGCACGGCG

TTCGTGTGCGTGACCACGGCGGCGTCCACGCTGCTCCCGTTCTTCGGCTCCATGGTGGGGCTCATCGGCGCGGCGTCGTT

CTGGCCGCTCACCGTCTACTTCCCCGTGGAGATGTACATCGCGCAGCGCCGGGTGCCGCGGGGGAGCGCGCAGTGGCTGT

CCCTCCAGGCGCTCAGCGCCGGGTGCCTCGTCGTGTCCGTCGCCGCCTCGGCGGGATCCATCGCCGGCGTCGTGGAGGCG

TTCAAGGCGCACAACCCGTTCTGCTGGACGTGCTGA

Gene sequences

>55902

ATGACAGCGGCTTCTTCAACCTCTGATGTAACTAGACTTGTAGATCAGCCCCTCTCATTTGAGCTTGAAAGGCAGAACGGGCATGCGTCAACATCAGGTA

ACTTTGAGCCCTTTTTATCCTGTGTTCTGTGCAATCTAGATACAAAGTGCTGTTCAGTCAAGACGAAGGTAGTCATTGAGCACCTCACAATCAGCAGCAG

TTGACCAGGTCTTGATCAGCTTGGGGTCTCTAGTACCACAAACAGCTTCCTTCCACAGGAGCCATATACATCTAAGCCCACTTCAAATGCCTTAGACAAA

CGCAAATTGCCCACTGCAGGCTCTACAGCACCACAGAGCAAATGGTACGATGCAACTTTTCACACGATCACCGCAGTAGTTGGTGTGGGGGTGCTATCGC

TTCCATATGCCTTCTCCTACCTCACGTGGACTGGCGGTGTAATCGCCCTGGCGGTGACAACAGCAACCAGCCTCTACACCGGGTACCTGCTAGCAGCACT

GCACGAGGACAAAAACGGCCACAGGCATAATAGATACAGGTGAGGCCAGCATGCCGCTTTACAAGTCAAACATTGACTGCGTAGAATGTATCATACAGCA

ATCTCACGTGAAAAGGGAGCCACTGAGAAGCATATCAAAGGTGCAAGGATGGGCTGCTTTGCAGGGATCTGGGGAGGGCTATCTTTGGGGAGAAGTGGGG

CAATTGGGCGATTGCCCCATTCCAGTGGTCTGTGCTGGTGGGCCTGGCCATCACATACACTGCTACTGCAGGCCAGAGCCTCCAGGTCTGCCTGCAGCCT

CACACAGAAATACTGCCTTGACTTGACCTCTAATAATACACATCTTACAGCTCTGACATCTTAAATGGCACTTCTTCTTAGTATGCATGACCAAAGCATC

CTGCAGGCATTCCCCCATTGCCGCGTCTTGATTGTCCATGCAATCATTTGCAATTAATAACACCTCAGATGCTGGTCTTGGCCAATCATTTGAAGACGAT

TGGGTTGCCCATTGACAGGCAGTGCACAGCAGCACGTGCAACAACGCTGTGTACAAGGCGGTTGGAGCGGGGCGGACTGATAGGAACTGTTCTAGTGCAT

TGGCCTGGTGGACGATTGTCTTCTCCTTCTTCGAGCTATTTCTGTCCCAGATAAAGGACTTCCACTCCCTCTGGTGGGCCCTGACTCCTCAGCTGCGCTT

GAATGCACACTCATGTTTCCTATCTGAGCATAACACCATGAGGTCCACACAGAAAGGGTGGATAACTACCACGCCTGCTGCCCTGTAGCAGAGCACAGCA

CAGCAGCCCTGCCAAGCCTTCCACCCGAATCCCTCTTCCAGTGTACCCAATATTCTCACACGCTGACGGAGGCACAACTGCCAGGATGCTGGAAGGAATC

AAATCCTCTTGAAGGGGCTTCCCACCCTCTTTAATAATCTTCCAGTCCTTCTGGACCTAACTGGGATTGCCTCTGTTCTGGCAGGTGGGTATCGCTGCTG

GGGGCGGCCATGTCAGCGATGTACTCAACGCTGGCATTTGCAACATCGGTGGCAGCTGGGTCGGAGGGCGCCAGCTACGGGCCACGCCAGGAGAGCCCGG

CCGCACTCATACTGGGAGCGTTCAATGCGCTGGGCACCATCATGTTTGCCTTTGGCGGCCATGCCATCCTGCTGGAGGTGCAGGTGAGGCTGCCTGCCCA

ACTCCCAAGTAATCCTTTATATTTTTTCTTAGCGAGCCCGAGGGATACACAAGGGGAGACTTCAGCACAGCAAGGGAGCCGTGAGGGCACTGCCAGATGC

CGGCTTCAGCCTCAAGCAGGGGCAACTGGATCTGACTAGCTGAGCTCATGGGTCATTCTTGATTTCACCTCCCAGCACCAGCGGCATCTTCAACCAGGCA

GCTTTAAGAAGAAGCTGTATGACGACCAGAAGACCTACTTGGGGCCAAGGGGCTGCTCACCACACGCGTCCAAGGCAACATGCAGTGCTGAAGATCAAGA

CAATGCTGTGCAGGCAACGATGCAGACGCCGCCGTCAGCGCTCAAGTCGATGATGCGCGGCCTGGGTGCGGCCTACACGGTGGTAGTGATCGCGTACTTC

CCTGTGGCGAGCGCGGGGTATGCGGCGTTTGGGAATGTGGTGTCCCCGGACGTGCTGCTGTCCGTGCGCAAGCCTGCCTGGCTCATCAGCATCGCCAATT

TCATGGTGGTCATCCACCTGGCAGCCTCCTACCAGGTGCCGCCTCCTCACCCGCCCCTCTGCATGTCCTGCCTGCCTGGGAAAGTCAAGATGGGGAGGAT

CAAATGTGATGATCCATCCAGGCTTCTATCATTACAACTTGCTGCCAGGAGCGATCTCCTTGCATGGACACTTGCTTGAGAGCTGAAATGGAGCTAGCGC

CTCGCATCCATCATGGCATTGCCCGCTGAAGCCTTCCTCCAGCAGGCAGGCAGGAAAATGCTGCAGGTGGACTGCCCTAGATGCATTCGCCAGCAAGCAG

CCACACCAAAATCTTCCTCATAGTAATCAGTTGAAGTGCTCCCTGGGAATGCATAAAGAGTGTGCGTGCGCGCAGGTGTTTGCGCAGCCAATATTCGAGA

CGGCGGAGGGCTGGCTGGCGGCGCGCAAGCACCGGCTGGTGGATCGGCCGATAGTGACGCGCGCGATCGTGCGCTGCAGCTACGTGGCACTCACCTGTTT

CGCGGCCATCCTCATCCCATTCTTCGGCGACCTCATGGGGTGCGCCGCCTACTTGCTCAGTACCCATGGCAGCAGTCAGGCACTTGCTCTTGCACCTCCG

TGGAAAGATCTTGTGCTGGAGGGGATGTAGCTGTGCAATCAGCCTCATGTGGCCAAATCACTATGCAGATCTGTATGACACGCTACAATTGCCAAGATGG

ACGCATCGCTGACCCTCCCTGCTGTTTCCTCTGTCGCAGGCTGGTGGGTTCGTTGGGACTGATGCCGCTCACTTTCATTCTGCCGCCCGCCCTTTGGATC

AAGGTGCGCCCACTGTTGCCCTTGCAGCAGTGCATGCACAAAAGAAGCAGCAGTCTGAGCCAAATCTTGGCTTAATCATTTGGCAAGGTGTTTAAAGAAA

GGTGTGGGCTGCGTGTGAACAGGCGACAAAGCCCAAGGGCCCGGAGCTGTGGTTCAATGTGGCGCTCATGGTGGTGTATGGGGTTGCGGGAGTGCTGGCA

GCCATCGGATCTGTGTACAACATCGTTGTGCACGCACATGAGTATCACACTGTGGGCTGA

>24967

CTCGTCATCTTGCTCACTGGATTAGGAGGCTGTAAAGGACGGCGGGAATCTGAGCTAGATACTGTGGGAGAGAGCCGATCGGAGAAGGAGTGGCTGGAGG

TGATTTGCTGTCTTAGCATATCCACTGGACTGGAATCTTCACAATTAGACTTCAGGTGCATATACAAGACGCTTATGACTGGTTCCGATGCATCACCCTG

ACCCCAAAAATGCTTTCGCGTTTGTTGTGCCGCTCCGATCGCATCCGTATAGGTAACCTAGGGTTTAAAGTCGGTTGCTTCTGTTAAGAAAGATCACTAG

CCAGAAAGAAAAACATTATGTAGCCGAACAAGTTGTAAGGCATCGGGGGGCTGTCCTGAGTCGAAGCTTGGCCAATTAAGGCTGAGACGGTGAAGATCAC

AGCAACGCAAGAGTTGTACTACAGATAGCGCAAGAGATATACAGTGACGCCTTGATCGAAATTGTGACATGTACAAACGCTAAATTTTGCTAGTATCTTG

ATCACAGACTGAAGCTTAGAACCAGTCATGGCGCTGGGCGAGAAGACCGGCATGGAAGACTCGGCACACGCCAACAAAGTCAACTTTTCGAAAGACCCGG

AGGGCCAGATGGAACTCGACGATAAGCAGACCGTGCCAGAATATGTTGGCAAGGGTGAGTATTCCAACGAGCCATCAGCTCGTCCTTGACGTTGGGAGTC

CCGCATGTCGATTCGTTGAAGCACGACCACGAGTTTGTACCACAAGGCTTCGTCATTGCACGAAATTCTTTGCAGAATGACCAGCAGGAGATTGCATGAT

AACTCGCGAGCCGAAAGCAGCCGTGACCAGGCCACTGCCACCCAGACCGTCAAACGATGCGAAACCCCACTGGCCTTGTGCACGCTGGGTGCCAGCCTGC

GTGGGGCCTTGCCAGCGCAGCTGATCATTCTCCGTTTGTGCATAGGCGAGTGGTACCACATCGGCTACCACATGACCGCGGCTGTGGCCTCTGTGCCCAC

CCTTGGCCTGCCCTTTGCTGTGTCTCTCCTCGGCTGGGGTGGAGGCCTGGTTGCGCTGATTGCTGGGGGCCTGGTGACCATGTTCACCTCCTTCCTGGTC

TCTAGCATGCTTGAGTATGGCGGCAAACGCCACATCCGCTTCCGCGACCTGTCTGTTGCTGTCTTTGGTGCGCAAGTTTTTCCCACTCTTATCTTCGATA

TCTTGTGTGGCTGACCACTCTGGTTTGATCCTGTATCGAGTGTCAAGTCCCTTCGAGGACTGATTCTGAGTTGTGCCAATGCCCGCGATACATTCCTGCT

CCATTTGCATGCAGAAAACCTGTCCTCCAGATATCTTGCTCCGCACCCTGCAGTGTGCATTATTGTCTGCTCCTGCGTTGCTGTTGAACACTGGAGTGGA

CCAGATCTAGGAAACTGCAAGTGTTGCAGGTGAGCTGGCCCTCTTAACATGCCTGCTGTGCACATGCAGGCAAGTCCGGCTGGTGGGCTGTGACCCCCTT

CCAATTCGCGGTTTGCATCGGGACCACCATTGCCAACCACATCGTGGGGGGGCAGGCCATCAAGGTGCGCCCTGTCAATCATTGCATCCGTCACTTTTGC

AAAAGCACTTGGAATCTCTTTGACGGGGAGACACATCTGAGAGATATCTCCATCCATGCAACTTCTGCAGCCGGCCCGATCCGGTCTAGGAGCAAAGGAT

CACACCTTTGGGCTCTTGTCCCTATTTATGGCATCTCCCGTATTTGAGAAGTTGGCAAACGCTGCTGCAAGGGCTCTTGTGATGCCATGCTGCCTGCTCA

TGCAGCAATGCTCTGTATCTCCTAGTGCAGACTTCCGGCCACCTGTGGCACCAGTGAGGCAATCATTGCCTTTGGGCTATGACTGTGCAAAACAATCTCA

GGGAAAGAGGAATGCAGACATCAAGGCAAGCGCCAGAAGACAGAAAAACCCAGTGCACGTCATGCAACAACAATAATGCCCCGCCCAATATTGTTGCAGA

CTTCAGATTTTTGAGGTTGTATTTGTGTGTGGCCAGGCCATAGACGTGCTGGCGCGGGGGGAGACACCGGTGACACTGACGCAGTACATTTTGGTGTTTG

GAGCGGTCAACCTGATCCTTGCCCAGTGCCCCAACTTCCACTCCATCCGCTTTGTCAACCAGACCGCCACTGTCTGCACAATCTCATTCTCAATCATCGC

AGTCGCACTCTCTCTGTACTCAGGTGAATCCCTACAAGTATTTAATTCGAATGAATGAACCCCAAACCCACCAGTGTTGAATCAATCTTTCTTAAGAGAT

TTCAGCAGAGCGCTTCGCGCACACATAAAACTGTTTCTTATTCTTGCACCATCTCATTCTCAATCAATGCCGTGGCTCTTTCTCTGTGCTGAGGTGAACC

CCTGCACCATCCCTTTTTTTCTCATTTTCCTTTTTTTCCCCTTTGTTCTTTCTGCAGAGGCTGATGTTTGCTTAGCGTCAGTTTGCACGACCAGATCAAA

ATTGCCCACGCTTGATGCAGATGCAAGTACGAATCCTGTCCTATGTGTGCCTGCAATTGGCTCCTGCAGTTTGGTGCATGCTTCTCCTGTGTGTTTCCCT

CTCACCTGCCTTCCTTGAAGACAGTTATCATGTGGAGGCTTTATGCTTCCACTGCCTATCGTATCAGCTGACCATATGTCAGGGCATGTCACATAGACAA

ACATCGTGGGAAGCTGAGTGTCAAAAAGCTGGTAAGGACTTCCAGGATTTTGAAATTTTTTCGTGGTGGTGCAGGTTTCACAATGGATCTGCAGCCAGAC

TACACAGTCCCAGGAGAAGGGGTGAACAAGCTCTTCAACATCTTCAATGGCCTGGGTATCATGTGAGGCCCACTCCCCGTGCATTTACATCTTAAAAAAG

CCGATGGATGTAAATCTATGTACAGAAAATGTCCTCCTATTGGTTTGTAAGGAGGCAGCTTGCACCCTGGACAGTGAGAGAATGAAAGCTGTGCAGGTTC

ATCATGTTTTCATGCATGCAGTTTCCTGTCTTCCAAGTCGCAGGATGTTGAGATTGCTTGTAGATTGGATGAATGTGCATGCAATGAGAGTGTGCTGACA

ATGTGCTGTGTGTTTTGTGCTGCTCAGGGCTTTTGCTTATGGAAACACCGTCATTCCAGGTGGGGAGTTATTGCTGAGAGACCTCTCCCATTTGACACCC

CTCGCTTCGTCACAATGATTGCAAGTATTTGTCCTAAGTAATGTCGTGATAATTCCTGCAAAGGAGTGCATGTTGGCAGCAACCTCTGTGACTCGAAAGA

GAAAGAGCTCTGTTCAAGTCAGTCTTGCTGATTCAGATGATTCATCATTTACTGATATTGATTGCAGAGATCGGTGCAACCGCCAAGGCGCCAGCCATGC

GGACAATGAAGGGTGGCATCATTATGGGCTACTGCACCATTGTGTCTGCCTACCTCTGTGTCTCCATTACTGGTGAGGGCCCCCTCCATACAATATCTGA

ACAGTCTCTTCTTTGCATGTCATGTGACAGGGTGCCCCCCACCTCTAAGAAATAGGATGCGGCACTTGGAGGTCTTCCGAGGCCTTGGGATTTGGTGGGA

TGGATTGTACACTGCTTGCTTCTCTTGCTTGCAGCATTGACATGTTGTGGGCATTCCAATAATTGAAACCGGGGTTGACTGCTAAGGAAGATTTCTGAAT

AATATGTCGGCTCATGGTCCTGCTGTGTGCAGGGTACTGGGCCTTTGGCAACGGCGTCAAGGGACTTGTTCTGGGCAGTCTCACAAACCCGGGCTGGGCT

GTCATCATGGCCTGGGCCTTTGCTGCCGTGCAGCTCTTCGGCACGACCCAGGTGACTTTTAATGCCCCTGTCAACAAATGATTTGTGGGGTATTAAATGC

TGGGGTGTAGATAAGCTGGCTGCTCACATCATACATGCTCTGTGGCTTGGTGGGCATCACTCATCAACTCATGGCATGGCAAGCAAGACCGCTGCTCGTG

ACAAGGAAATTTTTGAACTCTTGAGACTTCCATTTGTATCCTGTCAACAGATTGGGCTCCTGATGCGAATGGGTAGCTGTCTGGTAGGTAGTTTGCACAG

GATCTTACACTCCTGTTGACGTACAAGCAACGCTTTGGGCAGAAAGAATGGGGGAAAGAAAGGAAAGTTTATGACAAAGGTTTTGGACTGTGAGCAGGTG

TACTGCCAGCCTATCTACGAGGCCTGCGACAAGACCTTTGGCAATATCCTGGCGCCCACATGGAACCTTAAGAACACAATTGTCCGCCTTATCTGCCGCA

CTGTCTTCATCTGCCTCTGCATCCTGGTGAGTTCCCCAAATGAGCACAGCCTTGTTTGCGCATGGAAATCAAAACCAAGGAACTGTTTTCTGTCTTGTGC

CCAGCATTGCAGTGAAGCAGCGACTGCAACACACCTTTGGGTGTGACAGCAAGCTGCAGGAGAGTGGGACAATCAATGAGCAGAATTACCTGACTTCCAA

AGCCTTTCTCACTGGATTGCTGCTGGGTCGCCTTCAAGGGTGGATTATGATAAGCTTGACTGGTAGAGGGTTGAGGGTTTATGTACAGTCTGGGACAGAG

TGTGAGATTCTGGGGTGCCTGGCTGCAGGTTGGCGCAATGTTGCCCTTCTTTGTGGACTTCATGAGTCTGATCGGTGCAATTGGCTTCACTCCTATGGAC

TTTGTGCTACCCCAATTCCTGTGGATCAAGGCCTACAAGCCAAAGGGATTCTCGTGAGTGCTCTGCCTCTCTCGATACAAGCATTGAACACCGGTGATAG

TCCCATCTGAAATTTAACCTCATGGCCGCATGATTCTCTTGTGACAAGAGACTGCACTCCCAGATTTGCACCTTATAAGCATTTGTGTCTGTTTGTGTCC

CTGTTGCAGCACTACATACATTCCCGTTCGTTCTGATTCAAAATTCTAAAGACTGCACTGAATAGTAATTAGTGCCTTCTGAGGTGTGTTATTTCGGGGT

GGATGATGAATGTGCTGTGCAAATGCAGAAAGTGGTTCTCCCTCCTGGTCGCTATCATCTACATCATTGTGGGCATCATGGCATGCATCGGTGCTGTCAG

GAGCATTGTCCTGAACGCAGTCAACTACAGCCTCTTTGCCAACCTGTGAGCTAGTCTGTATAGTTTTGAAGGTGAGTCACCTTTGCATGACATTTGATGC

GCCCTTATGCTCTGCTGTGCATGCGTGGCTGTTTTGCATGCCCATTTTGACGAATTACATTGTCCTTCTCACTTTGTGCAAGGCTCCTTTGTCCTTTGCC

AGTGTCTCAGTGACTGAGAGACAGCCATTGCCGTACAATTATTGTAAGCTTGGCCATTGTTACAGCCATCAGCGTAATGCTCAGGCTTCGTTCCTCGTGT

CTACAGGAGTTGCCGCGCCTCATTTGGAGGGCTCTCATGGATCCTTTGAAAAAAGGAGCGTATTCTTTGGGCTCCAGTAATTGCACGAGCCAAGTTTTAT

TAGGACTTTCCGCCATGCGCACATTACATTGCCCATCAGCCCACATGGTGGCTGTATACTGTACCGATAAACTGAGCGCACTCAGTGCGAGAACGTCCAA

TGGCTCTCTCACCTGAACATCATCAGACAAGGGGAGCAGTCTAATTGTTTTGCAAGGTTTAGCATCTGCTGCATGGATTGGCAAGCCCTTGCCAACCAAT

GCAATCGCAGCTCAATTGGTATTATATCAACAGGGATTTGAATCCATGCCCCCTCGCTGTGCACAGCGGCGGCGATGGAATTCGTCGGCTGCCATTACGC

AGCCCAGAATGATCATTGCATTCATTCGTTAGGCCCTGACAACCTATGACAAATTGTGTCCCATTTTTTACTTTTTGCGGGTCTTAGCTCTTTCCCTGGC

AATTGATGTGCTCCATGGTTGTTGACAAGTTAGTACTGGGTTTTGGGATATGGGTCACCGATTGGGTGGCCCTGGAGCGGGGCTAAGGAATGTGCCATGC

TGGGACCATGGACTGCAGCAGTAATGTTTAGTGCTCAGTGAACACAGACGAGCTGTTTGCAAGATACAGTTTGTATAGTCTATAAAGTCTGACCAGGACA

CGGAGATGAGCACATTGCCGTTGAATAGAAAGTCGGGCCACTTTTGCATCCCACACTTCTGCCAGTGTCTGCCAGTGTTCCTCGTTACAGATGTCCTTCT

GCTACAATGGGTATTCATAATC

>31400

GCGGCGCATACAAAGTGTCGCTACATAACAAATATCTGTTTTTGGACGAGGAAACATGGCCGACGTCGAGGGTAAAGCTGCACCAGACACCGGCCATGAG

AATGGAAATGCCAAAGAACCGCTGGGCCATCTCAACAAGTACGATCAGGTGTGAAAAAACTGTTTTCGCTTGAAATGCAGCGTCAGAATGACGTTCACCT

CCTGCAGGGATACGACCGCTGGCATGGCAAAGCCAGCCGGCCATTTGTGTAGGTGCTTGGCTTCCTGAGCTTGGGTTTTAAGAAGGTACTCACTAGTGGT

GGTGCTGTGTCCGCAGGAATATAAACTTCCGATTACGGGAGACCGCACAGGGAAATGGTGCGATCCCAGTTTCTGCAAGATGTGTCCTATTGCTAAGAGG

CGAAGAGCTCTTCTCGAGACGTCAAATTCCAAACCCTAGCAAAGTCCACAGTCCAATTGGGCACCGTCCAATTTGGCTTAAACATTAGGGCGGACACATT

CAGACCATTTATATTGTCATCTCTGTTTTCAGGATGCATCATCATATTACTTTGTGGGGTTGTCTGTTGGCCTCTCTTTGTTGGATCAATGGGATCTGCA

GAAGGTGGGACAGTACAAGTCCTCTGAGAGTACAGTGTTACACTGATTTTCGGCTTGTGTTTTCAACAGGTGGTATTCTGCCTTCCACAATGTTACAGCG

ATGGTGCGCTCCCCCTTGAACAAGAATTTGCACTGTAACAAGTTTCATCTTAGCTTCAACGTGCAACCGGTCATGCATCAGGGCATGCCTGATGCACCTG

CATGCCCCTTGCCTGCGAGCTGTCTATTGATTGGCGTGAGCATCTTGCCAGGTCGGCGCTGGAGTGCTGGGCCTTCCTTCAGCTATGGCGTACCTGGGAT

GGGGCGGTGGCATGGTGTGGACCTCTCTCCTGCCTGTTACATCCCTTCTAGATGTGCAGGGGACTTGATATTGCTGCCCATGCATAATTGTGCCACAAAG

CCGCTCAAATGATTAGATGGAGAGGAATGCAGACATCAAGGCAGAGCGAGCGCCAGCAGATAGGAGAAAGGCCTGACACGCAAGTGCATATCATGAACAA

CTGTACAATGATCCCCGGGGTCAGGAACATTGAATGATGTAGATATCCTCCCCTGGAGTGATTTGTGTGCATTGATGCTGCGCAGTTCATCATGGTGTCC

TCCTGGATCATCACGCTGTACACGCTGTGGCAGCTGTGCTCCATGCACGAGATGAACGGCAAACGCTTCAACCGCTACCACGAATTAGGCCAGGTGAGTC

ACTGTCTTCTTCGTCTTGCTCTTATCATCAGACATGGTCAATTGTGCAGCTAAAGGCGCAAGCCTACCATCAAGCAACTATGTGAACATGCTGCGGCCTT

GCTCATGTGACTCTGTCACATCTGCAGCAAATTTCCTGACCCCCACACAGCCTTGTACCTGTGTAAAACGCTTTGTTAACAAAGCGTTCCCATGCGCCGA

GTTTACCCGAGGGGATTTCTGAGTTTGATTGGGTGTGTGGCTGGTGTGCAGTACGCTTTCGGGCAGAAGAGAGGCCTGTGGTTTGTCATCCCCTTCCAAC

TCATCGTCATGATTGGGCTGGCCATCGTGTACTGTGTCACCGGAGGTGTGTTCTCGGCAGCCTGTTACTTGACAGCAGAAGCCTGCTGCAGCAATTTGCG

CATCTTGAAAGATGTTGTGGTTTGATGTCCCACGTAGAAAAAATCACTCATGCCCACCCATCTGATCTGCGTTTGATCTTGCAAGCAAGGTGTACTGAGC

GCATCAGTGTTACTCAGGCGCATGAAACTTCAATGTGATTTTGCACAGGCAAGAGCATGCAGGCTGTGTGGCAGTTCTTGTGCAACAAGCCCTGCCCGGC

ATTTGGGCTGTCTGCATGGATCGTTGTGTTTGCCGGCGCACAGCTCTTCCTCTCTCAGGTCGGTCTGTGCTTTTCATCTCCCCATTATCCTTGCACCAGC

TGCTCTGATATCTTCTTGACGCATCATGGCGGTAGCTGTATAATGCAGTGTAACTACAGTAGACAGGAGAGTGCAGTGTGTTCGTGTGTGCAGTGCCCCA

ACTTCAACAGCCTGCGTGTGGTGTCCTTCGCGGCAGCAATCATGTCACTCGCATACAGCACGATCGCTGTGGGGGCATCCATTGCCTCTGGCCGTCAGCC

TGACGCTTACTACAACCTTGACACAAAGGACACTGCCGACAAGGTGTTCGGTGTCTTCAGTGCCCTGGGCACTGTTGCCTTTGCGTGAGTGTTCCGTCTC

CTGCTCAGTTGCATATCAAGCCGTTGTTACGTGTATGCTTGCCGAAGAGATGTTCGTTCCACACGTGGCTCGTCTTGTCACTGCCTGCAATTTCTGTTTT

CCTTGTCTGGCTTCCTCTGTCATTGTCAAGACTTGACAAGCAATCATTATGCATGCAGGTATGGAGGACACAATGTTGTGCTTGAGATCCAGGTGAGTGT

CAGGCCCTGCGACCCACTTACAATTCGTTTGAAACTCGTCCTTTGTAATCAGGCATGTGTCAGGAGTGTTTTGATGCTGCGACTGAGGGGTACACTGCTG

TGCAGGCAACGCTGCCCTCTCCGCCCGACACCTTCAAGCCCATGATGGCTGGTGTCTACGTCGCCTATGCACTTGTTGCTTGGTGAGTCCCAATCTTCCT

CCTGCTGTGCATACCTGAAAGCGCTGCAGCCCAATTCATGAACCCCAAATTACTTGTGCTGCTGCACCCTGCTGATCTTGGGAGGAGTATCTGTCACAGG

TGCTACTTTGCTGTTTCCATTACTGGGTACTGGGCGTTTGGTATCAACGTCGCAGACAACGTGCTCCTGACCAGTGCTCTGAAAGACACCGTTCCCAACG

GACTCATCATTGCTGCTGACCTCTTTGTGGTCATCCACGTCATCGGCAGTTTCCAAGTGTACTCCATGCCGGTGTGCACCCCTCCCTGTACCTGTTCCTC

TCCCAAGCGGCAAGTGATTTTAATGCTGGTGATGCTTTACCGGACCGGCGAAGAGGGACTGGGTGACAGGTCAGGTTCTGACATTGATGAGGGGGATTCT

GTATGTGTCCTGTGGGATTAGGTATTCCCCTGCCCTAGTGGGAGTGGACGTGGATTACAAAACAAGTGTGGGGTGACAGAGAACATGACACAAAGTCTTA

AATTCTGTCATTAACTGTGTTTGGGCGCTCTGTGCAGGTATTTGACATGATTGAGACCAGGATGGTGATGTCAGGCATCAGCAATGCCCTGCCCATGCGC

CTGTTGTACCGCAGCGTCTATGTCATCATCGTGGCATTCGTGGCCATCGTGCTTCCCTTCTTTGGGTGAGCCCCTGGAAACACATGAGACCCTTGCACTT

GTCTCATGCTTTCAGCTCTGACTGATGCTTATTTGCAAGTCCTGACCTCAAAACGTTTGTGCTTGCAGGGATCTCCTGGTGCGCATCTACAGCTGCTTTT

GGACAGAGCAGACCCAGAGCGGCACTGGGCTTATTCAATTGACCTGGCACACTGCTATGTCCTGTCTATCGCTTCCTTGCGTGCTAGTTCCTCAGAAATG

GTTGCCAACTGTTTCTGACTTTGTTGTTGTGCTGTGCAGGGCTTTATTGGAGCCTTCGCCTTTGTGAGTATCCTACCTTTATTCTGCACTCTCGAACAAT

CTTGATGTTGGGCCAGGCTGGCTTTTGTGAGCTCGCAATAGAGCGTACGGATATCTGACCTCTTGCAAAATGCGCGCAGGGCCCCACAACGTTCTGGATG

CCCCCCATCATATACCTGATTGTGAAGAAGCCCAAGATCAACAGCGGCCACTGGTGGGCCAGCTGGTTCTGCATCATCTACGGCCTCATCGTCACCATCT

TCGGTGCGAGCTTCTCCTGAGACCACACCTCCCATAAAATCCCCCTTCTGTAGGGGAAGTACTGAAGTTCATGAGACAGTGACGACACTGAGTAGATTGC

TGGAAATGTGGTTCCCACCCGGTGCATGTTGTGGGCAGCACAGGTGGGCTATTGAGTTCTGCAGGAAAGGGAGGATAGAGAAGTTGCTGTGATGTGTCGG

GAGCATGTAGCAGAATGTGCTGTGGTTGCACAACAAGTGAGACTGAGTAAGATGTGGCTGCAGGTTCCATCGGAGGCATGCGTGGCATCATCAAGAGCGC

CAGCACCTACAAGTTCTTCCAGTGAAGCTGCTGCTCACGCTCCCCAAGCTTGCTGAACTGCCTTGTTCATGATCAACCCAAAGTCAGGAGCGGAGACGAA

CTGCACTGCGGGTTGGAAATGGTGAAGGAACTTGTACAGTACTGTTTATGGTGAGTACATACAAATTTTCTCCATGACTTTCACAGCGTGGCACACTTGT

ACTAGAGCGAGGACCGAAGTATGGCCTGAAATTGAGCATGCTACATGCGGATCTCTCTTTGTCTGCTAATGAGCTTTTTGCTGACCTCAGCAGCTGCGCA

GCGGCTACTTGTTGGACAGGAATCTGTTGAAAAAGTGTACACTGGGACTGAGCTTTTTTGGACAGCTAGGACTGGAAGTGCCATAATCCTTGTAGGCCCT

TCTGGCTTGAAGTGGCCAATCTTCCGGTGCTTAAACCTTAGCCGGTGTATAACAAATTGCACACTGGTTGAGCAGCATTGCGTTTTGTTGTGATCTGGCT

GCAGCTGTCTTGCCGTTGCCTAAGTCTCATGAGCTCAGGATGGCATGATCCAGCTGCATATATAAGGGGTGCAAGGTGTACATGTTGGGAGAGGGAGTGG

CGAGAAATAAAAATTTCCTTGCAAGAATTGTTTCCTGATGGGGCTGGTCTTGTTACGGATAGCTTCTTGGGACCTCACGAGGAAAGGGTGTATTATCTAT

ACATGATTGTTATCAGTAATTTTTGACATAATCACAGGTAATATACATGTTTTTCTTGTTTTTTGTGGTCTGAGGCTTGTTGTAATGATGTCAGAGCAGA

AGC

>36205

ATGGAGAATGGCGAGCCTCACAATCCTAAACTTTTGGTAGATGAGAAGGGTTTCGCCCGCTCAGATCTCGAGAAGTACGATGACGACGGTCATGTTGCCA

GGACAGGTAAGGTCATTTGTGATCCGATGCACAGTGCAACTGAGCCGTAACTCCAGGGCCCTTGCCAGACCACAGACGCATCGATTGTATACGAATATCC

ATTTTGTCAGTACAATCGGTACAGCGCCCGACTCTTCCCAGGTATTTGATGACAGAAAGTATAGGTCCAAGATGGTGAGATCAGGAGGTGGCCTTGTGAC

ACAGGTGGCTGGATCACGGCCTACGCCCACATCGTGTGCGCTGTCATTGGCAGCGGCGTCCTGTCCCTCGCCTGGGGAGTCTCCTGGCTTGGTGCGACCC

TGTGCAGCTGCCCTGTAGCTACACCTATGCACACGCTTTAGCAGCTTGCATACTCTTCATCACCGCGTACAACCAGCAGCTAGCAGCAAGTTCCCTTCTT

TGCATTGTCTCTTTCCTTTGCAAATATCGCATGCAACCTCAAATGGCTGGATGTGTCACCTATTGACAGCATGCACCAACTGCACTTTCCTCGCAACAGA

AGACCTCCGCATGAGAGCATGTAACATGTTGGCGTGTGCCTTGAATCTCAAATATGAGGTCTTGCATGAAAGGTTTGTCGTGCAGGCTGGGTGGCGGGCC

CCATCGTGCTCTTCATGTTTGCATGGATCACCTGGTACTGCTCAGCGCTGCTCATCGACTGTTACCGCTTCCCGGACGTTGATGGGGAGAAGAGGAATTA

TACCTACATCCAGGCAGTCAAGCGCTACCTAGGTGAGCAACCCACCTTGTGCAATTGTGCAACAACGTATTACATCCAGTGTTGGAAATCTTTTCTTCTA

TGCAAGAGGAAAAGTACTGCTTGACGCAGCTTAATCAAATCACAGGGGCTCTGGAGCCTGCCCTTTGCACCTGCACCCTCCTCATTCCACTGCTGCTGTG

TGTTCCACAAGAGAGGAACAAGATGAGAGGAGGGGAAAATACCTGGTGTGACATGTGCTGCTGGCTGCTTTGCAGGTGGCAAATACTACATTGCTTGCGG

AGCGGTCCAATGTGAGCGCCACTCCCCTGTCCTGCTGCCTTTGTGGCTTGTGACTGCATTCAGAAACAGGCGTTCAACCTTTTCATTGTGACTGGAGGGG

TAAGGGTGTCGTGAAACGGTTTTAGTCGCTGACGAAACAGTGCATGTGCTGTGCAGATGCCAACATGGTGGGGACTTCCGTCGGCTACACAGTGACTGCT

GGGATTGCTGCCACGTGAGTGCAACCCTGCTGCGACTCATAATGTCATTTCAGATGCACTGCACAGACAGCTACACAAAGTCATGCTGGACTGTCTTGTG

CACGTGCTGTCCATTAGATGGCCGCGTCGTTGAGAATTTCGCATGTATGCACCTTGCCTGAGTAGTTTCATTGTTTCTTTGCAGGGCCATTCGTCGCTCC

GACTGCTTTCACGCTGACATCAGCAACCCCTGCGAAATCTCCAACAATCCCTGGATAATCCTGTTCGGCGCGCTCCAGATCCTCTTCTCTCAGGTGCGTA

CCCAGCTGCACCCTGTTTGGGTGCAGGACTAGAGAGGTCCGCACATAGGCACTTGGTTGAACAATTCCCAGTCTCTTCTGTCATCTGTGGGTAAGTTTGG

TGTTGTTTTAAGGTCCCCAGCCCTGCTTTTGCAGCTCAGTTCGAAGTTGGGCAGGCCATATCTCTTCAACCCCATTTTCTCAGGTACTTCTCTGGATTGG

GCGGACTAGAGATTCCCCCTGCTGAAGTCTTTAGTACCCCGGTCTTTACCAATTTAAGGGTGCCAAAATTTCACTTGAATCTGTGGCCTGGTTGCGATTA

CTTATCAGCATGCATGGCAGCTGTTTGTGAAAGGAAAGCCTGTCTCAGGCAAAGGGGTATGACAGGATCTGATGAGTTTAGTCTACTCAGAGTTTGCTGT

TATTATTATTACTACACATTTTCGAGTCCTTAAGGACTAGGTACGCCTGAGTCTGCTGTTTACAGGGCAGAATCTGCATGTATGTTTGTCCAGGGATGCT

CCAGGTTGTGGTTTGAATTCAGGTCGCAGCAAATGACAAGGTTGCTGCCTCCTTCTTGCAGATCCAGGACATTGACCGCATCTGGTGGCTGAGTATTGTC

GCCACTCTGATGAGCTTCACTTATGCCTTCATCGGCCTCGGGGAGTGCATTGCCCAAGCCGCACGTGAGTCCTTTGCCCCTCCCAAAAGGCCCTGACAAA

CCTGTCCATGCCCACTCCTCACAACAGGAGATTGAGCGCGCACTTGACATTGTGGTGGCAAATGACAGCTCTTCTCTTTAAGGCAGATCCAGTGCAAGAT

GTGCAGACCTAACATGCAAAGAGGCAGAACTGGGCAATCTTCAAGATCCACTTAGCAGTGCTGCTGAAGAGCGCTATATTAGGGAACGCTAAATATATGT

TATTAATATATGTTATAAATATATGTATTCTGAGGTCAGAGTTGCTGTGTTTTGGCAGAGGGGAGCACCACTGGCACGGGCACTGTTGGGGGCCTCCAGA

TTGGCATTGACACCACCGCCGCAGGGAAAGTCTGGGGCATCTTCCAGGTGCGGCCACAGCCTCCTCTCTAGCCTCCCATGCAGACAACTTACAACTATAT

CTTCCTTATTTGTCAAGACATGTGGCGCAAGAAGTACCCAAGTGAAGCTCTCAGCTGGCACTTCTGTGGCAGTGATGGCTATGCTCAGATGAACAGGGGA

TGTATTTTTGCGGGGAGAGGGAAGCTGTGGTCATGACAACTGCTGCTGTTGCATACATGCAATTTGTGAATCTTGCGAATAACTCATTTGCCTGTGCCAT

CTTGACAGCATAGTGTGTGATGCATGCAGGCGCTTGGTAACATTGCCTTCGCGTACAGCTTCAGCTTCATTCTGATTGAGATCACGGTGAGCACCAGATT

GTGAACAACTTTCTTCATGCAAACAGCACATTGCCAAGTACAGCTGTCTCAATTCAAGATGTGTCAACTTCTATGATGACCTCATGCACATGCTCACCTC

ATTTTGCACTGGCCTAACTGAGGACATTGAGACCTCTATCATGACATTGAATGCGTGCATTGGGGTCTGCGCACAGGACACAATCCAGTCACCAGGCGAG

ACCAAGAAGATGAGGAGGGCGACAGTGTATGGCATCGCCACGACAACTTTCTTCTACGCATGCATCGGCATCATCGGCTATGCCGCGTTTGGTAACAGCG

CGCCAGGAAACCTGCTCAGCGGGTGTGCTCTAGATTCCCGCACACATTCAATATGCGGTAGTTATTGCGCTTGAGCTCTGTTCTCTGTAAGCTTGCTCTA

AAACAGACGATGAGATATTTTCGCCCACAGTGTGTCCCTGGCTTGCTGACACCCATGCTCACTGCTTGCAGGTTTGGCTTTTACAATCCATGGTAAGAAC

TTTCCTAGCACCCCTTTCTTTCCTCAATTGCTGTACATTATGTTTTCAAACGAGACCTTAGTAGCTTACTTTTGCTCAAAACTTCCGCTTTCTCACAGGT

GGCTTATTGATATCGCCAACGCAGCCATCTTTGTGCATCTCCTCGGAGGCTACCAGGTATGCTGCATTGGTTAGACTGAACATCAGGGAATTACAGCAAG

TTTTTTTATGGCGCATGAGACCGCTCATCACTGACAGCCCTGACTGTACTGCAGGTGTGGATCCAGCCATTCTTCGGCTTTGTGGAAGCATCAGCTTTCA

GATATTTCCCAAAGAGCAGGTTCCTGCAGTGGGAGCTCTTTGCTGTTGAGGTGCGGTTATGACACCACCAGAACTTCTATTTTACTCCCTGCACACACTT

TTGGCTGCTGCTTACTGACATGCTACCCAGTAATGCTTTCAGGTGCCAGACATTGTGGTCCCAGCTGGTCTTGCAGCGTGTATGATCTGAACAAGCAATT

CTATGTAGAAGTGTCAAACTATGCTTACTGCAAGTGATGTCCGCACTACCGGTTGTGACAAGGGGGGGAATGCCTTTGCTGATGGGTGGATGGGTGTGTG

TAGATTCCCGGCATGGGCCTGTTCAGGGCGAGCCCCTTCCGCCTGATCTGGCGCACCGTCTACGTCATCATCGTCACAGTGAGTCCAGTTGTTTCCCATT

GAACTCAATAAAGTATTGAACTCAATAAACTCCTGCTTCTGGGACTCGCATCTCTCAGTAGGGATTCAACAGTTACCAGGTCCTTACAGTTTTTTGCCTG

TCGCAGATTGTGGCACTGCTGCTCCCCTTCTTCAATGACATTGTCGGCCTTCTTGGTGCGCCTTTCCCTCTGTTGAAATCTTGCATGTTTGCTCACTCAC

TAAAAAACCCAATAAAATAGGATCATCTGTATTCTTCATACTTGTCAGGCTCGAACAGAGCAGCAGTCTCTTGCATATCACTTCCTAAGCTCTGGAGTGA

CAAATTGGCTGGCTTTATTGCAGGTGCAATCGGCTTTGCTCCACTGACTGTCTTCTTCCCGGTGCGTCTCGCTGCCTCTTGCCGCAGCACTGCAACAGTG

TCAACCTGCTTCCCAGAAAACATTCACATGTCAAACTTTTGCAGCATGTTGCAGACAGCATTGGTTATACAGCAAATGGGCCAATCTCCACCATAATCCA

GAGTGCAAAAAGGGGACAGCAGCACCTGGGCTGACGGATAGCTGGTGTGTTGCGCGGTGCAGATCCAGATGCACATTGTGCAGAAGAAGATTCCCATGTG

GTCTGGGCGGTGGTGCTTCCTGCAGGGCCTCAATGTCCTCTGCTGGCTCATCTCCATCGCCGCCGCCATCGGCTCTGTGGAAGGAATCTATGCAGACACT

CGCAACTACACCCCCTTCCAGACCTCCTACCGCCGCTGA

>29311

GTGGGAGCCACCTGATGGGCCCACATGATCCTAACAAAAGCATCAATCGGCAAACTAGCAATTGACGGTAAAAACATAAGTGAGTGTATTTCAAAGCCGC

TGCATTGTCGCCTGATTCTCGTCCGAGGCCTGGTTGATTTTGACGCGAGCACAACATTTTGTTTTCCACATTGCGGTTCCAGATACAGAGCGGTCGACAC

CCCGCAACTCCGCGCGATCGTCTCGCAATCATCTGCAGTCCTGCCTTTGCTGCCCTTCTCGCAGCATTGACATTCTCGGATCGGGGACTTCCTGCTGCTG

ACCTAGCTGGAAAAAAGACCGACACGATGTCGAAGCTCAACGACAGTGCACACGGAAATGACTTGGAGAGGAAGCCTTCAAAAGTTGAGGAGGGAGTGAT

CGGCAAGCACGGATCAACGTCTCCCCTGATCGAGATCACGCACAATGGTTTGTTCCATGCCAACTCGTAATGTCGGTGCCGACGGCTGCAATTTGCTCGT

AAGAAGATGCTGATCACATGTCGGTGGATTATGGCGCTCAATGGCTTCAAGAAATGCTCACCGTGTTCACTAGCGAGTCCTGTTGTACAAGCTGTCGTAA

GTGGGGTTCTGGCAACGCTTAGTGTGCCAATCCCTTGCTACCTGCAAAGTCAGAGAGTTTCCGACTGCAAAGCGGTATTCTTCATCCATGTACATTCCGT

GATATCATTTGACGAGGCTTGAGGCTTGTAAATGCTCTTAAAAGTGCGATTCTTCTGCCGGTGTTCACAACTTCACTTGTGCCTTGCAGACAAGTGGTAC

CACGCTGGCGGCCACATTTGCACCATCATCGCAACACCAGCAGGTGAGCAAGAATCAGGATTGTTCTGTTTACCTGCGAGCTGTCATTTTTATGTCATGT

GGGCCATCACCCTTGTTGCTACATCATCTTGAGGCCCCACATGCAGAAGAGTTGCCATCAATAACTTCCATCTGACAGAACATCATTTCTCAGCTTCTGC

TGATTCTGTATATCACAGCTGAGTGTCATTGTGCTCTTTCACCTTTGCATCTCACCGCGGTGTGTTGCTTTGGAATGTGCTTGCTGATTTGAGGGGTATT

CCAGTGATGTCCTCGTCACTAATATTACCTCAGAGTCTTGGTGACACATCCTGCACCCTGTGTAGAAAATTCTATTGTCTTGCCCTGCAGCATACGCGCC

GCTACCCTTTGCCTTCGCGCACTTGGGCTGGGAGGCCGGAGTCATCTTTCTGCTGCTGGCCGGGTTAGTCACCTGGTACACATCCCTGCTGCTAGCTTCC

CTGGACCGCCACGACGGCAAGCGTCACACGCGCTACTGCGACCTGGCAGGCTCCATCTACGGTACTTCCCCAACTCTTGAAGCTTTTTGAATTTGAATCT

GAACCCATATCGCCTTTTGAATTTGATCTGATAGTCCAGACGTGGATGTTATACAGCCAGTTGATCAGCCCATTACCTCCGTCTCGGGGCAAACTAACAG

CATGTCTTAAATACAGTCACCGTTTCCAAGGGATCTTGCTCAGGGAATAGCTTATGGCTCTGAGCTGTACTTTGTGCATTGTACATTGAGGCTTCTTTTG

CAAGAGCTGTGATGGCTGACTGAGATGCAAATGTGCTGCAGGCAAGGGCGGCTACTGGTCGGTCATCTTCTTCCAGCAGCTGGCGTCCATTGGCAACAAC

CTGACCATCCAGATCGTGGCTGGCCAGTGCCTCAAGGTGAGAAGCTCTCCCCCTCTTGCTCTCTTGCCCCAATCCACATTTTTTATCTATGCCTTGGATG

CACTGCTATGACAATGTGCACCCCACACTGTTCATGATGGTGTGTGTTGTTCTTGCACACCCACACAAAATCTTTCTGCTCCTGAATCAGTCAGGAAATA

GACTTGGTATTCCTGCCAAACGGTCTGATCCCATTGCTCTCAGGAGGAATTGTTTGATCCACTCAACTTCATGTGTTCCTTCTGCCACGTGCGGGAAGCA

TGATTTGTAAATTGTGTGACAGCTGGCTGCGTGCTTAGGGGAAATTGTTTGAATCTCATGGGGGCACAACGCTTGATCCATAAGCGATGCTTGTCTCGCA

GGCACTGTACCGCCTCTACCACCCGGAGTGTGAGCCAACCGGCGCCTGCGGAATCTCCCTGCAGGCCTGGATTGCAGTCTTCGGCGCCTCCCAGCTCATT

CTGTCCCAGCTGCCAGACATCTCCTCCCTAAGGGAGATCAACCTCGTGTGCACCCTCTGCACCGTCTGCTTCGCTGTCGGCTGCCTGGCCATGTCAATCT

ACAACGGTGCGCCCTTTCTCTGCCTTGCCAACTTTGACCCTGCCAGTGGAACTGGTTGTCCAATAAGACATGTCTCGTTCCATGAGCCTGGATCTCCACA

AAACATAGATACGTCTCCTATCTCTAATGAGCCTAGCAATCGAGTCTTGAATAATTGGGCGCGATGGGTCCTGAGGTGGTATGCTTGGTCCATTGTGAAA

TACGCTTGGTTCCCCTTAGGGATTCAGATTCTGTCGTCAGCTGCATGTGAGCATCAGTGTCCCTTGCTTGCTTTTATCTGCCACAGCTGCATGTCAGCAG

GAACCCGCTGTTAACCCTGGGGAAGGCTATGCTTGTTGTGCTTGCTCCAGTGGCAATATGCTTTGCTAAGACAGAAGGCTGTCATGTACCATGATTCGAA

CAGCTGGAAGATGCTTGTAGGTGTTAATCAGCCTCCAGGAGATAACAGGAGAAGCAGTAGCGTGTGATGGGCGTTTGAGCTTTGAAGCTTTGCTGAACGT

TGAGAGTGTGTTTGCAGGAAACACGCAGGTGGACCGTTCCACAGTCAGCTACGATGTGCAGGGAGATGCGAAGCCCAAGATCTTCAACATCATGTTCTCC

CTGGGCATGTGAGCAACACCTTCTCTCTCATAGCTGCCTCGCTGCATTGCTGCTCCTCTGTTCACCTCCCTGTATTATTATTATTAATATTGTACGCTTT

TCCTGCCAAAAGGCAAGGTACGTCCTGCAGACAAGCTGCAAATAAAGGCTGCCATAATCCTACCCCTCGATGGGCTTGCCAGATCCAAGCAAGACCCAAT

CAACCGTGGGCACGGTGCTCAGTAACTATTCCGCCCCCCGCCAGCCGGGGCCTCCCTGTATATCAGCGTGAAATAGCTTGTTCAAACACAGAAATAGCAT

CTGCAGAATAACACGCCGTTCGTGCCACTGAAACAAAGCAGAGATGTAAGCAAGGATACAGATGGCCAACAGATGACCGGATTCATCATGGCTATGTTTG

AAGTGAACGTCCTGAGGAAAATTAGGCTCCTCTATTGTGTTCGTCTGCGCTAGGAAGTGCATCTTTGGTGTGCTGAAGAGTGAGGCGCTCAACAAGGCTG

ATGCTTGTTGTGTGCATTTCTGCAGCATTGCCTTTGCGTTCGGAGACACCATCCTGCCAGAGGTCCAGGCCACTGTTGGGGGTGACTCCAAGAAGGTGAT

GTACAAAGGAGTCAGCTGCGGCTACGCTATCCTGCTGTCATCCTACATGGTTGTCGCCATCGCCGGTGCGCTTCTGAAGTAATCTCCGTGGCACTGCTGA

ACTGAATATCTCGGGAATTAACACAGGAGTGCCTCATGTTGCGCCGTGTACAAGGGACCATCCGCACATATGTTTCAGGCGTATTTGCCATGCGCCTGAT

GAAGTGCAATACACAATCGAGTGATAGCCTGCCATGTGACTGTGCAGCCTAGGGGAGTCTCTTTAAATGACCTATTGCGAGTGCCATAAGTCAGGCACAC

ATGCAAGCAAAGGATACAGATGACAAAGAAACGAATCGGCACTGTGTCTGCACGCAGGCTACTGGGCGTTCGGCTTTGATGTGTCGCCGTTCGTGGTGTT

CTCCTTCAAGGAGCCCTCCGGCATGTTAGCAGCGCTGTACATCTTTGCCGTGCTGCAGATCATTGGCTGCTACCAGATCTATGCAAGGCCCACCTTTGGA

TTTGCCTACAACTACATGCTGAGGCCCTACGAGGGCGTCTGGTCATTCCACAACGTTCTGATGAGGGCCATTGTGACTACCATCTACATGGCCATCATCA

CCCTTATTGCTGCCATGATCCCCTTCTTCGGGTGAGTCACTCTTTTGGTATATCACCCTTGTCGCTGCCACGATTCCCTCCCTCTGTAGGTGGGTGTCTG

CTGTGCACTGTGATCATTACCCTTATCGCTGCCATGATCCCCTTTGGGTGAGTCACTCTTTTTTATTCACCCTTTTTCTGCCACAATCCCATTCGGGTTT

ACTGTACCCTGCAATTATCTCCCTTCTCTTGCCAGGAACCTCGGTTGCGGGGTGTGGTTGGGGGATAACTGTATCAAGCTGAGCTGTCTGTAATTGATTG

GTTTGAGCTCTTCTGGTGGCTTGAGCTTCTGTAGACTCAGAAGCATATGGCGGAGTTGCAGGGTTGTTACTGCCATGCTGTTCCTATGCTTGGAGCAGTG

AATGCTGTGCCTACAGCTGTTGAGATTGCAGTGACGTCTGTTTCAGGGAGCTTGGCAGGCGTCAATAAGATAAGTGCATGCGCAGGGACTTTGTCGCGTT

TGTGGGAGCTATCGGTTTCACCCCAATGGACTTCATTCTGCCGATCATCCTGTGGCAGAAAGTGGGCAAGCACTCGCTCATCGTCAGCATTGTCAACTGG

TGCATCGTGGTCTTCTACAGCATCATTGCCATTGCAGGTGACTATTTCTTTTGAATTCAAATTCCTCCAATCCTGACTGTGCACAGTTTCTGGGGGTCAG

CAAGTTGGGTTTGTGGGTTCTGGCTGTTTGCTGTCTGATGAGTGGTCCTGACAGCTCACACTTTCTACAAGTCAGCAAAGTTGGTTTTGTGGTTTCGGTG

CTTGCCATCTGATGAATGGTCCTGACATCGCCCAGTTATACGAGTCAGCCAGTCGGGGTTTTGGTGCTTGGTGTCTGATGAATGCATGGCTGTCCATTCT

GCTGTGCGGTGGCAGCGCGAGCCTCTCATGTGACCCCCGGAAGAAAAATGCATGAGAATAATAATAACAATGTATTACTGTTTTGCTCCGCTTACAGCTT

CCTCTCTGGCTGCCTGCAGGCGCAATCGGATCCATCCAGGCCATCAATGCCGACCTTGCAAACTTTAACGTCTTTGCAGACCTGTTCTGAGACCTTCCAC

CGCGCTGATCTGACCCAACAAGTGCGAGATACCCGGCTACCAAGCAACTCACCCTGCAATAGACCCGCCCGTGCTGCTGAGATGCATGTGCTGATGTCAA

ATTTCAGGCGTGCTCTTTTTCACAGAATGCAGTCAGCAATTTTGCGTCCCAGGATGGCTGAAAGAATGATCAGAGGCCACATTCTATGATTTGAATGCAG

CAGGGGGCTGTGGGTTTTCTGATGGCTGTGAAACTCTTGGTGTCAGCTGTGACACTCTGTGCAGATCTGTTGATGCAAGGGTGCTGAATTATTTTAGTGC

AGCTGCAACTGCCGCAAGACAGCTCCGCAGATTTATGAGGGTGCCCGTGCATTAAATGAGCACACATGAGGCTCTGAAGTTCGTGCTATTTGGAATTTCA

AGCGGCCCAGGGGCATTATTGGTGCGGGCTGAGAAAAGTCTGATTGCAGATTGATGAGTTGGTTTATTCCTGATATTTTTTTGGGAGGGTTTGCTCTTGA

ATGAAGGTTGCTCCGTGACGAGCTCCTATGTACAATGGTTGACTTGGGGTGTCACATTGGAGTTTTTACTACTCCTTGCGGGGAATAGCTTACTGGGATT

GCATGATATTAATGAGCTTGCAGGAGTGCTGCAGCTTTCCATTTTGTGTGGGACACTGTCGCACGCAATTCTCGTCTGAGAAAGTGGCCTTGTCATTGCT

GCATCCACAAATCGCTATTCCAAGTGTGCCTTGACAAGCTGTCCAAAGACATTGCTTGTTTGGCAACAGTTGGCAATTGCGACAATTTGCTGCAGAAGTA

TTTACCCTTTTAGGCGTGATAATCTGGTTTCACCACTTGCTGTGTCCAGTGTGACATGCCAACACATTTTTAACCACCATTATTTTTT

>Sphfalx0000s0509

GCCGCTTTGATTAAATTCTTTCACTGCTTTTCTTTCTCTGAAGCCAGTCTTCGACTTCGGAAGTTTCTTTGATGTCAGTAGGGAGTCGAGAATTCAAGTA

ATGCGACACCATCGTCCACAACTGTGCACTGAATTTCTTCCGAAAAATTTCTGCAGGTAGTCGTCTCCTTCCCCTCTTGATCGGTTGAGCCGCAGTCCGG

TCTGGATTTTCTTTGGAAGACGATTTCATACAAAGAAGTGCGGTTTCACGTCACGGAATCGATATAAATGATTTGTCCCTAACGCGCTTTCTTGCAAATA

GTCTCCCTGGCAGACTAGCTGCTTCCAAGTCTGGCATGAGGATGACGATCTCTGCTGCATGTGAAAAGGTTTGAATGCGCATGCATGCACTCGGATCACT

GTATAAGCATGCACAATTGAGGCTTCACGTAGCAAGCTTTCACGAAGTTGTGAGCAAGTCTTCTCTCTAAGCCCCCTTCTTTTTCCGCTCGTGATTTCAT

GTCCCAGAAACTGCTCAATTGTGATTCTCTCCTCGCGAAAGCTTAGTCACGTTGTTTTGTCTAAGGAGTGGGAAAAGAAGCTCCTCGTAAGGAAAGACGT

TTTGTTCGGTAGAACACTGTTGTTTCCTAGTTTTGTGAAATAAGGATCTGAATTTGTAGTTCCTTAATAGCAGGACTCGTGGATTTCATATTCTTAATAA

GATTTCCAATGCAAATGTTTGGTGAAGAATTTGGATCTCGACGATATCTCTGAAGGGACTCTGCTGCAAGATTGCAGCAATTTGCTTCAAAGAGGACTTT

GCTCGGAAATTAGTGAGGAGCTTGGAGCTCGTTGAAGAATTTGGAGAGCATGAAGTTTGAAGGAGCTGCAGGTGGCGATGAGAACCTGGGACGGGCAGAG

AATGGAAATGCAGCAGCTGCCGACCAGTTCAACAAGAGAAACCACGCTCTCAATCATATTCCCACCTCCAAACAGCTGGACGACGATGGCAAAACCAGAC

GCAGAGGTACAATACTCCCTGTTACTACTGTCCTGGACTGCTGAACAATAGGTGATGTTAGTTATACAAGAAGAGGCTGGCCAAAGATGAAGCTCCCCAT

TGGCTCTCTCTCTGTGATGAGGGCGTTTGGTGATGGCGCTGCTGTGCAGGGACGGTGTGGACAGCATCAGCACATGTGATAACAGCAGTCATAGGCTCAG

GGGTGTTGTCTCTGGCCTGGAGTGTGGCACAGATGGGCTGGGTCGTGGGTCCCACTGTGCTCCTCCTCTTTGCCCTGGTCACATACTACACTGCCCTGCT

GCTCACAGACTGCTATAGGTACCCTGACCCTGTCTCGGGCAAGAGGAACTATACTTACATGGATGCTGTGCAAGCAAACCTGGGTAAGTTTGCACCAAGC

AACTCAGGTATGTTTTTGTGTATATATATGAAATCTGATGCTAGCCAAATTGGCCTTGAGTAAGAGCCTCTTCCTCAGAAATTAACATGTAGCAAAGTTT

GGTTAAAACCCATTAATTGCTAAGAACTGGTCAAGTGACAGAAAGCAGCTGATGCACCAGTTTTACTATGATCCCATTGTTCTTGAAACCAGATGTTGGA

TGTCCAGCAATTCTGTTTTTTTGGAACTTTTGCTTCATAAGGTTTGTGAATAATTGTGTCTGGTGCAGGGCCAAGGCAAGTGTTCATCTGTGGGCTAGTA

CAGTACTCCAATTTGTTGGGGACTGCCATTGGGTACACCATCACTGCGACAATAAGCATGGTGTAAAGTCCCTTCTCTCTTTGCCTTTGAGTTTGAACTG

GTGATCAGCTCTTCAAGTTGTGGTTTGCAAGTTCCTTTGCAGTCTAGTTGTCAGCAGAGTTATATCTTTCACAAATATTGTAATCAGTGATGTCCATTAA

GATTGGCAGGATTTCCAAGAGCAAACTTGTGAGTATTTTATATCTTTTCTTAGTGGAGACCTTTCTGGCTCATTATTTCTTGCAGCATGTGGTGACTTGT

GAGCAAGAATGTGGAAGTCTGTGCTTCAAATCTTTAAACTGAGCAAAGTACTGCTGAAACTGCCATTGGTATATTTGATTGTTATCATGTATTACAGTGC

CTAATGTCCTGAGATGATTTACTGATGTTGTTTCTCACACTCTGGAGCAATTTGATCAGCAGTTGGGATAAGAACCACTGTAGCCCTGGCTATTTCTTTT

CTGAAGATTGACTATGCAACGCTCGTCTCTTATGTACTCCTCTGTCCTGAAAAAACTGACAGCATTTTCTGTTTGCACAGAGCTATAAAGAGGTCAGACT

GCTTTCATGCAGATGGAGATAGTGCACCATGTCGGGAATCCAATTTCATATACATGGTGTTCTTCGGGATAGTGCAGGTCATATTGTCACAGATCCCTAA

CTTTGATCGACTCTGGTGGCTCTCCATAGTAGCTGCAATAATGTCTTTCTCGTATTCCACAATTGGCCTTGGCCTCGGCTTAGGAAAAGCTTCAGGTAAT

CTGAGAAACACCCATCTATGGAATCACATATATTCAGATGGACCCACTTGTGTCCTTTATTTGATGAACTTATCAGTTCTCTTTGGATGTCTTTTTTGAG

ATGCATGTGTCAGCCTATGTCGTTCCCTGCACAAACAGAACAAGAGAAATTCATGAGATTAACTGTTTAGTAGACATGTTGAATATATAATTGTGGGTTT

CCTGGACATTGCAGAAGGAGATCATTCTCATGGCACTTTGACTGGGGTTGAAGTTGGTGATCATAGTCTTGGCTTTGCAACACGAGCTCAAAAGATCTGG

GATGTTTGCAATGCGCTTGGCAACATTGCCTTTGCATATTCCTTCTCCATGATCCTTATTGAAATCCAGGTATGCAGGACTCTCCCAAAACTACTGTACT

GTATTTGGCTTACAGACCACAAAGATGTGCATGTCATCAAATGGAGTTTCATGACTTTGAAGTATTCCTGAAAATGTAACAGGTCACTCTTATGTGTTGT

GCAGGACACACTGAAAGCTCCACCTGCTGAGAATAAAACAATGAAGAGGGCAACTCTTATTGGGATACTCACCACCACAATATTCTACATGAGTGTGGGC

TGTGTGGGTTATGCTGCCTTTGGGGATGCTGCCCCTGGCAATCTCCTCACTGGCTTTGGGTTCTACAATCCCTATTGGCTTGTTGACTTTGCCAATGCTT

GCATTGTTGTCCATCTTGTCGGTGCTTATCAGGTATTTATTGGTGCATTCCATCATTTCTCATCAACTTTGGTGTCTTTATTGCTGTAGTATTCTGTCCC

AGAACCTGGAAATAAAATTGTATGCAAATTATGCTAAGCCAATTTAGCAAAATGTTGCTATATTGCACAGGTTTATACCCAGCCACTTTTTGCATTTGTG

GAGGATTCAATGTCCCGAAAATTCCCCAAGAGCAACTTCATTCACAAGGAGCTTGAAATGAATCTCCCATTGGGGGGACCATTACATATCAACCTATTCC

GCCTTGTCTGGCGCACTTCTTTTGTCACATTCACAACACTGGTGTCCCTTATGGTTCCCTTCTTCAATGACGTACTGGGATTAATTGGTGCATGCGCATT

TTGGCCACTTACAGTATACTTCCCAGTTCAAATGTACATTATGCAACAGAGCATACAGAGATGGAGCTCAACATGGCTTGCACTCCAAACCTTAAACACG

GTTTGCTTCTTTGTATCCCTAGCTGCAGCTGTTGGATCCATTGCTGGAATTCTCACAGACGTGAAACATTACACCCCATTCAAATCCTAGGATCAGAAGT

ATACCCGTGGGCATTCTTCAGGGGTTTTAGTTACTTATGGAAAGAGGGTCAGACATTGTCAT

>Sphfalx0002s0399

GCGCAGGCAAGCACGCACTCTCGCTCGCTCACGAACTTGCTCACAAAATTGAAGATCATGGGTGTTTAGATCTGTTTCTTATTTGTTTTTTTGGTTTTTC

TTGAGTGCTCGATTTCTGGAGCCTCTGCGGGAGATGGCATTTGCGTGACAGCCAGGCTCTAGTTTTGCTTGCACAAGAGCTCTCGCCCGCTCCAACAACA

GCTTCTTGTTTTGCCTCTCTTTTTTTTCTCTCTTCGTTTCCAAAACTCAGTTCAGAAACAACTTCATCATCATCATCTTCTTCTTCTTCTTCTTCTTATT

CGTCATTACCGGCCAGCGATTCAAAAGTTTTGAGTTCTGGATCTCCTCAGCAAGGAAGCCTTCTATCTGATTCGTAATAATCATGTTTTCCAAAGACTCC

AAAGGCCCCTTGGCATCTGCCACTACCGATGAAGATTTCCAGGTGAACAAGAAAACCCCCTTCTTCTTTCCTAGGAAACGCCAAAAGCAAAAGAAAAAAT

CCTCTCTCTCTCTCTCTCTTCCTTCCTTCATTCCTTCTTTTCCCCCTCGTATGATCAGTGGTTTGAAGTAGGGAGTGAAATAGAGCGAATGGGTTATTGT

TGTTGCTCTACAATCACACATCAGGAGTCAGTCATAAGCTCAAGATATTGGGAGGGAATTTTAGCAAAGCAAAAGAGGGGGTTTTTTTTAATAATGCTCT

TCACCATTTCTTCTGACACAAAAATTGTTGTTCTAATCACAGGCATATGTGGAGGAAATTGAGCCTGCAAAACCAGTCAATGTTGAGGATTGGCTGCCAG

TGACTGGGAGCAGAAATGCCAAATGGTGGTATGCAGCATTCCATAATGTGACAGCTATGGTGGGTGCGGGAGTTCTTGGCCTGCCTTATGCTATGGTTTA

CCTTACCTGGTAACGTTCAAGCCTTGTTGCATCAGCCCCCAACTTTTATTCTTGTTCAGTTTGTAAAAACATTACTTCGTGATGCAAATGCATGACAAAA

TTGCAAAGTATATGACAAAAGATATTGCAGGAGCAGATGAAGTTAGTATAGCTTGTGAAAATGTTTGTCTTTCTTGGGTTTTATCGAGCTCTATGAAAGA

GTTTGGAGAATCTTGTGTGGTGAATTCATTGTTGGATGCCCCCCATTTTCAATAGAGAATTTCAAGCAATTGATTGTTGGATCCCCCCCACCCCCAATTT

CAATACAGAATTTAAGCAATTTGAGTGGGGGATAATACAATTTGCCAGGGGTCCTGGAGTGATAGTGTTGGTGTTATCGTGGGTCATCACACTCTACACC

TTGTGGCAAATGGTGGAGATGCATGAGATGGTGGAAGGCAAGCGCTTTGATCGCTACCATGAATTGGGTCAGGAAGCGTTTGGGGAGAAATTAGGACTCT

GGATTGTGGTACCTCAGCAGGTCATCGTGGAGGTAGGCGTGGACATTGTGTATATGGTAACTGGTGGCAAATCCTTGGAGAAGTTCTATGAGCTTACTTG

CAAGAAGAATTGCTACTTGCAAAATCGACTTTCTATCTGGATTCTTGTGTTTGGGTCTGTGCACTTGTTTTTGGCTCAACTACCGAACTTCAACTCCATT

GCGGGAATTTCATTAGCTGCAGCTATCATGTCACTCAGGTGGGTTAATAATATTCTGTGATTTGTCTCATGTTTATTGGAGGCTGCCAGGGTGCTTAGGA

GGGTTATTGTTTTTGAGCTCCAATTTGGTACTCATAGAGGGCCCATGTAGTATATTGCAGCTACTCAACAATTGCATGGGCCATCCCTGCCCATTATGGA

CACTCCTTGCTTCCTCCCGGGCAGAGGCCAGACTATCATTTGCCACCAAATCAGTCAACAGCAGCAATGGTCTTTGGTGCTTTCAATGCATTGGGAACAG

TAGCATTTGCATATGCTGGCCACAACGTCGTGCTGGAGATACAAGCCACAATCCCATCAACTTCTCATCGGCCATCAAGAATTGCCATGTGGAGAGGAGT

CCTGGTTGCTTATGGAATTGTGGCTGCATGCTACTTTCCTGTTGCTTTTGTTGGTTATTGGGCGTTCGGTAACCAGGTACAAGACAACATCATCACGTTT

GTGGCCAAACCTGTGTGGCTGGTTGCCATTGCCAATCTCATGGTTGTCATTCATGTCATCGGAAGTTATCAGGTACTTCTTTCCATTCATCCTGCAGCTA

GCAGCTGAATTAGTCGCGTTGAAGATGACTTTGAATAAACCTTTGAAGAAAAAACCCTTGTGAGAATGGAAGGAAAATGCAATAAATTCAAAGTTTTAGG

AAGTTACAAATGGGTGGTGGTTGCTTTGAGTGCAGATCTATGCAATGCCAGTGTTTGATATGCTGGAAACACTGCTTGTGAAGAGATTTCGTTTTTCTCC

TTCTCTCCGACTTCGGCTCATTACTCGCTCCATCTATGTTGGTAAAAGCGTTTTGCACAGATCCCAGAGATTCTTTTTAAAGGCCTTGGCCGCTCTGCTG

GTGAGATGGAATGAGGGCATCATATTTGTGCATGACAGGAGAAAACTTGGAATTCTGTACTCTCATGGTCTCATGACTGCACATTTGTTTGTGCAGGTTT

GACAATGTTTGTTGGCATAACCCTTCCGTTTTTTGGTGCCTTGCTGGGATTCTTTGGAGGGTTTGCCTTTGCCCCAACAACATACTTTGTGAGTCAGACT

TTCCAATGAACTTGCGTTGCTGAAAGTGGCCATGAAGCATGAGTTTCAGTCTGTATCATCAGTCCATGTGTGAAGAACTTTCCCTGGAGAAACTCTCAGA

TTAACCTCATTTTGTATCCATGTGGCAGTTGCCTTGCTGTATGTGGCTGACTATCAAAAAGCCAAGAATTGGAAGCCTATCGTGGCTTGCTAATTGGGTT

AGTCAAATCTCTTTTGCCCTGATCCTCTTTGAAGTTGTACTGTTTACAAGAATGAAGCATTTTTCTTTTCAGTGTGTTTTGGCAGTTGCATCACCAACTC

TTGAGAAATGTATCTGCTGAATATCACTTGTGCATGTGCTGCTGAATGTCTGTGTTTTTGGTGACTTTTGGCACTTGAATCACCAACTCTTGAGAAATGC

ATCTGCTGAATGTCACTTACACGTCTGCTGCTAAATGTCACTTGTGCATCTGCTGCTGAATGTCACTTACACATCTGCTGCTGCATGTCACGTACACATC

TGCTGAATCATCACTTTGTCATGTAATGATGGCTGTTGCACTTGGGCAAGATGATGTCACTGACATTGTATATCACCATGATGTATGCAGGTGTGTATCA

TACTAGGTGTCATGCTAATGCTTGTATCATCCATTGGTGGACTTCGACAAATTATCGTTGATGCCTCCAGCTACAAGTTCTATCAATGAGAGGGGTAAAA

AAGCAAAAAAAGTAGAAATATTATTATGTCAGATTCTTTACCATTTTGTCAGGGCCAGAAAGATTTGCTCTTTTTTAGAAAGAAGTTGAATAATGAATGT

TCAAGAGCTGCAACCTGTTTCCCAATTCATTCCTTTGCAGTTGTGGAGTGAAATGGCAATAAGAGACGAGTGCGGTAGTCCCAGAGCTTAAGGTGCAACC

TATTTATGCATTTTTTAAAGCTAATGGGTATACCCACAGGTTGAGGGCATTTTTTGGCACACCTGTAGGTTTAGGATTTCAGCATTTAAGCTTTAACGTA

TTTATGTGTAGAGTTGCTTAATCACAATGGAACTGAGTTGCATAATCACTAGCAACTCCAGGAATGTTTTCAATGGCACATATTATGGCAAAAGCACCTT

>Sphfalx0003s0314

AAGAAGAAGATTTGGGAGTTTTGAAGACAAGGATTTTGTCCATGACTGGTGCAAAACAGATCAGTTTCATGAAAGATGTGGTTTTTGTTTGATGTTTGAA

GTTCAAGTTTTCGACTTGTAAGACTACTACAACAACAACATAATAATAATAATACTGATGAGATGGTTGAAAAAGAAGAAGAAGAGGAAGATTGCCAAGT

GTTGTTGCGAGATTCATGATATAAGTAATGAATCGACGTTTGTAGAAGATTAGAAATTATTTCGACTAAGTTTTGCAAGTTAGCTGCCAGGAAAACAGGT

GCACGTTTGAAGGAAGTTGAGAAGCGGATTTCGACTTTGCAGCAGCAGTAGCAGCGGCGCCTCCATACCAGAACTGAAGAAGAATCTTATAAAAGTCTGA

AATTCTGTTTCCAAAATCTTCTCTGATCAAAGTGTGGGACTGCAGCAGCTGCTTCTACCTGGCCGTCATCATCTCTGGAAATATCTAGACACCTGTGGAG

GTGATGGAGTTTAATGCTCTTGAAGTAGAGTTCTTACTAGTGGAAGATTAGAACCTGCAACCCGGCTGGGCGGGGATGATGATGATGATGATACTCGACG

GAGTTGGAAGTCCGGATTTTCTTGGATTCTTCTTAGGATTCAAGATGTGGTGCTAGCAATATTTCAGATGCTGCTAGGAGTACTGGTATCAAGTTTGAGA

GAAAGGATTTGGGATTTGAGTGCATCATGATGGAGGAAACTGGGAACGGCATGCAGATGGCGGAATTGACAACCAAGTACACACCATTAGATGGCTCAGG

AGGTTCTTCATCACACTCACAAGAGGACGAGGAGCAGCAGCAGCAGCGGCGGCGGCCATATTCAAGCTTGAGCGATGCTGCAGATGCTCCCAAAGACGCC

GGGACTCTGTTTGTTCTTGAATCCAAAGGTACTGCACAACTATGTTGCATACTTTTCCCTTTGTGATCTTCCAACTTCGTCGTTGACGGTCGCCATCATG

TACTCTTGAAGCTGAAATGCACCTACAGAAGAACACATGAGATGGACACTCTTGAGACACAAGATTGCGCTCGAAAATCCAAGTAGAACACAATGGCGAT

CAGCGCGCGACAATAATATCGTAACTTGCTGCAAAGAAACGATCACGAGCTTTGTCGAATGCATAGTACTGAAACCGAGCTTCAAAAAACCCCGTCGCTC

ACAAACTCTTGTTGTTTGTTTGTTGCAGGGACATGGATGCATGCAGGGTATCATTTGACAACGGCCATCGCCGGTCCATCGCTGCTCTCGTTGCCGTATG

CATTCTCCTTTCTGGGATGGGCACCCGGATTGCTGGCGCTGACAATCTGTGGCCTCGTTTCAAGCTATGCCTACTGCTTGCTGTCGCAAGTCTTGGACGA

TTGTGCTTCCAAAGGCCATCGCTTCTACCGATTCCGAGAACTCTCCCAGTTCGTCATCGGTACCATATTCTATCATCATCATCTTGTCAATCCCTTCCTT

TGCTGCATCAAAGCTTCAATTCCTTCCGTCCTCCATCACAGTTTCAATTCCTTCCTTGCTGCATCAAAGCTGCAATTCCTTCCTCCATCACAGCTTCAAT

TCCTTCTTTACTCCGTCAGCTTCAATTCCTCCTTTCCTGCATCAGTGCTTCATTTCCTTCCTTCCTTCATCCATCATTTGTTTTCTTTCTTGTTTCTGCA

AATGCTTGAGGTTTTTAGGGTTTAAGGGTAGAGTTTTGATGCTTGCAAGAAACTCCAGATGCTTGATTGATCTGATTGCAAGGTCTTTCTGGATGTTTGA

TTCATGTGTTAGCAAGAGAATTCCAGATGTTTGATTGATCTAATGCAAGAACTTCCAGATGTTCAATTGGTCCAATTGCAGGATATTCGTGATTGATCAG

TGTTTTTGGATTTGCATTTGGTGTCTACCGTGGTCAAGATTCTGCAACTAGATGTTTTGTATCTGGCATTTTGAAACATGTTTTGTTCCAGTCATTCTTT

CTTATATTTTGAGATCAAAGAGATTTCTGAGAGCTTGAGTTATTTCCATTTCTTCTTTACAATCATATGATTCATCAAGAAAATGAGACTGTAAACTTCA

CCTTGAAATCAGCAGCAAGTTTTTGTTGATTCAGCTTCAGAATCCTTGTTACCATTCCATGACAAGGAAATTTTTTGTTTGATATTGCTTGAAAGTCTTT

GTGTCTGGTGAAAATTTTGAAAGAAGAAAACTGAGCTCTAACCTAATTAGGCCTCAAGACTAGAGCTGCACACACATGTACACTTAAAGAACTGAATCGG

AATATTTGATTTTGCAGGAAAATCTTGGACAAACTGCTTTGTGACACCGGTGCAATTTGGAGTGTGTTTTGTGACTGTTGTTGGTGCTATTTTAGCTGGT

GGCTTTGCGGTGAAGGTATGCATGTGCAAATTTATTGAGGTTTTTCTGTTACAAGAGCACTGCAACCAGAATGCATCCATGTATCTACTCTCTCTCACAT

ATGTTTGTTTGTTTCCTGGCACAAAAGAACTGGGGGGTGTAGTATATATTTAGGACTGTGTCACTAATCACCCACCCCTCACAGGCCCTTTTCTGGAAAA

CTGAGTGATGATCAGATGTTGGAACATAATCTTGACAATTCTCTCTGATATACAGAACAAACCTCTTGATCTTTGCAGCTTATATACCTGGGAGTGAATG

CGAATGGAACGATCCCATTGGCAGCATTTGTGGCAATGTTTGGGGCAGTGATGATAGTTCTTGCACAGCTACCATCATTTCATTCCTTGCGGTATATAAA

TCTGGTGTCTCTATTACTTTGCTTGACCTACAGCCTTTGTGCTACTGCTGGCTCTGTTTTGGCAGGTATCATAGTTTTCTTAACCTTGGGAAGTTGATCG

TCTATGTGGACATTTCAATTTTTCTCATGCAGACACATACAAGAGTACATTATTCTATTTTGCTTTTTATCTGCATTGCTGTGATCATTCCTTTAAGGTC

TCTGTGGTGCTTGCTTGATTTTTGATTCTTGGTGCAATTGGATTGCACACTGAAATGTGGTTTAATCTTTGATTTGTAGGCTACAATAAGAATGTACCAC

CTAAAGATTACTCTGTGGTTGGAAATCCTGCGGAGAAAATGTTTGGGGTTTTCACTGCACTTTCTGTCATGGCGGGAGTTTATGGAGTGGCTATTATCCC

TGAAATTCAGGTCATATTTCCATTCCACCGTCAGTAATCTGCTGATCTCGGTTCCAGATTTTTGTTTGGGATTATCAAGTTGGTCATATATATGTATACT

CAACTTTTTTGAGCTTACAACATCTTGCACACTTGTTTGCAAAAATGTGGGACTTTGATATGCTTACCCAATATCACTTATCATATTTTGACCATTTCAT

GTTTCTAGATAAACTTAAGAGCTATGCTATGCAACTGAATTGAGCTCTTGTAGTGCTTGATTTCATGTTCATATTTTTCTCTGCTACTTATGGCTAGACT

CTTCTTCTGCTGTAAATAATCAGAGAAATGTAAGACCAGGTACTTTAATGAAGCAATTGCTGATGCCCATGTCCTAAAACCATTAATGATAATGAGAATG

TACTAGCTGATATTGAGAACCAGTGCTCAGAGTGTGGCAGGCAACAATGGCTCCTCCAATTGTTGGGAAAATGGTGAAGGGCATTGCTTTGTGCTACGTT

GTGGTGGCAGCCACATTTTATTCGGTGTCCATTGCTGGGTACTGGGCTTTTGGGAATGGTGCACAGGGGAATATATTTGATAACTTGGTACCTAGTGGTG

GACCTCAATTGAATCCAGTTTGGCTCACAGCTATTTCAAGCTTTGCAATCGTTGCTCAACTACTTGCTATTGGCCTTGTAAGAAGATACACCCAATTTTG

CTTTGCTAATTTTCTTGTTCCTTACAAATTAGAACAGTCAAATGTGCATCCTCAAAAGGCTTGGTAAGGACCTCATTATAAAGAGAAAAATACTGAATCT

TTTTTAAGAGGCCTAAATGATGTGACATGGTGTACATTATGGTTAATTAGGTGTATCTTCAGCCAACATTTGATGTGTTGGAAACATTGACGGCTGATGT

AAATCGTGGGAAATATGCTTTGCGAAATGTGGTTCCACGCTTGGTGCTGCGCTCCACTTATGTTTCCTTGGCAACACTAATTGGGGCCATGCTCCCATTT

TTTGGGGACATTGTTTCTTTGGTTGGAGCGTTTGGATATACACCATTGGATTTTGTCCTGCCCATGCTTTTCTACCAACTTGTCTTCAAGCCCTCAAGAA

CAACGTATATATTTTGGCTCAATTGGGTCATCATAGTAAGTTTTTCAATAGTAGGGGTCATTGGATGCATAGCCACAATGCGTCATATTGTGATTGATGC

CAAAACCTACAAATTGTTTGCTGATGTGTAAAATGATGCAAGCCAAAAAATTGTTCAACATAATGGTCCTTGATTTGCTCAGCAATTACAACGCATTGTA

TATGACTTCATAGTTAGAACCTATACAGGGTAACAAGTTGTAAGTCTTTGGAATTTGCAAGCTTGAACCAAGAGCATCCTCCACACATCAGAACTTTTTT

TGTTTTGTGTAGCACATTGGTTAATCTGTTTGAGAGTGAAAGCTAGAATTGGGTGTCAACATTCTTTGTATGAGAGCTTGCTACACATAATGCAGAAATG

AGGCACCTGCATATTGAAAAGAATTGGTAGTCCTGTGGTGAAGTTAAAAGTGGAAAACTGTCAGCATGCACCAGTATGTCTCCAGTCACTAAAAGAGAAT

AAAATGATCATTTGG

>Sphfalx0005s0083

GAGCACAGGAAGGAAGGACGACGACAGGTCTCCACGCGAATTCCAATGCCCGAAAACGCGTCCCAAAAAGCAACCAAGCACGCGACCAGCAGCAACTGGT

GTGAGATCATGGTTCTCTTCAATGCCCATCAAATTCTTGTTACTTGTATCCAGAGGAGCAGCATCTCAAGATTGTTCACAATCGTAATTTTGTCAAGAAG

AACCACCAGCAGCAGCAGCAGCGATCCCTCCATCTCAAGATTGTTCACAATCGTAATTTTGTAAAGAAGAACTAGCAGCAGCAGATCCGAATACTTCTCA

TGGAGGGTCATGTTCCGCTTAGTGAGGTAACTATTTGTAGTTCAATTCCCCAGTTCTGCATTGAAATAATTGTTGCGTTTATATGATCTGTACAGTTAAT

TAATCTTGGGCGTCTTTGCTTGGGAAATAAATTTTAAACGAAAATTGGGTTCTGGTAGAATCTTGAAGAGCAGCCGGGTCCAATTACACTCAAAAAACTG

CAGCTTCAGACTTAATGAGAGTGTTACTGAGAACACGGCTGCCAGGTTTCTATATATCTAAACTGATCTCATCTTGTTTGTTGTATTTGCTTGTGGACAG

GATCTGGATTGGAATCGCAGGCTGAATGACGTGCTGCCTGTGACTGCGAGCAGGAACGCCAAATGGTATTACTCCATCTTCCATAATGTAACGGCTATAG

TGAGTGCTGGAGTTCTTGGCCTGCCCACTGCCATGGCAGATCTCACCTGGTAATATACACAATCACCAAACAGTACTACAACACATGAGAATATAACAAG

GAACATTAAAAGTTTAGTGATGATGTTCATTCCGAATTCCTATGACCGGCCTTGAGGAATCCTGATGAATATTCGGGATGTTTGACAAGTATGCGTTGTG

GATTTGTGGAGTGCTTCATGTGCAGGGGTCCAGGCATAGTCCTCTTGATCCTATCATGGGTCATCACCCTCTTCACCTTGTGGCAAATGGTAGAGATGCA

TGAGATGTTCCCCGGCAGACGTTTTGACAGGTACCATGAACTGGGCCAGGAAGCGTTTGGGAAGAGGTTGGGACGGTGGATCGTGATACCACAACAGCTT

CTTGTCCAGGTCAGCGTGGACATCGTGTACATGGTGGCCGGAGGCCAAGCGTTGAAAAACATCTACATGTTGAACTGCCATGGATGCAGTTCGAAAAATG

TGGGGAGCGATGATATTGCTGAAAAACAATACGAGTCGGCTTCCCTCTGGATTCTTATCTATGGCTCCGTGCACTTGCTACTCGTCCATCTCCCGAACTT

GAACTCCATTGCTGCACTCTCTTTGGCTGCAGCCATCATGTCAGTCAGGTCTGGAATTAGTCTACACTCAATGGCTCAATTAATATAAGCCCCGGATGCA

GCTCACACACAGATATATAGATTCCCCAGGATGTCTGGATCTGTTGCACATAGTAGAATTGCAAGTTTCCACGGATTATTGCATCTGATATGATTTGGTA

AACACCCCAGATGCAGATCTGCTACCCATGAGTACTAACTTAATACCCATTTTTTTGAACTTTTCTCAAGACTGAACAAAAAAAATTTGTGCACTGGACA

TGACCGCTTCTTCATTTGTGCAGCTACTCAACAATTGCGTGGGCCATACCCGTAAACAAGGGACATCACCAACCTCAGGACTACCATTTGCCATATTACC

CTGAAATTAGTCCAGCACCCGGGCCCGAGCCTCCGGCAACGGCAAATGCCCATACAGCACATCAAGTACTGAGTATTTTCAATGCGTTAGGGGTGATAGC

ATTTGCGTATGCAGGACACAACGTGGTTTTGGAGATTCAAGCCACGCTTCCCTCAACTCCCGAAAGGCCTTCCAAAATCGCCATGTGGAGAGGGGTCGTC

TGGGCTTACGTGATTGTGGCCGCTTGCTACTTCCCAGTGGCTATCATTTGTTACTGGGCTTATGGAAACCAGCTTGCAGCCTATTCCAATATCCTTCAAT

TCGAAGGCATGCTCCGACACAATTATAAGGGGATCCTCACTGCAGCTAATGTCATGTTGATTATCCACATTCTCGGAAGTTATCAGGTAGGCACATGAGT

TTCTTCCTCTCCTCTTTCTTCCTGACTTATTTCAACAATTCTCTTCTTATTTCTGTGCGTATTTCGAGGGTATACTCCTAGCTTTATATATATATGTTCA

CGTGTTCCCATAGACGAAAGAAAAAGACAACCTGTTTACTTGCACGGGTCCTTCTCCGGAGGTTGGGAAACATCCTTACTCAAGCTCACGTTCTAGAGTG

GTCCACAGTCAGGAGAACCCACTTGCATAGAACCTCCCCTACAACTACAACCTCAAAACATGTCTTTTACATTTTGTCCTCTTTGACTATAATGAAAAGA

TCGATTAAAAAATATATATAAATGATTTTTAAGAAGATTAAATAATAACTATAGGAATAAAACCAGTCATAAAAGACATGTTTTTAACTTTGAATTCAAA

CTGTATATGGTACAAACGAGTGCTGGTCTGGTTGCTCCTGCCTAGTATTGACCAGTGTTGTATGGGTAGATAGGTATTACCTTTTGAACAAACCAGAGAG

GTCCCCATGTTATGTTTTTTTTTTCCATTGGTGGCAGGTACTGATATTGGGTACTCTTTTAAGACTTCATACCAGTCTACTACTAGTATTAGGTCCAAGG

CTGACTTTTGCTTCGTTTAAGGCTGGCAGGTATAAACCTGGTATTACCTCAGGTCCCATAAGGGGGGGTGGGTTTGGTATAAGGTTGGTATTGCATAAGG

TCATATTGGACTGCTATGGTATTCAATAGGAGGAGTCATACCAGTATTATATACCAGGGTTGCATCCCATATACTAATACAACACGGATGTTGACACAGG

TACCAGTCAGTCTGGTATGATCTGAGGCAATACAAGCCTTATACCGGTTTTATATAGGGTGCACCTGAGGTAGTACCAGCCTTGTACCAGTTTTATATCA

ATCTCAAAAAACATATATGTATATCCTTCATATATCTAGCCAAAATTCAGTTGTAGTCCCAACATTGCACCAAAGACTGGTATGAAAGTTTTAAAAGAGT

ACCCAATACCAATATCAGCTGGATATGAAAAAAACCCCCAATACCGGGACCAGCCTGATATGTTCAAACTTGTCAATGCCAGATCAATACTTTGTAGGTA

CCAAGAGTGCAGCTGTTCTGGGAATTTGGGCTCTGCCTGATGTCGGATCCAAATACTCACAAAAGCCCTTTTGGAGTTCCTTACCCAGTGTAATGAACAC

TTGCAGAATTTCAACTATATAAACTATGATATTTGTGGGGGCGGGGGAAAAATCATGATTGAAATTCAGTGAGTTGATTTTGTGATTTGTTTTGCTGCTA

GACAGATCTATGCTATGCCAGTATTTGACATGCTGGAGACGGCGCTGGCGAAGAAATGGCTTCTTCCTCCTACCTTGAAACTTCGTCTGATCACTCGCAC

TACTTATGTCGGTAAGATTTTCAACATGACTAAACACTTGGCCCTGCATACATCTTCAAATCTGAAAAGCTCACAGTTGAGGGCATTCCCATAGTAAGGA

GCTTAGGATGATGAGATGGTAGTTGACTCGAGCACTGCATTTTGGGAATGTTGCCCCTCTGCCCCTCCACCATGCACTTTGCCCATCATAAAGCATGAGG

TGCAGAAAGTTATCATAAACCTAAACATATGCAGATCACAAGGTATCTGAGGAGGAGAGCTCTTACACCTACGTCTTATGAATGGGAGCATTATGGTTCT

TATGAATTTGCCTTCTTATAACATTGAACCTAAACATGGGAACAAATTTCTCCATGTGCTTTATGTTGGAGTTCTATTTGCCTTCTCATAGATTTGAATC

CTGGCTTCAAAACCTGCTGAAAGAACAAGAGGCCCCTTGAATTTCCTTAATGCTTAAAAATCGTGGTGGTTTATATTTGATTGCAAACTGCAGGTTTTAC

AATGTTTGTGGCTACAATCTTCCCATTTTTCAAAGCGTTGCTTGGATTCTTTGGAGGTTTCGCCTTTGCTCCAACAACATACTTTGTGAGTATATTATCT

ACATGCTTTTGGTTGAATCAAATATCAAGTCCATTGTTTCAGTCACATGACAAAAATTTCTTCGTAATCAATTAGGTTTTCAATTTTTTTGTTGACATGG

CATTCATTTATCTGGTTTGAGCAGAGCAGCTTGGCATTGTAGCCTGGCTGTTCCTATTATTGACCTCGTGAATAGGATTGAGTAGTCATTAGCTGAAAAC

TGCTAAAGAATCAGGTAAGAACCTTAACATTTCACCTGGCTGCAGCATGCCATCAACCTAGTCTCAACCAGAGTTCTTTTACCCAATTTTGTGAAATCAT

CCATAAGATGATTCAGCCAAATTTAGTTGTTAAAAAGTCTGAAAGTAAAAACATTAAAGCATTCATCAATATTGCTGGCTATCTACTATACATCTGAACT

TTTCTCTAAGATTTTGTAATCGGGTTTCATTGCAGTTACCTTGTTGTATATGGTTGATTGTCTGTAAGCCCAAACGATTCAGCATGTCATGGACAATAAA

CTGGGTGAGTGTTTTATTCATTAGTCTCTAGTTATAACATTTTGTGCACAATTCTTTCCCTTGTACTGAGGCTGCAGTTTTTTCACAGTTGTTTTCATAG

TTCTATTTCATGCAAGATTGAATCCCCTAGGCCTCATTAGTGGGCATTACTCTTGATGTTCTTGGTGGACAACTATCCAACATTAATTATACTATGGAAT

TACTGTAGCTTTGTGGCCACCAAAACTATGTACTTATTAAATGTTTTTCATATGGGATTTGTGCAGATTTGCATCATATTGGGGGTCCTACTCATGTTTA

CTGCAACCATTGGTGGCTGCTGGGCACTCGTGAACGAATGGAACAGCTACCAGTTTAGGAGGTTTTGGAAATGGCAAGATTGTCCAGGAGTAAACTCAGC

TCAATATTGTACTGCTCCAACCCCAGCTCCACACTAACTCATATTCCTTGACACTTGAAGTACACAGAATGATTATACAAAGGATGAGTGTAATTTAAAG

CTTTAGGAAGACAAAACCCTTGCAGAGAAGGGTACGTGCTGGTTCAACATTAGTGGGATTGCTATTCAGTACATTCAAGTTCTTAATTCAAAAGAAGAAC

ATACATTCCATTGTATCATTGAAGATTATGTGACTGGGTATCCCACTTCAGCCCAGTTCTCTCTGAAAGAACCAGCAGGTATTAACCAGGGTGATACCTG

AGGTCAGTACCTTACAAATCGGGTATCATTCTGAAGAGAACCAGTTGGTTAAGAACCAGTTTCTACTGAGAACCTTCATTAAATGAACTTTGAGTAATTA

TATTAATAACCCTGTGAATGGTTCTCAGGCTATGTATAGGTTTCTACATAGTTAGTTAGGTTTAGGTTAGGGTGGCGTTAGAGTTTAAGGTAGCACCATA

CCCAATCTAGCGAAGACGAAGCGTGAGCTTTAGAATAAGCTCAGTTCTCCACCTCTAGGCCCGCTTTCACACTTTGCTTTGTGTTCTCTTTGCTTTTGCT

CTTCTTTTTTTTCTTTTTTTCTTTTTAGGTTTCTTATAGTGTTGTAGGTGTGTGCAAGGAGTGCCCAGCTTTTTGAGGTCTGATGTTGTTTGTTGTGAGT

TTGAACTAATTGCAACCAAGTTGGTTTTGCAATTGTGTTTTCCTTTATGCACTTCAGGGTTTGCCAACCAACAATTTCTTTCTTGAAGTGATATCAATCA

TCCAGTCTTGAGTACAATCTTGATTAATGAGACATACACTTAGTCCATGCTATTGTGGGTTATGGTGTCACTTCAAAGGATCTACACCTACTTATAACAA

GTTTTGGTTTTATGTTGATGATTTGACTCGTTGTATGGGAGGCACTAGAAAAACTTACATTATTGATAGGTCAATTGTGTCTACGATATGACTTGTGCAA

GTAAGGAACAAGTTGACTTGTGATGAGGTGATATTACTAGAAGAAGTTGAATTTGTTCTATGCAAGGAATGAAAATGTTCTCCTTAAACACTTTTGGGAA

TGAGAGTTCTACTTTGGTGAATCAATCTTTGGACGTAATTTTTTGGCCAAAAGTTTACAATAATAAGAACTACTTGGTGGCCAACAATGTTGAAGCTTTC

AACCTTATAGAAAAATAGGTGGGAAACAAATAGGTTTTTGAAAGTTAATCTTTTGCATCCATAGATGTTCTTCCACCAAGGTATTGAGGAAGATGTTTAG

TTCAATCTGATCATGTAGGAAAATGACGCAACATCTGAAGATGATCTAAAGGACCAAGGGCTAAACCATCTTCTTCAACAAGAGTCCGCCAATCAAATTG

TGAATTTGATATTACAAGATCGGCACTGCCGACTGTTGGATGAATACATTATAAAGGGGATGATTATGCTAGTGGGCTTCAATAGGCAGTTGTTGAAGAA

TTAAAAAAACTCAAGGGAGTTTCTATCACTATGCCTATGCTTAATCCAATTCATCTGGATCATGAAAATATGCAACAAGTTGTTTCATCTTGAAATGAAA

GGTGGGATGAAATATGAAGCAGGATAAAGTTTGACTAGGCCCTAAGTGAAAACCAAGTTGAGGAGCTATGGACTCTATTGGAGGACTTTAAGGATGTCTT

TGCTTGGCACAAGGGGGAGTTGGGGTGCTGCACAATAGGGGAACATGCAATTGACACATAAGGTTTTCCCCATGTCGCACCACACCTAGGAGGTTGTTGT

ATTGGGAAGAAGCTAAAGTGAATAAACAAATTCAGGCATTGATCAAGCTTGGTAAGATGAAGAATAGTG

>Sphfalx0005s0085

AAACGCGTCCCAAAAAGCAACCAAGCACGCGACCAACAGCAACTAGTGTGAGATCATGGTTCTCTTCAATGCCCACTAAATTCTTGTTACTTGTATCCAG

AGGAGCAGCACATTGAGGCGCTATAAATTCCAATCTGAAACAGCAGCAGCAGTGCCAGCACTGATCGCTGCATCTCAAGATTGTTCACAAGCGTAATTTT

TTCAAGAAGAACTAGCAGCAGCAGATCCGAATACTTCTCATGGAGGGTCATGTTCCGCTTAGTGAGGTAACTATTTGTAGTTCAATTCCCCAGTTCTGGA

TTGAAATATTTTTTGTGTTTATATGATCTGTACAGTTAATTAATCTTGGGCGTCTTTGCTTGGGAAATAAATTTTAAACAAAAATTGGGTTCTGGTAGAA

TCTGGGTGGGTTCTAATAGAATTCTGGATTTGCTGTAAGGCTTTGGTTCATTTATATGTGTGTGAACTGGGAGGTAATGCCCAAGGTCGAGGGCATTACC

AGAATAAGGGTATAGCCCTGAGACAGCAGCTGAGGTGCTGCTCACATGCTGGTTATGCCCTAGTTCGATGACCTTACCAAACATAAAGCAACTGCCTCCC

CCCGGATCGATCGATTTGTGCCGACTAAAACGAATTTTTCCTCCTTAAAAAATAAGAACCCTCCAGTGTAACCGAGTGAATTTCACGTGTGCTCCCAATA

CACATGAGTGGGATTTCTGGCAGTGCTTCAAGTTCTCTATAATAGTTGTTACAGGAATTTCTAGAGTGCATCTCATGCTGATCAGACGAGCTAGTGATCG

GCGTTTCATATTGAAGAGCAGCCGGGTCCAATTACACTCAAAAAGCTGCAGCTTCAGACTTAATGAGAGTGTTACTGAGAACACGGCTGCCAGGTTTCTA

TATATCTAAACTTATCTCGTCTTGCTTGTTGTATTTTCTTGTGGACAGGATGTGGATTACAATGGCAAGCTGAATGACTTGCTGCCTGTCACTGCGACCA

GGGAAGCCAAATGGTATTACTCCACCTTCCATAATGTGACGGCTATAGTGAGTGCTGGAGTTCTTGGCCTGCCCACTGCCATGGCAGATCTCACCTGGTA

ATATACACAATCACCAAACAGTACTACAACACATGAGAATATAACAAGGAACATTAAAAGTTTAGTGATGATGTTCATTCCGAATTCCTATGACCGGCCT

TGAGGAATCCTGATGAATATTCGGGATGTTTGACAAGTATGCGTTTTGGATTTGTGGACTGCTCCATGTGCAGGGGTCCAGGCATAGTCCTCTTGATCCT

ATCATGGGTCATCACCCTCTTCACCTTGTGGCAAATGGTAGAGATGCATGAGATCGTGCCCGGGAAACGTTTTGACAGGTACCATGAACTGGGCCAGGAA

GCGTTTGGGAAGAGGTTGGGACTGTGGATCGTGTTACCACAACAGCTTCTTGTCCAGGTAAGCGTGGACATCGTGTACATGGTGGTCGGAGGCCAAGCGC

TGAAAAACATTTACATGTTGAACTGCCCTGGATGCAGTTCGAAAAATGTGGGGAGCGATGATATTGCTGAAATACAATACGAGTCGGTTTCCCTCTGGAT

TCTTATCTATGGCTCAGTGCACTTGCTACTCGTCCATCTCCCGAACTTGAACTCCATTGCTGCACTCTCTTTGGCTGCAGCCATCATGTCAGTCAGGTCT

GGAATTAGTCTACACTCAATGACTCAATTAATATAAGCCCCGGATGCAGCTCAGACACAGATATATAGATTCCCCAGGATGTCTAGATCTGTTGCACATA

GTAGAATTGCAAGTTTCGACGGATTATTGCATCTGATATGATTTGGTAAACACCCCAGATGCAGATCTGCTACCCATGAGTACTAACTTAATACCCCTTT

TTTTGAACTTTTCTCAAGACTGAGCAAAGAAACTTTGTGCACTGGACATGACCGCTTCTTCATTTGTGCAGCTACTCAACAATCGCGTGGGCCATACCCG

TAAACAAGGGACTGATCCAACCTCCGGACTACCATTTGCCATATTACGGTGAACCTAGTCCAGCACCCGGGCCCGAGCCTCCGGCAACGGCAAATGCCCA

TACAGCACATCAAGTACTGAGTATTTTCAATGCGTTAGGGGTGATAGCATTTGCGTATGCAGGACACAACGTGGTCTTGGAGATTCAAGCCACCCTTCCC

TCAACTCCCGAAAGGCCTTCCAAAATCGCCATGTGGAGAGGGGTCGTCTGGGCTTACGTGATTGTGGCCGCTTGCTACTTCCCAGTGGCTATCATTTGTT

ACTGGGCTTATGGAAACCAGCTTGAAGCCTATTCCAATATCCTTCAATTCGAAGGCATGCTCGGACAGAATTATAAGGGGATCCTCACTGCAGCTAATGT

CATGTTGATTATCCACATTCTCGGAAGTTATCAGGTAGGCACATGAGTTTCTTCCTCTCCTCTTTCTTCCTGACTTATTTCAACAATTCAGTCGTAGACC

CAATATTGCACCAAAGACTGGTATGAAAGTTTTAAAAGAGTACCCAATACCAATATCAGCTGGATATGAAAAAAACCCCCAATACCGGGACCAGCCTGAT

ATGTTCAAACTTGTCAATGCCAGACCAATACTTTGTAAGGACCAAGAGTGCAGCTGTTCTGGAAATTTGGGCTCTGCCTGATGTCGGACCCAAATACTCA

CAAAAGCCCTTTTGGAGTTCCTTACCCAGTGTAATACACACTTGCAGAATTTCAACTATATAAACTATAAACAAACAGCTGTGACTCAATTGAGAGGATG

GGAGGGAGCGCTGGCTTAATGTTCCTCATATTTTAGGAACATTATGGGTAAGGTGCCATCCTTGGCGAGGCAGAAAATCTATTGAGGATAAAGGTGCCCT

TAAAATATACTAGGAAAATTTTATTGGATCCTGATGGGTAAGGTTCCATCTCTCTCCAGGGTTAGGACAGTCCCAGAATAAGGGGCCTAGGACAATGCAA

TAGCTGAGTATCTATGACTTGTGATCTATACATATGTTTACATTCATGATTAATTCATCTCATGTGGATGATCACGTAACTTCCTAAGTCACATGTGGTA

CTAAAGTAAAATTTGGCAAGTCCTCTTGTGGATGATCACGCAACTACCCACCTTACAAAATTCAAAAAAGAAAAACCCTGGAGGATGATATTTGTAGGGG

GGAAAAAATCATAATTGTAATTCAGTGAGTTGATTTTGCTGCTGGACAGATCTATGCTATACCAGTCTTTGACATGCTGGAGACAGTGCTGGCGAAGAAA

TGGCTTCTTCCTCCTTCCTTGAAACTTCGTCTGATCACTCGCACTACTTATGTCGGTAAGATTTTCAACATGACTAAACACTTGGCCCTGCATACATCTT

CAAATCTGAAAAGCTCACAGTTGAGGGCATTCCCATAGTAAGGAGCTTAGGATGATGAGATGGTAGTTGACTCAAGCATCACATTTTGGGAATGTTGCCT

CTCTGCCCCTGCACCATGCACTTTGCCCATAGTAAAGCATGAGTGCAGAAAGTTATCATAAACCTAAACATATGCAGATCACAAGATATCTGAGGAGGAG

AGCTCTTACACCTACTTCTTATGAATGGGAGCATTATGGTTCTTATGAATTTGCCTTCTTATAACATTGAACCTAAACATGGGAACAAATTTCTCCATGT

GCTTTATGTTGGAGTTGTATTTGCCTCCTTATAGATTTGAATCCTGGCTGCAAATTGCCTTCCAATTCAAAACCTGCTGAAAAAACAAGAGGCCCCTTGA

ATTTCCTCAATGCTTAAAAATCGTGGTGGTCTATATTTGATTGCAAACTGCAGGTTTTACAATGTTTGTGGCTACAATCTTCCCATTTTTCCAAGCGTTG

CTTGGATTCTTTGGAGGTTTTGCCTTTGCTCCAACAACATACTTTGTGAGTGTATTATCTACATGCTTTTGGTTGAATCAAATTTCAAGTCCATTGTTTC

AGTCACATGACAAAAATGTGTTCCTAATCAATTAGGTTTTCAATTTTTTTGTTGACATGGCATTCATTTATCTGGTCTGAGCAGAGCTGCTTGGCCTTGT

AGCCTGGCTGTTCCTATTATTGACCTCATGAATAGAAACGAGTAGTCATTAGCTGAAAACTGCTAAAGAATTAGGTAAGAACCTTAACATTTCACCTGGC

TGTAGCATGCCACCAACCTAGTCTCAACCAGAGTTCTTTTTCCCAATTTTGTGAAATCGTCCATAAGACGATTCAGCCAAATTTAGTTGTGTAAAAAGTA

TGAAAGTAAAAACATTAAAGCATTCATCAATATTGTTGGCTATCTACTATACATCTGAACTTTTCTCTAAGATTTTGTAATCGGGTTTCATTGCAGTTAC

CTTGTTGTATATGGTTGATTGTCTGTAAGCCCAAACGATTCAGCATGTCATGGACCATAAACTGGGTGAGTGTTTTATTCATTAGTCTCTATTTATAACA

TTTTGTGCACAATTCTTTCCCTTGTACTGAGGCTGCAGTTTTTTCACACTTGTTTTCATAGTTCTATTTCATGCAAGATTGAATCCCCTAGGCCTCATTA

GTGGGCATTACTCTTGATGTTCTTGGTGGACAACTATCCAACATTAATTATACTACAGAATTACTGTAGCTTTTTGCCCACCAAAACTATGTATTTATTT

ATTTTTTTCGTATGGGATTTGTGCAGATTTGCATCATATTGGGGGTCCTACTCATGTGTGCTGCAACCATTGGTGGCTTCTGGGCAATCGTGCATGAATG

GAGCAACTACCAGTTTAGCGGGTTTTGGAAATGGCAAGATTGTCCAGGAGTAAACTCAGCTCAAAATTGTACTGCTCCAACCCCAGCTCCACTCTAACTC

ATATTCCTTCACACTTCAAGTACACAGAATGATTGTACAAAGGATGAGTGTAATTTAAAGCTTTAGGAAGACCAAACCTTTGCAAAGAAGGGTACATGCT

GGTTCAACATTAGTGGGATTGCTATTCAGTACATTCAAGTTCTTAATTGAAAAGAAAAACATACATTCCATTGTATCATTGAAGTAAATGTGACTGGGTA

TACCACTTCAGCCCAGTTCTCTCTGAAAGAACCAGCAGGTATTAACCAGGGTGATACCTGAGGTCAGTACCTTACCGACCGGGTATCATTCTGAAGAGAA

CCAGTTGCTTAAGAACCAGTTTCTACTGAGAACCTTCATTAAATGAACTTCAAGTAAATTATATTAATAATCCTGTCTATGGTTCTCAGGCTGTATATAG

GTTTCTAGGTAGTTCGTTAGGTGTAGGTTAGGTTGGTGATAGAGTTTAAGGTAGCACCATAGCCAATCTAGCGAAGAGGGAGAGCGAGCTTTAGAATAAG

CTCAGTTCTCAACCTCTAGGATCCACTTTCACACTTTGCTTTCTGTTTTCTTTGCTTTTGC

>Sphfalx0007s0031

ACAAATCACTTTGCGGAGAAGAAGGGAAGCTCCTGTGTCCTCCTGCGGGTTTTGCGTTTTGCAGGCACCGCCGCGCGGCACGACTTCCCGAGCCGGCGCT

ACAAAGGACTTGCTGAGATTTGGACGTCGTCGCTTGTTCATGACAGGGCTATTACTCGACTCTCGCCTTCCACCACCACCCCGAGTTCCCAGTTCCGTGA

AACCCACCAGTCTCGGGGAGCCGAAGAACAATCGATCCAAGACCTGATAAGGTAAAATTCATTCGCTGCTGAGCATCAAGTTTCTTGAAATTGCTGTTAT

GTAGACTTCTGCAACAGAATTTTTCAGCTCTCGTTCTGCAGTTGGAGTCTAAACTGGCGTTGTTTTGTGAATCTGATCATGAAGCTGGCGTCAGTTGTTC

TGTACTGGTGTGTTCGTTTTCTGTACATTAACGTGTAACTCCGGTGTGAGACTCTTTGTTTTCGATCGAAGGCGATAAGGTATTTCGGCGCTGAGCATCA

AGTTTCATGAATTTTTTGTTGTGTAGACTTCTGAAAGCGAAATTGTCAACTCTCACTCTTCGACTGGAGTCTAAACTGGCGTTCTTTTTTTGAATCTAAT

AATGAAGCCGTCAGTTGTTCTGTTCTAGTGTGTTTGTTTCCTTTATGACTCTTTGCTATCGATCGAAGGTGGTAAGGTGATAAGGTGATTCGGTGCTCGG

CATCAAATTTCATGAAATTGTCGTCATGTAGACTTCTACAAGAGAAATTGTTCATTCTCGCTCTTCCGCTGGATTCTAAGCTGGCGCTATTTTGTGAATC

TAATAATGAAGGCGTCAGTTGTTCTGTTCTGGTGTGTTTGCTTCTTTTATATGCACGTATAACTCCAGGCTGAGACTCTTTGCTATCCATCGACGGAGAT

AAGGTGCTGAGCATCAAGTTTCATGAAATTGTTGCCATGTTGACTTCTGTAAGAGAAATTGTCAATTCTTGCGCTTACGCTGCTGTCTAAAACTAGCGTT

GTTTTCTGAATCTGATAATGAAGCCGTCAGTTGGTCTACGCTGGTGTGTTTGTTTTCTTTATATGCCACTTGTAACTCCCATGTGAGACTCTTTGCTATC

TATCAAAGGTGATAAGATAGTTCGGTGCTGAGCATCAAGTTTCATGAGATTGTTGTCATGTAGATCTGTAAGGGAAACTGTTCAATTCTCGCTCTTCCGC

TGGAGTCTAAACTGGCGTTGTTTTGTGAATCTGATAATGAAGGGGTCAGTTGTTCTGTTCTGGCGTGTTTGTTTTCTTTATGACTCTTTGCTATCGATTG

AAGGTGATAAGGTGAATCGGTGCTGAGCATCAAGTTTCATGAAATTGCTGTTAAGTAGACTTGTCTAAGGGAAATTGTTCAATTCTCGCTCTTCTGCGGG

AGTCTAAGCTGGCGTTTTTTTGTGAATCTGATAATGAAGACGTCAGTTGTTCTGTGCTGGTGTGCTTCTTTCTTTTATGTGCACGTATAACTCCAGTGCG

AGCTTCTTTGCTCTGTATGGCCGAATGAATATATGCTGGGTGCTTTGACAGTGTGAATTTTTGCCCCCTTGTTTCCTTGCGAGCTGCAGCTTGAAGTCTA

TTTCCTGCGCGTTTTGTTGTCTAAAGATTTGTTGTTTTGCCCGTAACTAAAAGACGAGCAGCCTGCAAGAAATATTAGGAGCTGTGAAGACCGTAAAACA

GAGAGCGGTCTCATTTGCCCATTCCTGTAGGGATTCAATTTGAGTCAAGGTGGTAGACTTCATTCCCTTAATCCATCAAGTCATTCTCATCAGGTAGCTA

AAGAGAATTGAAGCAAGATTTGTGGAATCATGAATACAGGAGATTGCTGTGGCTGACTGCTGTGAAATTGTAGGCAATGTTTCCCAATTGCTGAGCTCTT

GTCTCCTCCAATCTGCTTGATTCCTTTTTTTATCCGTTTTGCATACAATTGAGAGCTTTGAACATCTTTAGAGTTGCCTTGAATATGTTCAGGAATGTGC

ACATAGTCTGTCTTTTCATTCTGTTCTTGAAATGAGACATAGATGAGTTCTATACAAAGATTTGGCCTTGGCTCTTGAGAATTTGGCTATCCTCTCCTGC

AATTGAGCTTGTGTCACCATCTTCCACTTAGAATCCAGTATGTGGACTGTAGATTCCTTTTAGGAATTTGTGGTCATGTTTAGCATGAGAGACTCTGCCA

TTTCTGCAGCAACCTAGTGAGAGAGAGACAGAAATGTCTGAGAAGCACGTGCAGATGATTCTTGGACACTTCTTTTATTTTTCTGTCTGCTTAATACAGG

TGAGCTAGTAGGATGTGTTGCATACTGTATGTTGTTAAAGTATGAAGCTTGATAACCCACCTCTAACATCTGGAAGAATGTTTGACAGGAAAGTACTGCA

GCAGTGAGAGGATAAAGATGGGGTGGGAAGAAGATGGAAAAATTGCACAGGCTGAGGATGGAGCTGCAAATGGGGACAGTAATGGCTCTGCTGTCTATGA

TCCGACAAAATTTGATGAGGATGGCAAACCACGACGCAAAGGCATATATCCTCTCTCTCTTCAAGGGTTCCCCTTGCTTAATTTGGATCCTCAGATCCTC

TTAGCAAAGGAAAGTTCATTTCTTTTGCAAAAAACAATGTGAACTATTCACCAAAGATTGGCTGAAATTGAGGTTCACAAACTCTGGAGGTGTTTGTCTT

TACAATAGTGACTAGCACATTTTGGGTTCTCTGAAAAGAATGGACATGGGATGTGAACATTGACTGACATATGTGAGTGGTTCTGGAAGTGCAGGAAATG

TCATGACAGCGTCAGCACATGTGATCACAGCAGTTATAGGATCAGGGGTGCTGTCATTAGCATGGAGTATCGCACAAATGGGTTGGATTGCAGGACCTTC

TGTGCTGCTATTGTTTGCAGCTGTCACGTACTACACCTCTTGCTTGCTAGCTGATTGCTACCGGTACCCTGACCCTGTCACTGGCAAGCGCAACTACACC

TACATGGAGACTGTGCAAGCCAATCTGGGTGTGTGTTGAGTTCTCTTTAGAAAACCTTGTGCCTGAGATAGTGTGTGTCATGTTGCATTGGAAGGAGACA

TTGTGATTCCTGAAAATACATCAGAGTGGCTTAAGAAGGAAAAAGTTAGAAAACCTTAAGAAACCCCAACAAAAGCTGTGCAATGAGATAGAAGATTGAA

TGGAAAGATGTCTTAAGGCAGTCATCACACAAAGAATTGATGGTACATCCTATTCTTGTCTTTGGAATGTGGGAGTGAATAGGTAGTACCTTCATAACTG

ATTTTCGTCAACTGGCAGGTCCAAAGCAAGTGTGGGCCTGTGGTTTAGTGCAGTACTCGAATTTGCTGGGGACTTCTATTGGCTACACCATCACTGGTGC

ACAAAGTGCAAGGTAAGTTGATCTTCTTCTCTTTTATCTATCTCACTAGAGTTTCACATTCACAGTAGCTTGAACGGCAGAGCCTCTCTTTGGAAAGGCT

TTTTTTGTTCTACTCTGTACTCAGAATGGTTAACTTCTAACATGTGGCCTCCAATGTGGGTAGCTTAGACGGTGGAGGATGAATTTTGTATTTATGCCAC

ATAAATCCAAACCCTAATCATAATACTTACATGCCTTTTCCATCACCCCTCCATAGAACGAATAGTATACAGCTGGCCTCAATCTGTATGAACTGTGTAG

AGTAGGAAATGACTATGTATCTGTAGGTTTTCAGTACTTCATTGCTATGATAGAGTTGTGTAGCTACAGTTGCCTATTCTTGAAACAACACTTTCTGAAG

GCAGGGTGTCAGGCTGAACAAGACTAGCTTCATTTGTAAACCTCATTGGAGGGATCACCTTGTGCAAACAATACGAGCTTTAACGTTATCTGCAATGGTG

GTTGCAGAGCCATCACAAAGTCTAACTGCTTCCATTCGAATCCAGATTCACCTTGCCTAAGATCAAATAATGGGTACATGATAAGCTTTGGGATAGTGCA

ACTTGTCCTCTCACAGATCCCCAATTTTGGCGAATTATGGTGGCTTTCTTATGTTGCTGCTGGCATGTCCTTCATTTACTCTACAATTGGCTTGGGCCTT

GGCATAGGGAAGATTGCAGGTGGGGGTCATCAATTGTATCTACTAAGTTTTGCGCTTTTGTAATAGACAAGAATAGCTTTTATTGTGATGTAATGTCTCA

CTGCAGTGATTCTGCCTTACGTATGTTTGAGAGAACTCAGTTCCTTGACAGGATCTTGAGAGAGGCCAAGTGTGGAAACTCCAGATGTAGGGAAAAATTG

TTGCATTCTTTTGTGGAAGATTGTTCTAATCTTTTTGGGAGTCCTGAAGCTTCCATCTTTCAATTGACTAGCAAAATACTGACCTGGAGGTGTTCCATCG

TGACAGAGGGAGGTTACTCACATGGGTCCATATCTGGCACTTCAATTGGTGATCCTAGTTTGGCTGGTTATAACACTCGTGCTCAAAAAACTTGGAATGT

ATTTAATGCCCTTGGAGATATGGCTTTTGCTTATTCCTTCTCACTGATTCTCATTGAAATCCAGGTATTGGATATGCCTATCTTACTACTTTACTGTGAC

AGGGCAGGTTGAATACTGGATGTGCATTATTGGGTCTAGTGGTTCATTTTAATCCATCAATTCGGCTATTTAAGAATATAAGGTAGTTACAGAACCTGAG

GCTTTGCAAAGCATTTGTGTGAGTTCTCCCTCATTCTTCATATGCTTCCTGCTTCATTCATGATCAGTCTGTGGAATCAACCAACGCAGGGAGATTTCCA

AACAAGTTCTGGGCAGTGAATGACCTGTATGTCAAGCCTTCACAGAGACTACAGATTATGACTACCATATTTGCTTTTTGTGCAGGATACACTAAAAGCT

CCTCCTGCTGAGAACAAGAGCATGAAGCGAGCCGCACTTATAGCAATTCTTACCACCACTGGGTTCTACATGTCAGTTGGCTGTGCGGGTTATGCAGCCT

TTGGCAATGCTTCACAAGGAAATCTTCTGACAGGCTTTGGGTTCTATAACCCCTACTGGCTTGTTGACTTTGCCAATGCATGTGTTGTTGTTCATCTCAT

TGGCGCCTATCAGGTAGGTTTGGCATTTTGTGTCTCTCCATTGTCATACAAGAGTATCCTTAATACACACAAAAGGATCCAATTTGGCAGGCTGGTAGCA

TTCTTGGGAGTGTGTGTTTGTAACTGATGTTTTGACCTTAGCGTACTGTGTGTGTCACACAGGTGTATACACAACCACTATATGCATTTATTGAGGAATG

GGTGTCCAGCAAGTTTCCCAAGAGTAACTTTATCAACAAGGAACACTATGTGAAGCTCCCCTTTGGTGAGCCATTGCCAATCAACCATTTCAGGTTGGTG

TGGCGCTCTATCTATGTGGTAATGACCACAATTGTTTCCATGCTGCTTCCATTTTTTAACGACATACTGGGTTTGATTGGAGCTTTTGCCTTCTGGCCCC

TGACAGTGTACTTTCCAGTTGAGATGTACATTCATCAAAAAAGACTCCCAAGATGGAGCCAAAAATGGATTCTTCTGCAGTCACTAAGTGTGGTGACTTT

TGTAGTATCACTTGCTGCAGCAATTGGATCTGTGGCAAGTATAGTCTCAGATGTGCAGGGCTACAAACCTTTCAGTAATGATGCCTAGGAGATTGAACCC

TGCTTAGTGAAACAGGGAACATGTATACAAGATTATTATCAGGTCACCATTGGAGCATTACCACAACTCAATTCAGGTGTGCAAATAAAAATGTGATCCC

TACAGGCTGATATGAATCAGGTGAGCTCAAGTTGATTTTGATCCATCCGTCCATCCATCTATCCTCCAGCCTCTTTCTCATTTCTAGTGCATCTCTAGTC

CACTTCTGGACTGTCACATGGGAGTTGTGGAAATGTGAGGATTGTGATTATAGGTATTAGTTATGATCAGAACAAGATTCCATCCGTCTTA

>Sphfalx0007s0033

GAAGCTCCTGTGTCCTCCTGCGGGTTTTGCGTTTTGCAGGCCCCGCCGCGCGGCACGACTTCCCGGGCCGACGCTACAAAGGACTTGCTAAGATTTGGAC

GTCGTCGCATGTTCATGACAGGGCCATTCCTCGACTCTCGCCTTCCACCACCACCCCGACTTCCCAGTTCCGTGAAGCTCACCACTCTCCGGGAACCCAA

GAACAAACGATCCAAGACCTGATAAGGTAAAATTCCTTCGCTGTTGAGCATCAAGTTTCATGAAATTGTTGTCATGTAGACTTCTGCAACAGAATTTTTC

AGCTCTCGTTCTTCGGTTGGAGTCTAAACTGGCGTTGTTTTGTGAATCTGGTCATGAAGCTGGCGTCAGTTGTTGTGTACTGGTGTGTTTGTTTTCTGTA

TATTGACGTGTAACTCCGGTGTGAGACTCTTTGTTATCGATCGAAGGCGATAAGGTATTGTTCTGTAGACTTCTGAAAGCGAAATTGTCAACTCTCACTC

TTCGACTGGAGTCTAAACCGGCGTTCTTTCGTGAATCTAATAATGAAACCGTCAGTTATTCTGTTCTGGTGTGTTTGTTTCCTTTATGACTCTTTGCTAT

CGATCGAAGGTGATAAGGTGATTCGGTGCTCAGCATCAAATTTCATGAAATTGTCGTCATTTAGACTTCTACAAGAGAAATTGTTCACTGTCGCTCTTCC

GCTGGATTCTAAGCTGGCGCTATTTTGTGAATCTAATAATGAAGGCGTGAGTTCTTCTGTTCTGGTGTGTTTGTTTCTTTTATATGCACGTATAACTCCA

GGCTGAGACTCATTGCTATCCATCGACGGAGATAAGGCGAGTCGGTGCTGAGCATCAAGTTTCATGAATCGTTGTCATGTTGACTTCTGTAAGAGAAATT

GTCAATTCTTGCGCTTACGCGTCTATAACTGCCGTTGTTTTCTGAATCTGATAATGAAGCCGTCAGTTGGTCTGCGCTGGTGAGTTTGTTTTCTTTGTAT

GCACTTGTAACTCCCATCTGAGACTCTTTGCTATCTATCAACGGTGATAAGATAATTCGGTGGTGAGCATCAAGTTTCATGAGATTGTTGTCATGTAGAT

CTGTAAGGGAAATTGTTCAATTCTCGCTCTTCCGCTCCAGTCTAAACTGGCATTCTTTTGTGCATCTGATAATGAAGGCGTCAGTTGTTCTCTTCTGGCG

TGTTTGTTTTCTTTATGACTCTTTGCTATCGATTGAAGGTGATACGGTGAGTCGGGGCTGAGCATCAAGTTTCGTGAAATTGCTGTTAAGTAGACTTGTC

TAAGGAAAATTGTTCAATTCTCGCTCTTCCGCGGGAGTCTAAGCTGGCGATTTTTTGTGAATCTGATAATGAAGACGTCAGTTGTTCTGTGCTGGTGTGC

GTGTTTCTTTTATGCGCACGTATAACTCCAGCGTGAGCTTCTTTGCTCTGTACGGCCGAATGAATATATGCTGTGTGCTATGACTCTGAATTTTTGCCCC

CTTCTTTCCTTGCGAGCTGCACCTTGAAGTCTATTTCCTGCGCGTTTTGTTGTCTTAAGATTTTTTTTTGCCGTCACTGAAAGACGAGCAGCCTGCAAGA

AATATTAGGAGCTGTGAAGACCGTACAACAGAGGGCGCCCTCATTTGCCCATTCCTGTAGGGATTCAATTTGAGTCAAGGTGTTTGACTTCATTCCCTTA

ATCCATCAAGTCATTCTCATCAGGTAGCTAAAGAGAATTGAAGCAAGATTTGTGCAATCATGAATACAGGAGATTGCTGTGGCTGACTGCTTTGAAATTG

TAGGCACTATTTCCCAATTGCTGAGCTCTTGTCTCCTCCAATCTGCTTGATTCCTTTTTTTATCCGTTTTGCATACAATTGAGAGCTTTGAACATCTTTA

GAGTTGCCCTGAATATGTTCGGGAATGTGCACATAGTCTGTCTTTTCATTCTGTTCTTGAAATGAGACATAGATGAGTTCTGTACAAAGATTTGGCCTTG

GCTCTTGAGAATTTGGCTATCCTCTCCTGCAATTGAGCTTGTGTCACCATCTTCCACTTAGAATCCAGTATGTGGACTGTAGATTCCTTTTAGGAATTTG

TGGTCATGTTTAGCATGAGAGGCTCTGCCATTTCTGCAGCAACCTAGTGAGAGAGAGAGAGAAATTTCTGAGAACCATGTGCAGATGATTCTTGGACACT

TCTTTTATTTTTCTGTCTGCTTAATACAGGTGAGCTAGTAGGATGTGTTGCATACTGTATGTTGTTAAAGTATGAAGCTTTATAACCCACCTCTAACATC

TGGAAGAATGTTTGACAGGAAGGAACTGCAGCAGTGAGAGGATAAAGATGGGGTGGAAAGAAGATGGAAGAATCGCACAGGCTGAGGATAGAGCTGCCAA

TGGGGACAGTAATGGCTCTGCTGTCTATGATCCGACAAAATTTGATGAGGATGGCAAACCACGACGCAAAGGCATATATCCTCTCTCTTTTCAAGGGTTC

CCCTTGCTTAATTTGGATCCTCAGATCCTCTTAGCAAAGGAAAGTTCATTTCTTTTTTCAAAAAACAATGTGAACTATTCCCCAAAGAATGGCTGAAATT

GAGGTTCACAAACTCTGGAGGTGTTTGTCTCTACAATAGTGACTAGCACATTTTGGGTTCTCTGAAAAGAATGGACATGGGATGTGAACATTGACTGATG

TATGGGAGTGGTTCTGGAACTGCAGGAAATGTCATGACAACATCAGCACATGTGATCACAGCAGTTATAGGGTCAGGGGTGCTGTCATTAGCATGGAGTA

TCGCACAAATGGGTTGGATTGCAGGACCTTCTGTGCTGCTATTGTTTGGAGCTATCACATACTACACCTCTTGCTTGCTAGCTGATTGCTACCGGTACCC

TGACCCTGTCACTGGCAAGCGCAACTACACCTACATGGAGACTGTGCAAGCCAATCTGGGTGTGTTGAGTTCTCTTTAGAAAACCTTGTGCCTGAGATAG

TGTGTGTCATGTTGCATTGGAAGGAGACATTGTGATTCCTGAAAATACATCAGATTGGCTTAAGAAGGAAAAAGTTAGAAAGACTTAAGAAATCCCAACA

AAAGCTGTGCAATGAGATAGAAGATTGGAATGGAAAGATGTCTTAAGGCAGTCATCACAAACAGAATTGATGGTACATCCTATTCTTGTCTTTGGAATGT

GGAGTGAATAGGTAGTACCTTCATAACTGCTTTTGGTCAACTGGCAGGTCCAAAGCAAGTGTGGGCCTGTGGTTTAGTGCAGTACTCGAATTTGCTGGGG

ACTTCTATTGGCTACGTCATCACTGGTGCACAAAGTGCAAGGTAAGTTGATCTTCTTCTCTTTTATCTATCTCACTAGAGTTTCACATTCACAGTAGCTT

GAATGGCAGAGCCTCTCTTTGGAAAGGCTTTTTTTGTTCTACTCTATACTCAGAATGGTTAACTTCTAACATGTGGGTAGCTTAGACACTGGAGGATGAA

TTTTGTATTTATGCCACATAAATCCAAACCCTAATCATAATACTTACATGCCTGTTCCATCACCCCTCCATAGAATGAATAGTATACAGCTGGCCTCAAT

GTGTATGAACTGTGTAGAGTAGGAAATGACTATGTATCTGTAGGTTTTCAGTACTTCATTGCTATGATAGAGTTGTGTAGCTACAGTTGCCTATTCTTGA

AACAACACTTCCTGAAGGCAGGGTGCCAGGCTGAAAAAGACCAGCTTCATTTGTAAACCTCATTGGAGGGATCACCTTGTGCAAACAATACGAGCTTTAA

CATTATCTGTAATGGTGGTTGCAGAGCCATCACAAAGTCTAACTGCTTCCATTCGAATCCAGATTCACCTTGCCTAAGATCAAATAATGGGTACATGATA

AGCTTTGGGATAGTGCAACTTGTCCTCTCACAGATCCCCAATTTTGGCGAATTATGGTGGCTTTCTTATCTTGCTGCTGCCATGTCCCTCATTTACTCTA

CAACTGGCTTGGGCCTTAGCATAGGGAAGATTGCAGGTAGGGGTCATCAATTGTATCTACTAAGTTTTGCCCTTTTGTAATAGACAAGAATAGCTTTTAT

TGTGATGTAATGTCTCACAGCAGTGATTCTGCCTTACCTATGTTTGAGAGAACCCAGTTCCTTGACAGGATCTTGAGAGAGGCCAAGTAACTGCATCCCA

AAGTGTGGAAACTCCAGATGTAGGGAAAAATTGTTGCATTCTTTTGTGGAAGCTTGTTCTAATCTTTTTGAGAGTCCTGAAGCTTCCATCTTTCAATTGA

CTAGCAAAATACTGACCTGGAGGTGTTGCAATGTGACAGAGGGAGGTTACTCACATGGGTCCATATCTGGCACTTCAATTGGTGATCCTAGTTTGGCTGG

TTATAACACTCGTGCTCAAAAAACTTGGAATGTATTTAATGCCCTTGGAGATATGGCTTTTGCTTATTCCTTCTCAATGATTCTCATTGAAATCCAGGTA

TTGGATATGCCTATCTTACTACTTTACTGTGACAGGGCAGGTTGAATACTGGATGTGCATTATTGGGTCTAGTGGTTCATTTTAATCCATCAATTCGGCT

ATTTAAGAATATAAGGTAGTTACAGAACCTGAGGCTTTGCAAAGCATTTGTGTGAGTTCTCCCTCATTCTTCATATGCTTCCCGCTGGGCAAAGTAGCAC

AAATCATTCATGATCAGTCTGTGGAATCAACCAACGCGGGGAGATTTCCAAACAAGTTCTGGGCAGTGAATGCCCTGTATGTAAAGCCTTCACAGAGACT

ACAGATTATGACTACCATATTTGCTTTTTGTGCAGGATACACTAAGAGCTCCTCCTGCTGAGAACAAGAGCATGAAGCGAGCCACACTTATAGGAATTCT

TACCAGCACTGGGTTCTACATGTCAGTTGGCTGTGCGGGTTATGCAGCCTTTGGCAATGCTTCACAAGGAAATCTTCTGACAGGCTTTGGGTTCTATAAC

CCCTACTGGCTTGTTGACTTTGCCAATGCATGTGTTGTTGTTCATCTCATTGGCGCCTATCAGGTAGGTTTGGCATTTTGTCTCTCTCCATTGTCATACA

AGAGTATCCTTAATACACACAAAAGGATCCAATTTGGCAGGCTGGTAGCATTCTTGGGAGTGTGTGTTTGTAACTGATATTTTGACCTTAGTGTACTGTG

TGTGTCACACAGGTGTATACACAACCACTATATGCATTTATTGAGGAATGGGTGTCCAGCAAGTTTCCCAAGAGTAACTTTATCAACAAGGAACACTATG

TGAAGCTCCCCTTTGGTGAGCCATTGCCAATCAACCATTTCAGGTTGGTGTGGCGCTCTATCTATGTGGTAATGACCACAATTGTTTCCATGCTGCTTCC

ATTTTTTAACGACATACTGGGTTTGATTGGAGCTTGTGGCTTCTGGCCCCTGACAGTGTACTTTCCAGTTGAGATGTACATTCATCAAACAAGACTCCCA

AGATGGAGCCAAAAATGGATTCTTCTGCAGTCACTAAGTGTGGTGACTTTTGTAATATCACTTGCTGCAGCAATCGGATCTGTGGCAAGTATAGTCTCAG

ATGTGCAGGGCTACAAACCTTTCAGTAATGATGCCTAGGAGATTGAACCCTGCTTAGTGAAACAGGGAACATGTATACAAGATTATTATCAGGTCACCAT

TGGAGCATTACCCCAACTCAATTCAGGTGTCCAAATAAAAATGTGATCCCTACAGGCTGATAT

>Sphfalx0007s0047

GTTGCCACAACTACTGTTGTTGTTGTCGTGTTGCTTTCGCTGCCGCGCCGCACAAAAGTTGCGTCACTCCTCGACTTCACACTACTGTGTGTGCTACTAC

TATTGCTACTGCTACTGCTTATTCAGAAGCTGGTTCGAGCAGCACCCGCTTGAGCTTCTTCGACGGCTTGTGACTCCCGCCCCGGTCTGCAGTAGCGCGG

AAACAGAAGAACACTTTGTAGCCATTTTACATGTTAGGGGAAAAAGATAAGATCTGCAGCCTAGCAGGAGAACTGGAGTGTAGTGCATGAGCCCCACAGA

AGCTTGCATTTTCTCCCTGTACCCCTCACACTCGCGACACCCTGCTGCGGCTGCTTTGACATACGTTCTAGACGGAATTTTTATGGTTAGTGGAATTGCG

ACATTTCGGAGGTAGTTTTAAGTAATGTGTTTGTTTTGATCTCGGTAGCTGTGATGTGGTTTTACTTGAGTATTGGGTTAGAGGAAGTAGTAGCAGAGGA

GAGAGAGCAGATGGAAGAAGCTCGGAAGGGAAACAGGACCCGCAGTTGAGCATCAAAGAAGCTTTTGTTGCCGGGAGTGTTCCTTCTTTCGCACAATGCT

GCATTACTAGGGGATGTTCCGAGGAAGAAGGTCTTTGCCAAGGCTGGTTTTTGTCCTTGTGAACATTTTTTCTTTGATCTATAGCTTGTGAGTGTCTTGG

GGAGCTTTGTGGAGCTTTGTGGGGAGGATTGTAGTGAGTTAGAAAAGAAAGGGACGGCGGTAAGTGCTTCTGTTTTCCTGACAAAGAGTCGAGGACATGG

TGGGGACTAAAGTATCCGGCGCCGCGATGACGTTTTGTGAAGGAGGTGATGAACTTACGGGTTTCCAAAAGATGGCTGTGGTGGATCAGGGAGTGAAGCA

GCCGTTACCATCGCTCAATGTGGGCGGAGGAAATGTGCAAAATGGGCAATTGGGTGGGGACCCGGAAGCAGGCCATGGTGGTGGAGGTCATTATCATCAT

GAAGCCTCCAAGTCTGGGTGGTCAGTCCCGGTGTCCAGGGAGACGGTCCATCGCGTCGGACAAGGTCTCGAAACCCTTTCTCGCACTCTCGTGTGCTCGC

TGCTGTTTTAACAGAATTTCACTACTCCGTTTGCTGGCTCGTCCTGCCCAAAAAATTCGCATCTTTCCAAGAGTCTTCTCATCTATTCCGCTGGAAATCT

CGAATGCAGGAAAGTGATATATATGCATCAGAAAAAAAAAAAAACCCCCTCTTCAATGATGGATGGGTCTTTTGAGAGAGAGGCAGACAGAAACCTTTGA

CCACCCTTCCTTCCTAGGGTTTGAAGGTCTCAGTTCGGCTTTAGGGGGTTTCTGAAAGAAACTAGGTTGGTCTGTGTAATGATGATTTGCAAGAAATGCG

GTGTTGTTCATTTTTGCAGACAGCTGGTGGGAGGTGGGTTTCCATTTCATTGCGGCTCTCAACAATGCTTTCATACTAGGCTATCCAGCTCTCATCATGG

CATACCTGGGTTTTGCAACTGGCTCTCTCTGCCTCATCGGAGGTGGTGTGATCTCTTTCTACAACAACTGCTTACTGGGAAGTCTTCATGAGACTGGTGG

GAAGCGCCACATTCGATACCGTGACCTTGCAGGTCATATCTATGGTACACATCTCAGGCCATCATATCCCATGTGTATAGAGAGCTTGAGCTCAAGCTGA

CAAGCTATTATTGATGGCAAGATGCAAAAAAGCATCACTAGAGCCAGTTCCTGATTTTCTGTAAATCTGTAATGCGTGAGCTTCTTCAAGGCTCAAATTA

GTCATGGGATGACCTGTGTGTTTGTTGGCTGTGCATTTTCTAGGTCGTGGCATGTACAGAGCAACGTGGTTTGTGCAGTATTTCAACTTGAGTATTGCGA

ATGTGGGAACCATCATTCTGGCTGGTGAAGCTCTTAAGGTACTTACCTCCAGAACTATGAAAGCTCAATAACAGGGAAGGCAGTACTTCTGTGTTCTGCC

ACCACCTCATTGCCAAACAGAAATAGTTCACAAACAGATCCAGTGTAGTATGATGGAGTGATGATATGTTTTTGCAAGCAGATGACCAGGCATTCTTGGA

ACTAAAAGCAGAAACGATACCTTTGTCATCAAGGAGTGGATCCTTGATTGTATACTGTGAGCCTTATCAGTGTAGTACACTTTTTATGAAAATCCCATAC

AGATCTTTAAGCTGAAGCATGTTTTTAGAGTGATCTTCTGATTGCCAACATGCAAGCAGATAGATCGGTTAAGCCAGCATTTTGGGGAATTGTAACTGAT

GCCTTCTTTAGTGTTCTGATTCAGTTTTGACCATCAATTCAGGTTCATGGCATGTTCTATGTCAAGAAACTTGACCATTGTAGTGTTGAAGCTATGTACT

GAGTTGTAGCTTGGTGTGGGTGGGCAGGCAATATGGGGGGCATTCACGGATAACACCAGTGTCAAGCTGGCAGGCTGGATTGTGGTTGCTGGTGTCTGTT

TTGGCTTGTTTGCTTTTGTGGTTCCCAATTTACATGCACTGCGGTTCTTCTCAACCTGCTCACTGTTTCTGTCTCTCATCTACACCTGTATTGCCATAGG

TGTTGCATTCTCTGATGGTATGTCCTCTGTCTGTCTGTGTGTCTCTCTCTCTCCTGCTTGATGTACAGCAGTTTTTTGCTTTATTTGGTTTCTTATCTAA

ACTGGGCATGACATGTATTGACATGGCTGATACAACTCATCTGCTCCTGTCCAAGACTCCCTTTGAAGAGCGTACAGCATGAATCAGTGAAAGACTCATT

GTGCTTACCTGATATCATGGGGAACCTGTACAGGTCTGAAAGCTGGACCCAGGGACTACTCGTTGAAAGGCACAAAAGCTGATAGAACATTCAATGCAAT

TGGAGCTCTAGCTACAATAGCGTTTGCATACAATACTGGCATCCTACCTGAGATGCAGGTAGTCCCCCATCTTGATGATGCAATCTTTTTCTTACTTTGA

GCAGCATGGCAAGAGTATATGTCAATATGGAAATGTCTTTCTGTTTATTGTTGAAGGTATGGCAGGATCCTTACACAGGTTTGGCATTGCAGGCAACAAT

TAGACAGCCAACAACAACAAACATCTATAAGGCTCTTGGAATGCAGTTCACAGTTGGAACCTTCCCTTTCTTAGTTCTCACCTTTGTGGGTTACTGGGCT

TATGGAAACACAGCCAACCCTTACCTGTTGCTCTCTCTTGGTGGCCCAAAATCCTTGGTGACAGTTGCCAATGCTGCTGCCTTCTTGCAAGCTATTGTGT

CTCTCCATGTAAGTTCACATCCTTCACAAAGGCCCAAATTGTTTGTGTTTGCCTACATAGATGGCATACTGAGCACATGTTGTCTGTTTGCAAAGAACCT

GAATTTTTTAAAAACTGAAGAGTTGAGGGGTTTTATACAGATCTATGCAACGCCCATGTATGAGTTCATGGACACACACTTTGCCAGAAAGGATCAGGGT

GATTGGTCTGCTCACAGTATGCTTGTCCGCCTCATCACACGAGGCACATACATCACTATCAGCACATTCCTTGGAGCTTTGCTGCCATTCTTTGGAGACT

TCATCACCCTCACCGGTGCAATGGCAGCCTTCCCTCTTGAGTCTGGCATCATCCACCACATGTACCTCAAGGTCTCTTTTCTCTTCCTACAAAACCCAAT

ACACTCCCATACATTCTTTTCTCCCAAAGCAACACTTCAAAGTCTCTCACTATGTGCAACTACCATCTTCTACCATAGTGCAACTAATATTCCTCATTGC

TGGGATAAATCAGCTGCAAAGTGATGAGAGAACTTGCTATGGATCCAGATGGCCATGTCTAAGGACTGAGACTTCCAGTCGCTTCTGGAAATGCAAGAGA

AACTCAGGACACAGATTTTGGGTGGATTATTTTTATACTTATCTTCACTCACTGCTGGGAGCTGATGAAGTCAGTTGTAGTCATGGCAGCTTGTCATTGC

TGTCCATAATGAAGGTGACGGTCTGACACTTTGTGGGCAACTCTCATAAGGTTATGACTGCACTCATAGTCACATTACCTTAAATTATTGTTGTTGGGAT

CTATCTCTGCAATTTTCCAGTTATAGCAAACTGATAGAAATTTCAAATTACAAGCAGTCATGTCAGCCACTGTTGAAAGCAATCTCAATGATTGTTTGAC

CTGTAGAAGCTTGGTTTGGAGATATATGTCCAAAGCCCGAATCTCATAACCGATCAAGACGTTCTGATGCTTGAGTGATAATTTCTGTGGCTATGTAAAC

TTGCAGGTGAAGGGAAAGGGGTTCAGTACATGGAGGCTAACATGGCACTGGTGCATTGTAGTTTTGTCAGGAGTACTGACTGTAGCCACATGTGCAGCAG

CAGTCCGGTACATCATCTCTGATTCAATATACTATCATGCTTTTGCTGACTTGTAAAACTTGGCAGCAGTTTGCATATAATGCAAAGCAAGTGTGTATGT

ATGTAGCAGCTAGCATCATCAACTCACCCTTCCCAGGGGTGCATAATCAAATTTACAAGCAAGGTTTCAATTAGCTCCTTTTGCTACAGATTTAATACAC

AAATGTGCAGGGATATGATAGAAGCCCTATAGATGCACAACAGAAATTCAACAGGTGAAAGACAATTTCATTGATTCGATTACAAATTTGCTCCGATGCT

TCATAATAAAAGCCGGAGAAATAGTTTTTTTGTAAGTTCTGTGTCCTATGGTCTTGGGAACTCCATTTCATTTGTCCTTCAAAATGCATGTGGAGGTGTA

TCAGGTGATGACCATTGTGAGGAGGTCATCACCAGTATTGGATGGGTATCAACTTTTTGACCCAACACTGGTCTGGTCCTGGTATTGCGTATTGAAAAAA

AAATCCATACCAGGATGGTCCAAGGCTGATTTTTACCTGGTATGAGGCAGGAATAAACCTGGTATATAAGGCTAGTATTGCCTCAGGTCATCCCAGGGTG

GTAGGGTCCTTGATAGCAGGCACATGTCCTATAGACTCAGGTCATCCCAGTCAGGTAGGGTCCTTGATACCAGTGTTGGTAACAAAATTGATACCAGGCA

CAGGTCCTGTAGATGGGTACAACACTAGTCATCTACATGGACACTGCAAGTTATGTAGTTGCAGTACGGAATCTTGAGACTAAATAATTTTGAATTGGAC

AAGTTCTCAATGAAAAATGTTACAG

>Sphfalx0007s0128

ATGGCTTACATCCCGGGACCGCAGAATTTCTCTTTCCATGAAGAGAATGAGGGCTCTGAAGATATAGAAACTCCGAAGACAATCAAGACCCAACAACCGA

AAGAACAGTGCAACAACATAGGCGTCGATCATGCAGCTGGCGCTTCTTCTATCTTCAAGGTGCTCTAGAATAGCTGCAGCACAGAAGAAACATATTATGT

ACTTGTACAATTTCCTCTTGTTCATTGCAGAAAATATGTTTCGTGAAGCCTGCTGGATTCTAGCTTTTTTCTAGAGCGAGCTGTATCTCAGCTGCGTGTT

TTTCTTAATTCACGTTAAAGAACTGCTTGGTGTTCCTCCTGCAGGACATCTCAGTGCCCAAGAGGAGGAATGTGGAGGAATGGTTGCCAGTTACGAGCTC

GAGGAATGCCAAGTGGTGGTACTCGGCCTTCCATAACGTCACGGCCATGGTGGGTGCCGGTGTTTTGAGCTTGCCCAGCGCAATGGCATATCTCACGTGG

TGAGAGAGCAATCTATATCCACGTACTAGACTCCTCGCTCGCTCAGCTCCAATTGCTGAAGATTTCTGAACAAAAAAACAAAAAAAAACCATTTCCTAGT

CTCTTGTTGATCATCGATCAAGCTGCTGTATTCATGATGATCTTTTGCAAGTCTGATGATCGGTCTCTTGCGCTCATGAGCTGCTAATCCTTGCGAAGAT

TGCATGATGGGTCTGACCTCGTCGTGGGTGTTTGTTTTCTTGGTACTCCGAAGAACAGGGGGCCAGGGGTCGCAGTGTTGTTGTCGTCTTGGGTCATCAC

ACTCTTCACGCTTTGGCAGCTCGTGCAGATGCACGAAATGAAGGAATTTCCAGGCAAGCGCTTCGATCGCTACCACGAGCTAGGCCAACACGCTTTCGGC

AAGAAGCTGGGACTCTGGCTGGTTGTGCCACAACAGCTCATTGTGGAAATCGGCGTCGACATTGTGTACATGGTGACTGGTAAGAAAGAAAGAAAGAATC

ACACTCTCTCTCTCTCTCTCTCTCTCTCTCGCATTCATTGTTAATGCCCATCATCTCTTTGCCCTGCCATCTGCATTAAACCATAAACCCCCGGAACTGT

GTCAGGTGGAAGCTCGTTGATGGGGGCATACGAGCTTCTGTGTACAGGAGGACCGTCATCATGTCGTCCCATTCGAAAGACAGCATGGATTGCCATCTTC

GGATCTGTCCATTTCTTTCTTGCACAATGTCCAAACTTCAATGCGATCTCCCTTGTGTCTTTCTGTGCTGCAATCATGTCCCTCAGGTACCTTATCTCAA

CAGAGTCTCCTTTGTTTCTTGTATTTACAACTATGACATCTAGACTGTCAGCTTGATGATGCACCAGGCGCTTGCACATCTGTAAGCTTGATTATGCACA

TCTGTGAGCATCATTGCGTGCACATCTGTGAGCTTAATTATGCACAATGAGCTTGCACATCTGTGAGCTTGATGATGCTTTTGTAGTTTCCTCAGCAATT

GACACTCCTCAAATCTGCTGCCTGTGGGAGGTTGTTGGGTTGAAGAATCTTTGCTCATTGTGCAATGCCAATGGGTTTTTGCAGCTATTCAGCGATAGCT

TGGGTTGCACCATTGGCCTCAGGACAAGTTGCAGATGTTAGTTATGCATTACCAGATACCTCAAGGGCAGGTCTTGTCTTTGGCATTCTAAATGCACTGG

GACAAATTGCATTCGCATATGCAGGCCATAATGTGGTGCTTGAGATTCAAGCAACACTGCCATCGACACCAGAGAAACCTTCCAAGGGTCCAATGTGGCG

TGGCTGCTTGGTGGCATATGTAGTGGTTGCTGCTTGCTATTTTCCAGTAGCAATGGTGGGTTATTGGGCCATGGGCAATGGAGTGGGAGACAATGTGCTC

CTTTCTTTGGGCAAACCTGTGTGGCTCATTGCTGCTGCCAGGCTCATGGTGGTTGTTCATGTCATTGGAAGCTATCAGGTGATTCAAAGCTCCTCCTTCC

TGTGTGCTTTTCTTCTGCAGAAGATCAACATGTATGCTTTTGGCTGCAGCTATGGACTTCCTGAATTCAGCAAAAATTTACAGCTGCTCTTGAGCTCAGG

GATCTTGAAGATAGTGGTCACGCATAGTGATTGCTATGGATCCAACAAACAATTTCAGGACAAAGGTTTTCAGTTTATGGTTTGTTTCTGGGTACTGATC

TTGAGCTCTGTGATCTTGAGCTTGCTTTCTGGGTGCTGATCTTGAGCTCTGTCATCTTGGTTCTTGCTACTGAACAGGATCCTGCATTCAGGTAGCTACA

GAACAGGATTCAGGATTCTTGAATCATGGGGTTTTTAGGTTTTTCAGATTGTTTGTCCTGCTCTGCCTGTCTTGAGTTCCTGATGTGGTTTGGATGTTTT

GGTGGGGAAGGTAGGTGTATGCCATGCCTGTCTTTGACATGATGGAGACCTTCCTGGTGAAGAAGCTAGAGTGGAATCCCACCCGATTTCTCCGCCTGTG

GGTTCGATCACTCTATGTTGGTATGTGAGCTTCTACTTATACCAGTTTGTATTGATTAGAGAGAGAGATCTTGCAAGCATCTCTGCTATTTCCTTTCAAT

TGAAGGCTACCTCTGTATTGATATTCTTCTACATCAGTATGCCAGCAATCACATCTGGTTGTTATGATAGCAAGCAGATAGTAGCAACCAGAGATGCATG

TCAAAATGGGCTTCAGGGAGCCTGATAGAATCATAGGGCATCTTTGAAGATGTTCAGGTAAAATCTAGTTTGATGGGTTTGGCAGCTTTTACTATTTTCA

TGGCAATGACAATCCCTTTCTTTGGAGATCTCCTTGGATTTCTTGGTGGATTTGCTTTTGCACCAACAACCTTCTTTGTAAGTCTGGATCTCTCATCTTT

TTCTCGGCAATAATGGCACATACAGTAGTAGTGGTGCAGAAGAAAACTGGAGAGAATTTTGTGTGTACTTGTCTGTTGTTCCATAGAATGGTCATCAATT

TCTGTTTTGCTTACAGTGAACACATCAGTTTGCATCCAGATTTCACTTAATGCATTCTTGATGTTTTTGTGAGATGTTGCAGCTGCCATGCTGCATGTGG

CTCACGATCTACAAGCCCAAAGCTTTCAGCATGTCATGGATTCTTAATTGGGTACCCATTTAA

>Sphfalx0013s0130

CCACACACACACACACACACACACACATTTTCCTCCTCTTTCTTGCCCTTCTCCTACAAAGTCCAATTGGACTTTGTAGTGCAAGTGCAATTGCCGCGAG

AGAGTTTTGCTTCCTGGCCCAGACAGCAAACTTCCACTCAAGTATCGGAAGCAGCTAAGTAAGTAAGTACATGGCCGTGCTACAGCAGTACGCGGATTTG

GCAATCCGTCGGCAATTGTAATACTATTATAAGCTTTCGTTTCCCTCCCGGATCTATGTGCAGTTGTAGTAGTGATCCATCCTGACTGCCAACGTCCCAA

AGAGTTGTTGCAATCACAGACAGACAGACAGACAGACAGACAGTTTGCGGGAGTAGTAAAGATTTCAGCTTCTGAGTCTAGCAGCGTCGTGAACAAGTTT

CAAACAGAATGGCGACTGCGAAGGTATAATTTTTAAGAGCTCTCGAGAATTACTCTCTAGTGGGTGTCTCTCTCTTTAGAGTGTCTTGCAGGGGAGGAGG

AGCTCAGCGACGAGCTGTGTTTGTTGGTTTGATTGTGGGATTCCTACACTCTTCAAGGGTGGCTGAATCTTCTTCTTCTTGTTCTTTTTCTTCTTCTTGC

ATTCTTGCAGGAGGTCCCCGGGGGGAAATTGTGAGATGAATCTTTGATTGAGACTTAGGATCAAGACGATCGATCTTGTTACGGAGAATTTTTATTCTTT

GGGGGTTTCTAAATTTGATGTCGTTGTTGTTTTTGCTGCTGCTGCTGCTGCTGCTGCTACAGGATGTGGAGAACGTCGACAATGGGCATGCACCGGATTC

ATCTGCGGCGGAACAAAAGGCTTTGGAAGATTGGCTTCCCGTGACAGGGAACCGGAAGGCTAAATGGTGGTATGCAGCTTTCCACAATGTCACAGCTATG

GTGGGTGCCGGCGTTCTTGGTCTACCAACTGCCATGCAATGGCTGACCTGGTAAAAAAACACTCCTTCAAGAGCTCTTGCTGGGTCTGGAAGCCATCAAA

AAAAAAAAAACTCCTATCATGAAATTTTGTCTGAGATCAATTGTGCACCACCAGTTGCTTGCTGTTCTTGCTGTGATTTTTTTTTTCCCTCACAGACAGA

CAGACTTCAGACTTCAGGCTCGAGTCATAAGACTTGGTCATCTGAAACATGAGCATCATCATGAGGAAGCAGCTGAGAGAGAAGAGATTGACAAGTTGAG

TAATAATTTCTTGTGTGCATCATTGTGGTGGTGGTGGTGCCTTTTTTCTTTCTTTCTTTGCAGGGGTCCAGGAGTGGTAGTCCTAATTCTTTCCTGGGTG

ATTACACTTTACACTCTGTGGCAAATGGTGGAGATGCATGAGATAGTGCCCGGCAAACGCTTTGACAGGTATCATGAACTCGGCCAGGAAGCTTTCGGTG

AGAAATTGGGATTGTGGATTGTGGTACCACAACAGCTGATTGTGCAGGTGGGTGTTGACATCGTCTACATGGTGACAGGAGGCAGATCTTTGCAAACAAT

CTACAAATTGCTATGCAAGGGTCCTTGCACTCTTGAATTGCACATATCCCTCTGGATTTTTATCTTTGGCTCCGTGCACTTCTTCCTGTCCCAGCTGCCA

AATTTCAACTCTATTTCTGGCCTCTCCTTGGCTGCAGCTATCATGTCGCTCAGGTGAGTACCTACTATTCAGATGATGATGATACATGCATGCCCTGAGG

ATGCAGCTCTGACTCTTTCTTTGAATCTTGTTTGAAATCTCTTGGGTATTCCTTGTTTGTGCATGGTGGTTGTTGTTGTTGTTCCATGTCTGCTGCAATT

TTCTCTTTTTAGTAAGTGTAAGTGCAGTGGTGAAGTGTGTTGTGGTGGTCCATTTTTTGTGCTGCAGCTACTCAACAATTGCATGGGCTATCCCTGCTCG

TTATGGACACAGACTACCAGGAACAAACTCGGTGGTGTCTGCTGACTATCATCTGCCATACGGTCACAAGATAGGGGGAGATGTGATGAACATCTTCAAT

GCATTAGGAACCGTGGCATTTGCATATGCAGGACACAATGTGGTGCTGGAGATTCAAGCCACAATCCCATCCACTCCAGAAAAACCTTCCAAATCTGCAA

TGTGGCGAGGAGTGCTGGTGGCTTACATGGTGGTTGCTGCTTGCTATTTCCCAGTCTCTCTCGTTGGTTACTGGGCTTATGGCAACAATGCAACACTGCT

GGTTGATGGCAACATCCTTACATTCGAAGCCTTCCCAGTTTGGCTGGTTGTAGTGGCCAATTTTATGGTTATCGTCCATGTCATGGGAAGCTATCAGGTG

TAGTTGATGATCTCTCTGCCATCTGCATCACCTTCTTCTCCTCCTCCTGCATTAACTTGGCTTTTGACTGATGACAAGTCAGGAAGTCATTCACAGACCA

TGCATCTTGGGAGTTTCATTATTTCTGAAAGAGATTTCAGATAATGTGCATCTCATGGGGTTACATGATTCTGAAGAGATTTCATCAGACAACATGATGC

ATCATCTGGGTGTTTCATGATTCAGAAGAGATTTCAGACACCATGACGCATCATCTGGGTGCTGCATAATTCTCAAGAGATTTCAGATACCATGATGCAT

CATCTGGGTGTCTCATGGTTCAGAAGAGATTTCAGAGAACATGGTGCGACCTTGGTTGTTTCATAATTCTGAAGAGATTTGCTAAATTTAAGAAGCACAA

CTTTGTTTGTCAATTTTAGTCAACTTTCTGTTGTTTCCTCGAGCTGCAAAAGTTTACAAATTTGTTGTTCTTTCTCATATATATGCAGATCTATGCGATG

CCCGTGTTTGATACCCTGGAGACTCTGTTGGTCAGGAGGCTTCATTTCCCTCCATCCTTTCATCTTCGACTTGTCACTCGCTCTCTTTATGTCAGTAAGT

AGAGCTGAATCACTCGTCTTTTTTATGCCAACTTGCTTTGAATCAAGATGATTGTTGATGATGATGATGATGACGTAGTTAGAAATTTATTTTCTGCAAC

AAGTTTCAGATTCTTACTTACAGCTTGTTTCCAATTCAAAATATACTGCAATTTCCATTTTTTTAAGCAAAAAGTTTTTCTTTACTAATTTATTTTTTAT

TTTGGGATTATTCAATGGATTGCAAACCTTGGCAGTTTTCACAATGTTTGTTGGTATAACCTTCCCATTTTTCGGGGCTTTGCTTGGATTCTTTGGGGGA

TTTGCCTTTGCTCCAACAACATACTTTGTGAGTTTATGCTCCCATTATCCTCTTGGTTGCAACTTTTTAAAGTAAAAGTTCGAGCTTTCCTTTGTTTTTG

TTCACTTTATGAAATAAGCTAACAGTTTTGCTCCATTGACCAGTATCATGGTCTTATTATCAGCTCTTAATAAAGCACACTTAGTCATGGATTTGTGTCT

TGAACTTTTTCTTTTTCTAATGCATTTTTTTGTTGGGTTTTTGTTGCAGTTACCATGTATTATGTGGTTGTGTATCGTCAAGCCTAAAGCCTTCAGTCTC

TCATGGATCATAAACTGGGTGAGTTCCTTGTTCCGCATTCTCCTTGTAAAACTTCAACTGCTTTGTTTCTGTTCTGTTTCCTCCCGCAAGAAAAATAGGA

TCAAAAGAGTCCAAGTGTGGACTTTCACGGATCAATCATGTTCTACGTCTTTTACAAGAAAAGCCTTTGTTCTATTATGGAATACCCTTTCCTTCTTTAC

AAAAGGTTCACTACTACAGAATATGGCTTTTCGTAGGCATTGACACTTGAATGTGGCAAATGTCACATATTACACGAATGTCAAATTACGATAAAACACA

CCACATATATCTTATAGAGAAATGGATGAAATCGAAAACAGATGTGTCATGATCCATGGCTCTGTCATATACATGGCTTATTTAACATGGATATGTCTTC

TTGCAATTGATGTAAGGTGAATGTTATTATGGTGTGGTGATTCCAGCAAACATAGTATCCAAAACTGATTTTTATTGGCTTTGAGTTTTCCGCTGTGTAC

TGGGATCCATGTAGAAATGTCTTCACAGTTTTGTGATGGGCATGTGGTCCAAAAAATTAACTGTGATCTGAGGTTTTTTGGTATGTGAATTTCATGCAGG

TTTGCATCGTATTAGGAGTCCTACTCATGTTTGCAGCAACAATTGGTGGGATGGCGAATATTATTGTCTCGGCGTCAACATACAAGTTCTATGAGTAGAA

CTCAGTAAACAGGGTCAAAAGGCTTCCAATAACTGCAACTTGTGATAAACAAAAAAACTACCCAAAAGCAGAAAAAAGTGAGATGAAAAAAGCCTTTTGA

AAAATTGTTTTTACTTAAAGAATTGCAGGTCACTCATTTCTGTAATTGTTCTACTACAGACTTTGATAGATCTTTAGTAGTATAGATCCAACCTCCTCTA

TGACTAGAGAGGGAATGGCTTTCACTCTACCAGAAAACAACATTGGTTCTCCTTTCCAACAAGCCAAATCTATAGAAATACAAGGAGCCTGGCTTAAACA

CCCATAATTTTTCTTCCTGTTTAATTTGCGTCTTTGTAAAATTGTGGTATTGTGTAATTTCTCTTCCTTTAGTGACGGCAAAGCATGAACTGATTCAAAA

ATGGATAATATGTATTTTGCAATTGTTTGGGATTGACCC

>Sphfalx0014s0033

AGCTTGTCACGAACAGCTCACGTACGTCTCCTTCCGTTTCTTTCGCCGCAGCGCGTCTCTCCAACACCCCATCCCGTGCGCCTCTTCCAATTTCTCTTCC

AATTCTTCACAAATTTCCACCTTCCTCTTCCAAGTTCGTGGTCAATAATGCAACAGTAAAATGTTGTAAAATTTAAGTGCCAATTAAATAGAGGTACGTT

TTTGTCATTGCTGCTAACTTATTTACCGTCGACGACTTCGCCGGAGTCGACATCCAGCAACTGCTTATCTTCGGTCAAGTGTGCAGATGTCGAGAGCTGG

AGCAAGGTTGCGGTTTTGTTTCCTTCCTTACTTCCATTGCGCTCTAAATTTTTATTATGTCCATCTCTTTAATACTTTTGCACTAACGTTTATTCTTCCT

TATGTCGGCAGTGGAGAGTAATTTCCATAGTTGCCTGCAGGAAAATTTTCAGATACTGGTAGTATATTAGTGTATGATTCGGTTTGTGCATAGTCATGAA

ATTGCGGCTTCAAGAAATTGATTCAGGCAGCTCAAGAAACATTTTGAACACTTGGGTTTTTGAGCTCTCACGTATGTTTGTTATTGCTCATCGCTTTCTG

ACTGACTGACGACTACCAGACTTATAAGTCGTGTTACCTCATGTTCCTGACTGCTTCAGATCGGCAATGGAGGAACCAGCGCCCGAGAAGCTGAACAGTA

CACTAGAGGAAGCTCATGAGGGATCTGTACTCGATCATTCCAATACAGGCACCACTTCAAAACAGACTGCTTGGGTGACAACAGGCAGAGGGACATGGAG

ACACGCCGGCTTTCATTTGGCCACCACCATTGCTACGCCCGCGGCGTTTGCACCTCTACCCTCTGCCGTTGCCGCCTTGGGCTGGCCTGCTGGTGCGTGA

CCCCATGCTAGTAACCTCCTAGAGTGCTAGATTATCTGAAGCACAGTCCTCATGAGGTGCCAGGACCTTCCAATTATTACCTGGCGGGCAAGAACTACGA

ACCTCGAACTTCGAAAGTCTATGACATTTTTTTTGAGAGCCATAGCAATGGATATATCAGCTGGTGCAAAGAACGTAGAGACCTTGAGATATATATAGTA

GCGTAGCTTCCGGAAATCAGCCTGCAGATCATCTGGGATTTGTAGCCGAGTACATTAATCCACAACCCTGGAGAAGTTTGCCGAAGGATCTCACCCCCTT

TTCTGTTGTTGTCCTTAGCCATTCCCTTATGGCTTTTGCTTTCTTGGGTTGGGCTTCACTTCCTCCCAGGACAGCACATGTCCCAAAAAGTCCATCTCTA

ACCGTGCAAACTCAGTATTTGCCTGGTTCTTGTAAAGCATATTTCCTAGAAATCAAATTTCACTGAATATTGACGAAGGCAAGGGAATTGAAGTGCAGGA

GTAATCAGCTTGGTGATGGGCACATTGGTGACAAGCTATTGCAGTCTGTTGCTGGCCAATCTGTGGGACTACAATGAACCCAACCGCTATGTTCGCTACC

GTGACCTTGGTCGCGCCATTTATGGTGCAGTACAAGCTCTGACATCACATCTCTAGTACTGTACATAAAATTTCTCCTATCATATGCATCATTGTGCTCA

GGAATCCTCCATCGCCCCCAAAACTGCCCTCAGCAAGTGGAAAACCTTCAGTGTCAGATCTGTGTGAATGCAGGTCGCTGCACTCACTGCAGGGGGCAAG

TTTTGTAATATGCTGTGTGATTCTGTGAAGAGGGGGAAGCTTTCGGTTGCTATGTTGACCACAGTCTAGTGATGTTCTGATCCATGTCTCTTGAGTTCTA

GTCAACATCATGACTGAGTCTTGGGCCCCCTTGCTTCCAGAAAATGTGACAGAAATAATTACTCTGGATCATGCCAATTTTGTACAATGTTTTGCCCAGA

TTTTGGGTGGATTATTGAAAATCTATACCCAGCGGCACTGGGACTTGGGAGCTGATGAAGTCAGTGGGTACAGACCTCATGGCAGCTTGTCACTGCTATT

CATGATGAAAAAGGTGATGGTTTGACTCTTTGTGAGTGACTCTCACAAGGTTATGACCGCACTTGTTGTGTCACATTAATTTCAAAAACTTGTTGTGAAG

ATTTTTCTGCAACTTCATAGATGTATGTTTGAAGTTGTTGTGCTCACAGTCAATGTTGTCTAATTTGAAACATGGTTCCAAACTTGCAAGAGCTTCTTAT

CCCTGAAGATTCTTTTTTTTTTTCCTTCGAACGAGATATTATAACAACTGTGAACAATTTTACAGTGATGTGATGGTTTAAATGGATTGAGCAGGAAGGA

CGGGGTATTGGTCTGTCACTGTATTTCAGCAAATAGCAGCCATTGGCAACAACATCACTATTCAAATTGTTGCAGGCTTGAGCATGCAGGTCACACCCCA

CCAAAAAAACATCTCAATTGCCCATTGCTCAATGCAGTACTTGGCTTTCAGAATTACAATTTTACAAATTTTATGCAACAAAGTGATGATCACTATGAAA

CCTTCAAAACCAATACCATAGAGCTTTCGTAGCAAGATCATCACTTGGAATTTTTAGTGCTCTAAGTGCCCACAAGACAAATCTACAGTAATCCTGCAAG

AAGATAAAACTCTAATTCTTTGTCCACATCTCGATTTTTGGTGCAACCTCAATCTGACACAATGCCCCCCTCTCTGTTTCTGCAACTAAAGATAGGACTG

TTTTCTTCTTTTAAAGATGACACTCTTTTGGTGGACATTTTATGTGTATAGCGACCTGATTTGTTGGGTGCACATGATGCAGGCAATCTTTGTGACATAT

AATACCTCAGACCCATCAAGGGTAACACTACAGGAGTTTATCATCATCTTTGGGGCAGCACAACTTGTACTGTCACAACTACCTGACATCCACTCATTGC

GGTGGTTCAATGCCCTCTGCACATTCTGCACAGTGGCCTTCACCATTGTTGTTATGGGTCTCTTGATTCATGCAGGTATCACCTGTTCACCCTCTCTACA

TTTGACGTCAAGTTTGTGACTCTTTGGTCTGAGTGTGTCCCTTTGGCACTCATCATGTTTCAGTCCCCATGATGAAGGTTGGGAAGTTGATGATTAGCCC

ACAATATGTACCAAAAGGTGTACTTTCAAAATTGCTGGCTTCTAAGCATATGTGCATTGCCAATTGTGAGTGCAGAAGTTAATAACCTCAGGATGTGATC

TTGCTTTCAAGCAAATGTTTTAAAATGGTAGGTTTTACAACTGCACAATTGGATCTGAATGTTCGAGTCACCAACAGTAGTGAAGTCAAACTGATCATCA

GCCTCTTTGAAATCTTGGGATTCTATTGTGGTATGATAATTTGTGGGCGGTGTACAGGGCAGAATAGAGATGGGCCTACAGACTATGGAGTGCATGGAAC

ACCAAGTAATAAAGTGTTTGGGATTTTCCTTGCACTAGGCACAATAGCTTTCTCCTTTGGTGATGCAATGCTTCCAGAAATACAGGTACAGAAAATGCCG

ATCTCAATGCTTAAAGATCTGAGTTACCTTCGAAACTGAAGAGTGTCTTTCTTTTGTGTTATCAATTATGGTTGTGAATTTCAGAGTTCAGAATTTAGAA

AAAAGTTGGATTATCTTTGACACATACTGTAATGCCACAGGCAACAATCCGGGAGCCAGCAAAGAAGAATATGTACAAAGGAATATGCTTGGCATACTCA

GTGATCACAACCACATACTGGCTAGTTGCATTTCTGGGATACTGGGCTTTTGGCTTTGCTGTACAAGCATATGTTGTCAACTCCTTCAGTGGTCCCAACT

GGGCCATCACTATGGCCAATGTCTTTGCTGTTATTCAGGTGGCAGGTTGCTTTCAGGTGTGCTATAAGCCTATAACACATTATCACATTCCCAATAAATC

AACATCTCTTGTGCATGGTTTTCTTTCAGTGCTAGCCAACATCATAACTGAATCTTCTCCACCCCCCCCCCCCCAATTGCTTCTGGAAATGCAACAGAAA

AACTAAGGAAACACATTTTCAGCATATTGTTGTATATTTATCTACACCCAAAAATTTATCAGGAGGTGATGAAGTCAGTTGGTAGTTAGTCATCATGGCA

GTTCATCCCTGCTATTTGCGACACAAGATGACAGTGATCTGACACTTTGTGAGCGACTCTCATGAGGTTATGATTGCACTCATGTTACTTTCAAAATTGT

TGTTGCAGAGAATTTTCTGCAGTTTCATAGATATGTTCTGATGATCTCTTTTGCCCACCCCAACAACAGAACATAATTATTTGAGGAGGATGCTGTTGAG

GAAATTAGTAGTGAAGAATTCAATAACAGCCAAATTGACAACAAGTTTAGGATTTCATGAAAGTCCTAGAACATTTATCATGCATACATTTTGCTTGGAT

CTTGATTGTTACATTTATGAATCTCATTTCGTTTCACAGATCTACTGTCGACCAACTTACCAGTATTTCGAGTTCCAGCTCATGAACCCAAAGCAACACC

GGTGGTCACTTTACAACAGCTTGGCCCGGCTACTGGTAACCTCAATATACACAGCACTGGTCACCCTAATTGCTGCTGCCATGCCATTTTTTGGTGACTT

TGTGGCACTCTGTGGAGCTATTGGGTTCACACCTCTTGACTTCATTTTCCCAATACTTGCGTTCCTCCGAGTCAAGAAGCCCAAAAGTCGAATTTTCTGG

GCATTCAACATCGGGATCGTAGTCGTGTACACATTGGTAGCCATTTTGGGTGCAATTGGTTCCATCCGGTATATTGTCAAGGATACAGTACGATATCACT

TCTTCCAGAATCAGTGATTTTCAATGTTTTTGAAAAGGAAAGGAAACTGCAAGTCTATCCAAAAGAAAAATTAAAGATGCAAGTTGAATGTGTTGTACCT

GCATTCTTATGAAGAAGCCTGCGCTTTCACTCATTTGTATAGTAATACTTGTAACCATTTTGTTATTGTCCAATTCAAGAGATGTTATCTTGGCAATGGC

TGCCTTTAAAATGTTCATATTTTATGTAAGCATTATATCGGCTCCAATAGCTA

>Sphfalx0015s0259

GGACCGTCTGGACAGCATCAGCACATGTGATAACAGCAGTGATAGGATCAGGGGTGTTGTCTCTGGCCTGGAGTGTGGCACAGATGGGCTGGGTAGTGGG

TCCCATTGTGCTGCTCCTCTTTGCCCTGGTCACATACTACACCGCCCTGCTGCTCACAGATTGCTACAGAAGCCCTGATCCTGTCTCTGGCAAGAGAAAC

TACACCTACATGGATGCTGTGAAAGCAAACTTGGGTAATTATCACATCAAGCACTTCTCATTCAACCACTACAGATAAATTAGGTTTGAAGAAGAAGAAG

AAGATGCTAGTCGAAGCTCTGTCCTGCTCAGAACAAAGAGCATCTTCCCCAGAACTATGATGTGCAATCAGATCAGCAAATTGTTGAATCGAACTGCACA

GATAAGAAGAGGTCTCAAAAAGTTTATAGCCTTGAAATAAGCAGTCAAGAAAGATTTCTTACTATTCCATTTGTTCTTGGAAAGCCCCATGATTGAAAGA

GGAAGATGTCAAGGATTATGTTGTATGCTCCCAGTTGTTTTATTTGCTTTATGTATATGTGTAAAGTACTTTGTGAGTTTCTCTATTGTGTGTTGGTGCA

GGACCAAAGCAAGTGGTTATCTGTGGGCTAGTGCAGTACACCAATCTGTTGGGGACTGCAATTGGATATACCATCACTGCTACAACAAGCATGGTGTAAT

GCCCCTTCTTTCATTTTGTCTTTCTGTTTGTAATTTCAGGATTTGTTGGCTTCACCATGCAGTAGTTGTCAACAGGGCTATGTAGTTCTTTACAAAAGTC

ACAAGCAGTCCATGTGATGTCTCGCAACATTCCAGGAAAATCACAGATGCCAAACTATTGTGTAATCATTTGAAGAAGACCCTTCAATGATTTCTTTGCG

GCACTTTCCTGAGCAAGAATATGGACTCATCTTTAAGATAGGCTGTCATGATGCATTTTGCAAGGAAGTTGAAACTGCCACTATCCCTCAGTTGTTGGGG

GTCATCAGCAAAGATTTAAATTGTAGTACTGATTTTAAAAATATCTCAGTCAAATATCTTTTATGTACACTCTAGAGAAAAAAATTGATACAAGAAAAAG

TTATATGCACTGATAGGAACAGGAATACCTTCTCTGCATATTTTCTTTTCTGAAGATGGACTTGCAGGGCTGTATTTTATGTATTCCACTGTTCCTCAAG

AAATTCACACCGCTTTTTTGCTTGTGTGTACAGAGCTATAAAGAGATCCGACTGCTTTCATGCAGATGGAGATAGTGCACCATGTCATGTATCCAACATC

ATATACATGGTTTTCTTTGGCATAGTACAGGTCATTTTGTCTCAGATCCCTGACTTTGATCGAATCTGGTGGCTCTCAATAGTTGCTGCAATTATGTCAT

TCTCATATTCAACCATTGGCCTTGGCCTTGGTTTAGGGAAAGCTTCAGGTATGGAGACACACCCATCACCACAATTACATATATATATATATATATATAT

GGATCCATTTGTGTCCCATGTTTCTTCTTTGATGTGGTTTGTCAGCTGTGTTTGAATGTCTTCATTGAGATGCCGTTGTGTCTGCCTATCTCTTCACCTG

TAGAAATAGAGAATTCAAGACATTTGACTACTGGCATGCATTTAGAACATACGGTTGTCGGACATTAACACAATTTACGTTGCAGAAGGAGATCATCCTC

ATGGCACTTTGACTGGGGTAGAAATTGGTGACCCCAGTATTGGCTTTGCAACAAAAGCTCAAAAGATCTGGGATGTTTGCAATGCACTTGGCAACATAGC

ATTTGCATACTCATTCTCCATGATCCTTATTGAAATCCAGGTATGGTGCAGAATTCTCCCAAAACCACTTTATTTGTCCTTTTTCAGACCACATAGATAT

ACACTTTTTAGGTCTTCATTTCTATTGTGAAAAGATAACAACAGATCTGTTTCAAGTGTTGAGCAGGACACATTGAAAGCTCCACCTGCTGAGAACAAAA

CTATGAAGAGAGCAACTCTTATTGGGATACTCACCACCACCATATTCTACATGTCTGTGGGCTGTGTGGGCTATGCTGCCTTTGGGGATGGGGCCCCTGG

CAATCTCCTTACAGGCTTTGGATTCTACAATCCATATTGGCTTGTTGACTTTGCCAACGCTTGCATTGTTGTCCATCTTGTTGGAGCTTATCAGGTACAC

GCACTTTGTCTCTCAATGTTTCTCATCAAGTTTATTGACTTTTTCTTTTGCTGCAGCACTCTAAATTTAGTTCTATCACAAAAACACAATAATTTGTCAT

CTAACACTACATTTGCAACTCAGTTTAGCAATGTGGGGGTATTGTACAGGTCTACACCCAACCACTGTTTGCATTCGTCGAACACACAATGTCCCGCAAG

TTCCCCAAGAGTGACTTCATCCACAAAGACCTTGAGATGAAGCTCCCATGGGGAGCACCTTTACATATCAACCTGTTCCGTCTTGTCTGGCGCACGGCAT

TTGTAGCATTCACCACAGTTGTGTCCCTTGTGATTCCCTTCTTCAACGACGTACTAGGGCTAATTGGAGCTTTCTCATTTTGGCCCCTCACAGTCTACTT

CCCAATTCAAATGTACACAGTGCAACAGAGCATACACAAATGGAGCTCAACATGGCTAGCACTCCATACCTTGAGTGTCGTGTGCTTCTTTGTATCATTA

GCTGCAGCAGTGGGATCTATTGCTGGAATTCTCACCGATCTCAAACATTACACTCCATTTAAATCCTAGATCAGACTCAACTGAAGCGTTGGGGATGGGA

ATCCATAATTCTCCACAGTTTTTCGCTGCTACTACTTTATGGAAAGAAGGACGGTGCCATGATATATTATTTATTGGAGGCCTCTTTTGGGTCGGTCACA

AAAGGCCTGTTTCTAAGACCTGTGTCTAAATTTCATACATATAAACAGAAATAGACATGAGAGAGTAACTTTTGGTACTGATCTAAAATGCACAACTCCT

GGTCACCTATAATTTACCTACAACACAAAGAAAACAGCTACGGATTGTATCTTGACTATCAAGAATGAACTAAAAATTCGTACTCTTGAACTCCCAATCT

TCTTGTATTGTTGAACATTTCAGG

>Sphfalx0018s0093

GGAGTCCCTGGACGCAGCAGCAGCAGCTACTAGTAATAGTAACTGTGTGCAGCAGCAGGTGGCTATAGAAGCAGCTGCTTTGCAGAGCTCTCATGATTAG

ATAACTGGTGGCTCGACATGAGTACTAGTTAGTAGTTCTGGTGTCATGGATTAGCAAGAATAGCAGCACCAGCAGCAATAGTCAAGGTTGTAGAATCTGA

GATTTCAAGAGTTTCGGCAAATTTGCAGCATGCAGGGTACTCACCAGTGTTGTCCTGCAGTTTTGGAGGATAGATTGTAGTTCTAGATTCTAGTGTTCAT

GATATTGTGTATGAGCAGCTTGTGGTACTTGGGTTTCCAGCTGCACATCTCCTCACAGCTTTGATCCCCCGCCGGCATTTCGCCGTACATTTGAAATAGT

TGTAGCAGAAGAATTGCGACAAAAGCAGCTGCTGCTGCATGAGGTCAGTGATCATTCGTAATGTTTGCTGATCTCGACTCGAGCTGGCTAACAAGCTAGC

TGGATCCGAATATTGTAGCAGCAGCTGGGGAGCTGCAGCAGGTGATCAAGGACTTAGGAGACCCCGGCCGGAAGGCGCAGAACAGGCCGAGATTAAGATC

CCTGGCGCTGTGGTCGTGGTGCGAGTTTTGCAGCATCAGCAGCTTCTCTGCAGGAATCCTCTCGACATCTTGAAGCTGCGATGATCAAGTTTTGTTTTGG

TTGAAGGTGTGGTTGGAGGTAGAATCTGAAGCAAGCTTGTGAGACTAGGTAGCAGCAGCCATCTAGCTTATAGTGGCCACTGCTGCAGAAGATCGATTCT

AGCTAGTGAACCGAGGTCTGAAGGTTTGGAGTGAGATCAGTTGAGGAGCAGCAAGCAAACAAGCAAGCAAGCAAGCAAGCAACCTGGGCTGAGCAGGCGC

AGCTCCCCTTTTGTTTGTTGGTTTCTTCTTTCGTCGTCGGGGAAAAGCTGGCCTTGTTGGGGCTGCGATGACGTTTAAGGAAGGATTGGAGCTCATGGTC

GACCCAAACATGAATGCCGCGGCTCCTCAAGGGGGACTATTGTCGAAGTCGACGTCGTTGCATCAGCAGCAGCCATCGCGGCTTTCATGCAATGTGCAGC

AAGGAGCTGGTGGAGTCGATCTTCTTCTTCATGAGCATCAAGATGTGCAATTGGGCCCCGGGGGGGATCCGGAAGCAGCAGCAGGAGGTACTACTGGTAC

TCCGGGCTCCAGTACTACCGGCAGCTCCGGCAAGTCCGTCCGCCCACTGCTGGTGTCCAGAGACACCGTCCATCGCGTGGGTCAAGGTACAAGCTCTCTG

GAATCTGAATCTGAATCTGAATCTTTTCAACTCTTTGGGCAGCTTTAGTTCAGCCTCAGTCTCGGAGGTCTCAGAATCTTAACATTGCATAAAAACCAAC

TCCATTTGTAATTTTCTTAATTCTCCGACCTCTCAGAGTCTTAACATTGTTGTTGTGTCGAGTGCAGACTGGAATTCTTGTTTCTTTCTTCATCATGGGT

TTGGTTGCAGCAGCAGTCTGAGAATGAATGATCTGAACTACCACTAGTTAGACTGTATAATGATGATGATGATGAAGATCTTGTTCTTCTTTGAAAATTT

TATGTGAAGGTTTTGTAATTGTGCTGTGCAGACAGTTGGTGGGAGGTGGGTTTCCATTTCATTGCAGCACTGGACAATGCCTTCATCCTGGGCTATCCAG

CTCTCATCATGGCCTATCTGGGCTGGGCAACTGGCACCATCTGCCTCATTGGAGGTGGCATCGTCTCGTTCTACAACAACTGCTTGTTGGGCAGTCTTCA

TGAGACTGGTGGAAAGCGCCACATTCGATACCGCGACCTTGCTGGTCACATCTATGGTAACCTAAAAAAAAATTCAACCGAAGTGCTTTTTCTCCTCCTC

CTTCAAGGCCAACCCGGCCCACAGCCGAATCCCCATAGAAAGCTGCTTAATCCTTGTGTCTCAGAGCTTCATGGGTGTAGTTTTGCAAACATGTACCAAA

GGAATTGAAACCATTGCTTGATTTTTTGTATCCAACATTTTAAAGGAAAGAAAAGAACTACAAATAATGTTTCCTCATGCAGTAGTACATTGTTGATGGA

AATATTACTGCAATAAAACAAAAGGTTCAACTAGAATCAGGTCCTCCTTCTTTTAGCCTTGCTTGTGAAGAAGGAAGGGGGGCCTTGAAATCAAGAGTTC

TAGTGTTAAACTGAGGAGGAATTGCATGATCTCTGATGAAGCTGAGTGCCTGCCTGTGTTTTTTTTGGGCTGTGCTTTTTTCAAGGTCGCGGCATGTACA

GAGCAACATGGTTTGTGCAGTACTTCAACTTGAGCATTGCAAATATTGGAACCATCATTCTGGCTGGTCAAGCCCTTAAGGTATGTAGTATGTACAACAC

AACAATTGTTTCAATCTTGCTTCTTTGGAGAACTTTTCTAGACTTATGATGCAGAACATCATGTATGTACAACACAAGAATTGCTTCAATCTTCCTTCCT

TAGAGAACTTGTCTAGACCTATGATGCAAGACATCATGGCCTCAGAGCTATTTCTGGTGTGGAGCATCATCAAATTTCAGTTTACATGTGAAGAAATTAG

TTGTGACAATTTGAGCATTTTTCTTTTCTGTTGTTGGAGCACAGTCTCTTGGACCTTGAGGAAAGAAAGCTAGCTAGCTACATGTATGGGTACCTGCTAG

ATTTGAGGAGAACTTTATGGGTGTGGCATTGGTGCATGTTGCAATTTTTTTGCAGGCAATTTTTGGGGCTTTCAGTGATAACACCAGTGTCAAGCTGGCG

GGCTGGATTGTGATTGCGGGTGTCTGTTTCGGCTTATTTGCCTTTGTGGTTCCCACTTTACATGCGCTGCGATTCTTCTCCAGTTGCTCGCTGTTTCTGT

CCCTCATCTACACCTGCATAGCCATTGGTGTTTCCTTCTCTGATGGTAATATTCTCTTTCTGCCTCTCTCTCTCTCTCTCAGAGTCTCTGAGCCTGCTTC

ATGCACAGTTGACAGGGTTTTTTTGCTTCAGTGTTTGAGGTTTCTCAGTTCAATGGGCTAGTTAGTTAGTCCCAAGACTTGCTCTAATATTACAATTACA

AGAACCTCCTCATCTGACATATGCCATTAAACCCATGATCCAAAACTGCTTTGACAGTTCTAGAGAACAACATTACTGGTAATCGAGTCTAGCTCAAGAG

CTGCAGTGCAATTCAAGGATTCTTCTTTGTTCGTTCATTCGTGCTTGTAACCCAAAAACTTATTGTGGGAACCTGGCTGGTGGTGAAACAGGTCTCAAAA

GTCCACCACGGGACTACTCTTTGAAAGGGACAAGAGCTGATCGAACTTTCAATGCAATTGGAGCTCTAGCAACAATCGCATTTGCATACAACACTGGCAT

CCTCCCTGAGATGCAGGTACTCACTAGTACATCATCCTTGATTCAGATGCAATTCTAGTCATTTGTTTTACATGGGAAAAATGTACCATTGTTGTTGTGG

AAAGACAACATTTGTCTTCTCTGATTTTCTTTTTTTTTTCCTGTTATTTCGCTCCTTGTTCCAGGGTTGAAGAATGTACTATTTTGCATTATGGCAGGAT

TGAACTTCAGGGTTTTTAGTTAACACAATTTAGTGGCAATTGCACAGGCAACAATCAGGCAGCCGACAACTACAAACATCTACAAAGCTCTTGGAATGCA

ATTCACATTAGGGACTTTCCCTTTCTTATTGCTCACCTTTGTCGGCTACTGGGCTTATGGAAATACAGCCAGTATTTACTTATTGTCGTCTCTTGGTGGT

CCAAAGTCGTTGGTCACCATTGCCAATGCTGCTGCCTTCTTGCAAGCTATTGTCTCTCTCCATGTAAGTCACAATCCTGGAGACTTCACTGGTGCCCATT

GAACTGCACTGCTTCTTGTGTATAGACTTTGTTATAGTACCTGCACGATTGCACACATATATACACCCAGGGACAAATCTTTGTCACTGCCTTGCAGAGT

TCTTCAACTGGAGTAAAATTTCTTAGACTGAATGGAGAGTGAGTCACAAAAAGATTTATTTGTGTGCACAGGTCTATGCAACACCCATGTATGAGTTCAT

GGACACACATTTTGCCAGGAAGGATCGGAGTGAGTGGTCTGTGCACAGCATGCTTGTCCGCTTCATCACCCGAGGCACCTACATCACTATCAGTACATTC

CTTGGAGCTTTGCTTCCTTTCTTTGGGGACTTCATCACTCTCACTGGTGCCATGGCTGCCTTCCCTCTCGAATCTGGCCTTATCCACCACATGTACCTCA

AGGTCTTCTTTTCCTCAACCAAAACTCTAACTCAGTCTGATTTCCATCCCATCCATCTCTTCTCATCATGATAGCCTAGCTTATCATATTTCCAAAAGTG

TACTAGTGTAAGGACTGAGAATGGGCCCATTCTGCAAATGACTTGGGCCCATTGCAAGGTGGGAAACTTAGCGTAGGACCCAAATTCCCAGAACTGGCCA

TGGTCCTTACCTGGCATAAAGAAACAGTGGCATTTTGCTTTTAAAACTGACACCTGTGATTTTATTTGAGGAGAAGAGGTACCTTACACTTAAGGTTCCT

CTTCCTCTTACATGTAAGGTGTCATCCTCTATTGAGGCAGAAATTTTACTGAGGGTAAGGCGCCTGCCCTCGACAGAATCTAGAAATTTAATTCTACCAT

GTCTTTATGCCCATGTAAGGACTTGAGATGGGGGTGATGGTATCGGGCTGACAGGGACTCTGGAATTTGCAGAAGTCTCCTCAGTAAATTCTGAATGTTC

TAAGCCATTTCTGAAACCAATCTCAAAAACATTGTTCAGCTGAAAGTAGAAGTTTGGTTTCCAGGATCCCACTTTTGTGACAGACCAAATTAAGATTGTC

TTGATGCTTGAGTGGGCTGATGATGGGTTCTTTTGTGGGGATGTGAAATTAAATTTTTTTTGCAGGTGAAGGGGAAGGGATTCAGCAAATGGAGACTACT

ATGGCACTGGTTTATTGTAGTTATTTCAGCAATTCTAACTGTAGTCACATGTGTAGCAGCAGTTCGGTACATCATCCAAGATTCAACAAACTACCATGCT

TTTGCTAACTTGTAAAAATTCGGCAGCTGTTTGCACAATGCAAAAGCAAAGCCAAGCCAAGTCAAGCCAAGCCAAGCCAAGCAAAGCTTAGCTTGTATGT

ATGTAGCAGCGAGTAGTATCAGCAGCATAGGTAGCCTCCCCACGACGGAGGGTACATAAAAACACCCCCAAAAAACAAAAAGTTTACAAAAGTCTCACTT

TAGCCCACTGTTTTTGCTTCTTCAGATTTTATACACAATGAATGTGCTGGGATTTGATAGAACCCCAATAGATGCACTAGGCTAACACAACAAAATCAAC

AGGTGAAAAGACATTTTGATTGATTTCAATTACCAACTTGCTCCAATGCTTCATAATAAA

>Sphfalx0025s0047

GCTCACTTCGTTGCAACAACCCTCTCGGACGGTGTGTCTCTGGGCTGCATTCTGAGGTAGCACATTTGTGGTCAGTCAGGATTACTCAGCCCTGGACCTG

CATCAGTTGGTCTCATCAGCTTGATGCCAAAAACAAACGAGTTTCTAAGTTGTAGAATCTTGTACACTAGTAGAGTCGAGATCGACATTTGTTTGGTCAT

GTAATTCGAACTTGGAGATGTTTGAGAATCGCGAGAAGAAAACATCAGTTTGAGTTTTGAGGTGGTGGAAATTGTTTTGGGTCTATTGGAAAGGTGTAGT

TTTGATATGGTTGGAGAGCAAAGCTGTATTCTATAATATGATATAGTTGATTTGTCTAGTTGATTCTTGAGCATCATTAACATGAAGCTCAGATTTTTCT

TTTGTTTTCAGAGATATTATGAGAGGAAACTAGATGAAGTTAGAAGAATTTTCTTTTGCGTTTATAATGGTCAGAAGCTTCTTCAAAAGATGATTTTGAT

GTAAGCGGGAAAAGAACTAGAAGAAAGAGTATCGAATTTCAAGGCTGATATAAAGAATTCATCATCAAAATTGGAGCTTGAAGTCACCAAATTTGAAGAG

GAGAGAGAGAGAGAGAGAGCTTTGCATGGTAACGGATTAGACACGGGGTTTGACGTACTGGAGTGAGTGAGTTTTCCAAATGATTTTAGAGCTTGGGATG

ATGGTCTTCAAATCCACAGGATTGATGTTCGTTTTTTTGGAGAAATTTCTTCAAGATTGAGAATTTGGGAAACTTCGTCCCTGAGCTGCATCATCGTCGT

CATGAATATGGAGGGAAAAGATCATGCTAGGGACTTCAAGATGCAAGAAGCGAATGGTTTGACAAACGGGTTCAACTCACTGGATGTGGTGTCACCATCA

CCATCACTCTCACAAGAGGAGTATCGACATTCAAGCTTGAGCGATGCTCGCAAAGATGTGGGGACTCTGAAGGTTCTTGAATCAAAAGGTGCTCGCACAA

CTGAAGAGGACATCCTTATGAACGAAAAGAAAACACATTGTCATTTTGCTGTACATAACTCTAAACTGGAGAATGTGATCAGATGAAGATGGAGATGTGT

TTTACAGGTCTAAGCTTCGCTTGAAATTTTCTATTCCCACATCCGGAGTTCTCGAATCTGACCTCAGATTTCTGAAGAGAATGTTTGAAACTTCAAAATT

AAAGCTCAATTTCTTGGTCGATGTCTTTCTTTCTTTCTTTCTTTGTTTTTTCTTCTTCCTTTCATGGGCCATCATCAAAACTGCTGAGCCTGCTGTGCAC
[truncated: 597,373 more chars]
