# Supplementary material for: Evolutionary significance of amino acid permease transporters in 17 plants from Chlorophyta to Angiospermae
Source: BMC Genomics. 2020 Jun 5;21:391. doi: 10.1186/s12864-020-6729-3 (PMC7275304; doi:10.1186/s12864-020-6729-3)
Supplement: Supplementary file 8 — Additional file 8. The information of GO annotation for each AAP members. [file 12864_2020_6729_MOESM8_ESM.docx]

Molecular Function (MF)

GO #Seqs

transmembrane transporter activity 103 Sphfalx0084s0062, Pp3c23_12700, 127260, Sphfalx0084s0063, Pp3c3_11320, Sphfalx0002s0399, Sphfalx0362s0007, Pp3c8_19000, Sphfalx0362s0005, Pp3c14_9480, Sphfalx0104s0057, Sphfalx0007s0128, 156907, Sphfalx0616s0001, Sphfalx0040s0186, Sphfalx0005s0083, Pp3c13_12390, Sphfalx0007s0031, Sphfalx0005s0085, Sphfalx0003s0314, Mapoly0032s0115, Mapoly0032s0116, 75458, Sphfalx0025s0047, 31400, Mapoly0047s0067, Sphfalx0013s0130, PpAAP9B, ZmAAAP17, ZmAAAP18, ZmAAAP59, ZmAAAP14, ZmAAAP55, ZmAAAP56, ZmAAAP54, ZmAAAP60, pa_MA_889393g0010, scaffold00015.85, scaffold00017.258, pa_MA_14300g0010, StAAP2, StAAP1, StAAP6, StAAP5, StAAP4, StAAP3, ZmAAAP29, OsAAP19, StAAP8, StAAP7, ZmAAAP69, OsAAP15, ZmAAAP66, ZmAAAP22, OsAAP16, ZmAAAP67, OsAAP17, ZmAAAP64, OsAAP18, ZmAAAP21, ZmAAAP65, OsAAP11, OsAAP12, pa_MA_902657g0010, OsAAP13, OsAAP14, AtAAP5, AtAAP6, AtAAP7, scaffold0016.169, AtAAP8, OsAAP10, AtAAP1, AtAAP2, AtAAP3, scaffold00040.261, AtAAP4, OsAAP1, pa_MA_74043g0010, OsAAP3, OsAAP2, scaffold00166.21, OsAAP5, scaffold00001.289, OsAAP4, scaffold00033.54, scaffold00029.259, OsAAP7, scaffold00002.493, OsAAP6, ZmAAAP36, ZmAAAP33, OsAAP8, ZmAAAP09, ZmAAAP46, ZmAAAP45, pa_MA_402129g0010, ZmAAAP52, pa_MA_6860g0010, scaffold00061.89, scaffold00059.251, pa_MA_101691g0010, scaffold00005.76

ion binding 3 OsAAP13, ZmAAAP09, ZmAAAP69

ATPase activity 3 OsAAP13, ZmAAAP09, ZmAAAP69

helicase activity 3 OsAAP13, ZmAAAP09, ZmAAAP69

Biological Process (BP)

GO #Seqs

transport 5 Sphfalx0193s0030, Pp3c9_4450, Sphfalx0007s0047, Sphfalx0018s0093, Sphfalx0075s0052

transmembrane transport 2 127270, 413158

DNA metabolic process 3 OsAAP13, ZmAAAP09, ZmAAAP69

response to stress 3 OsAAP13, ZmAAAP09, ZmAAAP69

Cellular Component (CC)

GO #Seqs

cellular_component 46 Sphfalx0065s0028, Mapoly0130s0012, SmAAP9A, Pp3c17_11220, SmAAP9B, Pp3c21_14080, 98385, SmAAP10, Sphfalx0075s0052, Mapoly0134s0048, 36205, Sphfalx0007s0033, 55902, Mapoly0107s0041, 173454, Sphfalx0018s0093, 24967, 127270, 98878, Sphfalx0015s0259, 426884, Sphfalx0065s0034, Sphfalx0014s0033, Sphfalx0257s0017, Mapoly0040s0055, Sphfalx0333s0002, Sphfalx0193s0030, Mapoly0170s0014, Pp3c9_4450, Sphfalx0000s0509, Sphfalx0007s0047, Mapoly0052s0005, 29311, Mapoly0004s0095, SmAAP9C, 413158, Pp3c6_1540, Sphfalx0026s0089, PpAAP9A, pa_MA_43770g0010, scaffold00071.167, pa_MA_165784g0010, scaffold00071.161, scaffold00033.53, OsAAP9, scaffold00071.165， Pp3c11_19940， Sphfalx0193s0031， Sphfalx0168s0015

plasma membrane 104 Sphfalx0084s0062, Pp3c23_12700, 127260, Sphfalx0084s0063, Pp3c3_11320, Sphfalx0002s0399, Sphfalx0362s0007, Pp3c8_19000, Sphfalx0362s0005, Pp3c14_9480, Sphfalx0104s0057, Sphfalx0007s0128, 156907, Sphfalx0616s0001, Sphfalx0040s0186, Sphfalx0005s0083, Pp3c13_12390, Sphfalx0007s0031, Sphfalx0005s0085, Sphfalx0003s0314, Mapoly0032s0115, Mapoly0032s0116, 75458, 99162, Sphfalx0025s0047, 31400, Mapoly0047s0067, Sphfalx0013s0130, PpAAP9B, ZmAAAP17, ZmAAAP18, ZmAAAP59, ZmAAAP14, ZmAAAP55, ZmAAAP56, ZmAAAP54, ZmAAAP60, pa_MA_889393g0010, scaffold00015.85, scaffold00017.258, pa_MA_14300g0010, StAAP2, StAAP1, StAAP6, StAAP5, StAAP4, StAAP3, ZmAAAP29, OsAAP19, StAAP8, StAAP7, ZmAAAP69, OsAAP15, ZmAAAP66, ZmAAAP22, OsAAP16, ZmAAAP67, OsAAP17, ZmAAAP64, OsAAP18, ZmAAAP21, ZmAAAP65, OsAAP11, OsAAP12, pa_MA_902657g0010, OsAAP13, OsAAP14, AtAAP5, AtAAP6, AtAAP7, scaffold0016.169, AtAAP8, OsAAP10, AtAAP1, AtAAP2, AtAAP3, scaffold00040.261, AtAAP4, OsAAP1, pa_MA_74043g0010, OsAAP3, OsAAP2, scaffold00166.21, OsAAP5, scaffold00001.289, OsAAP4, scaffold00033.54, scaffold00029.259, OsAAP7, scaffold00002.493, OsAAP6, ZmAAAP36, ZmAAAP33, OsAAP8, ZmAAAP09, ZmAAAP46, ZmAAAP45, pa_MA_402129g0010, ZmAAAP52, pa_MA_6860g0010, scaffold00061.89, scaffold00059.251, pa_MA_101691g0010, scaffold00005.76

plastid 7 270979, Sphfalx0616s0001, 127260, Sphfalx0084s0063, Pp3c13_12390, Sphfalx0002s0399, Sphfalx0013s0130

nuclear envelope 1 AtAAP3
