# Supplementary material for: Evolutionary significance of amino acid permease transporters in 17 plants from Chlorophyta to Angiospermae
Source: BMC Genomics. 2020 Jun 5;21:391. doi: 10.1186/s12864-020-6729-3 (PMC7275304; doi:10.1186/s12864-020-6729-3)
Supplement: Supplementary file 9 — Additional file 9. Painting a GO annotation results using R code. [file 12864_2020_6729_MOESM9_ESM.docx]

go <- read.csv("Table S5.csv")

library(ggplot2)

go<-within(go,{

GO.Name<-factor(GO.Name,levels=c("Cellular_component",

"Plasma membrane",

"Plastid",

"Nuclear envelope",

"Transport",

"Transmembrane transport",

"DNA metabolic process",

"Response to stress",

"Transmembrane transporter activity",

"Ion binding",

"ATPase activity",

"Helicase activity"

))

})

CPCOLS <- c("#8DA1CB", "#FD8D62", "#66C3A5")

## shorten the names of GO terms

shorten_names <- function(x, n_word=4, n_char=40){

if (length(strsplit(x, " ")[[1]]) > n_word || (nchar(x) > 40))

{

if (nchar(x) > 40) x <- substr(x, 1, 40)

x <- paste(paste(strsplit(x, " ")[[1]][1:min(length(strsplit(x," ")[[1]]), n_word)],

collapse=" "), "...", sep="")

return(x)

}

else

{

return(x)

}

}

labels=(sapply(

levels(go$GO.Type)[as.numeric(go$GO.Type)],

shorten_names))

names(labels) = rev(1:nrow(go))

windowsFonts(myFont=windowsFont("TimesNewRoman"))

p <- ggplot(data=go, aes(x=GO.Name, y=log10(Numbers.of.Seqs), fill=GO.Type)) +

geom_bar(stat="identity", width=0.9) + coord_flip()+

scale_fill_manual(values = CPCOLS,labels = c('Biological Process','Cellular Component','Molecular Function')) + theme_bw() +

scale_x_discrete(labels=labels) +

labs(x = "GO Term", y = "Number of Genes (log10)") +

theme(axis.text=element_text(color="black")) +

theme(axis.text.y = element_text(size=10,family="myFont", face= "italic", angle=20), axis.text.x = element_text(size=10,family="myFont")) +

# size=10,family="myFont",angle=15

theme(legend.position=c(0.7,0.9)) +

theme(legend.text = element_text(size = 10, family="myFont")) +

theme(legend.key.size = unit(0.3,'cm')) +

geom_text(aes(label = Numbers.of.Seqs, vjust = 0.35,hjust = 0), size=3.5, family="myFont") +

guides(fill=guide_legend(title='Type')) +

theme(panel.border = element_blank()) +

theme(panel.grid =element_blank()) +

theme(axis.ticks.y = element_blank()) +

theme(axis.line = element_line(size=0.5, colour = "black"))

p

p + ylim(0,3) +

theme(axis.title.y = element_text(size=12, family="myFont", color="black", face= "bold")) +

theme(axis.title.x = element_text(size=12, family="myFont", color="black", face= "bold")) +

theme(legend.title = element_text(size=12, family="myFont", color="black", face= "bold"))
